# PANoptosis gene signatures identify candidate biomarkers and genetically associated genes in COPD

**DOI:** 10.7189/jogh.16.04302

**Published:** 2026-10-30

**Authors:** Hongqing Chen, Jindao Wang, Dan Yang, Ting Ma, Liang Wang, Zongren Wan, Rong Zhu

**Affiliations:** 1 Huaian Clinical College of Xuzhou Medical University, Xuzhou, China; 2 The Affiliated Huai’an No.1 People’s Hospital of Nanjing Medical University, Huai’an, China; 3 The Affiliated Huai’an No.1 People’s Hospital of Nanjing Medical University, Huai’an Clinical College of Xuzhou Medical University, Huai’an, China

**Keywords:** chronic obstructive pulmonary disease, PANoptosis, biomarkers, Mendelian randomisation, immune cell infiltration, transcriptome analysis

## Abstract

**Background:**

PANoptosis contributes to chronic obstructive pulmonary disease (COPD) pathogenesis. We aimed to identify PANoptosis-related gene signatures as candidate biomarkers and evaluate their genetic associations with COPD risk.

**Methods:**

Three transcriptomic data sets were integrated to identify differentially expressed PANoptosis-related genes in COPD, followed by enrichment analyses and immune infiltration profiling using CIBERSORT and single-sample gene set enrichment analysis. We selected feature genes using machine learning and evaluated their discriminative performance. We used Mendelian randomisation (MR) and multivariable MR (MVMR) to assess genetic associations with COPD risk, using the forced expiratory volume in one second/forced vital capacity (FEV1/FVC) ratio as a proxy for airflow limitation and adjusting for smoking. We validated candidate genes by quantitative real-time polymerase chain reaction (qRT-PCR) in human lung tissues from COPD patients and controls.

**Results:**

Seven PANoptosis-related genes (*IL1B*, *IRF1*, *NTRK2*, *PIK3CG*, *SFN*, *TNFSF10*, and *UACA*) were differentially expressed in COPD. Immune profiling suggested immune microenvironment remodelling, characterised by increased memory B cells, activated mast cells, and CD4^+^ T helper cell subsets, with decreased M2 macrophages, monocytes, and neutrophils. *PIK3CG* and *IL1B* were positively associated with adaptive immune-related signals, whereas *UACA* showed predominantly negative immune correlations. *PIK3CG* showed moderate and variable discriminative performance, with an area under the curve of 0.775 in the training data set and 0.614 and 0.743 in validation data sets. The MR and MVMR analyses suggested that *IRF1*- and *UACA*-mediated increases in FEV1/FVC were associated with lower COPD risk independently of smoking. Additionally, qRT-PCR confirmed *PIK3CG* up-regulation in COPD lung tissues, whereas *IRF1* and *UACA* showed no remarkable expression changes.

**Conclusions:**

Our results suggest that *PIK3CG* is a computationally and experimentally supported biomarker for COPD, while *IRF1* and *UACA* act as genetically implicated regulators of airflow limitation. Together, these findings show the potential importance of PANoptosis-driven immune microenvironmental remodelling as a therapeutic direction warranting further functional validation.

Chronic obstructive pulmonary disease (COPD) is a major global health burden characterised by persistent respiratory symptoms and progressive airflow limitation [1]. Its prevalence is projected to increase by 112 million cases, reaching 592 million by 2050, driven by population ageing and sustained exposure to risk factors such as smoking and environmental pollutants [2]. Current diagnosis relies mainly on spirometry, complemented by clinical symptom assessment and medical history [3]. Existing treatments, including pharmacotherapy, pulmonary rehabilitation, and oxygen therapy, primarily aim to manage symptoms and prevent exacerbations [4] but do not fully address disease complexity, including chronic inflammation, airway remodelling, and comorbidities [5]. These limitations highlight the need for improved strategies, including reliable biomarkers for disease stratification and more targeted therapeutic approaches [6].

PANoptosis, a coordinated lytic cell death pathway that integrates pyroptosis, apoptosis, and necroptosis through multiprotein complexes called PANoptosomes [7], has been implicated in COPD pathogenesis [8]. It may promote inflammation, tissue damage, and disease progression, with key mediators such as receptor-interacting serine-threonine protein kinases 1 and 3 showing elevated expression in COPD patients [9]. In addition, cigarette smoke-induced apoptosis and activation of the canonical inflammasome pathway may exacerbate tissue destruction [10]. Endothelial apoptosis and dysregulated interferon signalling further contribute to disease progression [11].

Despite increasing evidence implicating PANoptosis in COPD, reliable PANoptosis-related biomarkers remain limited. For example, *PYCARD* has been identified using Gene Expression Omnibus (GEO) data sets, but its diagnostic performance was modest, and validation was limited [12]. More broadly, transcriptomic studies have reported diverse candidate genes and pathways related to inflammatory, immune-related, and extracellular matrix-associated processes [13–15]. However, the overlap between studies remains limited. This inconsistency is largely attributable to differences in tissue source, disease stage, and clinical heterogeneity, which may influence gene expression profiles [16]. These challenges highlight the need for integrative, multi-cohort approaches to improve the robustness and reproducibility of biomarker identification.

In this study, we focused on the potential relevance of PANoptosis-related genes to COPD biomarker discovery and disease-related biological interpretation. Although PANoptosis has been implicated in inflammatory and immune-mediated lung injury, its relationship with COPD-associated transcriptional alterations and immune microenvironmental changes remains incompletely understood. We therefore examined whether PANoptosis-related gene expression patterns were associated with immune-related features in COPD and whether selected genes showed genetic associations with airflow limitation and COPD risk. By integrating transcriptomic evidence, genetic association analyses, and tissue-level validation, we sought to provide a more comprehensive assessment of the potential relevance of PANoptosis-related genes in COPD.

Ultimately, we aimed to identify reliable candidate diagnostic markers based on gene expression and to discover genetically supported candidate targets that may influence COPD susceptibility.

## METHODS

We conducted and reported this study in accordance with the STROBE [20] and STROBE-MR [21] reporting guidelines (Tables S1 and S2 in the **Online Supplementary Document**).

We employed a multi-stage integrative analysis pipeline to identify PANoptosis-related gene signatures in COPD (Figure S1 and Table S3 in the **Online Supplementary Document**). We included five GEO datasets; GSE151052, GSE37768, and GSE103174 were merged as the discovery cohort, whereas GSE38974 and GSE106986 served as external validation cohorts (**Table 1**). After probe annotation, normalisation, and batch correction, we identified differentially expressed genes (DEGs) between COPD and control samples and intersected them with a previously reported PANoptosis-related gene set. Functional enrichment analyses were performed using Gene Ontology and Kyoto Encyclopedia of Genes and Genomes pathways, and immune infiltration was assessed using ssGSEA and CIBERSORT. Spearman correlation analysis was used to evaluate associations between candidate genes and immune cell-related signals, and consensus clustering was performed to identify COPD molecular subtypes. Feature genes were selected using SVM-RFE, LASSO regression, and random forest, and their discriminative performance was evaluated in the discovery and validation cohorts using receiver operating characteristic curves. Public GEO datasets are de-identified and publicly available and therefore did not require additional ethical approval.

**Table 1:** Summary of discovered and validated GEO cohorts for COPD*

ID	Platform	Total, n	COPD, n	Control, n
GSE151052	GPL17556	117	77	40
GSE37768	GPL570	38	18	20
GSE103174	GPL13667	53	37	16
GSE38974	GPL4133	32	23	9
GSE106986	GPL13497	19	14	5

COPD – chronic obstructive pulmonary disease, GEO – Gene Expression Omnibus

*All data sets comprised lung tissue samples and were based on mRNA array data.

To evaluate genetic associations, we performed univariable two-sample MR using the FEV1/FVC ratio as a genetic proxy for airflow limitation and COPD as the outcome. *Cis*-acting instrumental variables near target genes were selected based on association strength, minor allele frequency, F-statistic, and linkage disequilibrium criteria. Inverse-variance weighted analysis was used as the primary method, with complementary sensitivity analyses for heterogeneity, pleiotropy, and influential variants. We further conducted MVMR analyses by incorporating lifetime smoking and current smoking phenotypes from the US Million Veteran Program (MVP), as covariates to adjust for potential confounding. All genome-wide association study (GWAS) datasets for FEV1/FVC and COPD variants were restricted to European ancestry populations and did not require additional ethical approval.

We further assessed transcriptional expression levels of key candidate genes in human lung tissue samples from six patients with COPD and nine controls by qRT-PCR. GAPDH was used as the endogenous control, and relative gene expression was calculated using the 2−ΔΔCt method. We collected human lung tissue samples prospectively after approval. We obtained written informed consent from all participants before sample collection.

Additional technical details, including software parameters, data sources, and primer sequences, are provided in the **Online Supplementary Document**.

## RESULTS

### Identification of differentially expressed PANoptosis-related genes in COPD

To identify DEGs associated with COPD, we analysed transcriptomic profiles from 132 patient samples and 76 control samples in the merged data set. We observed distinct clustering patterns across data sets before removing batch effects. After batch effect removal, technical variation across data sets was effectively minimised, resulting in homogeneous integration in which samples clustered harmoniously based on biological attributes rather than cohort origin (Figure S2, Panels A–D in the **Online Supplementary Document**).

Differential expression screening after data normalisation identified a total of 361 high-confidence DEGs, comprising 224 significantly up-regulated and 137 down-regulated transcripts in the COPD group relative to the controls (Figure S2, Panel E and Table S4 in the **Online Supplementary Document****)**. The hierarchical clustering heatmap illustrates the expression architecture and transcriptional fidelity of the top 20 altered DEGs across individual biological replicates (Figure S2, Panel F in the **Online Supplementary Document**).

To delineate the functional landscapes governed by these pathological transcripts, we systematically performed Gene Ontology and Kyoto Encyclopedia of Genes and Genomes enrichment analyses. The identified DEGs were predominantly enriched in critical biological processes governing leukocytes and cell-cell adhesion, structural constituents of the collagen-containing extracellular matrix, and foundational molecular activities driven by cytokine and chemokine signalling (**Figure 1**, Panel A; Table S5 in the **Online Supplementary Document**). Consistently, Kyoto Encyclopedia of Genes and Genomes pathway topology mapping revealed that the most profoundly disrupted cascades were heavily channelled into the cytokine-cytokine receptor interaction, tumour necrosis factor (TNF) signalling pathway, and the interleukin-17 inflammatory axis, strongly indicating a localised hyper-inflammatory state and immune microenvironmental remodelling in the lungs of COPD patients (**Figure 1**, Panel B; Table S6 in the **Online Supplementary Document**).

**Figure 1:**
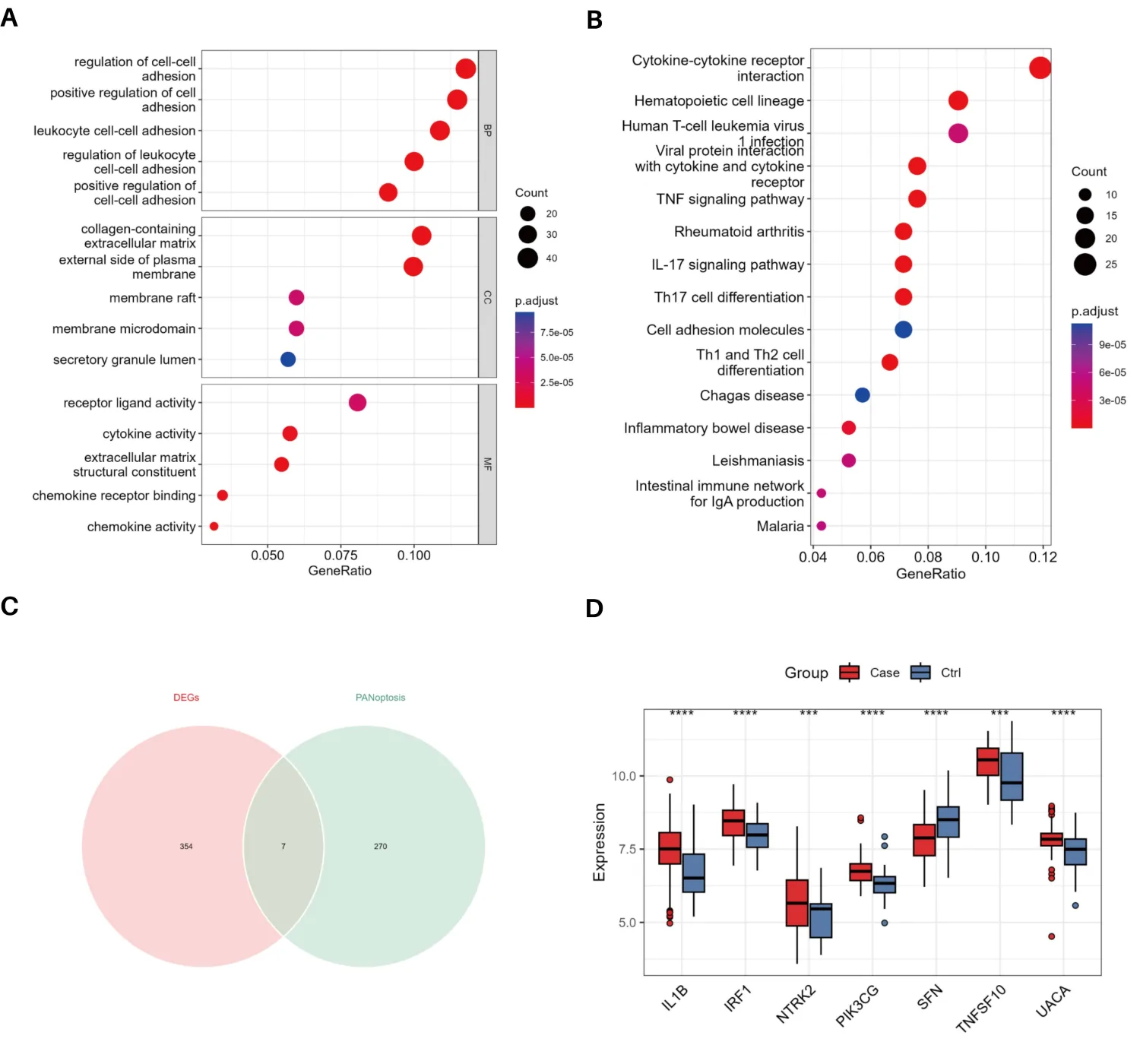
Functional enrichment landscapes of DEGs and identification of PANoptosis-related hub targets in COPD. **Panel A.** Bubble plot of enriched GO terms across BPs, CCs, and MFs branches. **Panel B.** Bubble plot of enriched KEGG pathways. **Panel C.** Venn diagram showing the overlap between COPD-associated DEGs and PANoptosis-related genes. **Panel D.** Boxplots showing the expression of seven overlapping genes in COPD and control samples. For Panels A and B, the colour gradient (shifting from blue to red) represents the ascending order of statistical significance, while the dot size corresponds to the count of enriched genes mapped to each specific term or pathway. Statistical significance is denoted as: *P < 0.05, **P < 0.01, ***P < 0.001, and ****P < 0.0001. BP – biological process, CC – cellular component, COPD – chronic obstructive pulmonary disease, DEG – differentially expressed gene, GO – Gene Ontology, KEGG – Kyoto Encyclopedia of Genes and Genomes, MF – molecular function, Ctrl – control, IgA – immunoglobulin A, IL-17 – interleukin-17, Th1 – T helper 1, Th2 – T helper 2, Th17 – T helper 17, TNF – tumour necrosis factor.

To identify differentially expressed PANoptosis-related genes, we intersected 361 DEGs with 277 PANoptosis-related genes and identified seven overlapping genes, including interleukin 1 beta (*IL1B*), interferon regulatory factor 1 (*IRF1*), neurotrophic tyrosine kinase receptor 2 (*NTRK2*), phosphatidylinositol-4,5-bisphosphate 3-kinase catalytic subunit gamma (*PIK3CG*), stratifin (*SFN*), TNF superfamily member 10 (*TNFSF10*), and uveal autoantigen with coiled-coil domains and ankyrin repeats (*UACA*) (**Figure 1**, Panel C). Notably, six of these core genes (*IL1B*, *IRF1*, *NTRK2*, *PIK3CG*, *TNFSF10*, and *UACA*) were prominently up-regulated in the lung tissues of COPD patients, whereas *SFN* was uniquely down-regulated (**Figure 1**, Panel D). To explore the potential functions and clinical heterogeneity of these seven core PANoptosis genes in COPD, we performed unsupervised clustering and feature selection analyses.

### Stratification of COPD patients based on PANoptosis-related gene expression profiles

To elucidate the role of PANoptosis-related genes in the clinical and biological heterogeneity of COPD, we performed unsupervised consensus clustering in the integrated cohort based on baseline expression profiles of the seven intersected hub DEGs. Cross-validation of the cumulative distribution function curves and tracking area changes established k = 3 as the optimal partition number, effectively stratifying the patient population into three distinct, highly stable molecular sub-clusters (Figure S3, Panels A–D in the **Online Supplementary Document**). Principal component analysis and t-distributed stochastic neighbour embedding showed clear separation into three subgroups, with 68 patients in Cluster 1, 41 in Cluster 2, and 23 in Cluster 3 (Figure S3, Panels E–F in the **Online Supplementary Document**). These findings highlight the heterogeneity in the COPD patient cohort and suggest potential stratification based on the molecular signatures of PANoptosis-related genes.

### Deciphering the immune infiltration landscapes and PANoptosis-driven transcriptomic correlations in COPD

To characterise differences in the immune microenvironment between COPD patients and controls, we first captured the relative enrichment scores of 28 immune cell lineages *via* ssGSEA. We then visualised the overall clustering of these 28 immune fractions across all integrated sample replicates in a comprehensive hierarchical clustering heatmap. Further comparative group-level screening revealed that 17 of these 28 immune cell types manifested profound statistical divergence between the COPD cases and controls. To strengthen methodological validation and cross-verify these cellular configurations, we performed an independent tissue-level deconvolution using the standard CIBERSORT algorithm (Figure S4, Panels A–C in the **Online Supplementary Document**). Consistent with the overall hyper-inflammatory trends mapped by the ssGSEA framework, the refined CIBERSORT outputs showed severe homeostatic disruption and polarised lymphocyte recruitment within the COPD cohorts, further supporting a severely remodelled myeloid-lymphoid axis in the lungs of COPD patients.

To systematically test whether the deregulated PANoptosis machinery actively orchestrates this distinctive cellular recruitment, we deployed Spearman correlation pipelines. The resulting interaction revealed intricate, high-density crosstalk networks linking the seven core PANoptosis-related hub DEGs to fluctuating immune cell fractions under both ssGSEA and CIBERSORT (Figure S4, Panels D and E in the **Online Supplementary Document**). Canonical inflammatory drivers (*IRF1*, *IL1B*, and *PIK3CG*) showed tight positive coupling with active immune effectors, whereas UACA showed reversed correlation trends, suggesting that this seven-gene signature plays a pivotal role in dictating immune microenvironment remodelling.

### Heterogeneous patterns of immune infiltration and PANoptosis signature expressions across COPD molecular subtypes

To interrogate the immunological heterogeneity driving COPD, we compared the microenvironmental landscape among the three identified patient clusters using ssGSEA. The analysis revealed that these molecular subtypes possessed highly polarised immune infiltration patterns (Figure S5, Panels A and C in the **Online Supplementary Document**). Cluster 1 presented a profound hyper-inflammatory posture, characterised by a generalised up-regulation of adaptive effector lymphocytes and functional myeloid lineages. In contrast, Cluster 3 showed a quiescent, universally suppressed immune profile, whereas Cluster 2 represented an intermediate transition state. Furthermore, we mapped all seven PANoptosis-related DEGs across these subtypes. All seven hub genes exhibited robust, high-confidence statistical variations among the three clusters (Figure S5, Panels B and D in the **Online Supplementary Document**). Canonical inflammatory drivers, including *IL1B*, *IRF1*, *PIK3CG*, and *TNFSF10*, were uniquely hyperactivated within the immune-high Cluster 1 niche. Conversely, counter-agents such as *UACA* and *NTRK2* displayed distinct expression peaks in Clusters, demonstrating a synchronised dual-axis disruption in COPD. These data suggest that the COPD subtypes differ in estimated immune infiltration and PANoptosis-related gene expression patterns.

### Screening and validation of the PANoptosis-based diagnostic machine-learning signature

To extract a robust diagnostic signature from the seven PANoptosis-related candidate DEGs, we integrated three complementary machine-learning algorithms (LASSO, SVM-RFE, and RF). All three computational workflows converged on the same seven-gene framework, demonstrating complete programmatic consensus (**Figure 2**, Panels A–G). To prioritise features with optimal diagnostic efficacy, ROC analysis was performed, identifying *PIK3CG* (area under the curve (AUC) = 0.775), *IL1B* (AUC = 0.723), *UACA* (AUC = 0.716), and *SFN* (AUC = 0.706) as the core diagnostic hubs with acceptable discriminative capacities (**Figure 2**, Panel H). External validation across different cohorts captured dataset-specific variations (Figure S6 in the **Online Supplementary Document**). In the GSE38974 cohort, diagnostic performance was similar in *PIK3CG* (AUC = 0.614), *IL1B* (AUC = 0.783), *UACA* (AUC = 0.758), and *SFN* (AUC = 0.754). In the independent GSE106986 cohort, the predictive performance was replicated, yielding varying AUC values for *PIK3CG* (AUC = 0.743), *IL1B* (AUC = 0.600), *UACA* (AUC = 0.729), and *SFN* (AUC = 0.843). These results indicate that while these candidate genes show moderate diagnostic value individually, their performance varies with the clinical heterogeneity of the validation cohorts (such as the drop in *PIK3CG* in GSE38974 and *IL1B* in GSE106986). Rather than dismissing their utility, this variability underscores the limitations of relying on any single biomarker and strongly supports integrating them into a composite multi-gene diagnostic framework to achieve more stable and reliable predictive power. Protein-protein interaction network and pairwise correlation analyses further confirmed that these four candidate genes occupied central topological hubs, suggesting a coordinated regulatory architecture in COPD (Figure S7 in the **Online Supplementary Document**).

**Figure 2:**
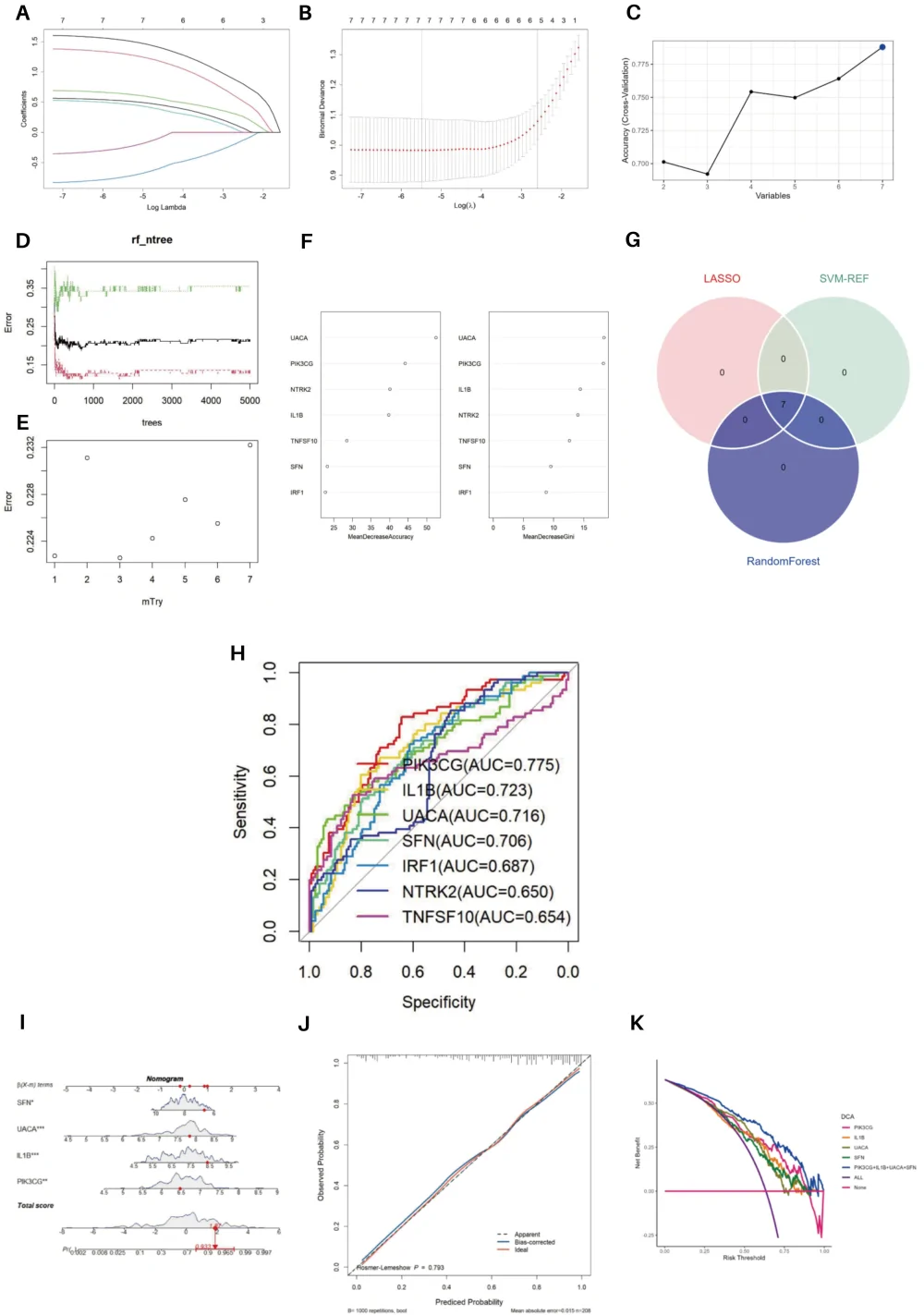
Identification, feature selection, and clinical evaluation of the PANoptosis-derived diagnostic signature based on consensus machine learning. **Panel A.** LASSO coefficient trajectories. **Panel B.** 10-fold cross-validation profile for selecting the optimal *lambda* value. **Panel C.** 5-fold cross-validation accuracy trajectory across feature numbers using SVM-RFE. **Panel D.** RF error rates according to the number of trees. **Panel E.** RF error rates across different *mtry* values. **Panel F.** Gene importance in the RF model based on mean decrease in accuracy and Gini coefficient. **Panel G.** Venn diagram showing overlapping feature genes identified by LASSO, SVM-RFE, and RF. **Panel H.** ROC curves of the feature genes in the merged dataset. **Panel I.** Diagnostic nomogram combining feature genes *SFN*, *UACA*, *IL1B*, and *PIK3CG*. **Panel J.** Calibration curve for the nomogram. **Panel K.** DCA of the individual genes and composite model across different threshold probabilities. DCA – decision curve analysis, LASSO – least absolute shrinkage and selection operator, RF – random forest, ROC – receiver operating characteristic, SVM-RFE – support vector machine-based recursive feature elimination, AUC – area under the curve.

Using these four refined core variables, SFN, UACA, IL1B, and PIK3CG, we established a diagnostic nomogram. Methodological evaluation using the calibration curve showed excellent agreement between nomogram-predicted probabilities and actual observations. Furthermore, decision curve analysis confirmed that the combined nomogram offered superior clinical net benefits over any individual gene across a wide range of threshold probabilities (**Figure 2**, Panels I–K).

Collectively, these findings suggest that *PIK3CG*, *IL1B*, *UACA*, and *SFN* may serve as candidate biomarkers for COPD, although their discriminative performance remains moderate.

### Association of feature genes with immune cell subsets in the merged data set

To delineate the immunological relevance of the four core diagnostic hubs, we implemented dual-algorithm evaluation *via* ssGSEA and CIBERSORT in the merged cohort.

*PIK3CG* showed broad positive correlations with adaptive immune-related cell subsets; ssGSEA established robust positive links with immature B cells, activated B cells, activated CD4^+^/CD8^+^ T cells, and mast cells, contrasted by inverse correlations with neutrophils, plasmacytoid dendritic cells (DCs), and activated DCs. CIBERSORT mirrored this pattern, validating positive associations with M1 macrophages, T follicular helper (Tfh) cells, and γδ T cells, alongside negative correlations with neutrophils, monocytes, and activated DCs (Figure S8, Panels A and B in the **Online Supplementary Document**).

*IL1B* exhibited a pronounced pro-inflammatory polarisation profile. ssGSEA analysis showed widespread positive correlations with multiple immune cell subsets, particularly mast cells, eosinophils, type 1 T helper cells, activated CD4^+^/CD8^+^ T cells, and Tfh cells, with only limited negative correlations. CIBERSORT also confirmed strong positive convergence with activated mast cells, M1 macrophages, and Tfh cells, whereas resting mast cells and plasma cells showed strong negative correlations (Figure S8, Panels C and D in the **Online Supplementary Document**).

Unlike *PIK3CG* and *IL1B*, *UACA* displayed a predominantly repressive immune signature. In ssGSEA analysis, *UACA* was negatively correlated with activated DCs, monocytes, macrophages, central memory CD4^+^/CD8^+^ T cells, and several NK/T-cell subsets, whereas it showed only weak positive correlations with type 2 T helper cells and mast cells (Figure S8, Panel E in the **Online Supplementary Document**). CIBERSORT corroborated this immunological silencing, showing negative correlations with M0 macrophages, eosinophils, monocytes, and activated NK cells, while the strongest positive correlation was with resting mast cells (Figure S8, Panel F in the **Online Supplementary Document**).

*SFN* demonstrated different immune association patterns between the two algorithms. In ssGSEA analysis, *SFN* was positively correlated with central memory CD4^+^/CD8^+^ T cells, activated DCs, immature DCs, macrophages, and neutrophils, but negatively correlated with immature B cells, activated B cells, and effector memory CD8^+^ T cells. In CIBERSORT analysis, *SFN* was positively associated with M0 macrophages, neutrophils, eosinophils, plasma cells, and activated NK cells, whereas resting mast cells, memory B cells, and resting natural killer (NK) cells showed negative correlations (Figure S8, Panels G and H in the **Online Supplementary Document**).

Taken together, *PIK3CG* and *IL1B* are closely associated with enhanced adaptive and pro-inflammatory immune activation, whereas *UACA* serves as an overall immune-suppressive indicator. *SFN* showed mixed associations involving both innate and adaptive immune cell subsets. These findings support the close relationship between PANoptosis-related genes and immune microenvironment remodelling in COPD. Having established the relationship between these candidate biomarkers and the immune microenvironment, we next performed MR analysis to assess their genetic associations with COPD risk.

### MR analysis of PANoptosis-related genes for COPD risk

To assess the effects of the feature genes on COPD-related lung function and disease risk, we conducted MR analysis using FEV1/FVC as a genetic proxy for airflow limitation. Among the seven candidate genes, four met our baseline inclusion criterion of having more than two valid instrumental variables (IVs): *IRF1* (17 IVs), *PIK3CG* (3 IVs), *TNFSF10* (3 IVs), and *UACA* (20 IVs) (Table S7 in the **Online Supplementary Document****)**.

The primary inverse-variance weighted meta-analysis revealed that genetically instrumented lung function (FEV1/FVC) mediated by specific feature genes exerted distinct causal effects on COPD susceptibility (**Figure 3**, Panel A). Notably, genetically predicted increases in *IRF1*-mediated FEV1/FVC were dynamic and robustly associated with a drastically reduced risk of COPD, presenting as the most prominent protective factor among the candidates (odds ratio (OR) = 0.19; 95% confidence interval (CI) = 0.12–0.31, *P* = 1.49 × 10^−11^). Similarly, a significant protective causal vector was discernible for *UACA*-mediated FEV1/FVC (OR = 0.29; 95% CI = 0.19–0.43; *P* = 7.39 × 10^−10^). Conversely, we found no statistically significant causal links between *TNFSF10* or *PIK3CG* and COPD. This lack of statistical significance was likely attributable to restricted statistical power, given the paucity of available IVs for these two loci (3 IVs each). Notably, Steiger directional tests confirmed that the vast majority of the utilised single nucleotide polymorphisms collectively supported the expected causal direction from lung function (FEV1/FVC) to COPD risk (Table S7 in the **Online Supplementary Document**).

**Figure 3:**
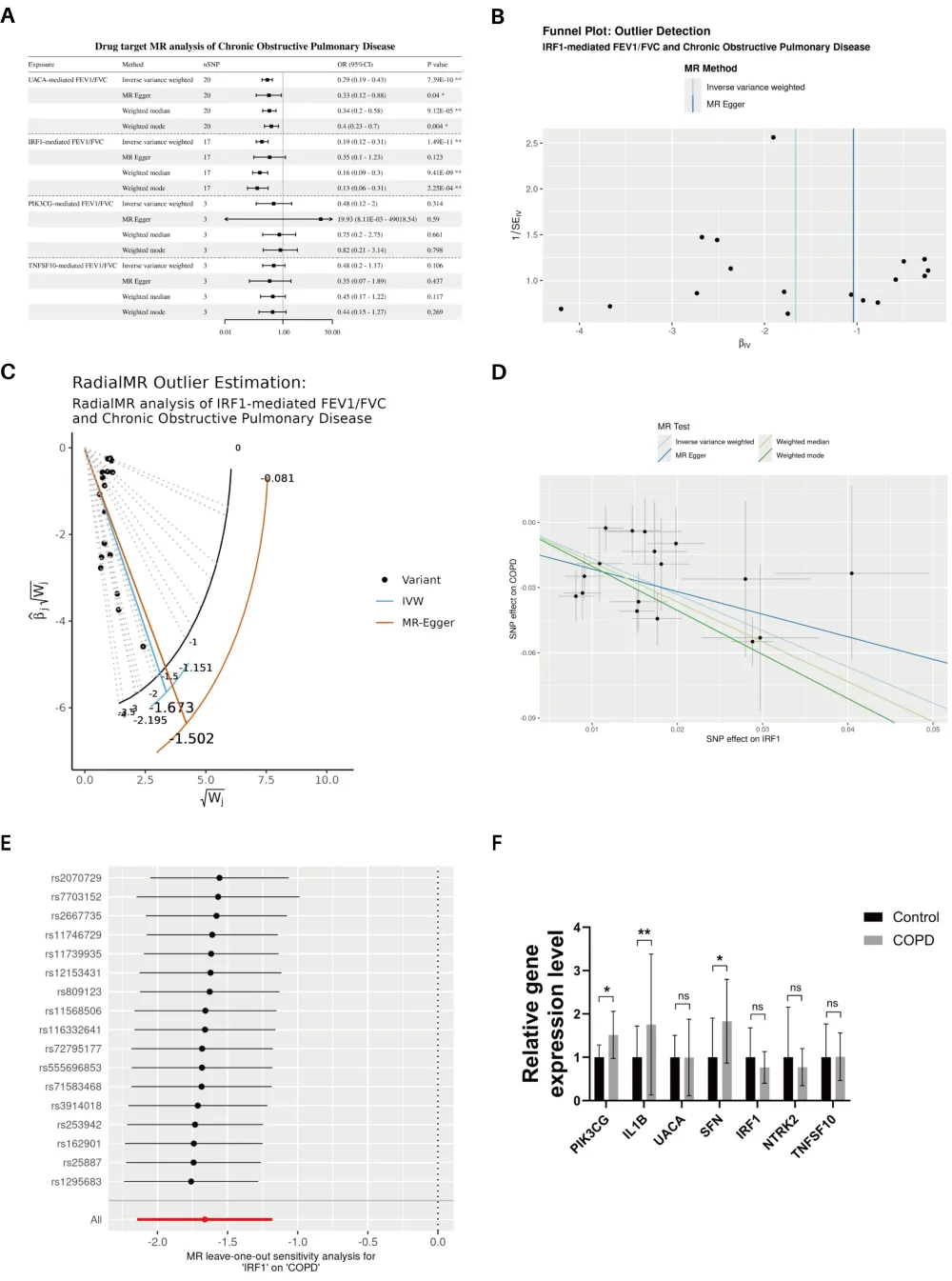
MR analysis and experimental validation of key genes. **Panel A.** Forest plot of MR estimates for PANoptosis-related gene-mediated FEV1/FVC and COPD risk. **Panel B.** Funnel plot for *IRF1*-mediated FEV1/FVC and COPD risk. **Panel C.** Radial MR plot for *IRF1*-mediated FEV1/FVC and COPD risk. **Panel D.** Scatter plot of SNP effects on *IRF1*-mediated FEV1/FVC and COPD risk across MR models. **Panel E.** Leave-one-out sensitivity analysis for *IRF1*-mediated FEV1/FVC and COPD risk. **Panel F.** qRT-PCR analysis of *PIK3CG*, *IL1B*, *UACA*, *SFN*, *IRF1*, *NTRK2*, and *TNFSF10* in lung tissue samples from COPD patients and non-COPD controls. Data are presented as mean (SD). Statistical significance was determined using an unpaired *t*-test. *P < 0.05, **P < 0.01. CI – confidence interval, FEV1 – forced expiratory volume in 1 second, FVC – forced vital capacity, IVW – inverse-variance weighted, MR – Mendelian randomisation, ns – non-significant, SD – standard deviation, SNP – single nucleotide polymorphism, qRT-PCR – quantitative real-time polymerase chain reaction, IV – instrumental variable, nSNP – number of single nucleotide polymorphisms, OR – odds ratio, SE – standard error.

Standard sensitivity metrics further substantiated the validity of our primary causal estimates. Cochran’s Q statistics ruled out any distorting heterogeneity within the inverse-variance weighted models (Cochran’s Q test *P* > 0.05). Except for a potential pleiotropy observed in the *UACA*-mediated FEV1/FVC in COPD (MR-Egger intercept *P* = 0.04), which should be interpreted with caution, no systematic evidence of directional horizontal pleiotropy was captured across the remaining candidate genes (MR-Egger intercept *P* > 0.05). Additionally, no significant outliers were detected based on MR-PRESSO global tests (Global *P* > 0.05). Complementary risk estimates derived from weighted median and weighted mode models yielded directionally concordant effects, reinforcing the epistemic certainty of our primary findings (Tables S8–11 in the **Online Supplementary Document**).

To visually validate these core MR assumptions, we conducted a comprehensive sensitivity diagnostic pipeline. We assessed potential horizontal pleiotropy with funnel plots, screened outliers with radial MR plots, evaluated consistency across complementary frameworks with scatter plots, and cross-verified causal architecture stability with leave-one-out analysis. After removing outliers, we generated sensitivity diagnostic plots for *IRF1* (**Figure 3**, Panels B–E) and *UACA* (Figure S9, Panels A–D in the **Online Supplementary Document**).

Post hoc statistical power analyses further supported the reliability of the findings. All utilised instrumental variables showed sufficient strength, with mean *F*-statistics ranging from 20.4 to 41.6, reducing the likelihood of weak-instrument bias. For the significant associations, statistical power to detect the protective effects of *IRF1*- and *UACA-*mediated increases in FEV1/FVC on COPD exceeded 99.9%. Moreover, the minimum detectable ORs required to achieve 80% statistical power were generally high for these non-significant outcomes, indicating limited ability to detect moderate or weak causal effects under the current sample size (Table S12 in the **Online Supplementary Document**).

### MVMR analysis adjusting for smoking-related genetic effects

To assess whether the observed associations were independent of smoking-related genetic confounding, we performed MVMR analyses using genetically predicted lifetime smoking and current smoking phenotypes as covariates. After adjustment for lifetime smoking, the protective effects of *IRF1*- and *UACA*-mediated increases in FEV1/FVC on COPD remained highly consistent with the primary univariable MR analyses. For *IRF1*, the OR remained stable after smoking adjustment (OR = 0.19; 95% CI = 0.12–0.30, *P* = 5.19 × 10^−6^). Similarly, the protective effect of *UACA*-mediated increases in FEV1/FVC on COPD remained significant (OR = 0.29; 95% CI = 0.19–0.43, *P* = 9.69 × 10^−6^) (Table S13 in the **Online Supplementary Document**).

After adjusting for current smoking, both *IRF1* (OR = 0.19; 95% CI = 0.12–0.31, *P* = 6.81 × 10^−6^) and *UACA* (OR = 0.31; 95% CI = 0.21–0.46; *P* = 1.50 × 10^−5^) maintained strong and significant protective associations with COPD risk, suggesting that these protective effects were not driven by smoking-related genetic confounding. In contrast, MVMR analyses for *PIK3CG* and *TNFSF10* showed unstable conditional *F*-statistics and limited reliability due to the small number of available *cis*-acting IVs. Importantly, MVMR-Egger intercept tests showed no evidence of directional horizontal pleiotropy (*P* > 0.05), while MVMR-Q analyses confirmed no significant heterogeneity across all evaluable models (*P* > 0.05), validating the statistical soundness of our findings (Table S13 in the **Online Supplementary Document**).

This genetic evidence prompted further evaluation of expression patterns at the transcriptional level in clinical samples, since genetic associations may not directly reflect tissue-level expression.

### Validation of feature gene alterations in patients with COPD

To validate the expression patterns of the feature genes and further investigate potential discrepancies between genetic causal predictions and actual tissue-level expression, we performed qRT-PCR on lung tissue samples from patients with or without COPD. The results showed that *PIK3CG*, *IL1B*, and *SFN* were significantly up-regulated in COPD lung tissues compared with controls (all *P* < 0.05) (**Figure 3**, Panel F). However, *IRF1* and *UACA* did not show significant differential expression, indicating that their functional roles in COPD require further investigation. Collectively, experimental validation supports *PIK3CG* as a candidate biomarker, while also highlighting the complexity and the inherent discrepancy between germline genetic associations and the actual transcriptional landscape of *IRF1* and *UACA* in diseased lung tissue.

## DISCUSSION

Distinguishing reliable diagnostic biomarkers from genetically implicated genes remains a challenge in COPD research. By combining transcriptomic analysis with MR, we identified candidate biomarkers and genes of interest. Notably, all seven DEGs were consistently prioritised as core features across all three machine learning frameworks. This high degree of overlap, despite the divergent mathematical logics of the algorithms, underscores the predictive robustness and non-redundancy of this candidate gene signature. We identified *PIK3CG* as a candidate biomarker because both transcriptomic analysis and qRT-PCR validation supported its up-regulation in COPD lung tissues. In contrast, *IRF1* and *UACA* showed genetically inferred associations with COPD risk, although their complex expression patterns warrant further investigation.

In this study, *PIK3CG* was up-regulated in COPD lung tissue and showed moderate, variable discriminative performance across cohorts, underscoring its value as a biomarker. *PIK3CG* encodes the catalytic subunit of phosphoinositide 3-kinase gamma (PI3Kγ), a leukocyte-restricted kinase regulating immune cell trafficking and activation [22]. In COPD, PI3Kγ-mediated neutrophil activation [23] and macrophage signalling [24] contribute to persistent inflammation and tissue damage. Activation of the PI3K/Akt pathway further promotes oxidative stress and airway remodelling [25]. Given its immune cell-restricted expression and involvement in systemic inflammation, *PIK3CG* may warrant further evaluation as a potential peripheral blood biomarker, which is more feasible for clinical application than lung tissue-based detection.

Integrated ssGSEA and CIBERSORT analyses showed highly consistent results in defining the chronic inflammatory microenvironment of COPD. For adaptive immunity, both methods confirmed the enrichment of B-cell and T-cell lines, highlighted by increased memory B and Tfh cells. This consensus points to persistent B-cell activation and potential tertiary lymphoid structure formation under continuous antigen stimulation. For cytotoxic immunity, significant elevations in activated CD8^+^ T cells and NK cells suggest persistent tissue damage. In the context of PANoptosis-related gene dysregulation, these cells might drive a vicious cycle of epithelial cell death and immune activation. In contrast, both algorithms showed decreased myeloid cells, including neutrophils and M2 macrophages. This pattern indicates a transition from acute myeloid-driven inflammation to chronic lymphocyte-mediated injury, accompanied by impaired tissue repair capacity. Notably, DCs showed increased recruitment but impaired maturation, evidenced by increased resting DCs in CIBERSORT but decreased activated DCs in ssGSEA. Both analyses also supported mast cell activation, consistent with airway hyperresponsiveness. Overall, this cross-method validation indicates that the Tfh-memory B cell axis, CD8^+^ T/NK cell cytotoxicity, and reduced M2 macrophage repair capacity together constitute the key immunopathological basis of COPD. These immune alterations may provide a potential mechanistic context linking PANoptosis-related genes with chronic inflammation, immune dysregulation, and lung function decline in COPD.

Further correlation analyses demonstrated specific immunoregulatory patterns among the feature genes. We found *PIK3CG* and *IL1B* to be positively correlated with pro-inflammatory and adaptive immune cell subsets in both ssGSEA and CIBERSORT analyses, supporting their roles in sustaining inflammatory immune activation in COPD. Additionally, *IL1B* showed a strong association with activated mast cells, consistent with its established role in amplifying inflammatory signalling and airway hyperresponsiveness. In contrast, *UACA* demonstrated predominantly negative correlations with multiple immune cell subsets, suggesting a potential immunosuppressive or immune-regulatory role in COPD. Furthermore, *SFN* displayed mixed association patterns involving both lymphoid and myeloid immune subsets, indicating a more complex role in immune-cell remodelling and inflammatory-state transition. Collectively, these findings suggest that PANoptosis-related genes may contribute to COPD progression through coordinated regulation of chronic adaptive immune activation, inflammatory amplification, and impaired tissue-repair responses.

In the MR analysis, genetic increases in *IRF1*- and *UACA*-mediated FEV1/FVC were associated with lower COPD risk, suggesting a potential association with lung function preservation. However, qRT-PCR validation did not show significant differences in *IRF1* or *UACA* mRNA expression in lung tissue, suggesting biological complexity. First, MR reflects lifelong germline perturbation across all cell types [26], whereas qRT-PCR provides only a late-stage snapshot in bulk lung tissue. Any protective effect could manifest earlier in disease progression or in specific cell types that are diluted in whole-lung RNA. Second, cell-type specificity is crucial, as *IRF1* or *UACA* may act in epithelial cells, alveolar macrophages, or specific immune subpopulations, whose signals are masked when averaging across heterogeneous lung tissue. Third, regulation may occur mostly at post-transcriptional, translational, or protein-modification levels. For example, *UACA* is known to bind the apoptosome and regulate stress-induced apoptosis rather than through large changes in transcript levels [27]. As a transcription factor, *IRF1* is regulated by phosphorylation, protein stability, and feedback loops [28], and its functional effects may not mirror linear mRNA differences. Moreover, *IRF1* has been implicated in lung vascular regulation and immune signalling, suggesting a broader role in lung immunopathology [29]. Thus, the discrepancy suggests unresolved mechanisms such as temporal dynamics, cell-type specificity, and post-transcriptional regulation, necessitating targeted studies of *IRF1* and *UACA* in COPD.

An important strength of this study is the incorporation of MVMR to distinguish the independent biological effects of lung function and smoking behaviour. By introducing smoking phenotypes from the MVP cohort, which is largely independent of FinnGen and UK Biobank, we reduced potential bias from sample overlap. Notably, analyses using lifetime smoking as the primary covariate and current smoking as the sensitivity covariate showed highly consistent results, suggesting that differences did not substantially influence the main findings in smoking phenotype definition. Although the conditional *F*-statistics for smoking covariates were generally low, this likely reflects the limited genetic correlation between the selected *cis*-acting IVs and smoking-related pathways rather than model invalidity. These findings further support that the protective effects of *IRF1*- and *UACA*-mediated increases in FEV1/FVC on COPD risk were not primarily driven by smoking-related genetic burden. The MVP cohort mainly consists of male veterans, and smoking phenotypes were derived from relatively simplified questionnaire-based classifications, which may limit the generalisability of smoking-related estimates. However, the high consistency of effect directions across different smoking definitions and the absence of substantial horizontal pleiotropy further support the robustness of the main conclusions.

From a translational perspective, PI3Kγ may represent a pharmacologically tractable pathway for further investigation. Selective PI3Kγ inhibitors such as eganelisib (IPI-549) have demonstrated anti-inflammatory activity in preclinical models [30] supporting the potential of PIK3CG-directed modulation in COPD. However, therapeutic intervention in this pathway requires careful calibration. Moderate inhibition may achieve beneficial immunomodulatory effects by attenuating excessive inflammation, whereas excessive or sustained suppression may shift the balance toward immunosuppression. Given that PANoptosis contributes not only to pathological inflammation but also to host defence against pathogens and clearance of damaged cells, indiscriminate inhibition of this pathway may impair antimicrobial immunity and increase susceptibility to infection [31]. Therefore, drug development targeting *PIK3CG* should emphasise precise control of intervention intensity and spatial-temporal specificity, aiming to preserve essential immune functions while reducing pathological inflammation. In contrast, *IRF1*, as a transcription factor, remains challenging to target with conventional small-molecule strategies, and evidence for *UACA* is currently limited. Although MR suggests a potential protective association for *IRF1*, further functional and context-specific studies are required before therapeutic modulation can be considered. Collectively, targeting PANoptosis-related pathways necessitates rigorous evaluation of safety boundaries, with a focus on balancing therapeutic efficacy against the risk of unintended immune suppression.

This study has several limitations. First, the qRT-PCR validation was based on a small number of lung tissue samples, which may limit statistical power and contribute to the lack of significance for *IRF1* and *UACA*. This is mainly due to the limited availability of well-characterised surgical specimens and the use of strictly defined inclusion criteria. The limited sample size of the experimental validation cohort did not allow comprehensive normality testing before parametric analyses or sensitivity analyses comparing parametric and non-parametric methods. Although we observed no obvious extreme skewness or outliers, the statistical results from the qRT-PCR analyses should still be interpreted cautiously. Therefore, the results should be regarded as preliminary and mainly reflect consistent trends. In addition, the MR analysis for *IRF1* and *UACA* was based on a limited number of IVs. A relatively relaxed LD threshold (*r*^2^<0.3) was applied due to the limited number of *cis*-acting variants, introducing potential residual correlation between instruments. Borderline pleiotropy was observed for UACA (MR-Egger intercept *P* = 0.04), and residual pleiotropy cannot be excluded. Combined with the absence of significant tissue-level expression differences, these findings are suggestive and require validation in independent cohorts and functional studies. The discriminative performance of the identified genes was moderate, and variability across validation data sets (AUC = 0.614–0.743) further suggests that these genes are not sufficient as standalone diagnostic tools but may serve as candidates for risk stratification or biological insight. Although we used external validation data sets, we cannot fully rule out overfitting in biomarker selection.

Second, smoking-related confounding was addressed through MVMR analysis, whereas other clinical variables, including age and sex, could not be fully adjusted due to incomplete annotation across data sets. Notably, we could not stratify by COPD severity, as severity-related information, such as Global Initiative for Chronic Obstructive Lung Disease classification, is not available in the underlying GWAS data. In addition, although the exposure and outcome data sets were derived from largely independent European cohorts, we cannot completely rule out potential sample overlap. Statistical power analysis further supported the reliability of the primary findings, with nearly 100% power observed for the *IRF1*-COPD and *UACA*-COPD models. Therefore, non-significant findings in these analyses should be interpreted cautiously, as false-negative results cannot be excluded. Larger GWAS or meta-analysis data sets will be needed to further evaluate potential weak-effect associations.

Furthermore, the analysis of immune infiltration-based subtypes relies on corresponding clinical phenotype data, including lung function, survival outcomes, and exacerbation history. However, these data were incomplete or unavailable in the GEO data sets. As a result, although higher immune infiltration has been associated with disease progression and exacerbation risk in COPD [32], the clinical significance of the identified subtypes could not be directly evaluated, and comparisons of long-term outcomes across groups were not feasible.

Third, bulk transcriptomic analysis is inherently subject to tissue heterogeneity and cell composition bias. Lung tissue consists of multiple cell types, including epithelial, endothelial, and immune cells, and COPD is characterised by substantial structural remodelling and inflammatory cell infiltration. Consequently, observed expression changes may reflect shifts in cell-type composition rather than true transcriptional regulation within specific cell populations. Methods such as ssGSEA provide indirect estimates of cell-related signals but cannot distinguish changes in cell abundance from changes in cellular activity. In addition, despite rigorous preprocessing and batch correction, differences in sequencing platforms, library preparation, and sample processing across GEO data sets may still introduce variability and affect reproducibility.

Fourth, we did not evaluate candidate gene expression across different lung regions. Bulk RNA-seq analysis inherently carries tissue heterogeneity bias and cell dilution effects. Because lung tissue includes multiple cell types and COPD involves substantial tissue remodelling, including alveolar wall destruction and inflammatory cell infiltration, the observed transcriptional alterations may reflect not only true regulatory differences but also shifts in tissue composition associated with disease progression. *IRF1* is active in alveolar epithelial cells under inflammatory and infectious conditions, where it regulates PANoptosis-related pathways [33]. In contrast, *UACA*, an apoptosome regulator, shows altered expression in tumour and hypoxic contexts [34], but its spatial distribution in COPD lung tissue remains unclear. This limitation is further compounded by the lack of comprehensive multi-tissue transcriptomic data sets in COPD, which constrains systematic evaluation of gene expression patterns. As a result, tissue-specific effects cannot be fully resolved, and transcriptional signals may be obscured or exaggerated. Therefore, future studies should integrate single-cell RNA sequencing or spatial transcriptomics to further validate and characterise these candidate genes.

Lastly, the retrospective design of the transcriptomic data sets carries inherent biases. Future work should validate these findings in larger independent cohorts with well-annotated clinical data to assess the stability and generalisability of PANoptosis-related features, including stratified analyses by smoking status, disease severity, and longitudinal outcomes. Given the limited sample size of the current validation cohort, expanding prospective cohorts will be important to improve statistical power and confirm the robustness of the findings. Functional studies are also required to clarify the underlying mechanisms, for example through gain- and loss-of-function experiments for candidate genes such as *PIK3CG* and *IRF1* in cellular and animal models, combined with tissue-specific or single-cell approaches to better define their roles within the immune microenvironment. In addition, extending validation from the transcript level to protein expression using approaches such as Western blotting or immunohistochemistry would strengthen evidence for candidate genes. At the translational level, peripheral blood–based biomarkers warrant further investigation, particularly for genes enriched in immune cells. Methodological improvements, including nested cross-validation, multi-omics integration, and more rigorous causal inference strategies, may further improve the robustness and clinical relevance of the analytical framework.

## CONCLUSIONS

Our results suggest that *PIK3CG* is a candidate biomarker in COPD, supported by computational analysis and tissue-level validation. Using an MR analysis, we found that *IRF1*- and *UACA*- mediated lung function was genetically associated with COPD risk. By distinguishing candidate biomarkers from genetically implicated genes, we propose a preliminary framework for biomarker and mechanistic research in COPD. Future studies should validate these findings in larger independent cohorts, clarify underlying mechanisms through functional experiments, and explore their potential for minimally invasive clinical applications.

## Additional material


Online Supplementary Document

## Data Availability

**Data availability:** The public data sets used in this study are available from the Gene Expression Omnibus, UK Biobank, FinnGen, and the US Million Veteran Program.
