# Supplementary material for: PANoptosis gene signatures identify candidate biomarkers and genetically associated genes in COPD
Source: J Glob Health. 2026 Oct 30;16:04302. doi: 10.7189/jogh.16.04302 (PMC13721839; doi:10.7189/jogh.16.04302)
Supplement: Supplementary file 1 [file jogh-16-04302-s001.pdf]

Supplement to: Chen H, Wang J, Yang D, Ma T, Wang L, Wan Z, Zhu R. PANoptosis gene signatures identify candidate biomarkers and genetically associated genes in COPD. J Glob Health. 2026;16:04302.

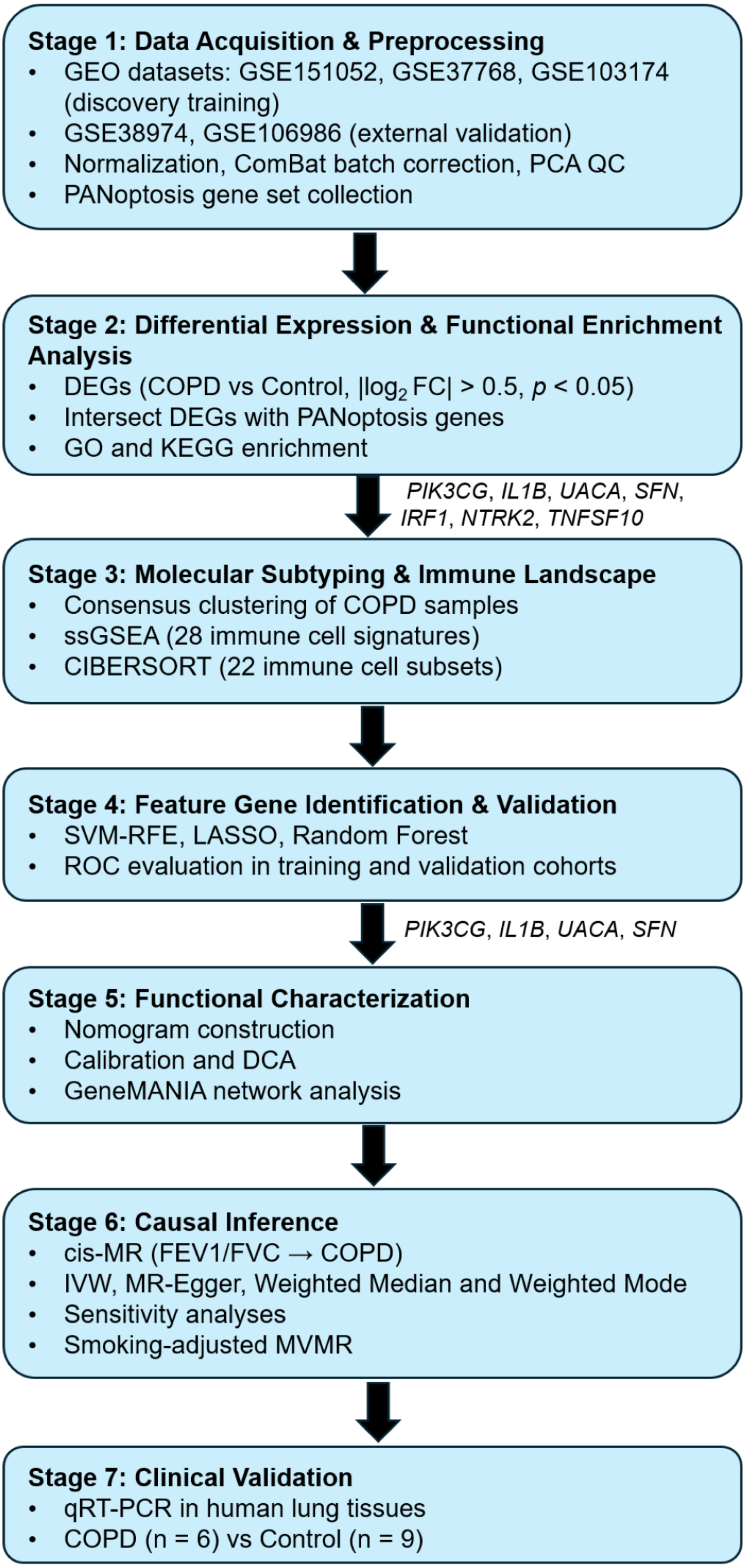
Stage 1: Data Acquisition & Preprocessing
GEO datasets: GSE151052, GSE37768, GSE103174 (discovery training)
GSE38974, GSE106986 (external validation)
Normalization, ComBat batch correction, PCA QC
PANoptosis gene set collection
Stage 2: Differential Expression & Functional Enrichment Analysis
DEGs (COPD vs Control, |log2 FC| > 0.5, p < 0.05)
Intersect DEGs with PANoptosis genes
GO and KEGG enrichment
PIK3CG, IL1B, UACA, SFN, IRF1, NTRK2, TNFSF10
Stage 3: Molecular Subtyping & Immune Landscape
Consensus clustering of COPD samples
ssGSEA (28 immune cell signatures)
CIBERSORT (22 immune cell subsets)
Stage 4: Feature Gene Identification & Validation
SVM-RFE, LASSO, Random Forest
ROC evaluation in training and validation cohorts
PIK3CG, IL1B, UACA, SFN
Stage 5: Functional Characterization
Nomogram construction
Calibration and DCA
GeneMANIA network analysis
Stage 6: Causal Inference
cis-MR (FEV1/FVC → COPD)
IVW, MR-Egger, Weighted Median and Weighted Mode
Sensitivity analyses
Smoking-adjusted MVMR
Stage 7: Clinical Validation
qRT-PCR in human lung tissues
COPD (n = 6) vs Control (n = 9)

Figure S1. Overview of the study workflow. GEO – Gene Expression Omnibus, GSE – Gene Expression Omnibus Series, PCA – principal component analysis, QC – quality control, DEG – differentially expressed gene, COPD – chronic obstructive pulmonary disease, FC – fold change, GO – Gene Ontology, KEGG – Kyoto Encyclopedia of Genes and Genomes, ssGSEA – single-sample gene set enrichment analysis, CIBERSORT – Cell-type Identification By Estimating Relative Subsets Of RNA Transcripts, SVM-RFE – support vector machine-based recursive feature elimination, LASSO – least absolute shrinkage and selection operator, ROC – receiver operating characteristic, DCA – decision curve analysis, MR – Mendelian randomisation, FEV1 – forced expiratory volume in one second, FVC – forced vital capacity, cis-MR – cis-acting Mendelian randomisation, IVW – inverse-variance weighted, MR-Egger – Mendelian randomisation-Egger regression, MVMR – multivariable Mendelian randomisation, qRT-PCR – quantitative real-time polymerase chain reaction.

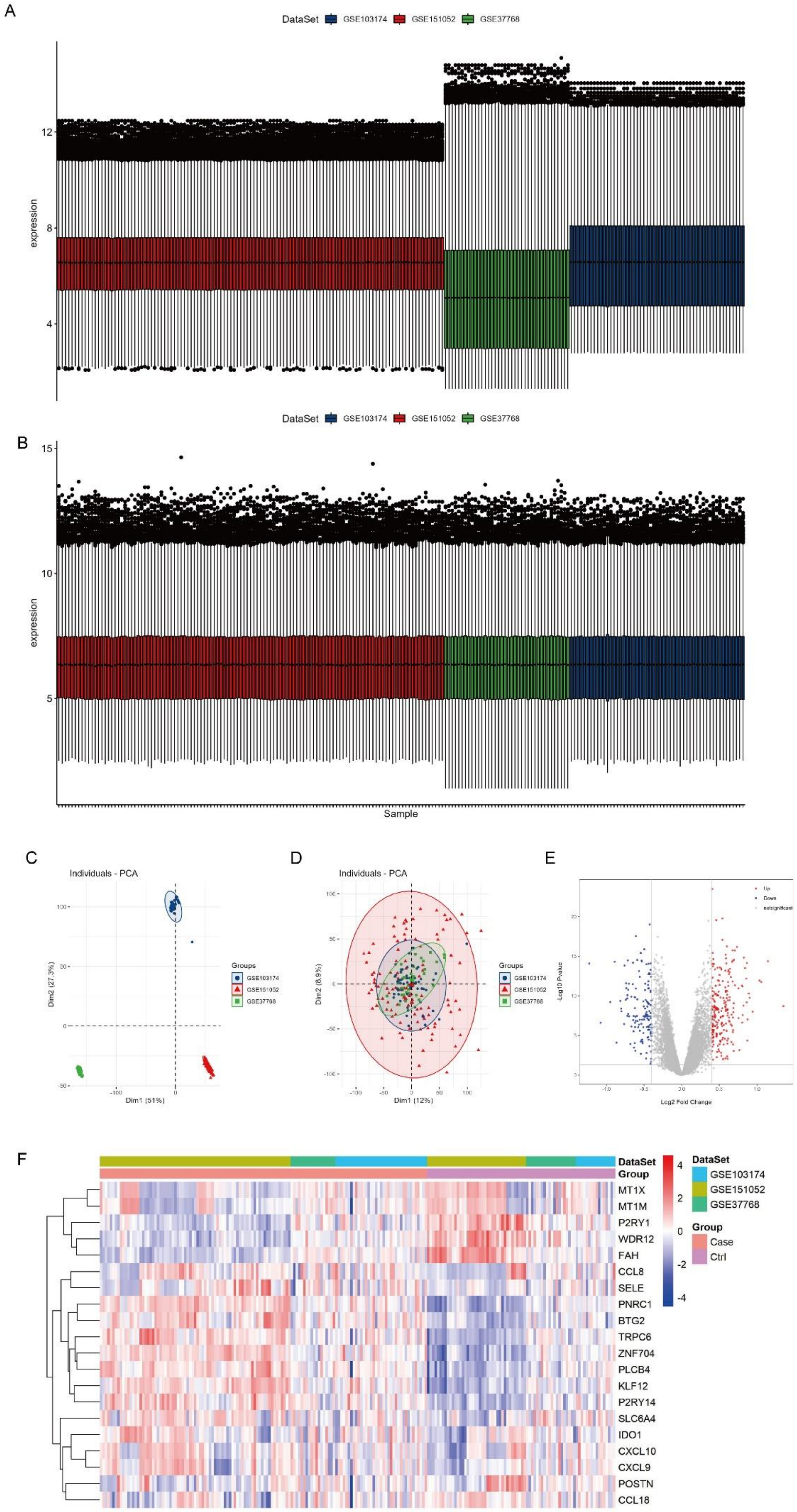

A
DataSet GSE103174 GSE151052 GSE37768
expression
12
8
4
B
DataSet GSE103174 GSE151052 GSE37768
15
10
5
expression
Sample
C
Individuals - PCA
Dim1 (51%)
Dim2 (27.3%)
Groups
GSE103174
GSE37768
D
Individuals - PCA
Dim1 (12%)
Dim2 (8.9%)
Groups
GSE103174
GSE37768
E
-Log10 Pvalue
Log2 Fold Change
F
DataSet
Group
MT1X
MT1M
P2RY1
WDR12
FAH
CCL8
SELE
PNRC1
BTG2
TRPC6
ZNF704
PLCB4
KLF12
P2RY14
SLC6A4
IDO1
CXCL10
CXCL9
POSTN
CCL18
4
2
0
-2
-4
DataSet
GSE103174
GSE151052
GSE37768
Group
Case
Ctrl

Figure S2. Batch-effect correction and differential expression analysis in COPD cohorts. Panel A. Expression distributions across GSE151052, GSE37768, and GSE103174 before batch-effect correction. **Panel B.** Expression distributions across the three data sets after batch-effect correction. **Panel C.** PCA before batch-effect correction. **Panel D.** PCA after batch-effect correction. **Panel E.** Volcano plot showing 361 DEGs, including 224 upregulated genes (red) and 137 downregulated genes (blue) in COPD versus control samples; grey indicates non-significant genes. **Panel F.** Heatmap showing the expression patterns of the top 20 DEGs across the integrated samples, with blue indicating lower expression and red indicating higher expression. COPD – chronic obstructive pulmonary disease, Ctrl – control, DEG – differentially expressed gene, Dim – dimension, GSE – Gene Expression Omnibus Series, PCA – principal component analysis.

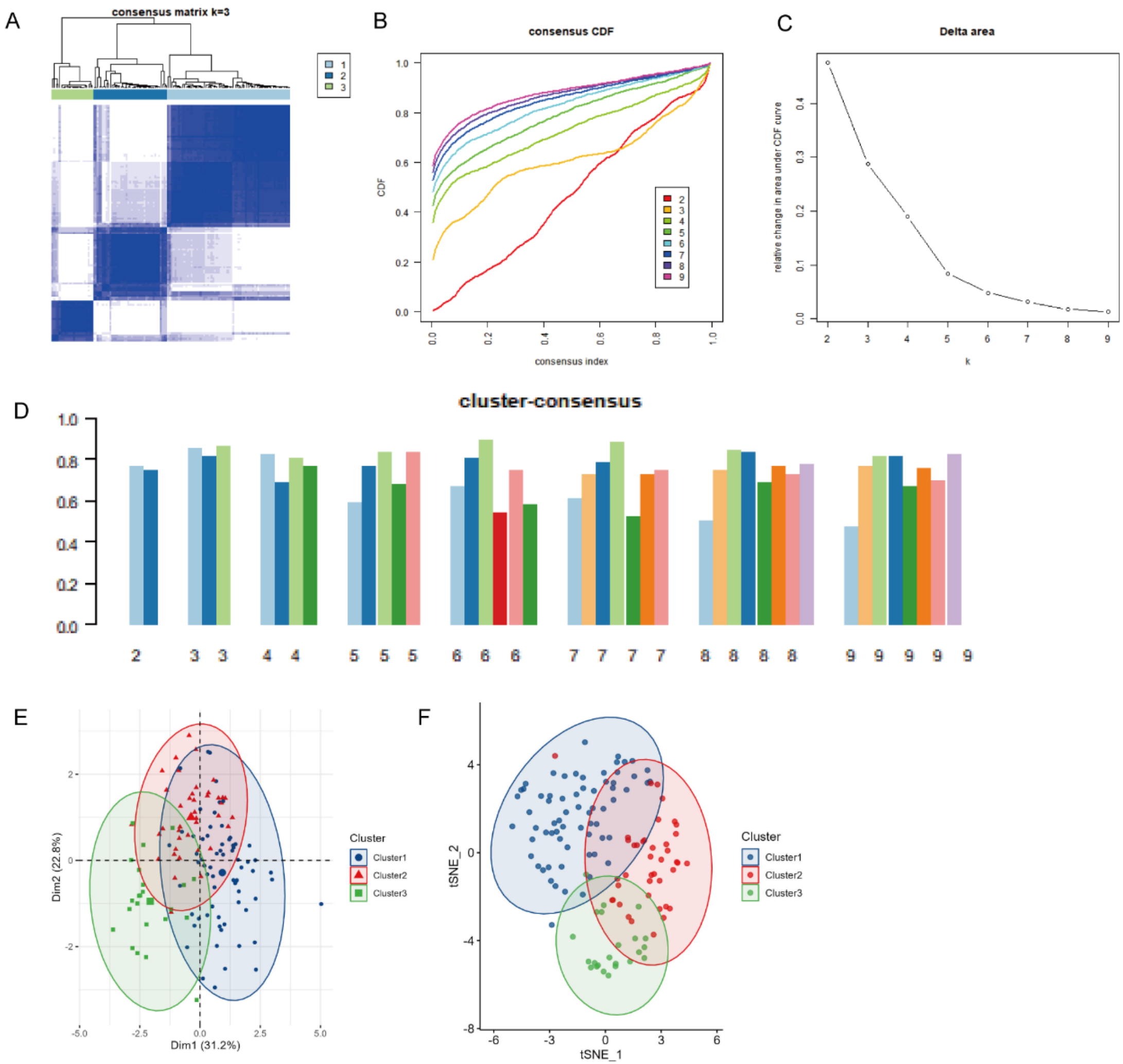

Figure S3. Consensus clustering and dimensionality reduction of COPD molecular subtypes. Panel A. Consensus matrix heatmap at *k* = 3. **Panel B.** Consensus cumulative distribution function curves for *k* = 2–9. **Panel C**. Relative change in area under the cumulative distribution function curve across different *k* values. **Panel D**. Cluster-consensus scores across different *k* assignments. **Panel E.** PCA of the three COPD clusters. **Panel F.** t-SNE visualisation of the three COPD clusters. Group sizes were Cluster 1 (n = 68, blue), Cluster 2 (n = 41, red), and Cluster 3 (n = 23, green), with 95% confidence ellipses. CDF – cumulative distribution function, COPD – chronic obstructive pulmonary disease, Dim – dimension, PCA – principal component analysis, t-SNE – t-distributed stochastic neighbour embedding.

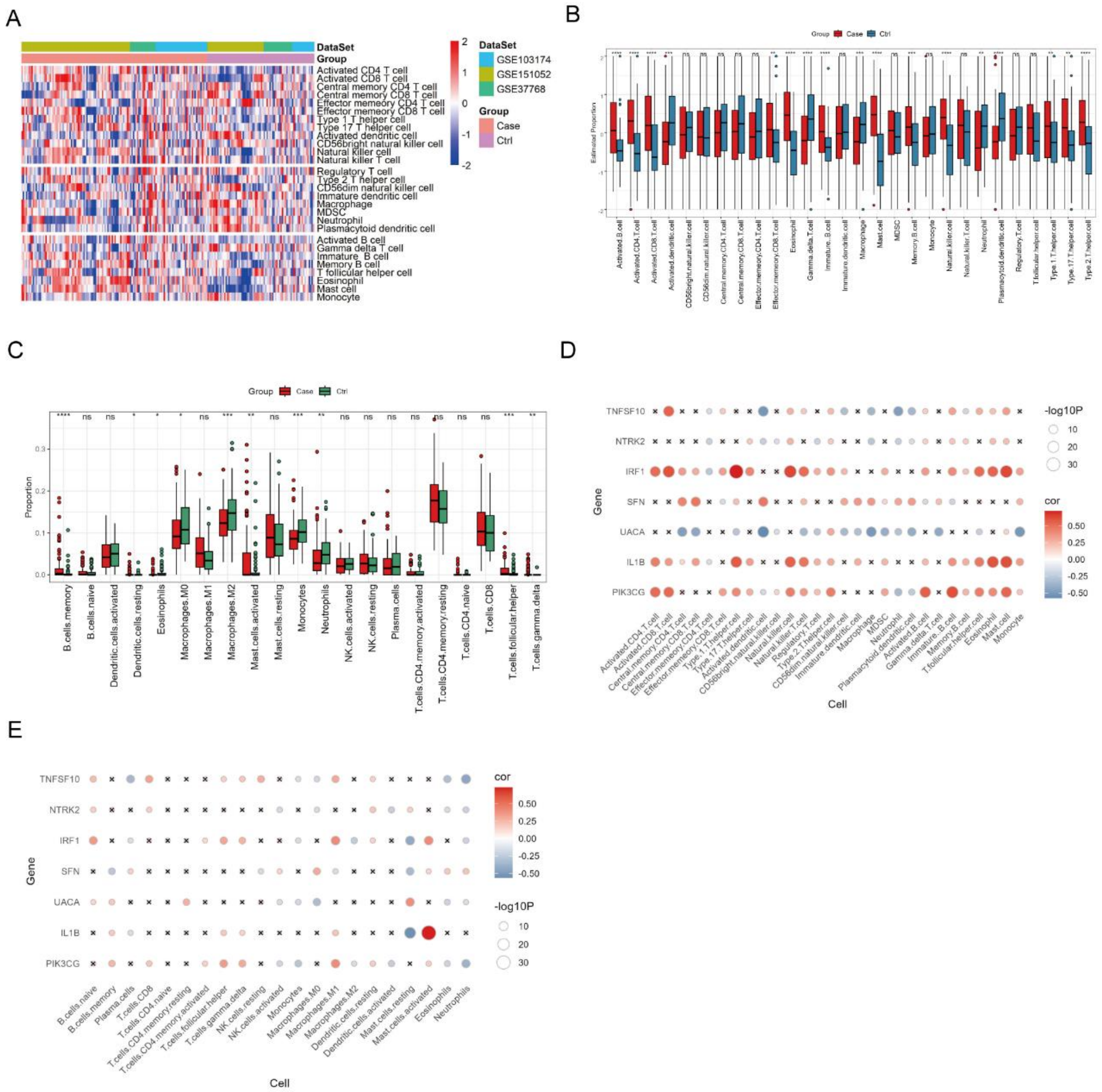

Figure S4. Immune infiltration and correlations with PANoptosis-related genes in COPD. Panel A. Heatmap showing immune cell enrichment across the integrated samples, with red indicating higher and blue indicating lower enrichment scores. **Panel B.** ssGSEA-based comparison of 28 immune cell types between COPD cases (red) and controls (blue). **Panel C.** CIBERSORT-based comparison of 22 immune cell subsets between COPD cases (red) and controls (green). **Panel D.** Spearman correlations between the seven PANoptosis-related genes and ssGSEA-derived immune cell signatures. **Panel E.** Spearman correlations between the seven PANoptosis-related genes and CIBERSORT-derived immune cell proportions. In Panels D and E, red indicates positive correlations and blue indicates negative correlations; bubble size represents statistical significance, and × indicates non-significant correlations. Statistical significance in Panels B and C was assessed using the Wilcoxon rank-sum test: **P* < 0.05; ***P* < 0.01; ****P* < 0.001; *****P* < 0.0001; ns, non-significant. CIBERSORT – Cell-type Identification By Estimating Relative Subsets Of RNA Transcripts, COPD – chronic obstructive pulmonary disease, cor – correlation coefficient, Ctrl

– control, MDSC – myeloid-derived suppressor cell, NK – natural killer, $-\log_{10} P$ – negative base-10 logarithm of the P-value, ssGSEA – single-sample gene set enrichment analysis, CD – cluster of differentiation, M0 – unpolarised macrophages, M1 – classically activated macrophages, M2 – alternatively activated macrophages.

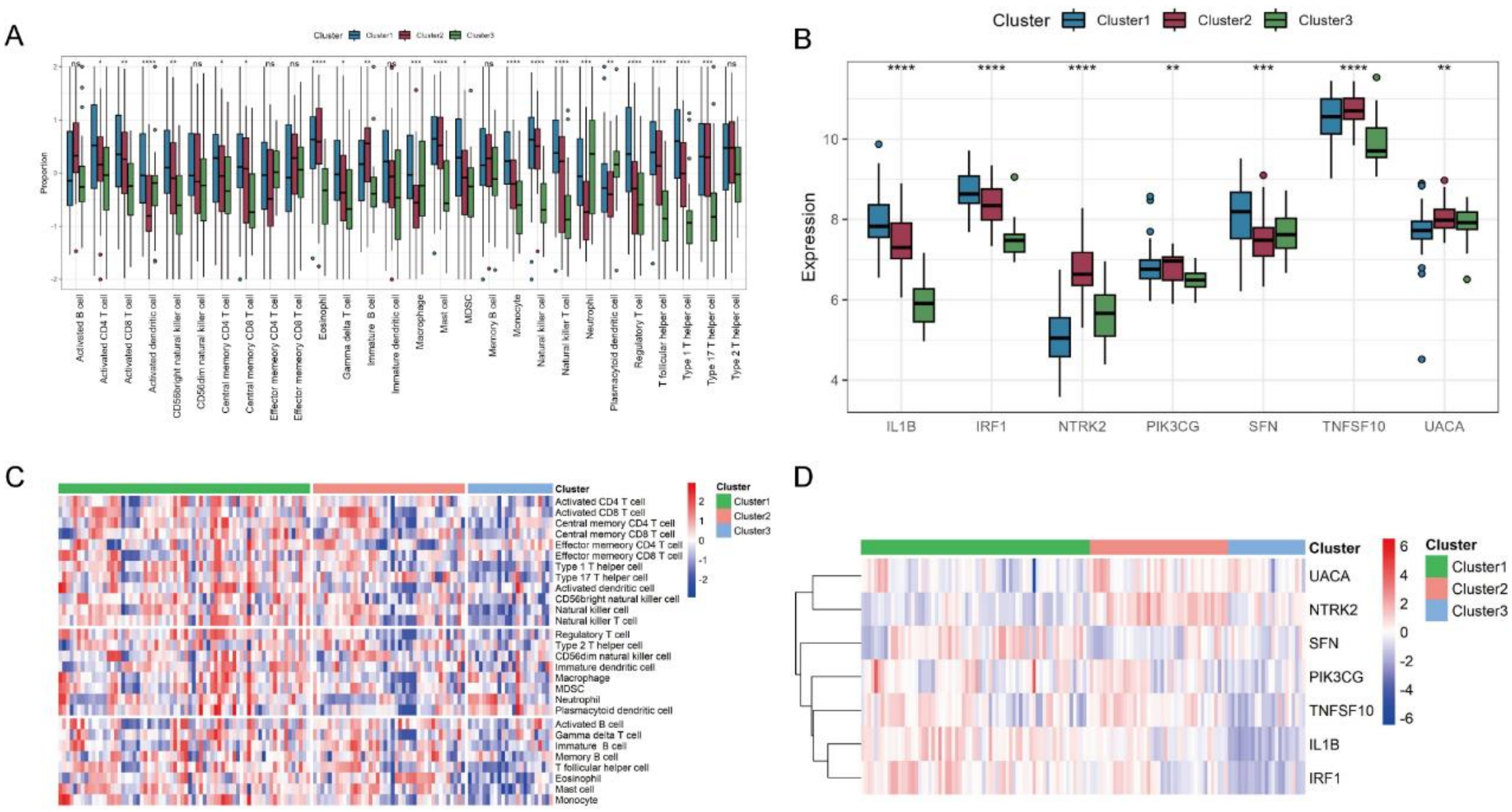

Figure S5. Immune infiltration and PANoptosis-related gene expression across COPD molecular subtypes. Panel A. ssGSEA-based comparison of 28 immune cell types across the three COPD clusters. **Panel B.** Expression of seven PANoptosis-related DEGs across the three COPD clusters. **Panel C.** Heatmap of ssGSEA-derived immune cell enrichment across the three COPD clusters, with red indicating higher and blue indicating lower enrichment scores. **Panel D.** Heatmap of the seven PANoptosis-related DEGs across the three COPD clusters, with red indicating higher and blue indicating lower expression levels. Inter-group comparisons among the three clusters were assessed using one-way ANOVA: *$P < 0.05$; **$P < 0.01$; ***$P < 0.001$; ****$P < 0.0001$; ns, non-significant. ANOVA – analysis of variance, COPD – chronic obstructive pulmonary disease, DEG – differentially expressed gene, ssGSEA – single-sample gene set enrichment analysis, CD – cluster of differentiation, MDSC – myeloid-derived suppressor cell.

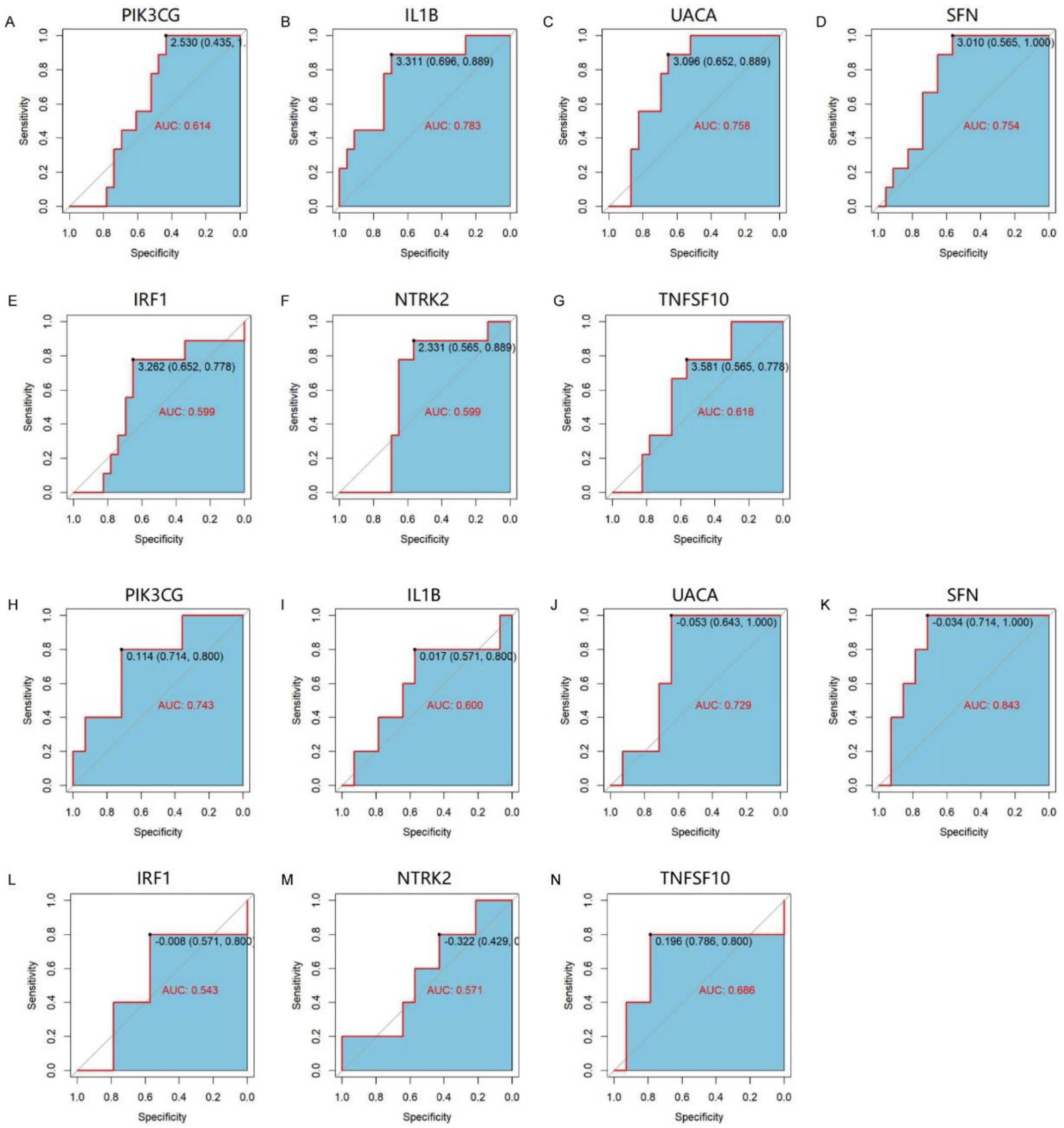

Figure S6. External validation of PANoptosis-related candidate biomarkers. Panels A–G. ROC curves for *PIK3CG*, *IL1B*, *UACA*, *SFN*, *IRF1*, *NTRK2*, and *TNFSF10* in GSE38974. **Panels H–N.** ROC curves for the same seven genes in GSE106986. Specifically, *PIK3CG* is shown in Panels A and H, *IL1B* in Panels B and I, *UACA* in Panels C and J, *SFN* in Panels D and K, *IRF1* in Panels E and L, *NTRK2* in Panels F and M, and *TNFSF10* in Panels G and N. AUC – area under the curve, GSE – Gene Expression Omnibus Series, ROC – receiver operating characteristic.

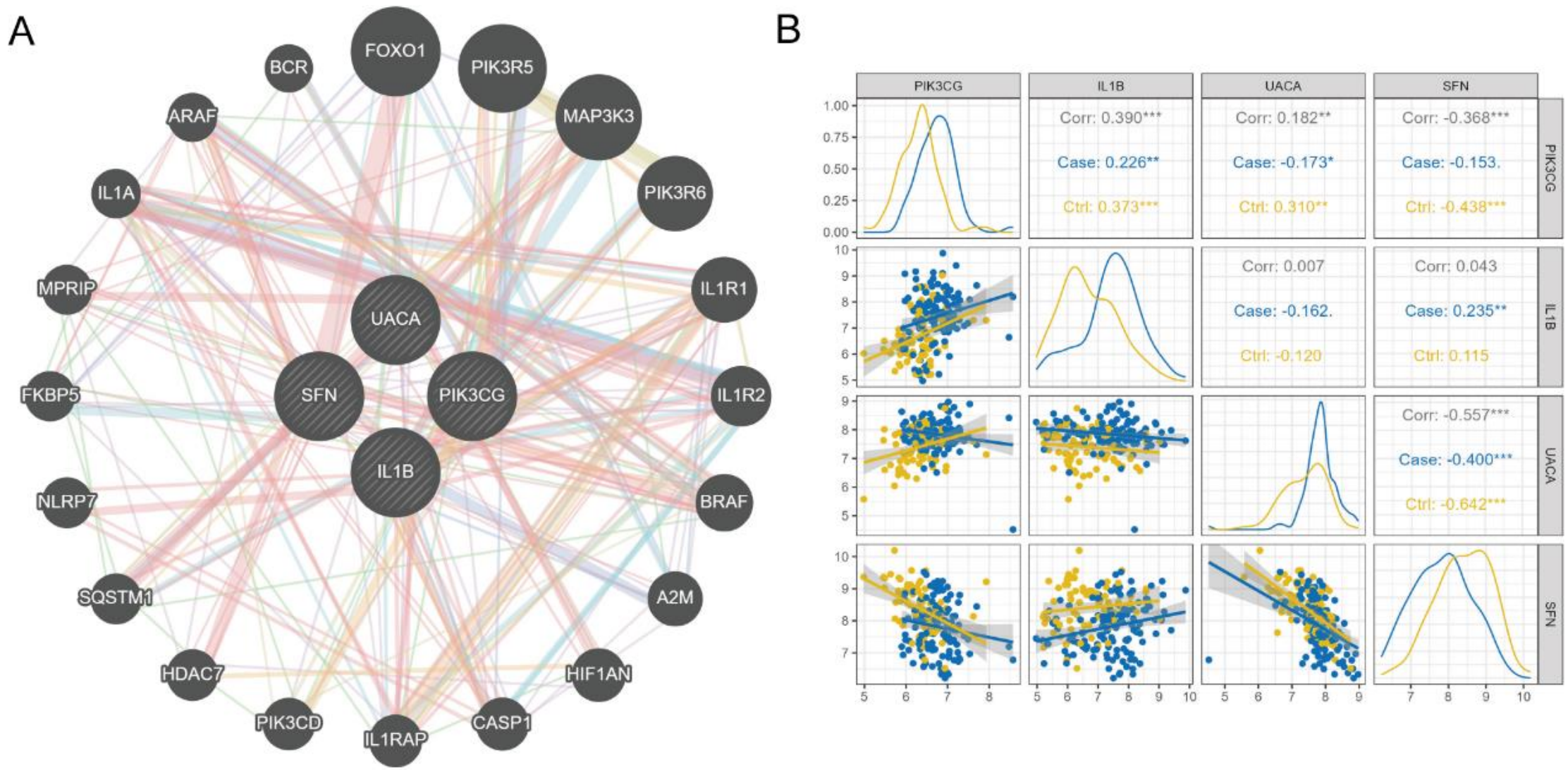

Figure S7. Protein-protein interaction network and expression correlations of four core feature genes. Panel A. GeneMANIA-based protein-protein interaction network of *SFN*, *UACA*, *IL1B*, and *PIK3CG* and their related genes. Nodes represent individual genes. **Panel B.** Pairwise expression correlations among *PIK3CG*, *IL1B*, *UACA*, and *SFN* in COPD cases and controls. Diagonal panels show group-specific expression density distributions, lower-triangle panels show scatter plots with fitted regression lines, and upper-triangle panels show overall and group-specific correlation coefficients. Blue represents COPD cases and yellow represents controls. Statistical significance is denoted as: **P* < 0.05; ***P* < 0.01; ****P* < 0.001; *****P* < 0.0001; ns, non-significant. COPD – chronic obstructive pulmonary disease, Corr – correlation coefficient, Ctrl – control, PPI – protein-protein interaction.

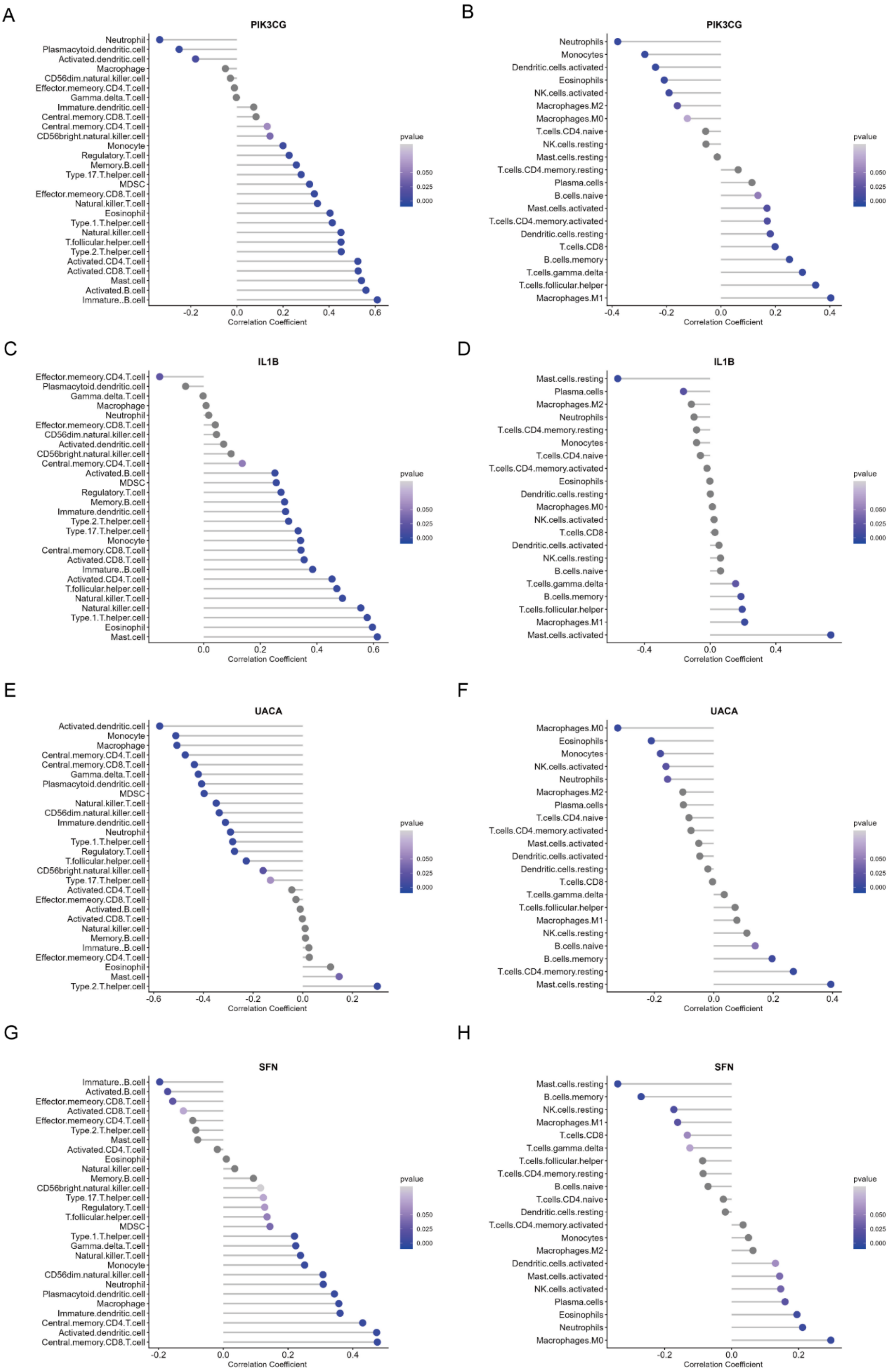

Figure S8. Correlations between feature genes and immune cell subsets based on ssGSEA and CIBERSORT analyses. Panels A and B. Correlations between *PIK3CG* and immune cell subsets estimated by

ssGSEA (Panel A) and CIBERSORT (Panel B). **Panels C and D**. Correlations between *IL1B* and immune cell subsets estimated by ssGSEA (Panel C) and CIBERSORT (Panel D). **Panels E and F**. Correlations between *UACA* and immune cell subsets estimated by ssGSEA (Panel E) and CIBERSORT (Panel F). **Panels G and H**. Correlations between *SFN* and immune cell subsets estimated by ssGSEA (Panel G) and CIBERSORT (Panel H). The horizontal axis shows the Spearman correlation coefficient, and node colour represents statistical significance according to the *P*-value gradient. CIBERSORT – Cell-type Identification By Estimating Relative Subsets Of RNA Transcripts, CD – cluster of differentiation, M0 – unpolarised macrophages, M1 – classically activated macrophages, M2 – alternatively activated macrophages, MDSC – myeloid-derived suppressor cell, NK – natural killer, ssGSEA – single-sample gene set enrichment analysis.

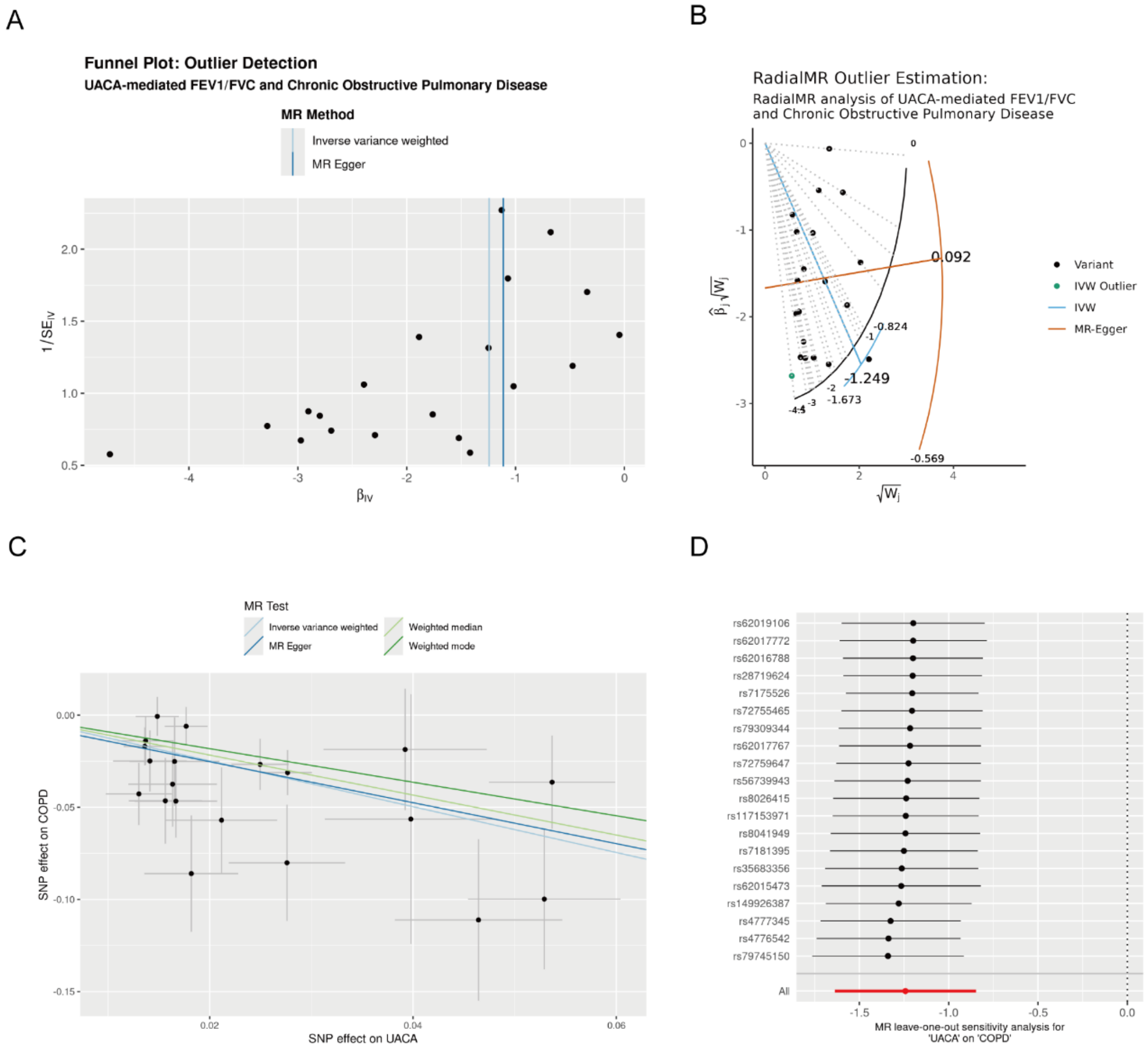

Figure S9. Sensitivity analyses for *UACA*-mediated MR. Panel A. Funnel plot for *UACA*-mediated FEV1/FVC and COPD risk. **Panel B.** Radial MR plot for *UACA*-mediated FEV1/FVC and COPD risk. **Panel C.** Scatter plot of SNP effects on *UACA*-mediated FEV1/FVC and COPD risk across MR models. **Panel D.** Leave-one-out sensitivity analysis for *UACA*-mediated FEV1/FVC and COPD risk. COPD – chronic obstructive pulmonary disease, FEV1/FVC – forced expiratory volume in one second/forced vital capacity, IV – instrumental variable, IVW – inverse-variance weighted, MR – Mendelian randomisation, MR-Egger – Mendelian randomisation-Egger regression, SE – standard error, SNP – single nucleotide polymorphism.

Table S1. STROBE Statement—Checklist of items that should be included in reports of *case-control studies*

	Item No	Recommendation	Page No
**Title and abstract**	1	(*a*) Indicate the study's design with a commonly used term in the title or the abstract	1
		(*b*) Provide in the abstract an informative and balanced summary of what was done and what was found	1
**Introduction**			
Background/rationale	2	Explain the scientific background and rationale for the investigation being reported	1–2
Objectives	3	State specific objectives, including any prespecified hypotheses	2
**Methods**			
Study design	4	Present key elements of study design early in the paper	2–3; Supplementary Methods
Setting	5	Describe the setting, locations, and relevant dates, including periods of recruitment, exposure, follow-up, and data collection	2–3; Supplementary Methods
Participants	6	(*a*) Give the eligibility criteria, and the sources and methods of case ascertainment and control selection. Give the rationale for the choice of cases and controls	2–3; Supplementary Methods
		(*b*) For matched studies, give matching criteria and the number of controls per case	-

Variables	7	Clearly define all outcomes, exposures, predictors, potential confounders, and effect modifiers. Give diagnostic criteria, if applicable	2-3; Supplementary Methods
Data sources/ measurement	8*	For each variable of interest, give sources of data and details of methods of assessment (measurement). Describe comparability of assessment methods if there is more than one group	2-3; Supplementary Methods
Bias	9	Describe any efforts to address potential sources of bias	2-3; Supplementary Methods
Study size	10	Explain how the study size was arrived at	2-3; Supplementary Methods
Quantitative variables	11	Explain how quantitative variables were handled in the analyses. If applicable, describe which groupings were chosen and why	-
Statistical methods	12	(*a*) Describe all statistical methods, including those used to control for confounding	2-3; Supplementary Methods
		(*b*) Describe any methods used to examine subgroups and interactions	2-3; Supplementary Methods
		(*c*) Explain how missing data were addressed	Supplementary Methods
		(*d*) If applicable, explain how matching of cases and controls was addressed	-
		(*e*) Describe any sensitivity analyses	-
**Results**			

Participants	13*	(a) Report numbers of individuals at each stage of study—eg numbers potentially eligible, examined for eligibility, confirmed eligible, included in the study, completing follow-up, and analysed	2-3; Supplementary Methods
		(b) Give reasons for non-participation at each stage	Supplementary Methods
		(c) Consider use of a flow diagram	2; Supplementary Methods
Descriptive data	14*	(a) Give characteristics of study participants (eg demographic, clinical, social) and information on exposures and potential confounders	2-3; Supplementary Methods
		(b) Indicate number of participants with missing data for each variable of interest	-
Outcome data	15*	Report numbers in each exposure category, or summary measures of exposure	3-5; 10-11

Main results	16	(*a*) Give unadjusted estimates and, if applicable, confounder-adjusted estimates and their precision (eg, 95% confidence interval). Make clear which confounders were adjusted for and why they were included	3-5; 10-11
		(*b*) Report category boundaries when continuous variables were categorized	-
		© If relevant, consider translating estimates of relative risk into absolute risk for a meaningful time period	-
-Other analyses	17	Report other analyses done—eg analyses of subgroups and interactions, and sensitivity analyses	5-6
**Discussion**			
Key results	18	Summarise key results with reference to study objectives	11
Limitations	19	Discuss limitations of the study, taking into account sources of potential bias or imprecision. Discuss both direction and magnitude of any potential bias	13-14
Interpretation	20	Give a cautious overall interpretation of results considering objectives, limitations, multiplicity of analyses, results from similar studies, and other relevant evidence	11-13
Generalisability	21	Discuss the generalisability (external validity) of the study results	11-14
**Other information**			
Funding	22	Give the source of funding and the role of the funders for the present study and, if applicable, for the original study on which the present article is based	14

*Give information separately for cases and controls.

Note: An Explanation and Elaboration article discusses each checklist item and gives methodological background and published examples of transparent reporting. The STROBE checklist is best used in conjunction with this article (freely available on the Web sites of PLoS Medicine at http://www.plosmedicine.org/, Annals of Internal Medicine at http://www.annals.org/, and Epidemiology at http://www.epidem.com/). Information on the STROBE Initiative is available at http://www.strobe-statement.org.

Table S2. STROBE-MR checklist of recommended items to address in reports of Mendelian randomization studies[1 2]

Item No.	Section	Checklist item	Page No.	Relevant text from manuscript
1	**TITLE and ABSTRACT**	Indicate Mendelian randomization (MR) as the study's design in the title and/or the abstract if that is a main purpose of the study	1	PANoptosis gene signatures identify candidate biomarkers and genetically associated genes in COPD<br>Background PANoptosis contributes to chronic obstructive pulmonary disease (COPD) pathogenesis …
	**INTRODUCTION**			
2	**Background**	Explain the scientific background and rationale for the reported study. What is the exposure? Is a potential causal relationship between exposure and outcome plausible? Justify why MR is a helpful method to address the study question	1-2	Chronic obstructive pulmonary disease (COPD) is a major global health burden characterised by persistent respiratory symptoms and progressive airflow limitation [1] …<br>PANoptosis, a coordinated lytic cell death pathway that integrates pyroptosis, apoptosis, and necroptosis through multiprotein complexes called PANoptosomes …

				Despite increasing evidence implicating PANoptosis in COPD, reliable PANoptosis-related biomarkers remain limited …
3	**Objectives**	State specific objectives clearly, including pre-specified causal hypotheses (if any). State that MR is a method that, under specific assumptions, intends to estimate causal effects	2	In this study, we focused on the potential relevance of PANoptosis-related genes to COPD biomarker discovery and disease-related biological interpretation …
	**METHODS**			
4	**Study design and data sources**	Present key elements of the study design early in the article. Consider including a table listing sources of data for all phases of the study. For each data source contributing to the analysis, describe the following:		
	a)	Setting: Describe the study design and the underlying population, if possible. Describe the setting, locations, and relevant dates, including periods of recruitment, exposure, follow-up, and data collection, when available.	2-3; Supplementary Methods	METHODS<br>We employed a multi-stage integrative analysis pipeline to identify PANoptosis-related gene signatures in COPD …<br>Supplementary Methods<br>1. Study design

	b)	Participants: Give the eligibility criteria, and the sources and methods of selection of participants. Report the sample size, and whether any power or sample size calculations were carried out prior to the main analysis	3; Supplementary Methods	METHODS<br>To evaluate genetic associations, we performed univariable two-sample MR using the FEV1/FVC ratio as a genetic proxy for airflow limitation and COPD as the outcome …<br>Supplementary Methods<br>10. MR analysis<br>10.1 Data sources for MR analysis
	c)	Describe measurement, quality control and selection of genetic variants	3; Supplementary Methods	METHODS<br>Cis-acting instrumental variables near target genes were selected based on association strength, minor allele frequency, F-statistic, and linkage disequilibrium criteria …<br>Supplementary Methods<br>10. MR analysis<br>10.2 Selection of instrumental variables (IVs)

		d) For each exposure, outcome, and other relevant variables, describe methods of assessment and diagnostic criteria for diseases	-	
		e) Provide details of ethics committee approval and participant informed consent, if relevant	3	METHODS<br>Public GEO datasets are de-identified and publicly available and therefore did not require additional ethical approval.<br>All genome-wide association study (GWAS) datasets for FEV1/FVC and COPD variants were restricted to European ancestry populations and did not require additional ethical approval.
5	**Assumptions**	Explicitly state the three core IV assumptions for the main analysis (relevance, independence and exclusion restriction) as well assumptions for any additional or sensitivity analysis	Supplementary Methods	Supplementary Methods<br>10. MR analysis<br>10.2 Selection of instrumental variables (IVs)
6	**Statistical methods: main analysis**	Describe statistical methods and statistics used		

	a)	Describe how quantitative variables were handled in the analyses (i.e., scale, units, model)	-	
	b)	Describe how genetic variants were handled in the analyses and, if applicable, how their weights were selected	3; Supplementary Methods	METHODS<br>Cis-acting instrumental variables near target genes were selected based on association strength, minor allele frequency, F-statistic, and linkage disequilibrium criteria …<br>Supplementary Methods<br>10. MR analysis<br>10.2 Selection of instrumental variables (IVs)
	c)	Describe the MR estimator (e.g. two-stage least squares, Wald ratio) and related statistics. Detail the included covariates and, in case of two-sample MR, whether the same covariate set was used for adjustment in the two samples	3; Supplementary Methods	METHODS<br>Inverse-variance weighted analysis was used as the primary method, with complementary sensitivity analyses for heterogeneity, pleiotropy, and influential variants …<br>Supplementary Methods<br>10. MR analysis<br>10.3 MR analysis

		d) Explain how missing data were addressed	-	
		e) If applicable, indicate how multiple testing was addressed	Supplementary Methods	Supplementary Methods<br>10. MR analysis<br>10.3 MR analysis
7	**Assessment of assumptions**	Describe any methods or prior knowledge used to assess the assumptions or justify their validity	-	
8	**Sensitivity analyses and additional analyses**	Describe any sensitivity analyses or additional analyses performed (e.g. comparison of effect estimates from different approaches, independent replication, bias analytic techniques, validation of instruments, simulations)	3; Supplementary Methods	METHODS<br>We further conducted MVMR analyses by incorporating lifetime smoking and current smoking phenotypes from the US Million Veteran Program (MVP), as covariates to adjust for potential confounding …<br>Supplementary Methods<br>10. MR analysis<br>10.4 Sensitivity analysis and hierarchical outlier removal<br>10.5 Quantitative assessment of statistical power

				11. Multivariable Mendelian randomization (MVMR) analysis<br>11.1 Data sources for MVMR analysis<br>11.2 Selection of IVs<br>11.3 MVMR analysis and sensitivity analysis
9	**Software and pre-registration**			
		a) Name statistical software and package(s), including version and settings used	Supplementary Methods	Supplementary Methods<br>10. MR analysis<br>10.6 Statistical analysis<br>Supplementary Methods<br>11. Multivariable Mendelian randomization (MVMR) analysis<br>11.4 Statistical analysis
		b) State whether the study protocol and details were pre-registered (as well as when and where)	-	

RESULTS

10	**Descriptive data**			
	a)	Report the numbers of individuals at each stage of included studies and reasons for exclusion. Consider use of a flow diagram	8	MR analysis of PANoptosis-related genes for COPD risk<br>Among the seven candidate genes, four met our baseline inclusion criterion of having more than two valid instrumental variables IVs: IRF1 (17 IVs), PIK3CG (3 IVs), TNFSF10 (3 IVs), and UACA (20 IVs) …
	b)	Report summary statistics for phenotypic exposure(s), outcome(s), and other relevant variables (e.g. means, SDs, proportions)	8	MR analysis of PANoptosis-related genes for COPD risk<br>The primary inverse-variance weighted meta-analysis revealed that genetically instrumented lung function (FEV1/FVC) mediated by specific feature genes exerted distinct causal effects on COPD susceptibility …
	c)	If the data sources include meta-analyses of previous studies, provide the assessments of heterogeneity across these studies	-	

		d) For two-sample MR:<br>i. Provide justification of the similarity of the genetic variant-exposure associations between the exposure and outcome samples<br>ii. Provide information on the number of individuals who overlap between the exposure and outcome studies	-	
11	**Main results**			
		a) Report the associations between genetic variant and exposure, and between genetic variant and outcome, preferably on an interpretable scale	8	MR analysis of PANoptosis-related genes for COPD risk<br>Among the seven candidate genes, four met our baseline inclusion criterion of having more than two valid instrumental variables IVs: IRF1 (17 IVs), PIK3CG (3 IVs), TNFSF10 (3 IVs), and UACA (20 IVs) …
		b) Report MR estimates of the relationship between exposure and outcome, and the measures of uncertainty from the MR analysis, on an interpretable scale, such as odds ratio or relative risk per SD difference	8	MR analysis of PANoptosis-related genes for COPD risk<br>The primary inverse-variance weighted meta-analysis revealed that genetically instrumented lung function (FEV1/FVC) mediated by specific feature genes exerted

					distinct causal effects on COPD susceptibility …
		c)	If relevant, consider translating estimates of relative risk into absolute risk for a meaningful time period	-	
		d)	Consider plots to visualize results (e.g. forest plot, scatterplot of associations between genetic variants and outcome versus between genetic variants and exposure)	9	Figure 3
12	**Assessment of assumptions**				
		a)	Report the assessment of the validity of the assumptions	8-9	MR analysis of PANoptosis-related genes for COPD risk<br>Standard sensitivity metrics further substantiated the validity of our primary causal estimates ...
		b)	Report any additional statistics (e.g., assessments of heterogeneity across genetic variants, such as I^2, Q statistic or E-value)	8-9	MR analysis of PANoptosis-related genes for COPD risk<br>Standard sensitivity metrics further substantiated the validity of our primary causal estimates ...<br>To visually validate these core MR assumptions, we conducted a

					comprehensive sensitivity diagnostic pipeline …
13	**Sensitivity analyses and additional analyses**				
		a)	Report any sensitivity analyses to assess the robustness of the main results to violations of the assumptions	8-9	MR analysis of PANoptosis-related genes for COPD risk<br>Standard sensitivity metrics further substantiated the validity of our primary causal estimates ...<br>To visually validate these core MR assumptions, we conducted a comprehensive sensitivity diagnostic pipeline …<br>Post hoc statistical power analyses further supported the reliability of the findings. All utilised instrumental variables showed sufficient strength …
		b)	Report results from other sensitivity analyses or additional analyses	10	MVMR analysis adjusting for smoking-related genetic effects

				To assess whether the observed associations were independent of smoking-related genetic confounding, we performed MVMR analyses using genetically predicted lifetime smoking and current smoking phenotypes as covariates …<br>After adjusting for current smoking …
		c) Report any assessment of direction of causal relationship (e.g., bidirectional MR)	-	
		d) When relevant, report and compare with estimates from non-MR analyses	-	
		e) Consider additional plots to visualize results (e.g., leave-one-out analyses)	9	Figure 3; Figure S9
	**DISCUSSION**			
14	**Key results**	Summarize key results with reference to study objectives	11	DISCUSSION<br>Distinguishing reliable diagnostic biomarkers from genetically implicated genes remains a challenge in COPD research …

15	**Limitations**	Discuss limitations of the study, taking into account the validity of the IV assumptions, other sources of potential bias, and imprecision. Discuss both direction and magnitude of any potential bias and any efforts to address them	13-14	DISCUSSION<br>This study has several limitations …
16	**Interpretation**			
		a) Meaning: Give a cautious overall interpretation of results in the context of their limitations and in comparison with other studies	12	DISCUSSION<br>In the MR analysis, genetic increases in IRF1- and UACA-mediated FEV1/FVC were associated with lower COPD risk, suggesting a potential association with lung function preservation …<br>An important strength of this study is the incorporation of MVMR to distinguish the independent biological effects of lung function and smoking behaviour …
		b) Mechanism: Discuss underlying biological mechanisms that could drive a potential causal relationship between the investigated exposure and the outcome, and whether the gene-environment equivalence assumption is reasonable.	11-13	DISCUSSION<br>In this study, PIK3CG was up-regulated in COPD lung tissue and showed moderate, variable discriminative performance across

	Use causal language carefully, clarifying that IV estimates may provide causal effects only under certain assumptions		cohorts, underscoring its value as a biomarker. PIK3CG encodes the catalytic subunit of phosphoinositide 3-kinase gamma (PI3Kγ), a leukocyte-restricted kinase regulating immune cell trafficking and activation [22] …<br><br>In the MR analysis, genetic increases in IRF1- and UACA-mediated FEV1/FVC were associated with lower COPD risk, suggesting a potential association with lung function preservation …<br><br>From a translational perspective, PI3Kγ may represent a pharmacologically tractable pathway for further investigation. Selective PI3Kγ inhibitors such as eganelisib (IPI-549) have demonstrated anti-inflammatory activity in preclinical models [30] …
c)	Clinical relevance: Discuss whether the results have clinical or public policy relevance, and to what extent they inform effect sizes of possible interventions	12-13	DISCUSSION

				However, therapeutic intervention in this pathway requires careful caliBration …
17	**Generalizability**	Discuss the generalizability of the study results (a) to other populations, (b) across other exposure periods/timings, and (c) across other levels of exposure	13-14	DISCUSSION<br>This study has several limitations. First, the qRT-PCR validation was based on a small number of lung tissue samples, which may limit statistical power and contribute to the lack of significance for IRF1 and UACA. This is mainly due to the limited availability of well-characterised surgical specimens and the use of strictly defined inclusion criteria …
	**OTHER INFORMATION**			
18	**Funding**	Describe sources of funding and the role of funders in the present study and, if applicable, sources of funding for the databases and original study or studies on which the present study is based	14	Funding: The authors received no specific funding for this work.

19	**Data and data sharing**	Provide the data used to perform all analyses or report where and how the data can be accessed, and reference these sources in the article. Provide the statistical code needed to reproduce the results in the article, or report whether the code is publicly accessible and if so, where	14	Data availability: The public data sets used in this study are available from the Gene Expression Omnibus, UK Biobank, FinnGen, and the US Million Veteran Program.
20	**Conflicts of Interest**	All authors should declare all potential conflicts of interest	14	Disclosure of interest: The authors completed the ICMJE Disclosure of Interest Form (available upon request from the corresponding author) and disclose no relevant interests.

This checklist is copyrighted by the Equator Network under the Creative Commons Attribution 3.0 Unported (CC BY 3.0) license.

1. Skrivankova VW, Richmond RC, Woolf BAR, Yarmolinsky J, Davies NM, Swanson SA, et al. Strengthening the Reporting of Observational Studies in Epidemiology using Mendelian Randomization (STROBE-MR) Statement. JAMA. 2021;under review.

2. Skrivankova VW, Richmond RC, Woolf BAR, Davies NM, Swanson SA, VanderWeele TJ, et al. Strengthening the Reporting of Observational Studies in Epidemiology using Mendelian Randomisation (STROBE-MR): Explanation and Elaboration. BMJ. 2021;375:n2233.

Table S3. Data sources and experimental information used in this study.
Part A. Baseline characteristics of the included GEO cohorts.

Dataset	COPD, n	Control, n	Tissue	Smoking	Severity	Age, years	Sex
GSE151052	77	40	Lung (transplant vs donor)	NA	GOLD 4	39–77	NA
GSE37768	18	20	Lung	Controls: 9 non-smokers, 11 smokers	Moderate	NA	NA
GSE103174	37	16	Lung	24 non-smokers, 29 smokers	NA	48–80	22 females, 31 males

COPD – chronic obstructive pulmonary disease, GEO – Gene Expression Omnibus, GSE – Gene Expression Omnibus Series, GOLD – Global Initiative for Chronic Obstructive Lung Disease, NA – not available.

Part B. Summary of GWAS data sources used in this study.

Trait	GWAS ID	Sample size	SNP counts	Ancestry	Link
FEV1/FVC ratio	GCST90292611	475,645	53,205,680	European	http://ftp.ebi.ac.uk/pub/databases/gwas/summary_statistics/GCST90292001-GCST90293000/GCST90292611/GCST90292611.tsv.gz
COPD	J10_COPD	21,617 cases / 372,627 controls	20,974,208	European	https://storage.googleapis.com/finngen-public-data-r11/summary_stats/finngen_R11_J10_COPD.gz

COPD – chronic obstructive pulmonary disease, FEV1/FVC – forced expiratory volume in one second/forced vital capacity, GWAS – genome-wide association study, ID – identifier, SNP – single nucleotide polymorphism.

Part C. Nucleotide primer sequences used for qRT-PCR validation.

Gene	Forward primer (5'–3')	Reverse primer (5'–3')
PIK3CG	GGCTACCATGAGCAGCTTACC	CTGTGAGGTCGGTGTTCCG
IL1B	TTCGACACATGGGATAACGAGG	TTTTTGCTGTGAGTCCCGGAG
UACA	GATGACCGATTGATGAAAGCAGC	CATCTAGTTTGCCTGGATTGACC
SFN	TGACGACAAGAAGCGCATCAT	GTAGTGGAAGACGGAAAAGTTCA
IRF1	ATGCCCATCACTCGGATGC	CCCTGCTTTGTATCGGCCTG
NTRK2	GCAATCCATTTACATGCTCCTGT	CATATTAGGAACCGGATCACCTG
TNFSF10	TGCGTGCTGATCGTGATCTTC	GCTCGTTGGTAAAGTACACGTA
GAPDH	GGAGCGAGATCCCTCCAAAAT	GGCTGTTGTCATACTTCTCATGG

GAPDH – glyceraldehyde-3-phosphate dehydrogenase, qRT-PCR – quantitative real-time polymerase chain reaction.

Table S4. Differentially expressed genes in lung tissues from patients with COPD compared with controls.

Gene	log_2FC	AveExpr	t	P-value	Adjusted P-value	B	Regulation
PNRC1	0.4065794	10.037082	11.553619	3.19E-24	4.02E-20	44.197434	Up
ZNF704	0.5422378	5.6619054	10.330286	1.71E-20	1.03E-16	35.84354	Up
KLF12	0.4608079	6.3712186	10.260234	2.78E-20	1.03E-16	35.37313	Up
WDR12	-0.424051	5.8753042	-10.07867	9.70E-20	2.45E-16	34.158665	Down
P2RY1	-0.609121	5.238938	-9.581237	2.86E-18	6.01E-15	30.870037	Down
P2RY14	0.5419856	8.0705381	9.4167006	8.62E-18	1.36E-14	29.795863	Up
PLCB4	0.6012334	6.9479468	9.1337367	5.67E-17	7.16E-14	27.965941	Up
TRPC6	0.7354795	6.9355556	9.1036171	6.92E-17	7.94E-14	27.772516	Up
FAH	-0.496137	6.6966185	-9.008386	1.30E-16	1.36E-13	27.162736	Down
BTG2	0.6754006	9.8585504	8.957017	1.82E-16	1.65E-13	26.834961	Up
CORIN	0.7065313	4.8134985	8.9499589	1.90E-16	1.65E-13	26.789987	Up
ZBTB20	0.4137199	8.8944986	8.8458976	3.76E-16	2.90E-13	26.128737	Up
RPS6KA2	-0.543178	8.6274901	-8.839675	3.91E-16	2.90E-13	26.089307	Down
RYR2	0.584361	5.7132287	8.764945	6.36E-16	4.46E-13	25.616716	Up
FRZB	0.8261233	6.2347001	8.6349423	1.48E-15	8.88E-13	24.798949	Up
PLXDC1	0.5898156	6.2725367	8.599422	1.86E-15	9.77E-13	24.576498	Up
FREM1	0.4210223	4.7448273	8.563597	2.34E-15	1.18E-12	24.352572	Up
CD1C	0.6954582	5.9474299	8.5360428	2.79E-15	1.31E-12	24.180645	Up
OLFM1	-0.588465	6.6424254	-8.529255	2.92E-15	1.31E-12	24.13833	Down
PSAT1	-0.715563	5.0521106	-8.508904	3.32E-15	1.45E-12	24.011563	Down
SHMT2	-0.531741	7.1413335	-8.499247	3.53E-15	1.46E-12	23.951463	Down
SLC18A2	0.676506	6.2623981	8.4923867	3.69E-15	1.46E-12	23.908786	Up
RTKN2	1.1466956	8.0426204	8.4713017	4.23E-15	1.56E-12	23.777723	Up
TRIB3	-0.530392	5.4849899	-8.467574	4.33E-15	1.56E-12	23.754568	Down

RNASE2	-0.890778	5.0177052	-8.448536	4.89E-15	1.71E-12	23.63639	Down
C1QTNF7	0.6712063	5.9762266	8.444269	5.03E-15	1.71E-12	23.609917	Up
SCNN1G	-0.645692	5.9833565	-8.401527	6.60E-15	1.97E-12	23.345127	Down
CXCL12	0.8660531	8.5682506	8.3894227	7.13E-15	1.97E-12	23.270258	Up
ENO1	-0.409626	9.6061334	-8.377618	7.69E-15	2.07E-12	23.197295	Down
SGIP1	0.5927589	6.7556036	8.3735805	7.89E-15	2.07E-12	23.172349	Up
CD163	-1.229878	8.6127139	-8.370165	8.06E-15	2.08E-12	23.151251	Down
HYOU1	-0.441193	8.0176625	-8.35462	8.90E-15	2.23E-12	23.055287	Down
CTSF	0.4026034	6.9202951	8.3499893	9.16E-15	2.23E-12	23.026717	Up
TRANK1	0.4176858	6.5865302	8.3496239	9.19E-15	2.23E-12	23.024463	Up
HLA-DPA1	0.4287241	12.134251	8.3434241	9.55E-15	2.28E-12	22.986225	Up
EIF4EBP1	-0.468183	6.8358749	-8.334245	1.01E-14	2.37E-12	22.929637	Down
AKR1C2	-0.759726	7.1019316	-8.272043	1.50E-14	3.33E-12	22.546986	Down
ARID5B	0.4149782	7.9809554	8.2341946	1.91E-14	4.15E-12	22.31485	Up
RFTN2	0.5077685	5.4815742	8.1853709	2.60E-14	5.46E-12	22.016191	Up
PCDH15	0.4759289	4.2921757	8.1768005	2.74E-14	5.67E-12	21.963858	Up
AGBL1	0.8413063	5.5615008	8.1529314	3.18E-14	6.38E-12	21.818253	Up
FXYD1	0.5253896	7.5091405	8.1419747	3.41E-14	6.73E-12	21.751489	Up
BMP5	0.6408553	7.06802	8.1266702	3.75E-14	7.29E-12	21.658307	Up
ANKRD44	0.4438174	7.1245942	8.1011262	4.41E-14	8.13E-12	21.502983	Up
LTBP4	0.529154	8.2818802	8.0928633	4.64E-14	8.37E-12	21.452793	Up
FUT3	-0.412799	4.3397285	-8.073187	5.25E-14	9.23E-12	21.333383	Down
GSTM5	0.6232722	6.3210591	8.0643095	5.55E-14	9.60E-12	21.279557	Up
GLT8D2	0.5803598	6.9912087	8.0405427	6.44E-14	1.08E-11	21.135604	Up
PLIN2	-0.566129	8.361868	-8.0191	7.36E-14	1.22E-11	21.00592	Down
FAM13C	0.6172671	6.918244	7.9959695	8.50E-14	1.36E-11	20.866228	Up

LTBP2	0.6371936	9.5738487	7.9717798	9.87E-14	1.56E-11	20.720367	Up
GTF2IRD1	-0.430811	6.207011	-7.921417	1.35E-13	2.03E-11	20.417436	Down
PYCR1	-0.422473	5.4494123	-7.881211	1.73E-13	2.49E-11	20.176325	Down
NFATC2	0.6282038	7.3009704	7.8768206	1.78E-13	2.49E-11	20.150037	Up
GCLM	-0.441982	5.792787	-7.866002	1.90E-13	2.64E-11	20.085288	Down
HGF	0.5026059	5.7047771	7.8345262	2.31E-13	3.07E-11	19.897189	Up
GPATCH4	-0.407076	5.4769959	-7.754363	3.78E-13	4.72E-11	19.419973	Down
CBS	-0.556815	6.1450122	-7.745075	4.00E-13	4.94E-11	19.36485	Down
PIM2	0.6527853	6.4410071	7.7342156	4.27E-13	5.17E-11	19.300453	Up
SIDT1	0.4093306	5.7614484	7.7137562	4.84E-13	5.77E-11	19.179258	Up
LRRC36	0.8598963	6.6956842	7.6611428	6.67E-13	7.80E-11	18.868407	Up
ZMAT1	0.4835913	6.7326435	7.576856	1.11E-12	1.21E-10	18.37289	Up
HBB	1.0347344	11.553912	7.5667385	1.18E-12	1.27E-10	18.313616	Up
KCNAB1	0.4944506	5.2149355	7.5573191	1.25E-12	1.33E-10	18.258473	Up
PRSS12	0.5456124	6.0095896	7.5388134	1.40E-12	1.45E-10	18.150248	Up
MAMLD1	-0.44281	6.0360803	-7.530548	1.47E-12	1.50E-10	18.101961	Down
ROBO2	0.6769599	6.5953548	7.5236398	1.53E-12	1.55E-10	18.061623	Up
PTGS2	1.0591417	8.8157605	7.5027564	1.74E-12	1.73E-10	17.939813	Up
ANK2	0.6156114	5.9406529	7.4963644	1.81E-12	1.77E-10	17.902568	Up
NAIP	-0.738277	5.6723187	-7.437578	2.57E-12	2.42E-10	17.560883	Down
PIGW	-0.484074	5.5717319	-7.419278	2.87E-12	2.67E-10	17.454828	Down
C9orf152	-0.702317	5.3029194	-7.417998	2.89E-12	2.67E-10	17.447419	Down
KRT7	-0.649827	8.0415796	-7.392854	3.36E-12	3.03E-10	17.301966	Down
STAMBPL1	0.503339	5.7957554	7.3559208	4.19E-12	3.70E-10	17.088837	Up
IDH1	-0.445295	8.7648038	-7.352905	4.27E-12	3.71E-10	17.071462	Down
NCKAP5	0.708386	7.6765084	7.3408391	4.58E-12	3.94E-10	17.001984	Up

GEM	0.8008511	7.8391568	7.3206483	5.17E-12	4.41E-10	16.885871	Up
TSHZ2	0.4500358	5.2348818	7.3173009	5.27E-12	4.47E-10	16.866639	Up
HMGCLL1	0.4535636	4.719204	7.3124082	5.43E-12	4.57E-10	16.838538	Up
TGFB2	0.6191248	6.5776384	7.3063159	5.63E-12	4.67E-10	16.803562	Up
AMPD1	0.8088826	4.4850965	7.2932842	6.08E-12	4.95E-10	16.728806	Up
LYZ	0.5798436	11.649893	7.2361501	8.52E-12	6.85E-10	16.401983	Up
SYTL2	0.4050242	6.9314385	7.2020203	1.04E-11	8.08E-10	16.207479	Up
GLDN	-0.736351	6.8839959	-7.197891	1.07E-11	8.17E-10	16.183985	Down
PKDCC	-0.438552	7.3047862	-7.191142	1.11E-11	8.45E-10	16.145597	Down
ABCA9	0.7549705	6.7577292	7.1857138	1.15E-11	8.67E-10	16.114743	Up
SRPRB	-0.444154	7.3480997	-7.17801	1.20E-11	8.83E-10	16.070974	Down
IL17RB	-0.609253	5.2242208	-7.177548	1.20E-11	8.83E-10	16.06835	Down
ITGA10	-0.711051	5.6483854	-7.12062	1.68E-11	1.17E-09	15.745804	Down
PHGDH	-0.487674	6.0991113	-7.102081	1.87E-11	1.29E-09	15.641102	Down
ADAMTS15	-0.415373	5.1511529	-7.077703	2.16E-11	1.43E-09	15.503669	Down
ECM2	0.5873485	6.4195267	7.0746623	2.20E-11	1.44E-09	15.486548	Up
PTGDS	0.6043775	9.9350153	7.0537366	2.48E-11	1.57E-09	15.36884	Up
EPHX2	0.4092061	5.2946836	7.0457238	2.60E-11	1.63E-09	15.323824	Up
STAT4	0.4914591	6.7631926	7.0318594	2.82E-11	1.75E-09	15.246007	Up
VSTM2L	-0.414117	5.7308522	-7.02374	2.95E-11	1.81E-09	15.20048	Down
SEMA3D	0.5877857	5.8414976	7.0141678	3.12E-11	1.88E-09	15.146846	Up
TIGIT	0.4322444	4.7215894	6.996515	3.46E-11	2.04E-09	15.048054	Up
EGR1	0.8906544	10.819503	6.9337214	4.96E-11	2.76E-09	14.697883	Up
BTN3A3	0.4557758	8.1711321	6.9317803	5.02E-11	2.78E-09	14.68709	Up
PIK3CG	0.4414754	6.5796545	6.8935898	6.25E-11	3.36E-09	14.475114	Up
ABCA6	0.6038686	7.796116	6.8702679	7.14E-11	3.77E-09	14.346026	Up

SDR16C5	-0.720948	8.0892031	-6.851068	7.97E-11	4.13E-09	14.239959	Down
PDE7B	0.4786483	5.5244041	6.848147	8.11E-11	4.18E-09	14.223838	Up
HLA-DOA	0.4681252	6.9083336	6.8383797	8.57E-11	4.40E-09	14.169966	Up
HLA-DOB	0.4858778	5.1782768	6.8336628	8.81E-11	4.48E-09	14.143967	Up
TXLNB	0.4151389	4.7012643	6.827069	9.14E-11	4.64E-09	14.107641	Up
BTN3A1	0.4145099	7.9136206	6.7975436	1.08E-10	5.46E-09	13.945257	Up
SIGLEC10	-0.669026	5.8595662	-6.771999	1.25E-10	6.19E-09	13.805124	Down
TIMP4	-0.624348	4.9964939	-6.747293	1.44E-10	7.01E-09	13.669911	Down
JUN	0.6372838	9.6687278	6.7443664	1.46E-10	7.10E-09	13.653917	Up
DUOX1	-0.620195	7.9293121	-6.73364	1.55E-10	7.52E-09	13.595329	Down
WEE1	0.5148477	7.3909185	6.7285396	1.60E-10	7.68E-09	13.567489	Up
APLNR	0.9034219	5.8327265	6.7168474	1.71E-10	8.14E-09	13.503722	Up
CNTN1	0.553923	4.9250323	6.6997377	1.88E-10	8.87E-09	13.410538	Up
ABCA8	0.5992499	7.08636	6.694229	1.94E-10	9.01E-09	13.380568	Up
PRR15L	-0.723691	6.792672	-6.681032	2.09E-10	9.60E-09	13.308835	Down
BNIPL	0.4020848	4.9437623	6.6785569	2.12E-10	9.70E-09	13.295392	Up
TRPC1	0.4239009	6.6187972	6.6355377	2.70E-10	1.18E-08	13.06225	Up
VCAN	-0.588266	9.2070628	-6.632562	2.75E-10	1.20E-08	13.046157	Down
MMRN1	-0.743126	7.1441792	-6.616944	3.00E-10	1.30E-08	12.961783	Down
DHCR7	-0.479024	7.2564301	-6.599162	3.31E-10	1.40E-08	12.865872	Down
MFAP4	0.6380807	10.419805	6.5913166	3.46E-10	1.46E-08	12.823611	Up
PAMR1	0.6137419	5.4292121	6.5795769	3.69E-10	1.54E-08	12.760432	Up
NQO2	-0.401427	7.3569281	-6.571259	3.87E-10	1.60E-08	12.715713	Down
ITGA4	0.4356564	7.0672322	6.5553197	4.23E-10	1.73E-08	12.630121	Up
HDC	0.5672446	5.863949	6.5541667	4.25E-10	1.73E-08	12.623934	Up
PKHD1L1	-0.815773	5.4208527	-6.548169	4.40E-10	1.79E-08	12.591767	Down

CNR1	-0.510891	4.5373617	-6.532791	4.79E-10	1.91E-08	12.509379	Down
CD177	-0.461832	4.9452055	-6.517166	5.22E-10	2.07E-08	12.425796	Down
PPP1R10	0.4387566	7.9062499	6.5026704	5.66E-10	2.21E-08	12.348372	Up
SLCO4A1	-0.949567	6.7608897	-6.489978	6.07E-10	2.35E-08	12.28067	Down
GXYLT2	0.5856744	7.0088984	6.4819218	6.35E-10	2.42E-08	12.237748	Up
EGR2	0.8629764	7.949166	6.4810744	6.38E-10	2.43E-08	12.233235	Up
CSPG5	-0.45182	4.6102342	-6.473447	6.65E-10	2.51E-08	12.19263	Down
HPGDS	0.5662543	7.2960557	6.4373266	8.12E-10	3.02E-08	12.000774	Up
CCL5	0.6921514	8.8017108	6.4308602	8.41E-10	3.11E-08	11.966502	Up
FASN	-0.439106	7.3941101	-6.414045	9.22E-10	3.38E-08	11.877489	Down
HRH2	-0.440634	4.8733459	-6.395452	1.02E-09	3.67E-08	11.779249	Down
SERPINF2	-0.418261	5.3958098	-6.347105	1.33E-09	4.66E-08	11.524686	Down
SEMA6D	0.5901922	6.5552712	6.3460004	1.34E-09	4.67E-08	11.518884	Up
RHOBTB2	-0.406668	7.9241672	-6.323318	1.51E-09	5.24E-08	11.399915	Down
EGR3	0.8198675	6.9944129	6.3038622	1.68E-09	5.73E-08	11.298098	Up
SKAP1	0.4344784	5.5758896	6.3002521	1.72E-09	5.81E-08	11.279228	Up
FOSB	1.3536159	9.0595427	6.2708004	2.01E-09	6.63E-08	11.125564	Up
ANGPTL1	-0.503721	6.2863896	-6.262184	2.11E-09	6.90E-08	11.080699	Down
DPYSL3	0.4031857	7.6731388	6.2602603	2.13E-09	6.93E-08	11.07069	Up
DUOXA1	-0.456569	7.4563318	-6.253973	2.20E-09	7.12E-08	11.03799	Down
TNR	0.5771129	4.1426273	6.2511965	2.24E-09	7.21E-08	11.023552	Up
LRRTM4	0.4499782	4.7461219	6.2318055	2.48E-09	7.90E-08	10.922862	Up
NFKBIZ	0.5818068	9.1615466	6.2248094	2.58E-09	8.14E-08	10.886586	Up
DDR2	0.44415	8.5102284	6.2188759	2.66E-09	8.37E-08	10.855842	Up
TMEM176A	0.423993	8.2198796	6.2188039	2.66E-09	8.37E-08	10.855469	Up
CLEC2D	0.4323737	6.1189942	6.2059431	2.86E-09	8.90E-08	10.7889	Up

TAGAP	0.4722719	5.9377767	6.1891027	3.13E-09	9.70E-08	10.701876	Up
RGS5	0.6869001	9.2958647	6.1693513	3.47E-09	1.07E-07	10.600015	Up
GKN2	-0.622296	9.0988778	-6.16678	3.52E-09	1.08E-07	10.586771	Down
IER2	0.5398182	8.9483539	6.1640135	3.58E-09	1.10E-07	10.572526	Up
CYSLTR1	0.4580855	6.239478	6.1531987	3.79E-09	1.14E-07	10.51688	Up
SPEF2	0.4562491	4.7207422	6.1490973	3.87E-09	1.16E-07	10.495795	Up
ST6GALNAC3	-0.48585	6.2570955	-6.144518	3.97E-09	1.17E-07	10.472266	Down
SERPINA3	-0.80479	8.4772813	-6.142118	4.02E-09	1.19E-07	10.459939	Down
TRAF3IP3	0.4850219	6.21556	6.127449	4.35E-09	1.26E-07	10.384662	Up
JUNB	0.611935	9.8016085	6.1095455	4.78E-09	1.37E-07	10.292957	Up
CASQ2	0.5845889	5.9664944	6.107715	4.83E-09	1.38E-07	10.283592	Up
ISLR	0.4025476	6.4653213	6.0976039	5.09E-09	1.45E-07	10.231894	Up
HMCN1	0.4680147	7.9491045	6.0949314	5.17E-09	1.46E-07	10.21824	Up
SLIT3	0.4078471	8.1383037	6.0906846	5.28E-09	1.48E-07	10.19655	Up
STAP1	0.4618344	4.1420728	6.0891016	5.33E-09	1.49E-07	10.188468	Up
JUND	0.4863403	8.3734262	6.0810001	5.56E-09	1.55E-07	10.147129	Up
BMP1	0.609579	6.6575683	6.0723516	5.82E-09	1.60E-07	10.10304	Up
CPA3	0.6014999	8.7495942	6.0602227	6.21E-09	1.67E-07	10.041283	Up
TRIL	0.4389617	5.5051963	6.0469153	6.66E-09	1.77E-07	9.9736233	Up
ACE	0.5277094	6.5930893	6.0171717	7.79E-09	2.04E-07	9.822773	Up
PLEKHH2	0.5859055	8.702842	6.0112789	8.04E-09	2.09E-07	9.7929487	Up
ZDHHC9	-0.430196	8.3460085	-6.009685	8.11E-09	2.10E-07	9.7848842	Down
PGC	-0.800149	10.887622	-5.98957	9.01E-09	2.28E-07	9.6832523	Down
MS4A1	0.9440874	5.120713	5.9709091	9.94E-09	2.49E-07	9.5891814	Up
SLC39A14	-0.46643	6.094715	-5.951568	1.10E-08	2.71E-07	9.4919007	Down
CD79A	0.6870703	5.316486	5.9388704	1.18E-08	2.86E-07	9.4281543	Up

LAX1	0.4307948	5.6128868	5.9386942	1.18E-08	2.86E-07	9.4272705	Up
IL2RG	0.4932516	8.139625	5.9347222	1.20E-08	2.90E-07	9.4073501	Up
FGD2	-0.413067	5.6195605	-5.90158	1.43E-08	3.39E-07	9.2415057	Down
COL6A6	0.6138197	7.8966671	5.8895676	1.52E-08	3.57E-07	9.1815564	Up
SH3PXD2B	-0.478132	7.0960453	-5.846997	1.89E-08	4.28E-07	8.9698106	Down
ORM1	-0.711968	4.5006634	-5.846083	1.90E-08	4.29E-07	8.9652746	Down
HLF	0.4397972	7.9618269	5.8297649	2.07E-08	4.61E-07	8.8844079	Up
HMGB3	-0.457046	5.5465439	-5.808246	2.31E-08	5.08E-07	8.7780157	Down
TRAT1	0.6624176	6.166475	5.8058658	2.34E-08	5.13E-07	8.766265	Up
CRISPLD2	-0.475736	8.6722174	-5.783431	2.63E-08	5.67E-07	8.6556794	Down
SLAMF7	0.8116551	6.5950387	5.7780848	2.70E-08	5.82E-07	8.6293712	Up
CD96	0.4466333	6.3429027	5.7649097	2.89E-08	6.16E-07	8.5646148	Up
MS4A2	0.6097749	6.1869459	5.7582751	2.99E-08	6.31E-07	8.5320453	Up
CTSE	-0.722273	8.278303	-5.732414	3.41E-08	7.06E-07	8.4053538	Down
NLRC4	-0.468478	5.2727434	-5.728843	3.47E-08	7.16E-07	8.3878918	Down
NELL2	0.5501899	5.7993972	5.7286419	3.48E-08	7.16E-07	8.386908	Up
TNFRSF11B	-0.594493	5.2605241	-5.728413	3.48E-08	7.16E-07	8.3857889	Down
DPP4	-0.456616	7.4930367	-5.726758	3.51E-08	7.21E-07	8.3776974	Down
SRPX	-0.639201	8.5976716	-5.726189	3.52E-08	7.22E-07	8.3749191	Down
ALPL	-0.49089	8.1335609	-5.725014	3.54E-08	7.25E-07	8.3691759	Down
MTHFD2	-0.436823	7.61744	-5.718138	3.67E-08	7.46E-07	8.3355937	Down
TACC2	-0.50687	7.3110607	-5.716492	3.70E-08	7.50E-07	8.327559	Down
ICOS	0.5457914	5.0195725	5.7158649	3.71E-08	7.50E-07	8.3244966	Up
HMOX1	-0.698196	8.290705	-5.712815	3.77E-08	7.59E-07	8.3096152	Down
FMN1	-0.40923	5.8375236	-5.707142	3.88E-08	7.79E-07	8.2819484	Down
UACA	0.4479954	7.6773052	5.6988995	4.04E-08	8.07E-07	8.2417825	Up

CD1E	0.4096315	5.204608	5.6963008	4.10E-08	8.14E-07	8.2291281	Up
OTUD1	0.4063765	7.88748	5.6903971	4.22E-08	8.34E-07	8.2003963	Up
CAPN3	0.4297422	7.0070741	5.6899692	4.23E-08	8.35E-07	8.1983148	Up
SLC7A11	-0.61412	5.5379946	-5.684861	4.34E-08	8.54E-07	8.1734741	Down
NQO1	-0.488683	7.0180556	-5.682014	4.41E-08	8.65E-07	8.1596355	Down
TMEM130	0.5204697	5.8536924	5.674331	4.58E-08	8.93E-07	8.122317	Up
ZFP36	0.5184164	10.988889	5.6431247	5.36E-08	1.02E-06	7.9711177	Up
CD83	0.6282172	7.7646437	5.6401223	5.45E-08	1.03E-06	7.9566028	Up
RARRES2	0.445125	9.3424545	5.6384759	5.49E-08	1.04E-06	7.9486457	Up
IER3	0.6820813	9.1244732	5.6370197	5.53E-08	1.04E-06	7.9416096	Up
IL1B	0.7560509	7.1539523	5.6074595	6.42E-08	1.18E-06	7.7990628	Up
MS4A4A	-0.457894	8.2110607	-5.606404	6.45E-08	1.19E-06	7.793984	Down
FGR	-0.410047	8.293266	-5.591527	6.95E-08	1.26E-06	7.7224588	Down
ADAM28	0.4064423	5.5138817	5.5482641	8.63E-08	1.54E-06	7.5152678	Up
SAMD3	0.4115108	4.8285153	5.5415582	8.92E-08	1.58E-06	7.4832587	Up
NR4A3	0.8755043	7.6921567	5.5271079	9.59E-08	1.68E-06	7.4143809	Up
SIGLEC1	-0.498908	6.8776249	-5.525901	9.65E-08	1.68E-06	7.4086357	Down
GPR4	-0.442958	6.3684283	-5.519973	9.94E-08	1.73E-06	7.3804211	Down
AKR1C3	-0.526403	7.6192014	-5.518773	1.00E-07	1.74E-06	7.3747109	Down
CCL23	-0.557952	5.0646776	-5.514642	1.02E-07	1.77E-06	7.3550671	Down
STAB1	-0.437989	7.3938565	-5.496721	1.12E-07	1.91E-06	7.2699761	Down
VSIG4	-0.644689	9.2091219	-5.494737	1.13E-07	1.92E-06	7.2605701	Down
FAM162B	0.4929177	8.3056416	5.4773771	1.23E-07	2.07E-06	7.1783586	Up
SULF1	0.4347551	6.9357435	5.4732839	1.25E-07	2.11E-06	7.1590033	Up
SLC14A1	0.6292354	5.2581587	5.4548608	1.37E-07	2.30E-06	7.0720195	Up
LYVE1	-0.609744	8.6728577	-5.445674	1.43E-07	2.39E-06	7.028724	Down

SFN	-0.585894	8.0360481	-5.433654	1.52E-07	2.49E-06	6.9721627	Down
LRRN3	0.6374509	7.2263496	5.3981725	1.81E-07	2.90E-06	6.8057465	Up
LY9	0.459521	5.4688791	5.3914843	1.87E-07	2.98E-06	6.774469	Up
SHISA3	0.5343256	6.1921751	5.350163	2.29E-07	3.53E-06	6.581877	Up
IL2RB	0.4755889	7.2579	5.3479907	2.31E-07	3.54E-06	6.5717833	Up
VCAM1	0.8047609	7.1862017	5.3479693	2.31E-07	3.54E-06	6.5716836	Up
IL1R2	-1.077394	6.6508483	-5.333601	2.48E-07	3.75E-06	6.5049982	Down
FPR1	-0.620245	7.8480545	-5.328739	2.54E-07	3.82E-06	6.4824633	Down
PYHIN1	0.4101333	6.2311026	5.2904345	3.06E-07	4.53E-06	6.3054752	Up
CD27	0.5900915	6.2677095	5.2871965	3.11E-07	4.59E-06	6.2905579	Up
CD69	0.5324603	8.1418152	5.2807693	3.20E-07	4.69E-06	6.2609694	Up
BCAT1	-0.536721	7.549464	-5.265771	3.44E-07	4.99E-06	6.1920298	Down
LCN2	-0.764053	6.7598321	-5.256852	3.59E-07	5.18E-06	6.1511038	Down
P2RY10	0.5272792	4.9695938	5.2532898	3.66E-07	5.26E-06	6.1347734	Up
KRT19	-0.403193	8.6138077	-5.244402	3.82E-07	5.47E-06	6.0940656	Down
GZMK	0.6524882	6.4851651	5.2340384	4.01E-07	5.72E-06	6.0466632	Up
FAT3	0.4698451	5.6167439	5.2189247	4.31E-07	6.09E-06	5.9776643	Up
RABGAP1L	0.4166061	6.4999489	5.214559	4.40E-07	6.19E-06	5.9577619	Up
AQP9	-0.567312	8.1775866	-5.212727	4.44E-07	6.22E-06	5.949414	Down
SEC14L4	-0.422533	5.9460473	-5.19972	4.72E-07	6.59E-06	5.8902083	Down
MYH10	0.559043	9.8235688	5.1971952	4.78E-07	6.65E-06	5.8787302	Up
RHOH	0.4188604	6.214702	5.1522612	5.92E-07	7.97E-06	5.6751535	Up
ICAM4	0.6674846	6.0199363	5.1503769	5.97E-07	8.04E-06	5.6666462	Up
ANO1	0.5372903	7.3938234	5.139504	6.29E-07	8.39E-06	5.6176043	Up
CD48	0.4422137	7.4950781	5.1336099	6.46E-07	8.58E-06	5.5910526	Up
ABI3BP	0.448214	8.8411513	5.1331043	6.48E-07	8.58E-06	5.5887764	Up

DCN	0.4097526	10.582635	5.1328496	6.49E-07	8.58E-06	5.5876296	Up
COL8A1	-0.442397	7.4040792	-5.112029	7.16E-07	9.36E-06	5.4940378	Down
LIPG	-0.66783	5.3593534	-5.105416	7.38E-07	9.63E-06	5.4643698	Down
FCGR2B	0.4114274	7.776599	5.095263	7.74E-07	1.00E-05	5.4188846	Up
PCSK2	-0.465059	5.2058397	-5.079853	8.32E-07	1.06E-05	5.3499784	Down
RASGRP1	0.4795277	7.2220593	5.0788641	8.36E-07	1.07E-05	5.3455634	Up
S100A8	-0.619955	9.3689998	-5.071019	8.67E-07	1.10E-05	5.3105529	Down
SULT1B1	-0.438696	4.4817854	-5.0555	9.33E-07	1.17E-05	5.2414165	Down
ADAMTS9	-0.576899	7.5629786	-5.05473	9.36E-07	1.18E-05	5.2379902	Down
SLC1A3	-0.510884	6.1212399	-5.051524	9.50E-07	1.19E-05	5.22373	Down
CTH	-0.402442	4.9117035	-5.036213	1.02E-06	1.27E-05	5.1557264	Down
THEMIS	0.4712586	5.6989726	4.9913558	1.26E-06	1.50E-05	4.9574228	Up
TFEC	-0.437922	6.5605042	-4.908536	1.84E-06	2.08E-05	4.5949591	Down
NR1D2	0.4058676	8.0362555	4.9023005	1.89E-06	2.13E-05	4.5678629	Up
ETV5	0.4082715	7.8111667	4.9013747	1.90E-06	2.14E-05	4.5638423	Up
TMTC1	-0.424199	7.6896904	-4.900785	1.91E-06	2.14E-05	4.5612815	Down
DTL	-0.446161	5.0761735	-4.894967	1.96E-06	2.19E-05	4.5360305	Down
RGS13	0.4276612	5.4752488	4.8949528	1.96E-06	2.19E-05	4.5359687	Up
CPE	0.4184405	8.0394807	4.8882877	2.02E-06	2.25E-05	4.5070705	Up
TRIB1	0.5603065	8.4611418	4.8828422	2.07E-06	2.30E-05	4.4834828	Up
ATF3	0.683904	7.3817054	4.8820744	2.08E-06	2.30E-05	4.480159	Up
KLRG1	0.4089211	5.5893087	4.849671	2.40E-06	2.60E-05	4.3402515	Up
DDIT4	-0.554942	8.078067	-4.847904	2.42E-06	2.62E-05	4.332644	Down
FOS	0.5968789	11.008394	4.8375072	2.54E-06	2.73E-05	4.2879232	Up
TNC	-0.63999	7.3829022	-4.808706	2.89E-06	3.07E-05	4.1644401	Down
NRXN3	0.4183884	5.9056926	4.7970529	3.05E-06	3.22E-05	4.1146443	Up

C1R	-0.407769	9.9737746	-4.763613	3.54E-06	3.68E-05	3.9722866	Down
TNIP3	0.6805306	4.8808248	4.7549008	3.68E-06	3.81E-05	3.9353312	Up
TFCP2L1	-0.408933	6.2186955	-4.744564	3.86E-06	3.95E-05	3.8915547	Down
IRF1	0.4237621	8.2446015	4.7322319	4.08E-06	4.14E-05	3.8394236	Up
CD5	0.4004645	5.4075443	4.7074751	4.55E-06	4.54E-05	3.7351039	Up
CD3G	0.5315812	5.9341973	4.6993377	4.72E-06	4.68E-05	3.7009112	Up
PLVAP	-0.508234	6.2763568	-4.694777	4.81E-06	4.77E-05	3.6817688	Down
INHBA	0.5112159	7.7129244	4.673866	5.28E-06	5.15E-05	3.594189	Up
HIGD1B	0.4616924	7.8686846	4.6687078	5.40E-06	5.25E-05	3.5726341	Up
MAOA	-0.405313	9.4658494	-4.66424	5.51E-06	5.32E-05	3.5539781	Down
ANPEP	-0.415355	6.7300018	-4.64639	5.96E-06	5.69E-05	3.4795944	Down
SEMA6A	0.4654975	8.0293839	4.6236486	6.58E-06	6.22E-05	3.3851622	Up
TNFSF10	0.5125816	10.27213	4.6135196	6.88E-06	6.44E-05	3.3432233	Up
CD2	0.4114313	7.355196	4.58884	7.66E-06	7.03E-05	3.2413502	Up
CD3E	0.4113651	6.233611	4.5831354	7.85E-06	7.17E-05	3.2178661	Up
MYH11	0.4641862	9.2331781	4.5734513	8.19E-06	7.40E-05	3.178054	Up
SYBU	-0.401488	7.1135254	-4.503415	1.11E-05	9.57E-05	2.8921777	Down
TPPP3	0.4623956	8.5984794	4.4996163	1.13E-05	9.71E-05	2.8767771	Up
LUM	0.4628608	10.575	4.4512806	1.38E-05	0.0001156	2.6817296	Up
CFTR	0.4231631	5.9023848	4.4184401	1.59E-05	0.0001304	2.550201	Up
SLC11A1	-0.445107	7.6208544	-4.413401	1.63E-05	0.000133	2.5300894	Down
DPT	0.4235215	7.5428579	4.401775	1.71E-05	0.0001381	2.4837637	Up
PI3	-0.453815	5.3098137	-4.388708	1.80E-05	0.0001445	2.4318163	Down
ASPN	0.5478406	6.4552559	4.3822139	1.85E-05	0.0001481	2.4060471	Up
COL14A1	0.4084845	6.7462986	4.3609016	2.03E-05	0.0001601	2.3216999	Up
CH25H	0.6165607	7.6115146	4.3169747	2.44E-05	0.0001861	2.148931	Up

OGN	0.5717004	7.5010183	4.3150588	2.46E-05	0.0001874	2.1414288	Up
CP	0.5406063	6.253451	4.2374976	3.38E-05	0.0002481	1.8400563	Up
ALOX15B	-0.523747	7.5229441	-4.237104	3.39E-05	0.0002483	1.8385395	Down
MARCO	-0.438319	8.4335073	-4.233495	3.44E-05	0.0002514	1.8246286	Down
SAA1	-0.532583	3.5781635	-4.217844	3.67E-05	0.0002656	1.7644176	Down
LILRA6	-0.405094	6.0884049	-4.177873	4.31E-05	0.0003064	1.6115027	Down
IL6	0.8733479	8.4891392	4.1746741	4.37E-05	0.0003095	1.599317	Up
AZGP1	0.6045521	6.3649378	4.1719542	4.42E-05	0.0003123	1.5889629	Up
PDLIM3	0.4193219	7.7884556	4.1545268	4.74E-05	0.0003308	1.5227552	Up
NR4A1	0.5103014	9.6059015	4.1541513	4.75E-05	0.0003311	1.5213313	Up
ERP27	0.5147303	7.0034318	4.1286859	5.26E-05	0.0003601	1.4250159	Up
NTRK2	0.515956	5.5186096	4.1008042	5.89E-05	0.0003967	1.3201373	Up
FPR2	-0.542145	6.3618614	-4.096254	5.99E-05	0.0004024	1.3030775	Down
CLEC4E	-0.517512	5.8360346	-4.084473	6.28E-05	0.0004183	1.2589883	Down
CXCL1	0.6612838	7.9587301	4.0747965	6.53E-05	0.0004325	1.2228525	Up
CD3D	0.4068623	7.291899	4.0498255	7.21E-05	0.0004717	1.129941	Up
PTX3	-0.866399	6.5617238	-4.025422	7.94E-05	0.0005081	1.0396113	Down
FKBP5	-0.622659	9.3554879	-3.991377	9.08E-05	0.0005685	0.9143759	Down
AOX1	-0.517886	6.9245784	-3.972189	9.78E-05	0.0006074	0.8441924	Down
CXCL2	0.6076761	8.7658444	3.9543409	0.0001049	0.0006451	0.7791709	Up
S100A12	-0.681079	6.5024567	-3.922659	0.0001186	0.0007176	0.6643712	Down
GBP5	0.5052677	6.6442559	3.8993284	0.0001298	0.000774	0.5803436	Up
HLA-DQA1	0.6834483	9.8260978	3.8915656	0.0001337	0.0007956	0.5524804	Up
CX3CR1	0.55668	6.7177106	3.8905572	0.0001342	0.0007983	0.5488646	Up
CX3CL1	0.4247366	8.6731569	3.8868061	0.0001362	0.0008084	0.5354207	Up
SEMA3G	0.4809109	7.1611825	3.7832001	0.0002019	0.0011345	0.1685462	Up

MS4A14	-0.428405	5.972537	-3.753521	0.0002257	0.0012431	0.0650391	Down
GBP4	0.4067616	7.8370572	3.7229203	0.0002529	0.0013694	-0.040938	Up
PLA2G2A	-0.633263	6.0916138	-3.642433	0.0003402	0.0017627	-0.316052	Down
SOCS3	0.490198	8.7148914	3.499523	0.000569	0.0027313	-0.791465	Up
CHIT1	0.7671778	6.4338203	3.4431591	0.0006939	0.003202	-0.974325	Up
GSTT1	-0.61625	6.7798678	-3.391236	0.0008312	0.0037242	-1.140434	Down
CCL2	0.6897288	10.518696	3.3709198	0.0008916	0.0039454	-1.204813	Up
MUC15	0.4040783	6.7482398	3.3511988	0.000954	0.0041608	-1.266976	Up
HAS2	0.4966277	6.6364393	3.3379601	0.0009982	0.0043282	-1.308521	Up
DNAH5	-0.407773	5.496652	-3.314528	0.0010812	0.0046322	-1.381695	Down
NR4A2	0.4705199	8.635287	3.2791284	0.0012187	0.0051361	-1.49136	Up
CSF3	0.5233784	6.5671537	3.2246517	0.0014626	0.0060034	-1.65805	Up
MGAM	-0.466823	5.7008682	-3.198985	0.0015925	0.0064363	-1.73571	Down
FGA	-0.541815	4.9945161	-3.11634	0.0020871	0.008081	-1.981948	Down
HLA-DQB1	0.5283864	8.4794053	2.9351506	0.0037048	0.01311	-2.50118	Up
CXCL10	0.6040983	7.8518158	2.8801083	0.0043872	0.0150964	-2.653256	Up
CCL8	0.4648299	5.8141118	2.8041198	0.0055184	0.0182497	-2.858832	Up
SLC6A4	0.580182	8.5870783	2.6913179	0.0076902	0.0240576	-3.154592	Up
POSTN	-0.472944	7.6114977	-2.677202	0.0080104	0.0248745	-3.190806	Down
CXCL9	0.6105809	7.80966	2.6559071	0.0085163	0.026175	-3.245103	Up
MT1X	-0.406097	10.461644	-2.652576	0.008598	0.0263939	-3.253561	Down
SELE	0.550864	6.3892965	2.6083217	0.0097521	0.0293097	-3.364969	Up
IDO1	0.4160245	7.5112034	2.5638651	0.0110497	0.0325662	-3.47512	Up
CCL18	-0.407341	10.129865	-2.330533	0.0207266	0.0546676	-4.023965	Down
MT1M	-0.411966	9.9634137	-2.092059	0.0376346	0.0890841	-4.533464	Down

AveExpr – average expression, B – log-odds of differential expression, COPD – chronic obstructive pulmonary disease, FC – fold change, FDR – false discovery rate, log2FC – log2-transformed fold change, t – moderated t-statistic.

Table S5. GO enrichment analysis for the identified DEGs.

Ontology	ID	Description	Gene ratio	Background ratio	P-value	Adjusted P-value	q-value	Gene ID	Count
BP	GO:0007159	leukocyte cell-cell adhesion	37/340	415/18614	1.50E-15	4.06E-12	3.08E-12	CXCL12/HLA-DPA1/TIGIT/HLA-DOA/HLA-DOB/ITGA4/CD177/CCL5/EGR3/SKAP1/NFKBIZ/LAX1/IL2RG/DPP4/ICOS/CD83/IL1B/NR4A3/VSIG4/VCAM1/CD27/RHOH/FCGR2B/RASGRP1/S100A8/IRF1/CD5/CD3E/IL6/HLA-DQA1/CX3CR1/PLA2G2A/CCL2/HAS2/HLA-DQB1/SELE/IDO1	37
BP	GO:0022407	regulation of cell-cell adhesion	40/340	491/18614	2.13E-15	4.06E-12	3.08E-12	CXCL12/HLA-DPA1/FUT3/TIGIT/HLA-DOA/HLA-DOB/MMRN1/ITGA4/CCL5/SERPINF2/EGR3/SKAP1/TNR/NFKBIZ/LAX1/IL2RG/DPP4/ICOS/CD83/IL1B/NR4A3/VSIG4/VCAM1/CD27/RHOH/FCGR2B/RASGRP1/IRF1/CD5/CD3E/IL6/HLA-DQA1/CX3CL1/PLA2G2A/CCL2/HAS2/FGA/HLA-DQB1/SELE/IDO1	40
BP	GO:0045785	positive regulation of cell adhesion	39/340	482/18614	6.13E-15	7.79E-12	5.91E-12	CXCL12/HLA-DPA1/FUT3/TGFB2/ECM2/HLA-DOA/HLA-DOB/MMRN1/ITGA4/CSPG5/CCL5/SERPINF2/EGR3/SKAP1/NFKBIZ/IL2RG/DPP4/ICOS/FMN1/CD83/IL1B/NR4A3/VCAM1/CD27/RHOH/ABI3BP/COL8A1/RASGRP1/CD5/CD3E/SAA1/IL6/HLA-DQA1/CX3CL1/CCL2/HAS2/FGA/HLA-DQB1/SELE	39

BP	GO:1903037	regulation of leukocyte cell-cell adhesion	34/340	378/18614	1.75E-14	1.66E-11	1.26E-11	CXCL12/HLA-DPA1/TIGIT/HLA-DOA/HLA-DOB/ITGA4/CCL5/EGR3/SKAP1/NFKBIZ/LAX1/IL2RG/DPP4/ICOS/CD83/IL1B/NR4A3/VSIG4/VCAM1/CD27/RHOH/FCGR2B/RASGRP1/IRF1/CD5/CD3E/IL6/HLA-DQA1/PLA2G2A/CCL2/HAS2/HLA-DQB1/SELE/IDO1	34
BP	GO:0022409	positive regulation of cell-cell adhesion	31/340	321/18614	3.89E-14	2.97E-11	2.25E-11	HLA-DPA1/FUT3/HLA-DOA/HLA-DOB/MMRN1/ITGA4/CCL5/SERPINF2/EGR3/SKAP1/NFKBIZ/IL2RG/DPP4/ICOS/CD83/IL1B/NR4A3/VCAM1/CD27/RHOH/RASGRP1/CD5/CD3E/IL6/HLA-DQA1/CX3CL1/CCL2/HAS2/FGA/HLA-DQB1/SELE	31
BP	GO:0002237	response to molecule of bacterial origin	32/340	366/18614	2.35E-13	1.49E-10	1.13E-10	PTGS2/TIMP4/CNR1/CCL5/NFKBIZ/STAP1/JUND/ACE/CD96/ALPL/NQO1/ZFP36/IL1B/VCAM1/FCGR2B/S100A8/TRIB1/FOS/TNIP3/SLC11A1/IL6/NR4A1/CXCL1/CXCL2/CX3CR1/CX3CL1/CCL2/CSF3/CXCL10/CXCL9/SELE/IDO1	32
BP	GO:0032496	response to lipopolysaccharide	31/340	345/18614	2.73E-13	1.49E-10	1.13E-10	PTGS2/TIMP4/CNR1/CCL5/NFKBIZ/STAP1/JUND/ACE/CD96/ALPL/NQO1/ZFP36/IL1B/VCAM1/S100A8/TRIB1/FOS/TNIP3/SLC11A1/IL6/NR4A1/CXCL1/CXCL2/CX3CR1/CX3CL1/CCL2/CSF3/CXCL10/CXCL9/SELE/IDO1	31

BP	GO:0050900	leukocyte migration	32/340	393/18614	1.62E-12	7.73E-10	5.87E-10	CXCL12/BMP5/TGFB2/PIK3CG/ITGA4/CD177/CCL5/STAP1/DPP4/HMOX1/RARRES2/IL1B/CCL23/LYVE1/VCAM1/S100A8/PLVAP/CH25H/SAA1/IL6/FPR2/CXCL1/CXCL2/S100A12/CX3CR1/CX3CL1/CCL2/CXCL10/CCL8/CXCL9/SELE/CCL18	32
BP	GO:0060326	cell chemotaxis	28/340	312/18614	4.21E-12	1.78E-09	1.35E-09	CXCL12/HGF/TGFB2/PIK3CG/CCL5/EGR3/STAP1/DPP4/RARRES2/IL1B/CCL23/VCAM1/S100A8/CH25H/SAA1/IL6/NR4A1/FPR2/CXCL1/CXCL2/S100A12/CX3CR1/CX3CL1/CCL2/CXCL10/CCL8/CXCL9/CCL18	28
BP	GO:1903039	positive regulation of leukocyte cell-cell adhesion	26/340	273/18614	6.60E-12	2.52E-09	1.91E-09	HLA-DPA1/HLA-DOA/HLA-DOB/ITGA4/CCL5/EGR3/SKAP1/NFKBIZ/IL2RG/DPP4/ICOS/CD83/IL1B/NR4A3/VCAM1/CD27/RHOH/RASGRP1/CD5/CD3E/IL6/HLA-DQA1/CCL2/HAS2/HLA-DQB1/SELE	26
BP	GO:0030593	neutrophil chemotaxis	17/340	107/18614	9.86E-12	3.41E-09	2.59E-09	TGFB2/PIK3CG/CCL5/DPP4/IL1B/CCL23/S100A8/SAA1/CXCL1/CXCL2/S100A12/CX3CL1/CCL2/CXCL10/CCL8/CXCL9/CCL18	17
BP	GO:0050863	regulation of T cell activation	30/340	377/18614	1.49E-11	4.64E-09	3.52E-09	HLA-DPA1/TIGIT/HLA-DOA/HLA-DOB/CCL5/EGR3/NFKBIZ/JUNB/LAX1/IL2RG/DPP4/ICOS/CD83/IL1B/VSIG4/VCAM1/CD27/RHOH/FCGR2B/RASGRP1/IRF1/CD5/CD2/CD3E/IL6/HLA-DQA1/PLA2G2A/CCL2/HLA-DQB1/IDO1	30

BP	GO:0030595	leukocyte chemotaxis	24/340	241/18614	1.71E-11	4.64E-09	3.52E-09	CXCL12/TGFB2/PIK3CG/CCL5/STAP1/DPP4/RARRES2/IL1B/CCL23/S100A8/CH25H/SAA1/IL6/FPR2/CXCL1/CXCL2/S100A12/CX3CR1/CX3CL1/CCL2/CXCL10/CCL8/CXCL9/CCL18	24
BP	GO:0097529	myeloid leukocyte migration	24/340	241/18614	1.71E-11	4.64E-09	3.52E-09	CXCL12/TGFB2/PIK3CG/CD177/CCL5/STAP1/DPP4/RARRES2/IL1B/CCL23/S100A8/SAA1/IL6/FPR2/CXCL1/CXCL2/S100A12/CX3CR1/CX3CL1/CCL2/CXCL10/CCL8/CXCL9/CCL18	24
BP	GO:1990266	neutrophil migration	18/340	128/18614	2.01E-11	5.10E-09	3.87E-09	TGFB2/PIK3CG/CD177/CCL5/DPP4/IL1B/CCL23/S100A8/SAA1/CXCL1/CXCL2/S100A12/CX3CL1/CCL2/CXCL10/CCL8/CXCL9/CCL18	18
BP	GO:0071621	granulocyte chemotaxis	18/340	131/18614	2.99E-11	7.12E-09	5.41E-09	TGFB2/PIK3CG/CCL5/DPP4/RARRES2/IL1B/CCL23/S100A8/SAA1/CXCL1/CXCL2/S100A12/CX3CL1/CCL2/CXCL10/CCL8/CXCL9/CCL18	18
BP	GO:0050867	positive regulation of cell activation	30/340	394/18614	4.45E-11	9.97E-09	7.57E-09	HLA-DPA1/NFATC2/HLA-DOA/HLA-DOB/CD177/CCL5/EGR3/NFKBIZ/DDR2/STAP1/JUND/IL2RG/DPP4/ICOS/CAPN3/CD83/IL1B/FGR/NR4A3/VCAM1/CD27/RHOH/RASGRP1/CD5/CD2/CD3E/IL6/HLA-DQA1/CCL2/HLA-DQB1	30
BP	GO:00	granulocyte	19/340	156/18614	7.19E	1.52E	1.16E	TGFB2/PIK3CG/CD177/CCL5/DPP4/RARRES2/IL1B/CCL23/S100A8/SAA1/CXCL1/CXCL2/S100A12/CX3CL1/CCL2/CXCL10/CCL8/CXCL9/CCL18	19

	97530	migration			-11	-08	-08		
BP	GO:0002274	myeloid leukocyte activation	23/340	240/18614	9.92E-11	1.99E-08	1.51E-08	SLC18A2/PTGDS/PIK3CG/JUN/CNR1/CD177/CCL5/STAP1/JUND/HMOX1/FGR/NR4A3/VSIG4/RHOH/FCGR2B/RASGRP1/CD2/SLC11A1/IL6/S100A12/CX3CR1/CX3CL1/PLA2G2A	23
BP	GO:0001819	positive regulation of cytokine production	33/340	489/18614	1.12E-10	2.14E-08	1.62E-08	ZBTB20/HLA-DPA1/HGF/PTGS2/NAIP/TIGIT/EGR1/PIK3CG/BTN3A1/SERPINF2/ORM1/NLRC4/HMOX1/CD83/IL1B/FGR/NR4A3/SULF1/LY9/PYHIN1/RASGRP1/IRF1/CD2/CD3E/LUM/SLC11A1/ALOX15B/SAA1/IL6/CLEC4E/GBP5/CX3CL1/IDO1	33
BP	GO:0050727	regulation of inflammatory response	30/340	414/18614	1.49E-10	2.71E-08	2.06E-08	HGF/PTGS2/NAIP/PIK3CG/SIGLEC10/CNR1/CCL5/DUOXA1/NFKBIZ/STAP1/ACE/NLRC4/ZFP36/IER3/IL1B/FGR/GPR4/FCGR2B/S100A8/NR1D2/TNC/SAA1/IL6/FPR2/S100A12/CX3CL1/PLA2G2A/SOCS3/SELE/IDO1	30
BP	GO:0002443	leukocyte mediated immunity	29/340	401/18614	3.22E-10	5.57E-08	4.23E-08	CD1C/SLC18A2/HLA-DPA1/PTGDS/BTN3A3/PIK3CG/HLA-DOA/HLA-DOB/CD177/NFKBIZ/STAP1/ACE/SLAMF7/CD96/HMOX1/CD1E/IL1B/FGR/NR4A3/IL2RB/CD27/FCGR2B/RASGRP1/C1R/CD2/IL6/HLA-DQA1/CX3CR1/HLA-DQB1	29

BP	GO:0002696	positive regulation of leukocyte activation	28/340	377/18614	3.60E-10	5.96E-08	4.52E-08	HLA-DPA1/NFATC2/HLA-DOA/HLA-DOB/CD177/CCL5/EGR3/NFKBIZ/STAP1/JUND/IL2RG/DPP4/ICOS/CD83/IL1B/FGR/NR4A3/VCAM1/CD27/RHOH/RASGRP1/CD5/CD2/CD3E/IL6/HLA-DQA1/CCL2/HLA-DQB1	28
BP	GO:0030198	extracellular matrix organization	25/340	314/18614	7.81E-10	1.24E-07	9.42E-08	TGFB2/ADAMTS15/ECM2/MFAP4/SERPINF2/TNR/DDR2/HMCN1/BMP1/SH3PXD2B/CRISPLD2/TNFRSF11B/DPP4/SULF1/ABI3BP/COL8A1/ADAMTS9/MYH11/LUM/DPT/COL14A1/IL6/PTX3/HAS2/POSTN	25
BP	GO:0043062	extracellular structure organization	25/340	315/18614	8.34E-10	1.27E-07	9.66E-08	TGFB2/ADAMTS15/ECM2/MFAP4/SERPINF2/TNR/DDR2/HMCN1/BMP1/SH3PXD2B/CRISPLD2/TNFRSF11B/DPP4/SULF1/ABI3BP/COL8A1/ADAMTS9/MYH11/LUM/DPT/COL14A1/IL6/PTX3/HAS2/POSTN	25
BP	GO:0045229	external encapsulating structure organization	25/340	317/18614	9.52E-10	1.39E-07	1.06E-07	TGFB2/ADAMTS15/ECM2/MFAP4/SERPINF2/TNR/DDR2/HMCN1/BMP1/SH3PXD2B/CRISPLD2/TNFRSF11B/DPP4/SULF1/ABI3BP/COL8A1/ADAMTS9/MYH11/LUM/DPT/COL14A1/IL6/PTX3/HAS2/POSTN	25

BP	GO:1903131	mononuclear cell differentiation	30/340	474/18614	3.71E-09	5.23E-07	3.97E-07	EGR1/HLA-DOA/JUN/ITGA4/FASN/EGR3/NFKBIZ/JUNB/MS4A1/CD79A/IL2RG/HMGB3/CD83/IL1B/LY9/VCAM1/CD27/RHOH/FCGR2B/RASGRP1/THEMIS/FOS/IRF1/CD3G/INHBA/CD2/CD3E/IL6/CLEC4E/CD3D	30
BP	GO:0002548	monocyte chemotaxis	12/340	71/18614	5.54E-09	7.54E-07	5.72E-07	CXCL12/CCL5/CCL23/IL6/FPR2/S100A12/CX3CR1/CX3CL1/CCL2/CXCL10/CCL8/CCL18	12
BP	GO:0050870	positive regulation of T cell activation	21/340	249/18614	6.66E-09	8.75E-07	6.64E-07	HLA-DPA1/HLA-DOA/HLA-DOB/CCL5/EGR3/NFKBIZ/IL2RG/DPP4/ICOS/CD83/IL1B/VCAM1/CD27/RHOH/RASGRP1/CD5/CD3E/IL6/HLA-DQA1/CCL2/HLA-DQB1	21
BP	GO:0002253	activation of immune response	30/340	495/18614	1.00E-08	1.27E-06	9.66E-07	NFATC2/NAIP/BTN3A3/BTN3A1/SKAP1/NFKBIZ/STAP1/TRIL/MS4A1/CD79A/LAX1/TRAT1/NLRC4/IL1B/FGR/NR4A3/VSIG4/FPR1/PYHIN1/FCGR2B/THEMIS/C1R/TNIP3/IRF1/CD3G/CD3E/FPR2/CLEC4E/CD3D/HLA-DQB1	30
BP	GO:0070098	chemokine-mediated signalin	13/340	91/18614	1.07E-08	1.31E-06	9.96E-07	CXCL12/CCL5/SLIT3/CCL23/CXCL1/CXCL2/CX3CR1/CX3CL1/CCL2/CXCL10/CCL8/CXCL9/CCL18	13

		g pathway							
BP	GO:0019884	antigen processing and presentation of exogenous antigen	10/340	49/18614	1.58E-08	1.88E-06	1.43E-06	CD1C/CTSF/HLA-DPA1/HLA-DOA/HLA-DOB/CTSE/CD1E/FCGR2B/HLA-DQA1/HLA-DQB1	10
BP	GO:0030098	lymphocyte differentiation	27/340	422/18614	1.84E-08	2.13E-06	1.62E-06	EGR1/HLA-DOA/ITGA4/EGR3/NFKBIZ/JUNB/MS4A1/CD79A/IL2RG/HMGB3/CD83/IL1B/LY9/VCAM1/CD27/RHOH/FCGR2B/RASGRP1/THEMIS/IRF1/CD3G/INHBA/CD2/CD3E/IL6/CLEC4E/CD3D	27
BP	GO:0050729	positive regulation of inflammatory response	16/340	154/18614	2.36E-08	2.64E-06	2.01E-06	PTGS2/NAIP/PIK3CG/CNR1/NFKBIZ/STAP1/ACE/NLRC4/IL1B/GPR4/S100A8/IL6/S100A12/CX3CL1/PLA2G2A/IDO1	16
BP	GO:19	response to	13/340	99/18614	3.02E	3.20E	2.43E	CXCL12/CCL5/SLIT3/CCL23/CXCL1/CXCL2/CX3CR1/CX3CL1/CCL2/CXCL10/CCL8/CXCL9/CCL18	13

	90868	chemokine			-08	-06	-06		
BP	GO:1990869	cellular response to chemokine	13/340	99/18614	3.02E-08	3.20E-06	2.43E-06	CXCL12/CCL5/SLIT3/CCL23/CXCL1/CXCL2/CX3CR1/CX3CL1/CCL2/CXCL10/CCL8/CXCL9/CCL18	13
BP	GO:0071674	mononuclear cell migration	18/340	202/18614	3.50E-08	3.60E-06	2.73E-06	CXCL12/BMP5/PIK3CG/ITGA4/CCL5/RARRES2/CCL23/CH25H/SAA1/IL6/FPR2/S100A12/CX3CR1/CX3CL1/CCL2/CXCL10/CCL8/CCL18	18
BP	GO:0006959	humoral immune response	20/340	250/18614	3.72E-08	3.74E-06	2.84E-06	LYZ/PGC/MS4A1/CD83/RARRES2/IL1B/VSIG4/FCGR2B/C1R/SLC11A1/PI3/IL6/CXCL1/CXCL2/S100A12/CCL2/FGA/HLA-DQB1/CXCL10/CXCL9	20
BP	GO:1901214	regulation of neuron death	23/340	327/18614	3.89E-08	3.80E-06	2.89E-06	BTG2/HYOU1/GCLM/NAIP/TGFB2/VSTM2L/EGR1/JUN/CCL5/DDR2/HMOX1/SLC7A11/NQO1/NR4A3/FCGR2B/DDIT4/FOS/NTRK2/CX3CR1/CX3CL1/CCL2/NR4A2/CSF3	23
BP	GO:0002429	immune response-	22/340	302/18614	4.16E-08	3.96E-06	3.01E-06	NFATC2/BTN3A3/BTN3A1/SKAP1/NFKBIZ/STAP1/TRIL/MS4A1/CD79A/LAX1/TRAT1/FGR/NR4A3/FPR1/FCGR2B/THEMIS/TNIP3/CD3G/CD3E/FPR2/CD3D/HLA-DQB1	22

		activating cell surface receptor signaling pathway							
BP	GO:0002768	immune response-regulating cell surface receptor signaling pathway	23/340	329/18614	4.35E-08	4.04E-06	3.07E-06	NFATC2/BTN3A3/BTN3A1/SKAP1/NFKBIZ/STAP1/TRIL/MS4A1/CD79A/LAX1/TRAT1/FGR/NR4A3/FPR1/FCGR2B/THEMIS/TNIP3/CD3G/CD3E/FPR2/CLEC4E/CD3D/HLA-DQB1	23
BP	GO:0019886	antigen processing and	8/340	31/18614	6.24E-08	5.66E-06	4.30E-06	CTSF/HLA-DPA1/HLA-DOA/HLA-DOB/CTSE/FCGR2B/HLA-DQA1/HLA-DQB1	8

		presentation of exogenous peptide antigen via MHC class II							
BP	GO:0002757	immune response-activating signaling pathway	26/340	423/18614	7.61E-08	6.74E-06	5.12E-06	NFATC2/NAIP/BTN3A3/BTN3A1/SKAP1/NFKBIZ/STAP1/TRIL/MS4A1/CD79A/LAX1/TRAT1/NLRC4/FGR/NR4A3/FPR1/FCGR2B/THEMIS/TNIP3/IRF1/CD3G/CD3E/FPR2/CLEC4E/CD3D/HLA-DQB1	26
BP	GO:0045123	cellular extravasation	11/340	74/18614	9.62E-08	8.33E-06	6.33E-06	CXCL12/PIK3CG/ITGA4/CD177/LYVE1/VCAM1/PLVAP/CX3CR1/CX3CL1/CCL2/SELE	11
BP	GO:00	myeloid leukocyte	13/340	110/18614	1.08E	9.15E	6.95E	SLC18A2/PTGDS/PIK3CG/CD177/STAP1/ACE/HMOX1/FGR/NR4A3/FCGR2B/RASGRP1/IL6/CX3CR1	13

	02444	mediated immunity			-07	-06	-06		
BP	GO:0019221	cytokine-mediated signaling pathway	28/340	492/18614	1.19E-07	9.84E-06	7.47E-06	CXCL12/NAIP/IL17RB/STAT4/EGR1/DUOX1/CCL5/NFKBIZ/SLIT3/STAP1/IL2RG/IL1B/CCL23/IL2RB/IL1R2/IRF1/PLVAP/IL6/CXCL1/CXCL2/CX3CR1/CX3CL1/CCL2/CSF3/CXCL10/CCL8/CXCL9/CCL18	28
BP	GO:0002526	acute inflammatory response	13/340	114/18614	1.65E-07	1.33E-05	1.01E-05	CD163/PTGS2/PIK3CG/CNR1/SERPINF2/SERPINA3/ORM1/IL1B/VCAM1/FCGR2B/S100A8/SAA1/IL6	13
BP	GO:0051251	positive regulation of lymphocyte activation	22/340	327/18614	1.68E-07	1.33E-05	1.01E-05	HLA-DPA1/NFATC2/HLA-DOA/HLA-DOB/CCL5/EGR3/NFKBIZ/IL2RG/DPP4/ICOS/CD83/IL1B/VCAM1/CD27/RHOH/RASGRP1/CD5/CD3E/IL6/HLA-DQA1/CCL2/HLA-DQB1	22

BP	GO:0031349	positive regulation of defense response	26/340	441/18614	1.72E-07	1.33E-05	1.01E-05	PTGS2/NAIP/PIK3CG/CNR1/CCL5/NFKBIZ/STAP1/TRIL/ACE/PGC/NLRC4/IL1B/GPR4/PYHIN1/RASGRP1/S100A8/TNIP3/IRF1/IL6/FPR2/CLEC4E/S100A12/GBP5/CX3CL1/PLA2G2A/IDO1	26
BP	GO:0002495	antigen processing and presentation of peptide antigen via MHC class II	8/340	35/18614	1.75E-07	1.33E-05	1.01E-05	CTSF/HLA-DPA1/HLA-DOA/HLA-DOB/CTSE/FCGR2B/HLA-DQA1/HLA-DQB1	8
BP	GO:0007584	response to nutrient	15/340	156/18614	1.82E-07	1.34E-05	1.02E-05	GCLM/CBS/PTGS2/CNR1/NFKBIZ/TNFRSF11B/ALPL/HMOX1/NQO1/AKR1C3/VCAM1/LIPG/CXCL10/SLC6A4/POSTN	15
BP	GO:0006953	acute-phase response	9/340	48/18614	1.82E-07	1.34E-05	1.02E-05	CD163/PTGS2/CNR1/SERPINF2/SERPINA3/ORM1/IL1B/SAA1/IL6	9

BP	GO:0032102	negative regulation of response to external stimulus	26/340	446/18614	2.15E-07	1.54E-05	1.17E-05	HGF/ROBO2/SEMA3D/SIGLEC10/MMRN1/SERPINF2/SEMA6D/TNR/STAP1/CD96/DPP4/ZFP36/IER3/FGR/VSIG4/FCGR2B/NR1D2/TRIB1/SEMA6A/SAA1/FPR2/CX3CL1/SEMA3G/SOCS3/CCL2/FGA	26
BP	GO:0048247	lymphocyte chemotaxis	10/340	64/18614	2.29E-07	1.61E-05	1.23E-05	PIK3CG/CCL5/CCL23/CH25H/SAA1/CX3CL1/CCL2/CXCL10/CCL8/CCL18	10
BP	GO:0002764	immune response-regulating signaling pathway	26/340	450/18614	2.55E-07	1.77E-05	1.34E-05	NFATC2/NAIP/BTN3A3/BTN3A1/SKAP1/NFKBIZ/STAP1/TRIL/MS4A1/CD79A/LAX1/TRAT1/NLRC4/FGR/NR4A3/FPR1/FCGR2B/THEMIS/TNIP3/IRF1/CD3G/CD3E/FPR2/CLEC4E/CD3D/HLA-DQB1	26

BP	GO:0043270	positive regulation of monoatomic ion transport	18/340	231/18614	2.68E-07	1.82E-05	1.38E-05	P2RY1/TRPC6/RYR2/CXCL12/FXYD1/ANK2/APLNR/CNTN1/TRPC1/CCL5/MS4A1/CAPN3/CFTR/CX3CL1/CCL2/CXCL10/SLC6A4/CXCL9	18
BP	GO:0002504	antigen processing and presentation of peptide or polysaccharide antigen via MHC class II	8/340	37/18614	2.78E-07	1.86E-05	1.41E-05	CTSF/HLA-DPA1/HLA-DOA/HLA-DOB/CTSE/FCGR2B/HLA-DQA1/HLA-DQB1	8
BP	GO:0070997	neuron death	23/340	373/18614	4.14E-07	2.72E-05	2.07E-05	BTG2/HYOU1/GCLM/NAIP/TGFB2/VSTM2L/EGR1/JUN/CCL5/DDR2/HMOX1/SLC7A11/NQO1/NR4A3/FCGR2B/DDIT4/FOS/NTRK2/CX3CR1/CX3CL1/CCL2/NR4A2/CSF3	23

BP	GO:0043410	positive regulation of MAPK cascade	27/340	495/18614	4.65E-07	3.01E-05	2.28E-05	P2RY1/HGF/NAIP/TGFB2/PIK3CG/JUN/CCL5/SERPINF2/DDR2/FGD2/IL1B/CCL23/CD27/FCGR2B/RASGRP1/INHBA/MARCO/IL6/NTRK2/FPR2/S100A12/CX3CL1/PLA2G2A/CCL2/FGA/CCL8/CCL18	27
BP	GO:0045576	mast cell activation	10/340	69/18614	4.74E-07	3.01E-05	2.28E-05	SLC18A2/PTGDS/PIK3CG/CNR1/HMOX1/FGR/NR4A3/RHOH/RASGRP1/S100A12	10
BP	GO:0070661	leukocyte proliferation	22/340	348/18614	4.88E-07	3.05E-05	2.32E-05	HLA-DPA1/NFATC2/PIK3CG/BTN3A1/CCL5/NFKBIZ/JUNB/ACE/MS4A1/CD79A/IL1B/VSIG4/VCAM1/FCGR2B/RASGRP1/IRF1/CD3E/SLC11A1/IL6/CX3CL1/PLA2G2A/IDO1	22
BP	GO:0002478	antigen processing and presentation of exogenous peptide antigen	8/340	40/18614	5.27E-07	3.24E-05	2.46E-05	CTSF/HLA-DPA1/HLA-DOA/HLA-DOB/CTSE/FCGR2B/HLA-DQA1/HLA-DQB1	8

BP	GO:0048002	antigen processing and presentation of peptide antigen	10/340	71/18614	6.23E-07	3.77E-05	2.86E-05	CTSF/HLA-DPA1/HLA-DOA/HLA-DOB/ACE/CTSE/FCGR2B/SLC11A1/HLA-DQA1/HLA-DQB1	10
BP	GO:0051928	positive regulation of calcium ion transport	13/340	129/18614	7.03E-07	4.18E-05	3.18E-05	TRPC6/RYR2/CXCL12/ANK2/APLNR/TRPC1/CCL5/MS4A1/CAPN3/CX3CL1/CCL2/CXCL10/CXCL9	13
BP	GO:0030217	T cell differentiation	20/340	300/18614	7.13E-07	4.18E-05	3.18E-05	EGR1/HLA-DOA/EGR3/NFKBIZ/JUNB/IL2RG/CD83/IL1B/LY9/CD27/RHOH/RASGRP1/THEMIS/IRF1/CD3G/CD2/CD3E/IL6/CLEC4E/CD3D	20
BP	GO:0050851	antigen receptor-mediated signaling	16/340	201/18614	9.36E-07	5.40E-05	4.10E-05	NFATC2/BTN3A3/BTN3A1/SKAP1/NFKBIZ/STAP1/MS4A1/CD79A/LAX1/TRAT1/FCGR2B/THEMIS/CD3G/CD3E/CD3D/HLA-DQB1	16

		pathway							
BP	GO:0002685	regulation of leukocyte migration	17/340	227/18614	1.00E-06	5.71E-05	4.34E-05	CXCL12/BMP5/ITGA4/CCL5/STAP1/DPP4/HMOX1/RARRES2/LYVE1/PLVAP/IL6/FPR2/CX3CR1/CX3CL1/CCL2/CXCL10/SELE	17
BP	GO:0019882	antigen processing and presentation	12/340	114/18614	1.18E-06	6.63E-05	5.04E-05	CD1C/CTSF/HLA-DPA1/HLA-DOA/HLA-DOB/ACE/CTSE/CD1E/FCGR2B/SLC11A1/HLA-DQA1/HLA-DQB1	12
BP	GO:0032943	mononuclear cell proliferation	20/340	313/18614	1.38E-06	7.63E-05	5.79E-05	HLA-DPA1/NFATC2/PIK3CG/BTN3A1/CCL5/NFKBIZ/ACE/MS4A1/CD79A/IL1B/VSIG4/VCAM1/FCGR2B/RASGRP1/IRF1/CD3E/SLC11A1/IL6/PLA2G2A/IDO1	20
BP	GO:0042742	defense response to bacterium	20/340	314/18614	1.45E-06	7.90E-05	6.00E-05	NAIP/LYZ/NFKBIZ/PGC/NLRC4/RARRES2/IL1B/FGR/STAB1/LCN2/S100A8/SLC11A1/PI3/IL6/FPR2/CLEC4E/S100A12/GBP4/PLA2G2A/FGA	20

BP	GO:0071219	cellular response to molecule of bacterial origin	17/340	237/18614	1.82E-06	9.77E-05	7.42E-05	CCL5/NFKBIZ/STAP1/ZFP36/IL1B/FCGR2B/TRIB1/TNIP3/IL6/CXCL1/CXCL2/CX3CR1/CX3CL1/CCL2/CSF3/CXCL10/CXCL9	17
BP	GO:0051924	regulation of calcium ion transport	18/340	266/18614	2.09E-06	0.000110404	8.39E-05	TRPC6/RYR2/CXCL12/PTGS2/ANK2/GEM/PIK3CG/APLNR/TRPC1/CCL5/CASQ2/ACE/MS4A1/CAPN3/CX3CL1/CCL2/CXCL10/CXCL9	18
BP	GO:0072676	lymphocyte migration	12/340	122/18614	2.44E-06	0.000121275	9.68E-05	CXCL12/PIK3CG/ITGA4/CCL5/CCL23/CH25H/SAA1/CX3CL1/CCL2/CXCL10/CCL8/CCL18	12
BP	GO:0002449	lymphocyte mediated immunity	19/340	300/18614	2.89E-06	0.000141491	0.000111132	CD1C/HLA-DPA1/BTN3A3/HLA-DOA/HLA-DOB/NFKBIZ/SLAMF7/CD96/CD1E/IL1B/IL2RB/CD27/FCGR2B/RASGRP1/C1R/CD2/IL6/HLA-DQA1/HLA-DQB1	19

BP	GO:0070374	positive regulation of ERK1 and ERK2 cascade	16/340	222/18614	3.45E-06	0.0001751	0.0001329	P2RY1/JUN/CCL5/SERPINF2/DDR2/CCL23/RASGRP1/INHBA/MARCO/FPR2/CX3CL1/PLA2G2A/CCL2/FGA/CCL8/CCL18	16
BP	GO:0002683	negative regulation of immune system process	25/340	487/18614	3.70E-06	0.0001855	0.0001409	CXCL12/BMP5/TGFB2/TIGIT/HLA-DOA/HLA-DOB/CNR1/STAP1/LAX1/HMGB3/CD96/TNFRSF11B/DPP4/HMOX1/FGR/VSIG4/FCGR2B/TRIB1/IRF1/INHBA/CX3CR1/CX3CL1/PLA2G2A/CCL2/IDO1	25
BP	GO:0046651	lymphocyte proliferation	19/340	306/18614	3.86E-06	0.000189	0.0001435	HLA-DPA1/NFATC2/PIK3CG/BTN3A1/CCL5/NFKBIZ/MS4A1/CD79A/IL1B/VSIG4/VCAM1/FCGR2B/RASGRP1/IRF1/CD3E/SLC11A1/IL6/PLA2G2A/IDO1	19
BP	GO:0071222	cellular response to lipopoly	16/340	224/18614	3.87E-06	0.000189	0.0001435	CCL5/NFKBIZ/STAP1/ZFP36/IL1B/TRIB1/TNIP3/IL6/CXCL1/CXCL2/CX3CR1/CX3CL1/CCL2/CSF3/CXCL10/CXCL9	16

BP	GO:0070372	saccharide regulation of ERK1 and ERK2 cascade	19/340	315/18614	5.87E-06	0.000028333	0.000021151	P2RY1/JUN/CCL5/SERPINF2/DDR2/IL1B/CCL23/RASGRP1/ATF3/INHBA/SEMA6A/MARCO/FPR2/CX3CL1/PLA2G2A/CCL2/FGA/CCL8/CCL18	19
BP	GO:0071347	cellular response to interleukin-1	11/340	111/18614	5.97E-06	0.000028446	0.000021161	EGR1/CCL5/IL1B/CCL23/IL1R2/IL6/CX3CL1/CCL2/HAS2/CCL8/CCL18	11
BP	GO:2001234	negative regulation of apoptotic signaling pathway	16/340	233/18614	6.40E-06	0.000030012	0.000022287	RTKN2/CXCL12/ENO1/HYOU1/BMP5/GCLM/HGF/PTGS2/HMOX1/IER3/IL1B/CTH/CX3CR1/CX3CL1/NR4A2/FGA	16

BP	GO:0050766	positive regulation of phagocytosis	9/340	73/18614	7.07E-06	0.00003251	0.00002469	STAP1/IL2RG/IL1B/IL2RB/FCGR2B/SLC11A1/FPR2/PTX3/CCL2	9
BP	GO:0009069	serine family amino acid metabolic process	7/340	40/18614	7.08E-06	0.00003251	0.00002469	PSAT1/SHMT2/GCLM/CBS/PHGDH/SLC7A11/CTH	7
BP	GO:0002399	MHC class II protein complex assembly	5/340	16/18614	7.31E-06	0.00003277	0.00002488	HLA-DPA1/HLA-DOA/HLA-DOB/HLA-DQA1/HLA-DQB1	5
BP	GO:0002503	peptide antigen assembly with MHC class II	5/340	16/18614	7.31E-06	0.00003277	0.00002488	HLA-DPA1/HLA-DOA/HLA-DOB/HLA-DQA1/HLA-DQB1	5

BP	GO:2001233	protein complex regulation of apoptotic signaling pathway	21/340	381/18614	7.70E-06	0.000034	0.00002582	RTKN2/CXCL12/ENO1/HYOU1/BMP5/GCLM/HGF/PTGS2/SRPX/HMOX1/IER3/IL1B/S100A8/CTH/ATF3/INHBA/TNFSF10/CX3CR1/CX3CL1/NR4A2/FGA	21
BP	GO:0071216	cellular response to biotic stimulus	17/340	264/18614	7.76E-06	0.000034	0.00002582	CCL5/NFKBIZ/STAP1/ZFP36/IL1B/FCGR2B/TRIB1/TNIP3/IL6/CXCL1/CXCL2/CX3CR1/CX3CL1/CCL2/CSF3/CXCL10/CXCL9	17
BP	GO:0001666	response to hypoxia	18/340	293/18614	8.05E-06	0.00003485	0.00002646	RYR2/CXCL12/ENO1/HYOU1/CBS/PTGS2/TGFB2/EGR1/DDR2/JUND/ACE/DPP4/HMOX1/VCAM1/DDIT4/FOS/NR4A2/SLC6A4	18
BP	GO:1902074	response to salt	21/340	383/18614	8.34E-06	0.00003573	0.00002713	P2RY1/RYR2/SCNN1G/GCLM/PTGS2/EGR1/JUN/TRPC1/CNR1/FOSB/JUNB/CASQ2/JUND/ALPL/CAPN3/IL1B/AKR1C3/SLC1A3/FOS/ASPN/FGA	21

BP	GO:0033631	cell-cell adhesion mediated by integrin	5/340	17/18614	1.02E-05	0.00004319	0.00003279	ITGA4/CCL5/SKAP1/DPP4/CD3E	5
BP	GO:0046394	carboxylic acid biosynthetic process	19/340	328/18614	1.04E-05	0.00004375	0.00003322	PSAT1/SHMT2/TRIB3/PYCR1/CBS/PTGS2/NAIP/PHGDH/PTGDS/HPGDS/FASN/NLRC4/IL1B/AKR1C3/BCAT1/SLC1A3/CTH/ALOX15B/IDO1	19
BP	GO:0070555	response to interleukin-1	12/340	141/18614	1.10E-05	0.00004556	0.00003459	EGR1/CCL5/IL1B/CCL23/IL1R2/IL6/CX3CL1/CCL2/HAS2/CCL8/SELE/CCL18	12
BP	GO:0048245	eosinophil chemotaxis	6/340	29/18614	1.18E-05	0.00004815	0.00003656	CCL5/CCL23/CX3CL1/CCL2/CCL8/CCL18	6
BP	GO:0016053	organic acid biosynthetic	19/340	331/18614	1.19E-05	0.00004815	0.00003656	PSAT1/SHMT2/TRIB3/PYCR1/CBS/PTGS2/NAIP/PHGDH/PTGDS/HPGDS/FASN/NLRC4/IL1B/AKR1C3/BCAT1/SLC1A3/CTH/ALOX15B/IDO1	19

		process							
BP	GO:0014074	response to purine-containing compound	12/340	143/18614	1.27E-05	0.0005092	0.0003867	P2RY1/RYR2/PTGS2/PIK3CG/DUOX1/FOSB/CASQ2/IL1B/AQP9/FOS/CFTR/SLC6A4	12
BP	GO:1901215	negative regulation of neuron death	15/340	219/18614	1.28E-05	0.0005096	0.0003869	BTG2/HYOU1/GCLM/NAIP/VSTM2L/JUN/HMOX1/SLC7A11/NR4A3/NTRK2/CX3CR1/CX3CL1/CCL2/NR4A2/CSF3	15
BP	GO:0043434	response to peptide hormone	22/340	427/18614	1.35E-05	0.000531	0.0004032	BTG2/TRIB3/SCNN1G/CXCL12/PTGS2/STAT4/EGR1/TIMP4/EGR2/DDR2/JUND/SLC39A14/ALPL/RARRES2/IL1B/NR4A3/FOS/INHBA/TNFSF10/NR4A1/SOCS3/NR4A2	22
BP	GO:0036293	response to decreased oxygen levels	18/340	306/18614	1.45E-05	0.0005645	0.0004287	RYR2/CXCL12/ENO1/HYOU1/CBS/PTGS2/TGFB2/EGR1/DDR2/JUND/ACE/DPP4/HMOX1/VCAM1/DDIT4/FOS/NR4A2/SLC6A4	18

BP	GO:0016485	protein processing	16/340	250/18614	1.55E-05	0.0005963	0.0004528	CORIN/RPS6KA2/ENO1/PRSS12/SERPINF2/BMP1/CPA3/ACE/CTSE/NLRC4/DPP4/IL1R2/PCSK2/CPE/C1R/FGA	16
BP	GO:0050764	regulation of phagocytosis	10/340	101/18614	1.60E-05	0.0006087	0.0004622	STAP1/IL2RG/IL1B/FGR/IL2RB/FCGR2B/SLC11A1/FPR2/PTX3/CCL2	10
BP	GO:0043299	leukocyte degranulation	9/340	81/18614	1.67E-05	0.0006271	0.0004761	SLC18A2/PTGDS/PIK3CG/CD177/HMOX1/FGR/NR4A3/FCGR2B/RASGRP1	9
BP	GO:0002687	positive regulation of leukocyte migration	12/340	147/18614	1.68E-05	0.0006271	0.0004761	CXCL12/ITGA4/CCL5/RARRES2/LYVE1/PLVAP/IL6/FPR2/CX3CR1/CX3CL1/CXCL10/SELE	12
BP	GO:0070371	ERK1 and ERK2 cascade	19/340	341/18614	1.80E-05	0.0006613	0.0005021	P2RY1/JUN/CCL5/SERPINF2/DDR2/IL1B/CCL23/RASGRP1/ATF3/INHBA/SEMA6A/MARCO/FPR2/CX3CL1/PLA2G2A/CCL2/FGA/CCL8/CCL18	19

BP	GO:0002460	adaptive immune response based on somatic recombination of immune receptors built from immunoglobulin superfamily domains	18/340	311/18614	1.80E-05	0.00006613	0.00005021	CD1C/HLA-DPA1/BTN3A3/HLA-DOA/HLA-DOB/NFKBIZ/JUNB/CD1E/IL1B/LY9/IL2RB/CD27/FCGR2B/C1R/SLC11A1/IL6/HLA-DQA1/HLA-DQB1	18

BP	GO:0051279	regulation of release of sequestered calcium ion into cytosol	9/340	82/18614	1.85E-05	0.0006708	0.0005094	RYR2/ANK2/APLNR/TRPC1/CASQ2/CAPN3/CX3CL1/CXCL10/CXCL9	9
BP	GO:0019730	antimicrobial humoral response	11/340	126/18614	2.01E-05	0.0007231	0.0005491	LYZ/PGC/RARRES2/SLC11A1/PI3/CXCL1/CXCL2/S100A12/FGA/CXCL10/CXCL9	11
BP	GO:0034767	positive regulation of monoatomic ion transmembrane transport	13/340	175/18614	2.07E-05	0.0007364	0.0005591	TRPC6/RYR2/FXYD1/ANK2/APLNR/TRPC1/MS4A1/CAPN3/CFTR/CX3CL1/CCL2/CXCL10/CXCL9	13

BP	GO:0050919	negative chemotaxis	7/340	47/18614	2.14E-05	0.00007494	0.0000569	ROBO2/SEMA3D/SEMA6D/SLIT3/DPP4/SEMA6A/SEMA3G	7
BP	GO:0009410	response to xenobiotic stimulus	22/340	440/18614	2.14E-05	0.00007494	0.0000569	PLIN2/NFATC2/GCLM/PTGS2/TGFB2/TIMP4/JUN/FOSB/NFKBIZ/ACE/TNFRSF11B/HMOX1/NQO1/IL1B/CD69/SULT1B1/SLC1A3/FOS/INHBA/AOX1/S100A12/SLC6A4	22
BP	GO:0050920	regulation of chemotaxis	15/340	230/18614	2.29E-05	0.00007931	0.00006022	CXCL12/ROBO2/SEMA3D/CCL5/SEMA6D/STAP1/DPP4/RARRES2/SEMA6A/IL6/FPR2/CX3CR1/SEMA3G/CCL2/CXCL10	15
BP	GO:0002396	MHC protein complex assembly	5/340	20/18614	2.44E-05	0.00008238	0.00006255	HLA-DPA1/HLA-DOA/HLA-DOB/HLA-DQA1/HLA-DQB1	5
BP	GO:0002501	peptide antigen assembly with MHC protein	5/340	20/18614	2.44E-05	0.00008238	0.00006255	HLA-DPA1/HLA-DOA/HLA-DOB/HLA-DQA1/HLA-DQB1	5

BP	GO:0002544	chronic inflammatory response	5/340	20/18614	2.44E-05	0.00008238	0.00006255	CCL5/NFKBIZ/VCAM1/S100A8/IDO1	5
BP	GO:0072677	eosinophil migration	6/340	33/18614	2.60E-05	0.00008676	0.00006587	CCL5/CCL23/CX3CL1/CCL2/CCL8/CCL18	6
BP	GO:0043524	negative regulation of neuron apoptotic process	12/340	155/18614	2.85E-05	0.00009435	0.00007164	BTG2/HYOU1/GCLM/NAIP/VSTM2L/JUN/HMOX1/NTRK2/CX3CR1/CX3CL1/CCL2/NR4A2	12
BP	GO:0032612	interleukin-1 production	11/340	131/18614	2.90E-05	0.00009435	0.00007164	NAIP/EGR1/ORM1/NLRC4/IL1R2/PYHIN1/SAA1/IL6/GBP5/CX3CR1/CX3CL1	11

BP	GO:0032652	regulation of interleukin-1 production	11/340	131/18614	2.90E-05	0.0009435	0.0007164	NAIP/EGR1/ORM1/NLRC4/IL1R2/PYHIN1/SAA1/IL6/GBP5/CX3CR1/CX3CL1	11
BP	GO:0033627	cell adhesion mediated by integrin	9/340	87/18614	2.99E-05	0.0009646	0.0007324	TGFB2/ITGA10/PIK3CG/MMRN1/ITGA4/CCL5/SKAP1/DPP4/CD3E	9
BP	GO:0009070	serine family amino acid biosynthetic process	5/340	21/18614	3.16E-05	0.001003	0.0007615	PSAT1/SHMT2/CBS/PHGDH/CTH	5
BP	GO:0090026	positive regulation of monocyte	5/340	21/18614	3.16E-05	0.001003	0.0007615	CXCL12/CCL5/FPR2/CX3CR1/CXCL10	5

BP	GO:0035914	chemotaxis skeletal muscle cell differentiation	8/340	68/18614	3.25E-05	0.00101187	0.00077735	BTG2/EGR1/EGR2/HLF/NR1D2/ATF3/FOS/NR4A1	8
BP	GO:0033632	regulation of cell-cell adhesion mediated by integrin	4/340	11/18614	3.26E-05	0.00101187	0.00077735	CCL5/SKAP1/DPP4/CD3E	4
BP	GO:0002366	leukocyte activation involved in immune response	17/340	296/18614	3.40E-05	0.0010521	0.00079789	CD1C/SLC18A2/PTGDS/PIK3CG/CD177/NFKBIZ/JUNB/HMOX1/FGR/NR4A3/LY9/FCGR2B/RASGRP1/SLC11A1/IL6/CLEC4E/CX3CR1	17

BP	GO:0055074	calcium ion homeostasis	18/340	327/18614	3.51E-05	0.0010776	0.0008182	P2RY1/PLCB4/TRPC6/RYR2/CXCL12/ANK2/APLNR/TRPC1/CNR1/CCL5/CASQ2/ALPL/CAPN3/CCL23/CX3CL1/CXCL10/CCL8/CXCL9	18
BP	GO:0018958	phenol-containing compound metabolic process	10/340	111/18614	3.65E-05	0.0011067	0.0008403	FAH/TGFB2/DUOX1/TRPC1/HDC/DUOXA1/SLC7A11/SULT1B1/MAOA/NR4A2	10
BP	GO:0010959	regulation of metal ion transport	21/340	423/18614	3.66E-05	0.0011067	0.0008403	TRPC6/RYR2/CXCL12/FXYD1/KCNAB1/PTGS2/ANK2/GEM/PIK3CG/APLNR/CNTN1/TRPC1/CCL5/CASQ2/ACE/MS4A1/CAPN3/CX3CL1/CCL2/CXCL10/CXCL9	21
BP	GO:0042098	T cell proliferation	14/340	213/18614	3.93E-05	0.001172	0.0008899	HLA-DPA1/PIK3CG/BTN3A1/CCL5/IL1B/VSIG4/VCAM1/RASGRP1/IRF1/CD3E/SLC11A1/IL6/PLA2G2A/IDO1	14
BP	GO:00	hormone metabo	15/340	241/18614	3.94E	0.001	0.000	CORIN/AKR1C2/BMP5/EGR1/SDR16C5/DUOX1/DHCR7/DUOXA1/CPA3/ACE/DPP4/AKR1C3/PCSK2/SULT1B1/CPE	15

	42445	lic process			-05	172	8899		
BP	GO:0002263	cell activation involved in immune response	17/340	300/18614	4.02E-05	0.0011179	0.0008952	CD1C/SLC18A2/PTGDS/PIK3CG/CD177/NFKBIZ/JUNB/HMOX1/FGR/NR4A3/LY9/FCGR2B/RASGRP1/SLC11A1/IL6/CLEC4E/CX3CR1	17
BP	GO:0050922	negative regulation of chemotaxis	8/340	70/18614	4.02E-05	0.0011179	0.0008952	ROBO2/SEMA3D/SEMA6D/STAP1/DPP4/SEMA6A/SEMA3G/CCL2	8
BP	GO:0009612	response to mechanical stimulus	14/340	215/18614	4.35E-05	0.0012657	0.0009961	P2RY1/BTG2/RYR2/CXCL12/PTGS2/JUN/FOSB/DDR2/JUND/IL1B/SLC1A3/FOS/IRF1/CXCL10	14

BP	GO:0070482	response to oxygen levels	18/340	333/18614	4.44E-05	0.0012832	0.0009743	RYR2/CXCL12/ENO1/HYOU1/CBS/PTGS2/TGFB2/EGR1/DDR2/JUND/ACE/DPP4/HMOX1/VCAM1/DDIT4/FOS/NR4A2/SLC6A4	18
BP	GO:0006874	intracellular calcium ion homeostasis	17/340	303/18614	4.55E-05	0.0013045	0.0009905	P2RY1/PLCB4/TRPC6/RYR2/CXCL12/ANK2/APLNR/TRPC1/CNR1/CCL5/CASQ2/CAPN3/CCL23/CX3CL1/CXCL10/CCL8/CXCL9	17
BP	GO:0010038	response to metal ion	19/340	367/18614	4.91E-05	0.0013973	0.0010609	RYR2/PTGS2/JUN/TRPC1/FOSB/JUNB/CASQ2/JUND/TNFRSF11B/HMOX1/CAPN3/NQO1/AKR1C3/VCAM1/S100A8/FOS/FGA/MT1X/MT1M	19
BP	GO:0034341	response to type II interferon	11/340	139/18614	5.01E-05	0.0014147	0.0010742	HLA-DPA1/CCL5/CCL23/IRF1/SLC11A1/GBP5/CX3CL1/GBP4/CCL2/CCL8/CCL18	11
BP	GO:1904427	positive regulation of calcium ion transmembran	9/340	93/18614	5.09E-05	0.0014249	0.0010819	RYR2/ANK2/APLNR/TRPC1/MS4A1/CAPN3/CX3CL1/CXCL10/CXCL9	9

		e transport							
BP	GO:0016486	peptide hormone processing	6/340	37/18614	5.12E-05	0.000142249	0.000108819	CORIN/CPA3/ACE/DPP4/PCSK2/CPE	6
BP	GO:0043523	regulation of neuron apoptotic process	14/340	219/18614	5.32E-05	0.000146007	0.000110091	BTG2/HYOU1/GCLM/NAIP/TGFB2/VSTM2L/JUN/HMOX1/NQO1/NTRK2/CX3CR1/CX3CL1/CCL2/NR4A2	14
BP	GO:0071346	cellular response to type II interferon	10/340	116/18614	5.33E-05	0.000146007	0.000110091	HLA-DPA1/CCL5/CCL23/IRF1/GBP5/CX3CL1/GBP4/CCL2/CCL8/CCL18	10
BP	GO:0043303	mast cell degranulation	7/340	54/18614	5.40E-05	0.000147008	0.000111168	SLC18A2/PTGDS/PIK3CG/HMOX1/FGR/NR4A3/RASGRP1	7

BP	GO:0031960	response to corticosteroid	12/340	166/18614	5.59E-05	0.001512	0.001481	SCNN1G/PTGS2/PTGDS/FOSB/SLIT3/ACE/ALPL/ZFP36/AKR1C3/DDIT4/FOS/IL6	12
BP	GO:0043368	positive T cell selection	6/340	38/18614	5.99E-05	0.0015841	0.0012028	LY9/THEMIS/CD3G/CD3E/IL6/CD3D	6
BP	GO:0048846	axon extension involved in axon guidance	6/340	38/18614	5.99E-05	0.0015841	0.0012028	CXCL12/SEMA3D/SEMA6D/SLIT3/SEMA6A/SEMA3G	6
BP	GO:1902284	neuron projection extension involved in neuron projection guidance	6/340	38/18614	5.99E-05	0.0015841	0.0012028	CXCL12/SEMA3D/SEMA6D/SLIT3/SEMA6A/SEMA3G	6

		guidance							
BP	GO:0032732	positive regulation of interleukin-1 production	8/340	74/18614	6.03E-05	0.0015841	0.0012028	NAIP/EGR1/ORM1/NLRC4/PYHIN1/SAA1/IL6/GBP5	8
BP	GO:0061900	glial cell activation	7/340	55/18614	6.10E-05	0.0015901	0.0012087	JUN/STAP1/IL1B/IL6/CX3CR1/CX3CL1/MT1X	7
BP	GO:0097305	response to alcohol	15/340	252/18614	6.55E-05	0.00169678	0.00128891	SCNN1G/AKR1C2/CNR1/FOSB/SLIT3/ACE/NQO1/AKR1C3/VCAM1/CD27/S100A8/FOS/INHBA/CFTR/CSF3	15
BP	GO:0042698	ovulation cycle	8/340	75/18614	6.64E-05	0.00170222	0.00129225	ROBO2/TGFB2/EGR1/TIMP4/SLIT3/INHBA/PTX3/HAS2	8
BP	GO:0007568	aging	12/340	169/18614	6.66E-05	0.00170222	0.00129225	P2RY1/GCLM/PTGS2/CNR1/JUND/ACE/ALPL/NQO1/VCAM1/FOS/INHBA/CX3CL1	12

BP	GO:0002279	mast cell activation involved in immune response	7/340	56/18614	6.86E-05	0.0017097	0.0012982	SLC18A2/PTGDS/PIK3CG/HMOX1/FGR/NR4A3/RASGRP1	7
BP	GO:0006534	cysteine metabolic process	4/340	13/18614	6.86E-05	0.0017097	0.0012982	GCLM/CBS/SLC7A11/CTH	4
BP	GO:0006563	L-serine metabolic process	4/340	13/18614	6.86E-05	0.0017097	0.0012982	PSAT1/SHMT2/CBS/PHGDH	4
BP	GO:0070486	leukocyte aggregation	4/340	13/18614	6.86E-05	0.0017097	0.0012982	IL1B/NR4A3/S100A8/HAS2	4

BP	GO:0150076	neuroinflammatory response	8/340	76/18614	7.31E-05	0.0018088	0.0013728	PTGS2/JUN/STAP1/IL1B/IL6/CX3CR1/CX3CL1/MT1X	8
BP	GO:0002275	myeloid cell activation involved in immune response	9/340	98/18614	7.69E-05	0.00188803	0.00142477	SLC18A2/PTGDS/PIK3CG/CD177/HMOX1/FGR/NR4A3/RASGRP1/CX3CR1	9
BP	GO:0002448	mast cell mediated immunity	7/340	57/18614	7.70E-05	0.00188803	0.00142477	SLC18A2/PTGDS/PIK3CG/HMOX1/FGR/NR4A3/RASGRP1	7
BP	GO:0097191	extrinsic apoptotic signalin	14/340	227/18614	7.83E-05	0.00190904	0.0014443	BMP5/GCLM/HGF/TGFB2/NFKBIZ/SRPX/HMOX1/IL1B/CD27/ATF3/INHBA/TNFSF10/CX3CL1/FGA	14

		g pathway							
BP	GO:0140448	signaling receptor ligand precursor processing	6/340	40/18614	8.08E-05	0.0019481	0.0014792	CORIN/CPA3/ACE/DPP4/PCSK2/CPE	6
BP	GO:0048661	positive regulation of smooth muscle cell proliferation	9/340	99/18614	8.32E-05	0.0019949	0.0015147	PTGS2/JUN/CCL5/SERPINF2/DDR2/HMOX1/NR4A3/IL6/CX3CL1	9
BP	GO:1902105	regulation of leukocyte differentiation	17/340	319/18614	8.59E-05	0.002046	0.0015535	HLA-DOA/EGR3/NFKBIZ/JUNB/IL2RG/HMGB3/TNFRSF11B/CD83/CD27/RHOH/FCGR2B/RASGRP1/TRIB1/FOS/IRF1/INHBA/CD2	17

BP	GO:0008652	amino acid biosynthetic process	8/340	78/18614	8.80E-05	0.00208835	0.00158582	PSAT1/SHMT2/PYCR1/CBS/PHGDH/BCAT1/SLC1A3/CTH	8
BP	GO:0002697	regulation of immune effector process	19/340	384/18614	8.96E-05	0.0021085	0.00160601	CD1C/TGFB2/CD177/NFKBIZ/JUNB/STAP1/TRIL/PGC/CD96/HMOX1/CD1E/IL1B/FGR/NR4A3/VSIG4/FCGR2B/RASGRP1/IL6/CX3CR1	19
BP	GO:0002695	negative regulation of leukocyte activation	13/340	202/18614	9.15E-05	0.00213394	0.00162245	TIGIT/CNR1/LAX1/HMGB3/HMOX1/FGR/VSIG4/FCGR2B/IRF1/INHBA/CX3CL1/PLA2G2A/IDO1	13
BP	GO:0051281	positive regulation of release of	6/340	41/18614	9.32E-05	0.00216451	0.00164439	APLNR/TRPC1/CAPN3/CX3CL1/CXCL10/CXCL9	6

		sequestered calcium ion into cytosol							
BP	GO:0051592	response to calcium ion	11/340	149/18614	9.41E-05	0.0021745	0.0016511	RYR2/JUN/TRPC1/FOSB/JUNB/CASQ2/JUND/CAPN3/AKR1C3/FOS/FGA	11
BP	GO:0010811	positive regulation of cell-substrate adhesion	10/340	125/18614	0.0001003	0.0023015	0.0017475	ECM2/CSPG5/SKAP1/FMN1/ABI3BP/COL8A1/CD3E/CX3CL1/HAS2/FGA	10
BP	GO:1902075	cellular response to salt	13/340	204/18614	0.0001011	0.0023066	0.0017514	P2RY1/SCNN1G/GCLM/PTGS2/EGR1/JUN/FOSB/JUNB/JUND/CAPN3/AKR1C3/SLC1A3/FOS	13
BP	GO:0006816	calcium ion transport	21/340	455/18614	0.0001034	0.0023449	0.0017805	PLCB4/TRPC6/RYR2/CXCL12/PTGS2/ANK2/GEM/PIK3CG/APLNR/TRPC1/CCL5/CYSLTR1/CASQ2/ACE/MS4A1/CAPN3/CX3CL1/CCL2/CXCL10/CCL8/CXCL9	21

BP	GO:0002688	regulation of leukocyte chemotaxis	10/340	126/18614	0.00010 72	0.00241 65	0.00183 48	CXCL12/CCL5/STAP1/DPP4/RARRES2/IL6/FPR2/CX3CR1/CCL2/CXCL10	10
BP	GO:0034764	positive regulation of transmembrane transport	14/340	235/18614	0.00011 32	0.00253 69	0.00192 62	TRPC6/RYR2/FXYD1/ANK2/APLNR/TRPC1/MS4A1/CAPN3/NR4A3/CFTR/CX3CL1/CCL2/CXCL10/CXCL9	14
BP	GO:0031589	cell-substrate adhesion	18/340	359/18614	0.00011 6	0.00258 53	0.00196 3	FREM1/ITGA10/ECM2/ITGA4/CSPG5/SKAP1/CD96/FMN1/SIGLEC1/LYVE1/VCAM1/ABI3BP/COL8A1/ADAMTS9/CD3E/CX3CL1/HAS2/FGA	18
BP	GO:0030183	B cell differentiation	11/340	153/18614	0.00011 93	0.00259 86	0.00197 31	ITGA4/NFKBIZ/MS4A1/CD79A/IL2RG/HMGB3/VCAM1/CD27/FCGR2B/INHBA/IL6	11
BP	GO:00	heterotypic cell-cell	7/340	61/18614	0.000	0.002	0.001	GLDN/ITGA4/SKAP1/IL1B/VCAM1/CD2/FGA	7

	34113	adhesion			1193	5986	9731		
BP	GO:0046456	icosanoid biosynthetic process	7/340	61/18614	0.000111193	0.002595986	0.001979731	PTGS2/NAIP/PTGDS/HPGDS/NLRC4/IL1B/AKR1C3	7
BP	GO:1905517	macrophage migration	7/340	61/18614	0.000111193	0.002595986	0.001979731	CCL5/STAP1/RARRES2/SAA1/CX3CR1/CX3CL1/CCL2	7
BP	GO:0046683	response to organophosphorus	10/340	129/18614	0.000131303	0.002828225	0.002141431	P2RY1/PTGS2/PIK3CG/DUOX1/FOSB/IL1B/AQP9/FOS/CFTR/SLC6A4	10
BP	GO:0007160	cell-matrix adhesion	14/340	239/18614	0.000131352	0.002919104	0.002220299	FREM1/ITGA10/ECM2/ITGA4/SKAP1/CD96/FMN1/SIGLEC1/LYVE1/VCAM1/ADAMTS9/CD3E/CX3CL1/FGA	14
BP	GO:0014068	positive regulation of phosphatidylin	8/340	83/18614	0.000131368	0.002929237	0.00222	HGF/TGFB2/CCL5/DDR2/FGR/DCN/NTRK2/CSF3	8

		ositol 3-kinase signaling							
BP	GO:0002675	positive regulation of acute inflammatory response	5/340	28/18614	0.0001373	0.0029237	0.002222	PTGS2/PIK3CG/CNR1/IL1B/IL6	5
BP	GO:0051209	release of sequestered calcium ion into cytosol	10/340	130/18614	0.000139	0.0029422	0.0022234	PLCB4/RYR2/ANK2/APLNR/TRPC1/CASQ2/CAPN3/CX3CL1/CXCL10/CXCL9	10
BP	GO:2001236	regulation of extrinsic apoptotic	11/340	156/18614	0.0001418	0.0029852	0.0022666	BMP5/GCLM/HGF/SRPX/HMOX1/IL1B/ATF3/INHBA/TNFSF10/CX3CL1/FGA	11

		signaling pathway							
BP	GO:0032731	positive regulation of interleukin-1 beta production	7/340	63/18614	0.0001467	0.0030714	0.0023321	NAIP/EGR1/ORM1/NLRC4/PYHIN1/IL6/GBP5	7
BP	GO:0051283	negative regulation of sequestering of calcium ion	10/340	131/18614	0.0001481	0.0030762	0.0023357	PLCB4/RYR2/ANK2/APLNR/TRPC1/CASQ2/CAPN3/CX3CL1/CXCL10/CXCL9	10
BP	GO:0045619	regulation of lymphocyte	13/340	212/18614	0.0001485	0.0030762	0.0023357	HLA-DOA/EGR3/NFKBIZ/JUNB/IL2RG/HMGB3/CD83/CD27/RHOH/RASGRP1/IRF1/INHBA/CD2	13

		differentiation							
BP	GO:0048511	rhythmic process	16/340	303/18614	0.0001534	0.0031609	0.0024	ROBO2/TGFB2/PTGDS/EGR1/TIMP4/EGR3/SLIT3/JUND/HLF/NR1D2/DTL/INHBA/NTRK2/PTX3/HAS2/SLC6A4	16
BP	GO:0030199	collagen fibril organization	7/340	64/18614	0.0001621	0.0033223	0.0025226	TGFB2/SERPINF2/DDR2/BMP1/LUM/DPT/COL14A1	7
BP	GO:0090025	regulation of monocyte chemotaxis	5/340	29/18614	0.0001635	0.0033314	0.0025295	CXCL12/CCL5/FPR2/CX3CR1/CXCL10	5
BP	GO:1903169	regulation of calcium ion transmembrane transport	12/340	186/18614	0.0001656	0.0033574	0.0025493	RYR2/ANK2/GEM/PIK3CG/APLNR/TRPC1/CASQ2/MS4A1/CAPN3/CX3CL1/CXCL10/CXCL9	12

BP	GO:0051282	regulation of sequestering of calcium ion	10/340	133/18614	0.00016 77	0.0033825	0.0025683	PLCB4/RYR2/ANK2/APLNR/TRPC1/CASQ2/CAPN3/CX3CL1/CXCL10/CXCL9	10
BP	GO:0042116	macrophage activation	9/340	109/18614	0.0001748	0.0035062	0.0026622	JUN/STAP1/JUND/VSIG4/FCGR2B/SLC11A1/IL6/CX3CR1/CX3CL1	9
BP	GO:0042180	cellular ketone metabolic process	13/340	216/18614	0.0001787	0.0035657	0.0027074	TRIB3/AKR1C2/BMP5/PTGS2/EGR1/NQO2/CNR1/SLC7A11/NQO1/IL1B/NR4A3/AKR1C3/IDO1	13
BP	GO:0031667	response to nutrient levels	21/340	477/18614	0.000198	0.0039308	0.0029846	SGIP1/PLIN2/GCLM/CBS/PTGS2/CNR1/NFKBIZ/ACE/TNFRSF11B/ALPL/HMOX1/NQO1/ZFP36/AKR1C3/VCAM1/LIPG/ATF3/FOS/CXCL10/SLC6A4/POSTN	21
BP	GO:0033002	muscle cell proliferation	14/340	249/18614	0.0002071	0.0040889	0.0031047	HGF/PTGS2/TGFB2/JUN/CCL5/SERPINF2/DDR2/HMOX1/NR4A3/TRIB1/FOS/OGN/IL6/CX3CL1	14

BP	GO:0019216	regulation of lipid metabolic process	17/340	344/18614	0.00002121	0.004109	0.0031199	ZBTB20/TRIB3/BMP5/PTGS2/IDH1/EPHX2/EGR1/PIK3CG/DHCR7/CNR1/SERPINA3/RARRES2/IL1B/FGR/NR4A3/AKR1C3/NR1D2	17
BP	GO:0051208	sequestering of calcium ion	10/340	137/18614	0.00002138	0.004109	0.0031199	PLCB4/RYR2/ANK2/APLNR/TRPC1/CASQ2/CAPN3/CX3CL1/CXCL10/CXCL9	10
BP	GO:0014066	regulation of phosphatidylinositol 3-kinase signaling	9/340	112/18614	0.00002148	0.004109	0.0031199	HGF/TGFB2/CCL5/DDR2/FGR/DCN/RASGRP1/NTRK2/CSF3	9
BP	GO:0032611	interleukin-1 beta production	9/340	112/18614	0.00002148	0.004109	0.0031199	NAIP/EGR1/ORM1/NLRC4/PYHIN1/IL6/GBP5/CX3CR1/CX3CL1	9

BP	GO:0032651	regulation of interleukin-1 beta production	9/340	112/18614	0.00002148	0.004109	0.0031199	NAIP/EGR1/ORM1/NLRC4/PYHIN1/IL6/GBP5/CX3CR1/CX3CL1	9
BP	GO:0097396	response to interleukin-17	4/340	17/18614	0.00002156	0.004109	0.0031199	NFKBIZ/IL1B/SOCS3/CXCL10	4
BP	GO:0097398	cellular response to interleukin-17	4/340	17/18614	0.00002156	0.004109	0.0031199	NFKBIZ/IL1B/SOCS3/CXCL10	4
BP	GO:0071276	cellular response to cadmium ion	5/340	31/18614	0.0000227	0.0043038	0.0032678	JUN/HMOX1/AKR1C3/FOS/MT1X	5
BP	GO:0002673	regulation of acute inflammatory	6/340	48/18614	0.00002284	0.0043093	0.003272	PTGS2/PIK3CG/CNR1/IL1B/FCGR2B/IL6	6

		response							
BP	GO:0045926	negative regulation of growth	14/340	252/18614	0.00023542	0.00439373	0.00333389	FRZB/ENO1/TGFB2/SEMA3D/SEMA6D/TNR/SLIT3/INHBA/SEMA6A/ALOX15B/SEMA3G/SLC6A4/MT1X/MT1M	14
BP	GO:1903706	regulation of hemopoiesis	19/340	415/18614	0.00024432	0.00454542	0.00034504	HLA-DOA/EGR3/NFKBIZ/JUNB/IL2RG/HMGB3/TNFRSF11B/ZFP36/CD83/CD27/RHOH/FCGR2B/RASGRP1/TRIB1/FOS/IRF1/INHBA/CD2/CSF3	19
BP	GO:0050852	T cell receptor signaling pathway	10/340	140/18614	0.00025549	0.0047176	0.00035809	BTN3A3/BTN3A1/SKAP1/NFKBIZ/TRAT1/THEMIS/CD3G/CD3E/CD3D/HLA-DQB1	10
BP	GO:1904019	epithelial cell apoptotic process	10/340	140/18614	0.00025549	0.0047176	0.00035809	RYR2/PIK3CG/ITGA4/NFKBIZ/HMOX1/ZFP36/AKR1C3/IL6/CCL2/FGA	10

BP	GO:0033628	regulation of cell adhesion mediated by integrin	6/340	49/18614	0.000256363	0.00471921	0.00358333	TGFB2/PIK3CG/CCL5/SKAP1/DPP4/CD3E	6
BP	GO:0010721	negative regulation of cell development	15/340	286/18614	0.000263838	0.00480897	0.0036522	BTG2/FRZB/SEMA3D/SEMA6D/TNR/HMGB3/TNFRSF11B/IL1B/FCGR2B/TRIB1/IRF1/INHBA/SEMA6A/IL6/SEMA3G	15
BP	GO:0001516	prostaglandin biosynthetic process	5/340	32/18614	0.0002665	0.00480897	0.0036522	PTGS2/PTGDS/HPGDS/IL1B/AKR1C3	5
BP	GO:0046457	prostanoid biosynthetic	5/340	32/18614	0.0002665	0.00480897	0.0036522	PTGS2/PTGDS/HPGDS/IL1B/AKR1C3	5

		process							
BP	GO:0050866	negative regulation of cell activation	13/340	225/18614	0.000026 64	0.004812 1	0.003653 8	TIGIT/CNR1/LAX1/HMGB3/HMOX1/FGR/VSIG4/FCGR2B/IRF1/INHBA/CX3CL1/PLA2G2A/IDO1	13
BP	GO:0006887	exocytosis	17/340	351/18614	0.000026 84	0.004825 1	0.003663 7	P2RY1/SLC18A2/PRSS12/SYTL2/PTGDS/PIK3CG/CD177/CSPG5/CCL5/HMOX1/FGR/NR4A3/FCGR2B/RASGRP1/CFTR/FGA/CCL8	17
BP	GO:0042063	gliogenesis	16/340	320/18614	0.000028 42	0.004969 2	0.003773 1	P2RY1/TGFB2/PHGDH/CNTN1/EGR2/CSPG5/STAP1/IL1B/ANO1/S100A8/IL6/NTRK2/CX3CR1/CX3CL1/CCL2/MT1X	16
BP	GO:1901607	alpha-amino acid biosynthetic process	7/340	70/18614	0.000028 5	0.004969 2	0.003773 1	PSAT1/SHMT2/PYCR1/CBS/PHGDH/BCAT1/CTH	7

BP	GO:0035296	regulation of tube diameter	10/340	142/18614	0.00002859	0.00496692	0.00377731	P2RY1/GCLM/CBS/HBB/PTGS2/HRH2/SERPINF2/ACE/FGA/SLC6A4	10
BP	GO:0051384	response to glucocorticoid	10/340	142/18614	0.00002859	0.00496692	0.00377731	PTGS2/PTGDS/FOSB/SLIT3/ACE/ALPL/ZFP36/DDIT4/FOS/IL6	10
BP	GO:0097746	blood vessel diameter maintenance	10/340	142/18614	0.00002859	0.00496692	0.00377731	P2RY1/GCLM/CBS/HBB/PTGS2/HRH2/SERPINF2/ACE/FGA/SLC6A4	10
BP	GO:0034612	response to tumor necrosis factor	14/340	257/18614	0.00002863	0.00496692	0.00377731	PTGS2/NAIP/CCL5/ZFP36/CCL23/VCAM1/FOS/PLVAP/CX3CL1/CCL2/HAS2/CCL8/SELE/CCL18	14
BP	GO:0006692	prostanoid metabolic process	6/340	50/18614	0.00002869	0.00496692	0.00377731	AKR1C2/PTGS2/PTGDS/HPGDS/IL1B/AKR1C3	6

BP	GO:0006693	prostaglandin metabolic process	6/340	50/18614	0.0002869	0.0049692	0.0037731	AKR1C2/PTGS2/PTGDS/HPGDS/IL1B/AKR1C3	6
BP	GO:0035150	regulation of tube size	10/340	143/18614	0.0003025	0.0052159	0.0039605	P2RY1/GCLM/CBS/HBB/PTGS2/HRH2/SERPINF2/ACE/FGA/SLC6A4	10
BP	GO:0050901	leukocyte tethering or rolling	5/340	33/18614	0.0003077	0.0052826	0.0040111	CXCL12/ITGA4/VCAM1/CX3CR1/SELE	5
BP	GO:0051402	neuron apoptotic process	14/340	259/18614	0.0003098	0.005294	0.0040198	BTG2/HYOU1/GCLM/NAIP/TGFB2/VSTM2L/JUN/HMOX1/NQO1/NTRK2/CX3CR1/CX3CL1/CCL2/NR4A2	14
BP	GO:0032609	type II interferon production	9/340	118/18614	0.0003177	0.0053752	0.0040814	HLA-DPA1/BTN3A1/CD96/IL1B/RASGRP1/INHBA/CD2/CD3E/SLC11A1	9

BP	GO:0032649	regulation of type II interferon production	9/340	118/18614	0.0003177	0.0053752	0.0040814	HLA-DPA1/BTN3A1/CD96/IL1B/RASGRP1/INHBA/CD2/CD3E/SLC11A1	9
BP	GO:0014065	phosphatidylinositol 3-kinase signaling	10/340	144/18614	0.0003199	0.0053752	0.0040814	HGF/TGFB2/PIK3CG/CCL5/DDR2/FGR/DCN/RASGRP1/NTRK2/CSF3	10
BP	GO:0045058	T cell selection	6/340	51/18614	0.0003202	0.0053752	0.0040814	LY9/THEMIS/CD3G/CD3E/IL6/CD3D	6
BP	GO:0071248	cellular response to metal ion	12/340	200/18614	0.0003237	0.0054101	0.0041079	PTGS2/JUN/FOSB/JUNB/JUND/HMOX1/CAPN3/NQO1/AKR1C3/FOS/MT1X/MT1M	12
BP	GO:00	phosphatidylinositol-	11/340	172/18614	0.000	0.005	0.004	PLCB4/HGF/TGFB2/PIK3CG/CCL5/DDR2/FGR/DCN/RASGRP1/NTRK2/CSF3	11

	48015	mediated signaling			3318	5225	1932		
BP	GO:0016064	immunoglobulin mediated immune response	10/340	145/18614	0.000033381	0.00055806	0.00042374	HLA-DPA1/HLA-DOA/HLA-DOB/NFKBIZ/IL2RB/CD27/FCGR2B/C1R/HLA-DQA1/HLA-DQB1	10
BP	GO:0021782	glial cell development	9/340	119/18614	0.000033383	0.00055806	0.00042374	PHGDH/CNTN1/CSPG5/IL1B/ANO1/S100A8/IL6/NTRK2/MT1X	9
BP	GO:0040013	negative regulation of locomotion	19/340	427/18614	0.000034472	0.00057036	0.00043307	CXCL12/BMP5/ROBO2/SEMA3D/SEMA6D/DPYSL3/STAP1/DPP4/HMOX1/SULF1/DCN/ADAMTS9/TRIB1/SEMA6A/ALOX15B/CX3CR1/CX3CL1/SEMA3G/CCL2	19
BP	GO:00	regulation of axon	5/340	34/18614	0.000	0.005	0.004	CXCL12/SEMA3D/SEMA6D/SEMA6A/SEMA3G	5

	48841	extension involved in axon guidance			3554	7988	403		
BP	GO:0006636	unsaturated fatty acid biosynthetic process	6/340	52/18614	0.0003564	0.0057988	0.004403	PTGS2/PTGDS/HPGDS/IL1B/AKR1C3/ALOX15B	6
BP	GO:0002831	regulation of response to biotic stimulus	20/340	463/18614	0.0003576	0.0057988	0.004403	NAIP/CCL5/NFKBIZ/TRIL/PGC/CD96/NLRC4/IL1B/FGR/VSIG4/PYHIN1/FCGR2B/RASGRP1/TRIB1/TNIP3/IRF1/FPR2/CLEC4E/GBP5/CX3CL1	20
BP	GO:0019724	B cell mediated	10/340	148/18614	0.0003981	0.0064429	0.0048815	HLA-DPA1/HLA-DOA/HLA-DOB/NFKBIZ/IL2RB/CD27/FCGR2B/C1R/HLA-DQA1/HLA-DQB1	10

		immunity							
BP	GO:0048017	inositol lipid-mediated signaling	11/340	176/18614	0.00040 37	0.0064646	0.0049085	PLCB4/HGF/TGFB2/PIK3CG/CCL5/DDR2/FGR/DCN/RASGRP1/NTRK2/CSF3	11
BP	GO:0048660	regulation of smooth muscle cell proliferation	11/340	176/18614	0.0004037	0.0064646	0.0049085	PTGS2/JUN/CCL5/SERPINF2/DDR2/HMOX1/NR4A3/TRIB1/OGN/IL6/CX3CL1	11
BP	GO:0071675	regulation of mononuclear cell migration	9/340	122/18614	0.0004067	0.006485	0.004924	CXCL12/BMP5/ITGA4/CCL5/RARRES2/FPR2/CX3CR1/CCL2/CXCL10	9
BP	GO:0062012	regulation of small molecul	16/340	331/18614	0.0004129	0.0065561	0.004978	P2RY1/ZBTB20/TRIB3/ENO1/BMP5/PTGS2/EPHX2/EGR1/DHCR7/CNR1/SLC7A11/IER3/IL1B/NR4A3/AKR1C3/DDIT4	16

		e metabolic process							
BP	GO:0002577	regulation of antigen processing and presentation	4/340	20/18614	0.000424204	0.00664476	0.00504475	HLA-DOA/HLA-DOB/FCGR2B/SLC11A1	4
BP	GO:1901605	alpha-amino acid metabolic process	12/340	206/18614	0.00042423	0.00666613	0.00505579	FAH/PSAT1/SHMT2/PYCR1/GCLM/CBS/HMGCLL1/PHGDH/SLC7A11/BCAT1/CTH/IDO1	12
BP	GO:0051604	protein maturation	16/340	332/18614	0.000424267	0.00669225	0.00508816	CORIN/RPS6KA2/ENO1/PRSS12/SERPINF2/BMP1/CPA3/ACE/CTSE/NLRC4/DPP4/IL1R2/PCSK2/CPE/C1R/FGA	16

BP	GO:0044703	multi-organism reproductive process	12/340	207/18614	0.00004418	0.00690808	0.00523397	CORIN/CBS/PTGS2/CNR1/FOSB/JUNB/ACE/IL1B/FOS/TPPP3/SLC6A4/IDO1	12
BP	GO:0048608	reproductive structure development	15/340	301/18614	0.00004529	0.00704445	0.00053489	ARID5B/BMP5/MAMLD1/ROBO2/IDH1/TGFB2/SLIT3/ACE/AKR1C3/SULF1/TNC/INHBA/TNFSF10/ALOX15B/PTX3	15
BP	GO:1901654	response to ketone	12/340	208/18614	0.00004614	0.00714472	0.00054269	SCNN1G/AKR1C2/TGFB2/FOSB/SLIT3/ACE/NQO1/AKR1C3/DDIT4/FOS/CFTR/POSTN	12
BP	GO:0002686	negative regulation of leukocyte migration	6/340	55/18614	0.00004848	0.00744425	0.00056511	CXCL12/BMP5/STAP1/DPP4/HMOX1/CCL2	6

BP	GO:0006690	icosanoid metabolic process	9/340	125/18614	0.0004861	0.0074425	0.0056511	AKR1C2/PTGS2/NAIP/PTGDS/HPGDS/NLRC4/IL1B/AKR1C3/ALOX15B	9
BP	GO:0045580	regulation of T cell differentiation	11/340	180/18614	0.0004882	0.0074425	0.0056511	HLA-DOA/EGR3/NFKBIZ/JUNB/IL2RG/CD83/CD27/RHOH/RASGRP1/IRF1/CD2	11
BP	GO:0048659	smooth muscle cell proliferation	11/340	180/18614	0.0004882	0.0074425	0.0056511	PTGS2/JUN/CCL5/SERPINF2/DDR2/HMOX1/NR4A3/TRIB1/OGN/IL6/CX3CL1	11
BP	GO:1904064	positive regulation of cation transmembrane transport	10/340	152/18614	0.0004917	0.0074652	0.0056683	RYR2/FXYD1/ANK2/APLNR/TRPC1/MS4A1/CAPN3/CX3CL1/CXCL10/CXCL9	10

BP	GO:0001818	negative regulation of cytokine production	17/340	371/18614	0.00005067	0.00766623	0.00558179	HGF/TGFB2/TIGIT/ORM1/CD96/HMOX1/ZFP36/CD83/VSIG4/IL1R2/FCGR2B/INHBA/SLC11A1/IL6/CX3CR1/CX3CL1/IDO1	17
BP	GO:1903206	negative regulation of hydrogen peroxide-induced cell death	4/340	21/18614	0.00005119	0.00776939	0.0055842	PYCR1/HGF/DDR2/NR4A3	4
BP	GO:0032729	positive regulation of type II interferon	7/340	77/18614	0.00005128	0.00776939	0.0055842	HLA-DPA1/BTN3A1/IL1B/RASGRP1/CD2/CD3E/SLC11A1	7

		production							
BP	GO:0061458	reproductive system development	15/340	305/18614	0.0005196	0.0077652	0.0058961	ARID5B/BMP5/MAMLD1/ROBO2/IDH1/TGFB2/SLIT3/ACE/AKR1C3/SULF1/TNC/INHBA/TNFSF10/ALOX15B/PTX3	15
BP	GO:1901617	organic hydroxy compound biosynthetic process	13/340	242/18614	0.0005344	0.0079554	0.0060406	P2RY1/PSAT1/BMP5/TGFB2/TRPC1/DHCR7/HDC/SLC7A11/AKR1C3/CFTR/CH25H/ALOX15B/NR4A2	13
BP	GO:1901653	cellular response to peptide	17/340	375/18614	0.0005717	0.0084772	0.0064367	TRIB3/SCNN1G/ITGA4/DDR2/SLC39A14/RARRES2/IL1B/NR4A3/VCAM1/ANO1/FCGR2B/FOS/INHBA/NR4A1/FPR2/SOCS3/NR4A2	17
BP	GO:0042113	B cell activation	14/340	276/18614	0.0005848	0.0086278	0.0065511	NFATC2/ITGA4/NFKBIZ/MS4A1/CD79A/LAX1/IL2RG/HMGB3/VCAM1/CD27/FCGR2B/RASGRP1/INHBA/IL6	14

BP	GO:0006749	glutathione metabolic process	6/340	57/18614	0.00005886	0.00086278	0.00065511	GSTM5/GCLM/IDH1/HPGDS/SLC7A11/GSTT1	6
BP	GO:0061756	leukocyte adhesion to vascular endothelial cell	6/340	57/18614	0.00005886	0.00086278	0.00065511	CXCL12/ITGA4/VCAM1/IL6/CX3CR1/SELE	6
BP	GO:0007620	copulation	4/340	22/18614	0.00006167	0.00089357	0.00067849	P2RY1/CNR1/PI3/SLC6A4	4
BP	GO:0033630	positive regulation of cell adhesion mediat	4/340	22/18614	0.00006167	0.00089357	0.00067849	TGFB2/CCL5/SKAP1/CD3E	4

		ed by integrin							
BP	GO:0071636	positive regulation of transforming growth factor beta production	4/340	22/18614	0.0006167	0.0089357	0.0067849	PTGS2/SERPINF2/LUM/CX3CL1	4
BP	GO:0044706	multi-multicellular organism process	12/340	216/18614	0.0006455	0.0093175	0.0070747	CORIN/CBS/PTGS2/CNR1/FOSB/JUNB/ACE/IL1B/FOS/TPPP3/SLC6A4/IDO1	12
BP	GO:0001820	serotonin secretion	3/340	10/18614	0.000659	0.0094058	0.0071418	SLC18A2/CNR1/SLC6A4	3
BP	GO:00	natural killer cell	3/340	10/18614	0.000	0.009	0.007	PIK3CG/CCL5/CCL2	3

	35747	chemotaxis			659	4058	1418		
BP	GO:1903596	regulation of gap junction assembly	3/340	10/18614	0.000659	0.0094058	0.0071418	APLNR/ACE/IL1B	3
BP	GO:0007565	female pregnancy	11/340	187/18614	0.0006717	0.0094807	0.0071987	CORIN/CBS/PTGS2/CNR1/FOSB/JUNB/ACE/IL1B/FOS/TPPP3/IDO1	11
BP	GO:0008217	regulation of blood pressure	11/340	187/18614	0.0006717	0.0094807	0.0071987	CORIN/RPS6KA2/SCNN1G/HBB/PTGS2/CNR1/SERPINF2/CPA3/ACE/HMOX1/IER3	11
BP	GO:0050728	negative regulation of inflammatory response	11/340	187/18614	0.0006717	0.0094807	0.0071987	HGF/SIGLEC10/ZFP36/IER3/FGR/FCGR2B/NR1D2/SAA1/FPR2/CX3CL1/SOCS3	11

BP	GO:0001990	regulation of systemic arterial blood pressure by hormone	5/340	39/18614	0.00006826	0.00959588	0.00728283	CORIN/RPS6KA2/SERPINF2/CPA3/ACE	5
BP	GO:0051235	maintenance of location	16/340	347/18614	0.0000688	0.00963392	0.0073319	PLCB4/RYR2/SLC18A2/PLIN2/ANK2/APLNR/TRPC1/CASQ2/CAPN3/IL1B/LCN2/S100A8/IL6/CX3CL1/CXCL10/CXCL9	16
BP	GO:0050673	epithelial cell proliferation	20/340	488/18614	0.00006928	0.00967706	0.00734429	CXCL12/BMP5/HGF/TGFB2/SDR16C5/JUN/APLNR/ITGA4/EGR3/NFKBIZ/HMOX1/ZFP36/NR4A3/SULF1/SFN/COL8A1/IL6/NR4A1/CCL2/HAS2	20
BP	GO:0008347	glial cell migration	6/340	59/18614	0.00007089	0.00985898	0.00748865	P2RY1/TGFB2/STAP1/CX3CR1/CX3CL1/CCL2	6
BP	GO:1901032	negative regulation of	4/340	23/18614	0.00007358	0.01019971	0.00774426	PYCR1/HGF/DDR2/NR4A3	4

		response to reactive oxygen species							
BP	GO:0090066	regulation of anatomical structure size	20/340	492/18614	0.0007661	0.0105356	0.0079997	P2RY1/OLFM1/CXCL12/GCLM/CBS/HBB/PTGS2/SEMA3D/HRH2/SERPINF2/SEMA6D/TNR/ACE/PLEKHH2/FMN1/SEMA6A/SEMA3G/CSF3/FGA/SLC6A4	20
BP	GO:0031099	regeneration	11/340	190/18614	0.0007662	0.0105356	0.0079997	CXCL12/HGF/JUN/CSPG5/TNR/ACE/HMOX1/CAPN3/NR4A3/TNC/IL6	11
BP	GO:0071634	regulation of transforming growth factor beta production	5/340	40/18614	0.0007685	0.0105356	0.0079997	PTGS2/TGFB2/SERPINF2/LUM/CX3CL1	5

BP	GO:1901655	cellular response to ketone	8/340	107/18614	0.000007751	0.001055583	0.000801169	SCNN1G/AKR1C2/ACE/AKR1C3/DDIT4/FOS/CFTR/POSTN	8
BP	GO:0002886	regulation of myeloid leukocyte mediated immunity	6/340	60/18614	0.000007757	0.001055583	0.000801169	CD177/STAP1/HMOX1/FGR/FCGR2B/CX3CR1	6
BP	GO:0051851	modulation by host of symbiont process	7/340	83/18614	0.000008066	0.0011094	0.000830367	JUN/CCL5/FASN/ZDHHC9/PTX3/CX3CR1/CCL8	7
BP	GO:0010565	regulation of cellular ketone metabolic	9/340	135/18614	0.0000848	0.001146 01	0.000087016	TRIB3/BMP5/PTGS2/EGR1/CNR1/SLC7A11/IL1B/NR4A3/AKR1C3	9

		process							
BP	GO:0002691	regulation of cellular extravasation	5/340	41/18614	0.000868623	0.011535312	0.008187556	CXCL12/ITGA4/LYVE1/PLVAP/SELE	5
BP	GO:0048246	macrophage chemotaxis	5/340	41/18614	0.000868623	0.011535312	0.008187556	CCL5/STAP1/RARRES2/SAA1/CCL2	5
BP	GO:0150077	regulation of neuroinflammatory response	5/340	41/18614	0.000868623	0.011535312	0.008187556	PTGS2/STAP1/IL1B/IL6/CX3CL1	5
BP	GO:0001660	fever generation	3/340	11/18614	0.000898939	0.011868694	0.009090124	PTGS2/CNR1/IL1B	3
BP	GO:0033089	positive regulation of T cell	3/340	11/18614	0.000898939	0.011868694	0.009090124	EGR3/IL2RG/RASGRP1	3

		differentiation in thymus							
BP	GO:0043030	regulation of macrophage activation	6/340	62/18614	0.0009239	0.0122262	0.0092833	STAP1/JUND/VSIG4/FCGR2B/IL6/CX3CL1	6
BP	GO:0006520	amino acid metabolic process	14/340	290/18614	0.0009464	0.0124802	0.0094762	FAH/PSAT1/SHMT2/PYCR1/GCLM/CBS/HMGCLL1/PHGDH/HDC/SLC7A11/BCAT1/SLC1A3/CTH/IDO1	14
BP	GO:1904037	positive regulation of epithelial cell apoptotic process	5/340	42/18614	0.0009644	0.0126737	0.0096231	ITGA4/HMOX1/AKR1C3/IL6/CCL2	5

BP	GO:0032418	lysosome localization	7/340	86/18614	0.000009965	0.01296414	0.00998415	SLC18A2/PTGDS/PIK3CG/HMOX1/FGR/NR4A3/RASGRP1	7
BP	GO:0033077	T cell differentiation in thymus	7/340	86/18614	0.000009965	0.01296414	0.00998415	EGR3/IL2RG/IL1B/RASGRP1/CD3G/CD3E/CD3D	7
BP	GO:1990849	vacuolar localization	7/340	86/18614	0.000009965	0.01296414	0.00998415	SLC18A2/PTGDS/PIK3CG/HMOX1/FGR/NR4A3/RASGRP1	7
BP	GO:0031638	zymogen activation	6/340	63/18614	0.000010058	0.01303373	0.00998992	ENO1/PRSS12/SERPINF2/NLRC4/C1R/FGA	6
BP	GO:0003081	regulation of systemic arterial blood pressure by renin-	4/340	25/18614	0.000010214	0.01315	0.00999848	RPS6KA2/SERPINF2/CPA3/ACE	4

		angiotensin							
BP	GO:0006833	water transport	4/340	25/18614	0.0010214	0.01315	0.0099848	SLC14A1/AQP9/CFTR/HAS2	4
BP	GO:0010720	positive regulation of cell development	18/340	432/18614	0.0010053	0.0135117	0.0102594	CXCL12/ROBO2/EGR2/EGR3/NFKBIZ/ACE/IL2RG/CD83/IL1B/CD27/RHOH/RASGRP1/TRIB1/FOS/IL6/NTRK2/CX3CR1/CX3CL1	18
BP	GO:0051250	negative regulation of lymphocyte activation	10/340	168/18614	0.0010669	0.0136438	0.0103597	TIGIT/LAX1/HMGB3/FGR/VSIG4/FCGR2B/IRF1/INHBA/PLA2G2A/IDO1	10
BP	GO:0071604	transforming growth factor beta	5/340	43/18614	0.0010752	0.0137041	0.0104055	PTGS2/TGFB2/SERPINF2/LUM/CX3CL1	5

BP	GO:0002440	production production of molecular mediator of immune response	15/340	328/18614	0.00108891	0.00138355	0.00105053	HLA-DPA1/TGFB2/HLA-DOA/HLA-DOB/NFKBIZ/TRIL/PGC/CD96/HMOX1/IL1B/NR4A3/FCGR2B/IL6/HLA-DQA1/HLA-DQB1	15
BP	GO:0050918	positive chemotaxis	6/340	64/18614	0.0010093	0.0013839	0.0010579	CXCL12/HGF/CCL5/FPR2/CX3CL1/CXCL10	6
BP	GO:0006979	response to oxidative stress	18/340	434/18614	0.00110092	0.00139971	0.0010628	PYCR1/GCLM/HGF/HBB/PTGS2/IDH1/JUN/DUOX1/DDR2/HMOX1/SLC7A11/NQO1/NR4A3/AKR1C3/ETV5/FOS/IL6/NR4A2	18
BP	GO:0071277	cellular response to calcium ion	7/340	88/18614	0.00114415	0.00143567	0.0010901	JUN/FOSB/JUNB/JUND/CAPN3/AKR1C3/FOS	7

BP	GO:0071241	cellular response to inorganic substance	12/340	231/18614	0.001157 5	0.014511 2	0.011018 3	PTGS2/JUN/FOSB/JUNB/JUND/HMOX1/CAPN3/NQO1/AKR1C3/FOS/MT1X/MT1M	12
BP	GO:0002887	negative regulation of myeloid leukocyte mediated immunity	3/340	12/18614	0.001175 7	0.014595 3	0.011082 2	HMOX1/FCGR2B/CX3CR1	3
BP	GO:0031650	regulation of heat generation	3/340	12/18614	0.001175 7	0.014595 3	0.011082 2	PTGS2/CNR1/IL1B	3
BP	GO:00	trans-synaptic	3/340	12/18614	0.001	0.014	0.011	CNR1/CSPG5/NTRK2	3

	99550	signaling, modulating synaptic transmission			1757	5953	0822		
BP	GO:0035994	response to muscle stretch	4/340	26/18614	0.0011898	0.0146747	0.0111425	RYR2/JUN/DDR2/FOS	4
BP	GO:1905523	positive regulation of macrophage migration	4/340	26/18614	0.0011898	0.0146747	0.0111425	CCL5/RARRES2/CX3CR1/CX3CL1	4
BP	GO:1905521	regulation of macrophage migration	5/340	44/18614	0.0011951	0.0146923	0.0111558	CCL5/STAP1/RARRES2/CX3CR1/CX3CL1	5

BP	GO:0097553	calcium ion transmembrane import into cytosol	11/340	201/18614	0.00121 24	0.01485 74	0.01128 12	PLCB4/RYR2/ANK2/APLNR/TRPC1/CASQ2/MS4A1/CAPN3/CX3CL1/CXCL10/CXCL9	11
BP	GO:0003018	vascular process in circulatory system	13/340	265/18614	0.00123 34	0.01506 56	0.01143 93	P2RY1/GCLM/CBS/HBB/PTGS2/HRH2/SERPINF2/ACE/GPR4/SLC1A3/PLVAP/FGA/SLC6A4	13
BP	GO:0051651	maintenance of location in cell	12/340	233/18614	0.00124 61	0.01517 25	0.01152 04	PLCB4/RYR2/ANK2/APLNR/TRPC1/CASQ2/CAPN3/LCN2/S100A8/CX3CL1/CXCL10/CXCL9	12
BP	GO:0031100	animal organ regeneration	6/340	66/18614	0.00128 49	0.01549 59	0.01176 6	CXCL12/HGF/ACE/HMOX1/NR4A3/IL6	6

BP	GO:0032613	interleukin-10 production	6/340	66/18614	0.0011282849	0.0154959	0.0117666	HGF/TIGIT/CD83/FCGR2B/IL6/IDO1	6
BP	GO:0032653	regulation of interleukin-10 production	6/340	66/18614	0.0011282849	0.0154959	0.0117666	HGF/TIGIT/CD83/FCGR2B/IL6/IDO1	6
BP	GO:0014910	regulation of smooth muscle cell migration	7/340	90/18614	0.0013024	0.0156087	0.0118517	CCL5/SEMA6D/DDR2/ACE/NR4A3/TRIB1/HAS2	7
BP	GO:0030516	regulation of axon extension	7/340	90/18614	0.0013024	0.0156087	0.0118517	OLFM1/CXCL12/SEMA3D/SEMA6D/TNR/SEMA6A/SEMA3G	7
BP	GO:0071356	cellular response to tumor	12/340	235/18614	0.0013402	0.0160116	0.0121575	NAIP/CCL5/ZFP36/CCL23/VCAM1/FOS/PLVAP/CX3CL1/CCL2/HAS2/CCL8/CCL18	12

		necrosis factor							
BP	GO:0061564	axon development	19/340	479/18614	0.000137	0.0163155	0.0123883	OLFM1/CXCL12/ROBO2/SEMA3D/JUN/CNTN1/ITGA4/CNR1/EGR2/CSPG5/SEMA6D/TNR/SLIT3/TNC/NRXN3/SEMA6A/NTRK2/SEMA3G/NR4A2	19
BP	GO:0009308	amine metabolic process	8/340	117/18614	0.0013862	0.0163492	0.0124139	TGFB2/HDC/SLC7A11/VCAM1/SULT1B1/MAOA/NR4A2/IDO1	8
BP	GO:0071887	leukocyte apoptotic process	8/340	117/18614	0.0013862	0.0163492	0.0124139	CXCL12/CCL5/NFKBIZ/SLC7A11/CD27/CD3G/IL6/IDO1	8
BP	GO:0006909	phagocytosis	12/340	236/18614	0.0013895	0.0163492	0.0124139	STAP1/IL2RG/SRPX/IL1B/FGR/IL2RB/FCGR2B/SLC11A1/MARCO/FPR2/PTX3/CCL2	12
BP	GO:0009620	response to fungus	6/340	67/18614	0.000139	0.0163492	0.0124139	RARRES2/S100A8/CLEC4E/PTX3/S100A12/CX3CR1	6

BP	GO:0000302	response to reactive oxygen species	11/340	205/18614	0.000142402	0.0166531	0.0126447	PYCR1/HGF/HBB/JUN/DDR2/HMOX1/NQO1/NR4A3/AKR1C3/FOS/IL6	11
BP	GO:0072593	reactive oxygen species metabolic process	12/340	237/18614	0.000144402	0.0168359	0.0127835	SLC18A2/CBS/HBB/DUOX1/CD177/DUOXA1/SH3PXD2B/NQO1/IER3/AKR1C3/DDIT4/FPR2	12
BP	GO:0046631	alpha-beta T cell activation	10/340	175/18614	0.000145513	0.0168664	0.0128066	NFKBIZ/JUNB/IL2RG/CD83/LY9/IRF1/CD3G/CD3E/IL6/CD3D	10
BP	GO:0010712	regulation of collagen metabolic	5/340	46/18614	0.000146643	0.0168664	0.0128066	MFAP4/SERPINF2/DDR2/INHBA/IL6	5

		process							
BP	GO:0014911	positive regulation of smooth muscle cell migration	5/340	46/18614	0.00146643	0.01686864	0.0128066	CCL5/SEMA6D/DDR2/NR4A3/HAS2	5
BP	GO:0030517	negative regulation of axon extension	5/340	46/18614	0.00146643	0.01686864	0.01280066	SEMA3D/SEMA6D/TNR/SEMA6A/SEMA3G	5
BP	GO:0045088	regulation of innate immune response	16/340	373/18614	0.00146649	0.01686864	0.01280066	NAIP/CCL5/NFKBIZ/TRIL/CD96/NLRC4/FGR/VSIG4/PYHIN1/FCGR2B/RASGRP1/TNIP3/IRF1/FPR2/CLEC4E/GBP5	16

BP	GO:1901216	positive regulation of neuron death	7/340	92/18614	0.0014807	0.0169963	0.0129052	TGFB2/EGR1/DDR2/NQO1/FCGR2B/DDIT4/FOS	7
BP	GO:0051091	positive regulation of DNA-binding transcription factor activity	13/340	271/18614	0.0015075	0.0172206	0.0130755	RTKN2/ARID5B/DDR2/NLRC4/CAPN3/IL1B/S100A8/CTH/IL6/S100A12/CX3CR1/CX3CL1/CSF3	13
BP	GO:0048545	response to steroid hormone	15/340	339/18614	0.0015092	0.0172206	0.0130755	SCNN1G/PTGS2/IDH1/TGFB2/PTGDS/FOSB/SLIT3/ACE/ALPL/ZFP36/NR4A3/AKR1C3/DDIT4/FOS/IL6	15
BP	GO:0002286	T cell activation involved in immune	8/340	119/18614	0.0015451	0.0174852	0.0132764	CD1C/NFKBIZ/JUNB/LY9/FCGR2B/SLC11A1/IL6/CLEC4E	8

		response							
BP	GO:0048675	axon extension	8/340	119/18614	0.00154551	0.0174852	0.0132764	OLFM1/CXCL12/SEMA3D/SEMA6D/TNR/SLIT3/SEMA6A/SEMA3G	8
BP	GO:0050670	regulation of lymphocyte proliferation	12/340	239/18614	0.00154562	0.0174852	0.0132764	HLA-DPA1/NFATC2/CCL5/IL1B/VSIG4/VCAM1/FCGR2B/IRF1/CD3E/IL6/PLA2G2A/IDO1	12
BP	GO:0050886	endocrine process	7/340	93/18614	0.00157566	0.0175934	0.0133586	CORIN/RPS6KA2/SERPINF2/CPA3/ACE/IL1B/INHBA	7
BP	GO:0003171	atrioventricular valve development	4/340	28/18614	0.00158334	0.0175934	0.0133586	OLFM1/TGFB2/APLNR/SLIT3	4
BP	GO:0003272	endocardial cushion formation	4/340	28/18614	0.00158334	0.0175934	0.0133586	BMP5/ROBO2/TGFB2/APLNR	4

BP	GO:0048843	negative regulation of axon extension involved in axon guidance	4/340	28/18614	0.00158834	0.0175934	0.0133586	SEMA3D/SEMA6D/SEMA6A/SEMA3G	4
BP	GO:1902883	negative regulation of response to oxidative stress	4/340	28/18614	0.00158834	0.0175934	0.0133586	PYCR1/HGF/DDR2/NR4A3	4
BP	GO:1903779	regulation of cardiac conduction	4/340	28/18614	0.00158834	0.0175934	0.0133586	CORIN/RYR2/ANK2/TRPC1	4

BP	GO:0001774	microglial cell activation	5/340	47/18614	0.0016145	0.0178857	0.0135806	JUN/STAP1/IL6/CX3CR1/CX3CL1	5
BP	GO:0071774	response to fibroblast growth factor	8/340	120/18614	0.0016297	0.0180025	0.0136692	GCLM/CCL5/EGR3/IER2/ZFP36/SULF1/NR4A1/CCL2	8
BP	GO:0071456	cellular response to hypoxia	9/340	149/18614	0.0016952	0.0186712	0.014177	ENO1/HYOU1/CBS/PTGS2/EGR1/DDR2/JUND/HMOX1/FOS	9
BP	GO:0007204	positive regulation of cytosolic calcium ion concentration	10/340	179/18614	0.0017173	0.0188603	0.0143206	P2RY1/TRPC6/RYR2/PIK3CG/CYSLTR1/GPR4/FPR1/P2RY10/SAA1/FPR2	10
BP	GO:00	regulation of systemi	5/340	48/18614	0.001	0.019	0.014	CORIN/RPS6KA2/SERPINF2/CPA3/ACE	5

	03044	c arterial blood pressure mediated by a chemical signal			7756	3549	6961		
BP	GO:0046189	phenol-containing compound biosynthetic process	5/340	48/18614	0.0017756	0.0193549	0.0146961	TGFB2/TRPC1/HDC/SLC7A11/NR4A2	5
BP	GO:0032944	regulation of mononuclear cell proliferation	12/340	243/18614	0.0017775	0.0193549	0.0146961	HLA-DPA1/NFATC2/CCL5/IL1B/VSIG4/VCAM1/FCGR2B/IRF1/CD3E/IL6/PLA2G2A/IDO1	12

BP	GO:0010575	positive regulation of vascular endothelial growth factor production	4/340	29/18614	0.00181806	0.01960633	0.01488848	PTGS2/IL1B/SULF1/IL6	4
BP	GO:1903205	regulation of hydrogen peroxide-induced cell death	4/340	29/18614	0.00181806	0.01960633	0.01488848	PYCR1/HGF/DDR2/NR4A3	4
BP	GO:0072678	T cell migration	6/340	71/18614	0.00187871	0.02026652	0.01538873	CXCL12/PIK3CG/ITGA4/CCL5/CCL2/CXCL10	6

BP	GO:0045059	positive thymic T cell selection	3/340	14/18614	0.00189933	0.0202727	0.0153933	CD3G/CD3E/CD3D	3
BP	GO:0071415	cellular response to purine-containing compound	3/340	14/18614	0.00189933	0.0202727	0.0153933	P2RY1/RYR2/CASQ2	3
BP	GO:0002690	positive regulation of leukocyte chemotaxis	7/340	96/18614	0.00189938	0.0202727	0.0153933	CXCL12/CCL5/RARRES2/IL6/FPR2/CX3CR1/CXCL10	7
BP	GO:0034614	cellular response to reactive	9/340	152/18614	0.0019944	0.0207525	0.0157573	PYCR1/HGF/JUN/DDR2/NQO1/NR4A3/AKR1C3/FOS/IL6	9

		oxygen species							
BP	GO:0001558	regulation of cell growth	17/340	420/18614	0.00195945	0.02080666	0.01579784	FRZB/OLFM1/CXCL12/ENO1/LTBP4/TGFB2/IL17RB/SEMA3D/SEMA6D/TNR/SLIT3/SFN/S100A8/TNC/INHBA/SEMA6A/SEMA3G	17
BP	GO:0002703	regulation of leukocyte mediated immunity	12/340	246/18614	0.00196691	0.0209034	0.01587219	CD1C/CD177/STAP1/CD96/HMOX1/CD1E/IL1B/FGR/FCGR2B/RASGRP1/IL6/CX3CR1	12
BP	GO:0062197	cellular response to chemical stress	15/340	349/18614	0.00200016	0.02115815	0.01606653	PYCR1/HGF/PTGS2/JUN/DDR2/HMOX1/CAPN3/SLC7A11/NQO1/NR4A3/AKR1C3/ETV5/FOS/IL6/NR4A2	15
BP	GO:0014909	smooth muscle cell migration	7/340	97/18614	0.00200098	0.02115815	0.01606653	CCL5/SEMA6D/DDR2/ACE/NR4A3/TRIB1/HAS2	7

BP	GO:0034109	homotypic cell-cell adhesion	7/340	97/18614	0.0020009 8	0.0211558 1	0.0160665 3	HBB/PIK3CG/MMRN1/CCL5/SLC7A11/IL6/FGA	7
BP	GO:0071677	positive regulation of mononuclear cell migration	6/340	72/18614	0.0020169	0.0211632	0.0160691	CXCL12/ITGA4/CCL5/FPR2/CX3CR1/CXCL10	6
BP	GO:0042129	regulation of T cell proliferation	10/340	183/18614	0.0020214	0.0211632	0.0160691	HLA-DPA1/CCL5/IL1B/VSIG4/VCAM1/IRF1/CD3E/IL6/PLA2G2A/IDO1	10
BP	GO:0001667	ameboidal-type cell migration	19/340	496/18614	0.0020355	0.0212523	0.0161368	ARID5B/PTGS2/TGFB2/SEMA3D/PIK3CG/JUN/ITGA4/SEMA6D/EGR3/DDR2/DPP4/HMOX1/DCN/ADAMTS9/SEMA6A/NR4A1/S100A12/SEMA3G/HAS2	19

BP	GO:0045907	positive regulation of vasoconstriction	4/340	30/18614	0.00020595	0.00213279	0.00161942	PTGS2/HRH2/ACE/FGA	4
BP	GO:0070498	interleukin-1-mediated signaling pathway	4/340	30/18614	0.00020595	0.00213279	0.00161942	EGR1/IL1B/IL1R2/IL6	4
BP	GO:0072539	T-helper 17 cell differentiation	4/340	30/18614	0.00020595	0.00213279	0.00161942	NFKBIZ/JUNB/LY9/IL6	4
BP	GO:0097306	cellular response to alcohol	7/340	98/18614	0.00021312	0.00219093	0.00166357	SCNN1G/AKR1C2/ACE/AKR1C3/FOS/INHBA/CFTR	7
BP	GO:00	leukocyte activati	5/340	50/18614	0.0002	0.0021	0.0016	JUN/STAP1/IL6/CX3CR1/CX3CL1	5

	02269	on involved in inflammatory response			1329	9093	6357		
BP	GO:1902041	regulation of extrinsic apoptotic signaling pathway via death domain receptors	5/340	50/18614	0.0021329	0.0219093	0.0166357	BMP5/HGF/HMOX1/ATF3/FGA	5
BP	GO:0003170	heart valve development	6/340	73/18614	0.0021644	0.0221139	0.016791	OLFM1/ROBO2/TGFB2/APLNR/SLIT3/ADAMTS9	6

BP	GO:0070527	platelet aggregation	6/340	73/18614	0.0021644	0.0221139	0.0167911	HBB/PIK3CG/MMRN1/SLC7A11/IL6/FGA	6
BP	GO:2001237	negative regulation of extrinsic apoptotic signaling pathway	7/340	99/18614	0.0022582	0.0230111	0.0174723	BMP5/GCLM/HGF/HMOX1/IL1B/CX3CL1/FGA	7
BP	GO:0031348	negative regulation of defense response	13/340	284/18614	0.0022771	0.0231417	0.0175715	HGF/SIGLEC10/CD96/ZFP36/IER3/FGR/VSIG4/FCGR2B/NR1D2/SAA1/FPR2/CX3CL1/SOCS3	13

BP	GO:0006801	superoxide metabolic process	6/340	74/18614	0.00231399	0.02335539	0.01773726	CBS/DUOX1/CD177/SH3PXD2B/NQO1/FPR2	6
BP	GO:0051961	negative regulation of nervous system development	9/340	156/18614	0.00232203	0.02335539	0.01773726	BTG2/ROBO2/SEMA3D/SEMA6D/TNR/IL1B/SEMA6A/IL6/SEMA3G	9
BP	GO:1902042	negative regulation of extrinsic apoptotic signaling pathwa	4/340	31/18614	0.0023331	0.02335539	0.01773726	BMP5/HGF/HMOX1/FGA	4

		y via death domain receptors							
BP	GO:0002517	T cell tolerance induction	3/340	15/18614	0.0023348	0.0233539	0.0177326	ICOS/CD3E/IDO1	3
BP	GO:0034116	positive regulation of heterotypic cell-cell adhesion	3/340	15/18614	0.0023348	0.0233539	0.0177326	SKAP1/IL1B/FGA	3
BP	GO:1902563	regulation of neutrophil activation	3/340	15/18614	0.0023348	0.0233539	0.0177326	CD177/FCGR2B/PLA2G2A	3

BP	GO:0060485	mesenchyme development	14/340	320/18614	0.000237 79	0.0023 7227	0.0018 0126	FRZB/OLFM1/BMP5/HGF/ROBO2/TGFB2/SEMA3D/APLNR/SEMA6D/IL1B/SEMA6A/IL6/SEMA3G/HAS2	14
BP	GO:0036294	cellular response to decreased oxygen levels	9/340	157/18614	0.000242 29	0.0024 1086	0.0018 3056	ENO1/HYOU1/CBS/PTGS2/EGR1/DDR2/JUND/HMOX1/FOS	9
BP	GO:0001503	ossification	17/340	429/18614	0.000243 6	0.0024 1763	0.0018 357	BMP5/HGF/CBS/PTGS2/PKDCC/VCAN/EGR2/FASN/DDR2/JUNB/JUND/BMP1/ALPL/FGR/TNC/ASPN/IL6	17
BP	GO:0002381	immunoglobulin production involved in immunoglobulin-mediat	6/340	75/18614	0.000248 37	0.0024 5849	0.0018 6673	HLA-DPA1/HLA-DOA/HLA-DOB/NFKBIZ/HLA-DQA1/HLA-DQB1	6

		ed immune response							
BP	GO:0120254	olefinic compound metabolic process	9/340	158/18614	0.002529	0.0249692	0.018959	AKR1C2/BMP5/PTGS2/PTGDS/EPHX2/EGR1/SDR16C5/AKR1C3/ALOX15B	9
BP	GO:0022602	ovulation cycle process	5/340	52/18614	0.0025398	0.0250112	0.0189909	ROBO2/TGFB2/SLIT3/INHBA/PTX3	5
BP	GO:0042542	response to hydrogen peroxide	8/340	129/18614	0.0025683	0.025226	0.019154	PYCR1/HGF/HBB/DDR2/HMOX1/NQO1/NR4A3/IL6	8
BP	GO:0051092	positive regulation of NF-	9/340	159/18614	0.0026388	0.0258523	0.0196296	RTKN2/NLRC4/CAPN3/IL1B/S100A8/CTH/S100A12/CX3CR1/CX3CL1	9

		kappaB transcription factor activity							
BP	GO:0048762	mesenchymal cell differentiation	12/340	255/18614	0.0026478	0.0258742	0.0196463	FRZB/OLFM1/BMP5/HGF/TGFB2/SEMA3D/SEMA6D/IL1B/SEMA6A/IL6/SEMA3G/HAS2	12
BP	GO:0034599	cellular response to oxidative stress	13/340	290/18614	0.0027282	0.0265591	0.0201905	PYCR1/HGF/JUN/DDR2/HMOX1/SLC7A11/NQO1/NR4A3/AKR1C3/ETV5/FOS/IL6/NR4A2	13
BP	GO:0010951	negative regulation of endopeptidase activity	9/340	160/18614	0.0027524	0.0267582	0.0203175	HGF/PTGS2/NAIP/TIMP4/SERPINF2/SERPINA3/SFN/CD27/NR4A1	9
BP	GO:00	negative regulati	10/340	191/18614	0.002	0.026	0.020	FRZB/ENO1/TGFB2/SEMA3D/SEMA6D/TNR/SLIT3/INHBA/SEMA6A/SEMA3G	10

	30308	on of cell growth			7596	7608	3194		
BP	GO:0019233	sensory perception of pain	7/340	103/18614	0.0028257	0.0271666	0.0206271	P2RY1/CXCL12/PTGS2/CNR1/ACE/ANO1/CCL2	7
BP	GO:0010715	regulation of extracellular matrix disassembly	3/340	16/18614	0.0028355	0.0271666	0.0206271	DDR2/DPP4/IL6	3
BP	GO:0042448	progesterone metabolic process	3/340	16/18614	0.0028355	0.0271666	0.0206271	AKR1C2/EGR1/AKR1C3	3
BP	GO:0006809	nitric oxide biosynthetic	6/340	77/18614	0.0028371	0.0271666	0.0206271	HBB/PTGS2/NQO1/IL1B/PTX3/CX3CR1	6

		process							
BP	GO:0050853	B cell receptor signaling pathway	6/340	77/18614	0.0028371	0.027166	0.0206271	NFATC2/NFKBIZ/STAP1/MS4A1/CD79A/FCGR2B	6
BP	GO:0019932	second-messenger-mediated signaling	13/340	292/18614	0.0028938	0.0276401	0.0209871	RYR2/NFATC2/CBS/ANK2/PDE7B/APLNR/CASQ2/TRAT1/VCAM1/FPR1/CD3E/FPR2/SELE	13
BP	GO:0007411	axon guidance	11/340	225/18614	0.0029424	0.0278578	0.0211524	CXCL12/ROBO2/SEMA3D/CNTN1/EGR2/SEMA6D/TNR/SLIT3/NRXN3/SEMA6A/SEMA3G	11
BP	GO:0097485	neuron projection guidance	11/340	225/18614	0.0029424	0.0278578	0.0211524	CXCL12/ROBO2/SEMA3D/CNTN1/EGR2/SEMA6D/TNR/SLIT3/NRXN3/SEMA6A/SEMA3G	11

BP	GO:0000096	sulfur amino acid metabolic process	4/340	33/18614	0.002945 9	0.027857 8	0.021152 4	GCLM/CBS/SLC7A11/CTH	4
BP	GO:0036474	cell death in response to hydrogen peroxide	4/340	33/18614	0.002945 9	0.027857 8	0.021152 4	PYCR1/HGF/DDR2/NR4A3	4
BP	GO:0046686	response to cadmium ion	5/340	54/18614	0.003000 4	0.028303 1	0.021490 4	JUN/HMOX1/AKR1C3/FOS/MT1X	5
BP	GO:0043154	negative regulation of cysteine-type	6/340	78/18614	0.003027 3	0.028486 2	0.021629 5	HGF/PTGS2/NAIP/SFN/CD27/NR4A1	6

		endopeptidase activity involved in apoptotic process							
BP	GO:0042060	wound healing	17/340	439/18614	0.00030835	0.00289435	0.00219767	P2RY1/HBB/TGFB2/PIK3CG/DUOX1/MMRN1/SERPINF2/DDR2/HMOX1/SLC7A11/GPR4/S100A8/SLC11A1/SAA1/IL6/CX3CL1/FGA	17
BP	GO:0030168	platelet activation	8/340	133/18614	0.00031015	0.00290411	0.00220509	P2RY1/HBB/PIK3CG/MMRN1/SLC7A11/SAA1/IL6/FGA	8
BP	GO:0061387	regulation of extent of cell growth	7/340	105/18614	0.00031476	0.00294003	0.00223236	OLFM1/CXCL12/SEMA3D/SEMA6D/TNR/SEMA6A/SEMA3G	7
BP	GO:1903522	regulation of blood	12/340	261/18614	0.0003198	0.00297985	0.00226259	CORIN/RYR2/FXYD1/PTGS2/ANK2/TGFB2/PIK3CG/TRPC1/HRH2/CASQ2/ACE/FGA	12

		circulation							
BP	GO:0019755	one-carbon compound transport	5/340	55/18614	0.00032519	0.0300385	0.0228082	HBB/SLC39A14/SLC14A1/AQP9/CFTR	5
BP	GO:1903202	negative regulation of oxidative stress-induced cell death	5/340	55/18614	0.00032519	0.0300385	0.0228082	PYCR1/HGF/DDR2/SLC7A11/NR4A3	5
BP	GO:0008406	gonad development	11/340	228/18614	0.00032553	0.0300385	0.0228082	ARID5B/MAMLD1/ROBO2/IDH1/TGFB2/SLIT3/ACE/AKR1C3/INHBA/TNFSF10/PTX3	11
BP	GO:0045055	regulated exocytosis	11/340	228/18614	0.00032553	0.0300385	0.0228082	P2RY1/SLC18A2/PTGDS/PIK3CG/CD177/CSPG5/HMOX1/FGR/NR4A3/FCGR2B/RASGRP1	11

BP	GO:0042044	fluid transport	4/340	34/18614	0.00329 13	0.0301514	0.0228939	SLC14A1/AQP9/CFTR/HAS2	4
BP	GO:0045577	regulation of B cell differentiation	4/340	34/18614	0.0032913	0.0301514	0.0228939	IL2RG/HMGB3/CD27/INHBA	4
BP	GO:2000406	positive regulation of T cell migration	4/340	34/18614	0.0032913	0.0301514	0.0228939	CXCL12/ITGA4/CCL5/CXCL10	4
BP	GO:0010566	regulation of ketone biosynthetic process	3/340	17/18614	0.0033963	0.0308169	0.0233992	BMP5/EGR1/AKR1C3	3
BP	GO:0031649	heat generation	3/340	17/18614	0.0033963	0.0308169	0.0233992	PTGS2/CNR1/IL1B	3

BP	GO:0034112	positive regulation of homotypic cell-cell adhesion	3/340	17/18614	0.0033963	0.0308169	0.0233992	MMRN1/CCL5/IL6	3
BP	GO:1903236	regulation of leukocyte tethering or rolling	3/340	17/18614	0.0033963	0.0308169	0.0233992	CXCL12/ITGA4/SELE	3
BP	GO:0001906	cell killing	10/340	197/18614	0.00344437	0.0311729	0.0236695	CD1C/LYZ/STAP1/SLAMF7/CD1E/FCGR2B/RASGRP1/CD2/S100A12/CX3CR1	10
BP	GO:0050832	defense response to fungus	5/340	56/18614	0.00351583	0.0317733	0.0241254	RARRES2/S100A8/CLEC4E/S100A12/CX3CR1	5

BP	GO:0002931	response to ischemia	5/340	57/18614	0.0038	0.0342362	0.0259954	HYOU1/EGR1/NQO1/CX3CR1/CX3CL1	5
BP	GO:0045137	development of primary sexual characteristics	11/340	233/18614	0.0038358	0.0344088	0.0261266	ARID5B/MAMLD1/ROBO2/IDH1/TGFB2/SLIT3/ACE/AKR1C3/INHBA/TNFSF10/PTX3	11
BP	GO:0002699	positive regulation of immune effector process	12/340	267/18614	0.0038373	0.0344088	0.0261266	CD1C/CD177/NFKBIZ/STAP1/PGC/HMOX1/CD1E/IL1B/FGR/NR4A3/RASGRP1/IL6	12
BP	GO:0007631	feeding behavior	7/340	109/18614	0.0038745	0.0345799	0.0262565	P2RY1/SGIP1/CNR1/ACE/NR4A3/FOS/NTRK2	7
BP	GO:19	regulation of epitheli	7/340	109/18614	0.003	0.034	0.026	ITGA4/HMOX1/ZFP36/AKR1C3/IL6/CCL2/FGA	7

	04035	al cell apoptotic process			8745	5799	2565		
BP	GO:0061844	antimicrobial humoral immune response mediated by antimicrobial peptide	6/340	82/18614	0.00388845	0.00345885	0.0026263	PGC/CXCL1/CXCL2/S100A12/CXCL10/CXCL9	6
BP	GO:0030336	negative regulation of cell migration	15/340	375/18614	0.00393972	0.00349761	0.00265573	CXCL12/BMP5/SEMA6D/DPYSL3/STAP1/DPP4/HMOX1/SULF1/DCN/ADAMTS9/TRIB1/ALOX15B/CX3CR1/CX3CL1/CCL2	15

BP	GO:0070663	regulation of leukocyte proliferation	12/340	268/18614	0.00395 32	0.03502 61	0.02659 53	HLA-DPA1/NFATC2/CCL5/IL1B/VSIG4/VCAM1/FCGR2B/IRF1/CD3E/IL6/PLA2G2A/IDO1	12
BP	GO:0009743	response to carbohydrate	11/340	234/18614	0.00396 12	0.03502 61	0.02659 53	ZBTB20/RPS6KA2/GCLM/PTGS2/EGR1/ACE/SLC39A14/NQO1/IL1B/ANO1/CFTR	11
BP	GO:0016264	gap junction assembly	3/340	18/18614	0.00402 08	0.03530 75	0.02680 89	APLNR/ACE/IL1B	3
BP	GO:0090594	inflammatory response to wounding	3/340	18/18614	0.00402 08	0.03530 75	0.02680 89	SIGLEC10/NFKBIZ/HMOX1	3
BP	GO:0150078	positive regulation of neuroinflammatory	3/340	18/18614	0.00402 08	0.03530 75	0.02680 89	STAP1/IL1B/IL6	3

BP	GO:0001909	response leukocyte mediated cytotoxicity	8/340	139/18614	0.0040583	0.0355548	0.0269967	CD1C/STAP1/SLAMF7/CD1E/FCGR2B/RASGRP1/CD2/CX3CR1	8
BP	GO:0014812	muscle cell migration	7/340	110/18614	0.0040746	0.0356156	0.0270429	CCL5/SEMA6D/DDR2/ACE/NR4A3/TRIB1/HAS2	7
BP	GO:0043616	keratinocyte proliferation	5/340	58/18614	0.0040975	0.0357337	0.0271325	SDR16C5/NFKBIZ/ZFP36/SFN/HAS2	5
BP	GO:0046209	nitric oxide metabolic process	6/340	83/18614	0.0041244	0.0358856	0.0272479	HBB/PTGS2/NQO1/IL1B/PTX3/CX3CR1	6
BP	GO:2001057	reactive nitrogen	6/340	84/18614	0.004375	0.0379794	0.0288377	HBB/PTGS2/NQO1/IL1B/PTX3/CX3CR1	6

		species metabolic process							
BP	GO:0043281	regulation of cysteine-type endopeptidase activity involved in apoptotic process	10/340	204/18614	0.004405	0.0381211	0.0289452	HGF/PTGS2/NAIP/NLRC4/UACA/SFN/CD27/S100A8/TNFSF10/NR4A1	10
BP	GO:0031102	neuron projection regeneration	5/340	59/18614	0.0044113	0.0381211	0.0289452	HGF/JUN/CSPG5/TNR/TNC	5
BP	GO:00	cellular response to	9/340	172/18614	0.00444	0.038	0.029	ENO1/HYOU1/CBS/PTGS2/EGR1/DDR2/JUND/HMOX1/FOS	9

	71453	oxygen levels				1957	0019		
BP	GO:1990138	neuron projection extension	9/340	172/18614	0.00444	0.0381957	0.0290019	OLFM1/CXCL12/SEMA3D/ITGA4/SEMA6D/TNR/SLIT3/SEMA6A/SEMA3G	9
BP	GO:0007617	mating behavior	4/340	37/18614	0.0044906	0.0384578	0.0292009	P2RY1/CNR1/PI3/SLC6A4	4
BP	GO:0008207	C21-steroid hormone metabolic process	4/340	37/18614	0.0044906	0.0384578	0.0292009	AKR1C2/BMP5/EGR1/AKR1C3	4
BP	GO:0050767	regulation of neurogenesis	15/340	381/18614	0.0045525	0.0387745	0.0294414	BTG2/CXCL12/ROBO2/SEMA3D/EGR2/SEMA6D/TNR/ACE/IL1B/SEMA6A/IL6/NTRK2/CX3CR1/CX3CL1/SEMA3G	15
BP	GO:00	cell growth	18/340	495/18614	0.004	0.038	0.029	FRZB/OLFM1/CXCL12/ENO1/LTBP4/TGFB2/IL17RB/SEMA3D/ITGA4/SEMA6D/TNR/SLIT3/SFN/S100A8/TNC/INHBA/SEMA6A/SEMA3G	18

	16049				5537	7745	4414		
BP	GO:0002819	regulation of adaptive immune response	10/340	205/18614	0.00455581	0.00387745	0.00294414	CD1C/SKAP1/NFKBIZ/JUNB/CD1E/IL1B/FCGR2B/IRF1/SLC11A1/IL6	10
BP	GO:0002523	leukocyte migration involved in inflammatory response	3/340	19/18614	0.00047108	0.00398152	0.00302316	S100A8/CX3CL1/SELE	3
BP	GO:0043031	negative regulation of macrophage	3/340	19/18614	0.00047108	0.00398152	0.00302316	VSIG4/FCGR2B/CX3CL1	3

BP	GO:0007200	activation phospholipase C-activating G protein-coupled receptor signaling pathway	7/340	113/18614	0.00047222	0.03981852	0.03023316	P2RY1/GPR4/FPR1/P2RY10/ANO1/FPR2/CX3CR1	7
BP	GO:0044344	cellular response to fibroblast growth factor stimulus	7/340	113/18614	0.00047222	0.03981852	0.03023316	GCLM/CCL5/EGR3/ZFP36/SULF1/NR4A1/CCL2	7

BP	GO:0003179	heart valve morphogenesis	5/340	60/18614	0.00047418	0.0398917	0.03028297	OLFM1/ROBO2/TGFB2/SLIT3/ADAMTS9	5
BP	GO:1904062	regulation of monoatomic cation transmembrane transport	14/340	346/18614	0.00047623	0.0399757	0.03035335	RYR2/FXYD1/KCNAB1/ANK2/GEM/PIK3CG/APLNR/TRPC1/CASQ2/MS4A1/CAPN3/CX3CL1/CXCL10/CXCL9	14
BP	GO:0061448	connective tissue development	12/340	275/18614	0.00048456	0.04058557	0.03081166	FRZB/ARID5B/BMP5/CBS/PKDCC/EGR1/BMP1/SLC39A14/SH3PXD2B/GPR4/SULF1/OGN	12
BP	GO:0002335	mature B cell differentiation	4/340	38/18614	0.00049479	0.04100467	0.03116667	NFKBIZ/IL2RG/FCGR2B/IL6	4
BP	GO:00	regulation of transcri	4/340	38/18614	0.004	0.041	0.031	EGR1/JUN/HMOX1/ATF3	4

	43618	ption from RNA polymerase II promoter in response to stress			9479	0467	1667		
BP	GO:0051385	response to mineralocorticoid	4/340	38/18614	0.0049479	0.0410467	0.0311667	SCNN1G/FOSB/ACE/FOS	4
BP	GO:1901661	quinone metabolic process	4/340	38/18614	0.0049479	0.0410467	0.0311667	AKR1C2/NQO2/NQO1/AKR1C3	4
BP	GO:0033559	unsaturated fatty acid metabo	7/340	114/18614	0.0049545	0.0410467	0.0311667	AKR1C2/PTGS2/PTGDS/HPGDS/IL1B/AKR1C3/ALOX15B	7

		lic process							
BP	GO:0051960	regulation of nervous system development	17/340	461/18614	0.000501093	0.04149439	0.03150662	BTG2/CXCL12/HGF/ROBO2/SEMA3D/EGR2/SEMA6D/TNR/ACE/IL1B/LRRN3/SEMA6A/IL6/NTRK2/CX3CR1/CX3CL1/SEMA3G	17
BP	GO:0009636	response to toxic substance	11/340	242/18614	0.000508871	0.0417712	0.03167718	SLC18A2/HBB/PTGS2/EPHX2/DUOX1/CCL5/SLC7A11/NQO1/FOS/GSTT1/SLC6A4	11
BP	GO:0050994	regulation of lipid catabolic process	5/340	61/18614	0.000508895	0.0417712	0.03167718	IDH1/PIK3CG/CNR1/RARRES2/IL1B	5
BP	GO:0051480	regulation of cytosolic	5/340	61/18614	0.000508895	0.0417712	0.03167718	P2RY1/TRPC6/RYR2/TRPC1/CNR1	5

BP	GO:0060135	calcium ion concentration maternal process involved in female pregnancy	5/340	61/18614	0.00508895	0.0417172	0.03167718	CBS/PTGS2/CNR1/JUNB/TPPP3	5
BP	GO:0042886	amide transport	14/340	349/18614	0.0051315	0.04187759	0.03179763	CNR1/CCL5/SLC7A11/IL1B/SLC14A1/AQP9/ANO1/S100A8/SLC1A3/CPE/CFTR/IL6/NTRK2/FGA	14
BP	GO:0045765	regulation of angiogenesis	14/340	349/18614	0.0051315	0.04187759	0.03179763	HGF/TGFB2/APLNR/HMOX1/IL1B/GPR4/STAB1/SULF1/DCN/ADAMTS9/SEMA6A/IL6/CX3CR1/CXCL10	14
BP	GO:0001960	negative regulation of cytokin	6/340	87/18614	0.00519943	0.04212252	0.03198855	NAIP/CCL5/SLIT3/STAP1/IL1R2/IL6	6

		e-mediated signaling pathway							
BP	GO:0001938	positive regulation of endothelial cell proliferation	7/340	115/18614	0.0051952	0.0421252	0.0319855	CXCL12/JUN/APLNR/ITGA4/EGR3/HMOX1/NR4A1	7
BP	GO:0035710	CD4-positive, alpha-beta T cell activation	7/340	115/18614	0.0051952	0.0421252	0.0319855	NFKBIZ/JUNB/IL2RG/CD83/LY9/CD3E/IL6	7
BP	GO:0007517	muscle organ development	14/340	351/18614	0.0053902	0.0435946	0.0331013	BTG2/RYR2/ARID5B/TGFB2/EGR1/EGR2/EGR3/HLF/CAPN3/NR1D2/ATF3/FOS/NR4A1/CXCL10	14

BP	GO:0032350	regulation of hormone metabolic process	4/340	39/18614	0.00543 53	0.04359 46	0.03310 13	BMP5/EGR1/DUOXA1/AKR1C3	4
BP	GO:0045582	positive regulation of T cell differentiation	7/340	116/18614	0.00544 46	0.04359 46	0.03310 13	EGR3/NFKBIZ/IL2RG/CD83/CD27/RHOH/RASGRP1	7
BP	GO:0022617	extracellular matrix disassembly	5/340	62/18614	0.00545 49	0.04359 46	0.03310 13	ADAMTS15/DDR2/SH3PXD2B/DPP4/IL6	5
BP	GO:0045428	regulation of nitric oxide biosynthetic	5/340	62/18614	0.00545 49	0.04359 46	0.03310 13	HBB/PTGS2/IL1B/PTX3/CX3CR1	5

		process							
BP	GO:0009914	hormone transport	13/340	315/18614	0.00546	0.0435946	0.0331013	P2RY1/SLC18A2/LTBP4/CNR1/SLCO4A1/CCL5/IL1B/ANO1/CPE/INHBA/CFTR/IL6/FGA	13
BP	GO:1903975	regulation of glial cell migration	3/340	20/18614	0.0054679	0.0435946	0.0331013	STAP1/CX3CR1/CX3CL1	3
BP	GO:1903978	regulation of microglial cell activation	3/340	20/18614	0.0054679	0.0435946	0.0331013	STAP1/IL6/CX3CL1	3
BP	GO:0033273	response to vitamin	6/340	88/18614	0.0054908	0.0436858	0.0331706	CBS/PTGS2/NFKBIZ/ALPL/CXCL10/POSTN	6
BP	GO:2000146	negative regulation of	15/340	390/18614	0.0056204	0.0446234	0.0338824	CXCL12/BMP5/SEMA6D/DPYSL3/STAP1/DPP4/HMOX1/SULF1/DCN/ADAMTS9/TRIB1/ALOX15B/CX3CR1/CX3CL1/CCL2	15

		cell motility							
BP	GO:0045444	fat cell differentiation	11/340	246/18614	0.0057378	0.0454601	0.0345178	FRZB/TRIB3/ARID5B/PTGS2/EGR2/ZFP36/RARRES2/NR4A3/IL6/NR4A1/NR4A2	11
BP	GO:0051047	positive regulation of secretion	13/340	317/18614	0.00574496	0.0454601	0.0345178	P2RY1/CXCL12/TGFB2/CD177/IL1B/FGR/MYH10/ANO1/S100A8/INHBA/CFTR/FGA/SLC6A4	13
BP	GO:0043367	CD4-positive, alpha-beta T cell differentiation	6/340	89/18614	0.00579796	0.0456656	0.0346738	NFKBIZ/JUNB/IL2RG/CD83/LY9/IL6	6
BP	GO:0051591	response to cAMP	6/340	89/18614	0.00579796	0.0456656	0.0346738	PIK3CG/DUOX1/FOSB/AQP9/FOS/CFTR	6
BP	GO:0032623	interleukin-2 production	5/340	63/18614	0.00583884	0.0456885	0.0346912	ZFP36/CD83/IL1B/VSIG4/CD3E	5

BP	GO:0032663	regulation of interleukin-2 production	5/340	63/18614	0.0058384	0.0456885	0.0346912	ZFP36/CD83/IL1B/VSIG4/CD3E	5
BP	GO:0035904	aorta development	5/340	63/18614	0.0058384	0.0456885	0.0346912	ROBO2/TGFB2/APLNR/EGR2/ADAMTS9	5
BP	GO:1901342	regulation of vasculature development	14/340	355/18614	0.0059392	0.0462444	0.0351132	HGF/TGFB2/APLNR/HMOX1/IL1B/GPR4/STAB1/SULF1/DCN/ADAMTS9/SEMA6A/IL6/CX3CR1/CXCL10	14
BP	GO:1901031	regulation of response to reactive oxygen species	4/340	40/18614	0.0059539	0.0462444	0.0351132	PYCR1/HGF/DDR2/NR4A3	4

BP	GO:1904994	regulation of leukocyte adhesion to vascular endothelial cell	4/340	40/18614	0.00595939	0.04624244	0.03511132	CXCL12/ITGA4/IL6/SELE	4
BP	GO:0050830	defense response to Gram-positive bacterium	7/340	118/18614	0.0059701	0.04624244	0.03511132	LYZ/RARRES2/IL1B/FGR/IL6/GBP4/PLA2G2A	7
BP	GO:0051702	biological process involved in interact	7/340	118/18614	0.0059701	0.04624244	0.03511132	JUN/CCL5/FASN/ZDHHC9/PTX3/CX3CR1/CCL8	7

		ion with symbiont							
BP	GO:0007548	sex differentiation	12/340	283/18614	0.00060553	0.04680687	0.03554718	ARID5B/BMP5/MAMLD1/ROBO2/IDH1/TGFB2/SLIT3/ACE/AKR1C3/INHBA/TNFSF10/PTX3	12
BP	GO:2000106	regulation of leukocyte apoptotic process	6/340	90/18614	0.00061208	0.04721193	0.03585835	CXCL12/CCL5/SLC7A11/CD27/CD3G/IDO1	6
BP	GO:0070050	neuron cellular homeostasis	5/340	64/18614	0.00062406	0.04780637	0.03629972	P2RY1/CNR1/NELL2/IL6/CX3CL1	5
BP	GO:0071384	cellular response to corticosteroid stimulus	5/340	64/18614	0.00062406	0.04780637	0.03629972	SCNN1G/ACE/ZFP36/AKR1C3/DDIT4	5

BP	GO:0002718	regulation of cytokine production involved in immune response	7/340	119/18614	0.0062467	0.0478037	0.0362972	TGFB2/TRIL/CD96/HMOX1/IL1B/NR4A3/IL6	7
BP	GO:0046632	alpha-beta T cell differentiation	7/340	119/18614	0.0062467	0.0478037	0.0362972	NFKBIZ/JUNB/IL2RG/CD83/LY9/IRF1/IL6	7
BP	GO:0006837	serotonin transport	3/340	21/18614	0.0062939	0.0479722	0.0364252	SLC18A2/CNR1/SLC6A4	3
BP	GO:1901739	regulation of myoblast fusion	3/340	21/18614	0.0062939	0.0479722	0.0364252	NFATC2/CXCL10/CXCL9	3

BP	GO:0043491	protein kinase B signaling	10/340	215/18614	0.00633304	0.0481539	0.03656232	AKR1C2/PIK3CG/CCL5/IL1B/AKR1C3/NTRK2/CX3CR1/CX3CL1/CCL2/CSF3	10
BP	GO:0046890	regulation of lipid biosynthetic process	9/340	182/18614	0.00633856	0.04829945	0.03666699	ZBTB20/TRIB3/BMP5/PTGS2/IDH1/EGR1/DHCR7/IL1B/AKR1C3	9
BP	GO:0050768	negative regulation of neurogenesis	8/340	150/18614	0.00633869	0.04829945	0.03666699	BTG2/SEMA3D/SEMA6D/TNR/IL1B/SEMA6A/IL6/SEMA3G	8
BP	GO:1903038	negative regulation of leukocyte cell-cell	8/340	150/18614	0.00633869	0.04829945	0.03666699	CXCL12/TIGIT/LAX1/VSIG4/FCGR2B/IRF1/PLA2G2A/IDO1	8

		adhesion							
BP	GO:2000117	negative regulation of cysteine-type endopeptidase activity	6/340	91/18614	0.00645448	0.0487112	0.0369863	HGF/PTGS2/NAIP/SFN/CD27/NR4A1	6
BP	GO:0003203	endocardial cushion morphogenesis	4/340	41/18614	0.00650543	0.0487795	0.0370499	BMP5/ROBO2/TGFB2/APLNR	4
BP	GO:0032965	regulation of collagen biosynthetic process	4/340	41/18614	0.00650543	0.0487795	0.0370499	SERPINF2/DDR2/INHBA/IL6	4

BP	GO:2000403	positive regulation of lymphocyte migration	4/340	41/18614	0.006504 3	0.048795	0.037049 9	CXCL12/ITGA4/CCL5/CXCL10	4
BP	GO:0016525	negative regulation of angiogenesis	8/340	151/18614	0.006640 2	0.049489 7	0.037577 4	TGFB2/GPR4/STAB1/SULF1/DCN/ADAMTS9/SEMA6A/CXCL10	8
BP	GO:0006879	intracellular iron ion homeostasis	5/340	65/18614	0.006661 8	0.049489 7	0.037577 4	SLC39A14/HMOX1/LCN2/SLC11A1/CP	5
BP	GO:0031663	lipopolysaccharide-mediated signaling	5/340	65/18614	0.006661 8	0.049489 7	0.037577 4	CCL5/IL1B/TRIB1/CX3CL1/CCL2	5

		pathway							
BP	GO:0071715	icosanoid transport	5/340	65/18614	0.00666618	0.0494897	0.0375774	PTGS2/SLCO4A1/ACE/IL1B/PLA2G2A	5
BP	GO:0080164	regulation of nitric oxide metabolic process	5/340	65/18614	0.00666618	0.0494897	0.0375774	HBB/PTGS2/IL1B/PTX3/CX3CR1	5
BP	GO:0070588	calcium ion transmembrane transport	14/340	360/18614	0.00668677	0.0495849	0.0376497	PLCB4/TRPC6/RYR2/ANK2/GEM/PIK3CG/APLNR/TRPC1/CASQ2/MS4A1/CAPN3/CX3CL1/CXCL10/CXCL9	14
BP	GO:0060761	negative regulation of respon	6/340	92/18614	0.00680018	0.0502701	0.03817	NAIP/CCL5/SLIT3/STAP1/IL1R2/IL6	6

		se to cytokine stimulus							
BP	GO:0002367	cytokine production involved in immune response	7/340	121/18614	0.0068284	0.0502701	0.03817	TGFB2/TRIL/CD96/HMOX1/IL1B/NR4A3/IL6	7
BP	GO:1902107	positive regulation of leukocyte differentiation	9/340	184/18614	0.0068432	0.0502701	0.03817	EGR3/NFKBIZ/IL2RG/CD83/CD27/RHOH/RASGRP1/TRIB1/FOS	9
BP	GO:1903708	positive regulation of	9/340	184/18614	0.0068432	0.0502701	0.03817	EGR3/NFKBIZ/IL2RG/CD83/CD27/RHOH/RASGRP1/TRIB1/FOS	9

BP	GO:0050878	hemopoiesis regulation of body fluid levels	14/340	361/18614	0.0068846	0.0502701	0.0383817	P2RY1/SCNN1G/HBB/PIK3CG/APLNR/MMRN1/SERPINF2/SLC7A11/ANO1/CFTR/SAA1/IL6/HAS2/FGA	14
BP	GO:0001936	regulation of endothelial cell proliferation	9/340	185/18614	0.0070812	0.0514115	0.0390367	CXCL12/JUN/APLNR/ITGA4/EGR3/HMOX1/SULF1/NR4A1/CCL2	9
BP	GO:0032733	positive regulation of interleukin-10 production	4/340	42/18614	0.0070874	0.0514115	0.0390367	HGF/TIGIT/CD83/IL6	4
BP	GO:0042119	neutrophil activation	4/340	42/18614	0.0070874	0.0514115	0.0390367	CD177/CCL5/FCGR2B/PLA2G2A	4

BP	GO:0045429	positive regulation of nitric oxide biosynthetic process	4/340	42/18614	0.00708874	0.05141415	0.03903367	HBB/PTGS2/IL1B/PTX3	4
BP	GO:0050771	negative regulation of axonogenesis	5/340	66/18614	0.00710026	0.05141415	0.03903367	SEMA3D/SEMA6D/TNR/SEMA6A/SEMA3G	5
BP	GO:1903532	positive regulation of secretion by cell	12/340	289/18614	0.00711107	0.05141415	0.03903367	P2RY1/CXCL12/TGFB2/CD177/IL1B/FGR/MYH10/ANO1/INHBA/CFTR/FGA/SLC6A4	12
BP	GO:0034765	regulation of monoatomic ion	17/340	478/18614	0.00711171	0.05141415	0.03903367	TRPC6/RYR2/FXYD1/KCNAB1/ANK2/GEM/PIK3CG/APLNR/TRPC1/CASQ2/MS4A1/CAPN3/CFTR/CX3CL1/CCL2/CXCL10/CXCL9	17

		transmembrane transport							
BP	GO:0070301	cellular response to hydrogen peroxide	6/340	93/18614	0.0071622	0.0514115	0.0390367	PYCR1/HGF/DDR2/NQO1/NR4A3/IL6	6
BP	GO:2000181	negative regulation of blood vessel morphogenesis	8/340	153/18614	0.0071697	0.0514115	0.0390367	TGFB2/GPR4/STAB1/SULF1/DCN/ADAMTS9/SEMA6A/CXCL10	8
BP	GO:0009713	catechol-containing compound	3/340	22/18614	0.0071903	0.0514115	0.0390367	TGFB2/HDC/NR4A2	3

		biosynthetic process							
BP	GO:0010881	regulation of cardiac muscle contraction by regulation of the release of sequestered calcium ion	3/340	22/18614	0.0071903	0.05141 15	0.03903 67	RYR2/ANK2/CASQ2	3
BP	GO:0042423	catecholamine biosynthetic process	3/340	22/18614	0.00719 03	0.05141 15	0.03903 67	TGFB2/HDC/NR4A2	3

BP	GO:0045061	thymic T cell selection	3/340	22/18614	0.007190303	0.051411 5	0.039036 7	CD3G/CD3E/CD3D	3
BP	GO:1902644	tertiary alcohol metabolic process	3/340	22/18614	0.007190303	0.0514115	0.0390367	AKR1C2/BMP5/AKR1C3	3
BP	GO:0043405	regulation of MAP kinase activity	9/340	186/18614	0.007323254	0.0522796	0.0396958	TRIB3/CBS/PIK3CG/LAX1/FGD2/IL1B/RASGRP1/TRIB1/S100A12	9
BP	GO:0002833	positive regulation of response to biotic stimulus	13/340	327/18614	0.007387 2	0.0525233	0.0398809	NAIP/CCL5/NFKBIZ/TRIL/PGC/NLRC4/PYHIN1/RASGRP1/TNIP3/IRF1/FPR2/CLEC4E/GBP5	13
BP	GO:00	cellular response to	13/340	327/18614	0.007	0.052	0.039	PLIN2/CBS/PTGS2/ITGA4/HMOX1/IL1B/AKR1C3/VCAM1/ATF3/FOS/IRF1/NR4A2/POSTN	13

	71496	external stimulus			3872	5233	8809		
BP	GO:1901343	negative regulation of vasculature development	8/340	154/18614	0.00744461	0.0528439	0.0401243	TGFB2/GPR4/STAB1/SULF1/DCN/ADAMTS9/SEMA6A/CXCL10	8
BP	GO:0003073	regulation of systemic arterial blood pressure	6/340	94/18614	0.00753361	0.0533369	0.0405523	CORIN/RPS6KA2/SERPINF2/CPA3/ACE/IER3	6
BP	GO:0034605	cellular response to heat	5/340	67/18614	0.00756632	0.0533369	0.0405523	PTGS2/HMOX1/ANO1/TFEC/CXCL10	5

BP	GO:0070613	regulation of protein processing	5/340	67/18614	0.00756632	0.0536369	0.0405523	RPS6KA2/ENO1/SERPINF2/NLRC4/IL1R2	5
BP	GO:0019722	calcium-mediated signaling	9/340	187/18614	0.00757761	0.0536369	0.0405523	RYR2/NFATC2/ANK2/CASQ2/TRAT1/VCAM1/CD3E/FPR2/SELE	9
BP	GO:0014002	astrocyte development	4/340	43/18614	0.00770041	0.0538872	0.0409049	IL1B/S100A8/IL6/MT1X	4
BP	GO:0019098	reproductive behavior	4/340	43/18614	0.00770041	0.0538872	0.0409049	P2RY1/CNR1/PI3/SLC6A4	4
BP	GO:0042554	superoxide anion generation	4/340	43/18614	0.00770041	0.0538872	0.0409049	DUOX1/CD177/SH3PXD2B/FPR2	4

BP	GO:0060412	ventricular septum morphogenesis	4/340	43/18614	0.0077041	0.0538872	0.0409049	ROBO2/TGFB2/APLNR/SLIT3	4
BP	GO:0050770	regulation of axonogenesis	8/340	155/18614	0.0077305	0.0539576	0.0409699	CXCL12/ROBO2/SEMA3D/SEMA6D/TNR/SEMA6A/NTRK2/SEMA3G	8
BP	GO:0023061	signal release	17/340	483/18614	0.0078536	0.0546666	0.0415082	P2RY1/SLC18A2/CXCL12/LTBP4/PTGS2/CNR1/CSPG5/CCL5/IL1B/ANO1/CPE/INHBA/CFTR/IL6/NR4A1/FGA/SLC6A4	17
BP	GO:0010810	regulation of cell-substrate adhesion	10/340	222/18614	0.0078607	0.0546666	0.0415082	ECM2/CSPG5/SKAP1/FMN1/ABI3BP/COL8A1/CD3E/CX3CL1/HAS2/FGA	10
BP	GO:0001910	regulation of leukocyte mediated	6/340	95/18614	0.0079924	0.054906	0.04169	CD1C/STAP1/CD1E/FCGR2B/RASGRP1/CX3CR1	6

BP	GO:0046330	positive regulation of JNK cascade	6/340	95/18614	0.00792 4	0.0549 06	0.04169	NAIP/SERPINF2/IL1B/CD27/FCGR2B/RASGRP1	6
BP	GO:2000401	regulation of lymphocyte migration	5/340	68/18614	0.0080443	0.0556388	0.0422464	CXCL12/ITGA4/CCL5/CCL2/CXCL10	5
BP	GO:0002700	regulation of production of molecular mediator of immune response	9/340	189/18614	0.0080971	0.0558975	0.0424428	TGFB2/TRIL/PGC/CD96/HMOX1/IL1B/NR4A3/FCGR2B/IL6	9

BP	GO:0002456	T cell mediated immunity	7/340	125/18614	0.00811111	0.0558975	0.0424428	CD1C/BTN3A3/NFKBIZ/CD1E/IL1B/FCGR2B/IL6	7
BP	GO:0002689	negative regulation of leukocyte chemotaxis	3/340	23/18614	0.00815885	0.055921	0.0424607	STAP1/DPP4/CCL2	3
BP	GO:0061760	antifungal innate immune response	3/340	23/18614	0.00815885	0.055921	0.0424607	RARRES2/CLEC4E/CX3CR1	3
BP	GO:2000353	positive regulation of endothelial cell	3/340	23/18614	0.00815885	0.055921	0.0424607	ITGA4/AKR1C3/CCL2	3

		apoptotic process							
BP	GO:0010976	positive regulation of neuron projection development	8/340	157/18614	0.00832336	0.05645453	0.04286864	BMP5/HGF/CNTN1/CNR1/DPYSL3/DDR2/NTRK2/CX3CL1	8
BP	GO:0033003	regulation of mast cell activation	4/340	44/18614	0.0083355	0.05645453	0.04286864	CNR1/HMOX1/FGR/NR4A3	4
BP	GO:0043620	regulation of DNA-templated transcription in respon	4/340	44/18614	0.0083355	0.05645453	0.04286864	EGR1/JUN/HMOX1/ATF3	4

		se to stress							
BP	GO:0071526	semaphorin-plexin signaling pathway	4/340	44/18614	0.0083555	0.0564553	0.0428664	SEMA3D/SEMA6D/SEMA6A/SEMA3G	4
BP	GO:0072538	T-helper 17 type immune response	4/340	44/18614	0.0083555	0.0564553	0.0428664	NFKBIZ/JUNB/LY9/IL6	4
BP	GO:1904407	positive regulation of nitric oxide metabolic process	4/340	44/18614	0.0083555	0.0564553	0.0428664	HBB/PTGS2/IL1B/PTX3	4

BP	GO:1904646	cellular response to amyloid-beta	4/340	44/18614	0.0083355	0.0564553	0.0428664	ITGA4/VCAM1/FCGR2B/FPR2	4
BP	GO:2000516	positive regulation of CD4-positive, alpha-beta T cell activation	4/340	44/18614	0.0083355	0.0564553	0.0428664	NFKBIZ/IL2RG/CD83/CD3E	4
BP	GO:0003206	cardiac chamber morphogenesis	7/340	126/18614	0.0084577	0.0569478	0.0432403	RYR2/BMP5/ROBO2/TGFB2/APLNR/SLIT3/CPE	7
BP	GO:0045471	response to ethanol	7/340	126/18614	0.0084577	0.0569478	0.0432403	CNR1/NQO1/VCAM1/CD27/S100A8/FOS/CSF3	7

BP	GO:0006081	cellular aldehyde metabolic process	5/340	69/18614	0.00854463	0.0574424	0.0436159	BMP5/IDH1/SDR16C5/ALPL/AKR1C3	5
BP	GO:0050679	positive regulation of epithelial cell proliferation	10/340	225/18614	0.00859778	0.0576871	0.0438017	CXCL12/BMP5/JUN/APLNR/ITGA4/EGR3/HMOX1/NR4A3/NR4A1/HAS2	10
BP	GO:0006576	biogenic amine metabolic process	6/340	97/18614	0.00874424	0.0585544	0.0444603	TGFB2/HDC/SULT1B1/MAOA/NR4A2/IDO1	6
BP	GO:0003014	renal system process	7/340	127/18614	0.00888152	0.058938	0.0447515	CORIN/HBB/SERPINF2/ACE/AKR1C3/SULF1/HAS2	7

BP	GO:0031295	T cell costimulation	4/340	45/18614	0.009040909	0.060320606	0.045801 3	DPP4/ICOS/CD5/CD3E	4
BP	GO:0014823	response to activity	5/340	70/18614	0.009069 5	0.060320606	0.045801 3	RYR2/GCLM/CAPN3/FOS/IL6	5
BP	GO:0032722	positive regulation of chemokine production	5/340	70/18614	0.0090695	0.0603206	0.0458013	EGR1/HMOX1/IL1B/ALOX15B/IL6	5
BP	GO:0052548	regulation of endopeptidase activity	13/340	336/18614	0.0091582	0.0605526	0.0459774	HGF/PTGS2/NAIP/TIMP4/SERPINF2/SERPINA3/NLRC4/UACA/SFN/CD27/S100A8/TNFSF10/NR4A1	13
BP	GO:0043279	response to alkaloid	6/340	98/18614	0.0091736	0.0605526	0.0459774	RYR2/CNR1/FOSB/CASQ2/NQO1/SLC1A3	6

BP	GO:0002922	positive regulation of humoral immune response	3/340	24/18614	0.00919997	0.06055526	0.04597774	PGC/IL1B/FCGR2B	3
BP	GO:0048143	astrocyte activation	3/340	24/18614	0.00919997	0.06055526	0.04597774	IL1B/IL6/MT1X	3
BP	GO:0070233	negative regulation of T cell apoptotic process	3/340	24/18614	0.00919997	0.06055526	0.04597774	CCL5/CD27/IDO1	3
BP	GO:0120255	olefinic compound biosynt	3/340	24/18614	0.00919997	0.06055526	0.04597774	BMP5/EGR1/AKR1C3	3

		hetic process							
BP	GO:0010975	regulation of neuron projection development	16/340	452/18614	0.0092686	0.0609014	0.0462423	CXCL12/BMP5/HGF/ROBO2/SEMA3D/CNTN1/CNR1/SEMA6D/DPYSL3/TNR/DDR2/FAT3/SEMA6A/NTRK2/CX3CL1/SEMA3G	16
BP	GO:0045089	positive regulation of innate immune response	12/340	300/18614	0.0094174	0.061772	0.0469034	NAIP/CCL5/NFKBIZ/TRIL/NLRC4/PYHIN1/RASGRP1/TNIP3/IRF1/FPR2/CLEC4E/GBP5	12
BP	GO:0002821	positive regulation of adaptive immune	7/340	129/18614	0.0095632	0.0625132	0.0474662	CD1C/SKAP1/NFKBIZ/CD1E/IL1B/SLC11A1/IL6	7

		response							
BP	GO:0032874	positive regulation of stress-activated MAPK cascade	7/340	129/18614	0.0095632	0.06251 32	0.04746 62	NAIP/TGFB2/SERPINF2/IL1B/CD27/FCGR2B/RASGRP1	7
BP	GO:0006633	fatty acid biosynthetic process	8/340	161/18614	0.00961 14	0.06263 29	0.04755 7	TRIB3/PTGS2/PTGDS/HPGDS/FASN/IL1B/AKR1C3/ALOX15B	8
BP	GO:1903317	regulation of protein maturation	5/340	71/18614	0.00961 43	0.06263 29	0.04755 7	RPS6KA2/ENO1/SERPINF2/NLRC4/IL1R2	5
BP	GO:0007622	rhythmic behavior	4/340	46/18614	0.00976 24	0.06327 32	0.04804 32	PTGDS/EGR1/EGR2/NR1D2	4

BP	GO:0044788	modulation by host of viral process	4/340	46/18614	0.0097624	0.0632732	0.0480432	FASN/ZDHHC9/PTX3/CCL8	4
BP	GO:2001239	regulation of extrinsic apoptotic signaling pathway in absence of ligand	4/340	46/18614	0.0097624	0.0632732	0.0480432	SRPX/IL1B/INHBA/CX3CL1	4
BP	GO:0045621	positive regulation of lymphocyte differentiation	7/340	130/18614	0.0095541	0.0642966	0.0488203	EGR3/NFKBIZ/IL2RG/CD83/CD27/RHOH/RASGRP1	7

BP	GO:0050864	regulation of B cell activation	7/340	130/18614	0.0099541	0.06429 66	0.04882 03	NFATC2/IL2RG/HMGB3/CD27/FCGR2B/INHBA/IL6	7
BP	GO:0060411	cardiac septum morphogenesis	5/340	72/18614	0.01018 13	0.06565 31	0.04985 02	BMP5/ROBO2/TGFB2/APLNR/SLIT3	5
BP	GO:0003181	atrioventricular valve morphogenesis	3/340	25/18614	0.01031 49	0.06606 73	0.05016 48	OLFM1/TGFB2/SLIT3	3
BP	GO:0034114	regulation of heterotypic cell-cell adhesion	3/340	25/18614	0.01031 49	0.06606 73	0.05016 48	SKAP1/IL1B/FGA	3
BP	GO:0046697	decidualization	3/340	25/18614	0.01031 49	0.06606 73	0.05016 48	PTGS2/JUNB/TPPP3	3

BP	GO:1904996	positive regulation of leukocyte adhesion to vascular endothelial cell	3/340	25/18614	0.01031 49	0.06606 73	0.05016 48	ITGA4/IL6/SELE	3
BP	GO:0050868	negative regulation of T cell activation	7/340	131/18614	0.01035 65	0.06611 17	0.05019 85	TIGIT/LAX1/VSIG4/FCGR2B/IRF1/PLA2G2A/IDO1	7
BP	GO:0070304	positive regulation of stress-activated protein	7/340	131/18614	0.01035 65	0.06611 17	0.05019 85	NAIP/TGFB2/SERPINF2/IL1B/CD27/FCGR2B/RASGRP1	7

		kinase signaling cascade							
BP	GO:0050678	regulation of epithelial cell proliferation	15/340	419/18614	0.0105037	0.0667108	0.0506534	CXCL12/BMP5/TGFB2/JUN/APLNR/ITGA4/EGR3/HMOX1/ZFP36/NR4A3/SULF1/SFN/NR4A1/CCL2/HAS2	15
BP	GO:0002701	negative regulation of production of molecular mediator of immune response	4/340	47/18614	0.0105204	0.0667108	0.0506534	TGFB2/CD96/HMOX1/FCGR2B	4

BP	GO:0002920	regulation of humoral immune response	4/340	47/18614	0.0105204	0.0667108	0.0506534	PGC/IL1B/VSIG4/FCGR2B	4
BP	GO:0031294	lymphocyte costimulation	4/340	47/18614	0.0105204	0.0667108	0.0506534	DPP4/ICOS/CD5/CD3E	4
BP	GO:0046879	hormone secretion	12/340	305/18614	0.0106413	0.0673655	0.0511505	P2RY1/SLC18A2/LTBP4/CNR1/CCL5/IL1B/ANO1/CPE/INHBA/CFTR/IL6/FGA	12
BP	GO:0007519	skeletal muscle tissue development	8/340	165/18614	0.011042	0.0697263	0.0529431	BTG2/EGR1/EGR2/HLF/NR1D2/ATF3/FOS/NR4A1	8
BP	GO:0008585	female gonad development	6/340	102/18614	0.0110508	0.0697263	0.0529431	ARID5B/ROBO2/IDH1/SLIT3/INHBA/PTX3	6

BP	GO:0060560	developmental growth involved in morphogenesis	10/340	234/18614	0.011121299	0.069993838	0.053141461	OLFM1/CXCL12/SEMA3D/ITGA4/SEMA6D/TNR/SLIT3/FMN1/SEMA6A/SEMA3G	10
BP	GO:2000116	regulation of cysteine-type endopeptidase activity	10/340	234/18614	0.011121299	0.069993838	0.053141461	HGF/PTGS2/NAIP/NLRC4/UACA/SFN/CD27/S100A8/TNFSF10/NR4A1	10
BP	GO:0051346	negative regulation of hydrolase activity	13/340	345/18614	0.011242465	0.070153636	0.053266275	HGF/PTGS2/NAIP/TGFB2/TIMP4/SERPINF2/SERPINA3/SLC39A14/SFN/CD27/PI3/NR4A1/PTX3	13
BP	GO:0007520	myoblast fusion	4/340	48/18614	0.011131154	0.070153636	0.053266275	NFATC2/ADAMTS15/CXCL10/CXCL9	4

BP	GO:0014075	response to amine	4/340	48/18614	0.00113154	0.00701536	0.00532675	SLC18A2/NQO1/NR4A1/NR4A2	4
BP	GO:0032309	icosanoid secretion	4/340	48/18614	0.00113154	0.00701536	0.00532675	PTGS2/ACE/IL1B/PLA2G2A	4
BP	GO:0035094	response to nicotine	4/340	48/18614	0.00113154	0.00701536	0.00532675	CNR1/HMOX1/SLC7A11/VCAM1	4
BP	GO:0036230	granulocyte activation	4/340	48/18614	0.00113154	0.00701536	0.00532675	CD177/CCL5/FCGR2B/PLA2G2A	4
BP	GO:0043300	regulation of leukocyte degranulation	4/340	48/18614	0.00113154	0.00701536	0.00532675	CD177/HMOX1/FGR/FCGR2B	4
BP	GO:2000404	regulation of T cell	4/340	48/18614	0.00113154	0.00701536	0.00532675	CXCL12/ITGA4/CCL5/CXCL10	4

		migration							
BP	GO:0035924	cellular response to vascular endothelial growth factor stimulus	5/340	74/18614	0.01138832	0.07015536	0.05326275	EGR3/VCAM1/DCN/SEMA6A/NR4A1	5
BP	GO:0003205	cardiac chamber development	8/340	166/18614	0.0114423	0.07015536	0.05326275	RYR2/BMP5/ROBO2/ANK2/TGFB2/APLNR/SLIT3/CPE	8
BP	GO:0071375	cellular response to peptide hormone stimulus	12/340	308/18614	0.01143426	0.07015536	0.05326275	TRIB3/SCNN1G/DDR2/SLC39A14/RARRES2/IL1B/NR4A3/FOS/INHBA/NR4A1/SOCS3/NR4A2	12

BP	GO:0010001	glial cell differentiation	10/340	235/18614	0.0114428	0.0701536	0.0532675	PHGDH/CNTN1/EGR2/CSPG5/IL1B/ANO1/S100A8/IL6/NTRK2/MT1X	10
BP	GO:0010882	regulation of cardiac muscle contraction by calcium ion signaling	3/340	26/18614	0.0115051	0.0701536	0.0532675	RYR2/ANK2/CASQ2	3
BP	GO:0031639	plasminogen activation	3/340	26/18614	0.0115051	0.0701536	0.0532675	ENO1/SERPINF2/FGA	3
BP	GO:0033622	integrin activation	3/340	26/18614	0.0115051	0.0701536	0.0532675	CXCL12/SKAP1/CX3CL1	3
BP	GO:0046629	gamma-delta T cell	3/340	26/18614	0.0115051	0.0701536	0.0532675	EGR3/CD3G/CD3E	3

BP	GO:0050995	negative regulation of lipid catabolic process	3/340	26/18614	0.011505051	0.07015536	0.05326275	PIK3CG/CNR1/IL1B	3
BP	GO:0051238	sequestering of metal ion	3/340	26/18614	0.011505051	0.07015536	0.05326275	CASQ2/LCN2/S100A8	3
BP	GO:1901623	regulation of lymphocyte chemotaxis	3/340	26/18614	0.011505051	0.07015536	0.05326275	CCL5/CCL2/CXCL10	3
BP	GO:0042100	B cell proliferation	6/340	103/18614	0.011555596	0.07037732	0.05334342	NFATC2/NFKBIZ/MS4A1/CD79A/FCGR2B/RASGRP1	6

BP	GO:0010466	negative regulation of peptidase activity	10/340	236/18614	0.011767623	0.071493733	0.054282847	HGF/PTGS2/NAIP/TIMP4/SERPINF2/SERPINA3/SFN/CD27/PI3/NR4A1	10
BP	GO:0001935	endothelial cell proliferation	9/340	201/18614	0.011816864	0.071593535	0.054363608	CXCL12/JUN/APLNR/ITGA4/EGR3/HMOX1/SULF1/NR4A1/CCL2	9
BP	GO:0007626	locomotory behavior	9/340	201/18614	0.011816864	0.071593535	0.054363608	SLC18A2/CXCL12/EGR1/CNTN1/HPGDS/TNR/DPP4/NR4A2/IDO1	9
BP	GO:0051090	regulation of DNA-binding transcription factor activity	16/340	465/18614	0.011906061	0.072020226	0.054686866	RTKN2/ARID5B/DDR2/JUND/NLRC4/HMOX1/CAPN3/IL1B/S100A8/CTH/TRIB1/IL6/S100A12/CX3CR1/CX3CL1/CSF3	16

BP	GO:0051403	stress-activated MAPK cascade	10/340	237/18614	0.0120886	0.0730102	0.0554365	HGF/NAIP/TGFB2/SERPINF2/ZFP36/IL1B/CD27/FCGR2B/RASGRP1/TRIB1	10
BP	GO:0010043	response to zinc ion	4/340	49/18614	0.012148	0.0732532	0.055621	VCAM1/S100A8/MT1X/MT1M	4
BP	GO:0046661	male sex differentiation	8/340	168/18614	0.0122139	0.0735345	0.0558346	ARID5B/BMP5/MAMLD1/TGFB2/ACE/AKR1C3/INHBA/TNFSF10	8
BP	GO:0007409	axonogenesis	15/340	427/18614	0.0123163	0.0740337	0.0562136	OLFM1/CXCL12/ROBO2/SEMA3D/CNTN1/ITGA4/EGR2/SEMA6D/TNR/SLIT3/NRXN3/SEMA6A/NTRK2/SEMA3G/NR4A2	15
BP	GO:0006766	vitamin metabolic process	6/340	105/18614	0.0126263	0.0756586	0.0574474	PSAT1/SHMT2/ALPL/MTHFD2/NQO1/AKR1C3	6
BP	GO:00	positive regulation of T	6/340	105/18614	0.012	0.075	0.057	HLA-DPA1/CCL5/IL1B/VCAM1/CD3E/IL6	6

	42102	cell proliferation			6263	6586	4474		
BP	GO:0010818	T cell chemotaxis	3/340	27/18614	0.0127711	0.0756934	0.0574738	PIK3CG/CCL5/CXCL10	3
BP	GO:0032967	positive regulation of collagen biosynthetic process	3/340	27/18614	0.0127711	0.0756934	0.0574738	SERPINF2/DDR2/INHBA	3
BP	GO:0033081	regulation of T cell differentiation in thymus	3/340	27/18614	0.0127711	0.0756934	0.0574738	EGR3/IL2RG/RASGRP1	3
BP	GO:00	thyroid hormone	3/340	27/18614	0.012	0.075	0.057	DUOX1/DUOXA1/SULT1B1	3

	42403	metabolic process			7711	6934	4738		
BP	GO:0042537	benzene-containing compound metabolic process	3/340	27/18614	0.0127711	0.0756934	0.0574738	FAH/EPHX2/IDO1	3
BP	GO:0060259	regulation of feeding behavior	3/340	27/18614	0.0127711	0.0756934	0.0574738	SGIP1/CNR1/NR4A3	3
BP	GO:0060384	innervation	3/340	27/18614	0.0127711	0.0756934	0.0574738	ITGA4/SULF1/VCAM1	3
BP	GO:00	learning or	11/340	276/18614	0.012	0.076	0.058	BTG2/PTGS2/CNR1/EGR2/TNR/SLC7A11/FOS/NRXN3/NTRK2/CX3CR1/SLC6A4	11

	07611	memory			9483	6244	1807		
BP	GO:0046545	development of primary female sexual characteristics	6/340	106/18614	0.0131848	0.0779026	0.0591513	ARID5B/ROBO2/IDH1/SLIT3/INHBA/PTX3	6
BP	GO:0022408	negative regulation of cell-cell adhesion	9/340	205/18614	0.0133026	0.0784573	0.0595724	CXCL12/TIGIT/TNR/LAX1/VSIG4/FCGR2B/IRF1/PLA2G2A/IDO1	9
BP	GO:2000514	regulation of CD4-positive, alpha-beta T cell activation	5/340	77/18614	0.0133616	0.0784573	0.0595724	NFKBIZ/JUNB/IL2RG/CD83/CD3E	5

BP	GO:0002002	regulation of angiotensin levels in blood	2/340	10/18614	0.01358575	0.07845573	0.05957724	CPA3/ACE	2
BP	GO:0002676	regulation of chronic inflammatory response	2/340	10/18614	0.01358575	0.07845573	0.05957724	CCL5/IDO1	2
BP	GO:0006828	manganese ion transport	2/340	10/18614	0.01358575	0.07845573	0.05957724	SLC39A14/SLC11A1	2
BP	GO:0030223	neutrophil differentiation	2/340	10/18614	0.01358575	0.07845573	0.05957724	FASN/TRIB1	2
BP	GO:00	polyketide metabo	2/340	10/18614	0.013	0.078	0.059	AKR1C2/AKR1C3	2

	30638	lic process			5875	4573	5724		
BP	GO:0030647	aminoglycoside antibiotic metabolic process	2/340	10/18614	0.0135875	0.07845573	0.05957724	AKR1C2/AKR1C3	2
BP	GO:0036093	germ cell proliferation	2/340	10/18614	0.0135875	0.07845573	0.05957724	PTGDS/HPGDS	2
BP	GO:0044598	doxorubicin metabolic process	2/340	10/18614	0.0135875	0.07845573	0.05957724	AKR1C2/AKR1C3	2
BP	GO:0048251	elastic fiber assembly	2/340	10/18614	0.0135875	0.07845573	0.05957724	MFAP4/MYH11	2

BP	GO:0070099	regulation of chemokine-mediated signaling pathway	2/340	10/18614	0.0135875	0.0784573	0.0595724	CCL5/SLIT3	2
BP	GO:0071421	manganese ion transmembrane transport	2/340	10/18614	0.0135875	0.0784573	0.0595724	SLC39A14/SLC11A1	2
BP	GO:0097050	type B pancreatic cell apoptotic process	2/340	10/18614	0.0135875	0.0784573	0.0595724	RYR2/IL6	2

BP	GO:2000659	regulation of interleukin-1-mediated signaling pathway	2/340	10/18614	0.013587 5	0.078457 3	0.059572 4	IL1R2/IL6	2
BP	GO:0003279	cardiac septum development	6/340	107/18614	0.013760 3	0.079215 3	0.060148	BMP5/ROBO2/ANK2/TGFB2/APLNR/SLIT3	6
BP	GO:0062014	negative regulation of small molecule metabolic process	6/340	107/18614	0.013760 3	0.079215 3	0.060148	TRIB3/BMP5/CNR1/IER3/AKR1C3/DDIT4	6

BP	GO:0032964	collagen biosynthetic process	4/340	51/18614	0.013928 6	0.079822 3	0.060608 9	SERPINF2/DDR2/INHBA/IL6	4
BP	GO:1902895	positive regulation of miRNA transcription	4/340	51/18614	0.013928 6	0.079822 3	0.060608 9	TGFB2/EGR1/JUN/FOS	4
BP	GO:2001238	positive regulation of extrinsic apoptotic signaling pathway	4/340	51/18614	0.013928 6	0.079822 3	0.060608 9	SRPX/ATF3/INHBA/TNFSF10	4
BP	GO:00	inflammatory respon	5/340	78/18614	0.014	0.080	0.06113	CNR1/CYSLTR1/FGR/FCGR2B/RASGRP1	5

	02437	se to antigenic stimulus			0695	5086			
BP	GO:0010714	positive regulation of collagen metabolic process	3/340	28/18614	0.0141137	0.0805197	0.0611385	SERPINF2/DDR2/INHBA	3
BP	GO:0010758	regulation of macrophage chemotaxis	3/340	28/18614	0.0141137	0.0805197	0.0611385	CCL5/STAP1/RARRES2	3
BP	GO:0009064	glutamine family amino acid metabo	5/340	79/18614	0.0148021	0.0840696	0.0638338	FAH/PYCR1/GCLM/PHGDH/SLC7A11	5

		lic process							
BP	GO:0042310	vasoconstriction	5/340	79/18614	0.0148021	0.0840696	0.0638338	PTGS2/HRH2/ACE/FGA/SLC6A4	5
BP	GO:1903201	regulation of oxidative stress-induced cell death	5/340	79/18614	0.0148021	0.0840696	0.0638338	PYCR1/HGF/DDR2/SLC7A11/NR4A3	5
BP	GO:0003197	endocardial cushion development	4/340	52/18614	0.0148776	0.0841227	0.0638742	BMP5/ROBO2/TGFB2/APLNR	4
BP	GO:0006968	cellular defense response	4/340	52/18614	0.0148776	0.0841227	0.0638742	TRAT1/KLRG1/CX3CR1/CXCL9	4

BP	GO:0031103	axon regeneration	4/340	52/18614	0.014877 6	0.084122 7	0.063874 2	JUN/CSPG5/TNR/TNC	4
BP	GO:0031098	stress-activated protein kinase signaling cascade	10/340	245/18614	0.014949 7	0.084233 6	0.063958 4	HGF/NAIP/TGFB2/SERPINF2/ZFP36/IL1B/CD27/FCGR2B/RASGRP1/TRIB1	10
BP	GO:0045833	negative regulation of lipid metabolic process	6/340	109/18614	0.014963 6	0.084233 6	0.063958 4	TRIB3/BMP5/PIK3CG/CNR1/IL1B/AKR1C3	6
BP	GO:1902106	negative regulation of	6/340	109/18614	0.014963 6	0.084233 6	0.063958 4	HMGB3/TNFRSF11B/FCGR2B/TRIB1/IRF1/INHBA	6

		leukocyte differentiation							
BP	GO:0001562	response to protozoan	3/340	29/18614	0.0155332	0.0869989	0.0660581	IER3/SLC11A1/GBP4	3
BP	GO:0015732	prostaglandin transport	3/340	29/18614	0.0155332	0.0869989	0.0660581	PTGS2/SLCO4A1/IL1B	3
BP	GO:0002292	T cell differentiation involved in immune response	5/340	80/18614	0.0155598	0.0869989	0.0660581	NFKBIZ/JUNB/LY9/IL6/CLEC4E	5
BP	GO:0070227	lymphocyte apoptotic	5/340	80/18614	0.0155598	0.0869989	0.0660581	CCL5/NFKBIZ/CD27/CD3G/IDO1	5

		process							
BP	GO:0009408	response to heat	6/340	110/18614	0.01559518	0.08699989	0.06660581	CXCL12/PTGS2/HMOX1/ANO1/TFEC/CXCL10	6
BP	GO:0031341	regulation of cell killing	6/340	110/18614	0.01559518	0.08699989	0.06660581	CD1C/STAP1/CD1E/FCGR2B/RASGRP1/CX3CR1	6
BP	GO:0060047	heart contraction	10/340	247/18614	0.01573779	0.08768858	0.06665796	CORIN/RPS6KA2/RYR2/FXYD1/ANK2/TGFB2/PIK3CG/TRPC1/CASQ2/ACE	10
BP	GO:0010977	negative regulation of neuron projection development	7/340	143/18614	0.01615222	0.0897322	0.06681333	SEMA3D/SEMA6D/DPYSL3/TNR/FAT3/SEMA6A/SEMA3G	7
BP	GO:00	positive regulation of	7/340	143/18614	0.016	0.089	0.068	CXCL12/CCL5/RARRES2/IL6/FPR2/CX3CR1/CXCL10	7

	50921	chemotaxis			1522	732	1333		
BP	GO:0002604	regulation of dendritic cell antigen processing and presentation	2/340	11/18614	0.0164083	0.0899739	0.068317	FCGR2B/SLC11A1	2
BP	GO:0019065	receptor-mediated endocytosis of virus by host cell	2/340	11/18614	0.0164083	0.0899739	0.068317	DPP4/SIGLEC1	2
BP	GO:0032610	interleukin-1 alpha production	2/340	11/18614	0.0164083	0.0899739	0.068317	IL1R2/CX3CL1	2

BP	GO:0032650	regulation of interleukin-1 alpha production	2/340	11/18614	0.01640 83	0.08997 39	0.06831 7	IL1R2/CX3CL1	2
BP	GO:0043084	penile erection	2/340	11/18614	0.01640 83	0.08997 39	0.06831 7	P2RY1/CNR1	2
BP	GO:0071313	cellular response to caffeine	2/340	11/18614	0.01640 83	0.08997 39	0.06831 7	RYR2/CASQ2	2
BP	GO:0071918	urea transmembrane transport	2/340	11/18614	0.01640 83	0.08997 39	0.06831 7	SLC14A1/AQP9	2
BP	GO:2000253	positive regulation of feeding	2/340	11/18614	0.01640 83	0.08997 39	0.06831 7	SGIP1/NR4A3	2

BP	GO:2001241	behavior or positive regulation of extrinsic apoptotic signaling pathway in absence of ligand	2/340	11/18614	0.0164083	0.0899739	0.0683317	SRPX/INHBA	2
BP	GO:0008584	male gonad development	7/340	144/18614	0.0167212	0.0915558	0.0695198	ARID5B/MAMLD1/TGFB2/ACE/AKR1C3/INHBA/TNFSF10	7
BP	GO:0060538	skeletal muscle organ development	8/340	178/18614	0.0167836	0.0917681	0.0696793	BTG2/EGR1/EGR2/HLF/NR1D2/ATF3/FOS/NR4A1	8

BP	GO:0038093	Fc receptor signaling pathway	4/340	54/18614	0.01689 59	0.0921176	0.0699447	FGR/NR4A3/FCGR2B/CLEC4E	4
BP	GO:1904645	response to amyloid-beta	4/340	54/18614	0.0168959	0.0921176	0.0699447	ITGA4/VCAM1/FCGR2B/FPR2	4
BP	GO:0002507	tolerance induction	3/340	30/18614	0.0170303	0.0924538	0.0702	ICOS/CD3E/IDO1	3
BP	GO:0034505	tooth mineralization	3/340	30/18614	0.0170303	0.0924538	0.0702	ALPL/CFTR/ASPN	3
BP	GO:0060142	regulation of syncytium formation by plasma	3/340	30/18614	0.0170303	0.0924538	0.0702	NFATC2/CXCL10/CXCL9	3

BP	GO:0046546	development of primary male sexual characteristics	7/340	145/18614	0.0173041	0.0936734	0.0711266	ARID5B/MAMLD1/TGFB2/ACE/AKR1C3/INHBA/TNFSF10	7
BP	GO:0050671	positive regulation of lymphocyte proliferation	7/340	145/18614	0.0173041	0.0936734	0.0711266	HLA-DPA1/NFATC2/CCL5/IL1B/VCAM1/CD3E/IL6	7
BP	GO:0009063	amino acid catabolic process	6/340	113/18614	0.0175855	0.0950617	0.0721801	FAH/SHMT2/CBS/HMGCLL1/HDC/IDO1	6

BP	GO:0006584	catecholamine metabolic process	4/340	55/18614	0.0179666	0.0965703	0.0733257	TGFB2/HDC/MAOA/NR4A2	4
BP	GO:0009712	catechol-containing compound metabolic process	4/340	55/18614	0.0179666	0.0965703	0.0733257	TGFB2/HDC/MAOA/NR4A2	4
BP	GO:0043370	regulation of CD4-positive, alpha-beta T cell differentiation	4/340	55/18614	0.0179666	0.0965703	0.0733257	NFKBIZ/JUNB/IL2RG/CD83	4

BP	GO:0050805	negative regulation of synaptic transmission	4/340	55/18614	0.0179666	0.0965703	0.0733257	PTGS2/TNR/IL1B/SLC6A4	4
BP	GO:0046634	regulation of alpha-beta T cell activation	6/340	114/18614	0.0182873	0.0981588	0.0745318	NFKBIZ/JUNB/IL2RG/CD83/IRF1/CD3E	6
BP	GO:0010880	regulation of release of sequestered calcium ion into cytosol by sarcopl	3/340	31/18614	0.0186053	0.0995852	0.0756148	RYR2/ANK2/CASQ2	3

		asmic reticulum							
BP	GO:0070293	renal absorption	3/340	31/18614	0.0186053	0.0995852	0.0756148	HBB/AKR1C3/HAS2	3
BP	GO:1903707	negative regulation of hemopoiesis	6/340	115/18614	0.0190079	0.1004635	0.0762818	HMGB3/TNFRSF11B/FCGR2B/TRIB1/IRF1/INHBA	6
BP	GO:0018108	peptidyl-tyrosine phosphorylation	13/340	370/18614	0.0190087	0.1004635	0.0762818	HGF/PKDCC/WEE1/CNTN1/CCL5/DDR2/ACE/FGR/CD3E/IL6/NTRK2/SOCS3/CSF3	13
BP	GO:0001954	positive regulation of cell-matrix	4/340	56/18614	0.0190773	0.1004635	0.0762818	SKAP1/FMN1/CD3E/CX3CL1	4

		adhesion							
BP	GO:0019369	arachidonic acid metabolic process	4/340	56/18614	0.0190773	0.1004635	0.0762818	PTGS2/PTGDS/AKR1C3/ALOX15B	4
BP	GO:0055078	sodium ion homeostasis	4/340	56/18614	0.0190773	0.1004635	0.0762818	CORIN/SCNN1G/ACE/SLC1A3	4
BP	GO:0140962	multicellular organismal-level chemical homeostasis	4/340	56/18614	0.0190773	0.1004635	0.0762818	SCNN1G/CFTR/SLC11A1/HAS2	4
BP	GO:2000107	negative regulation of	4/340	56/18614	0.0190773	0.1004635	0.0762818	CXCL12/CCL5/CD27/IDO1	4

		leukocyte apoptotic process							
BP	GO:0002705	positive regulation of leukocyte mediated immunity	7/340	148/18614	0.01913389	0.10046635	0.07662818	CD1C/CD177/STAP1/CD1E/IL1B/RASGRP1/IL6	7
BP	GO:0032946	positive regulation of mononuclear cell proliferation	7/340	148/18614	0.01913389	0.10046635	0.07662818	HLA-DPA1/NFATC2/CCL5/IL1B/VCAM1/CD3E/IL6	7
BP	GO:00	I-kappaB kinase/	11/340	293/18614	0.019	0.100	0.076	PIM2/HMOX1/CAPN3/IL1B/RHOH/CTH/TNIP3/TNFSF10/S100A12/CX3CR1/CX3CL1	11

	07249	NF-kappaB signaling			3214	4635	2818		
BP	GO:0002645	positive regulation of tolerance induction	2/340	12/18614	0.01945448	0.10046635	0.07628818	CD3E/IDO1	2
BP	GO:0015840	urea transport	2/340	12/18614	0.01945448	0.10046635	0.07628818	SLC14A1/AQP9	2
BP	GO:0019336	phenol-containing compound catabolic process	2/340	12/18614	0.01945448	0.10046635	0.07628818	FAH/MAOA	2

BP	GO:0032252	secretory granule localization	2/340	12/18614	0.01945548	0.1004635	0.0762818	PIK3CG/RASGRP1	2
BP	GO:0032351	negative regulation of hormone metabolic process	2/340	12/18614	0.01945548	0.1004635	0.0762818	BMP5/AKR1C3	2
BP	GO:0032908	regulation of transforming growth factor beta1 production	2/340	12/18614	0.01945548	0.1004635	0.0762818	LUM/CX3CL1	2

BP	GO:0042416	dopamine biosynthetic process	2/340	12/18614	0.0194548	0.1004635	0.0762818	TGFB2/NR4A2	2
BP	GO:0043383	negative T cell selection	2/340	12/18614	0.0194548	0.1004635	0.0762818	THEMIS/CD3E	2
BP	GO:0051593	response to folic acid	2/340	12/18614	0.0194548	0.1004635	0.0762818	CBS/NFKBIZ	2
BP	GO:0070170	regulation of tooth mineralization	2/340	12/18614	0.0194548	0.1004635	0.0762818	CFTR/ASPN	2
BP	GO:0071372	cellular response to follicle-stimulating hormon	2/340	12/18614	0.0194548	0.1004635	0.0762818	GCLM/INHBA	2

		e stimulus							
BP	GO:0086015	SA node cell action potential	2/340	12/18614	0.0194548	0.1004635	0.0762818	RYR2/ANK2	2
BP	GO:0086018	SA node cell to atrial cardiac muscle cell signaling	2/340	12/18614	0.0194548	0.1004635	0.0762818	RYR2/ANK2	2
BP	GO:1901731	positive regulation of platelet aggregation	2/340	12/18614	0.0194548	0.1004635	0.0762818	MMRN1/IL6	2

BP	GO:1903238	positive regulation of leukocyte tethering or rolling	2/340	12/18614	0.0194548	0.1004635	0.0762818	ITGA4/SELE	2
BP	GO:1903977	positive regulation of glial cell migration	2/340	12/18614	0.0194548	0.1004635	0.0762818	CX3CR1/CX3CL1	2
BP	GO:0003012	muscle system process	15/340	452/18614	0.0195892	0.1010211	0.0767051	P2RY1/RYR2/ENO1/FXYD1/GTF2IRD1/PTGS2/ANK2/PIK3CG/CASQ2/HMOX1/IL1B/NR4A3/SULF1/MYH11/NR4A1	15
BP	GO:0072524	pyridine-containing compound metabo	5/340	85/18614	0.0197369	0.1014057	0.0769971	PSAT1/IDH1/ALPL/NQO1/IDO1	5

		lic process							
BP	GO:0015908	fatty acid transport	6/340	116/18614	0.019747478	0.101404057	0.076999971	PLIN2/PTGS2/SLCO4A1/ACE/IL1B/PLA2G2A	6
BP	GO:0048640	negative regulation of developmental growth	6/340	116/18614	0.019747478	0.101404057	0.076999971	SEMA3D/SEMA6D/TNR/SEMA6A/SEMA3G/SLC6A4	6
BP	GO:0018212	peptidyl-tyrosine modification	13/340	372/18614	0.019777702	0.101404057	0.076999971	HGF/PKDCC/WEE1/CNTN1/CCL5/DDR2/ACE/FGR/CD3E/IL6/NTRK2/SOCS3/CSF3	13
BP	GO:0072330	monocarboxylic acid biosynthetic	9/340	220/18614	0.020121279	0.102665653	0.077953536	TRIB3/PTGS2/PTGDS/HPGDS/FASN/IL1B/AKR1C3/ALOX15B/IDO1	9

		process							
BP	GO:0006826	iron ion transport	4/340	57/18614	0.0202304	0.1026653	0.07795336	SLC39A14/LCN2/SLC11A1/CP	4
BP	GO:0072132	mesenchyme morphogenesis	4/340	57/18614	0.0202304	0.1026653	0.07795336	BMP5/ROBO2/TGFB2/APLNR	4
BP	GO:0001975	response to amphetamine	3/340	32/18614	0.02025583	0.1026653	0.07795336	SLC18A2/NR4A1/NR4A2	3
BP	GO:0002313	mature B cell differentiation involved in immune response	3/340	32/18614	0.02025583	0.10266553	0.07795336	NFKBIZ/FCGR2B/IL6	3

BP	GO:0002431	Fc receptor mediated stimulatory signaling pathway	3/340	32/18614	0.02025 83	0.10266 53	0.07795 36	FGR/NR4A3/FCGR2B	3
BP	GO:0033198	response to ATP	3/340	32/18614	0.02025 83	0.10266 53	0.07795 36	P2RY1/PTGS2/IL1B	3
BP	GO:0046685	response to arsenic-containing substance	3/340	32/18614	0.02025 83	0.10266 53	0.07795 36	TNFRSF11B/HMOX1/ATF3	3
BP	GO:00	multicellular organis	3/340	32/18614	0.020	0.102	0.077	SCNN1G/CFTR/HAS2	3

	50891	mal-level water homeostasis			2583	6653	9536		
BP	GO:0043122	regulation of I-kappaB kinase/NF-kappaB signaling	10/340	258/18614	0.0206359	0.10397	0.0789442	PIM2/HMOX1/CAPN3/IL1B/RHOH/CTH/TNIP3/TNFSF10/S100A12/CX3CL1	10
BP	GO:0032640	tumor necrosis factor production	8/340	185/18614	0.0206415	0.10397	0.0789442	ZBTB20/ORM1/ZFP36/RASGRP1/CD2/IL6/CX3CR1/CX3CL1	8
BP	GO:0032680	regulation of tumor necrosis factor production	8/340	185/18614	0.0206415	0.10397	0.0789442	ZBTB20/ORM1/ZFP36/RASGRP1/CD2/IL6/CX3CR1/CX3CL1	8

BP	GO:0008625	extrinsic apoptotic signaling pathway via death domain receptors	5/340	86/18614	0.020206521	0.10397	0.0789442	BMP5/HGF/HMOX1/ATF3/FGA	5
BP	GO:0015844	monoamine transport	5/340	86/18614	0.020206521	0.10397	0.0789442	P2RY1/SLC18A2/CXCL12/CNR1/SLC6A4	5
BP	GO:0003015	heart process	10/340	259/18614	0.021211307	0.1060991	0.0805608	CORIN/RPS6KA2/RYR2/FXYD1/ANK2/TGFB2/PIK3CG/TRPC1/CASQ2/ACE	10
BP	GO:0015833	peptide transport	10/340	259/18614	0.021211307	0.1060991	0.0805608	CNR1/CCL5/SLC7A11/IL1B/ANO1/S100A8/CPE/CFTR/IL6/FGA	10

BP	GO:0051897	positive regulation of protein kinase B signaling	6/340	118/18614	0.0212857	0.1067366	0.0810448	AKR1C2/PIK3CG/AKR1C3/CX3CR1/CX3CL1/CSF3	6
BP	GO:0001755	neural crest cell migration	4/340	58/18614	0.0214254	0.1070146	0.0812559	SEMA3D/SEMA6D/SEMA6A/SEMA3G	4
BP	GO:0006767	water-soluble vitamin metabolic process	4/340	58/18614	0.0214254	0.1070146	0.0812559	PSAT1/SHMT2/ALPL/MTHFD2	4
BP	GO:0010718	positive regulation of epithelial to mesen	4/340	58/18614	0.0214254	0.1070146	0.0812559	OLFM1/TGFB2/IL1B/IL6	4

		chymal transition							
BP	GO:0048678	response to axon injury	5/340	87/18614	0.0215947	0.10771791	0.08179909	JUN/CSPG5/DPYSL3/TNR/TNC	5
BP	GO:0007612	learning	7/340	152/18614	0.02179716	0.1084173	0.0823321	BTG2/PTGS2/TNR/SLC7A11/FOS/NRXN3/NTRK2	7
BP	GO:0071322	cellular response to carbohydrate stimulus	7/340	152/18614	0.02179716	0.1084173	0.0823321	ZBTB20/RPS6KA2/GCLM/ACE/SLC39A14/ANO1/CFTR	7
BP	GO:0021591	ventricular system development	3/340	33/18614	0.02198895	0.10855519	0.08242432	SEMA6D/SLC7A11/DNAH5	3
BP	GO:00	Fc-gamma recepto	3/340	33/18614	0.021	0.108	0.082	FGR/FCGR2B/CLEC4E	3

	38094	r signaling pathway			9895	5519	4232		
BP	GO:0048566	embryonic digestive tract development	3/340	33/18614	0.0219895	0.1085519	0.0824232	TGFB2/PKDCC/RARRES2	3
BP	GO:0090279	regulation of calcium ion import	3/340	33/18614	0.0219895	0.1085519	0.0824232	CXCL12/ACE/CCL2	3
BP	GO:1901889	negative regulation of cell junction assembly	3/340	33/18614	0.0219895	0.1085519	0.0824232	ROBO2/ACE/IL1B	3

BP	GO:1990776	response to angiotensin	3/340	33/18614	0.02198 95	0.1085519	0.0824232	PTGS2/DDR2/INHBA	3
BP	GO:0031668	cellular response to extracellular stimulus	10/340	261/18614	0.0221462	0.1089027	0.0826896	PLIN2/CBS/ITGA4/HMOX1/AKR1C3/VCAM1/ATF3/FOS/NR4A2/POSTN	10
BP	GO:0007596	blood coagulation	9/340	224/18614	0.0223114	0.1089027	0.0826896	P2RY1/HBB/PIK3CG/MMRN1/SERPINF2/SLC7A11/SAA1/IL6/FGA	9
BP	GO:0021675	nerve development	5/340	88/18614	0.0225648	0.1089027	0.0826896	ITGA4/EGR2/SULF1/VCAM1/SLC1A3	5
BP	GO:0010574	regulation of vascular endothelial growth	4/340	59/18614	0.0226628	0.1089027	0.0826896	PTGS2/IL1B/SULF1/IL6	4

		factor production							
BP	GO:0050432	catecholamine secretion	4/340	59/18614	0.02266628	0.1089027	0.08268896	P2RY1/SLC18A2/CXCL12/CNR1	4
BP	GO:0070228	regulation of lymphocyte apoptotic process	4/340	59/18614	0.02266628	0.1089027	0.08268896	CCL5/CD27/CD3G/IDO1	4
BP	GO:1903053	regulation of extracellular matrix organization	4/340	59/18614	0.02266628	0.1089027	0.08268896	DDR2/DPP4/IL6/HAS2	4
BP	GO:20	regulation of endoth	4/340	59/18614	0.022	0.108	0.082	ITGA4/AKR1C3/CCL2/FGA	4

	00351	elial cell apoptotic process			6628	9027	6896		
BP	GO:0003007	heart morphogenesis	10/340	262/18614	0.0226667	0.1089027	0.0826896	RYR2/OLFM1/BMP5/ROBO2/TGFB2/JUN/APLNR/SLIT3/CPE/HAS2	10
BP	GO:0002430	complement receptor mediated signaling pathway	2/340	13/18614	0.0227178	0.1089027	0.0826896	FPR1/FPR2	2
BP	GO:0002664	regulation of T cell tolerance	2/340	13/18614	0.0227178	0.1089027	0.0826896	CD3E/IDO1	2

		induction							
BP	GO:0005513	detection of calcium ion	2/340	13/18614	0.0227178	0.1089027	0.0826896	RYR2/CASQ2	2
BP	GO:0031392	regulation of prostaglandin biosynthetic process	2/340	13/18614	0.0227178	0.1089027	0.0826896	PTGS2/IL1B	2
BP	GO:0033604	negative regulation of catecholamine secretion	2/340	13/18614	0.0227178	0.1089027	0.0826896	P2RY1/CNR1	2
BP	GO:00	G protein-couple	2/340	13/18614	0.022	0.108	0.082	P2RY1/P2RY14	2

	35589	d purinergic nucleotide receptor signaling pathway			7178	9027	6896		
BP	GO:0051610	serotonin uptake	2/340	13/18614	0.02271778	0.1089027	0.0826896	SLC18A2/SLC6A4	2
BP	GO:0051775	response to redox state	2/340	13/18614	0.02271778	0.1089027	0.0826896	RYR2/SLC7A11	2
BP	GO:0060213	positive regulation of nuclear-transcribed	2/340	13/18614	0.02271778	0.1089027	0.0826896	BTG2/ZFP36	2

		mRNA poly(A) tail shortening							
BP	GO:0070234	positive regulation of T cell apoptotic process	2/340	13/18614	0.0227178	0.1089027	0.0826896	CCL5/IDO1	2
BP	GO:0071389	cellular response to mineralocorticoid stimulus	2/340	13/18614	0.0227178	0.1089027	0.0826896	SCNN1G/ACE	2
BP	GO:0072537	fibroblast activation	2/340	13/18614	0.0227178	0.1089027	0.0826896	GCLM/DDR2	2

BP	GO:0075509	endocytosis involved in viral entry into host cell	2/340	13/18614	0.02271 78	0.108902 7	0.082689 6	DPP4/SIGLEC1	2
BP	GO:1901298	regulation of hydrogen peroxide-mediated programmed cell death	2/340	13/18614	0.02271 78	0.108902 7	0.082689 6	HGF/DDR2	2
BP	GO:0097193	intrinsic apoptotic signaling	11/340	301/18614	0.02301 86	0.110206	0.083679 2	RTKN2/TRIB3/CXCL12/ENO1/HYOU1/PTGS2/HMOX1/IER3/SFN/S100A8/DDIT4	11

		pathway							
BP	GO:0055072	iron ion homeostasis	5/340	89/18614	0.0235627	0.1122502	0.0852313	SLC39A14/HMOX1/LCN2/SLC11A1/CP	5
BP	GO:0030282	bone mineralization	6/340	121/18614	0.0237414	0.1122502	0.0852313	PTGS2/PKDCC/DDR2/ALPL/FGR/ASPN	6
BP	GO:0002822	regulation of adaptive immune response based on somatic recombination of immune	8/340	190/18614	0.023757	0.1122502	0.0852313	CD1C/NFKBIZ/JUNB/CD1E/IL1B/FCGR2B/SLC11A1/IL6	8

		receptors built from immunoglobulin superfamily domains							
BP	GO:0071706	tumor necrosis factor superfamily cytokine production	8/340	190/18614	0.0237557	0.1122502	0.0852313	ZBTB20/ORM1/ZFP36/RASGRP1/CD2/IL6/CX3CR1/CX3CL1	8
BP	GO:1903555	regulation of tumor necrosis factor superfamily	8/340	190/18614	0.0237557	0.1122502	0.0852313	ZBTB20/ORM1/ZFP36/RASGRP1/CD2/IL6/CX3CR1/CX3CL1	8

		cytokine production							
BP	GO:0007431	salivary gland development	3/340	34/18614	0.0237999	0.1122502	0.0852313	HGF/TGFB2/TFCP2L1	3
BP	GO:0032743	positive regulation of interleukin-2 production	3/340	34/18614	0.0237999	0.1122502	0.0852313	CD83/IL1B/CD3E	3
BP	GO:0034405	response to fluid shear stress	3/340	34/18614	0.0237999	0.1122502	0.0852313	PTGS2/ACE/HAS2	3
BP	GO:0043372	positive regulation of CD4-positive, alpha-	3/340	34/18614	0.0237999	0.1122502	0.0852313	NFKBIZ/IL2RG/CD83	3

		beta T cell differentiation							
BP	GO:0050869	negative regulation of B cell activation	3/340	34/18614	0.023799	0.1122502	0.0852313	HMGB3/FCGR2B/INHBA	3
BP	GO:0051450	myoblast proliferation	3/340	34/18614	0.023799	0.1122502	0.0852313	HGF/FGR/FOS	3
BP	GO:0060317	cardiac epithelial to mesenchymal transition	3/340	34/18614	0.023799	0.1122502	0.0852313	OLFM1/TGFB2/HAS2	3
BP	GO:0019748	secondary metabolic	4/340	60/18614	0.0239428	0.1123721	0.0853239	AKR1C2/TRPC1/SLC7A11/AKR1C3	4

		process							
BP	GO:0046579	positive regulation of Ras protein signal transduction	4/340	60/18614	0.0239428	0.1123721	0.0853239	PIK3CG/GPR4/P2RY10/RASGRP1	4
BP	GO:0090497	mesenchymal cell migration	4/340	60/18614	0.0239428	0.1123721	0.0853239	SEMA3D/SEMA6D/SEMA6A/SEMA3G	4
BP	GO:2000630	positive regulation of miRNA metabolic process	4/340	60/18614	0.0239428	0.1123721	0.0853239	TGFB2/EGR1/JUN/FOS	4
BP	GO:00	negative regulati	13/340	383/18614	0.024	0.114	0.086	TRIB3/HGF/JUN/DDR2/STAP1/LAX1/IER3/IL1B/SFN/TRIB1/DDIT4/INHBA/SOCS3	13

	42326	on of phosphorylation			3753	2612	7583		
BP	GO:0050829	defense response to Gram-negative bacterium	5/340	90/18614	0.0245887	0.1147517	0.0871307	LYZ/NFKBIZ/RARRES2/SLC11A1/IL6	5
BP	GO:0060395	SMAD protein signal transduction	5/340	90/18614	0.0245887	0.1147517	0.0871307	BMP5/TGFB2/JUN/FOS/INHBA	5
BP	GO:0002698	negative regulation of immune effector	6/340	122/18614	0.0246004	0.1147517	0.0871307	TGFB2/CD96/HMOX1/VSIG4/FCGR2B/CX3CR1	6

		process							
BP	GO:0046660	female sex differentiation	6/340	122/18614	0.0246004	0.1147517	0.0871307	ARID5B/ROBO2/IDH1/SLIT3/INHBA/PTX3	6
BP	GO:0052547	regulation of peptidase activity	14/340	425/18614	0.0249784	0.1163724	0.0883613	HGF/PTGS2/NAIP/TIMP4/SERPINF2/SERPINA3/NLRC4/UACA/SFN/CD27/S100A8/TNFSF10/PI3/NR4A1	14
BP	GO:0019229	regulation of vasoconstriction	4/340	61/18614	0.0252659	0.1173161	0.0890779	PTGS2/HRH2/ACE/FGA	4
BP	GO:0048705	skeletal system morphogenesis	9/340	229/18614	0.0252733	0.1173161	0.0890779	FREM1/ARID5B/CBS/SPEF2/BMP1/ALPL/FMN1/FGR/HAS2	9
BP	GO:0050817	coagulation	9/340	229/18614	0.0252733	0.1173161	0.0890779	P2RY1/HBB/PIK3CG/MMRN1/SERPINF2/SLC7A11/SAA1/IL6/FGA	9

BP	GO:0030902	hindbrain development	7/340	157/18614	0.0254528	0.1179879	0.0895858	BMP5/CBS/CNTN1/EGR2/FCGR2B/CD3E/SLC6A4	7
BP	GO:0002824	positive regulation of adaptive immune response based on somatic recombination of immune receptors built from immunoglobuli	6/340	123/18614	0.0254799	0.1179879	0.0895858	CD1C/NFKBIZ/CD1E/IL1B/SLC11A1/IL6	6

		n superfamily domains							
BP	GO:0001893	maternal placenta development	3/340	35/18614	0.02568668	0.11808 48	0.08966615	PTGS2/JUNB/TPPP3	3
BP	GO:0002446	neutrophil mediated immunity	3/340	35/18614	0.02568668	0.11808 48	0.08966615	CD177/ACE/IL6	3
BP	GO:0002719	negative regulation of cytokine production involve	3/340	35/18614	0.02568668	0.11808 48	0.08966615	TGFB2/CD96/HMOX1	3

		d in immune response								
BP	GO:0014808	release of sequestered calcium ion into cytosol by sarcoplasmic reticulum	3/340	35/18614	0.0256868	0.11808 48	0.08966 15	RYR2/ANK2/CASQ2		3
BP	GO:0030212	hyaluronan metabolic process	3/340	35/18614	0.02568 68	0.11808 48	0.08966 15	IL1B/LYVE1/HAS2		3
BP	GO:00	positive regulation of	3/340	35/18614	0.025	0.118	0.089	PTGS2/TNR/CCL2		3

	51968	synaptic transmission, glutamatergic			6868	0848	6615		
BP	GO:0032872	regulation of stress-activated MAPK cascade	8/340	193/18614	0.0257774	0.118252	0.0897885	HGF/NAIP/TGFB2/SERPINF2/IL1B/CD27/FCGR2B/RASGRP1	8
BP	GO:0007599	hemostasis	9/340	230/18614	0.0258976	0.118252	0.0897885	P2RY1/HBB/PIK3CG/MMRN1/SERPINF2/SLC7A11/SAA1/IL6/FGA	9
BP	GO:0030213	hyaluronan biosynthetic process	2/340	14/18614	0.0261886	0.118252	0.0897885	IL1B/HAS2	2

BP	GO:0031000	response to caffeine	2/340	14/18614	0.02618186	0.1182252	0.08978785	RYR2/CASQ2	2
BP	GO:0033004	negative regulation of mast cell activation	2/340	14/18614	0.02618186	0.1182252	0.08978785	CNR1/HMOX1	2
BP	GO:0043301	negative regulation of leukocyte degranulation	2/340	14/18614	0.02618186	0.1182252	0.08978785	HMOX1/FCGR2B	2
BP	GO:0043922	negative regulation by host of viral	2/340	14/18614	0.02618186	0.1182252	0.08978785	JUN/CCL5	2

		transcription							
BP	GO:0044793	negative regulation by host of viral process	2/340	14/18614	0.02618886	0.1182522	0.08978785	PTX3/CCL8	2
BP	GO:0050667	homocysteine metabolic process	2/340	14/18614	0.02618886	0.1182522	0.08978785	CBS/CTH	2
BP	GO:0061029	eyelid development in camera-type eye	2/340	14/18614	0.02618886	0.1182522	0.08978785	JUN/INHBA	2
BP	GO:00	T-helper 17 cell	2/340	14/18614	0.026	0.118	0.089	LY9/IL6	2

	72540	lineage commitment			1886	252	7885		
BP	GO:0072672	neutrophil extravasation	2/340	14/18614	0.0261886	0.118252	0.0897885	PIK3CG/CD177	2
BP	GO:0086070	SA node cell to atrial cardiac muscle cell communication	2/340	14/18614	0.0261886	0.118252	0.0897885	RYR2/ANK2	2
BP	GO:1902947	regulation of tau-protein kinase activity	2/340	14/18614	0.0261886	0.118252	0.0897885	HGF/EGR1	2
BP	GO:1904044	response to aldosterone	2/340	14/18614	0.0261886	0.118252	0.0897885	SCNN1G/ACE	2

BP	GO:0006575	cellular modified amino acid metabolic process	6/340	124/18614	0.02638302	0.11897762	0.09033384	SHMT2/CBS/DUOX1/DUOXA1/MTHFD2/SULT1B1	6
BP	GO:0017157	regulation of exocytosis	8/340	194/18614	0.02647767	0.11927702	0.09056416	P2RY1/CD177/CSPG5/HMOX1/FGR/FCGR2B/CFTR/FGA	8
BP	GO:0048588	developmental cell growth	9/340	231/18614	0.02653528	0.11938318	0.09064464	OLFM1/CXCL12/SEMA3D/ITGA4/SEMA6D/TNR/SLIT3/SEMA6A/SEMA3G	9
BP	GO:0000768	syncytium formation by plasma membrane fusion	4/340	62/18614	0.02663621	0.11954667	0.09077715	NFATC2/ADAMTS15/CXCL10/CXCL9	4

BP	GO:0140253	cell-cell fusion	4/340	62/18614	0.0266321	0.1195467	0.0907715	NFATC2/ADAMTS15/CXCL10/CXCL9	4
BP	GO:0050769	positive regulation of neurogenesis	9/340	232/18614	0.02717189	0.1216169	0.09233434	CXCL12/ROBO2/EGR2/ACE/IL1B/IL6/NTRK2/CX3CR1/CX3CL1	9
BP	GO:0002791	regulation of peptide secretion	8/340	195/18614	0.02718891	0.1216169	0.09233434	CNR1/CCL5/IL1B/ANO1/S100A8/CFTR/IL6/FGA	8
BP	GO:0031345	negative regulation of cell projection organization	8/340	195/18614	0.02718891	0.1216169	0.09233434	SEMA3D/SEMA6D/DPYSL3/TNR/STAP1/FAT3/SEMA6A/SEMA3G	8
BP	GO:19	release of seques	3/340	36/18614	0.027	0.123	0.093	RYR2/ANK2/CASQ2	3

	03514	tered calcium ion into cytosol by endoplasmic reticulum			6526	5452	8076		
BP	GO:1901606	alpha-amino acid catabolic process	5/340	93/18614	0.0278374	0.1240801	0.0942137	FAH/SHMT2/CBS/HMGCLL1/IDO1	5
BP	GO:1903035	negative regulation of response to wounding	5/340	93/18614	0.0278374	0.1240801	0.0942137	SIGLEC10/MMRN1/SERPINF2/TNR/FGA	5

BP	GO:0070302	regulation of stress-activated protein kinase signaling cascade	8/340	196/18614	0.02791 47	0.12427 9	0.09436 48	HGF/NAIP/TGFB2/SERPINF2/IL1B/CD27/FCGR2B/RASGRP1	8
BP	GO:0010573	vascular endothelial growth factor production	4/340	63/18614	0.02804 19	0.12469 97	0.09468 42	PTGS2/IL1B/SULF1/IL6	4
BP	GO:0003231	cardiac ventricle development	6/340	126/18614	0.02824 35	0.12544 99	0.09525 38	RYR2/ROBO2/TGFB2/APLNR/SLIT3/CPE	6

BP	GO:0032868	response to insulin	10/340	272/18614	0.0283733	0.1258785	0.0955793	TRIB3/EGR1/EGR2/SLC39A14/ALPL/RARRES2/IL1B/FOS/TNFSF10/SOCS3	10
BP	GO:0090087	regulation of peptide transport	8/340	197/18614	0.0286536	0.1269754	0.0964121	CNR1/CCL5/IL1B/ANO1/S100A8/CFTR/IL6/FGA	8
BP	GO:0007162	negative regulation of cell adhesion	11/340	312/18614	0.0289141	0.1279811	0.0971758	CXCL12/TIGIT/TNR/LAX1/VSIG4/FCGR2B/IRF1/SEMA6A/CX3CL1/PLA2G2A/IDO1	11
BP	GO:1900407	regulation of cellular response to oxidative stress	5/340	94/18614	0.0289978	0.1281148	0.0972773	PYCR1/HGF/DDR2/SLC7A11/NR4A3	5

BP	GO:0044403	biological process involved in symbiotic interaction	11/340	313/18614	0.0294992	0.1281423	0.0972982	JUN/CCL5/FASN/ZDHHC9/DPP4/SIGLEC1/ANPEP/CH25H/PTX3/CX3CR1/CCL8	11
BP	GO:0030104	water homeostasis	3/340	37/18614	0.0296963	0.1281423	0.0972982	SCNN1G/CFTR/HAS2	3
BP	GO:0031646	positive regulation of nervous system process	3/340	37/18614	0.0296963	0.1281423	0.0972982	HGF/EGR2/TNR	3
BP	GO:0033280	response to vitamin D	3/340	37/18614	0.0296963	0.1281423	0.0972982	PTGS2/ALPL/CXCL10	3

BP	GO:0034110	regulation of homotypic cell-cell adhesion	3/340	37/18614	0.0296963	0.1281423	0.0972982	MMRN1/CCL5/IL6	3
BP	GO:0070536	protein K63-linked deubiquitination	3/340	37/18614	0.0296963	0.1281423	0.0972982	SHMT2/STAMBPL1/OTUD1	3
BP	GO:0071312	cellular response to alkaloid	3/340	37/18614	0.0296963	0.1281423	0.0972982	RYR2/CASQ2/SLC1A3	3
BP	GO:0086019	cell-cell signaling involved in cardiac conduction	3/340	37/18614	0.0296963	0.1281423	0.0972982	RYR2/ANK2/CASQ2	3

BP	GO:0002468	dendritic cell antigen processing and presentation	2/340	15/18614	0.02985584	0.12814 23	0.09729 82	FCGR2B/SLC11A1	2
BP	GO:0002864	regulation of acute inflammatory response to antigenic stimulus	2/340	15/18614	0.02985584	0.12814 23	0.09729 82	CNR1/FCGR2B	2
BP	GO:0003214	cardiac left ventricle morphogenesis	2/340	15/18614	0.02985584	0.12814 23	0.09729 82	RYR2/CPE	2

BP	GO:0009071	serine family amino acid catabolic process	2/340	15/18614	0.02985584	0.12814423	0.09729982	SHMT2/CBS	2
BP	GO:0010421	hydrogen peroxide-mediated programmed cell death	2/340	15/18614	0.02985584	0.12814423	0.09729982	HGF/DDR2	2
BP	GO:0032725	positive regulation of granulocyte macrophage colony-	2/340	15/18614	0.02985584	0.12814423	0.09729982	IL1B/RASGRP1	2

		stimulating factor production							
BP	GO:0032905	transforming growth factor beta1 production	2/340	15/18614	0.02985584	0.1281423	0.09729282	LUM/CX3CL1	2
BP	GO:0034310	primary alcohol catabolic process	2/340	15/18614	0.02985584	0.1281423	0.09729282	AKR1C3/SULT1B1	2
BP	GO:0035641	locomotory exploration behavior	2/340	15/18614	0.02985584	0.1281423	0.09729282	TNR/DPP4	2

BP	GO:0045820	negative regulation of glycolytic process	2/340	15/18614	0.02985584	0.1281423	0.0972982	IER3/DDIT4	2
BP	GO:0046653	tetrahydrofolate metabolic process	2/340	15/18614	0.02985584	0.1281423	0.0972982	SHMT2/MTHFD2	2
BP	GO:0046655	folic acid metabolic process	2/340	15/18614	0.02985584	0.1281423	0.0972982	SHMT2/MTHFD2	2
BP	GO:0051284	positive regulation of sequestering	2/340	15/18614	0.02985584	0.1281423	0.0972982	RYR2/CASQ2	2

		of calcium ion							
BP	GO:0060211	regulation of nuclear-transcribed mRNA poly(A) tail shortening	2/340	15/18614	0.02985584	0.1281423	0.09729982	BTG2/ZFP36	2
BP	GO:0090280	positive regulation of calcium ion import	2/340	15/18614	0.02985584	0.1281423	0.09729982	CXCL12/CCL2	2
BP	GO:0098883	synapse pruning	2/340	15/18614	0.02985584	0.1281423	0.09729982	CX3CR1/CX3CL1	2

BP	GO:1901142	insulin metabolic process	2/340	15/18614	0.02985584	0.1281423	0.0972982	PCSK2/CPE	2
BP	GO:2001279	regulation of unsaturated fatty acid biosynthetic process	2/340	15/18614	0.02985584	0.1281423	0.0972982	PTGS2/IL1B	2
BP	GO:0014033	neural crest cell differentiation	5/340	95/18614	0.0301476	0.128762	0.0977687	FRZB/SEMA3D/SEMA6D/SEMA6A/SEMA3G	5
BP	GO:0019217	regulation of fatty acid metabolic	5/340	95/18614	0.0301476	0.128762	0.0977687	TRIB3/PTGS2/CNR1/IL1B/NR4A3	5

		process							
BP	GO:0043648	dicarboxylic acid metabolic process	5/340	95/18614	0.03014476	0.1287762	0.09776687	SHMT2/GCLM/IDH1/MTHFD2/SLC7A11	5
BP	GO:0045638	negative regulation of myeloid cell differentiation	5/340	95/18614	0.03014476	0.1287762	0.09776687	HMGB3/TNFRSF11B/ZFP36/TRIB1/INHBA	5
BP	GO:0009746	response to hexose	8/340	199/18614	0.03017717	0.1287762	0.09776687	ZBTB20/GCLM/PTGS2/EGR1/ACE/SLC39A14/ANO1/CFTR	8
BP	GO:0050708	regulation of protein secretion	10/340	275/18614	0.03026681	0.1290286	0.09799711	TGFB2/CNR1/CCL5/IL1B/MYH10/ANO1/CFTR/SAA1/IL6/FGA	10

BP	GO:0072577	endothelial cell apoptotic process	4/340	65/18614	0.03099926	0.1319696	0.10020042	ITGA4/AKR1C3/CCL2/FGA	4
BP	GO:0045936	negative regulation of phosphate metabolic process	14/340	439/18614	0.03164485	0.13461121	0.10222107	TRIB3/HGF/TGFB2/JUN/DDR2/STAP1/LAX1/IER3/IL1B/SFN/TRIB1/DDIT4/INHBA/SOCS3	14
BP	GO:0009595	detection of biotic stimulus	3/340	38/18614	0.03181676	0.13472798	0.10230001	NAIP/NLRC4/SRPX	3
BP	GO:0014046	dopamine secretion	3/340	38/18614	0.03181676	0.13472798	0.10230001	SLC18A2/CXCL12/CNR1	3

BP	GO:0045923	positive regulation of fatty acid metabolic process	3/340	38/18614	0.03181876	0.1347298	0.10230001	PTGS2/IL1B/NR4A3	3
BP	GO:0070229	negative regulation of lymphocyte apoptotic process	3/340	38/18614	0.03181876	0.1347298	0.10230001	CCL5/CD27/IDO1	3
BP	GO:0050890	cognition	11/340	317/18614	0.03192454	0.13503323	0.10252297	BTG2/PTGS2/CNR1/EGR2/TNR/SLC7A11/FOS/NRXN3/NTRK2/CX3CR1/SLC6A4	11
BP	GO:00	negative regulati	14/340	440/18614	0.032	0.135	0.103	TRIB3/HGF/TGFB2/JUN/DDR2/STAP1/LAX1/IER3/IL1B/SFN/TRIB1/DDIT4/INHBA/SOCS3	14

	10563	on of phosphorus metabolic process			1702	9209	2044		
BP	GO:1903305	regulation of regulated secretory pathway	6/340	130/18614	0.0322258	0.1360052	0.1032685	P2RY1/CD177/CSPG5/HMOX1/FGR/FCGR2B	6
BP	GO:0006949	syncytium formation	4/340	66/18614	0.0325339	0.137024	0.104042	NFATC2/ADAMTS15/CXCL10/CXCL9	4
BP	GO:0002709	regulation of T cell mediated immunity	5/340	97/18614	0.0325751	0.137024	0.104042	CD1C/CD1E/IL1B/FCGR2B/IL6	5

BP	GO:0006942	regulation of striated muscle contraction	5/340	97/18614	0.03257551	0.1370024	0.1040442	RYR2/ANK2/PIK3CG/CASQ2/NR4A1	5
BP	GO:0070665	positive regulation of leukocyte proliferation	7/340	166/18614	0.03306628	0.13892219	0.10548831	HLA-DPA1/NFATC2/CCL5/IL1B/VCAM1/CD3E/IL6	7
BP	GO:0051216	cartilage development	8/340	203/18614	0.03337721	0.13922229	0.10571116	FRZB/BMP5/CBS/PKDCC/BMP1/SLC39A14/SULF1/OGN	8
BP	GO:0051962	positive regulation of nervous system development	10/340	280/18614	0.03362227	0.13922229	0.10571116	CXCL12/ROBO2/EGR2/ACE/IL1B/LRRN3/IL6/NTRK2/CX3CR1/CX3CL1	10

BP	GO:0006544	glycine metabolic process	2/340	16/18614	0.03371189	0.13922229	0.10571116	SHMT2/PHGDH	2
BP	GO:0006750	glutathione biosynthetic process	2/340	16/18614	0.03371189	0.13922229	0.10571116	GCLM/SLC7A11	2
BP	GO:0007158	neuron cell-cell adhesion	2/340	16/18614	0.03371189	0.13922229	0.10571116	TNR/NRXN3	2
BP	GO:0009074	aromatic amino acid family catabolic process	2/340	16/18614	0.03371189	0.13922229	0.10571116	FAH/IDO1	2

BP	GO:0010819	regulation of T cell chemotaxis	2/340	16/18614	0.00337189	0.13922229	0.1057116	CCL5/CXCL10	2
BP	GO:0016045	detection of bacterium	2/340	16/18614	0.00337189	0.13922229	0.1057116	NAIP/NLRC4	2
BP	GO:0044849	estrous cycle	2/340	16/18614	0.00337189	0.13922229	0.1057116	EGR1/HAS2	2
BP	GO:0045579	positive regulation of B cell differentiation	2/340	16/18614	0.00337189	0.13922229	0.1057116	IL2RG/CD27	2
BP	GO:0045779	negative regulation of bone resorption	2/340	16/18614	0.00337189	0.13922229	0.1057116	TNFRSF11B/IL6	2

BP	GO:0061469	regulation of type B pancreatic cell proliferation	2/340	16/18614	0.03371 89	0.13922 29	0.10571 16	NR4A3/NR4A1	2
BP	GO:0070230	positive regulation of lymphocyte apoptotic process	2/340	16/18614	0.03371 89	0.13922 29	0.10571 16	CCL5/IDO1	2
BP	GO:0097468	programmed cell death in response to reactive	2/340	16/18614	0.03371 89	0.13922 29	0.10571 16	HGF/DDR2	2

		oxygen species							
BP	GO:1900747	negative regulation of vascular endothelial growth factor signaling pathway	2/340	16/18614	0.0337189	0.1392229	0.1057116	DCN/SEMA6A	2
BP	GO:1901741	positive regulation of myoblast fusion	2/340	16/18614	0.0337189	0.1392229	0.1057116	NFATC2/CXCL9	2
BP	GO:0032642	regulation of chemokine	5/340	98/18614	0.0338332	0.1395438	0.1059553	EGR1/HMOX1/IL1B/ALOX15B/IL6	5

		production							
BP	GO:0030851	granulocyte differentiation	3/340	39/18614	0.0340161	0.1398158	0.1061619	FASN/TRIB1/CSF3	3
BP	GO:0042401	biogenic amine biosynthetic process	3/340	39/18614	0.0340161	0.1398158	0.1061619	TGFB2/HDC/NR4A2	3
BP	GO:0070232	regulation of T cell apoptotic process	3/340	39/18614	0.0340161	0.1398158	0.1061619	CCL5/CD27/IDO1	3
BP	GO:0001912	positive regulation of leukocyte mediat	4/340	67/18614	0.03411193	0.1398158	0.1061619	CD1C/STAP1/CD1E/RASGRP1	4

		ed cytotoxicity							
BP	GO:0038034	signal transduction in absence of ligand	4/340	67/18614	0.0341193	0.1398158	0.1061619	SRPX/IL1B/INHBA/CX3CL1	4
BP	GO:0097192	extrinsic apoptotic signaling pathway in absence of ligand	4/340	67/18614	0.0341193	0.1398158	0.1061619	SRPX/IL1B/INHBA/CX3CL1	4
BP	GO:0050804	modulation of chemical synaptic	15/340	487/18614	0.0347493	0.1422443	0.1080058	P2RY1/PTGS2/CNR1/EGR2/CSPG5/TNR/ACE/SLC7A11/IL1B/SLC1A3/NTRK2/CX3CR1/CX3CL1/CCL2/SLC6A4	15

		transmission							
BP	GO:0002790	peptide secretion	9/340	243/18614	0.0350319	0.1428462	0.1084628	CNR1/CCL5/IL1B/ANO1/S100A8/CPE/CFTR/IL6/FGA	9
BP	GO:0019218	regulation of steroid metabolic process	5/340	99/18614	0.0351212	0.1428462	0.1084628	BMP5/EPHX2/EGR1/DHCR7/AKR1C3	5
BP	GO:0032602	chemokine production	5/340	99/18614	0.0351212	0.1428462	0.1084628	EGR1/HMOX1/IL1B/ALOX15B/IL6	5
BP	GO:0036473	cell death in response to oxidative stress	5/340	99/18614	0.0351212	0.1428462	0.1084628	PYCR1/HGF/DDR2/SLC7A11/NR4A3	5

BP	GO:0098869	cellular oxidant detoxification	5/340	99/18614	0.0351212	0.1428462	0.1084628	HBB/PTGS2/DUOX1/NQO1/GSTT1	5
BP	GO:2001243	negative regulation of intrinsic apoptotic signaling pathway	5/340	99/18614	0.0351212	0.1428462	0.1084628	RTKN2/CXCL12/ENO1/HYOU1/PTGS2	5
BP	GO:0099177	regulation of trans-synaptic signaling	15/340	488/18614	0.0352809	0.1433427	0.1088398	P2RY1/PTGS2/CNR1/EGR2/CSPG5/TNR/ACE/SLC7A11/IL1B/SLC1A3/NTRK2/CX3CR1/CX3CL1/CCL2/SLC6A4	15
BP	GO:0002704	negative regulation of	4/340	68/18614	0.035749	0.1447816	0.1099323	CD96/HMOX1/FCGR2B/CX3CR1	4

		leukocyte mediated immunity							
BP	GO:0051057	positive regulation of small GTPase mediated signal transduction	4/340	68/18614	0.0357749	0.1447816	0.1099323	PIK3CG/GPR4/P2RY10/RASGRP1	4
BP	GO:1902893	regulation of miRNA transcription	4/340	68/18614	0.0357749	0.1447816	0.1099323	TGFB2/EGR1/JUN/FOS	4
BP	GO:0009309	amine biosynthetic	3/340	40/18614	0.0362914	0.1465113	0.1112457	TGFB2/HDC/NR4A2	3

		process							
BP	GO:0032570	response to progesterone	3/340	40/18614	0.03629 14	0.14651 13	0.11124 57	TGFB2/FOSB/FOS	3
BP	GO:0035886	vascular associated smooth muscle cell differentiation	3/340	40/18614	0.03629 14	0.14651 13	0.11124 57	NFATC2/APLNR/CTH	3
BP	GO:0030101	natural killer cell activation	5/340	100/18614	0.03643 91	0.14695 17	0.11158 01	SLAMF7/FGR/IL2RB/RASGRP1/CD2	5
BP	GO:0034284	response to monosaccharide	8/340	207/18614	0.03679 63	0.14823 53	0.11255 47	ZBTB20/GCLM/PTGS2/EGR1/ACE/SLC39A14/ANO1/CFTR	8

BP	GO:0007254	JNK cascade	7/340	170/18614	0.036888552	0.148280 1	0.11258 88	NAIP/SERPINF2/IL1B/CD27/FCGR2B/RASGRP1/TRIB1	7
BP	GO:1903034	regulation of response to wounding	7/340	170/18614	0.036888552	0.14828 01	0.11258 88	SIGLEC10/DUOX1/MMRN1/SERPINF2/TNR/DDR2/FGA	7
BP	GO:0002711	positive regulation of T cell mediated immunity	4/340	69/18614	0.0374 23	0.14861 35	0.11284 19	CD1C/CD1E/IL1B/IL6	4
BP	GO:0042093	T-helper cell differentiation	4/340	69/18614	0.0374 23	0.14861 35	0.11284 19	NFKBIZ/JUNB/LY9/IL6	4
BP	GO:00	miRNA transcription	4/340	69/18614	0.037	0.148	0.112	TGFB2/EGR1/JUN/FOS	4

	61614				423	6135	8419		
BP	GO:0002285	lymphocyte activation involved in immune response	8/340	208/18614	0.0376879	0.1486135	0.1128419	CD1C/NFKBIZ/JUNB/LY9/FCGR2B/SLC11A1/IL6/CLEC4E	8
BP	GO:0042476	odontogenesis	6/340	135/18614	0.0376951	0.1486135	0.1128419	TGFB2/TNFRSF11B/ALPL/INHBA/CFTR/ASPN	6
BP	GO:0009615	response to virus	13/340	408/18614	0.0377271	0.1486135	0.1128419	RNASE2/CXCL12/ENO1/PIM2/CCL5/IL1B/FGR/DDIT4/IRF1/IL6/CXCL10/CCL8/CXCL9	13
BP	GO:0000097	sulfur amino acid biosynthetic	2/340	17/18614	0.0377619	0.1486135	0.1128419	CBS/CTH	2

		process							
BP	GO:0001991	regulation of systemic arterial blood pressure by circulatory renin-angiotensin	2/340	17/18614	0.0377619	0.1486135	0.1128419	CPA3/ACE	2
BP	GO:0002921	negative regulation of humoral immune response	2/340	17/18614	0.0377619	0.1486135	0.1128419	VSIG4/FCGR2B	2

BP	GO:0017014	protein nitrosylation	2/340	17/18614	0.03776 19	0.14861 35	0.11284 19	ACE/S100A8	2
BP	GO:0018119	peptidyl-cysteine S-nitrosylation	2/340	17/18614	0.03776 19	0.14861 35	0.11284 19	ACE/S100A8	2
BP	GO:0019184	nonribosomal peptide biosynthetic process	2/340	17/18614	0.03776 19	0.14861 35	0.11284 19	GCLM/SLC7A11	2
BP	GO:0035729	cellular response to hepatocyte growth factor stimulus	2/340	17/18614	0.03776 19	0.14861 35	0.11284 19	GCLM/HGF	2

BP	GO:0036005	response to macrophage colony-stimulating factor	2/340	17/18614	0.037619	0.1486135	0.1128419	STAP1/ALPL	2
BP	GO:0046827	positive regulation of protein export from nucleus	2/340	17/18614	0.037619	0.1486135	0.1128419	IL1B/SFN	2
BP	GO:0051412	response to corticosterone	2/340	17/18614	0.037619	0.1486135	0.1128419	FOSB/FOS	2
BP	GO:0072109	glomerular mesangium development	2/340	17/18614	0.037619	0.1486135	0.1128419	EGR1/GPR4	2

BP	GO:0098712	L-glutamate import across plasma membrane	2/340	17/18614	0.0377619	0.1486135	0.1128419	SLC7A11/SLC1A3	2
BP	GO:0099509	regulation of presynaptic cytosolic calcium ion concentration	2/340	17/18614	0.0377619	0.1486135	0.1128419	P2RY1/CNR1	2
BP	GO:0110096	cellular response to aldehyde	2/340	17/18614	0.0377619	0.1486135	0.1128419	SCNN1G/ACE	2
BP	GO:00	cardiac conduction	5/340	101/18614	0.037	0.148	0.112	CORIN/RYR2/ANK2/TRPC1/CASQ2	5

	61337				7871	6135	8419			
BP	GO:0046883	regulation of hormone secretion	9/340	247/18614	0.0382329	0.1502236	0.1140739	P2RY1/CNR1/CCL5/IL1B/ANO1/INHBA/CFTR/IL6/FGA		9
BP	GO:0042417	dopamine metabolic process	3/340	41/18614	0.0386443	0.1513553	0.1149237	TGFB2/MAOA/NR4A2		3
BP	GO:0140467	integrated stress response signaling	3/340	41/18614	0.0386443	0.1513553	0.1149237	JUN/JUNB/FOS		3
BP	GO:1902742	apoptotic process involve	3/340	41/18614	0.0386443	0.1513553	0.1149237	ROBO2/TGFB2/SLIT3		3

		d in development							
BP	GO:0071333	cellular response to glucose stimulus	6/340	136/18614	0.03885 57	0.15203 19	0.11543 75	ZBTB20/GCLM/ACE/SLC39A14/ANO1/CFTR	6
BP	GO:0001837	epithelial to mesenchymal transition	7/340	172/18614	0.03890 13	0.15205 42	0.11545 44	OLFM1/BMP5/HGF/TGFB2/IL1B/IL6/HAS2	7
BP	GO:2000379	positive regulation of reactive oxygen species metabolic process	4/340	70/18614	0.03914 14	0.15283 6	0.11604 81	CD177/DUOXA1/AKR1C3/FPR2	4

BP	GO:0006937	regulation of muscle contraction	7/340	173/18614	0.03993 59	0.15561 93	0.11816 14	RYR2/ENO1/PTGS2/ANK2/PIK3CG/CASQ2/NR4A1	7
BP	GO:0031214	biomineral tissue development	7/340	173/18614	0.03993 59	0.15561 93	0.11816 14	PTGS2/PKDCC/DDR2/ALPL/FGR/CFTR/ASPN	7
BP	GO:0008630	intrinsic apoptotic signaling pathway in response to DNA damage	5/340	103/18614	0.04057 37	0.15778 19	0.11980 34	CXCL12/HMOX1/IER3/SFN/DDIT4	5
BP	GO:1902882	regulation of response to	5/340	103/18614	0.04057 37	0.15778 19	0.11980 34	PYCR1/HGF/DDR2/SLC7A11/NR4A3	5

		oxidative stress							
BP	GO:0010631	epithelial cell migration	12/340	371/18614	0.0407218	0.1581107	0.1200531	PTGS2/TGFB2/PIK3CG/JUN/EGR3/DPP4/HMOX1/DCN/ADAMTS9/NR4A1/S100A12/HAS2	12
BP	GO:0001649	osteoblast differentiation	9/340	250/18614	0.040772	0.1581107	0.1200531	HGF/VCAN/FASN/DDR2/JUNB/JUND/ALPL/TNC/IL6	9
BP	GO:0045861	negative regulation of proteolysis	11/340	330/18614	0.0407827	0.1581107	0.1200531	HGF/PTGS2/NAIP/TIMP4/SERPINF2/SERPINA3/SFN/IL1R2/CD27/PI3/NR4A1	11
BP	GO:0002294	CD4-positive, alpha-beta T cell differentiation involved in	4/340	71/18614	0.0409042	0.1582599	0.1201664	NFKBIZ/JUNB/LY9/IL6	4

		immune response							
BP	GO:0045600	positive regulation of fat cell differentiation	4/340	71/18614	0.04090042	0.15825599	0.12016164	FRZB/PTGS2/ZFP36/RARRES2	4
BP	GO:0070296	sarcoplasmic reticulum calcium ion transport	3/340	42/18614	0.04107704	0.15858809	0.1204101	RYR2/ANK2/CASQ2	3
BP	GO:0085029	extracellular matrix assembly	3/340	42/18614	0.04107704	0.15858809	0.1204101	MFAP4/MYH11/HAS2	3
BP	GO:00	cellular response to	6/340	138/18614	0.041	0.159	0.120	ZBTB20/GCLM/ACE/SLC39A14/ANO1/CFTR	6

	71331	hexose stimulus			2446	0299	7511		
BP	GO:0002377	immunoglobulin production	8/340	212/18614	0.04139393	0.15902299	0.12075111	HLA-DPA1/HLA-DOA/HLA-DOB/NFKBIZ/FCGR2B/IL6/HLA-DQA1/HLA-DQB1	8
BP	GO:0002295	T-helper cell lineage commitment	2/340	18/18614	0.04197795	0.15902299	0.12075111	LY9/IL6	2
BP	GO:0002693	positive regulation of cellular extravasation	2/340	18/18614	0.04197795	0.15902299	0.12075111	LYVE1/PLVAP	2
BP	GO:0002827	positive regulation of T-helper 1 type immun	2/340	18/18614	0.04197795	0.15902299	0.12075111	IL1B/SLC11A1	2

		e response							
BP	GO:0002830	positive regulation of type 2 immune response	2/340	18/18614	0.04197995	0.1590299	0.1207511	IL6/IDO1	2
BP	GO:0010755	regulation of plasminogen activation	2/340	18/18614	0.04197995	0.1590299	0.1207511	ENO1/SERPINF2	2
BP	GO:0030730	sequestering of triglyceride	2/340	18/18614	0.04197995	0.1590299	0.1207511	PLIN2/IL1B	2
BP	GO:0032310	prostaglandin secretion	2/340	18/18614	0.04197995	0.1590299	0.1207511	PTGS2/IL1B	2

BP	GO:0032354	response to follicle-stimulating hormone	2/340	18/18614	0.0419795	0.1590299	0.12075511	GCLM/INHBA	2
BP	GO:0032604	granulocyte macrophage colony-stimulating factor production	2/340	18/18614	0.0419795	0.1590299	0.12075511	IL1B/RASGRP1	2
BP	GO:0032645	regulation of granulocyte macrophage colony-stimulating	2/340	18/18614	0.0419795	0.1590299	0.12075511	IL1B/RASGRP1	2

		factor production							
BP	GO:0042574	retinal metabolic process	2/340	18/18614	0.0419795	0.1590299	0.1207511	SDR16C5/AKR1C3	2
BP	GO:0046851	negative regulation of bone remodeling	2/340	18/18614	0.0419795	0.1590299	0.1207511	TNFRSF11B/IL6	2
BP	GO:0050665	hydrogen peroxide biosynthetic process	2/340	18/18614	0.0419795	0.1590299	0.1207511	DUOX1/DUOXA1	2
BP	GO:00	detection of temper	2/340	18/18614	0.041	0.159	0.120	CXCL12/ANO1	2

	50965	ature stimulus involved in sensory perception of pain			9795	0299	7511		
BP	GO:0071318	cellular response to ATP	2/340	18/18614	0.0419795	0.1590299	0.1207511	P2RY1/PTGS2	2
BP	GO:1900037	regulation of cellular response to hypoxia	2/340	18/18614	0.0419795	0.1590299	0.1207511	ENO1/HYOU1	2
BP	GO:1902548	negative regulation of cellular respon	2/340	18/18614	0.0419795	0.1590299	0.1207511	DCN/SEMA6A	2

		se to vascular endothelial growth factor stimulus							
BP	GO:0008016	regulation of heart contraction	8/340	213/18614	0.04236 38	0.16032 61	0.12173 53	CORIN/RYR2/FXYD1/ANK2/TGFB2/PIK3CG/TRPC1/CASQ2	8
BP	GO:0046328	regulation of JNK cascade	6/340	139/18614	0.04247 31	0.16058 03	0.12192 82	NAIP/SERPINF2/IL1B/CD27/FCGR2B/RASGRP1	6
BP	GO:0002287	alpha-beta T cell activation involved in	4/340	72/18614	0.04271 15	0.16084 33	0.12212 79	NFKBIZ/JUNB/LY9/IL6	4

		immune response							
BP	GO:0002293	alpha-beta T cell differentiation involved in immune response	4/340	72/18614	0.0427115	0.1608433	0.1221279	NFKBIZ/JUNB/LY9/IL6	4
BP	GO:0046635	positive regulation of alpha-beta T cell activation	4/340	72/18614	0.0427115	0.1608433	0.1221279	NFKBIZ/IL2RG/CD83/CD3E	4
BP	GO:00	negative regulati	4/340	72/18614	0.042	0.160	0.122	PTGS2/GEM/CASQ2/ACE	4

	51926	on of calcium ion transport			7115	8433	1279		
BP	GO:0090132	epithelium migration	12/340	374/18614	0.042842	0.1611755	0.1223802	PTGS2/TGFB2/PIK3CG/JUN/EGR3/DPP4/HMOX1/DCN/ADAMTS9/NR4A1/S100A12/HAS2	12
BP	GO:0001764	neuron migration	7/340	176/18614	0.0431469	0.1621625	0.1231296	CXCL12/FAT3/DDIT4/SEMA6A/NTRK2/CX3CL1/NR4A2	7
BP	GO:0032760	positive regulation of tumor necrosis factor production	5/340	105/18614	0.0434818	0.1632603	0.1239632	ZBTB20/ORM1/RASGRP1/CD2/IL6	5
BP	GO:0043271	negative regulation of monoat	6/340	140/18614	0.0437244	0.1638481	0.1244095	KCNAB1/PTGS2/GEM/CNR1/CASQ2/ACE	6

		omic ion transport							
BP	GO:0071326	cellular response to monosaccharide stimulus	6/340	140/18614	0.0437244	0.1638481	0.1244095	ZBTB20/GCLM/ACE/SLC39A14/ANO1/CFTR	6
BP	GO:0015718	monocarboxylic acid transport	7/340	177/18614	0.0442532	0.1656671	0.1257907	PLIN2/PTGS2/SLCO4A1/ACE/IL1B/AQP9/PLA2G2A	7
BP	GO:0090257	regulation of muscle system process	9/340	254/18614	0.0443227	0.1657641	0.1258643	RYR2/ENO1/GTF2IRD1/PTGS2/ANK2/PIK3CG/CASQ2/NR4A3/NR4A1	9
BP	GO:00	regulation of alpha-	4/340	73/18614	0.044	0.166	0.126	NFKBIZ/JUNB/IL2RG/CD83	4

	46637	beta T cell differentiation			5631	1741	1757		
BP	GO:0050795	regulation of behavior	4/340	73/18614	0.0445631	0.1661741	0.1261757	SGIP1/PTGDS/CNR1/NR4A3	4
BP	GO:0051937	catecholamine transport	4/340	73/18614	0.0445631	0.1661741	0.1261757	P2RY1/SLC18A2/CXCL12/CNR1	4
BP	GO:0008361	regulation of cell size	7/340	178/18614	0.0453777	0.1689347	0.1282718	OLFM1/CXCL12/SEMA3D/SEMA6D/TNR/SEMA6A/SEMA3G	7
BP	GO:0042181	ketone biosynthetic process	3/340	44/18614	0.0461498	0.1689347	0.1282718	BMP5/EGR1/AKR1C3	3
BP	GO:0048512	circadian behavior	3/340	44/18614	0.0461498	0.1689347	0.1282718	PTGDS/EGR1/NR1D2	3

BP	GO:0051602	response to electrical stimulus	3/340	44/18614	0.00461498	0.1689347	0.1282718	BTG2/NQO1/NR4A1	3
BP	GO:1900026	positive regulation of substrate adhesion-dependent cell spreading	3/340	44/18614	0.00461498	0.1689347	0.1282718	CSPG5/HAS2/FGA	3
BP	GO:0006898	receptor-mediated endocytosis	9/340	256/18614	0.00461734	0.1689347	0.1282718	SGIP1/ITGA4/DPP4/SIGLEC1/STAB1/LYVE1/FCGR2B/MARCO/SELE	9
BP	GO:00	negative regulati	11/340	337/18614	0.046	0.168	0.128	HGF/PIK3CG/TIMP4/CNR1/HMOX1/NQO1/ZFP36/IER3/IL1B/DDIT4/SLC11A1	11

	09895	on of catabolic process			1994	9347	2718		
BP	GO:0001889	liver development	6/340	142/18614	0.0462956	0.1689347	0.1282718	FRZB/ARID5B/HGF/JUN/HMOX1/IL6	6
BP	GO:0003184	pulmonary valve morphogenesis	2/340	19/18614	0.0463641	0.1689347	0.1282718	ROBO2/TGFB2	2
BP	GO:0010744	positive regulation of macrophage derived foam cell differentiation	2/340	19/18614	0.0463641	0.1689347	0.1282718	ALOX15B/PLA2G2A	2

BP	GO:0010759	positive regulation of macrophage chemotaxis	2/340	19/18614	0.04636641	0.1689347	0.12827718	CCL5/RARRES2	2
BP	GO:0031643	positive regulation of myelination	2/340	19/18614	0.04636641	0.1689347	0.12827718	HGF/EGR2	2
BP	GO:0032930	positive regulation of superoxide anion generation	2/340	19/18614	0.04636641	0.1689347	0.12827718	CD177/FPR2	2
BP	GO:0035728	response to hepatocyte growth factor	2/340	19/18614	0.04636641	0.1689347	0.12827718	GCLM/HGF	2

BP	GO:0035743	CD4-positive, alpha-beta T cell cytokine production	2/340	19/18614	0.04636 41	0.1689347	0.1282718	IL1B/IL6	2
BP	GO:0051900	regulation of mitochondrial depolarization	2/340	19/18614	0.0463641	0.1689347	0.1282718	GCLM/DCN	2
BP	GO:0086014	atrial cardiac muscle cell action potential	2/340	19/18614	0.0463641	0.1689347	0.1282718	RYR2/ANK2	2
BP	GO:0086026	atrial cardiac muscle cell to	2/340	19/18614	0.0463641	0.1689347	0.1282718	RYR2/ANK2	2

		AV node cell signaling							
BP	GO:0086066	atrial cardiac muscle cell to AV node cell communication	2/340	19/18614	0.0463641	0.1689347	0.1282718	RYR2/ANK2	2
BP	GO:0098543	detection of other organism	2/340	19/18614	0.0463641	0.1689347	0.1282718	NAIP/NLRC4	2
BP	GO:0003281	ventricular septum development	4/340	74/18614	0.0464591	0.1689347	0.1282718	ROBO2/TGFB2/APLNR/SLIT3	4

BP	GO:0031343	positive regulation of cell killing	4/340	74/18614	0.04645 91	0.16893 47	0.12827 18	CD1C/STAP1/CD1E/RASGRP1	4
BP	GO:1901616	organic hydroxy compound catabolic process	4/340	74/18614	0.04645 91	0.16893 47	0.12827 18	FAH/AKR1C3/SULT1B1/MAOA	4
BP	GO:1903578	regulation of ATP metabolic process	4/340	74/18614	0.04645 91	0.16893 47	0.12827 18	ZBTB20/ENO1/IER3/DDIT4	4
BP	GO:0015837	amine transport	5/340	107/18614	0.04651 21	0.16893 47	0.12827 18	P2RY1/SLC18A2/CXCL12/CNR1/AQP9	5

BP	GO:0034308	primary alcohol metabolic process	5/340	107/18614	0.00465121	0.1689347	0.12827718	AKR1C2/BMP5/SDR16C5/AKR1C3/SULT1B1	5
BP	GO:0007219	Notch signaling pathway	7/340	179/18614	0.00465204	0.1689347	0.12827718	WDR12/ROBO2/TGFB2/TIMP4/CNTN1/KRT19/POSTN	7
BP	GO:0090130	tissue migration	12/340	379/18614	0.00465446	0.1689347	0.12827718	PTGS2/TGFB2/PIK3CG/JUN/EGR3/DPP4/HMOX1/DCN/ADAMTS9/NR4A1/S100A12/HAS2	12
BP	GO:0033135	regulation of peptidyl-serine phosphorylation	6/340	143/18614	0.00476158	0.17266582	0.1310999	HGF/PTGS2/DDIT4/IL6/NTRK2/CSF3	6
BP	GO:0051048	negative regulation of	7/340	180/18614	0.00476814	0.1727317	0.13115548	P2RY1/CNR1/ACE/HMOX1/IL1B/FCGR2B/INHBA	7

BP	GO:0032963	secretion collagen metabolic process	5/340	108/18614	0.04807 32	0.17398 59	0.13210 71	MFAP4/SERPINF2/DDR2/INHBA/IL6		5
BP	GO:0045669	positive regulation of osteoblast differentiation	4/340	75/18614	0.04839 94	0.17483 42	0.13275 12	HGF/DDR2/JUND/IL6		4
BP	GO:2000242	negative regulation of reproductive process	4/340	75/18614	0.04839 94	0.17483 42	0.13275 12	RPS6KA2/PTGDS/HPGDS/SULF1		4
BP	GO:00	neurotransmitt	3/340	45/18614	0.048	0.175	0.133	SLC18A2/SLC1A3/SLC6A4		3

	01504	er uptake			8004	783	4717		
BP	GO:0097009	energy homeostasis	3/340	45/18614	0.0488004	0.175783	0.1334717	SGIP1/NR4A3/NR1D2	3
BP	GO:1902692	regulation of neuroblast proliferation	3/340	45/18614	0.0488004	0.175783	0.1334717	BTG2/CX3CR1/CX3CL1	3
BP	GO:0050731	positive regulation of peptidyl-tyrosine phosphorylation	7/340	181/18614	0.0488607	0.1758339	0.1335103	HGF/CNTN1/CCL5/ACE/CD3E/IL6/CSF3	7
BP	GO:0034976	response to endoplasmic	9/340	259/18614	0.049045	0.1763305	0.1338874	TRIB3/HYOU1/JUN/SRPX/FCGR2B/CTH/ATF3/CFTR/ERP27	9

		reticulum stress							
BP	GO:0060840	artery development	5/340	109/18614	0.0496665	0.1782236	0.1353248	ROBO2/TGFB2/APLNR/EGR2/ADAMTS9	5
BP	GO:1903557	positive regulation of tumor necrosis factor superfamily cytokine production	5/340	109/18614	0.0496665	0.1782236	0.1353248	ZBTB20/ORM1/RASGRP1/CD2/IL6	5
BP	GO:0060537	muscle tissue development	13/340	426/18614	0.0501171	0.1796765	0.136428	BTG2/RYR2/BMP5/TGFB2/EGR1/EGR2/HLF/ADAMTS9/NR1D2/ATF3/FOS/MYH11/NR4A1	13
BP	GO:00	hepatic obiliary system	6/340	145/18614	0.050	0.180	0.13678	FRZB/ARID5B/HGF/JUN/HMOX1/IL6	6

	61008	development			3254	1401			
BP	GO:0050810	regulation of steroid biosynthetic process	4/340	76/18614	0.0503839	0.1801401	0.13678	BMP5/EGR1/DHCR7/AKR1C3	4
BP	GO:0000272	polysaccharide catabolic process	2/340	20/18614	0.0509081	0.1801401	0.13678	CHIT1/MGAM	2
BP	GO:0001780	neutrophil homeostasis	2/340	20/18614	0.0509081	0.1801401	0.13678	SLC7A11/IL6	2
BP	GO:0002643	regulation of tolerance induction	2/340	20/18614	0.0509081	0.1801401	0.13678	CD3E/IDO1	2

BP	GO:0006700	C21-steroid hormone biosynthetic process	2/340	20/18614	0.0509081	0.1801401	0.13678	BMP5/EGR1	2
BP	GO:0034755	iron ion transmembrane transport	2/340	20/18614	0.0509081	0.1801401	0.13678	SLC39A14/SLC11A1	2
BP	GO:0035813	regulation of renal sodium excretion	2/340	20/18614	0.0509081	0.1801401	0.13678	CORIN/ACE	2
BP	GO:0070314	G1 to G0 transition	2/340	20/18614	0.0509081	0.1801401	0.13678	PHGDH/CAPN3	2

BP	GO:0071243	cellular response to arsenic-containing substance	2/340	20/18614	0.0509081	0.1801401	0.13678	HMOX1/ATF3	2
BP	GO:0071294	cellular response to zinc ion	2/340	20/18614	0.0509081	0.1801401	0.13678	MT1X/MT1M	2
BP	GO:0071371	cellular response to gonadotropin stimulus	2/340	20/18614	0.0509081	0.1801401	0.13678	GCLM/INHBA	2
BP	GO:0071379	cellular response to prostaglandin	2/340	20/18614	0.0509081	0.1801401	0.13678	AKR1C2/AKR1C3	2

		stimulus							
BP	GO:2000251	positive regulation of actin cytoskeleton reorganization	2/340	20/18614	0.0509081	0.1801401	0.13678	STAP1/CSF3	2
BP	GO:0009266	response to temperature stimulus	7/340	183/18614	0.0512746	0.1811464	0.1375441	CXCL12/PTGS2/HMOX1/ANO1/TFEC/FOS/CXCL10	7
BP	GO:0010717	regulation of epithelial to mesenchymal transition	5/340	110/18614	0.0512876	0.1811464	0.1375441	OLFM1/BMP5/TGFB2/IL1B/IL6	5

BP	GO:0035272	exocrine system development	3/340	46/18614	0.051524	0.1814769	0.137795	HGF/TGFB2/TFCP2L1	3
BP	GO:0042088	T-helper 1 type immune response	3/340	46/18614	0.051524	0.1814769	0.137795	NFKBIZ/IL1B/SLC11A1	3
BP	GO:0042551	neuron maturation	3/340	46/18614	0.051524	0.1814769	0.137795	GLDN/CX3CL1/NR4A2	3
BP	GO:0045598	regulation of fat cell differentiation	6/340	146/18614	0.051715	0.1818136	0.1380507	FRZB/TRIB3/PTGS2/ZFP36/RARRES2/IL6	6
BP	GO:0062013	positive regulation of small molecul	6/340	146/18614	0.051715	0.1818136	0.1380507	P2RY1/ZBTB20/ENO1/PTGS2/IL1B/NR4A3	6

		e metabolic process							
BP	GO:0010827	regulation of glucose transmembrane transport	4/340	77/18614	0.0524125	0.1840959	0.1397836	TRIB3/ACE/IL1B/NR4A3	4
BP	GO:0002706	regulation of lymphocyte mediated immunity	7/340	184/18614	0.0525092	0.1842658	0.1399126	CD1C/CD96/CD1E/IL1B/FCGR2B/RASGRP1/IL6	7
BP	GO:0008637	apoptotic mitochondrial	5/340	111/18614	0.0529409	0.1856096	0.140933	GCLM/HGF/IER3/SFN/TNFSF10	5

		changes							
BP	GO:0033674	positive regulation of kinase activity	13/340	430/18614	0.05321215	0.1863868	0.1415231	TGFB2/EGR1/PIK3CG/CCL5/DDR2/ACE/FGD2/IL1B/FGR/RASGRP1/SLC11A1/NTRK2/S100A12	13
BP	GO:0002861	regulation of inflammatory response to antigenic stimulus	3/340	47/18614	0.05431197	0.1892253	0.1436784	CNR1/FGR/FCGR2B	3
BP	GO:0010837	regulation of keratinocyte proliferation	3/340	47/18614	0.05431197	0.1892253	0.1436784	ZFP36/SFN/HAS2	3
BP	GO:00	lung alveolus	3/340	47/18614	0.054	0.189	0.143	PKDCC/ACE/SLC7A11	3

	48286	development			3197	2253	6784		
BP	GO:0060711	labyrinthine layer development	3/340	47/18614	0.0543197	0.1892253	0.1436784	BMP5/JUNB/SOCS3	3
BP	GO:0140353	lipid export from cell	3/340	47/18614	0.0543197	0.1892253	0.1436784	PTGS2/IL1B/INHBA	3
BP	GO:1905314	semi-lunar valve development	3/340	47/18614	0.0543197	0.1892253	0.1436784	ROBO2/TGFB2/SLIT3	3
BP	GO:0055117	regulation of cardiac muscle contraction	4/340	78/18614	0.0544851	0.1896281	0.1439842	RYR2/ANK2/PIK3CG/CASQ2	4
BP	GO:0006090	pyruvate metabolic	5/340	112/18614	0.0546249	0.1899411	0.1442219	ZBTB20/ENO1/IER3/NR4A3/DDIT4	5

BP	GO:0051896	process regulation of protein kinase B signaling	7/340	186/18614	0.05500343	0.19026639	0.14446469	AKR1C2/PIK3CG/AKR1C3/NTRK2/CX3CR1/CX3CL1/CSF3	7
BP	GO:0002888	positive regulation of myeloid leukocyte mediated immunity	2/340	21/18614	0.05560644	0.19026639	0.14446469	CD177/STAP1	2
BP	GO:0006590	thyroid hormone generation	2/340	21/18614	0.05560644	0.19026639	0.14446469	DUOX1/DUOXA1	2

BP	GO:0007252	I-kappaB phosphorylation	2/340	21/18614	0.00556044	0.1902639	0.1444669	CX3CR1/CX3CL1	2
BP	GO:0010829	negative regulation of glucose transmembrane transport	2/340	21/18614	0.00556044	0.1902639	0.1444669	ACE/IL1B	2
BP	GO:0014856	skeletal muscle cell proliferation	2/340	21/18614	0.00556044	0.1902639	0.1444669	HGF/FOS	2
BP	GO:0016137	glycoside metabolic process	2/340	21/18614	0.00556044	0.1902639	0.1444669	AKR1C2/AKR1C3	2

BP	GO:0021756	striatum development	2/340	21/18614	0.0556044	0.1902639	0.1444669	SLC7A11/INHBA	2
BP	GO:0030728	ovulation	2/340	21/18614	0.0556044	0.1902639	0.1444669	PTGS2/INHBA	2
BP	GO:0033005	positive regulation of mast cell activation	2/340	21/18614	0.0556044	0.1902639	0.1444669	FGR/NR4A3	2
BP	GO:0043373	CD4-positive, alpha-beta T cell lineage commitment	2/340	21/18614	0.0556044	0.1902639	0.1444669	LY9/IL6	2
BP	GO:00	mitochondrial	2/340	21/18614	0.055	0.190	0.144	GCLM/DCN	2

	51882	depolarization			6044	2639	4669		
BP	GO:0060252	positive regulation of glial cell proliferation	2/340	21/18614	0.0556044	0.1902639	0.1444669	IL1B/IL6	2
BP	GO:0071498	cellular response to fluid shear stress	2/340	21/18614	0.0556044	0.1902639	0.1444669	PTGS2/HAS2	2
BP	GO:1903204	negative regulation of oxidative stress-induced neuron death	2/340	21/18614	0.0556044	0.1902639	0.1444669	SLC7A11/NR4A3	2

BP	GO:1903579	negative regulation of ATP metabolic process	2/340	21/18614	0.05560 44	0.19026 39	0.14446 69	IER3/DDIT4	2
BP	GO:2000319	regulation of T-helper 17 cell differentiation	2/340	21/18614	0.05560 44	0.19026 39	0.14446 69	NFKBIZ/JUNB	2
BP	GO:0015711	organic anion transport	13/340	433/18614	0.05561 64	0.19026 39	0.14446 69	PLIN2/PTGS2/SLCO4A1/ACE/SLC39A14/SLC7A11/IL1B/AQP9/SLC1A3/CFTR/SLC11A1/NTRK2/PLA2G2A	13
BP	GO:0032414	positive regulation of ion transmembrane	5/340	113/18614	0.05633 97	0.19256 55	0.14621 46	TRPC6/RYR2/ANK2/CFTR/CCL2	5

		transporter activity							
BP	GO:0002228	natural killer cell mediated immunity	4/340	79/18614	0.0566015	0.19328871	0.1467625	SLAMF7/CD96/RASGRP1/CD2	4
BP	GO:0006110	regulation of glycolytic process	3/340	48/18614	0.05718867	0.19458579	0.1477502	ZBTB20/IER3/DDIT4	3
BP	GO:0030850	prostate gland development	3/340	48/18614	0.05718867	0.19458579	0.1477502	SULF1/TNC/ALOX15B	3
BP	GO:0035987	endodermal cell differentiation	3/340	48/18614	0.05718867	0.19458579	0.1477502	ITGA4/COL8A1/INHBA	3

BP	GO:0070509	calcium ion import	3/340	48/18614	0.0571867	0.1945879	0.1477502	CXCL12/ACE/CCL2	3
BP	GO:0002573	myeloid leukocyte differentiation	8/340	227/18614	0.05744457	0.1952949	0.148287	JUN/FASN/JUNB/TNFRSF11B/TRIB1/FOS/INHBA/CSF3	8
BP	GO:2000377	regulation of reactive oxygen species metabolic process	6/340	150/18614	0.05750064	0.1953269	0.1483113	SLC18A2/CD177/DUOXA1/IER3/AKR1C3/FPR2	6
BP	GO:0002040	sprouting angiogenesis	7/340	188/18614	0.05763339	0.1955858	0.1485079	PTGS2/APLNR/EGR3/HMOX1/ADAMTS9/SEMA6A/NR4A1	7
BP	GO:00	positive regulation of	4/340	80/18614	0.058	0.198	0.151	CD177/FGR/CFTR/FGA	4

	45921	exocytosis			7616	8815	0103		
BP	GO:0051966	regulation of synaptic transmission, glutamatergic	4/340	80/18614	0.0587616	0.1988815	0.1510103	PTGS2/CNR1/TNR/CCL2	4
BP	GO:0070972	protein localization to endoplasmic reticulum	4/340	80/18614	0.0587616	0.1988815	0.1510103	RYR2/HYOU1/ANK2/SRPRB	4
BP	GO:0002221	pattern recognition receptor signaling pathway	7/340	189/18614	0.0589618	0.1993819	0.1513902	NAIP/NFKBIZ/TRIL/NLRC4/TNIP3/IRF1/CLEC4E	7

BP	GO:0046942	carboxylic acid transport	11/340	352/18614	0.0594157	0.2007388	0.1524205	PLIN2/PTGS2/SLCO4A1/ACE/SLC7A11/IL1B/AQP9/SLC1A3/SLC11A1/NTRK2/PLA2G2A	11
BP	GO:1990748	cellular detoxification	5/340	115/18614	0.0598614	0.2018922	0.1532962	HBB/PTGS2/DUOX1/NQO1/GSTT1	5
BP	GO:0006911	phagocytosis, engulfment	3/340	49/18614	0.0601124	0.2018922	0.1532962	STAP1/FCGR2B/MARCO	3
BP	GO:0015849	organic acid transport	11/340	353/18614	0.0603771	0.2018922	0.1532962	PLIN2/PTGS2/SLCO4A1/ACE/SLC7A11/IL1B/AQP9/SLC1A3/SLC11A1/NTRK2/PLA2G2A	11
BP	GO:0030449	regulation of complement activation	2/340	22/18614	0.0604458	0.2018922	0.1532962	IL1B/VSIG4	2
BP	GO:00	regulation of supero	2/340	22/18614	0.060	0.201	0.153	CD177/FPR2	2

	32928	xide anion generation			4458	892	2962		
BP	GO:0034104	negative regulation of tissue remodeling	2/340	22/18614	0.0604458	0.201892	0.1532962	TNFRSF11B/IL6	2
BP	GO:0035584	calcium-mediated signaling using intracellular calcium source	2/340	22/18614	0.0604458	0.201892	0.1532962	RYR2/VCAM1	2
BP	GO:0035812	renal sodium excretion	2/340	22/18614	0.0604458	0.201892	0.1532962	CORIN/ACE	2

BP	GO:0045723	positive regulation of fatty acid biosynthetic process	2/340	22/18614	0.00604458	0.2018920	0.15329962	PTGS2/IL1B	2
BP	GO:0046597	negative regulation of viral entry into host cell	2/340	22/18614	0.00604458	0.2018920	0.15329962	CH25H/PTX3	2
BP	GO:0050961	detection of temperature stimulus involved in	2/340	22/18614	0.00604458	0.2018920	0.15329962	CXCL12/ANO1	2

BP	GO:0060713	sensory perception labyrinthine layer morphogenesis	2/340	22/18614	0.06044458	0.201892	0.15352962	BMP5/SOCS3	2
BP	GO:1903589	positive regulation of blood vessel endothelial cell proliferation involved in sprouting angiogenesis	2/340	22/18614	0.06044458	0.201892	0.15352962	APLNR/HMOX1	2

BP	GO:0001890	placenta development	6/340	152/18614	0.0605422	0.2019654	0.1533519	BMP5/PTGS2/JUNB/KRT19/TPPP3/SOCS3	6
BP	GO:0021537	telencephalon development	9/340	270/18614	0.0605737	0.2019654	0.1533519	BTG2/CXCL12/ROBO2/TNR/TACC2/SLC7A11/INHBA/NTRK2/DNAH5	9
BP	GO:2000628	regulation of miRNA metabolic process	4/340	81/18614	0.0609652	0.2030993	0.1542081	TGFB2/EGR1/JUN/FOS	4
BP	GO:0043123	positive regulation of I-kappaB kinase/NF-kappaB signaling	7/340	192/18614	0.063058	0.2092096	0.1588524	PIM2/HMOX1/IL1B/CTH/TNFSF10/S100A12/CX3CL1	7

BP	GO:0090276	regulation of peptide hormone secretion	7/340	192/18614	0.063058	0.2092096	0.1588524	CNR1/CCL5/IL1B/ANO1/CFTR/IL6/FGA	7
BP	GO:0001914	regulation of T cell mediated cytotoxicity	3/340	50/18614	0.0631307	0.2092096	0.1588524	CD1C/CD1E/FCGR2B	3
BP	GO:0010862	positive regulation of pathway-restricted SMAD protein phosphorylation	3/340	50/18614	0.0631307	0.2092096	0.1588524	BMP5/TGFB2/INHBA	3

BP	GO:0018198	peptidyl-cysteine modification	3/340	50/18614	0.06313 07	0.20920 96	0.15885 24	ACE/ZDHHC9/S100A8	3
BP	GO:0042304	regulation of fatty acid biosynthetic process	3/340	50/18614	0.06313 07	0.20920 96	0.15885 24	TRIB3/PTGS2/IL1B	3
BP	GO:0014032	neural crest cell development	4/340	82/18614	0.06321 2	0.20929 72	0.15891 89	SEMA3D/SEMA6D/SEMA6A/SEMA3G	4
BP	GO:0043406	positive regulation of MAP kinase activity	5/340	117/18614	0.06350 59	0.21008 78	0.15951 92	PIK3CG/FGD2/IL1B/RASGRP1/S100A12	5

BP	GO:0002218	activation of innate immune response	8/340	233/18614	0.064832 9	0.213174 8	0.161863 2	NAIP/NFKBIZ/TRIL/NLRC4/PYHIN1/TNIP3/IRF1/CLEC4E	8
BP	GO:0071560	cellular response to transforming growth factor beta stimulus	9/340	274/18614	0.065162 5	0.213174 8	0.161863 2	LTBP4/LTBP2/TGFB2/JUN/NFKBIZ/DDR2/FOS/ASPN/CX3CR1	9
BP	GO:0008037	cell recognition	6/340	155/18614	0.065271 6	0.213174 8	0.161863 2	FUT3/ROBO2/VCAN/CNR1/CD5/PTX3	6
BP	GO:0002363	alpha-beta T cell lineage	2/340	23/18614	0.065425 5	0.213174 8	0.161863 2	LY9/IL6	2

BP	GO:0003177	commitment pulmonary valve development	2/340	23/18614	0.06542 55	0.21317 48	0.16186 32	ROBO2/TGFB2	2
BP	GO:0003283	atrial septum development	2/340	23/18614	0.06542 55	0.21317 48	0.16186 32	ANK2/TGFB2	2
BP	GO:0006883	intracellular sodium ion homeostasis	2/340	23/18614	0.06542 55	0.21317 48	0.16186 32	SCNN1G/SLC1A3	2
BP	GO:0006925	inflammatory cell apoptotic process	2/340	23/18614	0.06542 55	0.21317 48	0.16186 32	SLC7A11/IL6	2

BP	GO:0019835	cytolysis	2/340	23/18614	0.00654255	0.2131748	0.1618632	LYZ/PLA2G2A	2
BP	GO:0031065	positive regulation of histone deacetylation	2/340	23/18614	0.00654255	0.2131748	0.1618632	SDR16C5/APLNR	2
BP	GO:0032288	myelin assembly	2/340	23/18614	0.00654255	0.2131748	0.1618632	ANK2/CNTN1	2
BP	GO:0043171	peptide catabolic process	2/340	23/18614	0.00654255	0.2131748	0.1618632	ACE/ANPEP	2
BP	GO:0044062	regulation of excretion	2/340	23/18614	0.00654255	0.2131748	0.1618632	CORIN/ACE	2
BP	GO:00	transport of virus	2/340	23/18614	0.065	0.213	0.161	DPP4/SIGLEC1	2

	46794				0.0654255	0.2131748	0.1618632			
BP	GO:0050855	regulation of B cell receptor signaling pathway	2/340	23/18614	0.0654255	0.2131748	0.1618632	STAP1/FCGR2B		2
BP	GO:1904754	positive regulation of vascular associated smooth muscle cell migration	2/340	23/18614	0.0654255	0.2131748	0.1618632	DDR2/NR4A3		2
BP	GO:20	negative regulati	2/340	23/18614	0.065	0.213	0.161	BTG2/SLC6A4		2

	00178	on of neural precursor cell proliferation			4255	1748	8632		
BP	GO:0001570	vasculogenesis	4/340	83/18614	0.0655019	0.2131748	0.1618632	APLNR/JUNB/NTRK2/HAS2	4
BP	GO:0050871	positive regulation of B cell activation	4/340	83/18614	0.0655019	0.2131748	0.1618632	NFATC2/IL2RG/CD27/IL6	4
BP	GO:0009749	response to glucose	7/340	194/18614	0.0658829	0.2141854	0.1626305	ZBTB20/GCLM/EGR1/ACE/SLC39A14/ANO1/CFTR	7
BP	GO:0007188	adenylate cyclase-modulating G	8/340	234/18614	0.0661189	0.2141854	0.1626305	P2RY1/CNR1/GPR4/FPR1/MARCO/FPR2/CXCL10/CXCL9	8

		protein-coupled receptor signaling pathway							
BP	GO:0015872	dopamine transport	3/340	51/18614	0.0662058	0.2141854	0.1626305	SLC18A2/CXCL12/CNR1	3
BP	GO:0019083	viral transcription	3/340	51/18614	0.0662058	0.2141854	0.1626305	JUN/CCL5/ZFP36	3
BP	GO:0050885	neuromuscular process controlling balance	3/340	51/18614	0.0662058	0.2141854	0.1626305	PCDH15/TNR/SLC1A3	3

BP	GO:0061082	myeloid leukocyte cytokine production	3/340	51/18614	0.0662058	0.2141854	0.1626305	TGFB2/HMOX1/NR4A3	3
BP	GO:1904707	positive regulation of vascular associated smooth muscle cell proliferation	3/340	51/18614	0.0662058	0.2141854	0.1626305	JUN/DDR2/NR4A3	3
BP	GO:1903531	negative regulation of secretion by cell	6/340	156/18614	0.0668949	0.2162312	0.1641839	P2RY1/CNR1/HMOX1/IL1B/FCGR2B/INHBA	6

BP	GO:0060348	bone development	8/340	235/18614	0.0674206	0.2177456	0.1653338	FREM1/CBS/DDR2/SH3PXD2B/ALPL/SULF1/OGN/HAS2	8
BP	GO:0006869	lipid transport	13/340	447/18614	0.0678138	0.21883	0.1661572	PLIN2/PTGS2/ABCA9/ABCA6/ABCA8/SLCO4A1/ACE/IL1B/AQP9/LIPG/INHBA/CFTR/PLA2G2A	13
BP	GO:0001678	intracellular glucose homeostasis	6/340	157/18614	0.0685416	0.2209917	0.1677985	ZBTB20/GCLM/ACE/SLC39A14/ANO1/CFTR	6
BP	GO:0002762	negative regulation of myeloid leukocyte differentiation	3/340	52/18614	0.0693484	0.2226509	0.1690584	TNFRSF11B/TRIB1/INHBA	3
BP	GO:0007157	heterophilic cell-cell adhesi	3/340	52/18614	0.0693484	0.2226509	0.1690584	HMCN1/VCAM1/SELE	3

		on via plasma membrane cell adhesion molecules								
BP	GO:0032692	negative regulation of interleukin-1 production	3/340	52/18614	0.0693484	0.2226509	0.1690584	IL1R2/CX3CR1/CX3CL1		3
BP	GO:0046638	positive regulation of alpha-beta T cell differentiation	3/340	52/18614	0.0693484	0.2226509	0.1690584	NFKBIZ/IL2RG/CD83		3

BP	GO:0072599	establishment of protein localization to endoplasmic reticulum	3/340	52/18614	0.0693484	0.2226509	0.1690584	RYR2/HYOU1/SRPRB	3
BP	GO:0032412	regulation of monoatomic ion transmembrane transporter activity	9/340	278/18614	0.0699657	0.2236409	0.16981	TRPC6/RYR2/FXYD1/KCNAB1/ANK2/GEM/CASQ2/CFTR/CCL2	9
BP	GO:0098742	cell-cell adhesion via plasma-	9/340	278/18614	0.0699657	0.2236409	0.16981	PCDH15/ROBO2/TGFB2/CD177/HMCN1/VCAM1/FAT3/CX3CL1/SELE	9

		membrane adhesion molecules							
BP	GO:0006096	glycolytic process	4/340	85/18614	0.0702098	0.2236409	0.16981	ZBTB20/ENO1/IER3/DDIT4	4
BP	GO:0006730	one-carbon metabolic process	2/340	24/18614	0.0705369	0.2236409	0.16981	SHMT2/MTHFD2	2
BP	GO:0014850	response to muscle activity	2/340	24/18614	0.0705369	0.2236409	0.16981	RYR2/CAPN3	2
BP	GO:0035458	cellular response to interferon-beta	2/340	24/18614	0.0705369	0.2236409	0.16981	PYHIN1/IRF1	2

BP	GO:0042438	melanin biosynthetic process	2/340	24/18614	0.0705369	0.2236409	0.16981	TRPC1/SLC7A11	2
BP	GO:0043369	CD4-positive or CD8-positive, alpha-beta T cell lineage commitment	2/340	24/18614	0.0705369	0.2236409	0.16981	LY9/IL6	2
BP	GO:0045932	negative regulation of muscle contraction	2/340	24/18614	0.0705369	0.2236409	0.16981	PTGS2/PIK3CG	2

BP	GO:0051043	regulation of membrane protein ectodomain proteolysis	2/340	24/18614	0.07053 69	0.22364 09	0.16981	TIMP4/IL1B	2
BP	GO:0060143	positive regulation of syncytium formation by plasma membrane fusion	2/340	24/18614	0.07053 69	0.22364 09	0.16981	NFATC2/CXCL9	2
BP	GO:0060571	morphogenesis of an epithelial fold	2/340	24/18614	0.07053 69	0.22364 09	0.16981	BMP5/SULF1	2

BP	GO:0071676	negative regulation of mononuclear cell migration	2/340	24/18614	0.0705369	0.2236409	0.16981	BMP5/CCL2	2
BP	GO:0098581	detection of external biotic stimulus	2/340	24/18614	0.0705369	0.2236409	0.16981	NAIP/NLRC4	2
BP	GO:1900543	negative regulation of purine nucleotide metabolic process	2/340	24/18614	0.0705369	0.2236409	0.16981	IER3/DDIT4	2

BP	GO:0030072	peptide hormone secretion	8/340	238/18614	0.07142	0.2262525	0.171793	CNR1/CCL5/IL1B/ANO1/CPE/CFTR/IL6/FGA	8
BP	GO:0071559	response to transforming growth factor beta	9/340	280/18614	0.0724481	0.2287457	0.1736861	LTBP4/LTBP2/TGFB2/JUN/NFKBIZ/DDR2/FOS/ASPN/CX3CR1	9
BP	GO:0035176	social behavior	3/340	53/18614	0.0725573	0.2287457	0.1736861	NRXN3/CX3CR1/SLC6A4	3
BP	GO:0045744	negative regulation of G protein-coupled receptor signalin	3/340	53/18614	0.0725573	0.2287457	0.1736861	APLNR/CCL5/RGS13	3

		g pathway							
BP	GO:0071622	regulation of granulocyte chemotaxis	3/340	53/18614	0.07255573	0.22874457	0.17368861	CCL5/DPP4/RARRES2	3
BP	GO:1902930	regulation of alcohol biosynthetic process	3/340	53/18614	0.07255573	0.22874457	0.17368861	P2RY1/BMP5/DHCR7	3
BP	GO:0006757	ATP generation from ADP	4/340	86/18614	0.07262672	0.22874457	0.17368861	ZBTB20/ENO1/IER3/DDIT4	4
BP	GO:0008306	associative learning	4/340	86/18614	0.07262672	0.22874457	0.17368861	BTG2/TNR/SLC7A11/FOS	4

BP	GO:0010876	lipid localization	14/340	497/18614	0.0732298	0.230453	0.1749825	PLIN2/PTGS2/ABCA9/ABCA6/ABCA8/SLCO4A1/ACE/IL1B/AQP9/LIPG/INHBA/CFTR/IL6/PLA2G2A	14
BP	GO:0006066	alcohol metabolic process	11/340	366/18614	0.0738112	0.2318997	0.1760881	P2RY1/AKR1C2/BMP5/IDH1/EPHX2/SDR16C5/DHCR7/AKR1C3/SULT1B1/CFTR/CH25H	11
BP	GO:0045860	positive regulation of protein kinase activity	11/340	366/18614	0.0738112	0.2318997	0.1760881	TGFB2/EGR1/PIK3CG/CCL5/DDR2/ACE/FGD2/IL1B/RASGRP1/SLC11A1/S100A12	11
BP	GO:0048708	astrocyte differentiation	4/340	87/18614	0.0750866	0.2349543	0.1784003	IL1B/S100A8/IL6/MT1X	4
BP	GO:0022612	gland morphogenesis	5/340	123/18614	0.0751712	0.2349543	0.1784003	HGF/TGFB2/SULF1/TNC/IL6	5
BP	GO:00	antigen processing	2/340	25/18614	0.075	0.234	0.178	CD1C/CD1E	2

	02475	and presentation via MHC class Ib			7736	9543	4003		
BP	GO:0003148	outflow tract septum morphogenesis	2/340	25/18614	0.0757736	0.2349543	0.1784003	ROBO2/TGFB2	2
BP	GO:0006582	melanin metabolic process	2/340	25/18614	0.0757736	0.2349543	0.1784003	TRPC1/SLC7A11	2
BP	GO:0006760	folic acid-containing compound metabolic	2/340	25/18614	0.0757736	0.2349543	0.1784003	SHMT2/MTHFD2	2

		process							
BP	GO:0016048	detection of temperature stimulus	2/340	25/18614	0.0757736	0.2349543	0.1784003	CXCL12/ANO1	2
BP	GO:0018904	ether metabolic process	2/340	25/18614	0.0757736	0.2349543	0.1784003	EPHX2/FASN	2
BP	GO:0032693	negative regulation of interleukin-10 production	2/340	25/18614	0.0757736	0.2349543	0.1784003	FCGR2B/IDO1	2
BP	GO:0033233	regulation of protein sumoylation	2/340	25/18614	0.0757736	0.2349543	0.1784003	EGR1/CAPN3	2

BP	GO:0043302	positive regulation of leukocyte degranulation	2/340	25/18614	0.07577736	0.2349543	0.1784003	CD177/FGR	2
BP	GO:0045649	regulation of macrophage differentiation	2/340	25/18614	0.07577736	0.2349543	0.1784003	TRIB1/INHBA	2
BP	GO:0045980	negative regulation of nucleotide metabolic process	2/340	25/18614	0.07577736	0.2349543	0.1784003	IER3/DDIT4	2
BP	GO:00	regulation of hormon	2/340	25/18614	0.075	0.234	0.178	BMP5/EGR1	2

	46885	e biosynthetic process			7736	9543	4003		
BP	GO:1905063	regulation of vascular associated smooth muscle cell differentiation	2/340	25/18614	0.0757736	0.2349543	0.1784003	NFATC2/CTH	2
BP	GO:0014009	glial cell proliferation	3/340	54/18614	0.0758315	0.2349543	0.1784003	IL1B/IL6/CX3CL1	3
BP	GO:0071320	cellular response to cAMP	3/340	54/18614	0.0758315	0.2349543	0.1784003	PIK3CG/AQP9/CFTR	3

BP	GO:0043542	endothelial cell migration	9/340	283/18614	0.0762731	0.2361305	0.1792934	PTGS2/PIK3CG/EGR3/DPP4/HMOX1/DCN/ADAMTS9/NR4A1/S100A12	9
BP	GO:0016052	carbohydrate catabolic process	6/340	162/18614	0.0771255	0.2384846	0.1810808	ZBTB20/ENO1/IER3/DDIT4/CHIT1/MGAM	6
BP	GO:0032411	positive regulation of transporter activity	5/340	124/18614	0.0772212	0.2384846	0.1810808	TRPC6/RYR2/ANK2/CFTR/CCL2	5
BP	GO:0097237	cellular response to toxic substance	5/340	124/18614	0.0772212	0.2384846	0.1810808	HBB/PTGS2/DUOX1/NQO1/GSTT1	5
BP	GO:00	stem cell	4/340	88/18614	0.077	0.239	0.181	SEMA3D/SEMA6D/SEMA6A/SEMA3G	4

	48864	development			5876	2285	6457		
BP	GO:0050672	negative regulation of lymphocyte proliferation	4/340	88/18614	0.0775876	0.2392285	0.1816457	VSIG4/FCGR2B/PLA2G2A/IDO1	4
BP	GO:0050796	regulation of insulin secretion	6/340	163/18614	0.0789121	0.2429192	0.1844481	CNR1/CCL5/IL1B/ANO1/CFTR/IL6	6
BP	GO:0051100	negative regulation of binding	6/340	163/18614	0.0789121	0.2429192	0.1844481	P2RY1/JUN/ITGA4/ACE/HMOX1/CCL23	6
BP	GO:0003229	ventricular cardiac muscle tissue	3/340	55/18614	0.0791699	0.2431236	0.1846032	RYR2/TGFB2/ADAMTS9	3

		development							
BP	GO:0042743	hydrogen peroxide metabolic process	3/340	55/18614	0.0791699	0.2431236	0.1846032	HBB/DUOX1/DUOXA1	3
BP	GO:0051703	biological process involved in intraspecies interaction between organisms	3/340	55/18614	0.0791699	0.2431236	0.1846032	NRXN3/CX3CR1/SLC6A4	3

BP	GO:0001892	embryonic placenta development	4/340	89/18614	0.008013	0.24547486	0.18639 14	BMP5/JUNB/KRT19/SOCS3	4
BP	GO:0032945	negative regulation of mononuclear cell proliferation	4/340	89/18614	0.008013	0.24547486	0.18639 14	VSIG4/FCGR2B/PLA2G2A/IDO1	4
BP	GO:2000177	regulation of neural precursor cell proliferation	4/340	89/18614	0.008013	0.24547486	0.18639 14	BTG2/CX3CR1/CX3CL1/SLC6A4	4
BP	GO:0001911	negative regulation of	2/340	26/18614	0.0811294	0.2459698	0.1867643	FCGR2B/CX3CR1	2

		leukocyte mediated cytotoxicity							
BP	GO:0002407	dendritic cell chemotaxis	2/340	26/18614	0.0811294	0.24596 98	0.18676 43	PIK3CG/CCL5	2
BP	GO:0002433	immune response-regulating cell surface receptor signaling pathway involved in	2/340	26/18614	0.08112 94	0.24596 98	0.18676 43	FGR/FCGR2B	2

		phagocytosis							
BP	GO:0002438	acute inflammatory response to antigenic stimulus	2/340	26/18614	0.08112294	0.24596698	0.18676643	CNR1/FCGR2B	2
BP	GO:0002726	positive regulation of T cell cytokine production	2/340	26/18614	0.08112294	0.24596698	0.18676643	IL1B/IL6	2
BP	GO:0010259	multicellular organism aging	2/340	26/18614	0.08112294	0.24596698	0.18676643	ALPL/INHBA	2

BP	GO:0010954	positive regulation of protein processing	2/340	26/18614	0.0811294	0.2459698	0.1867643	ENO1/NLRC4	2
BP	GO:0038096	Fc-gamma receptor signaling pathway involved in phagocytosis	2/340	26/18614	0.0811294	0.2459698	0.1867643	FGR/FCGR2B	2
BP	GO:0044550	secondary metabolite biosynthetic process	2/340	26/18614	0.0811294	0.2459698	0.1867643	TRPC1/SLC7A11	2

BP	GO:0050857	positive regulation of antigen receptor-mediated signaling pathway	2/340	26/18614	0.08112 94	0.24596 98	0.18676 43	STAP1/TRAT1	2
BP	GO:0060740	prostate gland epithelium morphogenesis	2/340	26/18614	0.08112 94	0.24596 98	0.18676 43	SULF1/TNC	2
BP	GO:1903306	negative regulation of regulated secretory	2/340	26/18614	0.08112 94	0.24596 98	0.18676 43	HMOX1/FCGR2B	2

		pathway							
BP	GO:1903901	negative regulation of viral life cycle	2/340	26/18614	0.081121294	0.245969698	0.186767643	CH25H/PTX3	2
BP	GO:0002708	positive regulation of lymphocyte mediated immunity	5/340	126/18614	0.0814411	0.2462236	0.18696965	CD1C/CD1E/IL1B/RASGRP1/IL6	5
BP	GO:0006022	aminoglycan metabolic process	5/340	126/18614	0.0814411	0.2462236	0.18696965	IL1B/LYVE1/DCN/CHIT1/HAS2	5
BP	GO:00	cellular component	5/340	126/18614	0.081	0.246	0.186	ANK2/CNTN1/CAPN3/KRT19/MYH11	5

	10927	assembly involved in morphogenesis			411	236	9665		
BP	GO:0009306	protein secretion	11/340	373/18614	0.0817775	0.2471408	0.1876535	LTBP2/TGFB2/CNR1/CCL5/IL1B/MYH10/ANO1/CFTR/SAA1/IL6/FGA	11
BP	GO:0001959	regulation of cytokine-mediated signaling pathway	6/340	165/18614	0.0825548	0.2485965	0.1887588	NAIP/CCL5/SLIT3/STAP1/IL1R2/IL6	6
BP	GO:0007187	G protein-coupled receptor	3/340	56/18614	0.0825713	0.2485965	0.1887588	CNR1/HRH2/CCL2	3

		signaling pathway, coupled to cyclic nucleotide second messenger							
BP	GO:0038066	p38MAPK cascade	3/340	56/18614	0.0825713	0.2485965	0.1887588	HGF/ZFP36/IL1B	3
BP	GO:0070231	T cell apoptotic process	3/340	56/18614	0.0825713	0.2485965	0.1887588	CCL5/CD27/IDO1	3
BP	GO:0033555	multicellular organismal respon	4/340	90/18614	0.08271133	0.2485965	0.1887588	DPP4/AQP9/NR4A2/IDO1	4

		se to stress							
BP	GO:0048525	negative regulation of viral process	4/340	90/18614	0.08271733	0.24859565	0.18875588	CCL5/ZFP36/CH25H/PTX3	4
BP	GO:0051262	protein tetramerization	4/340	90/18614	0.08271733	0.24859565	0.18875588	SHMT2/MS4A1/CTH/GBP5	4
BP	GO:0035592	establishment of protein localization to extracellular region	11/340	374/18614	0.08295548	0.2491259	0.18916608	LTBP2/TGFB2/CNR1/CCL5/IL1B/MYH10/ANO1/CFTR/SAA1/IL6/FGA	11
BP	GO:0048863	stem cell differentiation	8/340	247/18614	0.08427501	0.25253549	0.19174492	FRZB/TGFB2/SEMA3D/SEMA6D/ACE/ZFP36/SEMA6A/SEMA3G	8

BP	GO:0071695	anatomical structure maturation	8/340	247/18614	0.0842701	0.2525349	0.1917492	RPS6KA2/TGFB2/GLDN/TFCP2L1/CFTR/CX3CR1/CX3CL1/NR4A2	8
BP	GO:0022898	regulation of transmembrane transporter activity	9/340	289/18614	0.0842888	0.2525349	0.1917492	TRPC6/RYR2/FXYD1/KCNAB1/ANK2/GEM/CASQ2/CFTR/CCL2	9
BP	GO:2000241	regulation of reproductive process	7/340	206/18614	0.0844162	0.2527182	0.1918884	P2RY1/RPS6KA2/PTGDS/CNR1/HPGDS/SULF1/INHBA	7
BP	GO:0060021	roof of mouth development	4/340	91/18614	0.0853372	0.2552748	0.1938296	ARID5B/TGFB2/PKDCC/INHBA	4

BP	GO:0042593	glucose homeostasis	8/340	248/18614	0.0857768	0.2554373	0.1939953	ZBTB20/GCLM/CNR1/ACE/SLC39A14/ANO1/CFTR/IL6	8
BP	GO:0001706	endoderm formation	3/340	57/18614	0.0860347	0.2554373	0.1939953	ITGA4/COL8A1/INHBA	3
BP	GO:0007566	embryo implantation	3/340	57/18614	0.0860347	0.2554373	0.1939953	PTGS2/IL1B/TPPP3	3
BP	GO:0043470	regulation of carbohydrate catabolic process	3/340	57/18614	0.0860347	0.2554373	0.1939953	ZBTB20/IER3/DDIT4	3
BP	GO:0050433	regulation of catecholamine	3/340	57/18614	0.0860347	0.2554373	0.1939953	P2RY1/CXCL12/CNR1	3

		secretion							
BP	GO:0007623	circadian rhythm	7/340	207/18614	0.0860829	0.2554373	0.1939953	PTGDS/EGR1/EGR3/JUND/NR1D2/NTRK2/SLC6A4	7
BP	GO:0001963	synaptic transmission, dopaminergic	2/340	27/18614	0.0865598	0.2554373	0.1939953	PTGS2/SLC6A4	2
BP	GO:0009081	branched-chain amino acid metabolic process	2/340	27/18614	0.0865598	0.2554373	0.1939953	HMGCLL1/BCAT1	2
BP	GO:0021544	subpallium development	2/340	27/18614	0.0865598	0.2554373	0.1939953	SLC7A11/INHBA	2

BP	GO:0032740	positive regulation of interleukin-17 production	2/340	27/18614	0.086598	0.2554373	0.193953	LY9/IL6	2
BP	GO:0042730	fibrinolysis	2/340	27/18614	0.086598	0.2554373	0.193953	SERPINF2/FGA	2
BP	GO:0042832	defense response to protozoan	2/340	27/18614	0.086598	0.2554373	0.193953	SLC11A1/GBP4	2
BP	GO:0048520	positive regulation of behavior	2/340	27/18614	0.086598	0.2554373	0.193953	SGIP1/NR4A3	2
BP	GO:0051953	negative regulation of	2/340	27/18614	0.086598	0.2554373	0.193953	P2RY1/CNR1	2

		amine transport							
BP	GO:0060669	embryonic placenta morphogenesis	2/340	27/18614	0.086598	0.2554373	0.1939953	BMP5/SOCS3	2
BP	GO:0062149	detection of stimulus involved in sensory perception of pain	2/340	27/18614	0.086598	0.2554373	0.1939953	CXCL12/ANO1	2
BP	GO:0071280	cellular response to copper ion	2/340	27/18614	0.086598	0.2554373	0.1939953	MT1X/MT1M	2

BP	GO:2000108	positive regulation of leukocyte apoptotic process	2/340	27/18614	0.0865598	0.2554373	0.1939953	CCL5/IDO1	2
BP	GO:0033500	carbohydrate homeostasis	8/340	249/18614	0.0872992	0.2573063	0.1953721	ZBTB20/GCLM/CNR1/ACE/SLC39A14/ANO1/CFTR/IL6	8
BP	GO:0002758	innate immune response-activating signaling pathway	7/340	209/18614	0.0894724	0.2629498	0.1996572	NAIP/NFKBIZ/TRIL/NLRC4/TNIP3/IRF1/CLEC4E	7

BP	GO:0048167	regulation of synaptic plasticity	7/340	209/18614	0.0894724	0.2629498	0.1996572	PTGS2/EGR2/TNR/ACE/NTRK2/CX3CR1/CX3CL1	7
BP	GO:0001836	release of cytochrome c from mitochondria	3/340	58/18614	0.08955589	0.2629498	0.1996572	HGF/SFN/TNFSF10	3
BP	GO:0006956	complement activation	3/340	58/18614	0.08955589	0.2629498	0.1996572	IL1B/VSIG4/C1R	3
BP	GO:0099024	plasma membrane invagination	3/340	58/18614	0.08955589	0.2629498	0.1996572	STAP1/FCGR2B/MARCO	3
BP	GO:1900542	regulation of purine nucleoti	4/340	93/18614	0.09070554	0.2661111	0.2020575	ZBTB20/ENO1/IER3/DDIT4	4

		de metabolic process							
BP	GO:0002064	epithelial cell development	7/340	210/18614	0.0911951	0.2671286	0.2028302	FRZB/BMP5/FASN/IL1B/GPR4/SFN/TFCP2L1	7
BP	GO:0050806	positive regulation of synaptic transmission	6/340	170/18614	0.0920637	0.2671286	0.2028302	PTGS2/TNR/SLC1A3/NTRK2/CX3CR1/CCL2	6
BP	GO:0014072	response to isoquinoline alkaloid	2/340	28/18614	0.0921737	0.2671286	0.2028302	CNR1/FOSB	2
BP	GO:0016540	protein autoprocessing	2/340	28/18614	0.0921737	0.2671286	0.2028302	CTSE/PCSK2	2

BP	GO:0031664	regulation of lipopolysaccharide-mediated signaling pathway	2/340	28/18614	0.0921737	0.2671286	0.2028302	TRIB1/CX3CL1	2
BP	GO:0032703	negative regulation of interleukin-2 production	2/340	28/18614	0.0921737	0.2671286	0.2028302	ZFP36/VSIG4	2
BP	GO:0043278	response to morphine	2/340	28/18614	0.0921737	0.2671286	0.2028302	CNR1/FOSB	2
BP	GO:00	positive regulation of	2/340	28/18614	0.092	0.267	0.202	SFN/ALOX15B	2

	45606	epidermal cell differentiation			1737	1286	8302		
BP	GO:0050951	sensory perception of temperature stimulus	2/340	28/18614	0.0921737	0.2671286	0.2028302	CXCL12/ANO1	2
BP	GO:0060512	prostate gland morphogenesis	2/340	28/18614	0.0921737	0.2671286	0.2028302	SULF1/TNC	2
BP	GO:0070269	pyroptosis	2/340	28/18614	0.0921737	0.2671286	0.2028302	NAIP/NLRC4	2
BP	GO:0150146	cell junction disassembly	2/340	28/18614	0.0921737	0.2671286	0.2028302	CX3CR1/CX3CL1	2

BP	GO:1900746	regulation of vascular endothelial growth factor signaling pathway	2/340	28/18614	0.09217 37	0.26712 86	0.20283 02	DCN/SEMA6A	2
BP	GO:1903055	positive regulation of extracellular matrix organization	2/340	28/18614	0.09217 37	0.26712 86	0.20283 02	DDR2/IL6	2
BP	GO:1903319	positive regulation of protein maturation	2/340	28/18614	0.09217 37	0.26712 86	0.20283 02	ENO1/NLRC4	2

BP	GO:1903671	negative regulation of sprouting angiogenesis	2/340	28/18614	0.0921737	0.2671286	0.2028302	ADAMTS9/SEMA6A	2
BP	GO:0016032	viral process	12/340	426/18614	0.0923525	0.2674434	0.2030692	JUN/CCL5/ST6GALNAC3/ZDHHC9/DPP4/ZFP36/SIGLEC1/ANPEP/CH25H/PTX3/CCL2/CCL8	12
BP	GO:0071692	protein localization to extracellular region	11/340	382/18614	0.0927731	0.2684573	0.2038391	LTBP2/TGFB2/CNR1/CCL5/IL1B/MYH10/ANO1/CFTR/SAA1/IL6/FGA	11
BP	GO:0001505	regulation of neurotransmitter levels	7/340	211/18614	0.0929363	0.2685071	0.2038769	P2RY1/SLC18A2/CSPG5/SLC1A3/MAOA/NR4A1/SLC6A4	7

BP	GO:0001913	T cell mediated cytotoxicity	3/340	59/18614	0.09314126	0.2685071	0.20387769	CD1C/CD1E/FCGR2B	3
BP	GO:0006611	protein export from nucleus	3/340	59/18614	0.09314126	0.2685071	0.20387769	EGR2/IL1B/SFN	3
BP	GO:0051289	protein homotetramerization	3/340	59/18614	0.09314126	0.2685071	0.20387769	SHMT2/CTH/GBP5	3
BP	GO:2001258	negative regulation of cation channel activity	3/340	59/18614	0.09314126	0.2685071	0.20387769	KCNAB1/GEM/CASQ2	3
BP	GO:0006140	regulation of nucleotide metabo	4/340	94/18614	0.09344488	0.26898329	0.20423382	ZBTB20/ENO1/IER3/DDIT4	4

		lic process							
BP	GO:0046031	ADP metabolic process	4/340	94/18614	0.09344488	0.26898829	0.20423382	ZBTB20/ENO1/IER3/DDIT4	4
BP	GO:0015850	organic hydroxy compound transport	9/340	296/18614	0.09425566	0.2711034	0.20584482	P2RY1/SLC18A2/CXCL12/ABCA8/CNR1/AQP9/LIPG/CFTR/SLC6A4	9
BP	GO:1904705	regulation of vascular associated smooth muscle cell proliferation	4/340	95/18614	0.09623313	0.27657742	0.21000022	JUN/DDR2/HMOX1/NR4A3	4

BP	GO:0050730	regulation of peptidyl-tyrosine phosphorylation	8/340	255/18614	0.0967627	0.276639	0.2100514	HGF/CNTN1/CCL5/ACE/CD3E/IL6/SOCS3/CSF3	8
BP	GO:0045604	regulation of epidermal cell differentiation	3/340	60/18614	0.0967847	0.276639	0.2100514	ZFP36/SFN/ALOX15B	3
BP	GO:0045620	negative regulation of lymphocyte differentiation	3/340	60/18614	0.0967847	0.276639	0.2100514	HMGB3/IRF1/INHBA	3
BP	GO:00	positive regulation of	3/340	60/18614	0.096	0.276	0.210	PTGS2/IL1B/SFN	3

	46824	nucleocytoplasmic transport			7847	639	0514		
BP	GO:0034219	carbohydrate transmembrane transport	5/340	133/18614	0.0970025	0.276639	0.2100514	TRIB3/ACE/IL1B/NR4A3/AQP9	5
BP	GO:0002052	positive regulation of neuroblast proliferation	2/340	29/18614	0.0978508	0.276639	0.2100514	CX3CR1/CX3CL1	2
BP	GO:0003156	regulation of animal organ formation	2/340	29/18614	0.0978508	0.276639	0.2100514	ROBO2/SULF1	2

BP	GO:0007263	nitric oxide mediated signal transduction	2/340	29/18614	0.0978508	0.2766639	0.2100514	CBS/FPR1	2
BP	GO:0010955	negative regulation of protein processing	2/340	29/18614	0.0978508	0.2766639	0.2100514	SERPINF2/IL1R2	2
BP	GO:0010996	response to auditory stimulus	2/340	29/18614	0.0978508	0.2766639	0.2100514	SLC1A3/CXCL10	2
BP	GO:0015813	L-glutamate transmembrane	2/340	29/18614	0.0978508	0.2766639	0.2100514	SLC7A11/SLC1A3	2

		transport							
BP	GO:0030431	sleep	2/340	29/18614	0.0978508	0.276639	0.2100514	PTGDS/FOS	2
BP	GO:0031342	negative regulation of cell killing	2/340	29/18614	0.0978508	0.276639	0.2100514	FCGR2B/CX3CR1	2
BP	GO:0035640	exploration behavior	2/340	29/18614	0.0978508	0.276639	0.2100514	TNR/DPP4	2
BP	GO:0044342	type B pancreatic cell proliferation	2/340	29/18614	0.0978508	0.276639	0.2100514	NR4A3/NR4A1	2
BP	GO:0060037	pharyngeal system development	2/340	29/18614	0.0978508	0.276639	0.2100514	BMP5/TGFB2	2

BP	GO:0061436	establishment of skin barrier	2/340	29/18614	0.0978508	0.276639	0.2100514	NFKBIZ/SFN	2
BP	GO:0072012	glomerulus vasculature development	2/340	29/18614	0.0978508	0.276639	0.2100514	EGR1/GPR4	2
BP	GO:1900017	positive regulation of cytokine production involved in inflammatory response	2/340	29/18614	0.0978508	0.276639	0.2100514	IL6/GBP5	2
BP	GO:19	negative regulati	2/340	29/18614	0.097	0.276	0.210	KCNAB1/CASQ2	2

	01017	on of potassium ion transmembrane transporter activity			8508	639	0514		
BP	GO:1903318	negative regulation of protein maturation	2/340	29/18614	0.0978508	0.276639	0.2100514	SERPINF2/IL1R2	2
BP	GO:1904385	cellular response to angiotensin	2/340	29/18614	0.0978508	0.276639	0.2100514	DDR2/INHBA	2
BP	GO:2001242	regulation of intrinsic apoptotic	6/340	173/18614	0.0980418	0.2769736	0.2103055	RTKN2/CXCL12/ENO1/HYOU1/PTGS2/S100A8	6

		signaling pathway							
BP	GO:0045637	regulation of myeloid cell differentiation	7/340	214/18614	0.0982711	0.2772105	0.2104853	HMGB3/TNFRSF11B/ZFP36/TRIB1/FOS/INHBA/CSF3	7
BP	GO:1901888	regulation of cell junction assembly	7/340	214/18614	0.0982711	0.2772105	0.2104853	ROBO2/APLNR/ACE/FMN1/IL1B/LRRN3/NTRK2	7
BP	GO:0002042	cell migration involved in sprouting angiogenesis	4/340	96/18614	0.0990523	0.2787795	0.2116885	PTGS2/EGR3/HMOX1/NR4A1	4

BP	GO:0070664	negative regulation of leukocyte proliferation	4/340	96/18614	0.0990523	0.278795	0.2116885	VSIG4/FCGR2B/PLA2G2A/IDO1	4
BP	GO:1903510	mucopolysaccharide metabolic process	4/340	96/18614	0.0990523	0.278795	0.2116885	IL1B/LYVE1/DCN/HAS2	4
BP	GO:0030216	keratinocyte differentiation	6/340	174/18614	0.1000795	0.2814781	0.2137257	KRT7/NFKBIZ/ZFP36/AKR1C3/SFN/ALOX15B	6
BP	GO:0015800	acidic amino acid transport	3/340	61/18614	0.1004839	0.2815766	0.2138005	SLC7A11/SLC1A3/NTRK2	3

BP	GO:0086065	cell communication involved in cardiac conduction	3/340	61/18614	0.10048 39	0.28157 66	0.21380 05	RYR2/ANK2/CASQ2	3
BP	GO:0098900	regulation of action potential	3/340	61/18614	0.10048 39	0.28157 66	0.21380 05	RYR2/ANK2/CNR1	3
BP	GO:1900024	regulation of substrate adhesion-dependent cell spreading	3/340	61/18614	0.10048 39	0.28157 66	0.21380 05	CSPG5/HAS2/FGA	3
BP	GO:19	reactive oxygen	3/340	61/18614	0.100	0.281	0.213	SLC18A2/DUOX1/DUOXA1	3

	03409	species biosynthetic process			4839	5766	8005		
BP	GO:0044282	small molecule catabolic process	11/340	388/18614	0.1005802	0.2816393	0.2138481	FAH/SHMT2/ENO1/CBS/HMGCLL1/NQO2/HDC/CNR1/AKR1C3/SULT1B1/IDO1	11
BP	GO:0000041	transition metal ion transport	4/340	97/18614	0.1019116	0.2847398	0.2162023	SLC39A14/LCN2/SLC11A1/CP	4
BP	GO:0010586	miRNA metabolic process	4/340	97/18614	0.1019116	0.2847398	0.2162023	TGFB2/EGR1/JUN/FOS	4
BP	GO:19	vascular associa	4/340	97/18614	0.101	0.284	0.216	JUN/DDR2/HMOX1/NR4A3	4

	90874	ted smooth muscle cell proliferation			9116	7398	2023		
BP	GO:0006694	steroid biosynthetic process	6/340	175/18614	0.1021394	0.2851672	0.2165268	BMP5/EGR1/DHCR7/AKR1C3/CFTR/CH25H	6
BP	GO:0002360	T cell lineage commitment	2/340	30/18614	0.1036237	0.2855459	0.2168144	LY9/IL6	2
BP	GO:0002825	regulation of T-helper 1 type immune response	2/340	30/18614	0.1036237	0.2855459	0.2168144	IL1B/SLC11A1	2
BP	GO:00	intracellular zinc ion	2/340	30/18614	0.103	0.285	0.216	SLC39A14/S100A8	2

	06882	homeostasis			6237	5459	8144		
BP	GO:0010447	response to acidic pH	2/340	30/18614	0.1036237	0.2855459	0.2168144	SCNN1G/GPR4	2
BP	GO:0015701	bicarbonate transport	2/340	30/18614	0.1036237	0.2855459	0.2168144	SLC39A14/CFTR	2
BP	GO:0034368	protein-lipid complex remodeling	2/340	30/18614	0.1036237	0.2855459	0.2168144	LIPG/PLA2G2A	2
BP	GO:0034369	plasma lipoprotein particle remodeling	2/340	30/18614	0.1036237	0.2855459	0.2168144	LIPG/PLA2G2A	2
BP	GO:0034698	response to gonadotropin	2/340	30/18614	0.1036237	0.2855459	0.2168144	GCLM/INHBA	2

BP	GO:0035774	positive regulation of insulin secretion involved in cellular response to glucose stimulus	2/340	30/18614	0.10362637	0.28554659	0.21681144	ANO1/CFTR	2
BP	GO:0042744	hydrogen peroxide catabolic process	2/340	30/18614	0.10362637	0.28554659	0.21681144	HBB/DUOX1	2
BP	GO:0043032	positive regulation of macrop	2/340	30/18614	0.10362637	0.28554659	0.21681144	STAP1/JUND	2

		hage activation							
BP	GO:0051938	L-glutamate import	2/340	30/18614	0.1036237	0.2855459	0.2168144	SLC7A11/SLC1A3	2
BP	GO:0090312	positive regulation of protein deacetylation	2/340	30/18614	0.1036237	0.2855459	0.2168144	SDR16C5/APLNR	2
BP	GO:0090330	regulation of platelet aggregation	2/340	30/18614	0.1036237	0.2855459	0.2168144	MMRN1/IL6	2
BP	GO:0097066	response to thyroid hormone	2/340	30/18614	0.1036237	0.2855459	0.2168144	GCLM/ACE	2
BP	GO:00	renal tubular	2/340	30/18614	0.103	0.285	0.216	CORIN/ACE	2

	97254	secretion			6237	5459	8144		
BP	GO:0097421	liver regeneration	2/340	30/18614	0.1036237	0.2855459	0.2168144	HMOX1/IL6	2
BP	GO:1902547	regulation of cellular response to vascular endothelial growth factor stimulus	2/340	30/18614	0.1036237	0.2855459	0.2168144	DCN/SEMA6A	2
BP	GO:0010634	positive regulation of epithelial cell migration	6/340	176/18614	0.1042215	0.2868269	0.217787	PTGS2/TGFB2/PIK3CG/JUN/HMOX1/HAS2	6

BP	GO:0090303	positive regulation of wound healing	3/340	62/18614	0.104239 1	0.286826 9	0.217787	DUOX1/SERPINF2/DDR2	3
BP	GO:1902476	chloride transmembrane transport	4/340	98/18614	0.104808 5	0.288185 6	0.218818 8	FXYD1/ANO1/SLC1A3/CFTR	4
BP	GO:0060759	regulation of response to cytokine stimulus	6/340	177/18614	0.106325 6	0.292146 3	0.221826 1	NAIP/CCL5/SLIT3/STAP1/IL1R2/IL6	6
BP	GO:0008277	regulation of G protein-coupled recepto	5/340	137/18614	0.106542 9	0.292321 8	0.221959 3	APLNR/CCL5/RGS5/SLC39A14/RGS13	5

		r signaling pathway							
BP	GO:0061041	regulation of wound healing	5/340	137/18614	0.10654 29	0.29232 18	0.22195 93	DUOX1/MMRN1/SERPINF2/DDR2/FGA	5
BP	GO:0051051	negative regulation of transport	13/340	483/18614	0.10681 72	0.29286 35	0.22237 07	P2RY1/KCNAB1/PTGS2/GEM/PKDCC/CNR1/CASQ2/ACE/TRAT1/HMOX1/IL1B/FCGR2B/INHBA	13
BP	GO:0060349	bone morphogenesis	4/340	99/18614	0.10774 27	0.29487 99	0.22390 17	FREM1/CBS/ALPL/HAS2	4
BP	GO:0016126	sterol biosynthetic process	3/340	63/18614	0.10804 9	0.29487 99	0.22390 17	DHCR7/CFTR/CH25H	3

BP	GO:0032757	positive regulation of interleukin-8 production	3/340	63/18614	0.1080049	0.2948799	0.2239017	IL1B/CD2/IL6	3
BP	GO:0060393	regulation of pathway-restricted SMAD protein phosphorylation	3/340	63/18614	0.1080049	0.2948799	0.2239017	BMP5/TGFB2/INHBA	3
BP	GO:0070542	response to fatty acid	3/340	63/18614	0.1080049	0.2948799	0.2239017	AKR1C2/PTGS2/AKR1C3	3
BP	GO:0006936	muscle contraction	10/340	349/18614	0.1081153	0.2948799	0.2239017	RYR2/ENO1/FXYD1/PTGS2/ANK2/PIK3CG/CASQ2/SULF1/MYH11/NR4A1	10

BP	GO:0032635	interleukin-6 production	6/340	178/18614	0.10845 17	0.29487 99	0.22390 17	ZBTB20/HGF/ORM1/IL1B/IL6/CX3CL1	6
BP	GO:0032675	regulation of interleukin-6 production	6/340	178/18614	0.10845 17	0.29487 99	0.22390 17	ZBTB20/HGF/ORM1/IL1B/IL6/CX3CL1	6
BP	GO:0006631	fatty acid metabolic process	11/340	394/18614	0.10876 59	0.29487 99	0.22390 17	TRIB3/AKR1C2/PTGS2/PTGDS/CNR1/HPGDS/FASN/IL1B/NR4A3/AKR1C3/ALOX15B	11
BP	GO:0008360	regulation of cell shape	5/340	138/18614	0.10899 77	0.29487 99	0.22390 17	P2RY1/FGD2/FGR/MYH10/CCL2	5
BP	GO:0046887	positive regulation of hormone	5/340	138/18614	0.10899 77	0.29487 99	0.22390 17	P2RY1/ANO1/INHBA/CFTR/FGA	5

		secretion							
BP	GO:0001958	endochondral ossification	2/340	31/18614	0.1094887	0.2948799	0.2239017	CBS/ALPL	2
BP	GO:0033137	negative regulation of peptidyl-serine phosphorylation	2/340	31/18614	0.1094887	0.2948799	0.2239017	HGF/DDIT4	2
BP	GO:0035767	endothelial cell chemotaxis	2/340	31/18614	0.1094887	0.2948799	0.2239017	EGR3/NR4A1	2
BP	GO:0036075	replacement ossification	2/340	31/18614	0.1094887	0.2948799	0.2239017	CBS/ALPL	2

BP	GO:0043304	regulation of mast cell degranulation	2/340	31/18614	0.109487	0.2948799	0.2239017	HMOX1/FGR	2
BP	GO:0060325	face morphogenesis	2/340	31/18614	0.109487	0.2948799	0.2239017	ARID5B/CRISPLD2	2
BP	GO:0060674	placenta blood vessel development	2/340	31/18614	0.109487	0.2948799	0.2239017	JUNB/SOCS3	2
BP	GO:0061437	renal system vasculature development	2/340	31/18614	0.109487	0.2948799	0.2239017	EGR1/GPR4	2
BP	GO:0061440	kidney vasculature development	2/340	31/18614	0.109487	0.2948799	0.2239017	EGR1/GPR4	2

BP	GO:0070570	regulation of neuron projection regeneration	2/340	31/18614	0.109487	0.2948799	0.2239017	HGF/TNR	2
BP	GO:0072525	pyridine-containing compound biosynthetic process	2/340	31/18614	0.109487	0.2948799	0.2239017	PSAT1/IDO1	2
BP	GO:0098901	regulation of cardiac muscle cell action potential	2/340	31/18614	0.109487	0.2948799	0.2239017	RYR2/ANK2	2

BP	GO:1903203	regulation of oxidative stress-induced neuron death	2/340	31/18614	0.1094887	0.2948799	0.2239017	SLC7A11/NR4A3	2
BP	GO:2000316	regulation of T-helper 17 type immune response	2/340	31/18614	0.1094887	0.2948799	0.2239017	NFKBIZ/JUNB	2
BP	GO:0001894	tissue homeostasis	8/340	263/18614	0.1102508	0.2965179	0.2251454	PCDH15/PTGS2/LYZ/NFKBIZ/SERPINA3/TNFRSF11B/IL6/AZGP1	8
BP	GO:0060249	anatomical structure	8/340	263/18614	0.1102508	0.2965179	0.2251454	PCDH15/PTGS2/LYZ/NFKBIZ/SERPINA3/TNFRSF11B/IL6/AZGP1	8

		homeostasis							
BP	GO:0001659	temperature homeostasis	6/340	179/18614	0.1105996	0.2967159	0.2252957	PTGS2/EGR1/CNR1/ALPL/IL1B/LCN2	6
BP	GO:0015698	inorganic anion transport	6/340	179/18614	0.1105996	0.2967159	0.2252957	FXYD1/SLC39A14/ANO1/SLC1A3/CFTR/SLC11A1	6
BP	GO:0019080	viral gene expression	4/340	100/18614	0.1107137	0.2967159	0.2252957	JUN/CCL5/ST6GALNAC3/ZFP36	4
BP	GO:0034446	substrate adhesion-dependent cell spreading	4/340	100/18614	0.1107137	0.2967159	0.2252957	ITGA4/CSPG5/HAS2/FGA	4
BP	GO:1901019	regulation of calcium ion	4/340	100/18614	0.1107137	0.2967159	0.2252957	RYR2/ANK2/GEM/CASQ2	4

		transmembrane transporter activity							
BP	GO:0048732	gland development	12/340	441/18614	0.1113381	0.2981796	0.2264072	FRZB/ARID5B/HGF/TGFB2/JUN/FASN/HMOX1/SULF1/TNC/TFCP2L1/ALOX15B/IL6	12
BP	GO:0007586	digestion	5/340	139/18614	0.111148	0.2983499	0.2265364	AKR1C2/HRH2/SERPINA3/PGC/CHIT1	5
BP	GO:0019731	antibacterial humoral response	3/340	64/18614	0.1119123	0.2986678	0.2267778	PGC/PI3/FGA	3
BP	GO:0032615	interleukin-12 production	3/340	64/18614	0.1119123	0.2986678	0.2267778	TIGIT/IRF1/IDO1	3

BP	GO:0032655	regulation of interleukin-12 production	3/340	64/18614	0.11191123	0.29866678	0.22677778	TIGIT/IRF1/IDO1	3
BP	GO:0051055	negative regulation of lipid biosynthetic process	3/340	64/18614	0.11191123	0.29866678	0.22677778	TRIB3/BMP5/AKR1C3	3
BP	GO:0070167	regulation of biomineral tissue development	4/340	101/18614	0.1137221	0.30318776	0.23020097	PKDCC/DDR2/CFTR/ASPN	4
BP	GO:0098657	import into cell	8/340	265/18614	0.11377761	0.30318776	0.23020097	SLC18A2/SCNN1G/ITGA4/MS4A1/SLC39A14/SLC7A11/SLC1A3/SLC6A4	8

BP	GO:2001235	positive regulation of apoptotic signaling pathway	5/340	140/18614	0.11398895	0.30318876	0.23020097	SRPX/S100A8/ATF3/INHBA/TNFSF10	5
BP	GO:0031346	positive regulation of cell projection organization	10/340	353/18614	0.11413372	0.30318876	0.23020097	CXCL12/BMP5/HGF/ROBO2/CNTN1/CNR1/DPYSL3/DDR2/NTRK2/CX3CL1	10
BP	GO:0002862	negative regulation of inflammatory response to antigen	2/340	32/18614	0.11543356	0.30318876	0.23020097	FGR/FCGR2B	2

		ic stimulus							
BP	GO:0007435	salivary gland morphogenesis	2/340	32/18614	0.11154356	0.3031876	0.23020097	HGF/TGFB2	2
BP	GO:0009072	aromatic amino acid metabolic process	2/340	32/18614	0.11154356	0.3031876	0.23020097	FAH/IDO1	2
BP	GO:0010644	cell communication by electrical coupling	2/340	32/18614	0.11154356	0.3031876	0.23020097	RYR2/CASQ2	2
BP	GO:00	regulation of macrop	2/340	32/18614	0.115	0.303	0.230	ALOX15B/PLA2G2A	2

	10743	hage derived foam cell differentiation			4356	1876	2097		
BP	GO:0010765	positive regulation of sodium ion transport	2/340	32/18614	0.1154356	0.3031876	0.2302097	FXYD1/CNTN1	2
BP	GO:0019883	antigen processing and presentation of endogenous antigen	2/340	32/18614	0.1154356	0.3031876	0.2302097	CD1C/CD1E	2
BP	GO:0034367	protein-containing comple	2/340	32/18614	0.1154356	0.3031876	0.2302097	LIPG/PLA2G2A	2

		x remodeling							
BP	GO:0034694	response to prostaglandin	2/340	32/18614	0.11543 56	0.30318 76	0.23020 97	AKR1C2/AKR1C3	2
BP	GO:0035456	response to interferon-beta	2/340	32/18614	0.11543 56	0.30318 76	0.23020 97	PYHIN1/IRF1	2
BP	GO:0035590	purinergic nucleotide receptor signaling pathway	2/340	32/18614	0.11543 56	0.30318 76	0.23020 97	P2RY1/P2RY14	2
BP	GO:0042558	pteridine-containing compo	2/340	32/18614	0.11543 56	0.30318 76	0.23020 97	SHMT2/MTHFD2	2

		und metabolic process							
BP	GO:0046825	regulation of protein export from nucleus	2/340	32/18614	0.1154356	0.3031876	0.2302097	IL1B/SFN	2
BP	GO:0050482	arachidonic acid secretion	2/340	32/18614	0.1154356	0.3031876	0.2302097	ACE/PLA2G2A	2
BP	GO:0051482	positive regulation of cytosolic calcium ion concentration involve	2/340	32/18614	0.11543556	0.30318776	0.23020097	GPR4/P2RY10	2

		d in phospholipase C-activating G protein-coupled signaling pathway							
BP	GO:0060055	angiogenesis involved in wound healing	2/340	32/18614	0.11543456	0.30318776	0.23020297	GPR4/CX3CL1	2
BP	GO:0061384	heart trabecula morphogenesis	2/340	32/18614	0.11543456	0.30318776	0.23020297	BMP5/TGFB2	2

BP	GO:0071353	cellular response to interleukin-4	2/340	32/18614	0.1154356	0.3031876	0.2302097	FASN/IL2RG	2
BP	GO:1903963	arachidonate transport	2/340	32/18614	0.1154356	0.3031876	0.2302097	ACE/PLA2G2A	2
BP	GO:0032535	regulation of cellular component size	10/340	354/18614	0.1156718	0.3033811	0.2303566	OLFM1/CXCL12/SEMA3D/SEMA6D/TNR/PLEKHH2/FMN1/SEMA6A/SEMA3G/CSF3	10
BP	GO:0010324	membrane invagination	3/340	65/18614	0.1158277	0.3033811	0.2303566	STAP1/FCGR2B/MARCO	3
BP	GO:0050854	regulation of antigen receptor-mediated	3/340	65/18614	0.1158277	0.3033811	0.2303566	STAP1/TRAT1/FCGR2B	3

		signaling pathway							
BP	GO:1905330	regulation of morphogenesis of an epithelium	3/340	65/18614	0.115827 7	0.303381 1	0.230356 6	HGF/SULF1/CXCL10	3
BP	GO:0001822	kidney development	9/340	310/18614	0.116165 7	0.304057 4	0.230870 2	ARID5B/ROBO2/TGFB2/EGR1/ACE/FMN1/GPR4/SULF1/HAS2	9
BP	GO:0002532	production of molecular mediator involved in inflammatory	4/340	102/18614	0.116764 1	0.304995 2	0.231582 2	SLC18A2/IL1R2/IL6/GBP5	4

BP	GO:0006165	response nucleoside diphosphate phosphorylation	4/340	102/18614	0.11167641	0.30499552	0.23158322	ZBTB20/ENO1/IER3/DDIT4	4
BP	GO:1990830	cellular response to leukemia inhibitory factor	4/340	102/18614	0.11167641	0.30499552	0.23158322	ARID5B/GCLM/CTH/SOCS3	4
BP	GO:0001655	urogenital system development	3/340	66/18614	0.11979941	0.31239938	0.2372	SULF1/TNC/ALOX15B	3
BP	GO:0045639	positive regulation of myeloid	4/340	103/18614	0.11984825	0.31239938	0.2372	TRIB1/FOS/INHBA/CSF3	4

		cell differentiation							
BP	GO:1990823	response to leukemia inhibitory factor	4/340	103/18614	0.11984 25	0.31239 38	0.23722	ARID5B/GCLM/CTH/SOCS3	4
BP	GO:0002828	regulation of type 2 immune response	2/340	33/18614	0.12146 44	0.31532 76	0.23942 75	IL6/IDO1	2
BP	GO:0033006	regulation of mast cell activation involved in immun	2/340	33/18614	0.12146 44	0.31532 76	0.23942 75	HMOX1/FGR	2

		e response se							
BP	GO:0036336	dendritic cell migration	2/340	33/18614	0.1214644	0.3153276	0.2394275	PIK3CG/CCL5	2
BP	GO:0045684	positive regulation of epidermis development	2/340	33/18614	0.1214644	0.3153276	0.2394275	SFN/ALOX15B	2
BP	GO:0061036	positive regulation of cartilage development	2/340	33/18614	0.1214644	0.3153276	0.2394275	PKDCC/BMP1	2
BP	GO:1901380	negative regulation of potassi	2/340	33/18614	0.1214644	0.3153276	0.2394275	KCNAB1/CASQ2	2

		um ion transmembrane transport							
BP	GO:0048469	cell maturation	6/340	184/18614	0.12166617	0.3156248	0.23965533	RPS6KA2/GLDN/TFCP2L1/CFTR/CX3CL1/NR4A2	6
BP	GO:0046939	nucleotide phosphorylation	4/340	104/18614	0.12229557	0.31817647	0.24204374	ZBTB20/ENO1/IER3/DDIT4	4
BP	GO:0045786	negative regulation of cell cycle	11/340	404/18614	0.12324426	0.31929213	0.24243372	BTG2/RPS6KA2/PTGS2/WEE1/PPP1R10/IER3/DTL/INHBA/ALOX15B/NR4A1/CCL2	11
BP	GO:0042446	hormone biosynthetic	3/340	67/18614	0.12381101	0.31898917	0.24289931	BMP5/EGR1/DUOX1	3

		process							
BP	GO:0045682	regulation of epidermis development	3/340	67/18614	0.1238101	0.3198917	0.2428931	ZFP36/SFN/ALOX15B	3
BP	GO:0060389	pathway-restricted SMAD protein phosphorylation	3/340	67/18614	0.1238101	0.3198917	0.2428931	BMP5/TGFB2/INHBA	3
BP	GO:1901224	positive regulation of NIK/NF-kappaB signaling	3/340	67/18614	0.1238101	0.3198917	0.2428931	RTKN2/IL1B/CD27	3

BP	GO:0042552	myelination	5/340	144/18614	0.124294 5	0.320708 3	0.243513 2	HGF/ANK2/CNTN1/EGR2/NTRK2	5
BP	GO:0098754	detoxification	5/340	144/18614	0.124294 5	0.320708 3	0.243513 2	HBB/PTGS2/DUOX1/NQO1/GSTT1	5
BP	GO:0021549	cerebellum development	4/340	105/18614	0.126103 2	0.325155 1	0.246889 6	CBS/CNTN1/FCGR2B/CD3E	4
BP	GO:0000289	nuclear-transcribed mRNA poly(A) tail shortening	2/340	34/18614	0.127568 5	0.326503 4	0.247913 3	BTG2/ZFP36	2
BP	GO:0006026	aminoglycan catabolic	2/340	34/18614	0.127568 5	0.326503 4	0.247913 3	LYVE1/CHIT1	2

		process							
BP	GO:0006536	glutamate metabolic process	2/340	34/18614	0.127568 5	0.326503 4	0.247913 3	GCLM/SLC7A11	2
BP	GO:0033238	regulation of amine metabolic process	2/340	34/18614	0.127568 5	0.326503 4	0.247913 3	SLC7A11/NR4A2	2
BP	GO:0036475	neuron death in response to oxidative stress	2/340	34/18614	0.127568 5	0.326503 4	0.247913 3	SLC7A11/NR4A3	2
BP	GO:00	synapse	2/340	34/18614	0.127	0.326	0.247	SYBU/CX3CR1	2

	600 74	maturation			56 85	50 34	91 33		
BP	GO:0060964	regulation of miRNA-mediated gene silencing	2/340	34/18614	0.127 56 85	0.326 50 34	0.247 91 33	ZFP36/IL6	2
BP	GO:0070633	transepithelial transport	2/340	34/18614	0.127 56 85	0.326 50 34	0.247 91 33	SLC1A3/CFTR	2
BP	GO:0086004	regulation of cardiac muscle cell contraction	2/340	34/18614	0.127 56 85	0.326 50 34	0.247 91 33	RYR2/ANK2	2
BP	GO:0090322	regulation of superoxide	2/340	34/18614	0.127 56 85	0.326 50 34	0.247 91 33	CD177/FPR2	2

		metabolic process							
BP	GO:0098801	regulation of renal system process	2/340	34/18614	0.12756585	0.32650034	0.24791133	CORIN/ACE	2
BP	GO:0015909	long-chain fatty acid transport	3/340	68/18614	0.12787445	0.32684475	0.24817746	PLIN2/ACE/PLA2G2A	3
BP	GO:0016925	protein sumoylation	3/340	68/18614	0.12787445	0.32684475	0.24817746	EGR1/EGR2/CAPN3	3
BP	GO:0009135	purine nucleoside diphosphate metabo	4/340	106/18614	0.12928844	0.33000086	0.25057749	ZBTB20/ENO1/IER3/DDIT4	4

		lic process								
BP	GO:0009179	purine ribonucleoside diphosphate metabolic process	4/340	106/18614	0.12928 44	0.33000 86	0.25057 49	ZBTB20/ENO1/IER3/DDIT4		4
BP	GO:0007272	ensheathment of neurons	5/340	146/18614	0.12960 36	0.33016 01	0.25068 98	HGF/ANK2/CNTN1/EGR2/NTRK2		5
BP	GO:0008366	axon ensheathment	5/340	146/18614	0.12960 36	0.33016 01	0.25068 98	HGF/ANK2/CNTN1/EGR2/NTRK2		5
BP	GO:0050714	positive regulation of protein	5/340	146/18614	0.12960 36	0.33016 01	0.25068 98	TGFB2/MYH10/ANO1/CFTR/FGA		5

BP	GO:0021700	secretion developmental maturation	9/340	319/18614	0.13160 97	0.33504 64	0.25444	RPS6KA2/TGFB2/GLDN/TFCP2L1/SYBU/CFTR/CX3CR1/CX3CL1/NR4A2	9
BP	GO:0009311	oligosaccharide metabolic process	3/340	69/18614	0.13198 59	0.33510 87	0.25444 74	FUT3/ST6GALNAC3/MGAM	3
BP	GO:0014015	positive regulation of gliogenesis	3/340	69/18614	0.13198 59	0.33510 87	0.25444 74	EGR2/IL1B/IL6	3
BP	GO:0032835	glomerulus development	3/340	69/18614	0.13198 59	0.33510 87	0.25444 74	EGR1/GPR4/SULF1	3
BP	GO:19	regulation of potassi	3/340	69/18614	0.131	0.335	0.254	KCNAB1/ANK2/CASQ2	3

	01016	um ion transmembrane transporter activity			9859	1087	4474		
BP	GO:0046822	regulation of nucleocytoplasmic transport	4/340	107/18614	0.1324989	0.3357675	0.2549476	PTGS2/IER3/IL1B/SFN	4
BP	GO:0071466	cellular response to xenobiotic stimulus	6/340	189/18614	0.1332482	0.3357675	0.2549476	NQO1/IL1B/CD69/SULT1B1/AOX1/S100A12	6
BP	GO:0045862	positive regulation of proteolysis	10/340	365/18614	0.1333089	0.3357675	0.2549476	TRIB3/ENO1/NLRC4/UACA/CAPN3/IL1B/PYHIN1/S100A8/TRIB1/TNFSF10	10

BP	GO:0072001	renal system development	9/340	320/18614	0.1333896	0.3357675	0.2549476	ARID5B/ROBO2/TGFB2/EGR1/ACE/FMN1/GPR4/SULF1/HAS2	9
BP	GO:0007095	mitotic G2 DNA damage checkpoint signaling	2/340	35/18614	0.1337431	0.3357675	0.2549476	IER3/DTL	2
BP	GO:0018149	peptide cross-linking	2/340	35/18614	0.1337431	0.3357675	0.2549476	DCN/PI3	2
BP	GO:0032633	interleukin-4 production	2/340	35/18614	0.1337431	0.3357675	0.2549476	CD83/CD3E	2
BP	GO:0032673	regulation of interleukin-4	2/340	35/18614	0.1337431	0.3357675	0.2549476	CD83/CD3E	2

		production							
BP	GO:0035025	positive regulation of Rho protein signal transduction	2/340	35/18614	0.13337431	0.3357675	0.2549476	GPR4/P2RY10	2
BP	GO:0035633	maintenance of blood-brain barrier	2/340	35/18614	0.13337431	0.3357675	0.2549476	PTGS2/IL6	2
BP	GO:0045777	positive regulation of blood pressure	2/340	35/18614	0.13337431	0.3357675	0.2549476	CNR1/ACE	2
BP	GO:0070670	response to interleukin-4	2/340	35/18614	0.13337431	0.3357675	0.2549476	FASN/IL2RG	2

BP	GO:0071398	cellular response to fatty acid	2/340	35/18614	0.1337431	0.3357675	0.2549476	AKR1C2/AKR1C3	2
BP	GO:0090050	positive regulation of cell migration involved in sprouting angiogenesis	2/340	35/18614	0.1337431	0.3357675	0.2549476	PTGS2/HMOX1	2
BP	GO:0098810	neurotransmitter reuptake	2/340	35/18614	0.1337431	0.3357675	0.2549476	SLC18A2/SLC6A4	2
BP	GO:1901099	negative regulation of	2/340	35/18614	0.1337431	0.3357675	0.2549476	IL1B/CX3CL1	2

		signal transduction in absence of ligand							
BP	GO:2001240	negative regulation of extrinsic apoptotic signaling pathway in absence of ligand	2/340	35/18614	0.1337431	0.3357675	0.2549476	IL1B/CX3CL1	2
BP	GO:0033138	positive regulation of peptidyl-serine	4/340	108/18614	0.135746	0.3405715	0.2585952	PTGS2/IL6/NTRK2/CSF3	4

		phosphorylation							
BP	GO:0002224	toll-like receptor signaling pathway	3/340	70/18614	0.1361431	0.3406706	0.2586705	NFKBIZ/TNIP3/IRF1	3
BP	GO:0030239	myofibril assembly	3/340	70/18614	0.1361431	0.3406706	0.2586705	CAPN3/KRT19/MYH11	3
BP	GO:0043627	response to estrogen	3/340	70/18614	0.1361431	0.3406706	0.2586705	TNFRSF11B/HMOX1/KRT19	3
BP	GO:0051881	regulation of mitochondrial membrane potential	3/340	70/18614	0.1361431	0.3406706	0.2586705	PYCR1/GCLM/DCN	3

BP	GO:0002793	positive regulation of peptide secretion	4/340	109/18614	0.13902 53	0.34765 44	0.26397 33	ANO1/S100A8/CFTR/FGA	4
BP	GO:0002068	glandular epithelial cell development	2/340	36/18614	0.13998 38	0.34818 75	0.26437 8	BMP5/FASN	2
BP	GO:0030224	monocyte differentiation	2/340	36/18614	0.13998 38	0.34818 75	0.26437 8	JUN/FASN	2
BP	GO:0045589	regulation of regulatory T cell differentiation	2/340	36/18614	0.13998 38	0.34818 75	0.26437 8	IL2RG/IRF1	2
BP	GO:00	regulation of fatty	2/340	36/18614	0.139	0.348	0.264	CNR1/NR4A3	2

	46320	acid oxidation			9838	1875	378		
BP	GO:0060218	hematopoietic stem cell differentiation	2/340	36/18614	0.1399838	0.3481875	0.264378	ACE/ZFP36	2
BP	GO:0086005	ventricular cardiac muscle cell action potential	2/340	36/18614	0.1399838	0.3481875	0.264378	RYR2/ANK2	2
BP	GO:1900368	regulation of post-transcriptional gene silencing by RNA	2/340	36/18614	0.1399838	0.3481875	0.264378	ZFP36/IL6	2

BP	GO:1903580	positive regulation of ATP metabolic process	2/340	36/18614	0.1399838	0.3481875	0.2643378	ZBTB20/ENO1	2
BP	GO:0071900	regulation of protein serine/threonine kinase activity	10/340	369/18614	0.1400607	0.3481875	0.2643378	TRIB3/CBS/PIK3CG/LAX1/FGD2/IL1B/SFN/RASGRP1/TRIB1/S100A12	10
BP	GO:0003208	cardiac ventricle morphogenesis	3/340	71/18614	0.1403448	0.3484391	0.2645691	RYR2/TGFB2/CPE	3
BP	GO:0055002	striated muscle cell development	3/340	71/18614	0.1403448	0.3484391	0.2645691	CAPN3/KRT19/MYH11	3

BP	GO:1902652	secondary alcohol metabolic process	5/340	150/18614	0.14052 44	0.34865 78	0.26473 51	IDH1/EPHX2/DHCR7/CFTR/CH25H	5
BP	GO:0032409	regulation of transporter activity	9/340	324/18614	0.14063 51	0.34870 55	0.26477 14	TRPC6/RYR2/FXYD1/KCNAB1/ANK2/GEM/CASQ2/CFTR/CCL2	9
BP	GO:0001676	long-chain fatty acid metabolic process	4/340	110/18614	0.14233 61	0.35246 46	0.26762 56	PTGS2/PTGDS/AKR1C3/ALOX15B	4
BP	GO:1901222	regulation of NIK/NF-kappaB	4/340	110/18614	0.14233 61	0.35246 46	0.26762 56	RTKN2/UACA/IL1B/CD27	4

		signaling							
BP	GO:0045834	positive regulation of lipid metabolic process	5/340	151/18614	0.1433159	0.3546602	0.2692928	ZBTB20/PTGS2/IL1B/FGR/NR4A3	5
BP	GO:0032715	negative regulation of interleukin-6 production	3/340	72/18614	0.1445897	0.3573486	0.2713334	HGF/ORM1/CX3CL1	3
BP	GO:0042130	negative regulation of T cell proliferation	3/340	72/18614	0.1445897	0.3573486	0.2713334	VSIG4/PLA2G2A/IDO1	3

BP	GO:0003230	cardiac atrium development	2/340	37/18614	0.146285 9	0.359442 7	0.272924 1	ANK2/TGFB2	2
BP	GO:0014037	Schwann cell differentiation	2/340	37/18614	0.146285 9	0.359442 7	0.272924 1	EGR2/NTRK2	2
BP	GO:0014059	regulation of dopamine secretion	2/340	37/18614	0.146285 9	0.359442 7	0.272924 1	CXCL12/CNR1	2
BP	GO:0035909	aorta morphogenesis	2/340	37/18614	0.146285 9	0.359442 7	0.272924 1	TGFB2/ADAMTS9	2
BP	GO:0038179	neurotrophin signaling pathway	2/340	37/18614	0.146285 9	0.359442 7	0.272924 1	DDIT4/NTRK2	2

BP	GO:0045920	negative regulation of exocytosis	2/340	37/18614	0.1462859	0.3594427	0.2729241	HMOX1/FCGR2B	2
BP	GO:0048854	brain morphogenesis	2/340	37/18614	0.1462859	0.3594427	0.2729241	SPEF2/SLC6A4	2
BP	GO:0060147	regulation of post-transcriptional gene silencing	2/340	37/18614	0.1462859	0.3594427	0.2729241	ZFP36/IL6	2
BP	GO:0060323	head morphogenesis	2/340	37/18614	0.1462859	0.3594427	0.2729241	ARID5B/CRISPLD2	2
BP	GO:0048638	regulation of developmenta	9/340	328/18614	0.14807785	0.3636129	0.2760905	OLFM1/CXCL12/SEMA3D/SEMA6D/TNR/CAPN3/SEMA6A/SEMA3G/SLC6A4	9

		l growth							
BP	GO:0007405	neuroblast proliferation	3/340	73/18614	0.14887766	0.36412224	0.27647774	BTG2/CX3CR1/CX3CL1	3
BP	GO:0061035	regulation of cartilage development	3/340	73/18614	0.14887766	0.36412224	0.27647774	FRZB/PKDCC/BMP1	3
BP	GO:0071260	cellular response to mechanical stimulus	3/340	73/18614	0.14887766	0.36412224	0.27647774	PTGS2/IL1B/IRF1	3
BP	GO:0034763	negative regulation of transmembrane	5/340	153/18614	0.14897703	0.36412224	0.27647774	KCNAB1/GEM/CASQ2/ACE/IL1B	5

		transport							
BP	GO:0046718	viral entry into host cell	5/340	153/18614	0.1489703	0.3641224	0.2764774	DPP4/SIGLEC1/ANPEP/CH25H/PTX3	5
BP	GO:0007229	integrin-mediated signaling pathway	4/340	112/18614	0.1490504	0.3641224	0.2764774	ITGA10/ITGA4/CD177/FGR	4
BP	GO:0035249	synaptic transmission, glutamatergic	4/340	112/18614	0.1490504	0.3641224	0.2764774	PTGS2/CNR1/TNR/CCL2	4
BP	GO:1905954	positive regulation of lipid	4/340	112/18614	0.1490504	0.3641224	0.2764774	PLIN2/ABCA8/IL1B/LIPG	4

		localization							
BP	GO:0016042	lipid catabolic process	9/340	330/18614	0.1518732	0.3700579	0.2809842	PLCB4/IDH1/PIK3CG/CNR1/RARRES2/IL1B/AKR1C3/LIPG/PLA2G2A	9
BP	GO:0006821	chloride transport	4/340	113/18614	0.1524526	0.3700579	0.2809842	FXYD1/ANO1/SLC1A3/CFTR	4
BP	GO:0002369	T cell cytokine production	2/340	38/18614	0.1526453	0.3700579	0.2809842	IL1B/IL6	2
BP	GO:0002724	regulation of T cell cytokine production	2/340	38/18614	0.1526453	0.3700579	0.2809842	IL1B/IL6	2
BP	GO:00	aortic valve	2/340	38/18614	0.152	0.370	0.280	ROBO2/SLIT3	2

	03180	morphogenesis			6453	0579	9842		
BP	GO:0010742	macrophage derived foam cell differentiation	2/340	38/18614	0.15264453	0.3700579	0.2809842	ALOX15B/PLA2G2A	2
BP	GO:0032373	positive regulation of sterol transport	2/340	38/18614	0.15264453	0.3700579	0.2809842	ABCA8/LIPG	2
BP	GO:0032376	positive regulation of cholesterol transport	2/340	38/18614	0.15264453	0.3700579	0.2809842	ABCA8/LIPG	2
BP	GO:0032728	positive regulation of interfer	2/340	38/18614	0.15264453	0.3700579	0.2809842	ZBTB20/IRF1	2

		on-beta production							
BP	GO:0033028	myeloid cell apoptotic process	2/340	38/18614	0.15264653	0.37005579	0.28098042	SLC7A11/IL6	2
BP	GO:0060306	regulation of membrane repolarization	2/340	38/18614	0.15264653	0.37005579	0.28098042	ANK2/CASQ2	2
BP	GO:0060966	regulation of gene silencing by RNA	2/340	38/18614	0.15264653	0.37005579	0.28098042	ZFP36/IL6	2
BP	GO:0030073	insulin secretion	6/340	197/18614	0.15282558	0.37026601	0.28113377	CNR1/CCL5/IL1B/ANO1/CFTR/IL6	6

BP	GO:0045661	regulation of myoblast differentiation	3/340	74/18614	0.1532041	0.3707053	0.2814757	CAPN3/CXCL10/CXCL9	3
BP	GO:0051145	smooth muscle cell differentiation	3/340	74/18614	0.1532041	0.3707053	0.2814757	NFATC2/APLNR/CTH	3
BP	GO:1904659	glucose transmembrane transport	4/340	114/18614	0.15588841	0.3769508	0.2862179	TRIB3/ACE/IL1B/NR4A3	4
BP	GO:0048662	negative regulation of smooth muscle cell proliferation	3/340	75/18614	0.1575771	0.3804439	0.28886665	HMOX1/TRIB1/OGN	3

BP	GO:0051899	membrane depolarization	3/340	75/18614	0.1575571	0.3804439	0.2888665	GCLM/ANK2/DCN	3
BP	GO:0008643	carbohydrate transport	5/340	156/18614	0.1576261	0.3804439	0.2888665	TRIB3/ACE/IL1B/NR4A3/AQP9	5
BP	GO:0045930	negative regulation of mitotic cell cycle	7/340	243/18614	0.1580408	0.3811983	0.2894431	BTG2/WEE1/PPP1R10/IER3/DTL/INHBA/CCL2	7
BP	GO:0007588	excretion	2/340	39/18614	0.1590576	0.3819588	0.2900205	CORIN/ACE	2
BP	GO:0045066	regulatory T cell differentiation	2/340	39/18614	0.1590576	0.3819588	0.2900205	IL2RG/IRF1	2

BP	GO:0045616	regulation of keratinocyte differentiation	2/340	39/18614	0.15905576	0.38195588	0.29002205	ZFP36/ALOX15B	2
BP	GO:0045730	respiratory burst	2/340	39/18614	0.15905576	0.38195588	0.29002205	PIK3CG/SLC11A1	2
BP	GO:0045823	positive regulation of heart contraction	2/340	39/18614	0.15905576	0.38195588	0.29002205	RYR2/TGFB2	2
BP	GO:0060251	regulation of glial cell proliferation	2/340	39/18614	0.15905576	0.38195588	0.29002205	IL1B/IL6	2
BP	GO:0090077	foam cell differentiation	2/340	39/18614	0.15905576	0.38195588	0.29002205	ALOX15B/PLA2G2A	2

BP	GO:0022037	metencephalon development	4/340	115/18614	0.15934344	0.38240 65	0.29036 04	CBS/CNTN1/FCGR2B/CD3E	4
BP	GO:0007422	peripheral nervous system development	3/340	76/18614	0.16197759	0.38682 05	0.29371 2	EGR2/EGR3/NTRK2	3
BP	GO:0042267	natural killer cell mediated cytotoxicity	3/340	76/18614	0.16197759	0.38682 05	0.29371 2	SLAMF7/RASGRP1/CD2	3
BP	GO:0050772	positive regulation of axonogenesis	3/340	76/18614	0.16197759	0.38682 05	0.29371 2	CXCL12/ROBO2/NTRK2	3

BP	GO:1903036	positive regulation of response to wounding	3/340	76/18614	0.16197759	0.3868205	0.29371 2	DUOX1/SERPINF2/DDR2	3
BP	GO:0009185	ribonucleoside diphosphate metabolic process	4/340	116/18614	0.16283328	0.3868205	0.29371 2	ZBTB20/ENO1/IER3/DDIT4	4
BP	GO:0001916	positive regulation of T cell mediated cytotoxicity	2/340	40/18614	0.16551 9	0.3868205	0.29371 2	CD1C/CD1E	2
BP	GO:00	regulation of histone	2/340	40/18614	0.165	0.386	0.293	SDR16C5/APLNR	2

	31063	deacetylation			519	8205	712		
BP	GO:0042092	type 2 immune response	2/340	40/18614	0.165519	0.3868205	0.293712	IL6/IDO1	2
BP	GO:0042755	eating behavior	2/340	40/18614	0.165519	0.3868205	0.293712	P2RY1/ACE	2
BP	GO:0043267	negative regulation of potassium ion transport	2/340	40/18614	0.165519	0.3868205	0.293712	KCNAB1/CASQ2	2
BP	GO:0045454	cell redox homeostasis	2/340	40/18614	0.165519	0.3868205	0.293712	NQO1/SLC11A1	2
BP	GO:00	negative regulati	2/340	40/18614	0.165	0.386	0.293	TRIB3/CNR1	2

	45922	on of fatty acid metabolic process			519	8205	712		
BP	GO:0046627	negative regulation of insulin receptor signaling pathway	2/340	40/18614	0.165519	0.386825	0.293712	IL1B/SOCS3	2
BP	GO:0050850	positive regulation of calcium-mediated	2/340	40/18614	0.165519	0.386825	0.293712	TRAT1/CD3E	2

		signaling							
BP	GO:0090181	regulation of cholesterol metabolic process	2/340	40/18614	0.1655519	0.3868205	0.2937712	EPHX2/DHCR7	2
BP	GO:1903115	regulation of actin filament-based movement	2/340	40/18614	0.1655519	0.3868205	0.2937712	RYR2/ANK2	2
BP	GO:1903524	positive regulation of blood circulation	2/340	40/18614	0.1655519	0.3868205	0.2937712	RYR2/TGFB2	2
BP	GO:00	neurotransmitter	6/340	202/18614	0.165	0.386	0.293	P2RY1/SLC18A2/CSPG5/SLC1A3/NR4A1/SLC6A4	6

	06836	transport			6754	8205	712		
BP	GO:0008645	hexose transmembrane transport	4/340	117/18614	0.166343488	0.386828205	0.293712	TRIB3/ACE/IL1B/NR4A3	4
BP	GO:0030203	glycosaminoglycan metabolic process	4/340	117/18614	0.166343488	0.386828205	0.293712	IL1B/LYVE1/DCN/HAS2	4
BP	GO:0003407	neural retina development	3/340	77/18614	0.166414177	0.386828205	0.293712	TGFB2/FAT3/NTRK2	3
BP	GO:0000098	sulfur amino acid catabolic process	1/340	10/18614	0.168683903	0.386828205	0.293712	CBS	1

BP	GO:0001915	negative regulation of T cell mediated cytotoxicity	1/340	10/18614	0.1683903	0.3868205	0.2937712	FCGR2B	1
BP	GO:0002138	retinoic acid biosynthetic process	1/340	10/18614	0.1683903	0.3868205	0.2937712	AKR1C3	1
BP	GO:0002361	CD4-positive, CD25-positive, alpha-beta regulatory T cell differentiation	1/340	10/18614	0.1683903	0.3868205	0.2937712	IL2RG	1

BP	GO:0003129	heart induction	1/340	10/18614	0.1683903	0.38682205	0.29371 2	ROBO2	1
BP	GO:0006547	histidine metabolic process	1/340	10/18614	0.1683903	0.38682205	0.29371 2	HDC	1
BP	GO:0006560	proline metabolic process	1/340	10/18614	0.1683903	0.38682205	0.29371 2	PYCR1	1
BP	GO:0007028	cytoplasm organization	1/340	10/18614	0.1683903	0.38682205	0.29371 2	TFCP2L1	1
BP	GO:0008298	intracellular mRNA localization	1/340	10/18614	0.1683903	0.38682205	0.29371 2	ZFP36	1

BP	GO:0008300	isoprenoid catabolic process	1/340	10/18614	0.1683903	0.3868205	0.2937712	AKR1C3	1
BP	GO:0008627	intrinsic apoptotic signaling pathway in response to osmotic stress	1/340	10/18614	0.1683903	0.3868205	0.2937712	PTGS2	1
BP	GO:0009113	purine nucleobase biosynthetic process	1/340	10/18614	0.1683903	0.3868205	0.2937712	SHMT2	1

BP	GO:0009629	response to gravity	1/340	10/18614	0.168390 3	0.386820 5	0.293712	FOS	1
BP	GO:0014012	peripheral nervous system axon regeneration	1/340	10/18614	0.168390 3	0.386820 5	0.293712	TNC	1
BP	GO:0014866	skeletal myofibril assembly	1/340	10/18614	0.168390 3	0.386820 5	0.293712	MYH11	1
BP	GO:0015851	nucleobase transport	1/340	10/18614	0.168390 3	0.386820 5	0.293712	AQP9	1
BP	GO:0019062	virion attachment to host cell	1/340	10/18614	0.168390 3	0.386820 5	0.293712	DPP4	1

BP	GO:0021561	facial nerve development	1/340	10/18614	0.1683903	0.3868205	0.2937712	EGR2	1
BP	GO:0021604	cranial nerve structural organization	1/340	10/18614	0.1683903	0.3868205	0.2937712	EGR2	1
BP	GO:0021610	facial nerve morphogenesis	1/340	10/18614	0.1683903	0.3868205	0.2937712	EGR2	1
BP	GO:0032353	negative regulation of hormone biosynthetic process	1/340	10/18614	0.1683903	0.3868205	0.2937712	BMP5	1

BP	GO:0032815	negative regulation of natural killer cell activation	1/340	10/18614	0.1683903	0.38682 05	0.293712	FGR	1
BP	GO:0033007	negative regulation of mast cell activation involved in immune response	1/340	10/18614	0.1683903	0.3868205	0.293712	HMOX1	1
BP	GO:00	iron import into cell	1/340	10/18614	0.168	0.386	0.293	SLC39A14	1

	33212				3903	8205	712		
BP	GO:0034651	cortisol biosynthetic process	1/340	10/18614	0.1683903	0.3868205	0.293712	BMP5	1
BP	GO:0035860	glial cell-derived neurotrophic factor receptor signaling pathway	1/340	10/18614	0.1683903	0.3868205	0.293712	SULF1	1
BP	GO:0042135	neurotransmitter catabolic process	1/340	10/18614	0.1683903	0.3868205	0.293712	MAOA	1

BP	GO:0046886	positive regulation of hormone biosynthetic process	1/340	10/18614	0.1683903	0.3868205	0.2937712	EGR1	1
BP	GO:0050861	positive regulation of B cell receptor signaling pathway	1/340	10/18614	0.1683903	0.3868205	0.2937712	STAP1	1
BP	GO:0051342	regulation of cyclic-nucleotide phosphodieste	1/340	10/18614	0.1683903	0.3868205	0.2937712	SLC39A14	1

		rase activity							
BP	GO:0051351	positive regulation of ligase activity	1/340	10/18614	0.1683903	0.3868205	0.2937172	GCLM	1
BP	GO:0051409	response to nitrosative stress	1/340	10/18614	0.1683903	0.3868205	0.2937172	GCLM	1
BP	GO:0060245	detection of cell density	1/340	10/18614	0.1683903	0.3868205	0.2937172	SRPX	1
BP	GO:0061626	pharyngeal arch artery morphogenesis	1/340	10/18614	0.1683903	0.3868205	0.2937172	TGFB2	1
BP	GO:0062042	regulation of cardiac epitheli	1/340	10/18614	0.1683903	0.3868205	0.2937172	TGFB2	1

		al to mesenchymal transition							
BP	GO:0071374	cellular response to parathyroid hormone stimulus	1/340	10/18614	0.1683903	0.3868205	0.29371 2	FOS	1
BP	GO:0071838	cell proliferation in bone marrow	1/340	10/18614	0.1683903	0.3868205	0.29371 2	ACE	1
BP	GO:0072203	cell proliferation involved in metanephros	1/340	10/18614	0.1683903	0.3868205	0.29371 2	EGR1	1

BP	GO:0090281	development negative regulation of calcium ion import	1/340	10/18614	0.1683903	0.38682 05	0.2937712	ACE	1
BP	GO:0097113	AMPA glutamate receptor clustering	1/340	10/18614	0.1683903	0.38682 05	0.2937712	SLC7A11	1
BP	GO:0097201	negative regulation of transcription from RNA polymerase II	1/340	10/18614	0.1683903	0.38682 05	0.2937712	JUN	1

		promoter in response to stress							
BP	GO:0097688	glutamate receptor clustering	1/340	10/18614	0.1683903	0.3868205	0.2937712	SLC7A11	1
BP	GO:0098917	retrograde trans-synaptic signaling	1/340	10/18614	0.1683903	0.3868205	0.2937712	CNR1	1
BP	GO:1902224	ketone body metabolic process	1/340	10/18614	0.1683903	0.3868205	0.2937712	HMGCLL1	1

BP	GO:1902645	tertiary alcohol biosynthetic process	1/340	10/18614	0.16839 03	0.38682 05	0.29371 2	BMP5	1
BP	GO:1903959	regulation of monoatomic anion transmembrane transport	1/340	10/18614	0.16839 03	0.38682 05	0.29371 2	CFTR	1
BP	GO:1903980	positive regulation of microglial cell activation	1/340	10/18614	0.16839 03	0.38682 05	0.29371 2	STAP1	1
BP	GO:19	positive regulation of	1/340	10/18614	0.168	0.386	0.293	DCN	1

	04181	membrane depolarization			3903	8205	712		
BP	GO:2000425	regulation of apoptotic cell clearance	1/340	10/18614	0.16839903	0.38682805	0.2937712	CCL2	1
BP	GO:2000563	positive regulation of CD4-positive, alpha-beta T cell proliferation	1/340	10/18614	0.16839903	0.38682805	0.2937712	CD3E	1
BP	GO:0044409	entry into host	5/340	160/18614	0.16947487	0.38908862	0.29543324	DPP4/SIGLEC1/ANPEP/CH25H/PTX3	5

BP	GO:0002534	cytokine production involved in inflammatory response	3/340	78/18614	0.170895	0.391963	0.2973639	IL1R2/IL6/GBP5	3
BP	GO:0061045	negative regulation of wound healing	3/340	78/18614	0.170895	0.391963	0.2973639	MMRN1/SERPINF2/FGA	3
BP	GO:1900015	regulation of cytokine production involved in inflammatory	3/340	78/18614	0.170895	0.391963	0.2973639	IL1R2/IL6/GBP5	3

BP	GO:0003351	response epithelial cilium movement involved in extracellular fluid movement	2/340	41/18614	0.17202 55	0.39374 72	0.29897 14	SPEF2/DNAH5	2
BP	GO:0072210	metanephric nephron development	2/340	41/18614	0.17202 55	0.39374 72	0.29897 14	EGR1/FMN1	2
BP	GO:0001933	negative regulation of protein phosph	9/340	341/18614	0.17358 4	0.39687 83	0.30134 89	TRIB3/HGF/JUN/LAX1/IL1B/SFN/TRIB1/DDIT4/SOCS3	9

		orylation							
BP	GO:0010632	regulation of epithelial cell migration	8/340	295/18614	0.17360 17	0.39687 83	0.30134 89	PTGS2/TGFB2/PIK3CG/JUN/HMOX1/DCN/ADAMTS9/HAS2	8
BP	GO:0016054	organic acid catabolic process	7/340	250/18614	0.17452 42	0.39850 91	0.30258 71	FAH/SHMT2/CBS/HMGCLL1/HDC/CNR1/IDO1	7
BP	GO:0046395	carboxylic acid catabolic process	7/340	250/18614	0.17452 42	0.39850 91	0.30258 71	FAH/SHMT2/CBS/HMGCLL1/HDC/CNR1/IDO1	7
BP	GO:0019362	pyridine nucleotide metabo	3/340	79/18614	0.17540 66	0.39889 17	0.30287 76	IDH1/NQO1/IDO1	3

		lic process							
BP	GO:0045913	positive regulation of carbohydrate metabolic process	3/340	79/18614	0.1754066	0.3988917	0.3028776	P2RY1/ZBTB20/HAS2	3
BP	GO:0046496	nicotinamide nucleotide metabolic process	3/340	79/18614	0.1754066	0.3988917	0.3028776	IDH1/NQO1/IDO1	3
BP	GO:1902475	L-alpha-amino acid transmembran	3/340	79/18614	0.1754066	0.3988917	0.3028776	SLC7A11/SLC1A3/SLC11A1	3

		e transport							
BP	GO:0000082	G1/S transition of mitotic cell cycle	7/340	251/18614	0.1769378	0.3988917	0.3028776	EIF4EBP1/PIM2/WEE1/DDR2/BCAT1/INHBA/CCL2	7
BP	GO:0031330	negative regulation of cellular catabolic process	7/340	251/18614	0.1769378	0.3988917	0.3028776	HGF/PIK3CG/TIMP4/CNR1/HMOX1/ZFP36/SLC11A1	7
BP	GO:0015749	monosaccharide transmembrane transport	4/340	120/18614	0.1770563	0.3988917	0.3028776	TRIB3/ACE/IL1B/NR4A3	4

BP	GO:0014912	negative regulation of smooth muscle cell migration	2/340	42/18614	0.1785732	0.3988917	0.3028776	SEMA6D/TRIB1	2
BP	GO:0045124	regulation of bone resorption	2/340	42/18614	0.1785732	0.3988917	0.3028776	TNFRSF11B/IL6	2
BP	GO:0045622	regulation of T-helper cell differentiation	2/340	42/18614	0.1785732	0.3988917	0.30287776	NFKBIZ/JUNB	2
BP	GO:0045981	positive regulation of nucleotide metabo	2/340	42/18614	0.1785732	0.3988917	0.30287776	ZBTB20/ENO1	2

		lic process							
BP	GO:0046688	response to copper ion	2/340	42/18614	0.17857 32	0.39889 17	0.30287 76	MT1X/MT1M	2
BP	GO:0050999	regulation of nitric-oxide synthase activity	2/340	42/18614	0.17857 32	0.39889 17	0.30287 76	CNR1/IL1B	2
BP	GO:1900077	negative regulation of cellular response to insulin stimulus	2/340	42/18614	0.17857 32	0.39889 17	0.30287 76	IL1B/SOCS3	2

BP	GO:1900544	positive regulation of purine nucleotide metabolic process	2/340	42/18614	0.17857 32	0.39889 17	0.30287 76	ZBTB20/ENO1	2
BP	GO:1901020	negative regulation of calcium ion transmembrane transporter activity	2/340	42/18614	0.17857 32	0.39889 17	0.30287 76	GEM/CASQ2	2
BP	GO:0001895	retina homeostasis	3/340	80/18614	0.17995 12	0.39889 17	0.30287 76	PCDH15/LYZ/AZGP1	3

BP	GO:0002720	positive regulation of cytokine production involved in immune response	3/340	80/18614	0.1799512	0.3988917	0.30287 76	IL1B/NR4A3/IL6	3
BP	GO:0007193	adenylate cyclase-inhibiting G protein-coupled receptor signaling	3/340	80/18614	0.1799512	0.3988917	0.30287 76	P2RY1/MARCO/FPR2	3

BP	GO:0042440	pathway pigment metabolic process	3/340	80/18614	0.17995512	0.39889817	0.30288776	TRPC1/HMOX1/SLC7A11	3
BP	GO:0048644	muscle organ morphogenesis	3/340	80/18614	0.17995512	0.39889817	0.30288776	RYR2/ARID5B/TGFB2	3
BP	GO:0043500	muscle adaptation	4/340	121/18614	0.18067766	0.39889817	0.30288776	GTF2IRD1/HMOX1/IL1B/NR4A3	4
BP	GO:0098773	skin epidermis development	4/340	121/18614	0.18067766	0.39889817	0.30288776	TGFB2/NFKBIZ/SFN/INHBA	4
BP	GO:0001547	antral ovarian follicle growth	1/340	11/18614	0.18358885	0.39889817	0.30288776	PTX3	1

BP	GO:0001821	histamine secretion	1/340	11/18614	0.18358885	0.3988917	0.30287776	SLC18A2	1
BP	GO:0002158	osteoclast proliferation	1/340	11/18614	0.18358885	0.3988917	0.30287776	JUNB	1
BP	GO:0002775	antimicrobial peptide production	1/340	11/18614	0.18358885	0.3988917	0.30287776	PGC	1
BP	GO:0002924	negative regulation of humoral immune response mediated by circulati	1/340	11/18614	0.18358885	0.3988917	0.30287776	FCGR2B	1

		ng immunoglobulin							
BP	GO:0006188	IMP biosynthetic process	1/340	11/18614	0.18358585	0.39889817	0.30287776	AMPD1	1
BP	GO:0006527	arginine catabolic process	1/340	11/18614	0.18358585	0.39889817	0.30287776	FAH	1
BP	GO:0006568	tryptophan metabolic process	1/340	11/18614	0.18358585	0.39889817	0.30287776	IDO1	1
BP	GO:0006686	sphingomyelin biosynthetic	1/340	11/18614	0.18358585	0.39889817	0.30287776	ABCA8	1

		process							
BP	GO:0007406	negative regulation of neuroblast proliferation	1/340	11/18614	0.18358 85	0.39889 17	0.30287 76	BTG2	1
BP	GO:0008611	ether lipid biosynthetic process	1/340	11/18614	0.18358 85	0.39889 17	0.30287 76	FASN	1
BP	GO:0010982	regulation of high-density lipoprotein particle clearance	1/340	11/18614	0.18358 85	0.39889 17	0.30287 76	LIPG	1

BP	GO:0010986	positive regulation of lipoprotein particle clearance	1/340	11/18614	0.18358585	0.39889017	0.30287776	LIPG	1
BP	GO:0016102	diterpenoid biosynthetic process	1/340	11/18614	0.18358585	0.39889017	0.30287776	AKR1C3	1
BP	GO:0021889	olfactory bulb interneuron differentiation	1/340	11/18614	0.18358585	0.39889017	0.30287776	ROBO2	1
BP	GO:0021936	regulation of cerebellar granule cell	1/340	11/18614	0.18358585	0.39889017	0.30287776	SLC6A4	1

BP	GO:0022605	precursor proliferation mammalian oogenesis stage	1/340	11/18614	0.18358 85	0.39889 17	0.30287 76	PTX3		1
BP	GO:0031943	regulation of glucocorticoid metabolic process	1/340	11/18614	0.18358 85	0.39889 17	0.30287 76	BMP5		1
BP	GO:0032261	purine nucleotide salvage	1/340	11/18614	0.18358 85	0.39889 17	0.30287 76	AMPD1		1
BP	GO:0032276	regulation of gonadotropin	1/340	11/18614	0.18358 85	0.39889 17	0.30287 76	INHBA		1

		secretion							
BP	GO:0032342	aldosterone biosynthetic process	1/340	11/18614	0.18358885	0.39889817	0.30287776	BMP5	1
BP	GO:0032836	glomerular basement membrane development	1/340	11/18614	0.18358885	0.39889817	0.30287776	SULF1	1
BP	GO:0033234	negative regulation of protein sumoylation	1/340	11/18614	0.18358885	0.39889817	0.30287776	CAPN3	1
BP	GO:00	positive regulation of	1/340	11/18614	0.183	0.398	0.302	SKAP1	1

	33625	integrin activation			5885	8917	8776		
BP	GO:0034350	regulation of glial cell apoptotic process	1/340	11/18614	0.18358885	0.39889917	0.30287776	CCL2	1
BP	GO:0038063	collagen-activated tyrosine kinase receptor signaling pathway	1/340	11/18614	0.18358885	0.39889917	0.30287776	DDR2	1

BP	GO:0042045	epithelial fluid transport	1/340	11/18614	0.18358885	0.3988917	0.30287876	CFTR	1
BP	GO:0042118	endothelial cell activation	1/340	11/18614	0.18358885	0.3988917	0.30287876	CXCL10	1
BP	GO:0042167	heme catabolic process	1/340	11/18614	0.18358885	0.3988917	0.30287876	HMOX1	1
BP	GO:0042487	regulation of odontogenesis of dentin-containing tooth	1/340	11/18614	0.18358885	0.3988917	0.30287876	TNFRSF11B	1
BP	GO:00	negative regulati	1/340	11/18614	0.183	0.398	0.302	APLNR	1

	43951	on of cAMP-mediated signaling			5885	8917	8776		
BP	GO:0044848	biological phase	1/340	11/18614	0.1835885	0.3988917	0.3028776	TGFB2	1
BP	GO:0045060	negative thymic T cell selection	1/340	11/18614	0.1835885	0.3988917	0.3028776	CD3E	1
BP	GO:0046149	pigment catabolic process	1/340	11/18614	0.1835885	0.3988917	0.3028776	HMOX1	1
BP	GO:0046504	glycerol ether biosynthetic	1/340	11/18614	0.1835885	0.3988917	0.3028776	FASN	1

		process							
BP	GO:0046643	regulation of gamma-delta T cell activation	1/340	11/18614	0.18358 85	0.39889 17	0.30287 76	EGR3	1
BP	GO:0060081	membrane hyperpolarization	1/340	11/18614	0.18358 85	0.39889 17	0.30287 76	CFTR	1
BP	GO:0060315	negative regulation of ryanodine-sensitive calcium-release channe	1/340	11/18614	0.18358 85	0.39889 17	0.30287 76	CASQ2	1

		I activity							
BP	GO:0060601	lateral sprouting from an epithelium	1/340	11/18614	0.1835885	0.3988917	0.3028776	SULF1	1
BP	GO:0061418	regulation of transcription from RNA polymerase II promoter in response to hypoxia	1/340	11/18614	0.1835885	0.3988917	0.3028776	EGR1	1
BP	GO:0070189	kynurenine metabolic	1/340	11/18614	0.1835885	0.3988917	0.3028776	IDO1	1

		process							
BP	GO:0070327	thyroid hormone transport	1/340	11/18614	0.18358585	0.3988917	0.30287776	SLCO4A1	1
BP	GO:0071872	cellular response to epinephrine stimulus	1/340	11/18614	0.18358585	0.3988917	0.30287776	RYR2	1
BP	GO:0072124	regulation of glomerular mesangial cell proliferation	1/340	11/18614	0.18358585	0.3988917	0.30287776	EGR1	1
BP	GO:0086016	AV node cell action	1/340	11/18614	0.18358585	0.3988917	0.30287776	RYR2	1

		potential							
BP	GO:0086027	AV node cell to bundle of His cell signaling	1/340	11/18614	0.1835885	0.3988917	0.3028776	RYR2	1
BP	GO:0090527	actin filament reorganization	1/340	11/18614	0.1835885	0.3988917	0.3028776	HMCN1	1
BP	GO:0090557	establishment of endothelial intestinal barrier	1/340	11/18614	0.1835885	0.3988917	0.3028776	FASN	1
BP	GO:00	mitochondrial respira	1/340	11/18614	0.183	0.398	0.302	HIGD1B	1

	97250	some assembly			5885	8917	8776		
BP	GO:0097384	cellular lipid biosynthetic process	1/340	11/18614	0.1835885	0.3988917	0.3028776	FASN	1
BP	GO:1902510	regulation of apoptotic DNA fragmentation	1/340	11/18614	0.1835885	0.3988917	0.3028776	IL6	1
BP	GO:1904238	pericyte cell differentiation	1/340	11/18614	0.1835885	0.3988917	0.3028776	GPR4	1
BP	GO:1905665	positive regulation of calcium ion import across	1/340	11/18614	0.1835885	0.3988917	0.3028776	MS4A1	1

		plasma membrane							
BP	GO:1990440	positive regulation of transcription from RNA polymerase II promoter in response to endoplasmic reticulum stress	1/340	11/18614	0.1835885	0.3988917	0.3028776	ATF3	1
BP	GO:2000049	positive regulation of cell-cell adhesi	1/340	11/18614	0.1835885	0.3988917	0.3028776	SERPINF2	1

		on mediated by cadherin							
BP	GO:2000169	regulation of peptidyl-cysteine S-nitrosylation	1/340	11/18614	0.1835885	0.3988917	0.3028776	ACE	1
BP	GO:2000826	regulation of heart morphogenesis	1/340	11/18614	0.1835885	0.3988917	0.3028776	ROBO2	1
BP	GO:2001223	negative regulation of neuron migration	1/340	11/18614	0.1835885	0.3988917	0.3028776	CX3CL1	1

BP	GO:0090100	positive regulation of transmembrane receptor protein serine/threonine kinase signaling pathway	4/340	122/18614	0.18432 16	0.39889 17	0.30287 76	BMP5/TGFB2/SULF1/INHBA	4
BP	GO:0098661	inorganic anion transmembrane transport	4/340	122/18614	0.18432 16	0.39889 17	0.30287 76	FXYD1/ANO1/SLC1A3/CFTR	4

BP	GO:0071383	cellular response to steroid hormone stimulus	6/340	209/18614	0.18440406	0.3988917	0.3028776	SCNN1G/ACE/ZFP36/NR4A3/AKR1C3/DDIT4	6
BP	GO:0014855	striated muscle cell proliferation	3/340	81/18614	0.18452775	0.3988917	0.3028776	HGF/TGFB2/FOS	3
BP	GO:0044773	mitotic DNA damage checkpoint signaling	3/340	81/18614	0.18452775	0.3988917	0.3028776	PPP1R10/IER3/DTL	3
BP	GO:0002251	organ or tissue specific immun	2/340	43/18614	0.18515586	0.3988917	0.30287776	RNASE2/IL6	2

		e<br>respon<br>se							
B<br>P	GO<br>:00<br>031<br>76	aortic<br>valve<br>develo<br>pment	2/<br>34<br>0	43/<br>186<br>14	0.1<br>85<br>15<br>86	0.3<br>98<br>89<br>17	0.3<br>02<br>87<br>76	ROBO2/SLIT3	2
B<br>P	GO<br>:00<br>067<br>39	NADP<br>metabo<br>lic<br>proces<br>s	2/<br>34<br>0	43/<br>186<br>14	0.1<br>85<br>15<br>86	0.3<br>98<br>89<br>17	0.3<br>02<br>87<br>76	IDH1/NQO1	2
B<br>P	GO<br>:00<br>069<br>04	vesicle<br>docking<br>involve<br>d in<br>exocyto<br>sis	2/<br>34<br>0	43/<br>186<br>14	0.1<br>85<br>15<br>86	0.3<br>98<br>89<br>17	0.3<br>02<br>87<br>76	SYTL2/CFTR	2
B<br>P	GO<br>:00<br>107<br>19	negativ<br>e<br>regulati<br>on of<br>epitheli<br>al to<br>mesen<br>chymal	2/<br>34<br>0	43/<br>186<br>14	0.1<br>85<br>15<br>86	0.3<br>98<br>89<br>17	0.3<br>02<br>87<br>76	BMP5/TGFB2	2

BP	GO:0031670	cellular response to nutrient	2/340	43/18614	0.18515586	0.39889917	0.30287776	HMOX1/POSTN	2
BP	GO:0031952	regulation of protein autophosphorylation	2/340	43/18614	0.18515586	0.39889917	0.30287776	JUN/ACE	2
BP	GO:0032689	negative regulation of type II interferon production	2/340	43/18614	0.18515586	0.39889917	0.30287776	CD96/INHBA	2
BP	GO:0032735	positive regulation of interleukin-12	2/340	43/18614	0.18515586	0.39889917	0.30287776	IRF1/IDO1	2

		production							
BP	GO:0032814	regulation of natural killer cell activation	2/340	43/18614	0.18515 86	0.39889 17	0.30287 76	FGR/RASGRP1	2
BP	GO:0034142	toll-like receptor 4 signaling pathway	2/340	43/18614	0.18515 86	0.39889 17	0.30287 76	TRIL/TNIP3	2
BP	GO:0051150	regulation of smooth muscle cell differentiation	2/340	43/18614	0.18515 86	0.39889 17	0.30287 76	NFATC2/CTH	2
BP	GO:00	cellular response to	2/340	43/18614	0.185	0.398	0.302	ZFP36/FOS	2

	71364	epidermal growth factor stimulus			1586	8917	8776		
BP	GO:0071548	response to dexamethasone	2/340	43/18614	0.185151586	0.398898917	0.302878776	ACE/DDIT4	2
BP	GO:0030856	regulation of epithelial cell differentiation	5/340	166/18614	0.187888816	0.404078888	0.306068162	FRZB/ZFP36/IL1B/SFN/ALOX15B	5
BP	GO:0001578	microtubule bundle formation	4/340	123/18614	0.187879905	0.404078888	0.306068162	NCKAP5/SPEF2/TPPP3/DNAH5	4
BP	GO:0002761	regulation of myeloid leukocy	4/340	123/18614	0.187879905	0.404078888	0.306068162	TNFRSF11B/TRIB1/FOS/INHBA	4

		te differentiation							
BP	GO:0006805	xenobiotic metabolic process	4/340	123/18614	0.1879905	0.4040788	0.3068162	NQO1/SULT1B1/AOX1/S100A12	4
BP	GO:0003151	outflow tract morphogenesis	3/340	82/18614	0.1891345	0.4060797	0.3083355	ROBO2/TGFB2/JUN	3
BP	GO:0042509	regulation of tyrosine phosphorylation of STAT protein	3/340	82/18614	0.1891345	0.4060797	0.3083355	CCL5/IL6/SOCS3	3
BP	GO:0007613	memory	4/340	124/18614	0.1916828	0.40893	0.3104997	PTGS2/CNR1/CX3CR1/SLC6A4	4

BP	GO:0043280	positive regulation of cysteine-type endopeptidase activity involved in apoptotic process	4/340	124/18614	0.19168 28	0.408 93	0.3104997	NLRC4/UACA/S100A8/TNFSF10	4
BP	GO:0006509	membrane protein ectodomain proteolysis	2/340	44/18614	0.1917781	0.40893	0.3104997	TIMP4/IL1B	2
BP	GO:0006858	extracellular transport	2/340	44/18614	0.1917781	0.40893	0.3104997	SPEF2/DNAH5	2

BP	GO:0032691	negative regulation of interleukin-1 beta production	2/340	44/18614	0.19177781	0.40893	0.3104997	CX3CR1/CX3CL1	2
BP	GO:0045214	sarcomere organization	2/340	44/18614	0.19177781	0.40893	0.3104997	CAPN3/KRT19	2
BP	GO:0046640	regulation of alpha-beta T cell proliferation	2/340	44/18614	0.19177781	0.40893	0.3104997	IRF1/CD3E	2
BP	GO:0120178	steroid hormone biosynthetic	2/340	44/18614	0.19177781	0.40893	0.31049997	BMP5/EGR1	2

		process							
BP	GO:2000249	regulation of actin cytoskeleton reorganization	2/340	44/18614	0.19177781	0.40893	0.31049 97	STAP1/CSF3	2
BP	GO:0030900	forebrain development	10/340	397/18614	0.19206613	0.40893	0.31049 97	BTG2/CXCL12/ROBO2/TNR/TACC2/SLC7A11/INHBA/NTRK2/DNAH5/NR4A2	10
BP	GO:0007492	endoderm development	3/340	83/18614	0.19377707	0.40893	0.31049 97	ITGA4/COL8A1/INHBA	3
BP	GO:0140029	exocytic process	3/340	83/18614	0.19377707	0.40893	0.31049 97	SLC18A2/SYTL2/CFTR	3
BP	GO:0048872	homeostasis of number of cells	8/340	304/18614	0.19387772	0.40893	0.31049 97	NFKBIZ/HMOX1/SLC7A11/ZFP36/INHBA/IL6/CX3CR1/PLA2G2A	8

BP	GO:0007178	transmembrane receptor protein serine/threonine kinase signaling pathway	10/340	398/18614	0.19406 21	0.408 93	0.3104997	BMP5/LTBP4/LTBP2/TGFB2/EGR1/JUN/SULF1/FOS/INHBA/ASPN	10
BP	GO:0001952	regulation of cell-matrix adhesion	4/340	125/18614	0.1953979	0.40893	0.3104997	SKAP1/FMN1/CD3E/CX3CL1	4
BP	GO:0098656	monoatomic anion transmembrane	4/340	125/18614	0.1953979	0.40893	0.3104997	FXYD1/ANO1/SLC1A3/CFTR	4

		transport							
BP	GO:0045216	cell-cell junction organization	6/340	214/18614	0.1982264	0.4089 3	0.3104997	ANK2/TGFB2/APLNR/CD177/ACE/IL1B	6
BP	GO:0009268	response to pH	2/340	45/18614	0.1984282	0.408093	0.3104997	SCNN1G/GPR4	2
BP	GO:0010677	negative regulation of cellular carbohydrate metabolic process	2/340	45/18614	0.1984282	0.408093	0.3104997	IER3/DDIT4	2
BP	GO:0048483	autonomic nervous system	2/340	45/18614	0.1984282	0.408093	0.3104997	EGR2/VCAM1	2

		development							
BP	GO:1900744	regulation of p38MAPK cascade	2/340	45/18614	0.198428 2	0.408893	0.310499 7	HGF/IL1B	2
BP	GO:1901021	positive regulation of calcium ion transmembrane transporter activity	2/340	45/18614	0.198428 2	0.408893	0.310499 7	RYR2/ANK2	2
BP	GO:1903307	positive regulation of regulated secretory	2/340	45/18614	0.198428 2	0.408893	0.310499 7	CD177/FGR	2

		pathway							
BP	GO:0001553	luteinization	1/340	12/18614	0.1985097	0.40893	0.3104997	INHBA	1
BP	GO:0001973	G protein-coupled adenosine receptor signaling pathway	1/340	12/18614	0.1985097	0.40893	0.3104997	P2RY1	1
BP	GO:0002674	negative regulation of acute inflammatory	1/340	12/18614	0.1985097	0.40893	0.3104997	FCGR2B	1

		response							
BP	GO:0002759	regulation of antimicrobial humoral response	1/340	12/18614	0.19850997	0.40893	0.3104997	PGC	1
BP	GO:0005984	disaccharide metabolic process	1/340	12/18614	0.19850997	0.40893	0.3104997	MGAM	1
BP	GO:0006068	ethanol catabolic process	1/340	12/18614	0.19850997	0.40893	0.31049997	SULT1B1	1
BP	GO:0006705	mineralocorticoid biosynthetic	1/340	12/18614	0.19850997	0.40893	0.31049997	BMP5	1

		process							
BP	GO:0009650	UV protection	1/340	12/18614	0.1985097	0.40893	0.3104997	MFAP4	1
BP	GO:0010749	regulation of nitric oxide mediated signal transduction	1/340	12/18614	0.1985097	0.40893	0.3104997	CBS	1
BP	GO:0010936	negative regulation of macrophage cytokine production	1/340	12/18614	0.1985097	0.40893	0.3104997	TGFB2	1

BP	GO:0014831	gastro-intestinal system smooth muscle contraction	1/340	12/18614	0.198509 7	0.408893	0.310499 7	SULF1	1
BP	GO:0015791	polyol transmembrane transport	1/340	12/18614	0.198509 7	0.408893	0.310499 7	AQP9	1
BP	GO:0021670	lateral ventricle development	1/340	12/18614	0.198509 7	0.408893	0.310499 7	DNAH5	1
BP	GO:0030656	regulation of vitamin metabolic process	1/340	12/18614	0.198509 7	0.408893	0.310499 7	AKR1C3	1

BP	GO:0031223	auditory behavior	1/340	12/18614	0.19850 97	0.40893	0.31049 97	SLC1A3	1
BP	GO:0031953	negative regulation of protein autophosphorylation	1/340	12/18614	0.19850 97	0.40893	0.31049 97	JUN	1
BP	GO:0032957	inositol trisphosphate metabolic process	1/340	12/18614	0.19850 97	0.40893	0.31049 97	P2RY1	1
BP	GO:0033083	regulation of immature T cell proliferation	1/340	12/18614	0.19850 97	0.40893	0.31049 97	IL1B	1

BP	GO:0033084	regulation of immature T cell proliferation in thymus	1/340	12/18614	0.19850697	0.4089 3	0.31049 97	IL1B	1
BP	GO:0034650	cortisol metabolic process	1/340	12/18614	0.19850697	0.40893	0.31049 97	BMP5	1
BP	GO:0035588	G protein-coupled purinergic receptor signaling pathway	1/340	12/18614	0.19850697	0.40893	0.31049 97	P2RY1	1

BP	GO:0035810	positive regulation of urine volume	1/340	12/18614	0.1985097	0.408093	0.31049997	HAS2	1
BP	GO:0042447	hormone catabolic process	1/340	12/18614	0.1985097	0.408093	0.31049997	ACE	1
BP	GO:0043312	neutrophil degranulation	1/340	12/18614	0.1985097	0.408093	0.31049997	CD177	1
BP	GO:0044650	adhesion of symbiont to host cell	1/340	12/18614	0.1985097	0.408093	0.31049997	DPP4	1
BP	GO:0045161	neuronal ion channel	1/340	12/18614	0.1985097	0.408093	0.31049997	GLDN	1

BP	GO:0048103	clustering somatic stem cell division	1/340	12/18614	0.1985097	0.40893	0.3104997	TGFB2	1
BP	GO:0048681	negative regulation of axon regeneration	1/340	12/18614	0.1985097	0.40893	0.3104997	TNR	1
BP	GO:0051001	negative regulation of nitric-oxide synthase activity	1/340	12/18614	0.1985097	0.40893	0.3104997	CNR1	1
BP	GO:0051340	regulation of ligase activity	1/340	12/18614	0.1985097	0.40893	0.3104997	GCLM	1

BP	GO:0051549	positive regulation of keratinocyte migration	1/340	12/18614	0.198509 7	0.408093	0.310499 7	HAS2	1
BP	GO:0051798	positive regulation of hair follicle development	1/340	12/18614	0.198509 7	0.408093	0.310499 7	TGFB2	1
BP	GO:0060221	retinal rod cell differentiation	1/340	12/18614	0.198509 7	0.408093	0.310499 7	NTRK2	1
BP	GO:0061517	macrophage proliferation	1/340	12/18614	0.198509 7	0.408093	0.310499 7	CX3CL1	1
BP	GO:0070778	L-aspartate transm	1/340	12/18614	0.198509 7	0.408093	0.310499 7	SLC1A3	1

		embrane transport							
BP	GO:0071472	cellular response to salt stress	1/340	12/18614	0.19850597	0.408093	0.31049 97	CAPN3	1
BP	GO:0071493	cellular response to UV-B	1/340	12/18614	0.19850597	0.408093	0.31049 97	MFAP4	1
BP	GO:0072110	glomerular mesangial cell proliferation	1/340	12/18614	0.19850597	0.408093	0.31049 97	EGR1	1
BP	GO:0072683	T cell extravasation	1/340	12/18614	0.19850597	0.408093	0.31049 97	CCL2	1
BP	GO:00	AV node cell to	1/340	12/18614	0.198	0.408093	0.310	RYR2	1

	86067	bundle of His cell communication			5097		4997		
BP	GO:0090209	negative regulation of triglyceride metabolic process	1/340	12/18614	0.1985097	0.408093	0.3104997	PIK3CG	1
BP	GO:0097048	dendritic cell apoptotic process	1/340	12/18614	0.1985097	0.408093	0.3104997	CXCL12	1
BP	GO:0097264	self proteolysis	1/340	12/18614	0.1985097	0.408093	0.3104997	CAPN3	1

BP	GO:0098911	regulation of ventricular cardiac muscle cell action potential	1/340	12/18614	0.19850 97	0.408 93	0.310 4997	RYR2	1
BP	GO:1900272	negative regulation of long-term synaptic potentiation	1/340	12/18614	0.19850 97	0.408 93	0.310 4997	CX3CR1	1
BP	GO:1901503	ether biosynthetic process	1/340	12/18614	0.19850 97	0.408 93	0.310 4997	FASN	1

BP	GO:1902337	regulation of apoptotic process involved in morphogenesis	1/340	12/18614	0.1985097	0.40893	0.31049 97	TGFB2	1
BP	GO:1902950	regulation of dendritic spine maintenance	1/340	12/18614	0.1985097	0.40893	0.31049 97	FCGR2B	1
BP	GO:1903867	extraembryonic membrane development	1/340	12/18614	0.1985097	0.40893	0.31049 97	BMP5	1
BP	GO:19	negative regulati	1/340	12/18614	0.198	0.40893	0.310	ROBO2	1

	05809	on of synapse organization			5097		4997		
BP	GO:2000668	regulation of dendritic cell apoptotic process	1/340	12/18614	0.1985097	0.4089 3	0.3104997	CXCL12	1
BP	GO:2001198	regulation of dendritic cell differentiation	1/340	12/18614	0.1985097	0.408093	0.3104997	FCGR2B	1
BP	GO:0031331	positive regulation of cellular catabolic	11/340	450/18614	0.2024487	0.416819	0.3164898	BTG2/ZBTB20/TRIB3/PIM2/HMOX1/ZFP36/IL1B/PYHIN1/DCN/TRIB1/IL6	11

		process							
BP	GO:0014902	myotube differentiation	4/340	127/18614	0.2028944	0.4172252	0.3167982	NFATC2/ADAMTS15/CXCL10/CXCL9	4
BP	GO:0001523	retinoid metabolic process	3/340	85/18614	0.2031262	0.4172252	0.3167982	SDR16C5/RARRES2/AKR1C3	3
BP	GO:0044774	mitotic DNA integrity checkpoint signaling	3/340	85/18614	0.2031262	0.4172252	0.3167982	PPP1R10/IER3/DTL	3
BP	GO:0098586	cellular response to virus	3/340	85/18614	0.2031262	0.4172252	0.3167982	CCL5/IL6/CXCL10	3
BP	GO:00	skin development	8/340	308/18614	0.203	0.417	0.316	KRT7/TGFB2/NFKBIZ/ZFP36/AKR1C3/SFN/INHBA/ALOX15B	8

	43588				1934	2252	7982		
BP	GO:0002262	myeloid cell homeostasis	5/340	171/18614	0.2037418	0.417875	0.3172916	HMOX1/SLC7A11/ZFP36/INHBA/IL6	5
BP	GO:0002455	humoral immune response mediated by circulating immunoglobulin	2/340	46/18614	0.2051057	0.417875	0.3172916	FCGR2B/C1R	2
BP	GO:0003254	regulation of membrane depolarization	2/340	46/18614	0.2051057	0.417875	0.3172916	GCLM/DCN	2

BP	GO:0008038	neuron recognition	2/340	46/18614	0.205105 7	0.417875	0.317291 6	ROBO2/CNR1	2
BP	GO:0046427	positive regulation of receptor signaling pathway via JAK-STAT	2/340	46/18614	0.205105 7	0.417875	0.317291 6	CCL5/IL6	2
BP	GO:0055010	ventricular cardiac muscle tissue morphogenesis	2/340	46/18614	0.205105 7	0.417875	0.317291 6	RYR2/TGFB2	2
BP	GO:0071827	plasma lipoprotein particle	2/340	46/18614	0.205105 7	0.417875	0.317291 6	LIPG/PLA2G2A	2

		organization							
BP	GO:0072583	clathrin-dependent endocytosis	2/340	46/18614	0.20510 57	0.417875	0.3172916	ITGA4/SIGLEC1	2
BP	GO:2000300	regulation of synaptic vesicle exocytosis	2/340	46/18614	0.2051057	0.417875	0.3172916	P2RY1/CSPG5	2
BP	GO:2001222	regulation of neuron migration	2/340	46/18614	0.2051057	0.417875	0.3172916	SEMA6A/CX3CL1	2
BP	GO:0051222	positive regulation of protein transport	8/340	309/18614	0.2055503	0.417875	0.3172916	PTGS2/TGFB2/IL1B/SFN/MYH10/ANO1/CFTR/FGA	8

BP	GO:0009314	response to radiation	11/340	452/18614	0.2063205	0.4178875	0.3172916	CXCL12/PTGS2/EGR1/MFAP4/JUND/SLC7A11/VCAM1/SLC1A3/DTL/FOS/CXCL10	11
BP	GO:0008286	insulin receptor signaling pathway	4/340	128/18614	0.2066739	0.4178875	0.3172916	SLC39A14/RARRES2/IL1B/SOCS3	4
BP	GO:0007260	tyrosine phosphorylation of STAT protein	3/340	86/18614	0.207843	0.4178875	0.3172916	CCL5/IL6/SOCS3	3
BP	GO:0015807	L-amino acid transport	3/340	86/18614	0.207843	0.4178875	0.3172916	SLC7A11/SLC1A3/SLC11A1	3
BP	GO:00	lipid storage	3/340	86/18614	0.207	0.417	0.317	PLIN2/IL1B/IL6	3

	19915				843	875	2916		
BP	GO:0021954	central nervous system neuron development	3/340	86/18614	0.207843	0.417875	0.317291 6	BTG2/NTRK2/NR4A2	3
BP	GO:0034103	regulation of tissue remodeling	3/340	86/18614	0.207843	0.417875	0.3172916	DDR2/TNFRSF11B/IL6	3
BP	GO:2000134	negative regulation of G1/S transition of mitotic cell cycle	3/340	86/18614	0.207843	0.417875	0.3172916	WEE1/INHBA/CCL2	3

BP	GO:0007179	transforming growth factor beta receptor signaling pathway	6/340	218/18614	0.20962 54	0.41787 5	0.31729 16	LTBP4/LTBP2/TGFB2/JUN/FOS/ASPN	6
BP	GO:0007259	receptor signaling pathway via JAK-STAT	5/340	173/18614	0.21020 86	0.41787 5	0.31729 16	STAT4/CCL5/IL6/SOCS3/CCL2	5
BP	GO:0045931	positive regulation of mitotic cell cycle	4/340	129/18614	0.21047 4	0.41787 5	0.31729 16	EIF4EBP1/PPP1R10/DDR2/DTL	4

BP	GO:2000027	regulation of animal organ morphogenesis	4/340	129/18614	0.210474	0.417875	0.3172916	HGF/ROBO2/TNFRSF11B/SULF1	4
BP	GO:0031018	endocrine pancreas development	2/340	47/18614	0.2118073	0.417875	0.3172916	BMP5/IL6	2
BP	GO:0042220	response to cocaine	2/340	47/18614	0.2118073	0.417875	0.3172916	CNR1/SLC1A3	2
BP	GO:0043277	apoptotic cell clearance	2/340	47/18614	0.2118073	0.417875	0.3172916	MARCO/CCL2	2
BP	GO:0045646	regulation of erythrocyte differentiation	2/340	47/18614	0.2118073	0.417875	0.3172916	ZFP36/INHBA	2

BP	GO:0046633	alpha-beta T cell proliferation	2/340	47/18614	0.2118073	0.4178875	0.3172916	IRF1/CD3E	2
BP	GO:0046677	response to antibiotic	2/340	47/18614	0.2118073	0.4178875	0.3172916	ALPL/SLC1A3	2
BP	GO:0061383	trabecula morphogenesis	2/340	47/18614	0.2118073	0.4178875	0.3172916	BMP5/TGFB2	2
BP	GO:0070849	response to epidermal growth factor	2/340	47/18614	0.2118073	0.4178875	0.3172916	ZFP36/FOS	2
BP	GO:0090199	regulation of release of cytochrome c from	2/340	47/18614	0.2118073	0.4178875	0.3172916	HGF/TNFSF10	2

		mitochondria							
BP	GO:0032092	positive regulation of protein binding	3/340	87/18614	0.2125843	0.4178875	0.3172916	TRIB3/ACE/CSF3	3
BP	GO:0032370	positive regulation of lipid transport	3/340	87/18614	0.2125843	0.4178875	0.3172916	ABCA8/IL1B/LIPG	3
BP	GO:0046889	positive regulation of lipid biosynthetic process	3/340	87/18614	0.2125843	0.4178875	0.3172916	ZBTB20/PTGS2/IL1B	3
BP	GO:0097006	regulation of plasma lipoprotein	3/340	87/18614	0.2125843	0.4178875	0.3172916	HMOX1/LIPG/PLA2G2A	3

		particle levels							
BP	GO:0000101	sulfur amino acid transport	1/340	13/18614	0.2131559	0.4178875	0.3172916	SLC7A11	1
BP	GO:0002524	hypersensitivity	1/340	13/18614	0.2131559	0.4178875	0.3172916	FCGR2B	1
BP	GO:0002638	negative regulation of immunoglobulin production	1/340	13/18614	0.2131559	0.4178875	0.3172916	FCGR2B	1
BP	GO:0002756	MyD88-independent toll-like recepto	1/340	13/18614	0.2131559	0.4178875	0.3172916	TNIP3	1

		r signaling pathway							
BP	GO:0002863	positive regulation of inflammatory response to antigenic stimulus	1/340	13/18614	0.213159	0.417875	0.3172916	CNR1	1
BP	GO:0003084	positive regulation of systemic arterial blood pressure	1/340	13/18614	0.213159	0.417875	0.3172916	ACE	1

BP	GO:0003334	keratinocyte development	1/340	13/18614	0.2131559	0.4178875	0.3172916	SFN	1
BP	GO:0006521	regulation of cellular amino acid metabolic process	1/340	13/18614	0.2131559	0.4178875	0.3172916	SLC7A11	1
BP	GO:0006586	indolalkylamine metabolic process	1/340	13/18614	0.2131559	0.4178875	0.3172916	IDO1	1
BP	GO:0006787	porphyrin-containing compound cataboli	1/340	13/18614	0.2131559	0.4178875	0.3172916	HMOX1	1

		c proces s							
BP	GO:0007320	insemination	1/340	13/18614	0.2131559	0.4178755	0.3172916	SLC6A4	1
BP	GO:0007512	adult heart development	1/340	13/18614	0.2131559	0.4178755	0.3172916	APLNR	1
BP	GO:0009214	cyclic nucleotide catabolic process	1/340	13/18614	0.2131559	0.4178755	0.3172916	PDE7B	1
BP	GO:0010896	regulation of triglyceride catabolic process	1/340	13/18614	0.2131559	0.4178755	0.3172916	PIK3CG	1

BP	GO:0032341	aldosterone metabolic process	1/340	13/18614	0.213159	0.417875	0.317291 6	BMP5	1
BP	GO:0032494	response to peptidoglycan	1/340	13/18614	0.213159	0.417875	0.317291 6	IL6	1
BP	GO:0032621	interleukin-18 production	1/340	13/18614	0.213159	0.417875	0.317291 6	GBP5	1
BP	GO:0032661	regulation of interleukin-18 production	1/340	13/18614	0.213159	0.417875	0.317291 6	GBP5	1
BP	GO:0032823	regulation of natural killer cell	1/340	13/18614	0.213159	0.417875	0.317291 6	RASGRP1	1

		differentiation							
BP	GO:0032966	negative regulation of collagen biosynthetic process	1/340	13/18614	0.213159	0.417875	0.3172916	IL6	1
BP	GO:0033015	tetrapyrrole catabolic process	1/340	13/18614	0.213159	0.417875	0.3172916	HMOX1	1
BP	GO:0033079	immature T cell proliferation	1/340	13/18614	0.213159	0.417875	0.3172916	IL1B	1
BP	GO:00	immature T cell	1/340	13/18614	0.213	0.417	0.317	IL1B	1

	33080	proliferation in thymus			159	875	2916			
BP	GO:0033605	positive regulation of catecholamine secretion	1/340	13/18614	0.213159	0.417875	0.3172916	CXCL12		1
BP	GO:0034616	response to laminar fluid shear stress	1/340	13/18614	0.213159	0.417875	0.3172916	ACE		1
BP	GO:0035745	T-helper 2 cell cytokine production	1/340	13/18614	0.213159	0.417875	0.3172916	IL6		1

BP	GO:0036006	cellular response to macrophage colony-stimulating factor stimulus	1/340	13/18614	0.213159	0.417875	0.3172916	STAP1	1
BP	GO:0036302	atrioventricular canal development	1/340	13/18614	0.213159	0.417875	0.3172916	HAS2	1
BP	GO:0036462	TRAIL-activated apoptotic signaling pathway	1/340	13/18614	0.213159	0.417875	0.3172916	ATF3	1

BP	GO:0042492	gamma-delta T cell differentiation	1/340	13/18614	0.213159	0.417875	0.3172916	EGR3		1
BP	GO:0045916	negative regulation of complement activation	1/340	13/18614	0.213159	0.417875	0.3172916	VSIG4		1
BP	GO:0048012	hepatocyte growth factor receptor signaling pathway	1/340	13/18614	0.213159	0.417875	0.3172916	HGF		1
BP	GO:00	negative regulati	1/340	13/18614	0.213	0.417	0.317	CASQ2		1

	51280	on of release of sequestered calcium ion into cytosol			159	875	2916		
BP	GO:0051918	negative regulation of fibrinolysis	1/340	13/18614	0.213159	0.417875	0.3172916	SERPINF2	1
BP	GO:0060100	positive regulation of phagocytosis, engulfment	1/340	13/18614	0.213159	0.417875	0.3172916	STAP1	1
BP	GO:0060670	branching involved in labyrint	1/340	13/18614	0.213159	0.417875	0.3172916	SOCS3	1

		hine layer morphogenesis							
BP	GO:0070254	mucus secretion	1/340	13/18614	0.2131559	0.4178875	0.3172916	ANO1	1
BP	GO:0070571	negative regulation of neuron projection regeneration	1/340	13/18614	0.2131559	0.4178875	0.3172916	TNR	1
BP	GO:0070572	positive regulation of neuron projection regeneration	1/340	13/18614	0.2131559	0.4178875	0.3172916	HGF	1

BP	GO:0071107	response to parathyroid hormone	1/340	13/18614	0.213159	0.417875	0.317291 6	FOS	1
BP	GO:0106049	regulation of cellular response to osmotic stress	1/340	13/18614	0.213159	0.417875	0.317291 6	PTGS2	1
BP	GO:1901678	iron coordination entity transport	1/340	13/18614	0.213159	0.417875	0.317291 6	LCN2	1
BP	GO:1901894	regulation of ATPase-coupled calcium	1/340	13/18614	0.213159	0.417875	0.317291 6	RYR2	1

		transmembrane transporter activity							
BP	GO:1902260	negative regulation of delayed rectifier potassium channel activity	1/340	13/18614	0.213159	0.417875	0.3172916	KCNAB1	1
BP	GO:1903054	negative regulation of extracellular matrix	1/340	13/18614	0.213159	0.417875	0.3172916	DPP4	1

		organization							
BP	GO:1903624	regulation of DNA catabolic process	1/340	13/18614	0.2131559	0.4178875	0.3172916	IL6	1
BP	GO:1904748	regulation of apoptotic process involved in development	1/340	13/18614	0.2131559	0.4178875	0.3172916	TGFB2	1
BP	GO:1904995	negative regulation of leukocyte adhesi	1/340	13/18614	0.2131559	0.4178875	0.3172916	CXCL12	1

		on to vascular endothelial cell							
BP	GO:1905064	negative regulation of vascular associated smooth muscle cell differentiation	1/340	13/18614	0.213159	0.417875	0.317291 6	NFATC2	1
BP	GO:1905065	positive regulation of vascular associated	1/340	13/18614	0.213159	0.417875	0.317291 6	CTH	1

BP	GO:1905155	smooth muscle cell differentiation positive regulation of membrane invagination	1/340	13/18614	0.2131559	0.4178875	0.3172916	STAP1	1
BP	GO:2000551	regulation of T-helper 2 cell cytokine production	1/340	13/18614	0.2131559	0.4178875	0.3172916	IL6	1
BP	GO:0016101	diterpenoid metabolic process	3/340	88/18614	0.2173489	0.4254322	0.3230298	SDR16C5/RARRES2/AKR1C3	3

BP	GO:0032273	positive regulation of protein polymerization	3/340	88/18614	0.217173489	0.425434322	0.323230298	FMN1/TPPP3/CSF3	3
BP	GO:0032413	negative regulation of ion transmembrane transporter activity	3/340	88/18614	0.217173489	0.425434322	0.323230298	KCNAB1/GEM/CASQ2	3
BP	GO:0031641	regulation of myelination	2/340	48/18614	0.218185299	0.425555582	0.323231255	HGF/EGR2	2
BP	GO:0031648	protein destabilization	2/340	48/18614	0.218185299	0.425555582	0.323231255	CAPN3/PYHIN1	2

BP	GO:0032620	interleukin-17 production	2/340	48/18614	0.21852 99	0.42555 82	0.32312 55	LY9/IL6	2
BP	GO:0032660	regulation of interleukin-17 production	2/340	48/18614	0.21852 99	0.42555 82	0.32312 55	LY9/IL6	2
BP	GO:0045047	protein targeting to ER	2/340	48/18614	0.21852 99	0.42555 82	0.32312 55	HYOU1/SRPRB	2
BP	GO:0045747	positive regulation of Notch signaling pathway	2/340	48/18614	0.21852 99	0.42555 82	0.32312 55	ROBO2/TGFB2	2
BP	GO:0046596	regulation of viral entry	2/340	48/18614	0.21852 99	0.42555 82	0.32312 55	CH25H/PTX3	2

		into host cell							
BP	GO:0060976	coronary vasculature development	2/340	48/18614	0.218529	0.4255582	0.3231255	APLNR/ACE	2
BP	GO:1903426	regulation of reactive oxygen species biosynthetic process	2/340	48/18614	0.218529	0.4255582	0.3231255	SLC18A2/DUOXA1	2
BP	GO:1903587	regulation of blood vessel endothelial cell	2/340	48/18614	0.218529	0.4255582	0.3231255	APLNR/HMOX1	2

		proliferation involved in sprouting angiogenesis							
BP	GO:0042692	muscle cell differentiation	10/340	410/18614	0.2187666	0.4258015	0.3233102	NFATC2/ANK2/ADAMTS15/APLNR/CAPN3/KRT19/CTH/MYH11/CXCL10/CXCL9	10
BP	GO:0002312	B cell activation involved in immune response	3/340	89/18614	0.2221356	0.4309941	0.3272529	NFKBIZ/FCGR2B/IL6	3
BP	GO:0043087	regulation of GTPase activity	9/340	364/18614	0.2231797	0.4309941	0.3272529	CCL5/FGD2/CCL23/RASGRP1/NTRK2/CX3CL1/CCL2/CCL8/CCL18	9

BP	GO:0010171	body morphogenesis	2/340	49/18614	0.225272706	0.430994141	0.327252529	ARID5B/CRISPLD2	2
BP	GO:0030838	positive regulation of actin filament polymerization	2/340	49/18614	0.225272706	0.430994141	0.327252529	FMN1/CSF3	2
BP	GO:0031640	killing of cells of another organism	2/340	49/18614	0.225272706	0.430994141	0.327252529	LYZ/S100A12	2
BP	GO:0043114	regulation of vascular permeability	2/340	49/18614	0.225272706	0.430994141	0.327252529	GPR4/PLVAP	2

BP	GO:0045933	positive regulation of muscle contraction	2/340	49/18614	0.225272706	0.430999941	0.327252529	ENO1/PTGS2	2
BP	GO:0046850	regulation of bone remodeling	2/340	49/18614	0.225272706	0.430999941	0.327252529	TNFRSF11B/IL6	2
BP	GO:0061028	establishment of endothelial barrier	2/340	49/18614	0.225272706	0.430999941	0.327252529	FASN/IL1B	2
BP	GO:0071470	cellular response to osmotic stress	2/340	49/18614	0.225272706	0.430999941	0.327252529	PTGS2/CAPN3	2
BP	GO:0071825	protein-lipid complex	2/340	49/18614	0.225272706	0.430999941	0.327252529	LIPG/PLA2G2A	2

		subunit organization							
BP	GO:0072337	modified amino acid transport	2/340	49/18614	0.2252706	0.4309941	0.3272529	SLC7A11/SLC11A1	2
BP	GO:0140895	cell surface toll-like receptor signaling pathway	2/340	49/18614	0.2252706	0.4309941	0.3272529	TRIL/TNIP3	2
BP	GO:1904752	regulation of vascular associated smooth muscle	2/340	49/18614	0.2252706	0.4309941	0.3272529	DDR2/NR4A3	2

BP	GO:0010657	cell migration muscle cell apoptotic process	3/340	90/18614	0.22269432	0.43099941	0.32725 29	RPS6KA2/PPP1R10/HMOX1	3
BP	GO:0022406	membrane docking	3/340	90/18614	0.22269432	0.43099941	0.32725 29	SYTL2/VCAM1/CFTR	3
BP	GO:0001976	nervous system process involved in regulation of systemic arterial blood	1/340	14/18614	0.22775414	0.43099941	0.32725 29	RPS6KA2	1

BP	GO:0003222	pressure ventricular trabecula myocardium morphogenesis	1/340	14/18614	0.22754 14	0.43099 41	0.32725 29	TGFB2	1
BP	GO:0006177	GMP biosynthetic process	1/340	14/18614	0.22754 14	0.43099 41	0.32725 29	AMPD1	1
BP	GO:0006617	SRP-dependent cotranslational protein targeting to membrane, signal	1/340	14/18614	0.22754 14	0.43099 41	0.32725 29	SRPRB	1

		sequence recognition							
BP	GO:0007638	mechanosensory behavior	1/340	14/18614	0.2275414	0.43099941	0.32725529	SLC1A3	1
BP	GO:0008212	mineralocorticoid metabolic process	1/340	14/18614	0.2275414	0.43099941	0.32725529	BMP5	1
BP	GO:0009812	flavonoid metabolic process	1/340	14/18614	0.2275414	0.43099941	0.32725529	SULT1B1	1
BP	GO:0010713	negative regulation of	1/340	14/18614	0.2275414	0.43099941	0.32725529	IL6	1

		collagen metabolic process							
BP	GO:0014061	regulation of norepinephrine secretion	1/340	14/18614	0.2275414	0.4309941	0.3272529	P2RY1	1
BP	GO:0014733	regulation of skeletal muscle adaptation	1/340	14/18614	0.2275414	0.4309941	0.3272529	GTF2IRD1	1
BP	GO:0015670	carbon dioxide transport	1/340	14/18614	0.2275414	0.4309941	0.3272529	HBB	1
BP	GO:0016322	neuron remodeling	1/340	14/18614	0.2275414	0.4309941	0.3272529	CX3CL1	1

BP	GO:0031665	negative regulation of lipopolysaccharide-mediated signaling pathway	1/340	14/18614	0.2275414	0.4309941	0.3272529	TRIB1	1
BP	GO:0032306	regulation of prostaglandin secretion	1/340	14/18614	0.2275414	0.4309941	0.3272529	IL1B	1
BP	GO:0032308	positive regulation of prostaglandin secretion	1/340	14/18614	0.2275414	0.4309941	0.3272529	IL1B	1

BP	GO:0034309	primary alcohol biosynthetic process	1/340	14/18614	0.2275414	0.4309941	0.3272529	BMP5	1
BP	GO:0042182	ketone catabolic process	1/340	14/18614	0.2275414	0.4309941	0.3272529	NQO2	1
BP	GO:0042541	hemoglobin biosynthetic process	1/340	14/18614	0.2275414	0.4309941	0.3272529	INHBA	1
BP	GO:0042762	regulation of sulfur metabolic process	1/340	14/18614	0.2275414	0.4309941	0.3272529	SLC7A11	1

BP	GO:0048243	norepinephrine secretion	1/340	14/18614	0.2275414	0.4309941	0.3272529	P2RY1	1
BP	GO:0050930	induction of positive chemotaxis	1/340	14/18614	0.2275414	0.4309941	0.3272529	CXCL12	1
BP	GO:0051127	positive regulation of actin nucleation	1/340	14/18614	0.2275414	0.4309941	0.3272529	FMN1	1
BP	GO:0051547	regulation of keratinocyte migration	1/340	14/18614	0.2275414	0.4309941	0.3272529	HAS2	1
BP	GO:0051608	histamine transport	1/340	14/18614	0.2275414	0.4309941	0.3272529	SLC18A2	1

BP	GO:0051764	actin crosslink formation	1/340	14/18614	0.22754 14	0.43099 41	0.32725 29	DPYSL3	1
BP	GO:0060732	positive regulation of inositol phosphate biosynthetic process	1/340	14/18614	0.22754 14	0.43099 41	0.32725 29	P2RY1	1
BP	GO:0071871	response to epinephrine	1/340	14/18614	0.22754 14	0.43099 41	0.32725 29	RYR2	1
BP	GO:0072350	tricarboxylic acid metabolic process	1/340	14/18614	0.22754 14	0.43099 41	0.32725 29	IDH1	1

BP	GO:1901029	negative regulation of mitochondrial outer membrane permeabilization involved in apoptotic signaling pathway	1/340	14/18614	0.22754 14	0.43099 41	0.32725 29	IER3		1
BP	GO:1901533	negative regulation of hematopoietic	1/340	14/18614	0.22754 14	0.43099 41	0.32725 29	ZFP36		1

		progenitor cell differentiation							
BP	GO:1901841	regulation of high voltage-gated calcium channel activity	1/340	14/18614	0.2275414	0.4309941	0.3272529	GEM	1
BP	GO:1902043	positive regulation of extrinsic apoptotic signaling pathway via death domain	1/340	14/18614	0.2275414	0.4309941	0.3272529	ATF3	1

		receptors								
BP	GO:1903798	regulation of miRNA maturation	1/340	14/18614	0.22754 14	0.43099 41	0.32725 29	IL6		1
BP	GO:1903909	regulation of receptor clustering	1/340	14/18614	0.22754 14	0.43099 41	0.32725 29	SLC7A11		1
BP	GO:1904321	response to forskolin	1/340	14/18614	0.22754 14	0.43099 41	0.32725 29	CFTR		1
BP	GO:1904322	cellular response to forskolin	1/340	14/18614	0.22754 14	0.43099 41	0.32725 29	CFTR		1
BP	GO:1905522	negative regulation of	1/340	14/18614	0.22754 14	0.43099 41	0.32725 29	STAP1		1

BP	GO:2001171	macrophage migration positive regulation of ATP biosynthetic process	1/340	14/18614	0.22754 14	0.43099 41	0.32725 29	ENO1		1
BP	GO:0002702	positive regulation of production of molecular mediator of immune response	4/340	134/18614	0.22975 93	0.43476 3	0.33011 46	PGC/IL1B/NR4A3/IL6		4

BP	GO:0009132	nucleoside diphosphate metabolic process	4/340	134/18614	0.2297593	0.434763	0.3301146	ZBTB20/ENO1/IER3/DDIT4	4
BP	GO:0043547	positive regulation of GTPase activity	7/340	272/18614	0.2306694	0.4362686	0.3312578	CCL5/CCL23/RASGRP1/CX3CL1/CCL2/CCL8/CCL18	7
BP	GO:0001974	blood vessel remodeling	2/340	50/18614	0.2320264	0.4375322	0.3322173	CBS/ACE	2
BP	GO:0002066	columnar/cuboidal epithelial cell development	2/340	50/18614	0.2320264	0.4375322	0.3322173	BMP5/FASN	2

BP	GO:0030195	negative regulation of blood coagulation	2/340	50/18614	0.2320264	0.4375322	0.3322173	SERPINF2/FGA	2
BP	GO:0038084	vascular endothelial growth factor signaling pathway	2/340	50/18614	0.2320264	0.4375322	0.3322173	DCN/SEMA6A	2
BP	GO:0045776	negative regulation of blood pressure	2/340	50/18614	0.2320264	0.4375322	0.3322173	CNR1/IER3	2

BP	GO:1904894	positive regulation of receptor signaling pathway via STAT	2/340	50/18614	0.2320264	0.43753222	0.3322173	CCL5/IL6	2
BP	GO:0019058	viral life cycle	8/340	320/18614	0.23216661	0.43757592	0.33225529	CCL5/DPP4/SIGLEC1/ANPEP/CH25H/PTX3/CCL2/CCL8	8
BP	GO:0043467	regulation of generation of precursor metabolites and energy	4/340	135/18614	0.2336669	0.43960049	0.33379911	ZBTB20/SHMT2/IER3/DDIT4	4

BP	GO:0051098	regulation of binding	9/340	369/18614	0.2346264	0.4396049	0.3337911	P2RY1/TRIB3/JUN/ITGA4/ACE/HMOX1/CCL23/PYHIN1/CSF3	9
BP	GO:0046777	protein autophosphorylation	6/340	227/18614	0.2359834	0.4396049	0.3337911	PIM2/JUN/DDR2/ACE/FGR/NTRK2	6
BP	GO:0001656	metanephros development	3/340	92/18614	0.2366169	0.4396049	0.3337911	ROBO2/EGR1/FMN1	3
BP	GO:0009791	post-embryonic development	3/340	92/18614	0.2366169	0.4396049	0.3337911	SLC18A2/ARID5B/NR4A2	3
BP	GO:0048771	tissue remodeling	5/340	181/18614	0.2367044	0.4396049	0.3337911	CBS/DDR2/ACE/TNFRSF11B/IL6	5
BP	GO:0043113	receptor clustering	2/340	51/18614	0.2387946	0.4396049	0.3337911	ITGA4/SLC7A11	2

BP	GO:0043124	negative regulation of I-kappaB kinase/NF-kappaB signaling	2/340	51/18614	0.2387946	0.4396049	0.3337911	RHOH/TNIP3	2
BP	GO:0043392	negative regulation of DNA binding	2/340	51/18614	0.2387946	0.4396049	0.3337911	JUN/HMOX1	2
BP	GO:0044818	mitotic G2/M transition checkpoint	2/340	51/18614	0.2387946	0.4396049	0.3337911	IER3/DTL	2
BP	GO:0045912	negative regulation of	2/340	51/18614	0.2387946	0.4396049	0.3337911	IER3/DDIT4	2

		carbohydrate metabolic process							
BP	GO:0060612	adipose tissue development	2/340	51/18614	0.2387946	0.4396049	0.3337911	ARID5B/SH3PXD2B	2
BP	GO:0060688	regulation of morphogenesis of a branching structure	2/340	51/18614	0.2387946	0.4396049	0.3337911	HGF/SULF1	2
BP	GO:0061178	regulation of insulin secretion involved in	2/340	51/18614	0.2387946	0.4396049	0.3337911	ANO1/CFTR	2

		cellular response to glucose stimulus							
BP	GO:1900047	negative regulation of hemostasis	2/340	51/18614	0.2387946	0.4396049	0.3337911	SERPINF2/FGA	2
BP	GO:1904738	vascular associated smooth muscle cell migration	2/340	51/18614	0.2387946	0.4396049	0.3337911	DDR2/NR4A3	2
BP	GO:0008202	steroid metabolic process	8/340	323/18614	0.2396302	0.4396049	0.3337911	AKR1C2/BMP5/EPHX2/EGR1/DHCR7/AKR1C3/CFTR/CH25H	8

BP	GO:0006941	striated muscle contraction	5/340	182/18614	0.240081	0.4396049	0.3337911	RYR2/ANK2/PIK3CG/CASQ2/NR4A1	5
BP	GO:1905952	regulation of lipid localization	5/340	182/18614	0.240081	0.4396049	0.3337911	PLIN2/ABCA8/IL1B/LIPG/IL6	5
BP	GO:0000463	maturation of LSU-rRNA from tricistronic rRNA transcript (SSU-rRNA, 5.8S rRNA, LSU-rRNA)	1/340	15/18614	0.2416616	0.4396049	0.3337911	WDR12	1

BP	GO:0002679	respiratory burst involved in defense response	1/340	15/18614	0.241 6616	0.439 6049	0.333 7911	PIK3CG	1
BP	GO:0003376	sphingosine-1-phosphate receptor signaling pathway	1/340	15/18614	0.241 6616	0.439 6049	0.333 7911	PIK3CG	1
BP	GO:0010649	regulation of cell communication by	1/340	15/18614	0.241 6616	0.439 6049	0.333 7911	CASQ2	1

		electrical coupling								
BP	GO:0010820	positive regulation of T cell chemotaxis	1/340	15/18614	0.24166616	0.4396049	0.33337911	CCL5		1
BP	GO:0010838	positive regulation of keratinocyte proliferation	1/340	15/18614	0.24166616	0.4396049	0.33337911	HAS2		1
BP	GO:0010889	regulation of sequestering of triglyceride	1/340	15/18614	0.24166616	0.43960449	0.33337911	PLIN2		1

BP	GO:0018410	C-terminal protein amino acid modification	1/340	15/18614	0.241661 6	0.439604 9	0.333791 1	AGBL1	1
BP	GO:0030201	heparan sulfate proteoglycan metabolic process	1/340	15/18614	0.241661 6	0.439604 9	0.333791 1	SULF1	1
BP	GO:0032352	positive regulation of hormone metabolic process	1/340	15/18614	0.241661 6	0.439604 9	0.333791 1	EGR1	1

BP	GO:0032897	negative regulation of viral transcription	1/340	15/18614	0.24166616	0.4396049	0.33337911	ZFP36	1
BP	GO:0033008	positive regulation of mast cell activation involved in immune response	1/340	15/18614	0.24166616	0.4396049	0.33337911	FGR	1
BP	GO:0035067	negative regulation of histone	1/340	15/18614	0.24166616	0.4396049	0.33337911	SDR16C5	1

		acetylation							
BP	GO:0035358	regulation of peroxisome proliferator activated receptor signaling pathway	1/340	15/18614	0.24166616	0.4396049	0.3337911	ALOX15B	1
BP	GO:0042532	negative regulation of tyrosine phosphorylation of	1/340	15/18614	0.24166616	0.4396049	0.3337911	SOCS3	1

BP	GO:0042976	STAT protein activation of Janus kinase activity	1/340	15/18614	0.24166616	0.4396049	0.33337911	CCL5	1
BP	GO:0043101	purine-containing compound salvage	1/340	15/18614	0.24166616	0.4396049	0.33337911	AMPD1	1
BP	GO:0043252	sodium-independent organic anion transport	1/340	15/18614	0.24166616	0.4396049	0.33337911	SLCO4A1	1
BP	GO:0043306	positive regulation of mast cell	1/340	15/18614	0.24166616	0.4396049	0.33337911	FGR	1

BP	GO:0043374	degranulation CD8-positive, alpha-beta T cell differentiation	1/340	15/18614	0.2416616	0.4396049	0.33337911	IRF1	1
BP	GO:0043455	regulation of secondary metabolic process	1/340	15/18614	0.2416616	0.4396049	0.33337911	SLC7A11	1
BP	GO:0044406	adhesion of symbiont to host	1/340	15/18614	0.2416616	0.4396049	0.33337911	DPP4	1
BP	GO:0045475	locomotor rhythm	1/340	15/18614	0.2416616	0.4396049	0.33337911	EGR1	1

BP	GO:0045986	negative regulation of smooth muscle contraction	1/340	15/18614	0.24166616	0.4396049	0.33337911	PTGS2	1
BP	GO:0046322	negative regulation of fatty acid oxidation	1/340	15/18614	0.24166616	0.4396049	0.33337911	CNR1	1
BP	GO:0046348	amino sugar catabolic process	1/340	15/18614	0.24166616	0.4396049	0.33337911	CHIT1	1
BP	GO:0048021	regulation of melanin	1/340	15/18614	0.24166616	0.4396049	0.33337911	SLC7A11	1

		biosynthetic process							
BP	GO:0048070	regulation of developmental pigmentation	1/340	15/18614	0.24166616	0.43960049	0.33337911	ADAMTS9	1
BP	GO:0048820	hair follicle maturation	1/340	15/18614	0.24166616	0.43960049	0.33337911	TGFB2	1
BP	GO:0048934	peripheral nervous system neuron differentiation	1/340	15/18614	0.24166616	0.43960049	0.33337911	NTRK2	1
BP	GO:00	peripheral nervou	1/340	15/18614	0.241	0.439	0.333	NTRK2	1

	48935	s system neuron development			6616	6049	7911		
BP	GO:0050966	detection of mechanical stimulus involved in sensory perception of pain	1/340	15/18614	0.2416616	0.4396049	0.3337911	CXCL12	1
BP	GO:0060026	convergent extension	1/340	15/18614	0.2416616	0.4396049	0.3337911	FRZB	1
BP	GO:0060099	regulation of phagocytosis,	1/340	15/18614	0.2416616	0.4396049	0.3337911	STAP1	1

BP	GO:0060413	engulfment atrial septum morphogenesis	1/340	15/18614	0.24166616	0.4396049	0.33337911	TGFB2	1
BP	GO:0060572	morphogenesis of an epithelial bud	1/340	15/18614	0.24166616	0.4396049	0.33337911	SULF1	1
BP	GO:0070365	hepatocyte differentiation	1/340	15/18614	0.24166616	0.4396049	0.33337911	FRZB	1
BP	GO:0070920	regulation of production of small RNA involved in gene silencin	1/340	15/18614	0.24166616	0.4396049	0.33337911	IL6	1

		g by RNA							
BP	GO:0071639	positive regulation of monocyte chemotactic protein-1 production	1/340	15/18614	0.24166 16	0.43960 49	0.33379 11	IL1B	1
BP	GO:0072283	metanephric renal vesicle morphogenesis	1/340	15/18614	0.24166 16	0.43960 49	0.33379 11	FMN1	1
BP	GO:0072378	blood coagulation, fibrin clot formation	1/340	15/18614	0.24166 16	0.43960 49	0.33379 11	FGA	1

BP	GO:0072531	pyrimidine-containing compound transmembrane transport	1/340	15/18614	0.24166616	0.4396049	0.33337911	AQP9	1
BP	GO:0098760	response to interleukin-7	1/340	15/18614	0.24166616	0.4396049	0.33337911	IL2RG	1
BP	GO:0098761	cellular response to interleukin-7	1/340	15/18614	0.24166616	0.4396049	0.33337911	IL2RG	1
BP	GO:0110156	methylguanosine-cap decapping	1/340	15/18614	0.24166616	0.4396049	0.33337911	ZFP36	1

BP	GO:0120305	regulation of pigmentation	1/340	15/18614	0.24166 16	0.43960 49	0.33379 11	ADAMTS9	1
BP	GO:1900153	positive regulation of nuclear-transcribed mRNA catabolic process, deadenylation-dependent decay	1/340	15/18614	0.24166 16	0.43960 49	0.33379 11	ZFP36	1
BP	GO:1900376	regulation of secondary metabo	1/340	15/18614	0.24166 16	0.43960 49	0.33379 11	SLC7A11	1

		lite biosynthetic process							
BP	GO:1900426	positive regulation of defense response to bacterium	1/340	15/18614	0.24166 16	0.43960 49	0.33379 11	PGC	1
BP	GO:1901722	regulation of cell proliferation involved in kidney development	1/340	15/18614	0.24166 16	0.43960 49	0.33379 11	EGR1	1

BP	GO:1903826	L-arginine transmembrane transport	1/340	15/18614	0.24166616	0.43960049	0.33337911	SLC11A1	1
BP	GO:1904936	interneuron migration	1/340	15/18614	0.24166616	0.43960049	0.33337911	FAT3	1
BP	GO:1905153	regulation of membrane invagination	1/340	15/18614	0.24166616	0.43960049	0.33337911	STAP1	1
BP	GO:2000402	negative regulation of lymphocyte migration	1/340	15/18614	0.24166616	0.43960049	0.33337911	CCL2	1

BP	GO:0030099	myeloid cell differentiation	10/340	421/18614	0.242454564	0.440848404	0.334727292	JUN/FASN/JUNB/HMGB3/TNFRSF11B/ZFP36/TRIB1/FOS/INHBA/CSF3	10
BP	GO:0006109	regulation of carbohydrate metabolic process	5/340	183/18614	0.243434707	0.442474734	0.335359691	P2RY1/ZBTB20/IER3/DDIT4/HAS2	5
BP	GO:1904951	positive regulation of establishment of protein localization	8/340	325/18614	0.244446515	0.443435434	0.336367816	PTGS2/TGFB2/IL1B/SFN/MYH10/ANO1/CFTR/FGA	8
BP	GO:0031669	cellular response to nutrient levels	6/340	230/18614	0.244449902	0.443435434	0.336367816	PLIN2/HMOX1/AKR1C3/ATF3/FOS/POSTN	6

BP	GO:0060048	cardiac muscle contraction	4/340	138/18614	0.245492 6	0.443543 4	0.336781 6	RYR2/ANK2/PIK3CG/CASQ2	4
BP	GO:0002715	regulation of natural killer cell mediated immunity	2/340	52/18614	0.245572 4	0.443543 4	0.336781 6	CD96/RASGRP1	2
BP	GO:0010543	regulation of platelet activation	2/340	52/18614	0.245572 4	0.443543 4	0.336781 6	MMRN1/IL6	2
BP	GO:0010656	negative regulation of muscle cell apoptotic	2/340	52/18614	0.245572 4	0.443543 4	0.336781 6	PPP1R10/HMOX1	2

		process							
BP	GO:0019933	cAMP-mediated signaling	2/340	52/18614	0.2455724	0.4435434	0.3367816	PDE7B/APLNR	2
BP	GO:0060324	face development	2/340	52/18614	0.2455724	0.4435434	0.3367816	ARID5B/CRISPLD2	2
BP	GO:0086009	membrane repolarization	2/340	52/18614	0.2455724	0.4435434	0.3367816	ANK2/CASQ2	2
BP	GO:0089718	amino acid import across plasma membrane	2/340	52/18614	0.2455724	0.4435434	0.3367816	SLC7A11/SLC1A3	2
BP	GO:0101023	vascular endothelial	2/340	52/18614	0.2455724	0.4435434	0.3367816	ITGA4/CCL2	2

		cell proliferation							
BP	GO:1901570	fatty acid derivative biosynthetic process	2/340	52/18614	0.2455724	0.4435434	0.3367816	HMGCLL1/ALOX15B	2
BP	GO:1905562	regulation of vascular endothelial cell proliferation	2/340	52/18614	0.2455724	0.4435434	0.3367816	ITGA4/CCL2	2
BP	GO:0006835	dicarboxylic acid transport	3/340	94/18614	0.2463608	0.4445459	0.3375427	SLC7A11/SLC1A3/NTRK2	3

BP	GO:1901379	regulation of potassium ion transmembrane transport	3/340	94/18614	0.24636608	0.44545459	0.33754427	KCNAB1/ANK2/CASQ2	3
BP	GO:0046578	regulation of Ras protein signal transduction	5/340	184/18614	0.24687832	0.4450049	0.33792948	TGFB2/PIK3CG/GPR4/P2RY10/RASGRP1	5
BP	GO:0097696	receptor signaling pathway via STAT	5/340	184/18614	0.24687832	0.4450049	0.33792948	STAT4/CCL5/IL6/SOCS3/CCL2	5
BP	GO:00	negative regulati	7/340	279/18614	0.249	0.448	0.340	TRIB3/DDR2/LAX1/ZFP36/IL1B/SFN/TRIB1	7

	51348	on of transferase activity			7084	3442	4268		
BP	GO:0044000	movement in host	5/340	185/18614	0.250288	0.4483442	0.3404268	DPP4/SIGLEC1/ANPEP/CH25H/PTX3	5
BP	GO:0045766	positive regulation of angiogenesis	5/340	185/18614	0.250288	0.4483442	0.3404268	HGF/APLNR/HMOX1/IL1B/CX3CR1	5
BP	GO:1904018	positive regulation of vasculature development	5/340	185/18614	0.250288	0.4483442	0.3404268	HGF/APLNR/HMOX1/IL1B/CX3CR1	5
BP	GO:0010659	cardiac muscle cell apoptotic	2/340	53/18614	0.2523573	0.4483442	0.3404268	RPS6KA2/PPP1R10	2

		process							
BP	GO:0010823	negative regulation of mitochondrion organization	2/340	53/18614	0.25235573	0.44834342	0.34042268	HGF/IER3	2
BP	GO:0010883	regulation of lipid storage	2/340	53/18614	0.25235573	0.44834342	0.34042268	PLIN2/IL6	2
BP	GO:0042572	retinol metabolic process	2/340	53/18614	0.25235573	0.44834342	0.34042268	SDR16C5/AKR1C3	2
BP	GO:0048146	positive regulation of fibroblast proliferation	2/340	53/18614	0.25235573	0.44834342	0.34042268	JUN/DDR2	2

BP	GO:0048260	positive regulation of receptor-mediated endocytosis	2/340	53/18614	0.25235573	0.44834342	0.34042268	SGIP1/SELE	2
BP	GO:0070169	positive regulation of biomineral tissue development	2/340	53/18614	0.25235573	0.44834342	0.34042268	PKDCC/CFTR	2
BP	GO:0008203	cholesterol metabolic process	4/340	140/18614	0.25344479	0.44834342	0.34042268	EPHX2/DHCR7/CFTR/CH25H	4
BP	GO:00	alcohol biosynthetic	4/340	140/18614	0.253	0.448	0.340	P2RY1/BMP5/DHCR7/CFTR	4

	46165	process			4479	3442	4268		
BP	GO:0044843	cell cycle G1/S phase transition	7/340	281/18614	0.2552355	0.4483442	0.3404268	EIF4EBP1/PIM2/WEE1/DDR2/BCAT1/INHBA/CCL2	7
BP	GO:0002713	negative regulation of B cell mediated immunity	1/340	16/18614	0.2555244	0.4483442	0.3404268	FCGR2B	1
BP	GO:0002890	negative regulation of immunoglobulin mediated	1/340	16/18614	0.2555244	0.4483442	0.3404268	FCGR2B	1

		immune response							
BP	GO:0003188	heart valve formation	1/340	16/18614	0.2555244	0.4483442	0.34042268	OLFM1	1
BP	GO:0006704	glucocorticoid biosynthetic process	1/340	16/18614	0.2555244	0.4483442	0.34042268	BMP5	1
BP	GO:0006735	NADH regeneration	1/340	16/18614	0.2555244	0.4483442	0.34042268	ENO1	1
BP	GO:0006825	copper ion transport	1/340	16/18614	0.2555244	0.4483442	0.34042268	CP	1
BP	GO:0010919	regulation of inositol phosph	1/340	16/18614	0.2555244	0.44834442	0.34042268	P2RY1	1

		ate biosynthetic process							
BP	GO:0014819	regulation of skeletal muscle contraction	1/340	16/18614	0.25552 44	0.44834 42	0.34042 68	NR4A1	1
BP	GO:0015671	oxygen transport	1/340	16/18614	0.25552 44	0.44834 42	0.34042 68	HBB	1
BP	GO:0021534	cell proliferation in hindbrain	1/340	16/18614	0.25552 44	0.44834 42	0.34042 68	SLC6A4	1
BP	GO:0021783	preganglionic parasympathetic fiber	1/340	16/18614	0.25552 44	0.44834 42	0.34042 68	EGR2	1

		development							
BP	GO:0021924	cell proliferation in external granule layer	1/340	16/18614	0.25552244	0.44834442	0.34042268	SLC6A4	1
BP	GO:0021930	cerebellar granule cell precursor proliferation	1/340	16/18614	0.25552244	0.44834442	0.34042268	SLC6A4	1
BP	GO:0030214	hyaluronan catabolic process	1/340	16/18614	0.25552244	0.44834442	0.34042268	LYVE1	1
BP	GO:00	regulation of granulo	1/340	16/18614	0.255	0.448	0.340	TRIB1	1

	30852	cyte differentiation			5244	3442	4268		
BP	GO:0034349	glial cell apoptotic process	1/340	16/18614	0.2555244	0.4483442	0.3404268	CCL2	1
BP	GO:0034374	low-density lipoprotein particle remodeling	1/340	16/18614	0.2555244	0.4483442	0.3404268	PLA2G2A	1
BP	GO:0034375	high-density lipoprotein particle remodeling	1/340	16/18614	0.2555244	0.4483442	0.3404268	LIPG	1
BP	GO:00	sodium ion export	1/340	16/18614	0.255	0.448	0.340	FXYD1	1

	36376	across plasma membrane			5244	3442	4268		
BP	GO:0038065	collagen-activated signaling pathway	1/340	16/18614	0.255525244	0.448343442	0.340404268	DDR2	1
BP	GO:0043517	positive regulation of DNA damage response, signal transduction by p53 class	1/340	16/18614	0.255525244	0.448343442	0.340404268	PYHIN1	1

BP	GO:0045187	mediator regulation of circadian sleep/wake cycle, sleep	1/340	16/18614	0.25552 44	0.44834 42	0.34042 68	PTGDS	1
BP	GO:0045651	positive regulation of macrophage differentiation	1/340	16/18614	0.25552 44	0.44834 42	0.34042 68	TRIB1	1
BP	GO:0046184	aldehyde biosynthetic process	1/340	16/18614	0.25552 44	0.44834 42	0.34042 68	BMP5	1
BP	GO:00	positive regulation of	1/340	16/18614	0.255	0.448	0.340	NR4A3	1

	46321	fatty acid oxidation			5244	3442	4268		
BP	GO:0046325	negative regulation of glucose import	1/340	16/18614	0.25552444	0.44834442	0.34042268	ACE	1
BP	GO:0047484	regulation of response to osmotic stress	1/340	16/18614	0.25552444	0.44834442	0.34042268	PTGS2	1
BP	GO:0048385	regulation of retinoic acid receptor signaling pathway	1/340	16/18614	0.25552444	0.44834442	0.34042268	AKR1C3	1

BP	GO:0048532	anatomical structure arrangement	1/340	16/18614	0.25552 44	0.44834 42	0.34042 68	EGR2	1
BP	GO:0050862	positive regulation of T cell receptor signaling pathway	1/340	16/18614	0.25552 44	0.44834 42	0.34042 68	TRAT1	1
BP	GO:0051044	positive regulation of membrane protein ectodomain proteolysis	1/340	16/18614	0.25552 44	0.44834 42	0.34042 68	IL1B	1

BP	GO:0051447	negative regulation of meiotic cell cycle	1/340	16/18614	0.25552 44	0.44834 42	0.34042 68	RPS6KA2	1
BP	GO:0055091	phospholipid homeostasis	1/340	16/18614	0.25552 44	0.44834 42	0.34042 68	LIPG	1
BP	GO:0060841	venous blood vessel development	1/340	16/18614	0.25552 44	0.44834 42	0.34042 68	APLNR	1
BP	GO:0061621	canonical glycolysis	1/340	16/18614	0.25552 44	0.44834 42	0.34042 68	ENO1	1
BP	GO:0061718	glucose catabolic process to	1/340	16/18614	0.25552 44	0.44834 42	0.34042 68	ENO1	1

BP	GO:0072578	pyruvate neurotransmitter-gated ion channel clustering	1/340	16/18614	0.25552244	0.44834442	0.34042268	SLC7A11	1
BP	GO:0090030	regulation of steroid hormone biosynthetic process	1/340	16/18614	0.25552244	0.44834442	0.34042268	BMP5	1
BP	GO:0090336	positive regulation of brown fat cell	1/340	16/18614	0.25552244	0.44834442	0.34042268	PTGS2	1

		differentiation							
BP	GO:1902033	regulation of hematopoietic stem cell proliferation	1/340	16/18614	0.25552 44	0.44834 42	0.34042 68	ACE	1
BP	GO:1902931	negative regulation of alcohol biosynthetic process	1/340	16/18614	0.25552 44	0.44834 42	0.34042 68	BMP5	1
BP	GO:1905664	regulation of calcium ion import across plasma	1/340	16/18614	0.25552 44	0.44834 42	0.34042 68	MS4A1	1

		membrane							
BP	GO:2000318	positive regulation of T-helper 17 type immune response	1/340	16/18614	0.25552 44	0.44834 42	0.34042 68	NFKBIZ	1
BP	GO:2000637	positive regulation of miRNA-mediated gene silencing	1/340	16/18614	0.25552 44	0.44834 42	0.34042 68	ZFP36	1
BP	GO:0032091	negative regulation of	3/340	96/18614	0.25616 61	0.44905 66	0.34096 77	ITGA4/ACE/CCL23	3

		protein binding							
BP	GO:0045807	positive regulation of endocytosis	3/340	96/18614	0.25616661	0.44905566	0.34096677	SGIP1/STAP1/SELE	3
BP	GO:0009395	phospholipid catabolic process	2/340	54/18614	0.25914468	0.45261662	0.34367705	IDH1/LIPG	2
BP	GO:0009409	response to cold	2/340	54/18614	0.25914468	0.45261662	0.34367705	FOS/CXCL10	2
BP	GO:0046164	alcohol catabolic process	2/340	54/18614	0.25914468	0.45261662	0.34367705	AKR1C3/SULT1B1	2
BP	GO:0050819	negative regulation of	2/340	54/18614	0.25914468	0.45261662	0.34367705	SERPINF2/FGA	2

BP	GO:0071385	coagulation cellular response to glucocorticoid stimulus	2/340	54/18614	0.2591468	0.4526162	0.3436705	ZFP36/DDIT4	2
BP	GO:0086002	cardiac muscle cell action potential involved in contraction	2/340	54/18614	0.2591468	0.4526162	0.3436705	RYR2/ANK2	2
BP	GO:1903170	negative regulation of calcium ion transm	2/340	54/18614	0.2591468	0.4526162	0.3436705	GEM/CASQ2	2

		embrane transport							
BP	GO:2000179	positive regulation of neural precursor cell proliferation	2/340	54/18614	0.2591468	0.4526162	0.3436705	CX3CR1/CX3CL1	2
BP	GO:0008593	regulation of Notch signaling pathway	3/340	97/18614	0.2610891	0.4555909	0.3459292	ROBO2/TGFB2/POSTN	3
BP	GO:1902807	negative regulation of cell cycle G1/S	3/340	97/18614	0.2610891	0.4555909	0.3459292	WEE1/INHBA/CCL2	3

		phase transition							
BP	GO:0001508	action potential	4/340	142/18614	0.261456	0.4556053	0.3459401	RYR2/ANK2/CNR1/NTRK2	4
BP	GO:0038061	NIK/NF-kappaB signaling	4/340	142/18614	0.261456	0.4556053	0.3459401	RTKN2/UACA/IL1B/CD27	4
BP	GO:2001056	positive regulation of cysteine-type endopeptidase activity	4/340	142/18614	0.261456	0.4556053	0.3459401	NLRC4/UACA/S100A8/TNFSF10	4
BP	GO:0030534	adult behavior	4/340	143/18614	0.2654784	0.4582984	0.347985	CXCL12/SLC7A11/NRXN3/NR4A2	4

BP	GO:0002043	blood vessel endothelial cell proliferation involved in sprouting angiogenesis	2/340	55/18614	0.26593 84	0.45829 84	0.34798 5	APLNR/HMOX1	2
BP	GO:0010518	positive regulation of phospholipase activity	2/340	55/18614	0.26593 84	0.45829 84	0.34798 5	CCL5/SELE	2
BP	GO:0010658	striated muscle cell apoptotic process	2/340	55/18614	0.26593 84	0.45829 84	0.34798 5	RPS6KA2/PPP1R10	2

BP	GO:0010761	fibroblast migration	2/340	55/18614	0.265938 4	0.458298 4	0.347985	ARID5B/DDR2	2
BP	GO:0034381	plasma lipoprotein particle clearance	2/340	55/18614	0.265938 4	0.458298 4	0.347985	HMOX1/LIPG	2
BP	GO:0043525	positive regulation of neuron apoptotic process	2/340	55/18614	0.265938 4	0.458298 4	0.347985	TGFB2/NQO1	2
BP	GO:0052372	modulation by symbiont of entry into host	2/340	55/18614	0.265938 4	0.458298 4	0.347985	CH25H/PTX3	2

BP	GO:0051952	regulation of amine transport	3/340	98/18614	0.2660243	0.45829984	0.34798 5	P2RY1/CXCL12/CNR1	3
BP	GO:0051495	positive regulation of cytoskeleton organization	5/340	190/18614	0.26753553	0.45829984	0.347985	SERPINF2/STAP1/FMN1/CX3CL1/CSF3	5
BP	GO:0001574	ganglioside biosynthetic process	1/340	17/18614	0.26913346	0.45829984	0.347985	ST6GALNAC3	1
BP	GO:0002076	osteoblast development	1/340	17/18614	0.26913346	0.45829984	0.347985	JUND	1
BP	GO:0003198	epithelial to mesenchymal	1/340	17/18614	0.26913346	0.45829984	0.347985	TGFB2	1

		transition involved in endocardial cushion formation							
BP	GO:0006103	2-oxoglutarate metabolic process	1/340	17/18614	0.26913 46	0.45829 84	0.34798 5	IDH1	1
BP	GO:0006570	tyrosine metabolic process	1/340	17/18614	0.26913 46	0.45829 84	0.34798 5	FAH	1
BP	GO:0006620	post-translational protein	1/340	17/18614	0.26913 46	0.45829 84	0.34798 5	HYOU1	1

		targeting to endoplasmic reticulum membrane							
BP	GO:0006677	glycosylceramide metabolic process	1/340	17/18614	0.2691346	0.4582984	0.3479985	ST6GALNAC3	1
BP	GO:0008228	opsonization	1/340	17/18614	0.2691346	0.4582984	0.3479985	PTX3	1
BP	GO:0009084	glutamine family amino acid biosynthetic	1/340	17/18614	0.2691346	0.4582984	0.3479985	PYCR1	1

		process							
BP	GO:0009251	glucan catabolic process	1/340	17/18614	0.26913346	0.45829984	0.347985	MGAM	1
BP	GO:0010763	positive regulation of fibroblast migration	1/340	17/18614	0.26913346	0.45829984	0.347985	DDR2	1
BP	GO:0016114	terpenoid biosynthetic process	1/340	17/18614	0.26913346	0.45829984	0.347985	AKR1C3	1
BP	GO:0021542	dentate gyrus development	1/340	17/18614	0.26913346	0.45829984	0.347985	BTG2	1

BP	GO:0030889	negative regulation of B cell proliferation	1/340	17/18614	0.26913 46	0.45829 84	0.34798 5	FCGR2B	1
BP	GO:0030949	positive regulation of vascular endothelial growth factor receptor signaling pathway	1/340	17/18614	0.26913 46	0.45829 84	0.34798 5	IL1B	1
BP	GO:0031053	primary miRNA processing	1/340	17/18614	0.26913 46	0.45829 84	0.34798 5	IL6	1

BP	GO:0032274	gonadotropin secretion	1/340	17/18614	0.26913 46	0.45829 84	0.34798 5	INHBA	1
BP	GO:0032488	Cdc42 protein signal transduction	1/340	17/18614	0.26913 46	0.45829 84	0.34798 5	RHOBTB2	1
BP	GO:0033623	regulation of integrin activation	1/340	17/18614	0.26913 46	0.45829 84	0.34798 5	SKAP1	1
BP	GO:0034035	purine ribonucleoside bisphosphate metabolic process	1/340	17/18614	0.26913 46	0.45829 84	0.34798 5	SULT1B1	1
BP	GO:00	protein O-linked	1/340	17/18614	0.269	0.458	0.347	TMTC1	1

	35269	mannosylation			1346	2984	985		
BP	GO:0036065	fucosylation	1/340	17/18614	0.269131346	0.458298484	0.347985	FUT3	1
BP	GO:0042481	regulation of odontogenesis	1/340	17/18614	0.269131346	0.458298484	0.347985	TNFRSF11B	1
BP	GO:0043923	positive regulation by host of viral transcription	1/340	17/18614	0.269131346	0.458298484	0.347985	JUN	1
BP	GO:0044320	cellular response to leptin stimulus	1/340	17/18614	0.269131346	0.458298484	0.347985	NR4A3	1
BP	GO:00	cell-cell junction	1/340	17/18614	0.269	0.458	0.347	CD177	1

	45217	maintenance			1346	2984	985		
BP	GO:0050427	3'-phosphoadenosine 5'-phosphosulfate metabolic process	1/340	17/18614	0.2691346	0.4582984	0.347985	SULT1B1	1
BP	GO:0051969	regulation of transmission of nerve impulse	1/340	17/18614	0.2691346	0.4582984	0.347985	TNR	1
BP	GO:0060148	positive regulation of post-transcriptional gene	1/340	17/18614	0.2691346	0.4582984	0.347985	ZFP36	1

		silencing							
BP	GO:0060965	negative regulation of miRNA-mediated gene silencing	1/340	17/18614	0.2691346	0.4582984	0.347985	IL6	1
BP	GO:0061158	3'-UTR-mediated mRNA destabilization	1/340	17/18614	0.2691346	0.4582984	0.347985	ZFP36	1
BP	GO:0071468	cellular response to acidic pH	1/340	17/18614	0.2691346	0.4582984	0.347985	SCNN1G	1

BP	GO:0072224	metanephric glomerulus development	1/340	17/18614	0.26913346	0.4582984	0.347985	EGR1	1
BP	GO:0097154	GABAergic neuron differentiation	1/340	17/18614	0.26913346	0.4582984	0.347985	INHBA	1
BP	GO:1900006	positive regulation of dendrite development	1/340	17/18614	0.26913346	0.4582984	0.347985	BMP5	1
BP	GO:1900370	positive regulation of post-transcriptional gene silencin	1/340	17/18614	0.26913346	0.4582984	0.347985	ZFP36	1

		g by RNA								
BP	GO:1901201	regulation of extracellular matrix assembly	1/340	17/18614	0.26913346	0.4582984	0.347985	HAS2		1
BP	GO:1901550	regulation of endothelial cell development	1/340	17/18614	0.26913346	0.4582984	0.347985	IL1B		1
BP	GO:1902036	regulation of hematopoietic stem cell differentiation	1/340	17/18614	0.26913346	0.4582984	0.347985	ZFP36		1

BP	GO:1903140	regulation of establishment of endothelial barrier	1/340	17/18614	0.26913 46	0.45829 84	0.34798 5	IL1B	1
BP	GO:1904948	midbrain dopaminergic neuron differentiation	1/340	17/18614	0.26913 46	0.45829 84	0.34798 5	NR4A2	1
BP	GO:0042391	regulation of membrane potential	10/340	433/18614	0.26929 59	0.45836 83	0.34803 81	RYR2/FXYD1/PYCR1/GCLM/ANK2/CNR1/CASQ2/DCN/CFTR/NTRK2	10
BP	GO:0006493	protein O-linked glycosylation	3/340	99/18614	0.27097 06	0.46068 02	0.34979 35	FUT3/GXYLT2/TMTC1	3

BP	GO:0006721	terpenoid metabolic process	3/340	99/18614	0.2709706	0.4606802	0.3497935	SDR16C5/RARRES2/AKR1C3	3
BP	GO:0050777	negative regulation of immune response	5/340	191/18614	0.27101 68	0.4606802	0.3497935	CD96/HMOX1/FGR/VSIG4/FCGR2B	5
BP	GO:0033673	negative regulation of kinase activity	6/340	239/18614	0.2725769	0.46194 36	0.35075 28	TRIB3/DDR2/LAX1/IL1B/SFN/TRIB1	6
BP	GO:0002707	negative regulation of lymphocyte	2/340	56/18614	0.27272 98	0.46194 36	0.35075 28	CD96/FCGR2B	2

		mediated immunity							
BP	GO:0002752	cell surface pattern recognition receptor signaling pathway	2/340	56/18614	0.2727298	0.4619436	0.3507528	TRIL/TNIP3	2
BP	GO:0006695	cholesterol biosynthetic process	2/340	56/18614	0.2727298	0.4619436	0.3507528	DHCR7/CFTR	2
BP	GO:0032608	interferon-beta production	2/340	56/18614	0.2727298	0.4619436	0.3507528	ZBTB20/IRF1	2

BP	GO:0032648	regulation of interferon-beta production	2/340	56/18614	0.2727298	0.4619436	0.3507528	ZBTB20/IRF1	2
BP	GO:0060986	endocrine hormone secretion	2/340	56/18614	0.2727298	0.4619436	0.3507528	IL1B/INHBA	2
BP	GO:1902653	secondary alcohol biosynthetic process	2/340	56/18614	0.2727298	0.4619436	0.3507528	DHCR7/CFTR	2
BP	GO:0055001	muscle cell development	5/340	192/18614	0.2745081	0.4647491	0.352883	NFATC2/ANK2/CAPN3/KRT19/MYH11	5
BP	GO:00	epidermal cell	6/340	240/18614	0.275	0.466	0.354	KRT7/NFKBIZ/ZFP36/AKR1C3/SFN/ALOX15B	6

	09913	differentiation			689	5412	2438		
BP	GO:0032755	positive regulation of interleukin-6 production	3/340	100/18614	0.275927	0.4667367	0.3543922	ZBTB20/IL1B/IL6	3
BP	GO:0032970	regulation of actin filament-based process	9/340	387/18614	0.2774594	0.4668667	0.3544909	RYR2/CXCL12/ANK2/SERPINF2/STAP1/PLEKHH2/FMN1/CX3CL1/CSF3	9
BP	GO:0001541	ovarian follicle development	2/340	57/18614	0.2795187	0.4668667	0.3544909	INHBA/PTX3	2
BP	GO:0010332	response to gamma radiation	2/340	57/18614	0.2795187	0.4668667	0.3544909	EGR1/CXCL10	2

BP	GO:0032768	regulation of monooxygenase activity	2/340	57/18614	0.27951587	0.4668667	0.35464909	CNR1/IL1B	2
BP	GO:0046148	pigment biosynthetic process	2/340	57/18614	0.27951587	0.4668667	0.35464909	TRPC1/SLC7A11	2
BP	GO:0022411	cellular component disassembly	11/340	488/18614	0.28089490	0.46686667	0.35464909	PLIN2/NCKAP5/ADAMTS15/DDR2/PLEKHH2/SH3PXD2B/DPP4/DDIT4/IL6/CX3CR1/CX3CL1	11
BP	GO:1901991	negative regulation of mitotic cell cycle phase	5/340	194/18614	0.28151586	0.46686667	0.35464909	WEE1/IER3/DTL/INHBA/CCL2	5

BP	GO:0006820	transition monoatomic anion transport	4/340	147/18614	0.28167667	0.46686667	0.35449909	FXYD1/ANO1/SLC1A3/CFTR	4
BP	GO:0006865	amino acid transport	4/340	147/18614	0.28167667	0.46686667	0.35449909	SLC7A11/SLC1A3/SLC11A1/NTRK2	4
BP	GO:0045667	regulation of osteoblast differentiation	4/340	147/18614	0.28167667	0.46686667	0.35449909	HGF/DDR2/JUND/IL6	4
BP	GO:0001696	gastric acid secretion	1/340	18/18614	0.28249666	0.46686667	0.35449909	HRH2	1
BP	GO:0002923	regulation of humoral immun	1/340	18/18614	0.28249666	0.46686667	0.35449909	FCGR2B	1

		e response mediated by circulating immunoglobulin							
BP	GO:0003128	heart field specification	1/340	18/18614	0.2824966	0.46686 67	0.35449 09	ROBO2	1
BP	GO:0003159	morphogenesis of an endothelium	1/340	18/18614	0.28249 66	0.46686 67	0.35449 09	CXCL10	1
BP	GO:0006957	complement activation, alternative	1/340	18/18614	0.28249 66	0.46686 67	0.35449 09	VSIG4	1

		pathway DNA damage response, signal transduction by p53 class mediator resulting in transcription of p21 class mediator							
BP	GO:0006978		1/340	18/18614	0.28249666	0.46686667	0.35449909	PYHIN1	1
BP	GO:0009313	oligosaccharide cataboli	1/340	18/18614	0.28249666	0.46686667	0.35449909	MGAM	1

		c process							
BP	GO:0019896	axonal transport of mitochondrion	1/340	18/18614	0.2824966	0.4668667	0.35454909	SYBU	1
BP	GO:0020027	hemoglobin metabolic process	1/340	18/18614	0.2824966	0.4668667	0.35454909	INHBA	1
BP	GO:0032769	negative regulation of monooxygenase activity	1/340	18/18614	0.2824966	0.4668667	0.35454909	CNR1	1
BP	GO:0034384	high-density lipoprotein	1/340	18/18614	0.2824966	0.46686667	0.35454909	LIPG	1

		particle clearance							
BP	GO:0035234	ectopic germ cell programmed cell death	1/340	18/18614	0.2824966	0.4668667	0.3544909	IL1B	1
BP	GO:0035455	response to interferon-alpha	1/340	18/18614	0.2824966	0.4668667	0.3544909	PYHIN1	1
BP	GO:0036003	positive regulation of transcription from RNA polymerase II promoter in	1/340	18/18614	0.2824966	0.4668667	0.3544909	ATF3	1

		response to stress							
BP	GO:0042402	cellular biogenic amine catabolic process	1/340	18/18614	0.28249666	0.46686667	0.35454909	MAOA	1
BP	GO:0043173	nucleotide salvage	1/340	18/18614	0.28249666	0.46686667	0.35454909	AMPD1	1
BP	GO:0044247	cellular polysaccharide catabolic process	1/340	18/18614	0.28249666	0.46686667	0.35454909	MGAM	1
BP	GO:0045945	positive regulation of transcri	1/340	18/18614	0.28249666	0.46686667	0.35454909	INHBA	1

		ption by RNA polymerase III							
BP	GO:0046040	IMP metabolic process	1/340	18/18614	0.28249966	0.46686667	0.35449909	AMPD1	1
BP	GO:0046112	nucleobase biosynthetic process	1/340	18/18614	0.28249966	0.46686667	0.35449909	SHMT2	1
BP	GO:0048486	parasympathetic nervous system development	1/340	18/18614	0.28249966	0.46686667	0.35449909	EGR2	1
BP	GO:00	regulation of lipoprot	1/340	18/18614	0.282	0.466	0.354	LIPG	1

	50746	ein metabolic process			4966	8667	4909		
BP	GO:0050802	circadian sleep/wake cycle, sleep	1/340	18/18614	0.2824966	0.4668667	0.3544909	PTGDS	1
BP	GO:0051770	positive regulation of nitric-oxide synthase biosynthetic process	1/340	18/18614	0.2824966	0.4668667	0.3544909	CCL2	1
BP	GO:0051917	regulation of fibrinolysis	1/340	18/18614	0.2824966	0.4668667	0.3544909	SERPINF2	1

BP	GO:0060149	negative regulation of post-transcriptional gene silencing	1/340	18/18614	0.2824966	0.4668667	0.3544909	IL6		1
BP	GO:0060967	negative regulation of gene silencing by RNA	1/340	18/18614	0.2824966	0.4668667	0.3544909	IL6		1
BP	GO:0061154	endothelial tube morphogenesis	1/340	18/18614	0.28249966	0.46686667	0.35449909	CXCL10		1
BP	GO:00	glycolytic proces	1/340	18/18614	0.282	0.466	0.354	ENO1		1

	61620	s through glucose-6-phosphate			4966	8667	4909		
BP	GO:0071071	regulation of phospholipid biosynthetic process	1/340	18/18614	0.2824966	0.4668667	0.3544909	IDH1	1
BP	GO:0071397	cellular response to cholesterol	1/340	18/18614	0.2824966	0.4668667	0.3544909	INHBA	1
BP	GO:0072077	renal vesicle morphogenesis	1/340	18/18614	0.2824966	0.4668667	0.3544909	FMN1	1
BP	GO:00	protein activation	1/340	18/18614	0.282	0.466	0.354	FGA	1

	72376	cascade			4966	8667	4909		
BP	GO:0090520	sphingolipid mediated signaling pathway	1/340	18/18614	0.2824966	0.4668667	0.3544909	PIK3CG	1
BP	GO:0097067	cellular response to thyroid hormone stimulus	1/340	18/18614	0.2824966	0.4668667	0.3544909	GCLM	1
BP	GO:0097094	craniofacial suture morphogenesis	1/340	18/18614	0.2824966	0.4668667	0.3544909	FREM1	1
BP	GO:01	RNA decapping	1/340	18/18614	0.282	0.466	0.354	ZFP36	1

	10154				4966	8667	4909			
BP	GO:0150079	negative regulation of neuroinflammatory response	1/340	18/18614	0.2824966	0.4668667	0.3544909	CX3CL1		1
BP	GO:1900369	negative regulation of post-transcriptional gene silencing by RNA	1/340	18/18614	0.2824966	0.4668667	0.3544909	IL6		1
BP	GO:1905209	positive regulation of cardioc	1/340	18/18614	0.2824966	0.4668667	0.3544909	TGFB2		1

		yte differentiation							
BP	GO:2000757	negative regulation of peptidyl-lysine acetylation	1/340	18/18614	0.2824966	0.4668667	0.3544909	SDR16C5	1
BP	GO:2000811	negative regulation of anoikis	1/340	18/18614	0.2824966	0.4668667	0.3544909	NTRK2	1
BP	GO:2001224	positive regulation of neuron migration	1/340	18/18614	0.2824966	0.4668667	0.3544909	SEMA6A	1
BP	GO:0031334	positive regulation of protein-	5/340	195/18614	0.2850371	0.47065669	0.35736388	SKAP1/FMN1/TPPP3/GBP5/CSF3	5

		containing complex assembly							
BP	GO:0071902	positive regulation of protein serine/threonine kinase activity	5/340	195/18614	0.2850371	0.47065669	0.35736388	PIK3CG/FGD2/IL1B/RASGRP1/S100A12	5
BP	GO:0030316	osteoclast differentiation	3/340	102/18614	0.2858661	0.4713177	0.35787706	JUNB/TNFRSF11B/FOS	3
BP	GO:1904063	negative regulation of cation transmembran	3/340	102/18614	0.2858661	0.4713177	0.35787706	KCNAB1/GEM/CASQ2	3

		e transport							
BP	GO:0015804	neutral amino acid transport	2/340	58/18614	0.286303	0.4713177	0.3578706	SLC7A11/SLC1A3	2
BP	GO:0030225	macrophage differentiation	2/340	58/18614	0.286303	0.4713177	0.3578706	TRIB1/INHBA	2
BP	GO:0048008	platelet-derived growth factor receptor signaling pathway	2/340	58/18614	0.286303	0.4713177	0.3578706	ARID5B/NR4A3	2
BP	GO:00	vascular endoth	2/340	58/18614	0.286	0.471	0.357	IL1B/SULF1	2

	48010	elial growth factor receptor signaling pathway			303	3177	8706		
BP	GO:0090311	regulation of protein deacetylation	2/340	58/18614	0.286303	0.4713177	0.3578706	SDR16C5/APLNR	2
BP	GO:0007369	gastrulation	5/340	196/18614	0.2885639	0.4748347	0.3605409	APLNR/ITGA4/NR4A3/COL8A1/INHBA	5
BP	GO:0008064	regulation of actin polymerization or depoly	4/340	149/18614	0.2898332	0.4767175	0.3619706	CXCL12/PLEKHH2/FMN1/CSF3	4

		merization							
BP	GO:0003333	amino acid transmembrane transport	3/340	103/18614	0.29084468	0.4779237	0.36288865	SLC7A11/SLC1A3/SLC11A1	3
BP	GO:0032677	regulation of interleukin-8 production	3/340	103/18614	0.29084468	0.4779237	0.36288865	IL1B/CD2/IL6	3
BP	GO:0002763	positive regulation of myeloid leukocyte differentiation	2/340	59/18614	0.29308805	0.4779237	0.36288865	TRIB1/FOS	2
BP	GO:00	membrane protein	2/340	59/18614	0.293	0.477	0.362	TIMP4/IL1B	2

	33619	proteolysis			0805	9237	8865		
BP	GO:0061351	neural precursor cell proliferation	4/340	150/18614	0.2939239	0.4779237	0.3628865	BTG2/CX3CR1/CX3CL1/SLC6A4	4
BP	GO:0001829	trophectodermal cell differentiation	1/340	19/18614	0.2956151	0.4779237	0.3628865	JUNB	1
BP	GO:0001845	phagolysosome assembly	1/340	19/18614	0.2956151	0.4779237	0.3628865	SRPX	1
BP	GO:0002281	macrophage activation involved in immune	1/340	19/18614	0.2956151	0.4779237	0.3628865	CX3CR1	1

		response							
BP	GO:0002283	neutrophil activation involved in immune response	1/340	19/18614	0.29561 51	0.47792 37	0.36288 65	CD177	1
BP	GO:0002755	MyD88-dependent toll-like receptor signaling pathway	1/340	19/18614	0.29561 51	0.47792 37	0.36288 65	IRF1	1
BP	GO:00	apoptotic DNA	1/340	19/18614	0.295	0.477	0.362	IL6	1

	06309	fragmentation			6151	9237	8865		
BP	GO:0006684	sphingomyelin metabolic process	1/340	19/18614	0.2956151	0.4779237	0.3628865	ABCA8	1
BP	GO:0006743	ubiquinone metabolic process	1/340	19/18614	0.2956151	0.4779237	0.3628865	NQO1	1
BP	GO:0010002	cardioblast differentiation	1/340	19/18614	0.2956151	0.4779237	0.3628865	TGFB2	1
BP	GO:0010801	negative regulation of peptidyl-threonine	1/340	19/18614	0.2956151	0.4779237	0.3628865	DDIT4	1

		phosphorylation							
BP	GO:0032026	response to magnesium ion	1/340	19/18614	0.29561 51	0.47792 37	0.36288 65	TNFRSF11B	1
BP	GO:0032695	negative regulation of interleukin-12 production	1/340	19/18614	0.29561 51	0.47792 37	0.36288 65	TIGIT	1
BP	GO:0042772	DNA damage response, signal transduction resulting in	1/340	19/18614	0.29561 51	0.47792 37	0.36288 65	PYHIN1	1

BP	GO:0044070	transcription regulation of monoatomic anion transport	1/340	19/18614	0.29561 51	0.47792 37	0.36288 65	CFTR	1
BP	GO:0045618	positive regulation of keratinocyte differentiation	1/340	19/18614	0.29561 51	0.47792 37	0.36288 65	ALOX15B	1
BP	GO:0045947	negative regulation of translational initiation	1/340	19/18614	0.29561 51	0.47792 37	0.36288 65	EIF4EBP1	1

BP	GO:0046782	regulation of viral transcription	1/340	19/18614	0.29561 51	0.47792 37	0.36288 65	ZFP36	1
BP	GO:0051152	positive regulation of smooth muscle cell differentiation	1/340	19/18614	0.29561 51	0.47792 37	0.36288 65	CTH	1
BP	GO:0051797	regulation of hair follicle development	1/340	19/18614	0.29561 51	0.47792 37	0.36288 65	TGFB2	1
BP	GO:0055062	phosphate ion homeostasis	1/340	19/18614	0.29561 51	0.47792 37	0.36288 65	ALPL	1
BP	GO:00	pericardium	1/340	19/18614	0.295	0.477	0.362	BMP5	1

	60039	development			6151	9237	8865		
BP	GO:0060716	labyrinthine layer blood vessel development	1/340	19/18614	0.2956151	0.4779237	0.3628865	JUNB	1
BP	GO:0060977	coronary vasculature morphogenesis	1/340	19/18614	0.2956151	0.4779237	0.3628865	ACE	1
BP	GO:0061615	glycolytic process through fructose-6-phosphate	1/340	19/18614	0.2956151	0.4779237	0.3628865	ENO1	1

BP	GO:0061744	motor behavior	1/340	19/18614	0.29561 51	0.47792 37	0.36288 65	DPP4	1
BP	GO:0071625	vocalization behavior	1/340	19/18614	0.29561 51	0.47792 37	0.36288 65	NRXN3	1
BP	GO:0071800	podosome assembly	1/340	19/18614	0.29561 51	0.47792 37	0.36288 65	SH3PXD2B	1
BP	GO:0072087	renal vesicle development	1/340	19/18614	0.29561 51	0.47792 37	0.36288 65	FMN1	1
BP	GO:0080182	histone H3-K4 trimethylation	1/340	19/18614	0.29561 51	0.47792 37	0.36288 65	NFKBIZ	1
BP	GO:0097202	activation of cysteine-type endope	1/340	19/18614	0.29561 51	0.47792 37	0.36288 65	NLRC4	1

		ptidase activity							
BP	GO:1901522	positive regulation of transcription from RNA polymerase II promoter involved in cellular response to chemical stimulus	1/340	19/18614	0.2956151	0.4779237	0.3628865	JUN	1
BP	GO:1902307	positive regulation of sodium	1/340	19/18614	0.2956151	0.4779237	0.3628865	FXYD1	1

		ion transmembrane transport							
BP	GO:1990822	basic amino acid transmembrane transport	1/340	19/18614	0.295 6151	0.477 9237	0.362 8865	SLC11A1	1
BP	GO:0032637	interleukin-8 production	3/340	104/18614	0.295 8336	0.477 9237	0.362 8865	IL1B/CD2/IL6	3
BP	GO:0060291	long-term synaptic potentiation	3/340	104/18614	0.295 8336	0.477 9237	0.362 8865	TNR/NTRK2/CX3CR1	3

BP	GO:0071868	cellular response to monoamine stimulus	3/340	104/18614	0.29583836	0.4779237	0.36288865	RYR2/HRH2/NR4A3	3
BP	GO:0071870	cellular response to catecholamine stimulus	3/340	104/18614	0.29583836	0.4779237	0.36288865	RYR2/HRH2/NR4A3	3
BP	GO:0051656	establishment of organelle localization	10/340	445/18614	0.2970152	0.47962892	0.36418814	SLC18A2/GEM/PTGDS/PIK3CG/ITGA4/HMOX1/FGR/NR4A3/RASGRP1/SYBU	10
BP	GO:0010821	regulation of mitochondrion	4/340	151/18614	0.2980222	0.48105120	0.36526612	HGF/IER3/DCN/TNFSF10	4

		organization							
BP	GO:0021545	cranial nerve development	2/340	60/18614	0.29984 93	0.48297 79	0.36672 41	EGR2/SLC1A3	2
BP	GO:0043407	negative regulation of MAP kinase activity	2/340	60/18614	0.29984 93	0.48297 79	0.36672 41	LAX1/IL1B	2
BP	GO:0055008	cardiac muscle tissue morphogenesis	2/340	60/18614	0.29984 93	0.48297 79	0.36672 41	RYR2/TGFB2	2
BP	GO:0060350	endochondral bone morphogenesis	2/340	60/18614	0.29984 93	0.48297 79	0.36672 41	CBS/ALPL	2
BP	GO:19	regulation of sprouti	2/340	60/18614	0.299	0.482	0.366	ADAMTS9/SEMA6A	2

	03670	ng angiogenesis			8493	9779	7241		
BP	GO:0051146	striated muscle cell differentiation	7/340	297/18614	0.3006228	0.4840193	0.3675148	NFATC2/ADAMTS15/CAPN3/KRT19/MYH11/CXCL10/CXCL9	7
BP	GO:0046425	regulation of receptor signaling pathway via JAK-STAT	3/340	105/18614	0.3008257	0.4841414	0.3676075	CCL5/IL6/SOCS3	3
BP	GO:0030832	regulation of actin filament length	4/340	152/18614	0.3021272	0.4858256	0.3688864	CXCL12/PLEKHH2/FMN1/CSF3	4
BP	GO:00	nephron	4/340	152/18614	0.302	0.485	0.368	EGR1/FMN1/GPR4/SULF1	4

	72006	development			1272	8256	8864		
BP	GO:0051056	regulation of small GTPase mediated signal transduction	7/340	298/18614	0.3035184	0.4870225	0.3697951	TGFB2/PIK3CG/TAGAP/FGD2/GPR4/P2RY10/RASGRP1	7
BP	GO:0002027	regulation of heart rate	3/340	106/18614	0.3058221	0.4870225	0.3697951	RYR2/ANK2/CASQ2	3
BP	GO:1901890	positive regulation of cell junction assembly	3/340	106/18614	0.3058221	0.4870225	0.3697951	FMN1/LRRN3/NTRK2	3
BP	GO:00	female gamete	4/340	153/18614	0.306	0.487	0.369	RPS6KA2/PTGS2/INHBA/PTX3	4

	07292	generation			2388	0225	7951		
BP	GO:0043393	regulation of protein binding	5/340	201/18614	0.3063109	0.4870225	0.3697951	TRIB3/ITGA4/ACE/CCL23/CSF3	5
BP	GO:0022029	telencephalon cell migration	2/340	61/18614	0.3066075	0.4870225	0.3697951	CXCL12/TNR	2
BP	GO:0035773	insulin secretion involved in cellular response to glucose stimulus	2/340	61/18614	0.3066075	0.4870225	0.3697951	ANO1/CFTR	2
BP	GO:0043903	regulation of biological	2/340	61/18614	0.3066075	0.4870225	0.3697951	CH25H/PTX3	2

		process involved in symbiotic interaction							
BP	GO:0045599	negative regulation of fat cell differentiation	2/340	61/18614	0.3066075	0.4870225	0.3697951	TRIB3/IL6	2
BP	GO:0046854	phosphatidylinositol phosphate biosynthetic process	2/340	61/18614	0.3066075	0.4870225	0.3697951	PIK3CG/SOCS3	2

BP	GO:0060042	retina morphogenesis in camera-type eye	2/340	61/18614	0.3066075	0.48702225	0.36979751	FAT3/NTRK2	2
BP	GO:0001502	cartilage condensation	1/340	20/18614	0.3084944	0.48702225	0.36979751	BMP1	1
BP	GO:0003215	cardiac right ventricle morphogenesis	1/340	20/18614	0.3084944	0.48702225	0.36979751	TGFB2	1
BP	GO:0006144	purine nucleobase metabolic process	1/340	20/18614	0.3084944	0.48702225	0.36979751	SHMT2	1

BP	GO:0006525	arginine metabolic process	1/340	20/18614	0.30849 44	0.48702 25	0.36979 51	FAH	1
BP	GO:0006662	glycerol ether metabolic process	1/340	20/18614	0.30849 44	0.48702 25	0.36979 51	FASN	1
BP	GO:0008340	determination of adult lifespan	1/340	20/18614	0.30849 44	0.48702 25	0.36979 51	TFCP2L1	1
BP	GO:0009110	vitamin biosynthetic process	1/340	20/18614	0.30849 44	0.48702 25	0.36979 51	PSAT1	1
BP	GO:0009168	purine ribonucleoside monophospha	1/340	20/18614	0.30849 44	0.48702 25	0.36979 51	AMPD1	1

		te biosynthetic process							
BP	GO:0009310	amine catabolic process	1/340	20/18614	0.3084944	0.4870225	0.3697951	MAOA	1
BP	GO:0010310	regulation of hydrogen peroxide metabolic process	1/340	20/18614	0.3084944	0.4870225	0.3697951	DUOXA1	1
BP	GO:0015810	aspartate transmembrane	1/340	20/18614	0.3084944	0.4870225	0.3697951	SLC1A3	1

		transport							
BP	GO:0030810	positive regulation of nucleotide biosynthetic process	1/340	20/18614	0.3084944	0.4870225	0.3697951	ENO1	1
BP	GO:0031998	regulation of fatty acid beta-oxidation	1/340	20/18614	0.3084944	0.4870225	0.3697951	CNR1	1
BP	GO:0035795	negative regulation of mitochondrial membrane	1/340	20/18614	0.3084944	0.4870225	0.3697951	IER3	1

		permeability							
BP	GO:0035902	response to immobilization stress	1/340	20/18614	0.3084944	0.4870225	0.36979751	FOS	1
BP	GO:0042053	regulation of dopamine metabolic process	1/340	20/18614	0.3084944	0.4870225	0.36979751	NR4A2	1
BP	GO:0042069	regulation of catecholamine metabolic process	1/340	20/18614	0.3084944	0.4870225	0.36979751	NR4A2	1

BP	GO:0042749	regulation of circadian sleep/wake cycle	1/340	20/18614	0.30849 44	0.48702 25	0.36979 51	PTGDS	1
BP	GO:0043691	reverse cholesterol transport	1/340	20/18614	0.30849 44	0.48702 25	0.36979 51	LIPG	1
BP	GO:0044794	positive regulation by host of viral process	1/340	20/18614	0.30849 44	0.48702 25	0.36979 51	ZDHHC9	1
BP	GO:0045342	MHC class II biosynthetic process	1/340	20/18614	0.30849 44	0.48702 25	0.36979 51	SLC11A1	1

BP	GO:0046485	ether lipid metabolic process	1/340	20/18614	0.30849944	0.4870225	0.3697951	FASN	1
BP	GO:0051546	keratinocyte migration	1/340	20/18614	0.30849944	0.4870225	0.3697951	HAS2	1
BP	GO:0070166	enamel mineralization	1/340	20/18614	0.30849944	0.4870225	0.3697951	CFTR	1
BP	GO:0097284	hepatocyte apoptotic process	1/340	20/18614	0.30849944	0.4870225	0.3697951	PIK3CG	1
BP	GO:0140131	positive regulation of lymphocyte	1/340	20/18614	0.30849944	0.4870225	0.3697951	CCL5	1

		chemotaxis							
BP	GO:1900227	positive regulation of NLRP3 inflammasome complex assembly	1/340	20/18614	0.30849 44	0.48702 25	0.36979 51	GBP5	1
BP	GO:1900373	positive regulation of purine nucleotide biosynthetic process	1/340	20/18614	0.30849 44	0.48702 25	0.36979 51	ENO1	1
BP	GO:19	negative regulati	1/340	20/18614	0.308	0.487	0.369	HYOU1	1

	02236	on of endoplasmic reticulum stress-induced intrinsic apoptotic signaling pathway			4944	0225	7951		
BP	GO:1903427	negative regulation of reactive oxygen species biosynthetic	1/340	20/18614	0.3084944	0.4870225	0.3697951	SLC18A2	1

		process							
BP	GO:2000269	regulation of fibroblast apoptotic process	1/340	20/18614	0.30849 44	0.48702 25	0.36979 51	PIK3CG	1
BP	GO:2001014	regulation of skeletal muscle cell differentiation	1/340	20/18614	0.30849 44	0.48702 25	0.36979 51	NR1D2	1
BP	GO:2001185	regulation of CD8-positive, alpha-beta T cell activation	1/340	20/18614	0.30849 44	0.48702 25	0.36979 51	IRF1	1

BP	GO:0031032	actomyosin structure organization	5/340	202/18614	0.30988803	0.48900078	0.37130026	SERPINF2/CAPN3/KRT19/MYH10/MYH11	5
BP	GO:2001257	regulation of cation channel activity	4/340	154/18614	0.31035563	0.48948801	0.37166612	KCNAB1/ANK2/GEM/CASQ2	4
BP	GO:0005976	polysaccharide metabolic process	3/340	107/18614	0.31082218	0.48948801	0.37166612	CHIT1/HAS2/MGAM	3
BP	GO:0071867	response to monoamine	3/340	107/18614	0.31082218	0.48948801	0.37166612	RYR2/HRH2/NR4A3	3
BP	GO:0071869	response to catech	3/340	107/18614	0.31082218	0.48948801	0.37166612	RYR2/HRH2/NR4A3	3

		olamine							
BP	GO:0090277	positive regulation of peptide hormone secretion	3/340	107/18614	0.3108218	0.4894801	0.3716612	ANO1/CFTR/FGA	3
BP	GO:0032233	positive regulation of actin filament bundle assembly	2/340	62/18614	0.3133531	0.4926521	0.3740697	SERPINF2/CX3CL1	2
BP	GO:0032481	positive regulation of type I interferon	2/340	62/18614	0.3133531	0.4926521	0.3740697	ZBTB20/IRF1	2

		production							
BP	GO:0043506	regulation of JUN kinase activity	2/340	62/18614	0.31335531	0.49265521	0.37406697	CBS/FGD2	2
BP	GO:1904036	negative regulation of epithelial cell apoptotic process	2/340	62/18614	0.31335531	0.49265521	0.37406697	HMOX1/FGA	2
BP	GO:0016125	sterol metabolic process	4/340	155/18614	0.31447591	0.49422186	0.37525591	EPHX2/DHCR7/CFTR/CH25H	4
BP	GO:0014013	regulation of gliogenesis	3/340	108/18614	0.31582839	0.49528295	0.37607723	EGR2/IL1B/IL6	3

BP	GO:0043266	regulation of potassium ion transport	3/340	108/18614	0.31582 39	0.4952895	0.3760723	KCNAB1/ANK2/CASQ2	3
BP	GO:0048568	embryonic organ development	10/340	453/18614	0.3158991	0.4952895	0.3760723	RYR2/FRZB/BMP5/TGFB2/PKDCC/APLNR/JUNB/RARRES2/KRT19/SOCS3	10
BP	GO:0051607	defense response to virus	7/340	303/18614	0.3180788	0.4952895	0.3760723	RNASE2/IL1B/DDIT4/IRF1/IL6/CXCL10/CXCL9	7
BP	GO:0001885	endothelial cell development	2/340	63/18614	0.3200844	0.4952895	0.3760723	FASN/IL1B	2
BP	GO:0048255	mRNA stabilization	2/340	63/18614	0.3200844	0.4952895	0.3760723	ZFP36/SLC11A1	2

BP	GO:0051965	positive regulation of synapse assembly	2/340	63/18614	0.32008 44	0.49528 95	0.37607 23	LRRN3/NTRK2	2
BP	GO:0070059	intrinsic apoptotic signaling pathway in response to endoplasmic reticulum stress	2/340	63/18614	0.32008 44	0.49528 95	0.37607 23	TRIB3/HYOU1	2
BP	GO:0032147	activation of protein kinase activity	3/340	109/18614	0.32082 77	0.49528 95	0.37607 23	TGFB2/CCL5/SLC11A1	3

BP	GO:0034766	negative regulation of monoatomic ion transmembrane transport	3/340	109/18614	0.320827 7	0.495289 5	0.376072 3	KCNAB1/GEM/CASQ2	3
BP	GO:0140546	defense response to symbiont	7/340	304/18614	0.321000 62	0.495289 5	0.376072 3	RNASE2/IL1B/DDIT4/IRF1/IL6/CXCL10/CXCL9	7
BP	GO:0002029	desensitization of G protein-coupled receptor	1/340	21/18614	0.321138 9	0.495289 5	0.376072 3	APLNR	1

		signaling pathway							
BP	GO:0002716	negative regulation of natural killer cell mediated immunity	1/340	21/18614	0.3211389	0.4952895	0.3760723	CD96	1
BP	GO:0006067	ethanol metabolic process	1/340	21/18614	0.3211389	0.4952895	0.3760723	SULT1B1	1
BP	GO:0007413	axonal fasciculation	1/340	21/18614	0.3211389	0.4952895	0.3760723	CNR1	1

BP	GO:0009083	branched-chain amino acid catabolic process	1/340	21/18614	0.32113 89	0.49528 95	0.37607 23	HMGCLL1	1
BP	GO:0010224	response to UV-B	1/340	21/18614	0.32113 89	0.49528 95	0.37607 23	MFAP4	1
BP	GO:0010288	response to lead ion	1/340	21/18614	0.32113 89	0.49528 95	0.37607 23	PTGS2	1
BP	GO:0010888	negative regulation of lipid storage	1/340	21/18614	0.32113 89	0.49528 95	0.37607 23	IL6	1
BP	GO:00	circadian sleep/w	1/340	21/18614	0.321	0.495	0.376	PTGDS	1

	22410	ake cycle process			1389	2895	0723			
BP	GO:0031290	retinal ganglion cell axon guidance	1/340	21/18614	0.3211389	0.4952895	0.3760723	ROBO2		1
BP	GO:0032305	positive regulation of icosanoid secretion	1/340	21/18614	0.3211389	0.4952895	0.3760723	IL1B		1
BP	GO:0032332	positive regulation of chondrocyte differentiation	1/340	21/18614	0.3211389	0.4952895	0.3760723	PKDCC		1

BP	GO:0035020	regulation of Rac protein signal transduction	1/340	21/18614	0.32113 89	0.49528 95	0.37607 23	PIK3CG	1
BP	GO:0035809	regulation of urine volume	1/340	21/18614	0.32113 89	0.49528 95	0.37607 23	HAS2	1
BP	GO:0036092	phosphatidylinositol-3-phosphate biosynthetic process	1/340	21/18614	0.32113 89	0.49528 95	0.37607 23	PIK3CG	1
BP	GO:0038083	peptidyl-tyrosine autoph	1/340	21/18614	0.32113 89	0.49528 95	0.37607 23	ACE	1

		osphorylation							
BP	GO:0043651	linoleic acid metabolic process	1/340	21/18614	0.32113 89	0.49528 95	0.37607 23	ALOX15B	1
BP	GO:0044321	response to leptin	1/340	21/18614	0.32113 89	0.49528 95	0.37607 23	NR4A3	1
BP	GO:0045063	T-helper 1 cell differentiation	1/340	21/18614	0.32113 89	0.49528 95	0.37607 23	NFKBIZ	1
BP	GO:0045821	positive regulation of glycolytic process	1/340	21/18614	0.32113 89	0.49528 95	0.37607 23	ZBTB20	1

BP	GO:0050765	negative regulation of phagocytosis	1/340	21/18614	0.32113 89	0.49528 95	0.37607 23	FCGR2B	1
BP	GO:0060353	regulation of cell adhesion molecule production	1/340	21/18614	0.32113 89	0.49528 95	0.37607 23	IL1B	1
BP	GO:0060445	branching involved in salivary gland morphogenesis	1/340	21/18614	0.32113 89	0.49528 95	0.37607 23	HGF	1
BP	GO:00	monocyte chemot	1/340	21/18614	0.321	0.495	0.376	IL1B	1

	71605	actic protein-1 production			1389	2895	0723			
BP	GO:0071637	regulation of monocyte chemotactic protein-1 production	1/340	21/18614	0.3211389	0.4952895	0.3760723	IL1B		1
BP	GO:0090141	positive regulation of mitochondrial fission	1/340	21/18614	0.3211389	0.4952895	0.3760723	DCN		1
BP	GO:0090201	negative regulation of release	1/340	21/18614	0.3211389	0.4952895	0.3760723	HGF		1

		of cytochrome c from mitochondria							
BP	GO:0097062	dendritic spine maintenance	1/340	21/18614	0.3211389	0.4952895	0.3760723	FCGR2B	1
BP	GO:0097503	sialylation	1/340	21/18614	0.3211389	0.4952895	0.3760723	ST6GALNAC3	1
BP	GO:0106030	neuron projection fasciculation	1/340	21/18614	0.3211389	0.4952895	0.3760723	CNR1	1
BP	GO:1902259	regulation of delayed rectifier potassium	1/340	21/18614	0.3211389	0.4952895	0.3760723	KCNAB1	1

		channel activity							
BP	GO:1903428	positive regulation of reactive oxygen species biosynthetic process	1/340	21/18614	0.3211389	0.4952895	0.3760723	DUOXA1	1
BP	GO:1903817	negative regulation of voltage-gated potassium channel activity	1/340	21/18614	0.3211389	0.4952895	0.3760723	KCNAB1	1

BP	GO:2000047	regulation of cell-cell adhesion mediated by cadherin	1/340	21/18614	0.32113389	0.49528995	0.37607723	SERPINF2	1
BP	GO:2000738	positive regulation of stem cell differentiation	1/340	21/18614	0.32113389	0.49528995	0.37607723	TGFB2	1
BP	GO:0007043	cell-cell junction assembly	4/340	157/18614	0.32273389	0.4973546	0.37764403	ANK2/APLNR/ACE/IL1B	4
BP	GO:0050905	neuromuscular process	4/340	157/18614	0.32273389	0.4973546	0.37764403	PCDH15/TNR/SLC1A3/NR4A1	4

BP	GO:0051701	biological process involved in interaction with host	5/340	206/18614	0.3242131	0.4994244	0.3792119	DPP4/SIGLEC1/ANPEP/CH25H/PTX3	5
BP	GO:0045445	myoblast differentiation	3/340	110/18614	0.3258321	0.5017156	0.3809516	CAPN3/CXCL10/CXCL9	3
BP	GO:0021885	forebrain cell migration	2/340	64/18614	0.3267996	0.5025962	0.38162202	CXCL12/TNR	2
BP	GO:0045453	bone resorption	2/340	64/18614	0.3267996	0.5025962	0.38162202	TNFRSF11B/IL6	2
BP	GO:0048278	vesicle docking	2/340	64/18614	0.3267996	0.5025962	0.38162202	SYTL2/CFTR	2

BP	GO:0042594	response to starvation	5/340	207/18614	0.32780 83	0.503944	0.38264 36	PLIN2/ZFP36/AKR1C3/ATF3/FOS	5
BP	GO:2000060	positive regulation of ubiquitin-dependent protein catabolic process	3/340	111/18614	0.33083 64	0.50624 06	0.38438 74	TRIB3/PYHIN1/TRIB1	3
BP	GO:0045927	positive regulation of growth	6/340	258/18614	0.33290 28	0.50624 06	0.38438 74	CXCL12/TGFB2/CAPN3/SFN/S100A8/TFCP2L1	6
BP	GO:0010972	negative regulation of G2/M transiti	2/340	65/18614	0.33349 71	0.50624 06	0.38438 74	IER3/DTL	2

		on of mitotic cell cycle							
BP	GO:0019226	transmission of nerve impulse	2/340	65/18614	0.333497	0.5062406	0.3843874	TNR/NTRK2	2
BP	GO:0030888	regulation of B cell proliferation	2/340	65/18614	0.333497	0.5062406	0.3843874	NFATC2/FCGR2B	2
BP	GO:0046888	negative regulation of hormone secretion	2/340	65/18614	0.333497	0.5062406	0.3843874	IL1B/INHBA	2
BP	GO:0050435	amyloid-beta metabolic	2/340	65/18614	0.333497	0.5062406	0.3843874	ACE/NTRK2	2

		process							
BP	GO:0000470	maturation of LSU-rRNA	1/340	22/18614	0.3335529	0.5062406	0.3843874	WDR12	1
BP	GO:0001919	regulation of receptor recycling	1/340	22/18614	0.3335529	0.5062406	0.3843874	TRAT1	1
BP	GO:0002115	store-operated calcium entry	1/340	22/18614	0.3335529	0.5062406	0.3843874	MS4A1	1
BP	GO:0003085	negative regulation of systemic arterial blood	1/340	22/18614	0.3335529	0.5062406	0.3843874	IER3	1

		pressure							
BP	GO:0009127	purine nucleoside monophosphate biosynthetic process	1/340	22/18614	0.33355529	0.50624 06	0.38438874	AMPD1	1
BP	GO:0010042	response to manganese ion	1/340	22/18614	0.33355529	0.50624 06	0.38438874	PTGS2	1
BP	GO:0015669	gas transport	1/340	22/18614	0.33355529	0.50624 06	0.38438874	HBB	1
BP	GO:0015802	basic amino acid transport	1/340	22/18614	0.33355529	0.50624 06	0.38438874	SLC11A1	1

BP	GO:0015874	norepinephrine transport	1/340	22/18614	0.33355529	0.50624060	0.38438874	P2RY1	1
BP	GO:0022401	negative adaptation of signaling pathway	1/340	22/18614	0.33355529	0.50624060	0.38438874	APLNR	1
BP	GO:0023019	signal transduction involved in regulation of gene expression	1/340	22/18614	0.33355529	0.50624060	0.38438874	P2RY1	1
BP	GO:0030277	maintenance of gastroi	1/340	22/18614	0.33355529	0.50624060	0.38438874	SERPINA3	1

		ntestinal epithelium							
BP	GO:0032303	regulation of icosanoid secretion	1/340	22/18614	0.3335529	0.5062406	0.38438 74	IL1B	1
BP	GO:0035739	CD4-positive, alpha-beta T cell proliferation	1/340	22/18614	0.3335529	0.5062406	0.38438 74	CD3E	1
BP	GO:0045624	positive regulation of T-helper cell differentiation	1/340	22/18614	0.3335529	0.5062406	0.38438 74	NFKBIZ	1

BP	GO:0051151	negative regulation of smooth muscle cell differentiation	1/340	22/18614	0.33355529	0.50624060	0.38438874	NFATC2	1
BP	GO:0051767	nitric-oxide synthase biosynthetic process	1/340	22/18614	0.33355529	0.50624060	0.38438874	CCL2	1
BP	GO:0051769	regulation of nitric-oxide synthase biosynthetic	1/340	22/18614	0.33355529	0.50624060	0.38438874	CCL2	1

		process							
BP	GO:0072111	cell proliferation involved in kidney development	1/340	22/18614	0.33355529	0.5062406	0.38438874	EGR1	1
BP	GO:0086012	membrane depolarization during cardiac muscle cell action potential	1/340	22/18614	0.33355529	0.5062406	0.38438874	ANK2	1
BP	GO:1900424	regulation of defense respon	1/340	22/18614	0.33355529	0.5062406	0.38438874	PGC	1

		se to bacterium							
BP	GO:1901386	negative regulation of voltage-gated calcium channel activity	1/340	22/18614	0.33355529	0.5062406	0.38438874	GEM	1
BP	GO:2000310	regulation of NMDA receptor activity	1/340	22/18614	0.33355529	0.5062406	0.38438874	CCL2	1
BP	GO:2000561	regulation of CD4-positive, alpha-beta T cell	1/340	22/18614	0.33355529	0.5062406	0.38438874	CD3E	1

		proliferation							
BP	GO:2001044	regulation of integrin-mediated signaling pathway	1/340	22/18614	0.33355529	0.5062406	0.38438874	CD177	1
BP	GO:0045732	positive regulation of protein catabolic process	5/340	209/18614	0.35010107	0.50825071	0.38591379	TRIB3/IER3/IL1B/DTL/TRIB1	5
BP	GO:0035051	cardiocyte differentiation	4/340	161/18614	0.3393	0.51455333	0.39069992	TGFB2/VCAM1/INHBA/MYH11	4

BP	GO:0010517	regulation of phospholipase activity	2/340	66/18614	0.340171753	0.515675555	0.391555513	CCL5/SELE	2
BP	GO:0002062	chondrocyte differentiation	3/340	113/18614	0.340848411	0.516272741	0.392000058	PKDCC/SLC39A14/SULF1	3
BP	GO:0006939	smooth muscle contraction	3/340	113/18614	0.340848411	0.516272741	0.392000058	PTGS2/SULF1/MYH11	3
BP	GO:0032869	cellular response to insulin stimulus	5/340	211/18614	0.342222669	0.517179614	0.39328287	TRIB3/SLC39A14/RARRES2/IL1B/SOCS3	5
BP	GO:0060541	respiratory system development	5/340	211/18614	0.342222669	0.517179614	0.39328287	PKDCC/SPEF2/ACE/CRISPLD2/SLC7A11	5

BP	GO:0002070	epithelial cell maturation	1/340	23/18614	0.345745405	0.518080802	0.393377272	TFCP2L1	1
BP	GO:0003091	renal water homeostasis	1/340	23/18614	0.345745405	0.518080802	0.393377272	HAS2	1
BP	GO:0016338	calcium-independent cell-cell adhesion via plasma membrane cell-adhesion molecules	1/340	23/18614	0.345745405	0.518080802	0.393377272	CX3CL1	1
BP	GO:00	removal of supero	1/340	23/18614	0.345	0.518	0.393	NQO1	1

	19430	xide radicals			7405	0802	3772			
BP	GO:0035268	protein mannosylation	1/340	23/18614	0.3457405	0.5180802	0.3933772	TMTC1		1
BP	GO:0035988	chondrocyte proliferation	1/340	23/18614	0.3457405	0.5180802	0.3933772	DDR2		1
BP	GO:0036315	cellular response to sterol	1/340	23/18614	0.3457405	0.5180802	0.3933772	INHBA		1
BP	GO:0038095	Fc-epsilon receptor signaling pathway	1/340	23/18614	0.3457405	0.5180802	0.3933772	NR4A3		1
BP	GO:0042219	cellular modified amino	1/340	23/18614	0.3457405	0.5180802	0.3933772	CBS		1

		acid catabolic process								
BP	GO:0042745	circadian sleep/wake cycle	1/340	23/18614	0.3457405	0.5180802	0.39933772	PTGDS		1
BP	GO:0044827	modulation by host of viral genome replication	1/340	23/18614	0.3457405	0.5180802	0.39933772	CCL8		1
BP	GO:0045591	positive regulation of regulatory T cell differentiation	1/340	23/18614	0.3457405	0.5180802	0.39933772	IL2RG		1

BP	GO:0045717	negative regulation of fatty acid biosynthetic process	1/340	23/18614	0.345457405	0.518080802	0.393933772	TRIB3	1
BP	GO:0046058	cAMP metabolic process	1/340	23/18614	0.345457405	0.518080802	0.393933772	PDE7B	1
BP	GO:0046716	muscle cell cellular homeostasis	1/340	23/18614	0.345457405	0.518080802	0.393933772	CAPN3	1
BP	GO:0060065	uterus development	1/340	23/18614	0.345457405	0.518080802	0.393933772	TGFB2	1

BP	GO:0072574	hepatocyte proliferation	1/340	23/18614	0.3457405	0.5180802	0.3933772	IL6	1
BP	GO:0072575	epithelial cell proliferation involved in liver morphogenesis	1/340	23/18614	0.3457405	0.5180802	0.3933772	IL6	1
BP	GO:0097164	ammonium ion metabolic process	1/340	23/18614	0.3457405	0.5180802	0.3933772	HDC	1
BP	GO:1901984	negative regulation of protein acetylation	1/340	23/18614	0.3457405	0.5180802	0.3933772	SDR16C5	1

BP	GO:1905709	negative regulation of membrane permeability	1/340	23/18614	0.34574405	0.51808802	0.39337772	IER3	1
BP	GO:2001169	regulation of ATP biosynthetic process	1/340	23/18614	0.34574405	0.51808802	0.39337772	ENO1	1
BP	GO:0098739	import across plasma membrane	5/340	212/18614	0.34583894	0.51808802	0.39337772	SCNN1G/MS4A1/SLC39A14/SLC7A11/SLC1A3	5
BP	GO:0031532	actin cytoskeleton reorganization	3/340	114/18614	0.34584	0.51808802	0.39337772	HMCN1/STAP1/CSF3	3

BP	GO:0032410	negative regulation of transporter activity	3/340	114/18614	0.34584	0.5180802	0.3933772	KCNAB1/GEM/CASQ2	3
BP	GO:0051963	regulation of synapse assembly	3/340	114/18614	0.34584	0.5180802	0.3933772	ROBO2/LRRN3/NTRK2	3
BP	GO:0042531	positive regulation of tyrosine phosphorylation of STAT protein	2/340	67/18614	0.3468327	0.5185483	0.3937326	CCL5/IL6	2
BP	GO:00	sensory percept	2/340	67/18614	0.346	0.518	0.393	SCNN1G/AZGP1	2

	50909	ion of taste			0.34683 27	0.51854 83	0.39373 26		
BP	GO:0060193	positive regulation of lipase activity	2/340	67/18614	0.34683 27	0.51854 83	0.39373 26	CCL5/SELE	2
BP	GO:0071806	protein transmembrane transport	2/340	67/18614	0.34683 27	0.51854 83	0.39373 26	HYOU1/AZGP1	2
BP	GO:1902750	negative regulation of cell cycle G2/M phase transition	2/340	67/18614	0.34683 27	0.51854 83	0.39373 26	IER3/DTL	2
BP	GO:00	synapse	10/340	466/18614	0.347	0.518	0.393	ROBO2/TNR/SLC7A11/LRRN3/FCGR2B/TNC/SYBU/NTRK2/CX3CR1/CX3CL1	10

	50808	organization			1237	7798	9084		
BP	GO:0042303	molting cycle	3/340	115/18614	0.350838354	0.5239196	0.397808083	PTGS2/TGFB2/INHBA	3
BP	GO:0042633	hair cycle	3/340	115/18614	0.350838354	0.5239196	0.397808083	PTGS2/TGFB2/INHBA	3
BP	GO:0030307	positive regulation of cell growth	4/340	164/18614	0.351517415	0.5250634	0.398676795	CXCL12/TGFB2/SFN/S100A8	4
BP	GO:0016051	carbohydrate biosynthetic process	5/340	214/18614	0.353530712	0.5267454	0.399995567	P2RY1/FUT3/SLC39A14/ATF3/HAS2	5
BP	GO:0000288	nuclear-transcribed mRNA	2/340	68/18614	0.353464678	0.5267454	0.399995567	BTG2/ZFP36	2

		catabolic process, deadenylation-dependent decay							
BP	GO:0045670	regulation of osteoclast differentiation	2/340	68/18614	0.35346678	0.52667454	0.39995567	TNFRSF11B/FOS	2
BP	GO:0048645	animal organ formation	2/340	68/18614	0.35346678	0.52667454	0.39995567	ROBO2/SULF1	2
BP	GO:0034440	lipid oxidation	3/340	116/18614	0.35582666	0.52667454	0.39995567	CNR1/NR4A3/ALOX15B	3
BP	GO:19	regulation of recepto	3/340	116/18614	0.355	0.526	0.399	CCL5/IL6/SOCS3	3

	04892	r signaling pathway via STAT			8266	7454	9567		
BP	GO:0050792	regulation of viral process	4/340	165/18614	0.35589	0.5267454	0.3999567	CCL5/ZFP36/CH25H/PTX3	4
BP	GO:0001759	organ induction	1/340	24/18614	0.3577059	0.5267454	0.3999567	ROBO2	1
BP	GO:0002082	regulation of oxidative phosphorylation	1/340	24/18614	0.3577059	0.5267454	0.3999567	SHMT2	1
BP	GO:0003323	type B pancreatic cell	1/340	24/18614	0.3577059	0.5267454	0.3999567	BMP5	1

BP	GO:0008045	development motor neuron axon guidance	1/340	24/18614	0.3577059	0.5267454	0.39995567	EGR2		1
BP	GO:0010839	negative regulation of keratinocyte proliferation	1/340	24/18614	0.3577059	0.5267454	0.39995567	SFN		1
BP	GO:0010842	retina layer formation	1/340	24/18614	0.3577059	0.5267454	0.39995567	FAT3		1
BP	GO:0018230	peptidyl-L-cysteine S-palmitoylation	1/340	24/18614	0.3577059	0.52674554	0.39995567	ZDHHC9		1

BP	GO:0018231	peptidyl-S-diacylglycerol-L-cysteine biosynthetic process from peptidyl-cysteine	1/340	24/18614	0.35770 59	0.52674 54	0.39995 67	ZDHHC9	1
BP	GO:0023058	adaptation of signaling pathway	1/340	24/18614	0.35770 59	0.52674 54	0.39995 67	APLNR	1
BP	GO:0030539	male genitalia development	1/340	24/18614	0.35770 59	0.52674 54	0.39995 67	BMP5	1

BP	GO:0032528	microvillus organization	1/340	24/18614	0.3577059	0.5267454	0.3999567	GLDN	1
BP	GO:0035357	peroxisome proliferator activated receptor signaling pathway	1/340	24/18614	0.3577059	0.5267454	0.3999567	ALOX15B	1
BP	GO:0036120	cellular response to platelet-derived growth factor stimulus	1/340	24/18614	0.3577059	0.5267454	0.3999567	HAS2	1

BP	GO:0036158	outer dynein arm assembly	1/340	24/18614	0.357 7059	0.526 7454	0.399 9567	DNAH5	1
BP	GO:0040037	negative regulation of fibroblast growth factor receptor signaling pathway	1/340	24/18614	0.357 7059	0.526 7454	0.399 9567	SULF1	1
BP	GO:0042430	indole-containing compound metabolic	1/340	24/18614	0.357 7059	0.526 7454	0.399 9567	IDO1	1

		process							
BP	GO:0043576	regulation of respiratory gaseous exchange	1/340	24/18614	0.35770 59	0.52674 54	0.39995 67	NR4A2	1
BP	GO:0045540	regulation of cholesterol biosynthetic process	1/340	24/18614	0.35770 59	0.52674 54	0.39995 67	DHCR7	1
BP	GO:0048011	neurotrophin TRK receptor signaling	1/340	24/18614	0.35770 59	0.52674 54	0.39995 67	DDIT4	1

		pathway							
BP	GO:0051923	sulfation	1/340	24/18614	0.3577059	0.5267454	0.3999567	SULT1B1	1
BP	GO:0060307	regulation of ventricular cardiac muscle cell membrane repolarization	1/340	24/18614	0.3577059	0.5267454	0.3999567	ANK2	1
BP	GO:0060352	cell adhesion molecule production	1/340	24/18614	0.3577059	0.5267454	0.3999567	IL1B	1

BP	GO:0072576	liver morphogenesis	1/340	24/18614	0.3577059	0.5267454	0.3999567	IL6	1
BP	GO:0090103	cochlea morphogenesis	1/340	24/18614	0.3577059	0.5267454	0.3999567	FRZB	1
BP	GO:0090335	regulation of brown fat cell differentiation	1/340	24/18614	0.3577059	0.5267454	0.3999567	PTGS2	1
BP	GO:0098743	cell aggregation	1/340	24/18614	0.3577059	0.5267454	0.3999567	BMP1	1
BP	GO:0106118	regulation of sterol biosynthetic process	1/340	24/18614	0.3577059	0.5267454	0.3999567	DHCR7	1

BP	GO:1903077	negative regulation of protein localization to plasma membrane	1/340	24/18614	0.35770 59	0.52674 54	0.39995 67	PKDCC	1
BP	GO:0010948	negative regulation of cell cycle process	7/340	317/18614	0.35941 39	0.52905 62	0.40171 12	RPS6KA2/WEE1/PPP1R10/IER3/DTL/INHBA/CCL2	7
BP	GO:0030858	positive regulation of epithelial cell differentiation	2/340	69/18614	0.36007 92	0.52983 09	0.40229 95	SFN/ALOX15B	2

BP	GO:0006469	negative regulation of protein kinase activity	5/340	217/18614	0.36393916	0.5351523	0.40634	TRIB3/LAX1/IL1B/SFN/TRIB1	5
BP	GO:0110053	regulation of actin filament organization	6/340	268/18614	0.36532377	0.5351523	0.40634	CXCL12/SERPINF2/PLEKHH2/FMN1/CX3CL1/CSF3	6
BP	GO:0006790	sulfur compound metabolic process	7/340	319/18614	0.3653666	0.5351523	0.40634	GCLM/CBS/PHGDH/SLC7A11/DCN/SULT1B1/CTH	7
BP	GO:0030193	regulation of blood coagulation	2/340	70/18614	0.36666656	0.5351523	0.40634	SERPINF2/FGA	2

BP	GO:0034394	protein localization to cell surface	2/340	70/18614	0.366665 6	0.535152 3	0.406634	ANK2/CD177	2
BP	GO:0043687	post-translational protein modification	2/340	70/18614	0.366665 6	0.535152 3	0.406634	AGBL1/EGR1	2
BP	GO:0046847	filopodium assembly	2/340	70/18614	0.366665 6	0.535152 3	0.406634	DPYSL3/FGD2	2
BP	GO:0007156	homophilic cell adhesion via plasma membrane adhesion	4/340	168/18614	0.368332 8	0.535152 3	0.406634	PCDH15/ROBO2/HMCN1/FAT3	4

BP	GO:0000466	molecules maturation of 5.8S rRNA from tricistronic rRNA transcript (SSU-rRNA, 5.8S rRNA, LSU-rRNA)	1/340	25/18614	0.3694531	0.5351523	0.40634	WDR12	1
BP	GO:0001779	natural killer cell differentiation	1/340	25/18614	0.3694531	0.5351523	0.40634	RASGRP1	1
BP	GO:00	renal system proces	1/340	25/18614	0.369	0.535	0.40634	SERPINF2	1

	03071	s involved in regulation of systemic arterial blood pressure			4531	1523			
BP	GO:0006007	glucose catabolic process	1/340	25/18614	0.3694531	0.5351523	0.40634	ENO1	1
BP	GO:0008211	glucocorticoid metabolic process	1/340	25/18614	0.3694531	0.5351523	0.40634	BMP5	1
BP	GO:0010226	response to lithium ion	1/340	25/18614	0.3694531	0.5351523	0.40634	PTGS2	1

BP	GO:0010623	programmed cell death involved in cell development	1/340	25/18614	0.36945 31	0.53515 23	0.40634	IL1B	1
BP	GO:0014047	glutamate secretion	1/340	25/18614	0.36945 31	0.53515 23	0.40634	NTRK2	1
BP	GO:0019985	translesion synthesis	1/340	25/18614	0.36945 31	0.53515 23	0.40634	DTL	1
BP	GO:0030318	melanocyte differentiation	1/340	25/18614	0.36945 31	0.53515 23	0.40634	ADAMTS9	1
BP	GO:0031579	membrane raft organization	1/340	25/18614	0.36945 31	0.53515 23	0.40634	CD2	1

BP	GO:0043501	skeletal muscle adaptation	1/340	25/18614	0.3694531	0.5351523	0.40634	GTF2IRD1	1
BP	GO:0043567	regulation of insulin-like growth factor receptor signaling pathway	1/340	25/18614	0.3694531	0.5351523	0.40634	BMP5	1
BP	GO:0045662	negative regulation of myoblast differentiation	1/340	25/18614	0.3694531	0.5351523	0.40634	CXCL10	1

BP	GO:0046033	AMP metabolic process	1/340	25/18614	0.369454531	0.535151523	0.40634	AMPD1	1
BP	GO:0046641	positive regulation of alpha-beta T cell proliferation	1/340	25/18614	0.369454531	0.535151523	0.40634	CD3E	1
BP	GO:0050927	positive regulation of positive chemotaxis	1/340	25/18614	0.369454531	0.535151523	0.40634	CXCL12	1
BP	GO:0060333	type II interferon-mediated signaling	1/340	25/18614	0.369454531	0.535151523	0.40634	IRF1	1

		pathway							
BP	GO:0060706	cell differentiation involved in embryonic placenta development	1/340	25/18614	0.36945431	0.53515523	0.40634	KRT19	1
BP	GO:0070129	regulation of mitochondrial translation	1/340	25/18614	0.36945431	0.53515523	0.40634	SHMT2	1
BP	GO:0070935	3'-UTR-mediated mRNA stabilization	1/340	25/18614	0.36945431	0.53515523	0.40634	ZFP36	1

BP	GO:0071450	cellular response to oxygen radical	1/340	25/18614	0.36945 31	0.53515 23	0.40634	NQO1	1
BP	GO:0071451	cellular response to superoxide	1/340	25/18614	0.36945 31	0.53515 23	0.40634	NQO1	1
BP	GO:0072273	metanephric nephron morphogenesis	1/340	25/18614	0.36945 31	0.53515 23	0.40634	FMN1	1
BP	GO:0086010	membrane depolarization during action potential	1/340	25/18614	0.36945 31	0.53515 23	0.40634	ANK2	1

BP	GO:0086064	cell communication by electrical coupling involved in cardiac conduction	1/340	25/18614	0.36945 31	0.53515 23	0.40634	RYR2	1
BP	GO:0097186	amelogenesis	1/340	25/18614	0.36945 31	0.53515 23	0.40634	CFTR	1
BP	GO:0098719	sodium ion import across plasma membrane	1/340	25/18614	0.36945 31	0.53515 23	0.40634	SCNN1G	1

BP	GO:1901071	glucosamine-containing compound metabolic process	1/340	25/18614	0.3694531	0.5351523	0.40634	CHIT1		1
BP	GO:1905563	negative regulation of vascular endothelial cell proliferation	1/340	25/18614	0.3694531	0.5351523	0.40634	CCL2		1
BP	GO:2000001	regulation of DNA damage	1/340	25/18614	0.3694531	0.5351523	0.40634	PPP1R10		1

BP	GO:2000193	positive regulation of fatty acid transport	1/340	25/18614	0.36945 31	0.53515 23	0.40634	IL1B	1
BP	GO:2000209	regulation of anoikis	1/340	25/18614	0.36945 31	0.53515 23	0.40634	NTRK2	1
BP	GO:0006720	isoprenoid metabolic process	3/340	119/18614	0.37076 75	0.53664 83	0.40747 59	SDR16C5/RARRES2/AKR1C3	3
BP	GO:0030278	regulation of ossification	3/340	119/18614	0.37076 75	0.53664 83	0.40747 59	PKDCC/EGR2/DDR2	3
BP	GO:00	embryonic heart	2/340	71/18614	0.373	0.540	0.410	RYR2/APLNR	2

	03143	tube morphogenesis			2256	0011	0217		
BP	GO:0032355	response to estradiol	3/340	120/18614	0.37573345	0.54278804	0.41213132	PTGS2/NQO1/SLC6A4	3
BP	GO:0043200	response to amino acid	3/340	120/18614	0.37573345	0.54278804	0.41213132	GCLM/NQO1/NTRK2	3
BP	GO:0051101	regulation of DNA binding	3/340	120/18614	0.37573345	0.54278804	0.41213132	JUN/HMOX1/PYHIN1	3
BP	GO:0009267	cellular response to starvation	4/340	170/18614	0.37662612	0.54278804	0.41213132	PLIN2/AKR1C3/ATF3/FOS	4
BP	GO:0009416	response to light stimulus	7/340	323/18614	0.37729909	0.54278804	0.41213132	PTGS2/MFAP4/JUND/SLC7A11/SLC1A3/DTL/FOS	7

BP	GO:0006644	phospholipid metabolic process	8/340	375/18614	0.3784254	0.5427804	0.4121332	PIGW/IDH1/PIK3CG/ABCA8/LIPG/ALOX15B/PLA2G2A/SOCS3	8
BP	GO:0008544	epidermis development	8/340	375/18614	0.3784254	0.5427804	0.4121332	KRT7/TGFB2/NFKBIZ/ZFP36/AKR1C3/SFN/INHBA/ALOX15B	8
BP	GO:0006024	glycosaminoglycan biosynthetic process	2/340	72/18614	0.3797581	0.5427804	0.4121332	IL1B/HAS2	2
BP	GO:0043489	RNA stabilization	2/340	72/18614	0.3797581	0.5427804	0.4121332	ZFP36/SLC11A1	2
BP	GO:0043954	cellular component maintenance	2/340	72/18614	0.3797581	0.5427804	0.4121332	CD177/FCGR2B	2

BP	GO:0046626	regulation of insulin receptor signaling pathway	2/340	72/18614	0.37975581	0.54278804	0.412132	IL1B/SOCS3	2
BP	GO:1900046	regulation of hemostasis	2/340	72/18614	0.37975581	0.54278804	0.412132	SERPINF2/FGA	2
BP	GO:1904888	cranial skeletal system development	2/340	72/18614	0.37975581	0.54278804	0.412132	FREM1/TGFB2	2
BP	GO:1901989	positive regulation of cell cycle phase transition	3/340	121/18614	0.38069336	0.54278804	0.412132	PPP1R10/DDR2/DTL	3

BP	GO:0010950	positive regulation of endopeptidase activity	4/340	171/18614	0.380762	0.5427804	0.4121132	NLRC4/UACA/S100A8/TNFSF10	4
BP	GO:1902905	positive regulation of supramolecular fiber organization	4/340	171/18614	0.380762	0.5427804	0.4121132	SERPINF2/FMN1/CX3CL1/CSF3	4
BP	GO:0002026	regulation of the force of heart contraction	1/340	26/18614	0.380986	0.5427804	0.4121132	RYR2	1
BP	GO:0006541	glutamine metabolic	1/340	26/18614	0.380986	0.5427804	0.4121132	PHGDH	1

		process							
BP	GO:0007274	neuromuscular synaptic transmission	1/340	26/18614	0.380986	0.5427804	0.4121132	EGR3	1
BP	GO:0009435	NAD biosynthetic process	1/340	26/18614	0.380986	0.5427804	0.4121132	IDO1	1
BP	GO:0010460	positive regulation of heart rate	1/340	26/18614	0.380986	0.5427804	0.4121132	RYR2	1
BP	GO:0022010	central nervous system myelination	1/340	26/18614	0.380986	0.5427804	0.4121132	CNTN1	1

BP	GO:0032291	axon ensheathment in central nervous system	1/340	26/18614	0.380986	0.5427804	0.4121132	CNTN1	1
BP	GO:0032816	positive regulation of natural killer cell activation	1/340	26/18614	0.380986	0.5427804	0.4121132	RASGRP1	1
BP	GO:0036119	response to platelet-derived growth factor	1/340	26/18614	0.380986	0.5427804	0.4121132	HAS2	1
BP	GO:00	fibroblast apoptot	1/340	26/18614	0.380	0.542	0.412	PIK3CG	1

	44346	ic process			986	7804	132			
BP	GO:0045672	positive regulation of osteoclast differentiation	1/340	26/18614	0.380986	0.5427804	0.412132	FOS		1
BP	GO:0045822	negative regulation of heart contraction	1/340	26/18614	0.380986	0.5427804	0.412132	PIK3CG		1
BP	GO:0046426	negative regulation of receptor signaling pathwa	1/340	26/18614	0.380986	0.5427804	0.412132	SOCS3		1

		y via JAK-STAT							
BP	GO:0050926	regulation of positive chemotaxis	1/340	26/18614	0.380986	0.5427804	0.4121312	CXCL12	1
BP	GO:0051894	positive regulation of focal adhesion assembly	1/340	26/18614	0.380986	0.5427804	0.4121312	FMN1	1
BP	GO:0060314	regulation of ryanodine-sensitive calcium-release channe	1/340	26/18614	0.380986	0.5427804	0.4121312	CASQ2	1

		l activity							
BP	GO:0060561	apoptotic process involved in morphogenesis	1/340	26/18614	0.380986	0.5427804	0.412132	TGFB2	1
BP	GO:0062009	secondary palate development	1/340	26/18614	0.380986	0.5427804	0.412132	TGFB2	1
BP	GO:0070102	interleukin-6-mediated signaling pathway	1/340	26/18614	0.380986	0.5427804	0.412132	IL6	1
BP	GO:00	zinc ion transmembran	1/340	26/18614	0.380	0.542	0.412	SLC39A14	1

	71577	e transport			986	7804	132		
BP	GO:0140374	antiviral innate immune response	1/340	26/18614	0.380986	0.5427804	0.412132	CXCL10	1
BP	GO:1900151	regulation of nuclear-transcribed mRNA catabolic process, deadenylation-dependent decay	1/340	26/18614	0.380986	0.5427804	0.412132	ZFP36	1

BP	GO:1901028	regulation of mitochondrial outer membrane permeabilization involved in apoptotic signaling pathway	1/340	26/18614	0.380986	0.5427804	0.4121132	IER3	1
BP	GO:1904376	negative regulation of protein localization to cell	1/340	26/18614	0.380986	0.5427804	0.4121132	PKDCC	1

BP	GO:2000647	periphery negative regulation of stem cell proliferation	1/340	26/18614	0.380986	0.54278 04	0.412132	SFN	1
BP	GO:0044242	cellular lipid catabolic process	5/340	222/18614	0.38204 47	0.54408 53	0.41312 28	IDH1/PIK3CG/CNR1/AKR1C3/LIPG	5
BP	GO:1901988	negative regulation of cell cycle phase transition	6/340	274/18614	0.38486 98	0.54774 23	0.41589 96	WEE1/PPP1R10/IER3/DTL/INHBA/CCL2	6

BP	GO:0055088	lipid homeostasis	4/340	172/18614	0.38849	0.5477423	0.4158996	ZBTB20/EPHX2/LIPG/NR1D2	4
BP	GO:0045109	intermediate filament organization	2/340	73/18614	0.3862618	0.5488604	0.4167485	KRT7/KRT19	2
BP	GO:0060415	muscle tissue morphogenesis	2/340	73/18614	0.3862618	0.5488604	0.4167485	RYR2/TGFB2	2
BP	GO:1900076	regulation of cellular response to insulin stimulus	2/340	73/18614	0.3862618	0.5488604	0.4167485	IL1B/SOCS3	2
BP	GO:1902373	negative regulation of	2/340	73/18614	0.3862618	0.5488604	0.4167485	ZFP36/SLC11A1	2

		mRNA catabolic process							
BP	GO:0018209	peptidyl-serine modification	7/340	327/18614	0.38923338	0.55066679	0.41812121	HGF/PTGS2/DCN/DDIT4/IL6/NTRK2/CSF3	7
BP	GO:0000077	DNA damage checkpoint signaling	3/340	123/18614	0.39058855	0.55066679	0.41812121	PPP1R10/IER3/DTL	3
BP	GO:0001573	ganglioside metabolic process	1/340	27/18614	0.39230086	0.55066679	0.41812121	ST6GALNAC3	1
BP	GO:0001783	B cell apoptotic	1/340	27/18614	0.39230086	0.55066679	0.41812121	NFKBIZ	1

		process								
BP	GO:0002092	positive regulation of receptor internalization	1/340	27/18614	0.39230086	0.55066679	0.4181211	SELE		1
BP	GO:0002710	negative regulation of T cell mediated immunity	1/340	27/18614	0.39230086	0.55066679	0.4181211	FCGR2B		1
BP	GO:0006614	SRP-dependent cotranslational protein targeting to	1/340	27/18614	0.39230086	0.55066679	0.4181211	SRPRB		1

BP	GO:0009065	membrane glutamine family amino acid catabolic process	1/340	27/18614	0.3923086	0.55066679	0.41812121	FAH	1
BP	GO:0009651	response to salt stress	1/340	27/18614	0.3923086	0.55066679	0.41812121	CAPN3	1
BP	GO:0010804	negative regulation of tumor necrosis factor-mediated signalin	1/340	27/18614	0.3923086	0.55066679	0.41812121	NAIP	1

		g pathway							
BP	GO:0010875	positive regulation of cholesterol efflux	1/340	27/18614	0.39230086	0.55066679	0.4181211	ABCA8	1
BP	GO:0010884	positive regulation of lipid storage	1/340	27/18614	0.39230086	0.55066679	0.4181211	PLIN2	1
BP	GO:0019433	triglyceride catabolic process	1/340	27/18614	0.39230086	0.55066679	0.4181211	PIK3CG	1
BP	GO:0031954	positive regulation of protein autoph	1/340	27/18614	0.39230086	0.55066679	0.4181211	ACE	1

		osphorylation							
BP	GO:0032682	negative regulation of chemokine production	1/340	27/18614	0.3923086	0.55066679	0.41812121	IL6	1
BP	GO:0032753	positive regulation of interleukin-4 production	1/340	27/18614	0.3923086	0.55066679	0.41812121	CD3E	1
BP	GO:0034643	establishment of mitochondrion localization, microtubule-	1/340	27/18614	0.3923086	0.55066679	0.41812121	SYBU	1

BP	GO:0035929	mediated steroid hormone secretion	1/340	27/18614	0.39230086	0.55066679	0.41812121	INHBA	1
BP	GO:0042133	neurotransmitter metabolic process	1/340	27/18614	0.39230086	0.55066679	0.41812121	MAOA	1
BP	GO:0045745	positive regulation of G protein-coupled receptor signaling pathway	1/340	27/18614	0.39230086	0.55066679	0.41812121	SLC39A14	1

BP	GO:0045954	positive regulation of natural killer cell mediated cytotoxicity	1/340	27/18614	0.39230 86	0.55066 79	0.41812 1	RASGRP1	1
BP	GO:0046037	GMP metabolic process	1/340	27/18614	0.39230 86	0.55066 79	0.41812 1	AMPD1	1
BP	GO:0047497	mitochondrion transport along microtubule	1/340	27/18614	0.39230 86	0.55066 79	0.41812 1	SYBU	1
BP	GO:0050996	positive regulation of lipid cataboli	1/340	27/18614	0.39230 86	0.55066 79	0.41812 1	IL1B	1

		c process							
BP	GO:0060547	negative regulation of necrotic cell death	1/340	27/18614	0.3923086	0.55066679	0.41812121	DDR2	1
BP	GO:0071467	cellular response to pH	1/340	27/18614	0.3923086	0.55066679	0.41812121	SCNN1G	1
BP	GO:0071549	cellular response to dexamethasone stimulus	1/340	27/18614	0.3923086	0.55066679	0.41812121	DDIT4	1
BP	GO:0090200	positive regulation of release	1/340	27/18614	0.3923086	0.55066679	0.41812121	TNFSF10	1

		of cytochrome c from mitochondria							
BP	GO:1902932	positive regulation of alcohol biosynthetic process	1/340	27/18614	0.39230886	0.55066679	0.41812121	P2RY1	1
BP	GO:1903523	negative regulation of blood circulation	1/340	27/18614	0.39230886	0.55066679	0.41812121	PIK3CG	1
BP	GO:1905564	positive regulation of vascular	1/340	27/18614	0.39230886	0.55066679	0.41812121	ITGA4	1

		endothelial cell proliferation							
BP	GO:2000737	negative regulation of stem cell differentiation	1/340	27/18614	0.39230086	0.55066679	0.418121	ZFP36	1
BP	GO:0002067	glandular epithelial cell differentiation	2/340	74/18614	0.39273556	0.55066679	0.418121	BMP5/FASN	2
BP	GO:0006023	aminoglycan biosynthetic process	2/340	74/18614	0.39273556	0.55066679	0.418121	IL1B/HAS2	2

BP	GO:0007585	respiratory gaseous exchange by respiratory system	2/340	74/18614	0.39273 56	0.55066 79	0.41812 1	CYSLTR1/NR4A2	2
BP	GO:0009247	glycolipid biosynthetic process	2/340	74/18614	0.39273 56	0.55066 79	0.41812 1	PIGW/ST6GALNAC3	2
BP	GO:0030301	cholesterol transport	3/340	124/18614	0.39551 69	0.55436 38	0.42092 72	ABCA8/LIPG/CFTR	3
BP	GO:0010822	positive regulation of mitochondrion organization	2/340	75/18614	0.39917 85	0.55826 13	0.42388 66	DCN/TNFSF10	2

BP	GO:0016239	positive regulation of macroautophagy	2/340	75/18614	0.39917 85	0.55826 13	0.42388 66	HMOX1/DCN	2
BP	GO:0030968	endoplasmic reticulum unfolded protein response	2/340	75/18614	0.39917 85	0.55826 13	0.42388 66	CTH/ATF3	2
BP	GO:0043536	positive regulation of blood vessel endothelial cell migration	2/340	75/18614	0.39917 85	0.55826 13	0.42388 66	PTGS2/HMOX1	2

BP	GO:0050818	regulation of coagulation	2/340	75/18614	0.399178 5	0.558261 3	0.423886 6	SERPINF2/FGA	2
BP	GO:0002832	negative regulation of response to biotic stimulus	3/340	125/18614	0.400437 9	0.558261 3	0.423886 6	CD96/VSIG4/TRIB1	3
BP	GO:0030509	BMP signaling pathway	4/340	176/18614	0.401415	0.558261 3	0.423886 6	TGFB2/EGR1/SULF1/INHBA	4
BP	GO:0051099	positive regulation of binding	4/340	176/18614	0.401415	0.558261 3	0.423886 6	TRIB3/ACE/PYHIN1/CSF3	4
BP	GO:0000303	response to superoxide	1/340	28/18614	0.403424 7	0.558261 3	0.423886 6	NQO1	1

BP	GO:0002227	innate immune response in mucosa	1/340	28/18614	0.40342 47	0.55826 13	0.42388 66	RNASE2		1
BP	GO:0006027	glycosaminoglycan catabolic process	1/340	28/18614	0.40342 47	0.55826 13	0.42388 66	LYVE1		1
BP	GO:0006829	zinc ion transport	1/340	28/18614	0.40342 47	0.55826 13	0.42388 66	SLC39A14		1
BP	GO:0007288	sperm axoneme assembly	1/340	28/18614	0.40342 47	0.55826 13	0.42388 66	SPEF2		1
BP	GO:00	negative regulati	1/340	28/18614	0.403	0.558	0.423	BMP5		1

	108<br>94	on of<br>steroid<br>biosynt<br>hetic<br>proces<br>s			42<br>47	26<br>13	88<br>66			
B<br>P	GO<br>:00<br>109<br>71	positive<br>regulati<br>on of<br>G2/M<br>transiti<br>on of<br>mitotic<br>cell<br>cycle	1/<br>34<br>0	28/<br>186<br>14	0.4<br>03<br>42<br>47	0.5<br>58<br>26<br>13	0.4<br>23<br>88<br>66	DTL		1
B<br>P	GO<br>:00<br>182<br>00	peptidyl<br>-<br>glutami<br>c acid<br>modific<br>ation	1/<br>34<br>0	28/<br>186<br>14	0.4<br>03<br>42<br>47	0.5<br>58<br>26<br>13	0.4<br>23<br>88<br>66	AGBL1		1
B<br>P	GO<br>:00<br>193<br>59	nicotina<br>mide<br>nucleoti<br>de<br>biosynt	1/<br>34<br>0	28/<br>186<br>14	0.4<br>03<br>42<br>47	0.5<br>58<br>26<br>13	0.4<br>23<br>88<br>66	IDO1		1

		hetic process								
BP	GO:0019363	pyridine nucleotide biosynthetic process	1/340	28/18614	0.40334247	0.55826613	0.42388866	IDO1		1
BP	GO:0022011	myelination in peripheral nervous system	1/340	28/18614	0.40334247	0.55826613	0.42388866	NTRK2		1
BP	GO:0030194	positive regulation of blood coagulation	1/340	28/18614	0.40334247	0.55826613	0.42388866	SERPINF2		1

BP	GO:0030262	apoptotic nuclear changes	1/340	28/18614	0.40342 47	0.55826 13	0.42388 66	IL6	1
BP	GO:0030325	adrenal gland development	1/340	28/18614	0.40342 47	0.55826 13	0.42388 66	ARID5B	1
BP	GO:0032292	peripheral nervous system axon ensheathment	1/340	28/18614	0.40342 47	0.55826 13	0.42388 66	NTRK2	1
BP	GO:0032958	inositol phosphate biosynthetic process	1/340	28/18614	0.40342 47	0.55826 13	0.42388 66	P2RY1	1

BP	GO:0036037	CD8-positive, alpha-beta T cell activation	1/340	28/18614	0.40342 47	0.55826 13	0.42388 66	IRF1	1
BP	GO:0042634	regulation of hair cycle	1/340	28/18614	0.40342 47	0.55826 13	0.42388 66	TGFB2	1
BP	GO:0042759	long-chain fatty acid biosynthetic process	1/340	28/18614	0.40342 47	0.55826 13	0.42388 66	ALOX15B	1
BP	GO:0043949	regulation of cAMP-mediated signaling	1/340	28/18614	0.40342 47	0.55826 13	0.42388 66	APLNR	1

BP	GO:0046337	phosphatidylethanolamine metabolic process	1/340	28/18614	0.4034247	0.55826613	0.42388866	PLA2G2A	1
BP	GO:0048679	regulation of axon regeneration	1/340	28/18614	0.4034247	0.55826613	0.42388866	TNR	1
BP	GO:0060219	camera-type eye photoreceptor cell differentiation	1/340	28/18614	0.4034247	0.55826613	0.42388866	NTRK2	1
BP	GO:0060402	calcium ion transport into cytosol	1/340	28/18614	0.4034247	0.55826613	0.42388866	RYR2	1

BP	GO:0070723	response to cholesterol	1/340	28/18614	0.40342247	0.55826130	0.42388866	INHBA	1
BP	GO:1900048	positive regulation of hemostasis	1/340	28/18614	0.40342247	0.55826130	0.42388866	SERPINF2	1
BP	GO:1903019	negative regulation of glycoprotein metabolic process	1/340	28/18614	0.40342247	0.55826130	0.42388866	PTX3	1
BP	GO:0000956	nuclear-transcribed mRNA catabolic	3/340	126/18614	0.40534477	0.56003366	0.42523346	PNRC1/BTG2/ZFP36	3

		process							
BP	GO:0021953	central nervous system neuron differentiation	4/340	177/18614	0.4055324	0.5600366	0.4252346	BTG2/INHBA/NTRK2/NR4A2	4
BP	GO:0050680	negative regulation of epithelial cell proliferation	4/340	177/18614	0.4055324	0.5600366	0.4252346	TGFB2/SULF1/SFN/CCL2	4
BP	GO:0002637	regulation of immunoglobulin production	2/340	76/18614	0.4055893	0.5600366	0.4252346	FCGR2B/IL6	2

BP	GO:0006919	activation of cysteine-type endopeptidase activity involved in apoptotic process	2/340	76/18614	0.40558 93	0.56003 66	0.42523 46	NLRC4/S100A8	2
BP	GO:0086003	cardiac muscle cell contraction	2/340	76/18614	0.40558 93	0.56003 66	0.42523 46	RYR2/ANK2	2
BP	GO:0001654	eye development	8/340	385/18614	0.40616 24	0.56062 47	0.42568 12	TGFB2/JUN/SH3PXD2B/SLC7A11/FAT3/COL8A1/INHBA/NTRK2	8
BP	GO:0022412	cellular process involve	9/340	438/18614	0.40747 27	0.56222 98	0.42689 99	RPS6KA2/ROBO2/PTGDS/HPGDS/SPEF2/SLIT3/INHBA/CFTR/PTX3	9

		d in reproduction in multicellular organism							
BP	GO:0001704	formation of primary germ layer	3/340	127/18614	0.41024 58	0.56544 19	0.42933 88	ITGA4/COL8A1/INHBA	3
BP	GO:0048593	camera-type eye morphogenesis	3/340	127/18614	0.41024 58	0.56544 19	0.42933 88	FAT3/COL8A1/NTRK2	3
BP	GO:0090288	negative regulation of cellular response to growth factor	3/340	127/18614	0.41024 58	0.56544 19	0.42933 88	SULF1/DCN/SEMA6A	3

		stimulus							
BP	GO:0001937	negative regulation of endothelial cell proliferation	2/340	77/18614	0.41196 73	0.56718 48	0.43066 22	SULF1/CCL2	2
BP	GO:0045685	regulation of glial cell differentiation	2/340	77/18614	0.41196 73	0.56718 48	0.43066 22	EGR2/IL6	2
BP	GO:1901568	fatty acid derivative metabolic process	2/340	77/18614	0.41196 73	0.56718 48	0.43066 22	HMGCLL1/ALOX15B	2

BP	GO:0043534	blood vessel endothelial cell migration	4/340	179/18614	0.4137508	0.5671848	0.4306622	PTGS2/EGR3/HMOX1/NR4A1	4
BP	GO:0050954	sensory perception of mechanical stimulus	4/340	179/18614	0.4137508	0.5671848	0.4306622	CXCL12/PCDH15/PKHD1L1/SLC1A3	4
BP	GO:0000305	response to oxygen radical	1/340	29/18614	0.4143381	0.5671848	0.4306622	NQO1	1
BP	GO:0001773	myeloid dendritic cell activation	1/340	29/18614	0.4143381	0.5671848	0.4306622	CD2	1

BP	GO:0003094	glomerular filtration	1/340	29/18614	0.41433381	0.5671848	0.43066622	SULF1	1
BP	GO:0003209	cardiac atrium morphogenesis	1/340	29/18614	0.41433381	0.5671848	0.43066622	TGFB2	1
BP	GO:0003416	endochondral bone growth	1/340	29/18614	0.41433381	0.5671848	0.43066622	DDR2	1
BP	GO:0006910	phagocytosis, recognition	1/340	29/18614	0.41433381	0.5671848	0.43066622	PTX3	1
BP	GO:0007271	synaptic transmission, cholinergic	1/340	29/18614	0.41433381	0.5671848	0.43066622	NQO1	1
BP	GO:0009312	oligosaccharide biosynt	1/340	29/18614	0.41433381	0.5671848	0.43066622	FUT3	1

		hetic process							
BP	GO:0021952	central nervous system projection neuron axonogenesis	1/340	29/18614	0.41433381	0.56718 48	0.43066 22	NR4A2	1
BP	GO:0072530	purine-containing compound transmembrane transport	1/340	29/18614	0.41433381	0.56718 48	0.43066 22	AQP9	1
BP	GO:0099623	regulation of cardiac muscle	1/340	29/18614	0.41433381	0.56718 48	0.43066 22	ANK2	1

BP	GO:0099625	cell membrane repolarization ventricular cardiac muscle cell membrane repolarization	1/340	29/18614	0.41433 81	0.56718 48	0.43066 22	ANK2		1
BP	GO:1901018	positive regulation of potassium ion transmembrane transporter activity	1/340	29/18614	0.41433 81	0.56718 48	0.43066 22	ANK2		1

BP	GO:1902430	negative regulation of amyloid-beta formation	1/340	29/18614	0.41433381	0.5671848	0.4306622	NTRK2	1
BP	GO:0150063	visual system development	8/340	389/18614	0.41726625	0.57098629	0.4335461	TGFB2/JUN/SH3PXD2B/SLC7A11/FAT3/COL8A1/INHBA/NTRK2	8
BP	GO:0010594	regulation of endothelial cell migration	5/340	232/18614	0.41817799	0.57139922	0.4338569	PTGS2/PIK3CG/HMOX1/DCN/ADAMTS9	5
BP	GO:0002220	innate immune response activating cell	2/340	78/18614	0.41831213	0.57139922	0.4338569	TRIL/TNIP3	2

		surface receptor signaling pathway							
BP	GO:0032720	negative regulation of tumor necrosis factor production	2/340	78/18614	0.41831313	0.57139222	0.43385569	ORM1/CX3CL1	2
BP	GO:0045824	negative regulation of innate immune response	2/340	78/18614	0.41831313	0.57139222	0.43385569	CD96/VSIG4	2

BP	GO:0070373	negative regulation of ERK1 and ERK2 cascade	2/340	78/18614	0.4183113	0.5713922	0.4338569	ATF3/SEMA6A	2
BP	GO:0019079	viral genome replication	3/340	129/18614	0.4200043	0.5734993	0.4354568	CCL5/CCL2/CCL8	3
BP	GO:0043010	camera-type eye development	7/340	338/18614	0.422079	0.5748411	0.4364756	TGFB2/JUN/SLC7A11/FAT3/COL8A1/INHBA/NTRK2	7
BP	GO:0032371	regulation of sterol transport	2/340	79/18614	0.4246205	0.5748411	0.4364756	ABCA8/LIPG	2

BP	GO:0032374	regulation of cholesterol transport	2/340	79/18614	0.4246205	0.5748411	0.43647756	ABCA8/LIPG	2
BP	GO:0086001	cardiac muscle cell action potential	2/340	79/18614	0.4246205	0.5748411	0.43647756	RYR2/ANK2	2
BP	GO:2000736	regulation of stem cell differentiation	2/340	79/18614	0.4246205	0.5748411	0.43647756	TGFB2/ZFP36	2
BP	GO:0007266	Rho protein signal transduction	3/340	130/18614	0.4248636	0.5748411	0.43647756	RHOBTB2/GPR4/P2RY10	3
BP	GO:00	oocyte maturation	1/340	30/18614	0.425	0.574	0.436	RPS6KA2	1

	01556				0524	8411	4756		
BP	GO:0006359	regulation of transcription by RNA polymerase III	1/340	30/18614	0.4250524	0.5748411	0.4364756	INHBA	1
BP	GO:0006921	cellular component disassembly involved in execution phase of apoptosis	1/340	30/18614	0.4250524	0.5748411	0.4364756	IL6	1
BP	GO:0007202	activation of phospholipase	1/340	30/18614	0.4250524	0.5748411	0.4364756	SELE	1

		C activity							
BP	GO:0008299	isoprenoid biosynthetic process	1/340	30/18614	0.4250524	0.5748411	0.4364756	AKR1C3	1
BP	GO:0015721	bile acid and bile salt transport	1/340	30/18614	0.4250524	0.5748411	0.4364756	AQP9	1
BP	GO:0015740	C4-dicarboxylate transport	1/340	30/18614	0.4250524	0.5748411	0.4364756	SLC1A3	1
BP	GO:0021602	cranial nerve morphogenesis	1/340	30/18614	0.4250524	0.5748411	0.4364756	EGR2	1
BP	GO:00	protein localization to	1/340	30/18614	0.425	0.574	0.436	GBP5	1

	34067	Golgi apparatus			0524	8411	4756		
BP	GO:0043094	cellular metabolic compound salvage	1/340	30/18614	0.4250524	0.5748411	0.4364756	AMPD1	1
BP	GO:0050820	positive regulation of coagulation	1/340	30/18614	0.4250524	0.5748411	0.4364756	SERPINF2	1
BP	GO:0062125	regulation of mitochondrial gene expression	1/340	30/18614	0.4250524	0.5748411	0.4364756	SHMT2	1
BP	GO:0070168	negative regulation of biomin	1/340	30/18614	0.4250524	0.5748411	0.4364756	ASPN	1

		eral tissue development							
BP	GO:0071480	cellular response to gamma radiation	1/340	30/18614	0.42505 24	0.57484 11	0.43647 56	EGR1	1
BP	GO:0090140	regulation of mitochondrial fission	1/340	30/18614	0.42505 24	0.57484 11	0.43647 56	DCN	1
BP	GO:0090382	phagosome maturation	1/340	30/18614	0.42505 24	0.57484 11	0.43647 56	SRPX	1
BP	GO:1900027	regulation of ruffle assembly	1/340	30/18614	0.42505 24	0.57484 11	0.43647 56	STAP1	1

BP	GO:1901798	positive regulation of signal transduction by p53 class mediator	1/340	30/18614	0.42505 24	0.57484 11	0.43647 56	PYHIN1	1
BP	GO:1902230	negative regulation of intrinsic apoptotic signaling pathway in response to DNA damage	1/340	30/18614	0.42505 24	0.57484 11	0.43647 56	CXCL12	1

BP	GO:0071214	cellular response to abiotic stimulus	7/340	339/18614	0.42505 96	0.57484 11	0.43647 56	SCNN1G/PTGS2/EGR1/MFAP4/CAPN3/IL1B/IRF1	7
BP	GO:0104004	cellular response to environmental stimulus	7/340	339/18614	0.42505 96	0.57484 11	0.43647 56	SCNN1G/PTGS2/EGR1/MFAP4/CAPN3/IL1B/IRF1	7
BP	GO:0050807	regulation of synapse organization	5/340	234/18614	0.42537 27	0.57506 05	0.43664 22	ROBO2/SLC7A11/LRRN3/FCGR2B/NTRK2	5
BP	GO:0048736	appendage development	4/340	182/18614	0.42603 16	0.57554 28	0.43700 84	TGFB2/PKDCC/FMN1/SLC7A11	4
BP	GO:00	limb development	4/340	182/18614	0.426	0.575	0.437	TGFB2/PKDCC/FMN1/SLC7A11	4

	601<br>73				03<br>16	54<br>28	00<br>84		
B<br>P	GO<br>:00<br>066<br>61	phosph<br>atidylin<br>ositol<br>biosynt<br>hetic<br>proces<br>s	3/<br>34<br>0	131<br>/18<br>614	0.4<br>29<br>70<br>89	0.5<br>79<br>89<br>39	0.4<br>40<br>31<br>22	PIGW/PIK3CG/SOCS3	3
B<br>P	GO<br>:00<br>105<br>95	positive<br>regulati<br>on of<br>endoth<br>elial<br>cell<br>migrati<br>on	3/<br>34<br>0	131<br>/18<br>614	0.4<br>29<br>70<br>89	0.5<br>79<br>89<br>39	0.4<br>40<br>31<br>22	PTGS2/PIK3CG/HMOX1	3
B<br>P	GO<br>:00<br>300<br>48	actin<br>filamen<br>t-based<br>movem<br>ent	3/<br>34<br>0	131<br>/18<br>614	0.4<br>29<br>70<br>89	0.5<br>79<br>89<br>39	0.4<br>40<br>31<br>22	RYR2/ANK2/MYH10	3
B<br>P	GO<br>:00<br>434<br>09	negativ<br>e<br>regulati<br>on of	4/<br>34<br>0	183<br>/18<br>614	0.4<br>30<br>11<br>11	0.5<br>80<br>05<br>55	0.4<br>40<br>43<br>49	LAX1/IL1B/ATF3/SEMA6A	4

BP	GO:0030500	MAPK cascade regulation of bone mineralization	2/340	80/18614	0.430894	0.580555	0.440349	PKDCC/DDR2	2
BP	GO:0048844	artery morphogenesis	2/340	80/18614	0.430894	0.580555	0.440349	TGFB2/ADAMTS9	2
BP	GO:0071230	cellular response to amino acid stimulus	2/340	80/18614	0.430894	0.580555	0.440349	GCLM/NTRK2	2
BP	GO:0090049	regulation of cell migration involved in	2/340	80/18614	0.430894	0.580555	0.440349	PTGS2/HMOX1	2

		sprouting angiogenesis							
BP	GO:0140056	organelle localization by membrane tethering	2/340	80/18614	0.430894	0.580055	0.440349	SYTL2/CFTR	2
BP	GO:1903556	negative regulation of tumor necrosis factor superfamily cytokine production	2/340	80/18614	0.430894	0.580055	0.440349	ORM1/CX3CL1	2

BP	GO:0048880	sensory system development	8/340	395/18614	0.43389 12	0.58203 43	0.44193 74	TGFB2/JUN/SH3PXD2B/SLC7A11/FAT3/COL8A1/INHBA/NTRK2	8
BP	GO:0042770	signal transduction in response to DNA damage	4/340	184/18614	0.43418 31	0.58203 43	0.44193 74	PPP1R10/IER3/PYHIN1/DTL	4
BP	GO:0002244	hematopoietic progenitor cell differentiation	3/340	132/18614	0.43453 95	0.58203 43	0.44193 74	ACE/ZFP36/INHBA	3
BP	GO:0031570	DNA integrity checkpoint signaling	3/340	132/18614	0.43453 95	0.58203 43	0.44193 74	PPP1R10/IER3/DTL	3

BP	GO:0003309	type B pancreatic cell differentiation	1/340	31/18614	0.43557 12	0.58203 43	0.44193 74	BMP5		1
BP	GO:0010039	response to iron ion	1/340	31/18614	0.43557 12	0.58203 43	0.44193 74	HMOX1		1
BP	GO:0010667	negative regulation of cardiac muscle cell apoptotic process	1/340	31/18614	0.43557 12	0.58203 43	0.44193 74	PPP1R10		1
BP	GO:0014044	Schwann cell development	1/340	31/18614	0.43557 12	0.58203 43	0.44193 74	NTRK2		1

BP	GO:0017145	stem cell division	1/340	31/18614	0.43555712	0.58200343	0.44193974	TGFB2	1
BP	GO:0045939	negative regulation of steroid metabolic process	1/340	31/18614	0.43555712	0.58200343	0.44193974	BMP5	1
BP	GO:0045948	positive regulation of translational initiation	1/340	31/18614	0.43555712	0.58200343	0.44193974	CCL5	1
BP	GO:0045987	positive regulation of smooth muscle	1/340	31/18614	0.43555712	0.58200343	0.44193974	PTGS2	1

		contraction							
BP	GO:0048240	sperm capacitation	1/340	31/18614	0.43557 12	0.58203 43	0.44193 74	CFTR	1
BP	GO:0048873	homeostasis of number of cells within a tissue	1/340	31/18614	0.43557 12	0.58203 43	0.44193 74	NFKBIZ	1
BP	GO:0051491	positive regulation of filopodium assembly	1/340	31/18614	0.43557 12	0.58203 43	0.44193 74	DPYSL3	1
BP	GO:0051654	establishment of mitochondrion	1/340	31/18614	0.43557 12	0.58203 43	0.44193 74	SYBU	1

		localization							
BP	GO:0097205	renal filtration	1/340	31/18614	0.4355712	0.5820343	0.4419374	SULF1	1
BP	GO:0150117	positive regulation of cell-substrate junction organization	1/340	31/18614	0.4355712	0.5820343	0.4419374	FMN1	1
BP	GO:1900225	regulation of NLRP3 inflammasome complex assembly	1/340	31/18614	0.4355712	0.5820343	0.4419374	GBP5	1

BP	GO:1900745	positive regulation of p38MAPK cascade	1/340	31/18614	0.43557 12	0.58203 43	0.44193 74	IL1B		1
BP	GO:1902751	positive regulation of cell cycle G2/M phase transition	1/340	31/18614	0.43557 12	0.58203 43	0.44193 74	DTL		1
BP	GO:0044262	cellular carbohydrate metabolic process	6/340	290/18614	0.43687 67	0.58350 5	0.44305 41	P2RY1/IDH1/IER3/DDIT4/HAS2/MGAM		6
BP	GO:00	adult locomotory	2/340	81/18614	0.437	0.583	0.443	CXCL12/NR4A2		2

	08344	behavior			1312	505	0541		
BP	GO:0031016	pancreas development	2/340	81/18614	0.4371312	0.583505	0.4430541	BMP5/IL6	2
BP	GO:0010952	positive regulation of peptidase activity	4/340	185/18614	0.4382471	0.5847898	0.4440296	NLRC4/UACA/S100A8/TNFSF10	4
BP	GO:0030324	lung development	4/340	186/18614	0.4423027	0.5888142	0.4470854	PKDCC/ACE/CRISPLD2/SLC7A11	4
BP	GO:2000045	regulation of G1/S transition of mitotic cell cycle	4/340	186/18614	0.4423027	0.5888142	0.4470854	WEE1/DDR2/INHBA/CCL2	4

BP	GO:0006446	regulation of translational initiation	2/340	82/18614	0.44333311	0.58881420	0.44708054	EIF4EBP1/CCL5	2
BP	GO:0046928	regulation of neurotransmitter secretion	2/340	82/18614	0.44333311	0.58881420	0.44708054	P2RY1/CSPG5	2
BP	GO:0050848	regulation of calcium-mediated signaling	2/340	82/18614	0.44333311	0.58881420	0.44708054	TRAT1/CD3E	2
BP	GO:1901657	glycosyl compound metabo	2/340	82/18614	0.44333311	0.58881420	0.44708054	AKR1C2/AKR1C3	2

		lic proces s								
BP	GO:1905897	regulation of response to endoplasmic reticulum stress	2/340	82/18614	0.44333 11	0.58881 42	0.44708 54	HYOU1/FCGR2B		2
BP	GO:2001021	negative regulation of response to DNA damage stimulus	2/340	82/18614	0.44333 11	0.58881 42	0.44708 54	CXCL12/PPP1R10		2
BP	GO:00	digestive tract	3/340	134/18614	0.444	0.588	0.447	TGFB2/PKDCC/RARRES2		3

	48565	development			155	8142	0854		
BP	GO:0032956	regulation of actin cytoskeleton organization	7/340	346/18614	0.4458711	0.5888142	0.4470854	CXCL12/SERPINF2/STAP1/PLEKHH2/FMN1/CX3CL1/CSF3	7
BP	GO:0002717	positive regulation of natural killer cell mediated immunity	1/340	32/18614	0.4458982	0.5888142	0.4470854	RASGRP1	1
BP	GO:0033338	metanephros morphogenesis	1/340	32/18614	0.4458982	0.5888142	0.4470854	FMN1	1
BP	GO:00	tricarboxylic	1/340	32/18614	0.445	0.588	0.447	IDH1	1

	06099	acid cycle			8982	8142	0854		
BP	GO:0006506	GPI anchor biosynthetic process	1/340	32/18614	0.4458982	0.5888142	0.4470854	PIGW	1
BP	GO:0006613	cotranslational protein targeting to membrane	1/340	32/18614	0.4458982	0.5888142	0.4470854	SRPRB	1
BP	GO:0010669	epithelial structure maintenance	1/340	32/18614	0.4458982	0.5888142	0.4470854	SERPINA3	1
BP	GO:0019082	viral protein processing	1/340	32/18614	0.4458982	0.5888142	0.4470854	ST6GALNAC3	1

BP	GO:0030204	chondroitin sulfate metabolic process	1/340	32/18614	0.44589 82	0.58881 42	0.44708 54	DCN	1
BP	GO:0031057	negative regulation of histone modification	1/340	32/18614	0.44589 82	0.58881 42	0.44708 54	SDR16C5	1
BP	GO:0031069	hair follicle morphogenesis	1/340	32/18614	0.44589 82	0.58881 42	0.44708 54	TGFB2	1
BP	GO:0032770	positive regulation of monooxygenase activity	1/340	32/18614	0.44589 82	0.58881 42	0.44708 54	IL1B	1

BP	GO:0033032	regulation of myeloid cell apoptotic process	1/340	32/18614	0.4458982	0.5888142	0.4470854	SLC7A11		1
BP	GO:0034389	lipid droplet organization	1/340	32/18614	0.4458982	0.5888142	0.4470854	PLIN2		1
BP	GO:0043552	positive regulation of phosphatidylinositol 3-kinase activity	1/340	32/18614	0.4458982	0.5888142	0.4470854	FGR		1
BP	GO:0045070	positive regulation of viral genom	1/340	32/18614	0.4458982	0.5888142	0.4470854	CCL5		1

		e replication							
BP	GO:0045671	negative regulation of osteoclast differentiation	1/340	32/18614	0.4458982	0.5888142	0.4470854	TNFRSF11B	1
BP	GO:0061037	negative regulation of cartilage development	1/340	32/18614	0.4458982	0.5888142	0.4470854	FRZB	1
BP	GO:0071295	cellular response to vitamin	1/340	32/18614	0.4458982	0.5888142	0.4470854	POSTN	1
BP	GO:00	bone growth	1/340	32/18614	0.445	0.588	0.447	DDR2	1

	98868				8982	8142	0854		
BP	GO:1902235	regulation of endoplasmic reticulum stress-induced intrinsic apoptotic signaling pathway	1/340	32/18614	0.4458982	0.5888142	0.4470854	HYOU1	1
BP	GO:0071772	response to BMP	4/340	187/18614	0.4463497	0.5890023	0.4472282	TGFB2/EGR1/SULF1/INHBA	4
BP	GO:0071773	cellular response to BMP	4/340	187/18614	0.4463497	0.5890023	0.4472282	TGFB2/EGR1/SULF1/INHBA	4

		stimulus							
BP	GO:0050803	regulation of synapse structure or activity	5/340	240/18614	0.4468434	0.5894497	0.4475679	ROBO2/SLC7A11/LRRN3/FCGR2B/NTRK2	5
BP	GO:0006612	protein targeting to membrane	3/340	135/18614	0.4489387	0.5919206	0.494444	HYOU1/SRPRB/ZDHHC9	3
BP	GO:0031424	keratinization	2/340	83/18614	0.4949931	0.5919206	0.494444	KRT7/SFN	2
BP	GO:0032024	positive regulation of insulin secretion	2/340	83/18614	0.4949931	0.5919206	0.494444	ANO1/CFTR	2

BP	GO:0034340	response to type I interferon	2/340	83/18614	0.4494931	0.5919206	0.449444	SHMT2/CH25H	2
BP	GO:0035023	regulation of Rho protein signal transduction	2/340	83/18614	0.4494931	0.5919206	0.449444	GPR4/P2RY10	2
BP	GO:0006813	potassium ion transport	5/340	241/18614	0.4504034	0.5929145	0.4501987	FXYD1/KCNAB1/ANK2/CASQ2/SLC1A3	5
BP	GO:0000086	G2/M transition of mitotic cell cycle	3/340	136/18614	0.4537059	0.594783	0.4516175	WEE1/IER3/DTL	3
BP	GO:0001101	response to acid	3/340	136/18614	0.4537059	0.594783	0.4516175	GCLM/NQO1/NTRK2	3

BP	GO:0098659	chemical inorganic cation import across plasma membrane	3/340	136/18614	0.4537059	0.5947783	0.4516175	SCNN1G/MS4A1/SLC39A14	3
BP	GO:0099587	inorganic ion import across plasma membrane	3/340	136/18614	0.4537059	0.5947783	0.4516175	SCNN1G/MS4A1/SLC39A14	3
BP	GO:0006094	gluconeogenesis	2/340	84/18614	0.4556164	0.5947783	0.4516175	SLC39A14/ATF3	2
BP	GO:0002063	chondrocyte development	1/340	33/18614	0.4560367	0.5947783	0.4516175	SULF1	1

BP	GO:0006308	DNA catabolic process	1/340	33/18614	0.45603367	0.594783	0.45161675	IL6	1
BP	GO:0006505	GPI anchor metabolic process	1/340	33/18614	0.45603367	0.594783	0.45161675	PIGW	1
BP	GO:0006958	complement activation, classical pathway	1/340	33/18614	0.45603367	0.594783	0.45161675	C1R	1
BP	GO:0007035	vacuolar acidification	1/340	33/18614	0.45603367	0.594783	0.45161675	SLC11A1	1
BP	GO:00	negative regulati	1/340	33/18614	0.456	0.594	0.451	PPP1R10	1

	106 64	on of striated muscle cell apoptot ic proces s			03 67	78 3	61 75		
B P	GO :00 183 45	protein palmito ylation	1/ 34 0	33/ 186 14	0.4 56 03 67	0.5 94 78 3	0.4 51 61 75	ZDHHC9	1
B P	GO :00 217 72	olfactor y bulb develo pment	1/ 34 0	33/ 186 14	0.4 56 03 67	0.5 94 78 3	0.4 51 61 75	ROBO2	1
B P	GO :00 309 47	regulati on of vascula r endoth elial growth factor recepto r	1/ 34 0	33/ 186 14	0.4 56 03 67	0.5 94 78 3	0.4 51 61 75	IL1B	1

		signaling pathway							
BP	GO:0036314	response to sterol	1/340	33/18614	0.45603367	0.594783	0.45161675	INHBA	1
BP	GO:0040020	regulation of meiotic nuclear division	1/340	33/18614	0.45603367	0.594783	0.45161675	RPS6KA2	1
BP	GO:0042573	retinoic acid metabolic process	1/340	33/18614	0.45603367	0.594783	0.45161675	AKR1C3	1
BP	GO:0048384	retinoic acid receptor signaling	1/340	33/18614	0.45603367	0.594783	0.45161675	AKR1C3	1

		pathway							
BP	GO:0048710	regulation of astrocyte differentiation	1/340	33/18614	0.4560367	0.594783	0.4516175	IL6	1
BP	GO:0050858	negative regulation of antigen receptor-mediated signaling pathway	1/340	33/18614	0.4560367	0.594783	0.4516175	FCGR2B	1
BP	GO:0071711	basement membrane	1/340	33/18614	0.4560367	0.594783	0.4516175	HMCN1	1

		organization								
BP	GO:1902992	negative regulation of amyloid precursor protein catabolic process	1/340	33/18614	0.45603 67	0.59478 3	0.45161 75	NTRK2		1
BP	GO:1903715	regulation of aerobic respiration	1/340	33/18614	0.45603 67	0.59478 3	0.45161 75	SHMT2		1
BP	GO:1904893	negative regulation of receptor signalin	1/340	33/18614	0.45603 67	0.59478 3	0.45161 75	SOCS3		1

		g pathway via STAT							
BP	GO:1905207	regulation of cardiocyte differentiation	1/340	33/18614	0.45603067	0.5947783	0.45161675	TGFB2	1
BP	GO:1905476	negative regulation of protein localization to membrane	1/340	33/18614	0.45603067	0.5947783	0.45161675	PKDCC	1
BP	GO:2000191	regulation of fatty acid transport	1/340	33/18614	0.45603067	0.5947783	0.45161675	IL1B	1

BP	GO:0048738	cardiac muscle tissue development	5/340	243/18614	0.4577505	0.5964945	0.4529917	RYR2/BMP5/TGFB2/ADAMTS9/MYH11	5
BP	GO:0030323	respiratory tube development	4/340	190/18614	0.4584352	0.5969169	0.4532377	PKDCC/ACE/CRISPLD2/SLC7A11	4
BP	GO:0003158	endothelium development	3/340	137/18614	0.458456	0.5969169	0.4532377	FASN/IL1B/CXCL10	3
BP	GO:0035270	endocrine system development	3/340	137/18614	0.458456	0.5969169	0.4532377	ARID5B/BMP5/IL6	3
BP	GO:1903052	positive regulation of proteolysis involved in	3/340	137/18614	0.458456	0.5969169	0.4532377	TRIB3/PYHIN1/TRIB1	3

		protein catabolic process							
BP	GO:0010660	regulation of muscle cell apoptotic process	2/340	85/18614	0.4617005	0.600321	0.4558224	PPP1R10/HMOX1	2
BP	GO:0019935	cyclic-nucleotide-mediated signaling	2/340	85/18614	0.4617005	0.600321	0.4558224	PDE7B/APLNR	2
BP	GO:0035050	embryonic heart tube development	2/340	85/18614	0.4617005	0.600321	0.4558224	RYR2/APLNR	2

BP	GO:1902369	negative regulation of RNA catabolic process	2/340	85/18614	0.4617005	0.600321	0.45582224	ZFP36/SLC11A1	2
BP	GO:0051260	protein homooligomerization	4/340	191/18614	0.46244442	0.60108280	0.45640409	SHMT2/NLRC4/CTH/GBP5	4
BP	GO:0015918	sterol transport	3/340	138/18614	0.46318885	0.60158584	0.45678848	ABCA8/LIPG/CFTR	3
BP	GO:0022604	regulation of cell morphogenesis	5/340	245/18614	0.4645582	0.60158584	0.45678848	P2RY1/FGD2/FGR/MYH10/CCL2	5
BP	GO:0030522	intracellular receptor	6/340	299/18614	0.46582802	0.60158584	0.45678848	NR4A3/AKR1C3/NR1D2/ALOX15B/NR4A1/NR4A2	6

		signaling pathway							
BP	GO:0002021	response to dietary excess	1/340	34/18614	0.4659903	0.6015884	0.4567848	SGIP1	1
BP	GO:0006688	glycosphingolipid biosynthetic process	1/340	34/18614	0.4659903	0.6015884	0.4567848	ST6GALNAC3	1
BP	GO:0009112	nucleobase metabolic process	1/340	34/18614	0.4659903	0.6015884	0.4567848	SHMT2	1
BP	GO:0009156	ribonucleoside monophosphate	1/340	34/18614	0.4659903	0.6015884	0.4567848	AMPD1	1

		biosynthetic process							
BP	GO:0016242	negative regulation of macroautophagy	1/340	34/18614	0.4659903	0.6015884	0.4567848	HMOX1	1
BP	GO:0034121	regulation of toll-like receptor signaling pathway	1/340	34/18614	0.4659903	0.6015884	0.4567848	IRF1	1
BP	GO:0034383	low-density lipoprotein particle	1/340	34/18614	0.4659903	0.6015884	0.4567848	HMOX1	1

		clearance							
BP	GO:0042462	eye photoreceptor cell development	1/340	34/18614	0.4659903	0.6015884	0.4567848	NTRK2	1
BP	GO:0044546	NLRP3 inflammasome complex assembly	1/340	34/18614	0.4659903	0.6015884	0.4567848	GBP5	1
BP	GO:0045648	positive regulation of erythrocyte differentiation	1/340	34/18614	0.4659903	0.6015884	0.4567848	INHBA	1
BP	GO:00	response to pain	1/340	34/18614	0.465	0.601	0.456	AQP9	1

	48265				9903	5884	7848		
BP	GO:0060351	cartilage development involved in endochondral bone morphogenesis	1/340	34/18614	0.4659903	0.6015884	0.4567848	CBS	1
BP	GO:0060914	heart formation	1/340	34/18614	0.4659903	0.6015884	0.4567848	ROBO2	1
BP	GO:0090022	regulation of neutrophil chemotaxis	1/340	34/18614	0.4659903	0.6015884	0.4567848	DPP4	1
BP	GO:00	relaxation of muscle	1/340	34/18614	0.465	0.601	0.456	P2RY1	1

	90075				9903	5884	7848		
BP	GO:0098664	G protein-coupled serotonin receptor signaling pathway	1/340	34/18614	0.46599903	0.6015884	0.4567848	HRH2	1
BP	GO:0110110	positive regulation of animal organ morphogenesis	1/340	34/18614	0.46599903	0.6015884	0.4567848	ROBO2	1
BP	GO:0008154	actin polymerization or depoly	4/340	192/18614	0.46644428	0.6019687	0.4570736	CXCL12/PLEKHH2/FMN1/CSF3	4

		merization							
BP	GO:0006970	response to osmotic stress	2/340	86/18614	0.46774748	0.60283224	0.45772294	PTGS2/CAPN3	2
BP	GO:1900182	positive regulation of protein localization to nucleus	2/340	86/18614	0.46774748	0.60283224	0.45772294	PTGS2/PYHIN1	2
BP	GO:0034101	erythrocyte homeostasis	3/340	139/18614	0.4679033	0.60283224	0.45772294	HMOX1/ZFP36/INHBA	3
BP	GO:0042157	lipoprotein metabolic process	3/340	139/18614	0.4679033	0.60283224	0.45772294	PIGW/ZDHHC9/LIPG	3
BP	GO:00	negative regulati	3/340	139/18614	0.467	0.602	0.457	FRZB/EGR1/SHISA3	3

	90090	on of canonical Wnt signaling pathway			903	8324	7294		
BP	GO:0045787	positive regulation of cell cycle	7/340	354/18614	0.4694922	0.6046755	0.4591288	EIF4EBP1/TGFB2/PPP1R10/DDR2/IL1B/DTL/SLC6A4	7
BP	GO:0090092	regulation of transmembrane receptor protein serine/threonine kinase signaling	6/340	301/18614	0.4722026	0.6072103	0.4610535	BMP5/LTBP4/TGFB2/SULF1/INHBA/ASPN	6

		pathway							
BP	GO:0007093	mitotic cell cycle checkpoint signaling	3/340	140/18614	0.47259591	0.60721033	0.46105535	PPP1R10/IER3/DTL	3
BP	GO:0007189	adenylate cyclase-activating G protein-coupled receptor signaling pathway	3/340	140/18614	0.47259591	0.60721033	0.46105535	GPR4/CXCL10/CXCL9	3

BP	GO:0010507	negative regulation of autophagy	2/340	87/18614	0.47374486	0.6072103	0.46105535	HGF/HMOX1	2
BP	GO:0019319	hexose biosynthetic process	2/340	87/18614	0.47374486	0.6072103	0.46105535	SLC39A14/ATF3	2
BP	GO:0048145	regulation of fibroblast proliferation	2/340	87/18614	0.47374486	0.6072103	0.46105535	JUN/DDR2	2
BP	GO:0097194	execution phase of apoptosis	2/340	87/18614	0.47374486	0.6072103	0.46105535	NFKBIZ/IL6	2
BP	GO:20	positive regulation of	2/340	87/18614	0.473	0.607	0.461	P2RY1/INHBA	2

	00243	reproductive process			7486	2103	0535		
BP	GO:0051259	protein complex oligomerization	5/340	248/18614	0.475141446	0.6072103	0.4610535	SHMT2/MS4A1/NLRC4/CTH/GBP5	5
BP	GO:0006301	postreplication repair	1/340	35/18614	0.4757623	0.6072103	0.4610535	DTL	1
BP	GO:0010923	negative regulation of phosphatase activity	1/340	35/18614	0.4757623	0.6072103	0.4610535	TGFB2	1
BP	GO:0021988	olfactory lobe development	1/340	35/18614	0.4757623	0.6072103	0.4610535	ROBO2	1

BP	GO:0031128	developmental induction	1/340	35/18614	0.4757623	0.6072103	0.4610535	ROBO2	1
BP	GO:0032228	regulation of synaptic transmission, GABAergic	1/340	35/18614	0.4757623	0.6072103	0.4610535	CNR1	1
BP	GO:0035137	hindlimb morphogenesis	1/340	35/18614	0.4757623	0.6072103	0.4610535	FMN1	1
BP	GO:0043276	anoikis	1/340	35/18614	0.4757623	0.6072103	0.4610535	NTRK2	1
BP	GO:0045773	positive regulation of axon	1/340	35/18614	0.4757623	0.6072103	0.4610535	CXCL12	1

		extension							
BP	GO:0045879	negative regulation of smoothened signaling pathway	1/340	35/18614	0.47576623	0.6072103	0.46105535	CD3E	1
BP	GO:0048333	mesodermal cell differentiation	1/340	35/18614	0.47576623	0.6072103	0.46105535	INHBA	1
BP	GO:0050974	detection of mechanical stimulus involved in sensor	1/340	35/18614	0.47576623	0.6072103	0.46105535	CXCL12	1

		y perception							
BP	GO:0051354	negative regulation of oxidoreductase activity	1/340	35/18614	0.4757623	0.6072103	0.4610535	CNR1	1
BP	GO:0051443	positive regulation of ubiquitin-protein transferase activity	1/340	35/18614	0.4757623	0.6072103	0.4610535	TRIB3	1
BP	GO:0061081	positive regulation of myeloid leukocyte	1/340	35/18614	0.4757623	0.6072103	0.4610535	NR4A3	1

		cytokine production involved in immune response							
BP	GO:0071425	hematopoietic stem cell proliferation	1/340	35/18614	0.47576623	0.6072103	0.4610535	ACE	1
BP	GO:0097502	mannosylation	1/340	35/18614	0.47576623	0.6072103	0.4610535	TMTC1	1
BP	GO:1905898	positive regulation of response to endopl	1/340	35/18614	0.47576623	0.6072103	0.4610535	FCGR2B	1

		asmic reticulum stress							
BP	GO:2000352	negative regulation of endothelial cell apoptotic process	1/340	35/18614	0.47576623	0.60721030	0.46105535	FGA	1
BP	GO:0007269	neurotransmitter secretion	3/340	141/18614	0.47727763	0.6087355	0.46221 12	P2RY1/CSPG5/NR4A1	3
BP	GO:0099643	signal release from synapse	3/340	141/18614	0.47727763	0.6087355	0.46221 12	P2RY1/CSPG5/NR4A1	3

BP	GO:0031058	positive regulation of histone modification	2/340	88/18614	0.4797113	0.611227	0.4641033	SDR16C5/APLNR	2
BP	GO:0046849	bone remodeling	2/340	88/18614	0.4797113	0.611227	0.4641033	TNFRSF11B/IL6	2
BP	GO:0051453	regulation of intracellular pH	2/340	88/18614	0.4797113	0.611227	0.4641033	CFTR/SLC11A1	2
BP	GO:0006986	response to unfolded protein	3/340	142/18614	0.48193943	0.6138542	0.4660982	CTH/ATF3/ERP27	3
BP	GO:0044772	mitotic cell cycle phase transition	9/340	467/18614	0.48260053	0.6145034	0.4665912	EIF4EBP1/PIM2/WEE1/DDR2/IER3/BCAT1/DTL/INHBA/CCL2	9

BP	GO:1903829	positive regulation of protein localization	9/340	468/18614	0.48516 67	0.61547 92	0.46733 2	PTGS2/TGFB2/IL1B/SFN/PYHIN1/MYH10/ANO1/CFTR/FGA	9
BP	GO:0000460	maturation of 5.8S rRNA	1/340	36/18614	0.48535 59	0.61547 92	0.46733 2	WDR12	1
BP	GO:0002474	antigen processing and presentation of peptide antigen via MHC class I	1/340	36/18614	0.48535 59	0.61547 92	0.46733 2	ACE	1
BP	GO:0010613	positive regulation of cardiac muscle	1/340	36/18614	0.48535 59	0.61547 92	0.46733 2	NR4A3	1

BP	GO:0010984	hypertrophy regulation of lipoprotein particle clearance	1/340	36/18614	0.4853559	0.61547920	0.46733 2	LIPG	1
BP	GO:0033687	osteoblast proliferation	1/340	36/18614	0.48535 59	0.61547 92	0.46733 2	JUNB	1
BP	GO:0046473	phosphatidic acid metabolic process	1/340	36/18614	0.48535 59	0.61547 92	0.46733 2	PLA2G2A	1
BP	GO:0048730	epidermis morphogenesis	1/340	36/18614	0.48535 59	0.61547 92	0.46733 2	TGFB2	1

BP	GO:0050931	pigment cell differentiation	1/340	36/18614	0.48535 59	0.61547 92	0.46733 2	ADAMTS9	1
BP	GO:0090218	positive regulation of lipid kinase activity	1/340	36/18614	0.48535 59	0.61547 92	0.46733 2	FGR	1
BP	GO:0140632	inflammasome complex assembly	1/340	36/18614	0.48535 59	0.61547 92	0.46733 2	GBP5	1
BP	GO:1903725	regulation of phospholipid metabolic process	1/340	36/18614	0.48535 59	0.61547 92	0.46733 2	IDH1	1

BP	GO:0060191	regulation of lipase activity	2/340	89/18614	0.48563 26	0.61547 92	0.46733 2	CCL5/SELE	2
BP	GO:0071229	cellular response to acid chemical	2/340	89/18614	0.48563 26	0.61547 92	0.46733 2	GCLM/NTRK2	2
BP	GO:0002520	immune system development	4/340	197/18614	0.48627 08	0.61585 14	0.46761 46	NFKBIZ/ICOS/CD3E/IDO1	4
BP	GO:0030258	lipid modification	4/340	197/18614	0.48627 08	0.61585 14	0.46761 46	EPHX2/CNR1/NR4A3/ALOX15B	4
BP	GO:0010212	response to ionizing radiation	3/340	143/18614	0.48657 27	0.61585 14	0.46761 46	EGR1/VCAM1/CXCL10	3

BP	GO:1903900	regulation of viral life cycle	3/340	143/18614	0.48657 27	0.61585 14	0.46761 46	CCL5/CH25H/PTX3	3
BP	GO:0042176	regulation of protein catabolic process	7/340	361/18614	0.48996 12	0.61993 44	0.47071 48	TRIB3/TIMP4/NQO1/IER3/IL1B/DTL/TRIB1	7
BP	GO:0001941	postsynaptic membrane organization	1/340	37/18614	0.49477 46	0.62292 23	0.47298 36	SLC7A11	1
BP	GO:0010092	specification of animal organ identity	1/340	37/18614	0.49477 46	0.62292 23	0.47298 36	ROBO2	1
BP	GO:0010934	macrophage cytokine	1/340	37/18614	0.49477 46	0.62292 23	0.47298 36	TGFB2	1

		production							
BP	GO:0010935	regulation of macrophage cytokine production	1/340	37/18614	0.49477746	0.62292223	0.47298836	TGFB2	1
BP	GO:0014742	positive regulation of muscle hypertrophy	1/340	37/18614	0.49477746	0.62292223	0.47298836	NR4A3	1
BP	GO:0035883	enteroendocrine cell differentiation	1/340	37/18614	0.49477746	0.62292223	0.47298836	BMP5	1
BP	GO:0043516	regulation of DNA damage	1/340	37/18614	0.49477746	0.62292223	0.47298836	PYHIN1	1

		response, signal transduction by p53 class mediator							
BP	GO:0044273	sulfur compound catabolic process	1/340	37/18614	0.49477746	0.62292223	0.47298336	CBS	1
BP	GO:0046461	neutral lipid catabolic process	1/340	37/18614	0.49477746	0.62292223	0.47298336	PIK3CG	1
BP	GO:0046464	acylglycerol catabolic	1/340	37/18614	0.49477746	0.62292223	0.47298336	PIK3CG	1

		process							
BP	GO:0048536	spleen development	1/340	37/18614	0.49477746	0.62292223	0.47298836	NFKBIZ	1
BP	GO:0050654	chondroitin sulfate proteoglycan metabolic process	1/340	37/18614	0.49477746	0.62292223	0.47298836	DCN	1
BP	GO:0070306	lens fiber cell differentiation	1/340	37/18614	0.49477746	0.62292223	0.47298836	SLC7A11	1
BP	GO:0097345	mitochondrial outer membrane permea	1/340	37/18614	0.49477746	0.62292223	0.47298836	IER3	1

		bilization							
BP	GO:0120033	negative regulation of plasma membrane bounded cell projection assembly	1/340	37/18614	0.49477746	0.62292223	0.47298836	STAP1	1
BP	GO:0001942	hair follicle development	2/340	91/18614	0.49734488	0.62533303	0.47481119	TGFB2/INHBA	2
BP	GO:0042475	odontogenesis of dentin-containing tooth	2/340	91/18614	0.49734488	0.62533303	0.47481119	TNFRSF11B/CFTR	2

BP	GO:0072091	regulation of stem cell proliferation	2/340	91/18614	0.497343488	0.625333003	0.474748119	ACE/SFN	2
BP	GO:0072384	organelle transport along microtubule	2/340	91/18614	0.497343488	0.625333003	0.474748119	RASGRP1/SYBU	2
BP	GO:0018105	peptidyl-serine phosphorylation	6/340	309/18614	0.497505076	0.625333003	0.474748119	HGF/PTGS2/DDIT4/IL6/NTRK2/CSF3	6
BP	GO:0008654	phospholipid biosynthetic process	5/340	255/18614	0.49951517	0.62764649	0.47657 25	PIGW/IDH1/PIK3CG/ABCA8/SOCS3	5
BP	GO:00	positive regulation of	3/340	146/18614	0.500	0.627	0.476	PIM2/HMOX1/DCN	3

	10508	autophagy			366	8877	7538		
BP	GO:0035107	appendage morphogenesis	3/340	146/18614	0.500366	0.6278877	0.4767538	TGFB2/PKDCC/FMN1	3
BP	GO:0035108	limb morphogenesis	3/340	146/18614	0.500366	0.6278877	0.4767538	TGFB2/PKDCC/FMN1	3
BP	GO:0055123	digestive system development	3/340	146/18614	0.500366	0.6278877	0.4767538	TGFB2/PKDCC/RARRES2	3
BP	GO:0002028	regulation of sodium ion transport	2/340	92/18614	0.5031428	0.6295723	0.4780329	FXYD1/CNTN1	2
BP	GO:0045104	intermediate filament cytoske	2/340	92/18614	0.5031428	0.6295723	0.4780329	KRT7/KRT19	2

		leton organization							
BP	GO:0045666	positive regulation of neuron differentiation	2/340	92/18614	0.50314 28	0.62957 23	0.47803 29	CXCL12/DUOXA1	2
BP	GO:0055006	cardiac cell development	2/340	92/18614	0.50314 28	0.62957 23	0.47803 29	INHBA/MYH11	2
BP	GO:0001662	behavioral fear response	1/340	38/18614	0.50402 13	0.62957 23	0.47803 29	DPP4	1
BP	GO:0021955	central nervous system neuron axonogenesis	1/340	38/18614	0.50402 13	0.62957 23	0.47803 29	NR4A2	1

BP	GO:0030808	regulation of nucleotide biosynthetic process	1/340	38/18614	0.5040213	0.6295723	0.47803329	ENO1	1
BP	GO:0032885	regulation of polysaccharide biosynthetic process	1/340	38/18614	0.5040213	0.6295723	0.47803329	HAS2	1
BP	GO:0040036	regulation of fibroblast growth factor receptor signaling	1/340	38/18614	0.5040213	0.6295723	0.47803329	SULF1	1

		pathway								
BP	GO:0043001	Golgi to plasma membrane protein transport	1/340	38/18614	0.5040213	0.6295723	0.4780329	PKDCC		1
BP	GO:0046839	phospholipid dephosphorylation	1/340	38/18614	0.5040213	0.6295723	0.4780329	EPHX2		1
BP	GO:0120316	sperm flagellum assembly	1/340	38/18614	0.5040213	0.6295723	0.4780329	SPEF2		1
BP	GO:1900371	regulation of purine nucleotide biosynthetic	1/340	38/18614	0.5040213	0.6295723	0.4780329	ENO1		1

		process							
BP	GO:1902229	regulation of intrinsic apoptotic signaling pathway in response to DNA damage	1/340	38/18614	0.50402 0213	0.6295723	0.47803329	CXCL12	1
BP	GO:0030641	regulation of cellular pH	2/340	93/18614	0.5088937	0.6346184	0.4818644	CFTR/SLC11A1	2
BP	GO:0032436	positive regulation of proteasomal ubiquiti	2/340	93/18614	0.5088937	0.6346184	0.4818644	TRIB3/TRIB1	2

		n-dependent protein catabolic process							
BP	GO:0034644	cellular response to UV	2/340	93/18614	0.50888937	0.63461684	0.48186644	PTGS2/MFAP4	2
BP	GO:0045103	intermediate filament-based process	2/340	93/18614	0.50888937	0.63461684	0.48186644	KRT7/KRT19	2
BP	GO:0051781	positive regulation of cell division	2/340	93/18614	0.50888937	0.63461684	0.48186644	TGFB2/IL1B	2
BP	GO:00	detection of chemic	1/340	39/18614	0.513	0.637	0.484	AZGP1	1

	01580	al stimulus involved in sensory perception of bitter taste			0993	7564	2471		
BP	GO:0002209	behavioral defense response	1/340	39/18614	0.513093	0.637564	0.482471	DPP4	1
BP	GO:0002385	mucosal immune response	1/340	39/18614	0.513093	0.637564	0.482471	RNASE2	1
BP	GO:00	serotonin recepto	1/340	39/18614	0.513	0.637	0.484	HRH2	1

	07210	r signaling pathway			0993	7564	2471		
BP	GO:0042307	positive regulation of protein import into nucleus	1/340	39/18614	0.51309993	0.6377564	0.4842471	PTGS2	1
BP	GO:0043403	skeletal muscle tissue regeneration	1/340	39/18614	0.51309993	0.6377564	0.4842471	CAPN3	1
BP	GO:0044060	regulation of endocrine process	1/340	39/18614	0.51309993	0.6377564	0.4842471	INHBA	1
BP	GO:00	negative regulati	1/340	39/18614	0.513	0.637	0.484	SLC6A4	1

	46621	on of organ growth			0993	7564	2471		
BP	GO:0052652	cyclic purine nucleotide metabolic process	1/340	39/18614	0.5130993	0.6377564	0.4842471	PDE7B	1
BP	GO:0097242	amyloid-beta clearance	1/340	39/18614	0.5130993	0.6377564	0.4842471	MARCO	1
BP	GO:0014706	striated muscle tissue development	5/340	259/18614	0.5132508	0.6377564	0.4842471	RYR2/BMP5/TGFB2/ADAMTS9/MYH11	5
BP	GO:0016575	histone deacetylation	2/340	94/18614	0.5146009	0.6386011	0.4848885	SDR16C5/APLNR	2

BP	GO:0022404	molting cycle process	2/340	94/18614	0.514600 9	0.638601 1	0.484888 5	TGFB2/INHBA	2
BP	GO:0022405	hair cycle process	2/340	94/18614	0.514600 9	0.638601 1	0.484888 5	TGFB2/INHBA	2
BP	GO:0035082	axoneme assembly	2/340	94/18614	0.514600 9	0.638601 1	0.484888 5	SPEF2/DNAH5	2
BP	GO:0031644	regulation of nervous system process	3/340	150/18614	0.518460 2	0.642971 6	0.488207	HGF/EGR2/TNR	3
BP	GO:0046467	membrane lipid biosynthetic process	3/340	150/18614	0.518460 2	0.642971 6	0.488207	PIGW/ABCA8/ST6GALNAC3	3

BP	GO:0006029	proteoglycan metabolic process	2/340	95/18614	0.520202642	0.6444444	0.489293219	SULF1/DCN	2
BP	GO:0000184	nuclear-transcribed mRNA catabolic process, nonsense-mediated decay	1/340	40/18614	0.522010116	0.6444444	0.489293219	PNRC1	1
BP	GO:0000266	mitochondrial fission	1/340	40/18614	0.522010116	0.6444444	0.489293219	DCN	1

BP	GO:0000731	DNA synthesis involved in DNA repair	1/340	40/18614	0.52201 16	0.644444	0.48932 19	DTL	1
BP	GO:0009187	cyclic nucleotide metabolic process	1/340	40/18614	0.52201 16	0.644444	0.48932 19	PDE7B	1
BP	GO:0010762	regulation of fibroblast migration	1/340	40/18614	0.52201 16	0.644444	0.48932 19	DDR2	1
BP	GO:0010863	positive regulation of phospholipase	1/340	40/18614	0.52201 16	0.644444	0.48932 19	SELE	1

		C activity							
BP	GO:0018023	peptidyl-lysine trimethylation	1/340	40/18614	0.52201 16	0.644444	0.48932 19	NFKBIZ	1
BP	GO:0035136	forelimb morphogenesis	1/340	40/18614	0.52201 16	0.644444	0.48932 19	FMN1	1
BP	GO:0051125	regulation of actin nucleation	1/340	40/18614	0.52201 16	0.644444	0.48932 19	FMN1	1
BP	GO:0070286	axonemal dynein complex assembly	1/340	40/18614	0.52201 16	0.644444	0.48932 19	DNAH5	1
BP	GO:0099622	cardiac muscle cell membr	1/340	40/18614	0.52201 16	0.644444	0.48932 19	ANK2	1

		ane repolarization							
BP	GO:1900016	negative regulation of cytokine production involved in inflammatory response	1/340	40/18614	0.5220116	0.644444	0.4893219	IL1R2	1
BP	GO:1901532	regulation of hematopoietic progenitor cell differentiation	1/340	40/18614	0.5220116	0.644444	0.4893219	ZFP36	1

BP	GO:0044839	cell cycle G2/M phase transition	3/340	151/18614	0.522928 5	0.645363 29	0.490022 7	WEE1/IER3/DTL	3
BP	GO:0007264	small GTPase mediated signal transduction	9/340	483/18614	0.523177 5	0.645461 2	0.490097 3	TGFB2/PIK3CG/RHOBTB2/TAGAP/FGD2/HMOX1/GPR4/P2RY10/RASGRP1	9
BP	GO:0043502	regulation of muscle adaptation	2/340	96/18614	0.525883 3	0.647750 9	0.491835 9	GTF2IRD1/NR4A3	2
BP	GO:0044264	cellular polysaccharide metabolic process	2/340	96/18614	0.525883 3	0.647750 9	0.491835 9	HAS2/MGAM	2

BP	GO:0046364	monosaccharide biosynthetic process	2/340	96/18614	0.52588833	0.64775092	0.49183591	SLC39A14/ATF3	2
BP	GO:0048477	oogenesis	2/340	96/18614	0.52588833	0.64775092	0.49183591	RPS6KA2/PTX3	2
BP	GO:1903312	negative regulation of mRNA metabolic process	2/340	96/18614	0.52588833	0.64775092	0.49183591	ZFP36/SLC11A1	2
BP	GO:0010675	regulation of cellular carbohydrate metabo	3/340	152/18614	0.52737342	0.64916776	0.49291116	P2RY1/IER3/DDIT4	3

		lic process							
BP	GO:0016331	morphogenesis of embryonic epithelium	3/340	152/18614	0.5273742	0.6491676	0.4929116	BMP5/TGFB2/SULF1	3
BP	GO:0006643	membrane lipid metabolic process	4/340	208/18614	0.5287852	0.6506943	0.4940708	FUT3/PIGW/ABCA8/ST6GALNAC3	4
BP	GO:0044270	cellular nitrogen compound catabolic process	9/340	486/18614	0.5306724	0.6518631	0.4949583	PNRC1/BTG2/RNASE2/PDE7B/HMOX1/ZFP36/SLC11A1/IL6/IDO1	9

BP	GO:0014904	myotube cell development	1/340	41/18614	0.5307613	0.6518631	0.4949583	NFATC2	1
BP	GO:0030513	positive regulation of BMP signaling pathway	1/340	41/18614	0.5307613	0.6518631	0.4949583	SULF1	1
BP	GO:0032941	secretion by tissue	1/340	41/18614	0.5307613	0.6518631	0.4949583	ANO1	1
BP	GO:1901223	negative regulation of NIK/NF-kappaB signaling	1/340	41/18614	0.5307613	0.6518631	0.4949583	UACA	1

BP	GO:1901381	positive regulation of potassium ion transmembrane transport	1/340	41/18614	0.53076 13	0.65186 31	0.49495 83	ANK2	1
BP	GO:0001657	ureteric bud development	2/340	97/18614	0.53145 79	0.65187 84	0.49496 99	ROBO2/FMN1	2
BP	GO:0006497	protein lipidation	2/340	97/18614	0.53145 79	0.65187 84	0.49496 99	PIGW/ZDHHC9	2
BP	GO:0014020	primary neural tube formation	2/340	97/18614	0.53145 79	0.65187 84	0.49496 99	BMP5/TGFB2	2
BP	GO:00	synaptic vesicle	2/340	97/18614	0.531	0.651	0.494	P2RY1/CSPG5	2

	16079	exocytosis			4579	8784	9699		
BP	GO:0032368	regulation of lipid transport	3/340	153/18614	0.5317969	0.6520843	0.4951262	ABCA8/IL1B/LIPG	3
BP	GO:0046700	heterocycle catabolic process	9/340	488/18614	0.5356464	0.6565932	0.4985498	PNRC1/BTG2/RNASE2/PDE7B/HMOX1/ZFP36/SLC11A1/IL6/IDO1	9
BP	GO:0009411	response to UV	3/340	154/18614	0.5361964	0.6568449	0.4987409	PTGS2/MFAP4/DTL	3
BP	GO:0046488	phosphatidylinositol metabolic process	3/340	154/18614	0.5361964	0.6568449	0.4987409	PIGW/PIK3CG/SOCS3	3

BP	GO:0034620	cellular response to unfolded protein	2/340	98/18614	0.5369878	0.6569697	0.4988357	CTH/ATF3	2
BP	GO:0072163	mesonephric epithelium development	2/340	98/18614	0.5369878	0.6569697	0.4988357	ROBO2/FMN1	2
BP	GO:0072164	mesonephric tubule development	2/340	98/18614	0.5369878	0.6569697	0.4988357	ROBO2/FMN1	2
BP	GO:0120162	positive regulation of cold-induced thermogenesis	2/340	98/18614	0.5369878	0.6569697	0.4988357	ALPL/LCN2	2

BP	GO:0006040	amino sugar metabolic process	1/340	42/18614	0.5393512	0.6575392	0.4992681	CHIT1	1
BP	GO:0009124	nucleoside monophosphate biosynthetic process	1/340	42/18614	0.5393512	0.6575392	0.4992681	AMPD1	1
BP	GO:0009167	purine ribonucleoside monophosphate metabolic process	1/340	42/18614	0.5393512	0.6575392	0.4992681	AMPD1	1

BP	GO:0019320	hexose catabolic process	1/340	42/18614	0.53935 12	0.65753 92	0.49926 81	ENO1	1
BP	GO:0043551	regulation of phosphatidylinositol 3-kinase activity	1/340	42/18614	0.53935 12	0.65753 92	0.49926 81	FGR	1
BP	GO:0050691	regulation of defense response to virus by host	1/340	42/18614	0.53935 12	0.65753 92	0.49926 81	IL1B	1
BP	GO:0071354	cellular response to interleukin-6	1/340	42/18614	0.53935 12	0.65753 92	0.49926 81	IL6	1

BP	GO:0071542	dopaminergic neuron differentiation	1/340	42/18614	0.5393512	0.6575392	0.4992681	NR4A2	1
BP	GO:0086091	regulation of heart rate by cardiac conduction	1/340	42/18614	0.5393512	0.6575392	0.4992681	ANK2	1
BP	GO:0090207	regulation of triglyceride metabolic process	1/340	42/18614	0.5393512	0.6575392	0.4992681	PIK3CG	1
BP	GO:1900274	regulation of phospholipase C activity	1/340	42/18614	0.5393512	0.6575392	0.4992681	SELE	1

BP	GO:0060041	retina development in camera-type eye	3/340	155/18614	0.5405724	0.6586065	0.50007786	TGFB2/FAT3/NTRK2	3
BP	GO:0090316	positive regulation of intracellular protein transport	3/340	155/18614	0.5405724	0.6586065	0.50007786	PTGS2/IL1B/SFN	3
BP	GO:0003300	cardiac muscle hypertrophy	2/340	99/18614	0.54247727	0.65986671	0.50103357	RYR2/NR4A3	2
BP	GO:0010596	negative regulation of endothelial cell	2/340	99/18614	0.54247727	0.65986671	0.50103357	DCN/ADAMTS9	2

		migration							
BP	GO:0042632	cholesterol homeostasis	2/340	99/18614	0.542 4727	0.659 8671	0.501 0357	EPHX2/LIPG	2
BP	GO:0051588	regulation of neurotransmitter transport	2/340	99/18614	0.542 4727	0.659 8671	0.501 0357	P2RY1/CSPG5	2
BP	GO:1901992	positive regulation of mitotic cell cycle phase transition	2/340	99/18614	0.542 4727	0.659 8671	0.501 0357	DDR2/DTL	2
BP	GO:0099111	microtubule-based	4/340	212/18614	0.543 8184	0.661 2929	0.502 1183	SPEF2/RASGRP1/SYBU/DNAH5	4

		transport							
BP	GO:0007605	sensory perception of sound	3/340	156/18614	0.544492 46	0.662426 7	0.502979 2	PCDH15/PKHD1L1/SLC1A3	3
BP	GO:0006775	fat-soluble vitamin metabolic process	1/340	43/18614	0.547784 4	0.662464 6	0.503000 94	NQO1	1
BP	GO:0009154	purine ribonucleotide catabolic process	1/340	43/18614	0.547784 4	0.662464 6	0.503000 94	PDE7B	1
BP	GO:0016266	O-glycan processing	1/340	43/18614	0.547784 4	0.662464 6	0.503000 94	GXYLT2	1

BP	GO:0016601	Rac protein signal transduction	1/340	43/18614	0.54778444	0.66246664	0.50300094	PIK3CG	1
BP	GO:0019674	NAD metabolic process	1/340	43/18614	0.54778444	0.66246664	0.50300094	IDO1	1
BP	GO:0021879	forebrain neuron differentiation	1/340	43/18614	0.54778444	0.66246664	0.50300094	INHBA	1
BP	GO:0030501	positive regulation of bone mineralization	1/340	43/18614	0.54778444	0.66246664	0.50300094	PKDCC	1
BP	GO:0033574	response to testosterone	1/340	43/18614	0.54778444	0.66246664	0.50300094	NQO1	1

BP	GO:0043507	positive regulation of JUN kinase activity	1/340	43/18614	0.5477844	0.6624664	0.5030094	FGD2	1
BP	GO:0045494	photoreceptor cell maintenance	1/340	43/18614	0.5477844	0.6624664	0.5030094	PCDH15	1
BP	GO:0045663	positive regulation of myoblast differentiation	1/340	43/18614	0.5477844	0.6624664	0.5030094	CXCL9	1
BP	GO:0045687	positive regulation of glial cell differentiation	1/340	43/18614	0.5477844	0.6624664	0.5030094	EGR2	1

BP	GO:0045840	positive regulation of mitotic nuclear division	1/340	43/18614	0.54778 44	0.66246 64	0.50300 94	IL1B	1
BP	GO:0051954	positive regulation of amine transport	1/340	43/18614	0.54778 44	0.66246 64	0.50300 94	CXCL12	1
BP	GO:0097178	ruffle assembly	1/340	43/18614	0.54778 44	0.66246 64	0.50300 94	STAP1	1
BP	GO:1902110	positive regulation of mitochondrial membrane permeability involve	1/340	43/18614	0.54778 44	0.66246 64	0.50300 94	IER3	1

		d in apoptotic process							
BP	GO:0055092	sterol homeostasis	2/340	100/18614	0.54791 24	0.66246 64	0.50300 94	EPHX2/LIPG	2
BP	GO:0048592	eye morphogenesis	3/340	157/18614	0.54925 29	0.66387 66	0.50408 01	FAT3/COL8A1/NTRK2	3
BP	GO:0030100	regulation of endocytosis	4/340	214/18614	0.55124 24	0.66585 89	0.50558 53	SGIP1/CD177/STAP1/SELE	4
BP	GO:1902806	regulation of cell cycle G1/S phase transition	4/340	214/18614	0.55124 24	0.66585 89	0.50558 53	WEE1/DDR2/INHBA/CCL2	4

BP	GO:0010389	regulation of G2/M transition of mitotic cell cycle	2/340	101/18614	0.55333068	0.66766617	0.50695541	IER3/DTL	2
BP	GO:0043255	regulation of carbohydrate biosynthetic process	2/340	101/18614	0.55333068	0.66766617	0.50695541	P2RY1/HAS2	2
BP	GO:0048709	oligodendrocyte differentiation	2/340	101/18614	0.55333068	0.66766617	0.50695541	CNTN1/NTRK2	2
BP	GO:0006913	nucleocytoplasmic transport	6/340	328/18614	0.55578808	0.66766617	0.50695541	PTGS2/PPP1R10/EGR2/IER3/IL1B/SFN	6

BP	GO:0051169	nuclear transport	6/340	328/18614	0.5557808	0.6676617	0.5069541	PTGS2/PPP1R10/EGR2/IER3/IL1B/SFN	6
BP	GO:0001881	receptor recycling	1/340	44/18614	0.5560636	0.6676617	0.5069541	TRAT1	1
BP	GO:0003009	skeletal muscle contraction	1/340	44/18614	0.5560636	0.6676617	0.5069541	NR4A1	1
BP	GO:0009126	purine nucleoside monophosphate metabolic process	1/340	44/18614	0.5560636	0.6676617	0.5069541	AMPD1	1
BP	GO:0010828	positive regulation of glucose	1/340	44/18614	0.5560636	0.6676617	0.5069541	NR4A3	1

		transmembrane transport							
BP	GO:0021587	cerebellum morphogenesis	1/340	44/18614	0.556060636	0.667666617	0.506959541	CBS	1
BP	GO:0030835	negative regulation of actin filament depolymerization	1/340	44/18614	0.556060636	0.667666617	0.506959541	PLEKHH2	1
BP	GO:0032881	regulation of polysaccharide metabolic	1/340	44/18614	0.556060636	0.667666617	0.506959541	HAS2	1

		process							
BP	GO:0032924	activin receptor signaling pathway	1/340	44/18614	0.5560636	0.6676617	0.5069541	INHBA	1
BP	GO:0042596	fear response	1/340	44/18614	0.5560636	0.6676617	0.5069541	DPP4	1
BP	GO:0042771	intrinsic apoptotic signaling pathway in response to DNA damage by p53	1/340	44/18614	0.5560636	0.6676617	0.5069541	DDIT4	1

BP	GO:0044275	cellular carbohydrate catabolic process	1/340	44/18614	0.55606036	0.66766617	0.50695941	MGAM	1
BP	GO:0050856	regulation of T cell receptor signaling pathway	1/340	44/18614	0.55606036	0.66766617	0.50695941	TRAT1	1
BP	GO:0050913	sensory perception of bitter taste	1/340	44/18614	0.55606036	0.66766617	0.50695941	AZGP1	1

class mediator

BP	GO:1903573	negative regulation of response to endoplasmic reticulum stress	1/340	44/18614	0.55606036	0.66766617	0.50069541	HYOU1	1
BP	GO:0016579	protein deubiquitination	3/340	159/18614	0.5578367	0.6695797	0.50841405	SHMT2/STAMBPL1/OTUD1	3
BP	GO:0001823	mesonephros development	2/340	102/18614	0.55865556	0.66971189	0.50085161	ROBO2/FMN1	2
BP	GO:0014897	striated muscle hypertrophy	2/340	102/18614	0.55865556	0.66971189	0.50085161	RYR2/NR4A3	2
BP	GO:00	spinal cord	2/340	102/18614	0.558	0.669	0.508	PLXDC1/PHGDH	2

	21510	development			6556	7189	5161		
BP	GO:0042158	lipoprotein biosynthetic process	2/340	102/18614	0.5586556	0.6697189	0.5085161	PIGW/ZDHHC9	2
BP	GO:0007015	actin filament organization	8/340	442/18614	0.5603921	0.6715894	0.5099364	CXCL12/SERPINF2/DPYSL3/HMCN1/PLEKHH2/FMN1/CX3CL1/CSF3	8
BP	GO:0006665	sphingolipid metabolic process	3/340	160/18614	0.5620917	0.671917	0.5101852	FUT3/ABCA8/ST6GALNAC3	3
BP	GO:1905039	carboxylic acid transmembrane	3/340	160/18614	0.5620917	0.671917	0.5101852	SLC7A11/SLC1A3/SLC11A1	3

		transport							
BP	GO:0071805	potassium ion transmembrane transport	4/340	217/18614	0.5622585	0.671917	0.5101852	KCNAB1/ANK2/CASQ2/SLC1A3	4
BP	GO:0001776	leukocyte homeostasis	2/340	103/18614	0.5639587	0.671917	0.5101852	SLC7A11/IL6	2
BP	GO:0006885	regulation of pH	2/340	103/18614	0.5639587	0.671917	0.5101852	CFTR/SLC11A1	2
BP	GO:0048144	fibroblast proliferation	2/340	103/18614	0.5639587	0.671917	0.5101852	JUN/DDR2	2
BP	GO:0001825	blastocyst formation	1/340	45/18614	0.5641917	0.671917	0.5101852	JUNB	1

BP	GO:0007019	microtubule depolymerization	1/340	45/18614	0.5641917	0.6719917	0.5101852	NCKAP5	1
BP	GO:0010799	regulation of peptidyl-threonine phosphorylation	1/340	45/18614	0.5641917	0.6719917	0.5101852	DDIT4	1
BP	GO:0014003	oligodendrocyte development	1/340	45/18614	0.5641917	0.6719917	0.5101852	CNTN1	1
BP	GO:0030890	positive regulation of B cell proliferation	1/340	45/18614	0.5641917	0.6719917	0.5101852	NFATC2	1

BP	GO:0032781	positive regulation of ATP-dependent activity	1/340	45/18614	0.5641917	0.6719917	0.51018 52	RYR2	1
BP	GO:0033173	calcineurin-NFAT signaling cascade	1/340	45/18614	0.5641917	0.6719917	0.5101852	NFATC2	1
BP	GO:0050912	detection of chemical stimulus involved in sensory percept	1/340	45/18614	0.5641917	0.6719917	0.5101852	AZGP1	1

		ion of taste							
BP	GO:0061098	positive regulation of protein tyrosine kinase activity	1/340	45/18614	0.5641917	0.671917	0.5101852	ACE	1
BP	GO:0070741	response to interleukin-6	1/340	45/18614	0.5641917	0.671917	0.5101852	IL6	1
BP	GO:0097028	dendritic cell differentiation	1/340	45/18614	0.5641917	0.671917	0.5101852	FCGR2B	1
BP	GO:1902622	regulation of neutrophil migration	1/340	45/18614	0.5641917	0.671917	0.5101852	DPP4	1

BP	GO:1902686	mitochondrial outer membrane permeabilization involved in programmed cell death	1/340	45/18614	0.5641917	0.6719917	0.5101852	IER3	1
BP	GO:1904706	negative regulation of vascular associated smooth muscle cell	1/340	45/18614	0.5641917	0.6719917	0.5101852	HMOX1	1

		proliferation							
BP	GO:0034329	cell junction assembly	8/340	444/18614	0.5655314	0.6733021	0.5112369	ROBO2/ANK2/APLNR/ACE/FMN1/IL1B/LRRN3/NTRK2	8
BP	GO:0046434	organophosphate catabolic process	3/340	161/18614	0.5663322	0.6736121	0.5114722	IDH1/PDE7B/LIPG	3
BP	GO:0051017	actin filament bundle assembly	3/340	161/18614	0.5663322	0.6736121	0.5114722	SERPINF2/DPYSL3/CX3CL1	3
BP	GO:1903825	organic acid transmembrane transport	3/340	161/18614	0.5663322	0.6736121	0.5114722	SLC7A11/SLC1A3/SLC11A1	3

BP	GO:0014896	muscle hypertrophy	2/340	104/18614	0.56921 61	0.67599 95	0.51328 5	RYR2/NR4A3	2
BP	GO:0032479	regulation of type I interferon production	2/340	104/18614	0.56921 61	0.67599 95	0.51328 5	ZBTB20/IRF1	2
BP	GO:0032606	type I interferon production	2/340	104/18614	0.56921 61	0.67599 95	0.51328 5	ZBTB20/IRF1	2
BP	GO:0050773	regulation of dendrite development	2/340	104/18614	0.56921 61	0.67599 95	0.51328 5	BMP5/FAT3	2
BP	GO:0070252	actin-mediated cell	2/340	104/18614	0.56921 61	0.67599 95	0.51328 5	RYR2/ANK2	2

		contraction							
BP	GO:0046034	ATP metabolic process	4/340	219/18614	0.56952 06	0.67615 04	0.51339 96	ZBTB20/ENO1/IER3/DDIT4	4
BP	GO:0051258	protein polymerization	5/340	276/18614	0.56974 97	0.67621 18	0.51344 62	CASQ2/FMN1/TPPP3/CSF3/FGA	5
BP	GO:0007265	Ras protein signal transduction	6/340	333/18614	0.57060 05	0.67701 08	0.51405 29	TGFB2/PIK3CG/RHOBTB2/GPR4/P2RY10/RASGRP1	6
BP	GO:0034331	cell junction maintenance	1/340	46/18614	0.57217 13	0.67739 83	0.51434 71	CD177	1
BP	GO:0035305	negative regulation of dephos	1/340	46/18614	0.57217 13	0.67739 83	0.51434 71	TGFB2	1

		phorylation							
BP	GO:0042168	heme metabolic process	1/340	46/18614	0.57217 13	0.67739 83	0.51434 71	HMOX1	1
BP	GO:0042789	mRNA transcription by RNA polymerase II	1/340	46/18614	0.57217 13	0.67739 83	0.51434 71	NFKBIZ	1
BP	GO:0047496	vesicle transport along microtubule	1/340	46/18614	0.57217 13	0.67739 83	0.51434 71	RASGRP1	1
BP	GO:1990090	cellular response to nerve growth factor stimulus	1/340	46/18614	0.57217 13	0.67739 83	0.51434 71	NTRK2	1

BP	GO:2000273	positive regulation of signaling receptor activity	1/340	46/18614	0.57217 13	0.67739 83	0.51434 71	CCL2	1
BP	GO:0006457	protein folding	4/340	220/18614	0.57312 65	0.67831 84	0.51504 57	HYOU1/LTBP4/ERP27/FKBP5	4
BP	GO:0060562	epithelial tube morphogenesis	6/340	334/18614	0.57353 57	0.67859 2	0.51525 34	RYR2/BMP5/TGFB2/APLNR/FMN1/CXCL10	6
BP	GO:0001841	neural tube formation	2/340	105/18614	0.57442 75	0.67922 54	0.51573 44	BMP5/TGFB2	2
BP	GO:0006672	ceramide metabolic process	2/340	105/18614	0.57442 75	0.67922 54	0.51573 44	FUT3/ST6GALNAC3	2

BP	GO:0021915	neural tube development	3/340	163/18614	0.5747072	0.67934352	0.51582254	BMP5/TGFB2/PHGDH	3
BP	GO:0043583	ear development	4/340	221/18614	0.57671155	0.6815079	0.51746 75	FRZB/PCDH15/BMP5/TGFB2	4
BP	GO:0035966	response to topologically incorrect protein	3/340	164/18614	0.57886 18	0.68327 68	0.51881 06	CTH/ATF3/ERP27	3
BP	GO:0001754	eye photoreceptor cell differentiation	1/340	47/18614	0.58000 53	0.68327 68	0.51881 06	NTRK2	1
BP	GO:0009066	aspartate family amino acid	1/340	47/18614	0.58000 53	0.68327 68	0.51881 06	PHGDH	1

		metabolic process								
BP	GO:0021575	hindbrain morphogenesis	1/340	47/18614	0.5800053	0.6832768	0.5188106	CBS		1
BP	GO:0035196	miRNA processing	1/340	47/18614	0.5800053	0.6832768	0.5188106	IL6		1
BP	GO:0045601	regulation of endothelial cell differentiation	1/340	47/18614	0.5800053	0.6832768	0.5188106	IL1B		1
BP	GO:0048806	genitalia development	1/340	47/18614	0.5800053	0.6832768	0.5188106	BMP5		1
BP	GO:19	regulation of long-	1/340	47/18614	0.580	0.683	0.518	CX3CR1		1

	00271	term synaptic potentiation			0053	2768	8106		
BP	GO:1901385	regulation of voltage-gated calcium channel activity	1/340	47/18614	0.580053	0.682768	0.518106	GEM	1
BP	GO:1901976	regulation of cell cycle checkpoint	1/340	47/18614	0.580053	0.682768	0.518106	PPP1R10	1
BP	GO:0061572	actin filament bundle organization	3/340	165/18614	0.582909	0.686517	0.52132	SERPINF2/DPYSL3/CX3CL1	3

BP	GO:0006664	glycolipid metabolic process	2/340	107/18614	0.584 7125	0.688 1838	0.522 5365	PIGW/ST6GALNAC3	2
BP	GO:0022600	digestive system process	2/340	107/18614	0.584 7125	0.688 1838	0.522 5365	HRH2/SERPINA3	2
BP	GO:0051168	nuclear export	3/340	166/18614	0.587 0944	0.688 9298	0.523 1029	EGR2/IL1B/SFN	3
BP	GO:0008542	visual learning	1/340	48/18614	0.587 6963	0.688 9298	0.523 1029	SLC7A11	1
BP	GO:0035794	positive regulation of mitochondrial membrane	1/340	48/18614	0.587 6963	0.688 9298	0.523 1029	IER3	1

BP	GO:0035850	permeability epithelial cell differentiation involved in kidney development	1/340	48/18614	0.5876963	0.6889298	0.5231029	GPR4	1
BP	GO:0042269	regulation of natural killer cell mediated cytotoxicity	1/340	48/18614	0.5876963	0.6889298	0.5231029	RASGRP1	1
BP	GO:0042461	photoreceptor cell development	1/340	48/18614	0.5876963	0.6889298	0.5231029	NTRK2	1

BP	GO:0043268	positive regulation of potassium ion transport	1/340	48/18614	0.5876963	0.6889298	0.5231029	ANK2	1
BP	GO:0044331	cell-cell adhesion mediated by cadherin	1/340	48/18614	0.5876963	0.6889298	0.5231029	SERPINF2	1
BP	GO:0046717	acid secretion	1/340	48/18614	0.5876963	0.6889298	0.5231029	HRH2	1
BP	GO:0048066	developmental pigmentation	1/340	48/18614	0.5876963	0.6889298	0.5231029	ADAMTS9	1
BP	GO:00	activated T cell	1/340	48/18614	0.587	0.688	0.523	BTN3A1	1

	50798	proliferation			6963	9298	1029		
BP	GO:1902003	regulation of amyloid-beta formation	1/340	48/18614	0.5876963	0.6889298	0.5231029	NTRK2	1
BP	GO:1990089	response to nerve growth factor	1/340	48/18614	0.5876963	0.6889298	0.5231029	NTRK2	1
BP	GO:0032231	regulation of actin filament bundle assembly	2/340	108/18614	0.5897858	0.6909541	0.52464	SERPINF2/CX3CL1	2
BP	GO:1903509	liposaccharide metabolic	2/340	108/18614	0.5897858	0.6909541	0.52464	PIGW/ST6GALNAC3	2

		process							
BP	GO:0006486	protein glycosylation	4/340	226/18614	0.5944017	0.6941511	0.5270674	FUT3/GXYLT2/ST6GALNAC3/TMTC1	4
BP	GO:0043413	macromolecule glycosylation	4/340	226/18614	0.5944017	0.6941511	0.5270674	FUT3/GXYLT2/ST6GALNAC3/TMTC1	4
BP	GO:0007218	neuropeptide signaling pathway	2/340	109/18614	0.5948131	0.6941511	0.5270674	CYSLTR1/CPE	2
BP	GO:0019395	fatty acid oxidation	2/340	109/18614	0.5948131	0.6941511	0.5270674	CNR1/NR4A3	2
BP	GO:0033209	tumor necrosis factor-mediat	2/340	109/18614	0.5948131	0.6941511	0.5270674	NAIP/PLVAP	2

		ed signaling pathway							
BP	GO:0007528	neuromuscular junction development	1/340	49/18614	0.5952468	0.6941511	0.5270674	TNC	1
BP	GO:0009261	ribonucleotide catabolic process	1/340	49/18614	0.5952468	0.6941511	0.5270674	PDE7B	1
BP	GO:0010665	regulation of cardiac muscle cell apoptotic process	1/340	49/18614	0.5952468	0.6941511	0.5270674	PPP1R10	1

BP	GO:0032892	positive regulation of organic acid transport	1/340	49/18614	0.59524 68	0.69415 11	0.52706 74	IL1B	1
BP	GO:0034198	cellular response to amino acid starvation	1/340	49/18614	0.59524 68	0.69415 11	0.52706 74	ATF3	1
BP	GO:0042149	cellular response to glucose starvation	1/340	49/18614	0.59524 68	0.69415 11	0.52706 74	PLIN2	1
BP	GO:0043330	response to exogenous dsRNA	1/340	49/18614	0.59524 68	0.69415 11	0.52706 74	RFTN2	1

BP	GO:0046580	negative regulation of Ras protein signal transduction	1/340	49/18614	0.59524 68	0.69415 11	0.52706 74	TGFB2	1
BP	GO:0097720	calcineurin-mediated signaling	1/340	49/18614	0.59524 68	0.69415 11	0.52706 74	NFATC2	1
BP	GO:1902108	regulation of mitochondrial membrane permeability involved in apoptot	1/340	49/18614	0.59524 68	0.69415 11	0.52706 74	IER3	1

		ic process							
BP	GO:0072659	protein localization to plasma membrane	5/340	285/18614	0.5982259	0.6974503	0.5295725	P2RY1/ANK2/PIGW/PKDCC/SKAP1	5
BP	GO:0030148	sphingolipid biosynthetic process	2/340	110/18614	0.5997942	0.6990262	0.5307691	ABCA8/ST6GALNAC3	2
BP	GO:0009100	glycoprotein metabolic process	7/340	401/18614	0.6012121	0.7004645	0.5318612	FUT3/GXYLT2/ST6GALNAC3/SULF1/DCN/TMTC1/PTX3	7
BP	GO:0043457	regulation of cellular respiration	1/340	50/18614	0.6026595	0.7008652	0.5321655	SHMT2	1

BP	GO:0045581	negative regulation of T cell differentiation	1/340	50/18614	0.60265 95	0.70086 52	0.53216 55	IRF1	1
BP	GO:0046365	monosaccharide catabolic process	1/340	50/18614	0.60265 95	0.70086 52	0.53216 55	ENO1	1
BP	GO:0048009	insulin-like growth factor receptor signaling pathway	1/340	50/18614	0.60265 95	0.70086 52	0.53216 55	BMP5	1

BP	GO:0090102	cochlea development	1/340	50/18614	0.6026595	0.7008652	0.5321655	FRZB	1
BP	GO:2000378	negative regulation of reactive oxygen species metabolic process	1/340	50/18614	0.6026595	0.7008652	0.53216555	SLC18A2	1
BP	GO:0051341	regulation of oxidoreductase activity	2/340	111/18614	0.60472 92	0.70305 77	0.53383 02	CNR1/IL1B	2
BP	GO:0090287	regulation of cellular respon	6/340	345/18614	0.60515 21	0.70333 47	0.53404 05	LTBP4/IL1B/SULF1/DCN/SEMA6A/ASPN	6

		se to growth factor stimulus							
BP	GO:0042177	negative regulation of protein catabolic process	2/340	112/18614	0.60961681	0.70717704	0.536953	TIMP4/NQO1	2
BP	GO:1902749	regulation of cell cycle G2/M phase transition	2/340	112/18614	0.60961681	0.70717704	0.536953	IER3/DTL	2
BP	GO:0009299	mRNA transcription	1/340	51/18614	0.60993368	0.70717704	0.536953	NFKBIZ	1

BP	GO:0010662	regulation of striated muscle cell apoptotic process	1/340	51/18614	0.60993 68	0.70717 04	0.53695 3	PPP1R10	1
BP	GO:0010939	regulation of necrotic cell death	1/340	51/18614	0.60993 68	0.70717 04	0.53695 3	DDR2	1
BP	GO:0045778	positive regulation of ossification	1/340	51/18614	0.60993 68	0.70717 04	0.53695 3	PKDCC	1
BP	GO:0051489	regulation of filopodium assembly	1/340	51/18614	0.60993 68	0.70717 04	0.53695 3	DPYSL3	1

BP	GO:0051646	mitochondrion localization	1/340	51/18614	0.60993683	0.70717040	0.5369533	SYBU	1
BP	GO:2000058	regulation of ubiquitin-dependent protein catabolic process	3/340	173/18614	0.61508879	0.71292585	0.5413233	TRIB3/PYHIN1/TRIB1	3
BP	GO:0006195	purine nucleotide catabolic process	1/340	52/18614	0.61708812	0.71393934	0.54208881	PDE7B	1
BP	GO:0007215	glutamate receptor	1/340	52/18614	0.61708812	0.71393934	0.54208881	CX3CL1	1

		signaling pathway							
BP	GO:0021872	forebrain generation of neurons	1/340	52/18614	0.6170812	0.7139334	0.5420881	INHBA	1
BP	GO:0048599	oocyte development	1/340	52/18614	0.6170812	0.7139334	0.5420881	RPS6KA2	1
BP	GO:0051496	positive regulation of stress fiber assembly	1/340	52/18614	0.6170812	0.7139334	0.5420881	SERPINF2	1
BP	GO:1990928	response to amino acid	1/340	52/18614	0.6170812	0.7139334	0.5420881	ATF3	1

		starvation							
BP	GO:0030178	negative regulation of Wnt signaling pathway	3/340	174/18614	0.61898913	0.71591443	0.54359 22	FRZB/EGR1/SHISA3	3
BP	GO:1901136	carbohydrate derivative catabolic process	3/340	175/18614	0.62284 79	0.71942 73	0.54625 96	PDE7B/LYVE1/CHIT1	3
BP	GO:0048259	regulation of receptor-mediated	2/340	115/18614	0.62400 86	0.71942 73	0.54625 96	SGIP1/SELE	2

		endocytosis							
BP	GO:0051261	protein depolymerization	2/340	115/18614	0.624000086	0.71942273	0.54625596	NCKAP5/PLEKHH2	2
BP	GO:0002639	positive regulation of immunoglobulin production	1/340	53/18614	0.6240951	0.71942273	0.54625596	IL6	1
BP	GO:0006778	porphyrin-containing compound metabolic process	1/340	53/18614	0.6240951	0.71942273	0.54625596	HMOX1	1

BP	GO:0010874	regulation of cholesterol efflux	1/340	53/18614	0.62409 51	0.71942 73	0.54625 96	ABCA8	1
BP	GO:0030834	regulation of actin filament depolymerization	1/340	53/18614	0.62409 51	0.71942 73	0.54625 96	PLEKHH2	1
BP	GO:0035065	regulation of histone acetylation	1/340	53/18614	0.62409 51	0.71942 73	0.54625 96	SDR16C5	1
BP	GO:0050873	brown fat cell differentiation	1/340	53/18614	0.62409 51	0.71942 73	0.54625 96	PTGS2	1
BP	GO:0050982	detection of mechanical	1/340	53/18614	0.62409 51	0.71942 73	0.54625 96	CXCL12	1

		stimulus							
BP	GO:1905710	positive regulation of membrane permeability	1/340	53/18614	0.6240951	0.7194273	0.5462596	IER3	1
BP	GO:0006476	protein deacetylation	2/340	116/18614	0.6287136	0.7238616	0.5496266	SDR16C5/APLNR	2
BP	GO:0010633	negative regulation of epithelial cell migration	2/340	116/18614	0.6287136	0.7238616	0.5496266	DCN/ADAMTS9	2
BP	GO:0042752	regulation of circadian rhythm	2/340	116/18614	0.6287136	0.7238616	0.5496266	PTGDS/NR1D2	2

BP	GO:0010469	regulation of signaling receptor activity	3/340	177/18614	0.630508	0.723816	0.549626	P2RY1/FCGR2B/CCL2	3
BP	GO:0022900	electron transport chain	3/340	177/18614	0.630508	0.723816	0.549626	PHGDH/NQO2/IDO1	3
BP	GO:0070646	protein modification by small protein removal	3/340	177/18614	0.630508	0.723816	0.549626	SHMT2/STAMBPL1/OTUD1	3
BP	GO:0002204	somatic recombination of immunoglobulin	1/340	54/18614	0.630989	0.723816	0.549626	NFKBIZ	1

		genes involved in immune response							
BP	GO:0002208	somatic diversification of immunoglobulins involved in immune response	1/340	54/18614	0.63098809	0.72386816	0.54962266	NFKBIZ	1
BP	GO:0006383	transcription by RNA polymerase III	1/340	54/18614	0.63098809	0.72386816	0.54962266	INHBA	1

BP	GO:0007632	visual behavior	1/340	54/18614	0.63098 09	0.72386 16	0.54962 66	SLC7A11	1
BP	GO:0030857	negative regulation of epithelial cell differentiation	1/340	54/18614	0.63098 09	0.72386 16	0.54962 66	FRZB	1
BP	GO:0032330	regulation of chondrocyte differentiation	1/340	54/18614	0.63098 09	0.72386 16	0.54962 66	PKDCC	1
BP	GO:0043550	regulation of lipid kinase activity	1/340	54/18614	0.63098 09	0.72386 16	0.54962 66	FGR	1
BP	GO:00	isotype switching	1/340	54/18614	0.630	0.723	0.549	NFKBIZ	1

	45190				9809	8616	6266		
BP	GO:0051452	intracellular pH reduction	1/340	54/18614	0.630989809	0.723868616	0.549626266	SLC11A1	1
BP	GO:0098703	calcium ion import across plasma membrane	1/340	54/18614	0.630989809	0.723868616	0.549626266	MS4A1	1
BP	GO:1901800	positive regulation of proteasomal protein catabolic process	2/340	117/18614	0.633333727	0.726383868	0.55151544	TRIB3/TRIB1	2
BP	GO:00	striated muscle	1/340	55/18614	0.637	0.729	0.554	GTF2IRD1	1

	148<br>88	adaptat<br>ion			74<br>1	88<br>25	19<br>83			
B<br>P	GO<br>:00<br>315<br>29	ruffle<br>organiz<br>ation	1/<br>34<br>0	55/<br>186<br>14	0.6<br>37<br>74<br>1	0.7<br>29<br>88<br>25	0.5<br>54<br>19<br>83	STAP1		1
B<br>P	GO<br>:00<br>436<br>47	inositol<br>phosph<br>ate<br>metabo<br>lic<br>proces<br>s	1/<br>34<br>0	55/<br>186<br>14	0.6<br>37<br>74<br>1	0.7<br>29<br>88<br>25	0.5<br>54<br>19<br>83	P2RY1		1
B<br>P	GO<br>:00<br>723<br>48	sulfur<br>compo<br>und<br>transpo<br>rt	1/<br>34<br>0	55/<br>186<br>14	0.6<br>37<br>74<br>1	0.7<br>29<br>88<br>25	0.5<br>54<br>19<br>83	SLC7A11		1
B<br>P	GO<br>:19<br>000<br>87	positive<br>regulati<br>on of<br>G1/S<br>transiti<br>on of<br>mitotic	1/<br>34<br>0	55/<br>186<br>14	0.6<br>37<br>74<br>1	0.7<br>29<br>88<br>25	0.5<br>54<br>19<br>83	DDR2		1

BP	GO:1902991	cell cycle regulation of amyloid precursor protein catabolic process	1/340	55/18614	0.637741	0.7298825	0.5541983	NTRK2		1
BP	GO:0002065	columnar/cuboidal epithelial cell differentiation	2/340	118/18614	0.639861	0.7298825	0.5541983	BMP5/FASN		2
BP	GO:0035967	cellular response to topologically incorre	2/340	118/18614	0.639861	0.7298825	0.5541983	CTH/ATF3		2

		ct							
BP	GO:1901990	protein regulation of mitotic cell cycle phase transition	6/340	357/18614	0.6381445	0.7298825	0.5541983	WEE1/DDR2/IER3/DTL/INHBA/CCL2	6
BP	GO:0035265	organ growth	3/340	180/18614	0.6417782	0.7338184	0.5571867	TGFB2/DDR2/SLC6A4	3
BP	GO:0045446	endothelial cell differentiation	2/340	119/18614	0.6425554	0.7344851	0.5576929	FASN/IL1B	2
BP	GO:0002823	negative regulation of adaptive immun	1/340	56/18614	0.6443776	0.7345866	0.55777	FCGR2B	1

		e response based on somatic recombination of immune receptors built from immunoglobulin superfamily domains							
BP	GO:0009994	oocyte differentiation	1/340	56/18614	0.644376	0.734586	0.55777	RPS6KA2	1

BP	GO:0010803	regulation of tumor necrosis factor-mediated signaling pathway	1/340	56/18614	0.644376	0.734866	0.55777	NAIP	1
BP	GO:0034205	amyloid-beta formation	1/340	56/18614	0.644376	0.734866	0.55777	NTRK2	1
BP	GO:0042908	xenobiotic transport	1/340	56/18614	0.644376	0.734866	0.55777	ABCA8	1
BP	GO:0043331	response to dsRNA	1/340	56/18614	0.644376	0.734866	0.55777	RFTN2	1

BP	GO:0045071	negative regulation of viral genome replication	1/340	56/18614	0.64437 76	0.73458 66	0.55777	CCL5	1
BP	GO:0051058	negative regulation of small GTPase mediated signal transduction	1/340	56/18614	0.64437 76	0.73458 66	0.55777	TGFB2	1
BP	GO:0065002	intracellular protein transmembran	1/340	56/18614	0.64437 76	0.73458 66	0.55777	HYOU1	1

		e transport							
BP	GO:0035725	sodium ion transmembrane transport	3/340	181/18614	0.645744832	0.73562627	0.5585856	SCNN1G/FXYD1/SLC6A4	3
BP	GO:0010676	positive regulation of cellular carbohydrate metabolic process	1/340	57/18614	0.65089929	0.74002017	0.56189969	P2RY1	1
BP	GO:0043388	positive regulation of DNA binding	1/340	57/18614	0.65089929	0.74002017	0.56189969	PYHIN1	1

BP	GO:0045010	actin nucleation	1/340	57/18614	0.65089 29	0.74002 17	0.56189 69	FMN1		1
BP	GO:0048016	inositol phosphate-mediated signaling	1/340	57/18614	0.65089 29	0.74002 17	0.56189 69	NFATC2		1
BP	GO:0048168	regulation of neuronal synaptic plasticity	1/340	57/18614	0.65089 29	0.74002 17	0.56189 69	EGR2		1
BP	GO:0050881	musculoskeletal movement	1/340	57/18614	0.65089 29	0.74002 17	0.56189 69	NR4A1		1

BP	GO:0051932	synaptic transmission, GABAergic	1/340	57/18614	0.65089 29	0.74002 17	0.56189 69	CNR1	1
BP	GO:0072523	purine-containing compound catabolic process	1/340	57/18614	0.65089 29	0.74002 17	0.56189 69	PDE7B	1
BP	GO:0072089	stem cell proliferation	2/340	121/18614	0.65155 34	0.74055 17	0.56229 93	ACE/SFN	2
BP	GO:0009205	purine ribonucleoside triphosphate metabolic	4/340	244/18614	0.65431 03	0.74346 35	0.56451 02	ZBTB20/ENO1/IER3/DDIT4	4

		process								
BP	GO:0034249	negative regulation of amide metabolic process	5/340	304/18614	0.6548075	0.7438067	0.5647708	BTG2/SHMT2/EIF4EBP1/ZFP36/NTRK2		5
BP	GO:0021543	pallium development	3/340	184/18614	0.6564357	0.7448209	0.5655409	BTG2/TACC2/NTRK2		3
BP	GO:0006893	Golgi to plasma membrane transport	1/340	58/18614	0.6572892	0.7448209	0.5655409	PKDCC		1
BP	GO:0030042	actin filament depoly	1/340	58/18614	0.6572892	0.7448209	0.5655409	PLEKHH2		1

		merization							
BP	GO:0042306	regulation of protein import into nucleus	1/340	58/18614	0.6572892	0.7448209	0.5655409	PTGS2	1
BP	GO:0050732	negative regulation of peptidyl-tyrosine phosphorylation	1/340	58/18614	0.6572892	0.7448209	0.5655409	SOCS3	1
BP	GO:0050879	multicellular organismal movement	1/340	58/18614	0.6572892	0.7448209	0.5655409	NR4A1	1

BP	GO:0061005	cell differentiation involved in kidney development	1/340	58/18614	0.657289 2	0.744820 9	0.565540 9	GPR4	1
BP	GO:2000649	regulation of sodium ion transmembrane transporter activity	1/340	58/18614	0.657289 2	0.744820 9	0.565540 9	FXYD1	1
BP	GO:0070085	glycosylation	4/340	245/18614	0.657459 3	0.744820 9	0.565540 9	FUT3/GXYLT2/ST6GALNAC3/TMTC1	4
BP	GO:0006413	translational initiation	2/340	123/18614	0.660371 9	0.747454	0.567540 2	EIF4EBP1/CCL5	2

BP	GO:0061157	mRNA destabilization	2/340	123/18614	0.660 3719	0.747 454	0.567 5402	BTG2/ZFP36	2
BP	GO:0071901	negative regulation of protein serine/threonine kinase activity	2/340	123/18614	0.660 3719	0.747 454	0.567 5402	LAX1/IL1B	2
BP	GO:0009161	ribonucleoside monophosphate metabolic process	1/340	59/18614	0.663 5687	0.750 181	0.569 6109	AMPD1	1
BP	GO:00	regulation of	1/340	59/18614	0.663	0.750	0.569	ACE	1

	46324	glucose import			5687	181	6109		
BP	GO:0051438	regulation of ubiquitin-protein transferase activity	1/340	59/18614	0.6635687	0.750181	0.5696109	TRIB3	1
BP	GO:0051785	positive regulation of nuclear division	1/340	59/18614	0.6635687	0.750181	0.5696109	IL1B	1
BP	GO:0006006	glucose metabolic process	3/340	187/18614	0.6671437	0.7537755	0.5723401	ENO1/SLC39A14/ATF3	3
BP	GO:0051302	regulation of cell division	3/340	187/18614	0.6671437	0.7537755	0.5723401	TGFB2/IL1B/SFN	3

BP	GO:0002011	morphogenesis of an epithelial sheet	1/340	60/18614	0.66973734	0.75595505	0.57399916	BMP5	1
BP	GO:0015695	organic cation transport	1/340	60/18614	0.66973734	0.75595505	0.57399916	P2RY1	1
BP	GO:0032007	negative regulation of TOR signaling	1/340	60/18614	0.66973734	0.75595505	0.57399916	DDIT4	1
BP	GO:0009144	purine nucleoside triphosphate metabolic process	4/340	249/18614	0.66988622	0.75595505	0.57399916	ZBTB20/ENO1/IER3/DDIT4	4

BP	GO:0000075	cell cycle checkpoint signaling	3/340	188/18614	0.670656587	0.756404014	0.574334	PPP1R10/IER3/DTL	3
BP	GO:0061138	morphogenesis of a branching epithelium	3/340	188/18614	0.670656587	0.756404014	0.574334	HGF/SULF1/SOCS3	3
BP	GO:0001838	embryonic epithelial tube formation	2/340	126/18614	0.673262636	0.758890202	0.576222237	BMP5/TGFB2	2
BP	GO:0021987	cerebral cortex development	2/340	126/18614	0.673262636	0.758890202	0.576222237	TACC2/NTRK2	2
BP	GO:00	negative regulati	1/340	61/18614	0.675	0.761	0.578	FCGR2B	1

	02820	on of adaptive immune response			7855	2396	0076		
BP	GO:0060038	cardiac muscle cell proliferation	1/340	61/18614	0.6757855	0.7612396	0.5780076	TGFB2	1
BP	GO:0009199	ribonucleoside triphosphate metabolic process	4/340	251/18614	0.6759472	0.7612396	0.5780076	ZBTB20/ENO1/IER3/DDIT4	4
BP	GO:0031109	microtubule polymerization or depoly	2/340	127/18614	0.6774719	0.7618311	0.5784567	NCKAP5/TPPP3	2

		merization							
BP	GO:0035601	protein deacylation	2/340	127/18614	0.67747719	0.76183111	0.5784567	SDR16C5/APLNR	2
BP	GO:0050779	RNA destabilization	2/340	127/18614	0.67747719	0.76183111	0.5784567	BTG2/ZFP36	2
BP	GO:0061014	positive regulation of mRNA catabolic process	2/340	127/18614	0.67747719	0.76183111	0.5784567	BTG2/ZFP36	2
BP	GO:0071482	cellular response to light stimulus	2/340	127/18614	0.67747719	0.76183111	0.5784567	PTGS2/MFAP4	2

BP	GO:0032271	regulation of protein polymerization	3/340	191/18614	0.6810405	0.7641358	0.5802067	FMN1/TPPP3/CSF3	3
BP	GO:0071478	cellular response to radiation	3/340	191/18614	0.6810405	0.7641358	0.5802067	PTGS2/EGR1/MFAP4	3
BP	GO:0006687	glycosphingolipid metabolic process	1/340	62/18614	0.681727	0.7641358	0.5802067	ST6GALNAC3	1
BP	GO:0016447	somatic recombination of immunoglobulin gene segments	1/340	62/18614	0.681727	0.7641358	0.5802067	NFKBIZ	1

BP	GO:0033013	tetrapyrrole metabolic process	1/340	62/18614	0.681727	0.7641358	0.5802067	HMOX1	1
BP	GO:0046173	polyol biosynthetic process	1/340	62/18614	0.681727	0.7641358	0.5802067	P2RY1	1
BP	GO:0046530	photoreceptor cell differentiation	1/340	62/18614	0.681727	0.7641358	0.5802067	NTRK2	1
BP	GO:0046902	regulation of mitochondrial membrane permeability	1/340	62/18614	0.681727	0.7641358	0.5802067	IER3	1

BP	GO:0061951	establishment of protein localization to plasma membrane	1/340	62/18614	0.681727	0.7641358	0.5802067	PKDCC	1
BP	GO:1903018	regulation of glycoprotein metabolic process	1/340	62/18614	0.681727	0.7641358	0.5802067	PTX3	1
BP	GO:2000756	regulation of peptidyl-lysine acetylation	1/340	62/18614	0.681727	0.7641358	0.5802067	SDR16C5	1
BP	GO:00	peptidyl-lysine	6/340	374/18614	0.681	0.764	0.580	EGR1/SDR16C5/EGR2/NFKBIZ/CAPN3/CTH	6

	18205	modification			0.987 5	0.203	0.2577		
BP	GO:0006814	sodium ion transport	4/340	254/18614	0.6849287	0.7672731	0.5825889	SCNN1G/FXYD1/CNTN1/SLC6A4	4
BP	GO:0007368	determination of left/right symmetry	2/340	129/18614	0.6857562	0.7679634	0.583113	APLNR/DNAH5	2
BP	GO:1902903	regulation of supramolecular fiber organization	6/340	376/18614	0.6869128	0.7679634	0.583113	CXCL12/SERPINF2/PLEKHH2/FMN1/CX3CL1/CSF3	6
BP	GO:0002090	regulation of receptor internalization	1/340	63/18614	0.68756	0.7679634	0.583113	SELE	1

BP	GO:0002712	regulation of B cell mediated immunity	1/340	63/18614	0.68756	0.7679634	0.5831113	FCGR2B	1
BP	GO:0002889	regulation of immunoglobulin mediated immune response	1/340	63/18614	0.68756	0.7679634	0.5831113	FCGR2B	1
BP	GO:0033692	cellular polysaccharide biosynthetic process	1/340	63/18614	0.68756	0.7679634	0.5831113	HAS2	1

BP	GO:0051353	positive regulation of oxidoreductase activity	1/340	63/18614	0.68756	0.7679634	0.583113	IL1B	1
BP	GO:0051784	negative regulation of nuclear division	1/340	63/18614	0.68756	0.7679634	0.583113	RPS6KA2	1
BP	GO:0099601	regulation of neurotransmitter receptor activity	1/340	63/18614	0.68756	0.7679634	0.583113	CCL2	1
BP	GO:2001244	positive regulation of intrinsic apoptot	1/340	63/18614	0.68756	0.7679634	0.583113	S100A8	1

		ic signaling pathway							
BP	GO:0048839	inner ear development	3/340	193/18614	0.6878826	0.7680355	0.5831677	FRZB/PCDH15/TGFB2	3
BP	GO:0030218	erythrocyte differentiation	2/340	130/18614	0.68983827	0.7698825	0.58452265	ZFP36/INHBA	2
BP	GO:0030833	regulation of actin filament polymerization	2/340	130/18614	0.68983827	0.7698825	0.58452265	FMN1/CSF3	2
BP	GO:0045664	regulation of neuron differentiation	3/340	194/18614	0.69117782	0.77093993	0.58537726	CXCL12/DUOXA1/SLC6A4	3

BP	GO:0006940	regulation of smooth muscle contraction	1/340	64/18614	0.6932863	0.7709393	0.58537726	PTGS2	1
BP	GO:0044458	motile cilium assembly	1/340	64/18614	0.6932863	0.7709393	0.58537726	SPEF2	1
BP	GO:0046503	glycerolipid catabolic process	1/340	64/18614	0.6932863	0.7709393	0.58537726	PIK3CG	1
BP	GO:0048524	positive regulation of viral process	1/340	64/18614	0.6932863	0.77093993	0.58537726	CCL5	1
BP	GO:0051445	regulation of meiotic	1/340	64/18614	0.6932863	0.77093993	0.58537726	RPS6KA2	1

BP	GO:0051568	cell cycle histone H3-K4 methylation	1/340	64/18614	0.6932863	0.77093993	0.5853726	NFKBIZ	1
BP	GO:0051893	regulation of focal adhesion assembly	1/340	64/18614	0.6932863	0.77093993	0.5853726	FMN1	1
BP	GO:0071300	cellular response to retinoic acid	1/340	64/18614	0.6932863	0.77093993	0.5853726	SLC6A4	1
BP	GO:0090109	regulation of cell-substrate junction assembly	1/340	64/18614	0.6932863	0.77093993	0.5853726	FMN1	1

BP	GO:1905953	negative regulation of lipid localization	1/340	64/18614	0.6932863	0.77093	0.5853726	IL6	1
BP	GO:0030317	flagellated sperm motility	2/340	131/18614	0.69388656	0.77093	0.5853726	SPEF2/DNAH5	2
BP	GO:0072329	monocarboxylic acid catabolic process	2/340	131/18614	0.69388656	0.77093	0.5853726	FAH/CNR1	2
BP	GO:0097722	sperm motility	2/340	131/18614	0.69388656	0.77093	0.5853726	SPEF2/DNAH5	2
BP	GO:0098732	macromolecule	2/340	131/18614	0.69388656	0.77093	0.5853726	SDR16C5/APLNR	2

		deacylation							
BP	GO:0043488	regulation of mRNA stability	3/340	195/18614	0.69450 32	0.77142 29	0.58573 97	BTG2/ZFP36/SLC11A1	3
BP	GO:0006401	RNA catabolic process	5/340	319/18614	0.69570 44	0.77253 19	0.58658 18	PNRC1/BTG2/RNASE2/ZFP36/SLC11A1	5
BP	GO:0001947	heart looping	1/340	65/18614	0.69890 8	0.77541 15	0.58876 83	APLNR	1
BP	GO:0098781	ncRNA transcription	1/340	65/18614	0.69890 8	0.77541 15	0.58876 83	NFATC2	1
BP	GO:0098930	axonal transport	1/340	65/18614	0.69890 8	0.77541 15	0.58876 83	SYBU	1
BP	GO:00	synapse	3/340	197/18614	0.701	0.777	0.590	ROBO2/LRRN3/NTRK2	3

	07416	assembly			0724	3601	2479		
BP	GO:0032388	positive regulation of intracellular transport	3/340	197/18614	0.7010724	0.7773601	0.5902479	PTGS2/IL1B/SFN	3
BP	GO:0099504	synaptic vesicle cycle	3/340	198/18614	0.7043165	0.7801718	0.5923828	P2RY1/SLC18A2/CSPG5	3
BP	GO:0010812	negative regulation of cell-substrate adhesion	1/340	66/18614	0.704427	0.7801718	0.5923828	CX3CL1	1
BP	GO:0060675	ureteric bud morphogenesis	1/340	66/18614	0.704427	0.7801718	0.5923828	FMN1	1

BP	GO:2001259	positive regulation of cation channel activity	1/340	66/18614	0.7044427	0.7801718	0.5923828	ANK2	1
BP	GO:0051224	negative regulation of protein transport	2/340	134/18614	0.7057056	0.780907	0.5929411	PKDCC/IL1B	2
BP	GO:0060972	left/right pattern formation	2/340	134/18614	0.7057056	0.780907	0.5929411	APLNR/DNAH5	2
BP	GO:1900180	regulation of protein localization to nucleus	2/340	134/18614	0.7057056	0.780907	0.5929411	PTGS2/PYHIN1	2

BP	GO:0009150	purine ribonucleotide metabolic process	7/340	445/18614	0.7074015	0.78255565	0.59419935	ZBTB20/ENO1/AMPD1/PDE7B/IER3/SULT1B1/DDIT4	7
BP	GO:0090068	positive regulation of cell cycle process	4/340	262/18614	0.70801092	0.78301126	0.59453398	PPP1R10/DDR2/IL1B/DTL	4
BP	GO:0042490	mechanoreceptor differentiation	1/340	67/18614	0.70984451	0.78412216	0.59538819	NTRK2	1
BP	GO:0043966	histone H3 acetylation	1/340	67/18614	0.70984451	0.78412216	0.59538819	NFKBIZ	1
BP	GO:00	mesonephric tubule	1/340	67/18614	0.709	0.784	0.595	FMN1	1

	72171	morphogenesis			8451	1216	3819		
BP	GO:1902305	regulation of sodium ion transmembrane transport	1/340	67/18614	0.709845 1	0.784121 6	0.595381 9	FXYD1	1
BP	GO:0034655	nucleobase-containing compound catabolic process	7/340	447/18614	0.711752	0.786000 2	0.596808 3	PNRC1/BTG2/RNASE2/PDE7B/ZFP36/SLC11A1/IL6	7
BP	GO:0016445	somatic diversification of immun	1/340	68/18614	0.715164 2	0.788625 7	0.598801 9	NFKBIZ	1

		oglobulins							
BP	GO:0042987	amyloid precursor protein catabolic process	1/340	68/18614	0.7151642	0.7886257	0.5988019	NTRK2	1
BP	GO:0046513	ceramide biosynthetic process	1/340	68/18614	0.7151642	0.7886257	0.5988019	ST6GALNAC3	1
BP	GO:1902808	positive regulation of cell cycle G1/S phase transition	1/340	68/18614	0.7151642	0.7886257	0.5988019	DDR2	1

BP	GO:2000144	positive regulation of DNA-templated transcription initiation	1/340	68/18614	0.715161642	0.788626257	0.598138019	JUN	1
BP	GO:0031929	TOR signaling	2/340	137/18614	0.717161623	0.790606003	0.600030012	EIF4EBP1/DDIT4	2
BP	GO:0001763	morphogenesis of a branching structure	3/340	203/18614	0.720131355	0.793000072	0.602121287	HGF/SULF1/SOCS3	3
BP	GO:0010611	regulation of cardiac muscle	1/340	69/18614	0.72038386	0.793000072	0.602121287	NR4A3	1

		hypertrophy							
BP	GO:0042273	ribosomal large subunit biogenesis	1/340	69/18614	0.720386	0.7930072	0.6021287	WDR12	1
BP	GO:0061077	chaperone-mediated protein folding	1/340	69/18614	0.720386	0.7930072	0.6021287	FKBP5	1
BP	GO:0150116	regulation of cell-substrate junction organization	1/340	69/18614	0.720386	0.7930072	0.6021287	FMN1	1
BP	GO:0072175	epithelial tube formation	2/340	138/18614	0.7208972	0.7931118	0.6022081	BMP5/TGFB2	2

BP	GO:1904950	negative regulation of establishment of protein localization	2/340	138/18614	0.720208972	0.793111118	0.602202081	PKDCC/IL1B	2
BP	GO:0006402	mRNA catabolic process	4/340	268/18614	0.724451563	0.795966626	0.604041449	PNRC1/BTG2/ZFP36/SLC11A1	4
BP	GO:0009581	detection of external stimulus	2/340	139/18614	0.724590505	0.795966626	0.604041449	CXCL12/ANO1	2
BP	GO:0009855	determination of bilateral	2/340	139/18614	0.724590505	0.795966626	0.604041449	APLNR/DNAH5	2

BP	GO:0000271	symmetry polysaccharide biosynthetic process	1/340	70/18614	0.7255124	0.7956626	0.6041449	HAS2	1
BP	GO:0006305	DNA alkylation	1/340	70/18614	0.7255124	0.7956626	0.6041449	FOS	1
BP	GO:0006306	DNA methylation	1/340	70/18614	0.7255124	0.7956626	0.6041449	FOS	1
BP	GO:0032206	positive regulation of telomere maintenance	1/340	70/18614	0.7255124	0.7956626	0.6041449	PPP1R10	1
BP	GO:00	receptor metabo	1/340	70/18614	0.725	0.795	0.604	TRAT1	1

	43112	lic process			5124	6626	1449		
BP	GO:0051148	negative regulation of muscle cell differentiation	1/340	70/18614	0.7255124	0.7956626	0.6041449	NFATC2	1
BP	GO:0061371	determination of heart left/right asymmetry	1/340	70/18614	0.7255124	0.7956626	0.6041449	APLNR	1
BP	GO:1901880	negative regulation of protein depolymerization	1/340	70/18614	0.7255124	0.7956626	0.6041449	PLEKHH2	1

BP	GO:0009141	nucleoside triphosphate metabolic process	4/340	269/18614	0.72719 76	0.79705 21	0.60 52	ZBTB20/ENO1/IER3/DDIT4	4
BP	GO:0090596	sensory organ morphogenesis	4/340	269/18614	0.72719 76	0.79705 21	0.60 52	FRZB/FAT3/COL8A1/NTRK2	4
BP	GO:0009799	specification of symmetry	2/340	140/18614	0.72824 26	0.79796 79	0.60589 53	APLNR/DNAH5	2
BP	GO:0002562	somatic diversification of immune receptors via germline	1/340	71/18614	0.73054 5	0.79911 23	0.60676 42	NFKBIZ	1

		recombination within a single locus							
BP	GO:0016444	somatic cell DNA recombination	1/340	71/18614	0.7305455	0.7991123	0.6067642	NFKBIZ	1
BP	GO:0032922	circadian regulation of gene expression	1/340	71/18614	0.7305455	0.7991123	0.6067642	EGR1	1
BP	GO:0033344	cholesterol efflux	1/340	71/18614	0.7305455	0.7991123	0.6067642	ABCA8	1
BP	GO:0070265	necrotic cell death	1/340	71/18614	0.7305455	0.7991123	0.6067642	DDR2	1

BP	GO:0070918	regulatory ncRNA processing	1/340	71/18614	0.7305545	0.7991123	0.6067642	IL6	1
BP	GO:0031056	regulation of histone modification	2/340	141/18614	0.7318536	0.7998549	0.6073281	SDR16C5/APLNR	2
BP	GO:0032434	regulation of proteasomal ubiquitin-dependent protein catabolic process	2/340	141/18614	0.7318536	0.7998549	0.6073281	TRIB3/TRIB1	2
BP	GO:00	positive regulation of	2/340	141/18614	0.731	0.799	0.607	CCL5/IL6	2

	45727	translation			8536	8549	3281		
BP	GO:0043487	regulation of RNA stability	3/340	207/18614	0.7323125	0.7998754	0.6073437	BTG2/ZFP36/SLC11A1	3
BP	GO:0072522	purine-containing compound biosynthetic process	4/340	271/18614	0.7325502	0.7998754	0.6073437	SHMT2/ENO1/AMPD1/IDO1	4
BP	GO:0090150	establishment of protein localization to membrane	4/340	271/18614	0.7325502	0.7998754	0.6073437	HYOU1/PKDCC/SRPRB/ZDHHC9	4
BP	GO:19	regulation of cell	7/340	457/18614	0.732	0.800	0.607	WEE1/PPP1R10/DDR2/IER3/DTL/INHBA/CCL2	7

	01987	cycle phase transition			855	0316	4623		
BP	GO:0009582	detection of abiotic stimulus	2/340	142/18614	0.7354239	0.8010677	0.60824 9	CXCL12/ANO1	2
BP	GO:0006487	protein N-linked glycosylation	1/340	72/18614	0.7354857	0.8010677	0.608249	FUT3	1
BP	GO:0009988	cell-cell recognition	1/340	72/18614	0.7354857	0.8010677	0.608249	FUT3	1
BP	GO:0014743	regulation of muscle hypertrophy	1/340	72/18614	0.7354857	0.8010677	0.608249	NR4A3	1
BP	GO:00	DNA damage	1/340	72/18614	0.735	0.801	0.608	PYHIN1	1

	30330	response, signal transduction by p53 class mediator			4857	0677	249		
BP	GO:0045665	negative regulation of neuron differentiation	1/340	72/18614	0.7354857	0.8010677	0.6082249	SLC6A4	1
BP	GO:0051310	metaphase plate congression	1/340	72/18614	0.7354857	0.8010677	0.6082249	GEM	1
BP	GO:0099518	vesicle cytoskeletal trafficking	1/340	72/18614	0.7354857	0.8010677	0.6082249	RASGRP1	1

BP	GO:0035567	non-canonical Wnt signaling pathway	1/340	73/18614	0.740336	0.8056598	0.6117358	FRZB	1
BP	GO:0042246	tissue regeneration	1/340	73/18614	0.740336	0.8056598	0.6117358	CAPN3	1
BP	GO:0043462	regulation of ATP-dependent activity	1/340	73/18614	0.740336	0.8056598	0.6117358	RYR2	1
BP	GO:0046474	glycerophospholipid biosynthetic process	3/340	210/18614	0.7411695	0.8063366	0.6122496	PIGW/PIK3CG/SOCS3	3

BP	GO:2000573	positive regulation of DNA biosynthetic process	1/340	74/18614	0.74509 77	0.81037 88	0.61531 89	HGF	1
BP	GO:0017148	negative regulation of translation	4/340	276/18614	0.74542 47	0.81050 32	0.61541 33	BTG2/SHMT2/EIF4EBP1/ZFP36	4
BP	GO:0035264	multicellular organism growth	2/340	145/18614	0.74589 3	0.81054 98	0.61544 88	ARID5B/PKDCC	2
BP	GO:0051783	regulation of nuclear division	2/340	145/18614	0.74589 3	0.81054 98	0.61544 88	RPS6KA2/IL1B	2
BP	GO:00	regulation of mRNA	3/340	212/18614	0.746	0.811	0.615	BTG2/ZFP36/SLC11A1	3

	61013	catabolic process			9442	2685	9945		
BP	GO:0009259	ribonucleotide metabolic process	7/340	464/18614	0.7469801	0.8112685	0.6159945	ZBTB20/ENO1/AMPD1/PDE7B/IER3/SULT1B1/DDIT4	7
BP	GO:0060294	cilium movement involved in cell motility	2/340	146/18614	0.7493031	0.812471	0.6169075	SPEF2/DNAH5	2
BP	GO:0072073	kidney epithelium development	2/340	146/18614	0.7493031	0.812471	0.6169075	ROBO2/FMN1	2
BP	GO:0001707	mesoderm formation	1/340	75/18614	0.7497723	0.812471	0.6169075	INHBA	1

BP	GO:0034637	cellular carbohydrate biosynthetic process	1/340	75/18614	0.74977723	0.81247 1	0.61690 75	HAS2	1
BP	GO:0046323	glucose import	1/340	75/18614	0.74977723	0.81247 1	0.61690 75	ACE	1
BP	GO:0071479	cellular response to ionizing radiation	1/340	75/18614	0.74977723	0.81247 1	0.61690 75	EGR1	1
BP	GO:0072078	nephron tubule morphogenesis	1/340	75/18614	0.74977723	0.81247 1	0.61690 75	FMN1	1
BP	GO:0050821	protein stabilization	3/340	213/18614	0.74979729	0.81247 1	0.61690 75	PIM2/ANK2/PYHIN1	3

BP	GO:0120161	regulation of cold-induced thermogenesis	2/340	147/18614	0.75267 39	0.81534 21	0.61908 75	ALPL/LCN2	2
BP	GO:0007389	pattern specification process	7/340	467/18614	0.75287 03	0.81534 21	0.61908 75	BTG2/BMP5/ROBO2/APLNR/EGR2/BMP1/DNAH5	7
BP	GO:0006073	cellular glucan metabolic process	1/340	76/18614	0.75436 14	0.81556 63	0.61925 78	MGAM	1
BP	GO:0006635	fatty acid beta-oxidation	1/340	76/18614	0.75436 14	0.81556 63	0.61925 78	CNR1	1
BP	GO:00	nucleoside monop	1/340	76/18614	0.754	0.815	0.619	AMPD1	1

	09123	hosphate metabolic process			3614	5663	2578		
BP	GO:0009166	nucleotide catabolic process	1/340	76/18614	0.7543614	0.8155663	0.6192578	PDE7B	1
BP	GO:0044042	glucan metabolic process	1/340	76/18614	0.7543614	0.8155663	0.6192578	MGAM	1
BP	GO:0046470	phosphatidylcholine metabolic process	1/340	76/18614	0.7543614	0.8155663	0.6192578	PLA2G2A	1

BP	GO:0051648	vesicle localization	3/340	215/18614	0.7554133	0.8164719	0.6199454	PIK3CG/ITGA4/RASGRP1	3
BP	GO:0017015	regulation of transforming growth factor beta receptor signaling pathway	2/340	148/18614	0.7560056	0.8166489	0.6200798	LTBP4/ASPN	2
BP	GO:0106106	cold-induced thermogenesis	2/340	148/18614	0.7560056	0.8166489	0.6200798	ALPL/LCN2	2
BP	GO:1990778	protein localization to cell	5/340	345/18614	0.7584394	0.8183476	0.6213696	P2RY1/ANK2/PIGW/PKDCC/SKAP1	5

		periphery							
BP	GO:0021536	diencephalon development	1/340	77/18614	0.7588666	0.8183476	0.6213696	NR4A2	1
BP	GO:0048332	mesoderm morphogenesis	1/340	77/18614	0.7588666	0.8183476	0.6213696	INHBA	1
BP	GO:0072088	nephron epithelium morphogenesis	1/340	77/18614	0.7588666	0.8183476	0.6213696	FMN1	1
BP	GO:0090559	regulation of membrane permeability	1/340	77/18614	0.7588666	0.8183476	0.6213696	IER3	1
BP	GO:1901983	regulation of protein	1/340	77/18614	0.7588666	0.8183476	0.6213696	SDR16C5	1

BP	GO:0002200	acetylation somatic diversification of immune receptors	1/340	78/18614	0.76328 94	0.82241 89	0.62446 09	NFKBIZ	1
BP	GO:0008088	axo-dendritic transport	1/340	78/18614	0.76328 94	0.82241 89	0.62446 09	SYBU	1
BP	GO:0050709	negative regulation of protein secretion	1/340	78/18614	0.76328 94	0.82241 89	0.62446 09	IL1B	1
BP	GO:0019693	ribose phosphate metabo	7/340	473/18614	0.76435 71	0.82333 66	0.62515 78	ZBTB20/ENO1/AMPD1/PDE7B/IER3/SULT1B1/DDIT4	7

		lic process							
BP	GO:0010506	regulation of autophagy	5/340	348/18614	0.7650132	0.8238105	0.6255176	TRIB3/HGF/PIM2/HMOX1/DCN	5
BP	GO:1903844	regulation of cellular response to transforming growth factor beta stimulus	2/340	151/18614	0.7657696	0.8243921	0.6259592	LTBP4/ASPN	2
BP	GO:0050688	regulation of defense response to virus	1/340	79/18614	0.7676313	0.8259296	0.6271266	IL1B	1

BP	GO:0061333	renal tubule morphogenesis	1/340	79/18614	0.76763 13	0.82592 96	0.62712 66	FMN1	1
BP	GO:0043535	regulation of blood vessel endothelial cell migration	2/340	152/18614	0.76894 82	0.82695 78	0.62790 73	PTGS2/HMOX1	2
BP	GO:0099003	vesicle-mediated transport in synapse	3/340	220/18614	0.76902 09	0.82695 78	0.62790 73	P2RY1/SLC18A2/CSPG5	3
BP	GO:0032890	regulation of organic acid transport	1/340	80/18614	0.77189 38	0.82838 75	0.62899 29	IL1B	1

BP	GO:0043242	negative regulation of protein-containing complex disassembly	1/340	80/18614	0.77189 38	0.82838 75	0.62899 29	PLEKHH2	1
BP	GO:0072028	nephron morphogenesis	1/340	80/18614	0.77189 38	0.82838 75	0.62899 29	FMN1	1
BP	GO:0097061	dendritic spine organization	1/340	80/18614	0.77189 38	0.82838 75	0.62899 29	FCGR2B	1
BP	GO:0140115	export across plasma membrane	1/340	80/18614	0.77189 38	0.82838 75	0.62899 29	FXYD1	1

BP	GO:2000142	regulation of DNA-templated transcription initiation	1/340	80/18614	0.7718938	0.8283875	0.62899929	JUN	1
BP	GO:0006352	DNA-templated transcription initiation	2/340	153/18614	0.7720893	0.8283875	0.62899929	JUN/NFKBIZ	2
BP	GO:0016573	histone acetylation	2/340	153/18614	0.7720893	0.8283875	0.62899929	SDR16C5/NFKBIZ	2
BP	GO:0035148	tube formation	2/340	154/18614	0.77519032	0.83148336	0.63134337	BMP5/TGFB2	2

BP	GO:0044272	sulfur compound biosynthetic process	2/340	155/18614	0.77826601	0.83453383	0.63366632	CBS/CTH	2
BP	GO:0033157	regulation of intracellular protein transport	3/340	224/18614	0.77945584	0.83519338	0.63416609	PTGS2/IL1B/SFN	3
BP	GO:0050953	sensory perception of light stimulus	3/340	224/18614	0.77945584	0.83519338	0.63416609	PCDH15/HMCN1/LUM	3
BP	GO:0006513	protein monoubiquitination	1/340	82/18614	0.78018863	0.83519338	0.63416609	DTL	1

BP	GO:0010921	regulation of phosphatase activity	1/340	82/18614	0.78018 63	0.83519 38	0.63416 09	TGFB2	1
BP	GO:0051149	positive regulation of muscle cell differentiation	1/340	82/18614	0.78018 63	0.83519 38	0.63416 09	CTH	1
BP	GO:0061097	regulation of protein tyrosine kinase activity	1/340	82/18614	0.78018 63	0.83519 38	0.63416 09	ACE	1
BP	GO:0001539	cilium or flagellum-dependent cell motility	2/340	156/18614	0.78129 04	0.83590 62	0.63470 18	SPEF2/DNAH5	2

BP	GO:0060285	cilium-dependent cell motility	2/340	156/18614	0.7812904	0.8359062	0.6347018	SPEF2/DNAH5	2
BP	GO:0032392	DNA geometric change	1/340	83/18614	0.7842191	0.8383335	0.6365449	HMGB3	1
BP	GO:0070897	transcription preinitiation complex assembly	1/340	83/18614	0.7842191	0.8383335	0.6365449	NFKBIZ	1
BP	GO:0072332	intrinsic apoptotic signaling pathway by p53 class	1/340	83/18614	0.7842191	0.8383335	0.6365449	DDIT4	1

BP	GO:0007163	mediator or establishment or maintenance of cell polarity	3/340	226/18614	0.78453303	0.8384311	0.63661 89	WEE1/RHOBTB2/CD3G	3
BP	GO:0003002	regionalization	6/340	422/18614	0.78623 53	0.84001 75	0.63782 35	BTG2/ROBO2/APLNR/EGR2/BMP1/DNAH5	6
BP	GO:0090501	RNA phosphodiester bond hydrolysis	2/340	158/18614	0.78724 25	0.84085 8	0.63846 17	RNASE2/AZGP1	2
BP	GO:0000281	mitotic cytokinesis	1/340	84/18614	0.78817 82	0.84138 57	0.63886 24	MYH10	1
BP	GO:00	establishment of	1/340	84/18614	0.788	0.841	0.638	GEM	1

	51303	chromosome localization			1782	3857	8624		
BP	GO:0019318	hexose metabolic process	3/340	228/18614	0.789505 7	0.842566 9	0.639759 3	ENO1/SLC39A14/ATF3	3
BP	GO:0016241	regulation of macroautophagy	2/340	159/18614	0.790164 9	0.843034 3	0.640114 1	HMOX1/DCN	2
BP	GO:0055013	cardiac muscle cell development	1/340	85/18614	0.792064 8	0.844588 4	0.641294 2	MYH11	1
BP	GO:1901292	nucleoside phosphate catabolic	1/340	85/18614	0.792064 8	0.844588 4	0.641294 2	PDE7B	1

		process							
BP	GO:0050657	nucleic acid transport	2/340	160/18614	0.7930519	0.845168	0.64173343	RFTN2/ZFP36	2
BP	GO:0050658	RNA transport	2/340	160/18614	0.7930519	0.845168	0.64173343	RFTN2/ZFP36	2
BP	GO:1903050	regulation of proteolysis involved in protein catabolic process	3/340	230/18614	0.7943857	0.8456062	0.6420067	TRIB3/PYHIN1/TRIB1	3
BP	GO:0006650	glycerophospholipid metabolic	4/340	297/18614	0.79453581	0.8456062	0.6420067	PIGW/PIK3CG/PLA2G2A/SOCS3	4

		process							
BP	GO:0008543	fibroblast growth factor receptor signaling pathway	1/340	86/18614	0.79588804	0.84560 62	0.6420667	SULF1	1
BP	GO:0021766	hippocampus development	1/340	86/18614	0.79588804	0.8456062	0.6420667	BTG2	1
BP	GO:0045069	regulation of viral genome replication	1/340	86/18614	0.79588804	0.8456062	0.6420667	CCL5	1
BP	GO:00	focal adhesion	1/340	86/18614	0.795	0.845	0.642	FMN1	1

	48041	assembly			8804	6062	067		
BP	GO:0061053	somite development	1/340	86/18614	0.79588804	0.84560662	0.642067	FRZB	1
BP	GO:1901879	regulation of protein depolymerization	1/340	86/18614	0.79588804	0.84560662	0.642067	PLEKHH2	1
BP	GO:0018393	internal peptidyl-lysine acetylation	2/340	161/18614	0.7959038	0.84560662	0.642067	SDR16C5/NFKBIZ	2
BP	GO:0051147	regulation of muscle cell differentiation	2/340	161/18614	0.7959038	0.84560662	0.642067	NFATC2/CTH	2
BP	GO:19	adaptive	2/340	161/18614	0.795	0.845	0.642	ALPL/LCN2	2

	90845	thermogenesis			9038	6062	067		
BP	GO:0030041	actin filament polymerization	2/340	162/18614	0.7987210	0.8479062	0.6438134	FMN1/CSF3	2
BP	GO:0002088	lens development in camera-type eye	1/340	87/18614	0.7996261	0.8479062	0.6438134	SLC7A11	1
BP	GO:0008589	regulation of smoothened signaling pathway	1/340	87/18614	0.7996261	0.8479062	0.6438134	CD3E	1
BP	GO:0010232	vascular transport	1/340	87/18614	0.7996261	0.8479062	0.6438134	SLC1A3	1

BP	GO:0046785	microtubule polymerization	1/340	87/18614	0.79962 61	0.84790 62	0.64381 34	TPPP3	1
BP	GO:0090502	RNA phosphodiester bond hydrolysis, endonucleolytic	1/340	87/18614	0.79962 61	0.84790 62	0.64381 34	RNASE2	1
BP	GO:0150104	transport across blood-brain barrier	1/340	87/18614	0.79962 61	0.84790 62	0.64381 34	SLC1A3	1
BP	GO:0006475	internal protein amino acid acetylation	2/340	163/18614	0.80150 38	0.84918 85	0.64478 7	SDR16C5/NFKBIZ	2

BP	GO:0006606	protein import into nucleus	2/340	163/18614	0.8015038	0.8491885	0.64478 7	PTGS2/PPP1R10	2
BP	GO:0051236	establishment of RNA localization	2/340	163/18614	0.8015038	0.8491885	0.644787	RFTN2/ZFP36	2
BP	GO:2000113	negative regulation of cellular macromolecule biosynthetic process	4/340	301/18614	0.80297971	0.8505513	0.64579 27	BTG2/SHMT2/EIF4EBP1/ZFP36	4
BP	GO:0016358	dendrite development	3/340	234/18614	0.80386 34	0.85121 51	0.64632 59	BMP5/FAT3/CD3E	3

BP	GO:0007589	body fluid secretion	1/340	89/18614	0.80691 32	0.85402 74	0.64846 12	ANO1	1
BP	GO:0048639	positive regulation of developmental growth	2/340	165/18614	0.80696 74	0.85402 74	0.64846 12	CXCL12/CAPN3	2
BP	GO:1903313	positive regulation of mRNA metabolic process	2/340	166/18614	0.80964 87	0.85662 72	0.65043 52	BTG2/ZFP36	2
BP	GO:0071103	DNA conformation change	1/340	90/18614	0.81045 7	0.85676 88	0.65054 27	HMGB3	1
BP	GO:01	neuron projection	1/340	90/18614	0.810	0.856	0.650	FCGR2B	1

	06027	organization			457	7688	5427		
BP	GO:1903351	cellular response to dopamine	1/340	90/18614	0.810457	0.8567688	0.6505427	HRH2	1
BP	GO:0001843	neural tube closure	1/340	91/18614	0.813936	0.8597311	0.652792	TGFB2	1
BP	GO:0030901	midbrain development	1/340	91/18614	0.813936	0.8597311	0.652792	NR4A2	1
BP	GO:1903350	response to dopamine	1/340	91/18614	0.813936	0.8597311	0.652792	HRH2	1
BP	GO:0051170	import into nucleus	2/340	168/18614	0.8149123	0.8605239	0.653394	PTGS2/PPP1R10	2
BP	GO:00	chromosome	1/340	92/18614	0.817	0.862	0.654	GEM	1

	50000	localization			3513	0569	558		
BP	GO:0055017	cardiac muscle tissue growth	1/340	92/18614	0.8173513	0.8620569	0.6545558	TGFB2	1
BP	GO:0060606	tube closure	1/340	92/18614	0.8173513	0.8620569	0.6545558	TGFB2	1
BP	GO:0032984	protein-containing complex disassembly	3/340	240/18614	0.8173914	0.8620569	0.6545558	NCKAP5/PLEKHH2/DDIT4	3
BP	GO:0034250	positive regulation of amide metabolic process	2/340	169/18614	0.8174951	0.8620569	0.6545558	CCL5/IL6	2

BP	GO:0007009	plasma membrane organization	2/340	170/18614	0.820045 6	0.864507 3	0.656418 6	ANK2/TGFB2	2
BP	GO:2001020	regulation of response to DNA damage stimulus	4/340	311/18614	0.822851 6	0.867225 5	0.658482 5	CXCL12/PPP1R10/IER3/PYHIN1	4
BP	GO:0045333	cellular respiration	3/340	243/18614	0.823854	0.867711 2	0.658851 3	SHMT2/IDH1/NR4A3	3
BP	GO:0035278	miRNA-mediated gene silencing by inhibitio	1/340	94/18614	0.823995 5	0.867711 2	0.658851 3	ZFP36	1

		n of translation							
BP	GO:0051492	regulation of stress fiber assembly	1/340	94/18614	0.823 99 55	0.867 71 12	0.658 85 13	SERPINF2	1
BP	GO:0010970	transport along microtubule	2/340	172/18614	0.825 05 13	0.868 34 31	0.659 33 11	RASGRP1/SYBU	2
BP	GO:0018394	peptidyl-lysine acetylation	2/340	172/18614	0.825 05 13	0.868 34 31	0.659 33 11	SDR16C5/NFKBIZ	2
BP	GO:0060070	canonical Wnt signaling pathway	4/340	313/18614	0.826 62 21	0.869 75 62	0.660 40 4	FRZB/EGR1/SHISA3/NR4A2	4
BP	GO:00	dorsal/ventral pattern	1/340	95/18614	0.827	0.869	0.660	BMP1	1

	09953	formation			2267	912	5223		
BP	GO:0042982	amyloid precursor protein metabolic process	1/340	95/18614	0.827222267	0.869912	0.6605223	NTRK2	1
BP	GO:0072331	signal transduction by p53 class mediator	2/340	173/18614	0.8275507	0.8699667	0.6605638	PYHIN1/DDIT4	2
BP	GO:0090305	nucleic acid phosphodiester bond hydrolysis	3/340	245/18614	0.8280534	0.8703301	0.6608177	RNASE2/ZFP36/AZGP1	3

BP	GO:0007044	cell-substrate junction assembly	1/340	96/18614	0.830398 7	0.872044 5	0.662141 6	FMN1	1
BP	GO:0048534	hematopoietic or lymphoid organ development	1/340	96/18614	0.830398 7	0.872044 5	0.662141 6	NFKBIZ	1
BP	GO:0072080	nephron tubule development	1/340	96/18614	0.830398 7	0.872044 5	0.662141 6	FMN1	1
BP	GO:1905475	regulation of protein localization to membrane	2/340	175/18614	0.832325 7	0.873827 4	0.663495 3	PKDCC/SLC7A11	2

BP	GO:0060993	kidney morphogenesis	1/340	97/18614	0.83335127	0.8743509	0.66389928	FMN1	1
BP	GO:0099175	regulation of postsynapse organization	1/340	97/18614	0.83335127	0.8743509	0.66389928	FCGR2B	1
BP	GO:1903321	negative regulation of protein modification by small protein conjugation or removal	1/340	97/18614	0.83335127	0.8743509	0.66389928	CAPN3	1

BP	GO:0007281	germ cell development	4/340	319/18614	0.837325374	0.878333111	0.666691549	RPS6KA2/SPEF2/CFTR/PTX3	4
BP	GO:0043543	protein acylation	3/340	250/18614	0.838131792	0.878525206	0.667670588	SDR16C5/NFKBIZ/ZDHHC9	3
BP	GO:0045017	glycerolipid biosynthetic process	3/340	250/18614	0.838131792	0.878525206	0.667670588	PIGW/PIK3CG/SOCS3	3
BP	GO:0006641	triglyceride metabolic process	1/340	100/18614	0.842425167	0.881616121	0.669694062	PIK3CG	1
BP	GO:0030512	negative regulation of transforming	1/340	100/18614	0.842425167	0.881616121	0.669694062	ASPN	1

		growth factor beta receptor signaling pathway							
BP	GO:0035282	segmentation	1/340	100/18614	0.84251 67	0.88161 21	0.66940 62	EGR2	1
BP	GO:0060419	heart growth	1/340	100/18614	0.84251 67	0.88161 21	0.66940 62	TGFB2	1
BP	GO:0061326	renal tubule development	1/340	100/18614	0.84251 67	0.88161 21	0.66940 62	FMN1	1
BP	GO:0140888	interferon-mediated signalin	1/340	100/18614	0.84251 67	0.88161 21	0.66940 62	IRF1	1

		g pathway							
BP	GO:0031647	regulation of protein stability	4/340	322/18614	0.84277767	0.881642	0.6694289	PIM2/ANK2/CAPN3/PYHIN1	4
BP	GO:0007040	lysosome organization	1/340	101/18614	0.84540888	0.8839103	0.67151512	SRPX	1
BP	GO:0080171	lytic vacuole organization	1/340	101/18614	0.84540888	0.8839103	0.67151512	SRPX	1
BP	GO:0009101	glycoprotein biosynthetic process	4/340	324/18614	0.84619003	0.8844847	0.67155874	FUT3/GXYLT2/ST6GALNAC3/TMTC1	4
BP	GO:1903320	regulation of protein modification	3/340	255/18614	0.84778877	0.8518814	0.67211164	TRIB3/EGR1/CAPN3	3

		by small protein conjugation or removal								
BP	GO:0006892	post-Golgi vesicle-mediated transport	1/340	102/18614	0.8482479	0.8851814	0.6721164	PKDCC		1
BP	GO:0042472	inner ear morphogenesis	1/340	102/18614	0.8482479	0.8851814	0.6721164	FRZB		1
BP	GO:0046620	regulation of organ growth	1/340	102/18614	0.8482479	0.8851814	0.6721164	SLC6A4		1
BP	GO:0006479	protein methylation	2/340	182/18614	0.8482505	0.8851814	0.6721164	BTG2/NFKBIZ		2

BP	GO:0008213	protein alkylation	2/340	182/18614	0.848482505	0.885181814	0.672116 4	BTG2/NFKBIZ	2
BP	GO:0005996	monosaccharide metabolic process	3/340	256/18614	0.84964 9	0.886393981	0.673040402	ENO1/SLC39A14/ATF3	3
BP	GO:0016055	Wnt signaling pathway	6/340	461/18614	0.850207 18	0.886596 5	0.673191909	FRZB/EGR1/SULF1/SHISA3/CPE/NR4A2	6
BP	GO:0061025	membrane fusion	2/340	183/18614	0.850541	0.88670 66	0.67327 44	DPP4/CH25H	2
BP	GO:0150115	cell-substrate junction organization	1/340	103/18614	0.851035 1	0.868873	0.67340 08	FMN1	1

BP	GO:1903076	regulation of protein localization to plasma membrane	1/340	103/18614	0.85103 51	0.88687 3	0.67340 08	PKDCC	1
BP	GO:0198738	cell-cell signaling by wnt	6/340	463/18614	0.85287 87	0.88855 14	0.67467 52	FRZB/EGR1/SULF1/SHISA3/CPE/NR4A2	6
BP	GO:0032204	regulation of telomere maintenance	1/340	104/18614	0.85377 12	0.88875 22	0.67482 77	PPP1R10	1
BP	GO:0034968	histone lysine methylation	1/340	104/18614	0.85377 12	0.88875 22	0.67482 77	NFKBIZ	1
BP	GO:0110020	regulation of actomyosin	1/340	104/18614	0.85377 12	0.88875 22	0.67482 77	SERPINF2	1

		structure organization							
BP	GO:0099173	postsynapse organization	2/340	185/18614	0.8546455	0.8894195	0.6753343	SLC7A11/FCGR2B	2
BP	GO:0009062	fatty acid catabolic process	1/340	105/18614	0.8564572	0.8910615	0.6765811	CNR1	1
BP	GO:0060828	regulation of canonical Wnt signaling pathway	3/340	260/18614	0.8568979	0.8912767	0.6767445	FRZB/EGR1/SHISA3	3
BP	GO:0006754	ATP biosynthetic	1/340	106/18614	0.859094	0.8929685	0.6780291	ENO1	1

		process								
BP	GO:1901796	regulation of signal transduction by p53 class mediator	1/340	106/18614	0.8590094	0.8929685	0.6780291	PYHIN1		1
BP	GO:0032386	regulation of intracellular transport	4/340	332/18614	0.8592274	0.8929685	0.6780291	PTGS2/IER3/IL1B/SFN		4
BP	GO:0006164	purine nucleotide biosynthetic process	3/340	262/18614	0.8604067	0.8939504	0.6787746	ENO1/AMPD1/IDO1		3

BP	GO:0006605	protein targeting	4/340	333/18614	0.860788891	0.894103912	0.678891239	HYOU1/LTBP2/SRPRB/ZDHHC9	4
BP	GO:0032526	response to retinoic acid	1/340	107/18614	0.861682543	0.894544322	0.679225625	SLC6A4	1
BP	GO:0062207	regulation of pattern recognition receptor signaling pathway	1/340	107/18614	0.861682543	0.894544322	0.679225625	IRF1	1
BP	GO:0018107	peptidyl-threonine phosphorylation	1/340	109/18614	0.866671781	0.898792613	0.682245513	DDIT4	1

BP	GO:0030038	contractile actin filament bundle assembly	1/340	109/18614	0.86671 81	0.89879 26	0.68245 13	SERPINF2	1
BP	GO:0043149	stress fiber assembly	1/340	109/18614	0.86671 81	0.89879 26	0.68245 13	SERPINF2	1
BP	GO:0120034	positive regulation of plasma membrane bounded cell projection assembly	1/340	109/18614	0.86671 81	0.89879 26	0.68245 13	DPYSL3	1

BP	GO:0030111	regulation of Wnt signaling pathway	4/340	338/18614	0.86837 82	0.90026 92	0.68357 25	FRZB/EGR1/SULF1/SHISA3	4
BP	GO:0009755	hormone-mediated signaling pathway	2/340	192/18614	0.86862 32	0.90027 83	0.68357 94	NR4A3/NR1D2	2
BP	GO:0003341	cilium movement	2/340	193/18614	0.87051 64	0.90199 51	0.68488 3	SPEF2/DNAH5	2
BP	GO:0043254	regulation of protein-containing complex	5/340	409/18614	0.87096 07	0.90221 02	0.68504 63	SKAP1/FMN1/TPPP3/GBP5/CSF3	5

		assembly							
BP	GO:2001252	positive regulation of chromosome organization	1/340	111/18614	0.8715709	0.9025597	0.68534	PPP1R10	1
BP	GO:0043433	negative regulation of DNA-binding transcription factor activity	2/340	194/18614	0.8723845	0.9029488	0.68560 71	HMOX1/TRIB1	2
BP	GO:0061136	regulation of proteasomal protein catabolic	2/340	194/18614	0.8723845	0.9029488	0.6856071	TRIB3/TRIB1	2

BP	GO:0000079	process regulation of cyclin-dependent protein serine/threonine kinase activity	1/340	112/18614	0.87393 09	0.90405 82	0.68644 95	SFN	1
BP	GO:0043473	pigmentation	1/340	112/18614	0.87393 09	0.90405 82	0.68644 95	ADAMTS9	1
BP	GO:0120032	regulation of plasma membrane bounded cell projection	2/340	195/18614	0.87422 78	0.90412	0.68649 64	DPYSL3/STAP1	2

		assembly							
BP	GO:0009060	aerobic respiration	2/340	196/18614	0.8760467	0.9057172	0.6877092	SHMT2/IDH1	2
BP	GO:0061640	cytoskeleton-dependent cytokinesis	1/340	113/18614	0.8762476	0.9057172	0.6877092	MYH10	1
BP	GO:0006403	RNA localization	2/340	197/18614	0.8778413	0.90638312	0.68821133	RFTN2/ZFP36	2
BP	GO:0007286	spermatid development	2/340	197/18614	0.8778413	0.90638312	0.68821133	SPEF2/CFTR	2
BP	GO:0051650	establishment of vesicle localization	2/340	197/18614	0.8778413	0.90638312	0.68821133	ITGA4/RASGRP1	2

BP	GO:0060491	regulation of cell projection assembly	2/340	197/18614	0.877841 3	0.906381 2	0.688213 3	DPYSL3/STAP1	2
BP	GO:0071897	DNA biosynthetic process	2/340	198/18614	0.879611 9	0.907963 5	0.689414 7	HGF/DTL	2
BP	GO:0021761	limbic system development	1/340	115/18614	0.880754 4	0.908896 6	0.690123 2	BTG2	1
BP	GO:0001824	blastocyst development	1/340	116/18614	0.882946	0.910172 4	0.691092	JUNB	1
BP	GO:0032006	regulation of TOR signaling	1/340	116/18614	0.882946	0.910172 4	0.691092	DDIT4	1

BP	GO:0072009	nephron epithelium development	1/340	116/18614	0.882946	0.9101724	0.6910092	FMN1	1
BP	GO:1904029	regulation of cyclin-dependent protein kinase activity	1/340	116/18614	0.882946	0.9101724	0.6910092	SFN	1
BP	GO:0007088	regulation of mitotic nuclear division	1/340	117/18614	0.8850975	0.9114041	0.6920272	IL1B	1
BP	GO:0009206	purine ribonucleoside triphosphate biosynthetic	1/340	117/18614	0.8850975	0.9114041	0.6920272	ENO1	1

		process							
BP	GO:0019751	polyol metabolic process	1/340	117/18614	0.88509075	0.91140041	0.69202072	P2RY1	1
BP	GO:0090630	activation of GTPase activity	1/340	117/18614	0.88509075	0.91140041	0.69202072	RASGRP1	1
BP	GO:0009145	purine nucleoside triphosphate biosynthetic process	1/340	118/18614	0.88720096	0.91308056	0.69330039	ENO1	1
BP	GO:0030510	regulation of BMP signaling	1/340	118/18614	0.88720096	0.91308056	0.69330039	SULF1	1

		pathway							
BP	GO:0009566	fertilization	2/340	203/18614	0.88811155	0.91377111	0.69382245	TRPC6/NELL2	2
BP	GO:0018210	peptidyl-threonine modification	1/340	119/18614	0.8892833	0.9147253	0.6945549	DDIT4	1
BP	GO:0048515	spermatid differentiation	2/340	204/18614	0.88974 81	0.91495 69	0.69472 48	SPEF2/CFTR	2
BP	GO:0006304	DNA modification	1/340	120/18614	0.89131 83	0.91558 33	0.69520 05	FOS	1
BP	GO:0007006	mitochondrial membrane organization	1/340	120/18614	0.89131 83	0.91558 33	0.69520 05	IER3	1

BP	GO:0030326	embryonic limb morphogenesis	1/340	120/18614	0.89131 83	0.91558 33	0.69520 05	TGFB2	1
BP	GO:0035113	embryonic appendage morphogenesis	1/340	120/18614	0.89131 83	0.91558 33	0.69520 05	TGFB2	1
BP	GO:0031398	positive regulation of protein ubiquitination	1/340	121/18614	0.89331 63	0.91714 13	0.69638 35	TRIB3	1
BP	GO:2000278	regulation of DNA biosynthetic process	1/340	121/18614	0.89331 63	0.91714 13	0.69638 35	HGF	1
BP	GO:00	nucleoside bisphos	1/340	122/18614	0.895	0.918	0.697	SULT1B1	1

	33865	phate metabolic process			2778	4128	3489			
BP	GO:0033875	ribonucleoside bisphosphate metabolic process	1/340	122/18614	0.8952778	0.9184128	0.6973489	SULT1B1		1
BP	GO:0034032	purine nucleoside bisphosphate metabolic process	1/340	122/18614	0.8952778	0.9184128	0.6973489	SULT1B1		1
BP	GO:0006473	protein acetylation	2/340	208/18614	0.8960605	0.9189683	0.6977707	SDR16C5/NFKBIZ		2

BP	GO:0009201	ribonucleoside triphosphate biosynthetic process	1/340	123/18614	0.89720 32	0.91915 09	0.69790 94	ENO1	1
BP	GO:0018022	peptidyl-lysine methylation	1/340	123/18614	0.89720 32	0.91915 09	0.69790 94	NFKBIZ	1
BP	GO:0042471	ear morphogenesis	1/340	123/18614	0.89720 32	0.91915 09	0.69790 94	FRZB	1
BP	GO:0043244	regulation of protein-containing complex disassembly	1/340	123/18614	0.89720 32	0.91915 09	0.69790 94	PLEKHH2	1

BP	GO:1903828	negative regulation of protein localization	2/340	210/18614	0.89990893	0.92083356	0.69918885	PKDCC/IL1B	2
BP	GO:0010639	negative regulation of organelle organization	4/340	362/18614	0.90000488	0.92157706	0.69974466	RPS6KA2/HGF/PLEKHH2/IER3	4
BP	GO:0031623	receptor internalization	1/340	125/18614	0.90094489	0.92224445	0.70002583	SELE	1
BP	GO:0030705	cytoskeleton-dependent intracellular	2/340	212/18614	0.90203358	0.92310492	0.70009148	RASGRP1/SYBU	2

		transport							
BP	GO:0035303	regulation of dephosphorylation	1/340	127/18614	0.90455584	0.92519338	0.70249977	TGFB2	1
BP	GO:0055007	cardiac muscle cell differentiation	1/340	127/18614	0.90455584	0.92519338	0.70249977	MYH11	1
BP	GO:0006639	acylglycerol metabolic process	1/340	128/18614	0.90631337	0.92649319	0.70348833	PIK3CG	1
BP	GO:1904375	regulation of protein localization to cell periphery	1/340	128/18614	0.90631337	0.92649319	0.70348833	PKDCC	1

BP	GO:0048562	embryonic organ morphogenesis	3/340	294/18614	0.907101	0.927048	0.703905 6	RYR2/FRZB/APLNR	3
BP	GO:0006638	neutral lipid metabolic process	1/340	129/18614	0.908036 8	0.927506 9	0.704254 1	PIK3CG	1
BP	GO:0048706	embryonic skeletal system development	1/340	129/18614	0.908036 8	0.927506 9	0.704254 1	SULF1	1
BP	GO:0009165	nucleotide biosynthetic process	3/340	295/18614	0.908302	0.927529 1	0.704270 9	ENO1/AMPD1/IDO1	3
BP	GO:00	purine ribonucleotide	2/340	217/18614	0.909	0.928	0.704	ENO1/AMPD1	2

	09152	biosynthetic process			0553	0497	6662			
BP	GO:0007052	mitotic spindle organization	1/340	130/18614	0.9097284	0.9282396	0.7048103	TACC2		1
BP	GO:0051028	mRNA transport	1/340	130/18614	0.9097284	0.9282396	0.7048103	ZFP36		1
BP	GO:1901293	nucleoside phosphate biosynthetic process	3/340	297/18614	0.9106618	0.9289433	0.7053447	ENO1/AMPD1/IDO1		3
BP	GO:0006888	endoplasmic reticulum to Golgi vesicle-	1/340	131/18614	0.9113888	0.9294361	0.7057189	HYOU1		1

		mediated transport							
BP	GO:0032543	mitochondrial translation	1/340	132/18614	0.913018 9	0.930620 4	0.706618 1	SHMT2	1
BP	GO:0007601	visual perception	2/340	220/18614	0.913038 5	0.930620 4	0.706618 1	HMCN1/LUM	2
BP	GO:0015931	nucleobase-containing compound transport	2/340	221/18614	0.914329 6	0.931687 2	0.707428 1	RFTN2/ZFP36	2
BP	GO:0016571	histone methylation	1/340	133/18614	0.914619	0.931733	0.707462 9	NFKBIZ	1

BP	GO:0009142	nucleoside triphosphate biosynthetic process	1/340	134/18614	0.91618898	0.93308837	0.70848885	ENO1	1
BP	GO:0051054	positive regulation of DNA metabolic process	3/340	303/18614	0.9174155	0.93408199	0.70924 64	HGF/PPP1R10/IL6	3
BP	GO:0043401	steroid hormone mediated signaling pathway	1/340	135/18614	0.91737317	0.93415548	0.70930 18	NR4A3	1

BP	GO:0034504	protein localization to nucleus	3/340	304/18614	0.918494 4	0.934681 4	0.709701 6	PTGS2/PPP1R10/PYHIN1	3
BP	GO:0030879	mammary gland development	1/340	136/18614	0.919245 4	0.935196	0.710092 3	FASN	1
BP	GO:0072594	establishment of protein localization to organelle	5/340	453/18614	0.919556 7	0.935263	0.710143 2	RYR2/HYOU1/PTGS2/SRPRB/PPP1R10	5
BP	GO:0007498	mesoderm development	1/340	137/18614	0.920731 3	0.936207 9	0.710860 6	INHBA	1
BP	GO:1903322	positive regulation of protein modific	1/340	139/18614	0.923621 8	0.938896 5	0.712902 1	TRIB3	1

		ation by small protein conjugation or removal							
BP	GO:0046486	glycerolipid metabolic process	4/340	385/18614	0.9239072	0.9389361	0.71293322	PIGW/PIK3CG/PLA2G2A/SOCS3	4
BP	GO:1903008	organelle disassembly	1/340	140/18614	0.92502274	0.93982391	0.71360063	PLIN2	1
BP	GO:0009260	ribonucleotide biosynthetic process	2/340	231/18614	0.92628882	0.9408548	0.71438385	ENO1/AMPD1	2

BP	GO:0140694	non-membrane-bounded organelle assembly	4/340	390/18614	0.928363651	0.942717124	0.715797995	SH3PXD2B/CAPN3/KRT19/MYH11	4
BP	GO:1902600	proton transmembrane transport	1/340	143/18614	0.929290914	0.943431985	0.716161686	SLC11A1	1
BP	GO:0007224	smoothened signaling pathway	1/340	146/18614	0.932939356	0.946465968	0.71874 9	CD3E	1
BP	GO:0030177	positive regulation of Wnt signalin	1/340	146/18614	0.932939356	0.946465968	0.71874 9	SULF1	1

		g pathway							
BP	GO:0046390	ribose phosphate biosynthetic process	2/340	238/18614	0.9337045	0.9471248	0.7191499	ENO1/AMPD1	2
BP	GO:0043414	macromolecule methylation	3/340	320/18614	0.9341021	0.947276	0.7192646	BTG2/NFKBIZ/FOS	3
BP	GO:0001701	in utero embryonic development	4/340	398/18614	0.9350109	0.9479453	0.7197729	BMP5/JUNB/KRT19/SOCS3	4
BP	GO:0006119	oxidative phosphorylation	1/340	148/18614	0.9353823	0.9480697	0.7198673	SHMT2	1

BP	GO:0098876	vesicle-mediated transport to the plasma membrane	1/340	149/18614	0.9365721	0.94902 32	0.72059 13	PKDCC	1
BP	GO:0000209	protein polyubiquitination	2/340	244/18614	0.93949 47	0.95173 16	0.72264 78	NQO1/DTL	2
BP	GO:1903311	regulation of mRNA metabolic process	3/340	329/18614	0.94162 49	0.95363 6	0.72409 38	BTG2/ZFP36/SLC11A1	3
BP	GO:0030010	establishment of cell polarity	1/340	155/18614	0.94326 56	0.95504 39	0.72516 28	WEE1	1
BP	GO:00	telomere	1/340	156/18614	0.944	0.955	0.725	PPP1R10	1

	00723	maintenance			3106	848	7734		
BP	GO:0015980	energy derivation by oxidation of organic compounds	3/340	337/18614	0.9476373	0.9589606	0.7281368	SHMT2/IDH1/NR4A3	3
BP	GO:0007018	microtubule-based movement	4/340	417/18614	0.9486153	0.9596955	0.7286948	SPEF2/RASGRP1/SYBU/DNAH5	4
BP	GO:1902850	microtubule cytoskeleton organization involved in mitosis	1/340	161/18614	0.9492547	0.9600875	0.7289924	TACC2	1

BP	GO:0016311	dephosphorylation	3/340	342/18614	0.9510978	0.96145111	0.73002278	TGFB2/EPHX2/ALPL	3
BP	GO:0007338	single fertilization	1/340	163/18614	0.9511075	0.96145111	0.73002278	TRPC6	1
BP	GO:0051494	negative regulation of cytoskeleton organization	1/340	164/18614	0.95200085	0.96185516	0.73033319	PLEKHH2	1
BP	GO:0140053	mitochondrial gene expression	1/340	164/18614	0.95200085	0.96185516	0.73033319	SHMT2	1
BP	GO:1902904	negative regulation of supram	1/340	167/18614	0.95461632	0.96397921	0.7319942	PLEKHH2	1

		olecular fiber organization								
BP	GO:2001022	positive regulation of response to DNA damage stimulus	1/340	167/18614	0.954613 2	0.963972 1	0.731942	PYHIN1		1
BP	GO:0090101	negative regulation of transmembrane receptor protein serine/threonin	1/340	169/18614	0.956270 9	0.965390 3	0.733018 8	ASPN		1

		e kinase signaling pathway							
BP	GO:0032259	methylation	3/340	365/18614	0.96444409	0.97383804	0.73908857	BTG2/NFKBIZ/FOS	3
BP	GO:0045165	cell fate commitment	2/340	281/18614	0.9658788	0.97457727	0.7399991	LY9/IL6	2
BP	GO:0000910	cytokinesis	1/340	185/18614	0.96753535	0.97598847	0.74106631	MYH10	1
BP	GO:0032200	telomere organization	1/340	187/18614	0.96872704	0.97769234	0.74177759	PPP1R10	1
BP	GO:0006839	mitochondrial transport	1/340	189/18614	0.96986641	0.9777818	0.74245552	IER3	1

BP	GO:0048193	Golgi vesicle transport	2/340	294/18614	0.97219 24	0.97990 62	0.74404 07	HYOU1/PKDCC	2
BP	GO:0140013	meiotic nuclear division	1/340	196/18614	0.97354 84	0.98101 35	0.74488 15	RPS6KA2	1
BP	GO:0007051	spindle organization	1/340	198/18614	0.97451 6	0.98172 89	0.74542 47	TACC2	1
BP	GO:0009952	anterior/posterior pattern specification	1/340	209/18614	0.97924 03	0.98622 75	0.74884 05	BTG2	1
BP	GO:0031396	regulation of protein ubiquitination	1/340	213/18614	0.98073 25	0.98746 93	0.74978 34	TRIB3	1
BP	GO:00	regulation of	1/340	214/18614	0.981	0.987	0.749	IER3	1

	06282	DNA repair			0885	5669	8574		
BP	GO:1903046	meiotic cell cycle process	1/340	215/18614	0.9814379	0.9876578	0.7499264	RPS6KA2	1
BP	GO:0007033	vacuole organization	1/340	217/18614	0.9821176	0.9878918	0.7501041	SRPX	1
BP	GO:0016236	macroautophagy	2/340	322/18614	0.9821973	0.9878918	0.7501041	HMOX1/DCN	2
BP	GO:0006364	rRNA processing	1/340	218/18614	0.9824481	0.9878918	0.7501041	WDR12	1
BP	GO:0006310	DNA recombination	2/340	332/18614	0.9848426	0.9900383	0.7517339	NFKBIZ/HMGB3	2
BP	GO:00	proteasome-mediat	3/340	439/18614	0.987	0.992	0.75375	TRIB3/DTL/TRIB1	3

	43161	ed ubiquitin-dependent protein catabolic process			7443	6935			
BP	GO:0065004	protein-DNA complex assembly	1/340	242/18614	0.98878861	0.99347485	0.75434461	NFKBIZ	1
BP	GO:0033044	regulation of chromosome organization	1/340	250/18614	0.9903343	0.99478805	0.75533347	PPP1R10	1
BP	GO:0060271	cilium assembly	2/340	367/18614	0.9914186	0.99544454	0.75583995	SPEF2/DNAH5	2

BP	GO:0016072	rRNA metabolic process	1/340	257/18614	0.99152273	0.99544454	0.75583895	WDR12	1
BP	GO:0071824	protein-DNA complex subunit organization	1/340	263/18614	0.99242665	0.99608057	0.75632257	NFKBIZ	1
BP	GO:0016570	histone modification	3/340	475/18614	0.99283612	0.99623344	0.75643387	SDR16C5/APLNR/NFKBIZ	3
BP	GO:0006260	DNA replication	1/340	278/18614	0.99427296	0.99716850	0.75714179	DTL	1
BP	GO:0044782	cilium organization	2/340	396/18614	0.99467774	0.99716850	0.75714179	SPEF2/DNAH5	2

BP	GO:0048285	organelle fission	3/340	497/18614	0.99488666	0.99716985	0.75714479	RPS6KA2/IL1B/DCN	3
BP	GO:0051321	meiotic cell cycle	1/340	284/18614	0.99488874	0.99716985	0.75714479	RPS6KA2	1
BP	GO:0140014	mitotic nuclear division	1/340	286/18614	0.9950753	0.99716985	0.75714479	IL1B	1
BP	GO:0042254	ribosome biogenesis	1/340	298/18614	0.99606066	0.9978995	0.7577703	WDR12	1
BP	GO:0034470	ncRNA processing	2/340	432/18614	0.99707785	0.9986508	0.75827734	WDR12/IL6	2
BP	GO:0000280	nuclear division	2/340	450/18614	0.99978413	0.99991521	0.75865541	RPS6KA2/IL1B	2

BP	GO:0098813	nuclear chromosome segregation	1/340	339/18614	0.998171771	0.999225910	0.758710 1	GEM	1
BP	GO:0016050	vesicle organization	1/340	368/18614	0.998943 1	0.99973	0.75909 29	SRPX	1
BP	GO:0070059	chromosome segregation	1/340	435/18614	0.999701	0.999869 8	0.759199	GEM	1
BP	GO:0022613	ribonucleoprotein complex biogenesis	1/340	475/18614	0.999859 6	0.999869 8	0.759199	WDR12	1
BP	GO:0050907	detection of chemical stimulus	1/340	479/18614	0.999869 8	0.999869 8	0.759199	AZGP1	1

		involved in sensory perception							
CC	GO:0062023	collagen-containing extracellular matrix	36/351	415/19518	5.68E-15	1.75E-12	1.54E-12	FREM1/CXCL12/CTSF/LTBP4/LTBP2/TGFB2/ADAMTS15/ECM2/VCAN/MMRN1/MFAP4/SERPINF2/ANGPTL1/TNR/SERPINA3/HMCN1/CPA3/COL6A6/ORM1/SRPX/RARRES2/SULF1/ABI3BP/DCN/COL8A1/S100A8/ADAMTS9/TNC/LUM/DPT/ASPN/COL14A1/OGN/AZGP1/FGA/POSTN	36
CC	GO:0009897	external side of plasma membrane	35/351	426/19518	7.01E-14	1.08E-11	9.48E-12	CD1C/SCNN1G/CXCL12/CD163/ITGA10/BTN3A3/BTN3A1/ITGA4/CLEC2D/ACE/MS4A1/CD79A/IL2RG/SLAMF7/MS4A2/CD1E/CD83/LY9/IL2RB/VCAM1/CD27/CD69/FCGR2B/CD5/CD3G/ANPEP/CD2/CD3E/AZGP1/CLEC4E/CD3D/FGA/CXCL10/CXCL9/SELE	35
CC	GO:0045121	membrane raft	21/351	323/19518	4.46E-07	3.61E-05	3.17E-05	FXYD1/PTGS2/ANK2/CNTN1/CNR1/CD177/SKAP1/TNR/MS4A1/CD79A/DPP4/HMOX1/MS4A4A/SULF1/ANO1/CD48/PLVAP/CD2/HAS2/SLC6A4/SELE	21
CC	GO:0098857	membrane microdomain	21/351	324/19518	4.69E-07	3.61E-05	3.17E-05	FXYD1/PTGS2/ANK2/CNTN1/CNR1/CD177/SKAP1/TNR/MS4A1/CD79A/DPP4/HMOX1/MS4A4A/SULF1/ANO1/CD48/PLVAP/CD2/HAS2/SLC6A4/SELE	21

CC	GO:0034774	secretory granule lumen	20/351	322/19518	1.71E-06	9.51E-05	8.35E-05	RNASE2/HGF/IDH1/TGFB2/LYZ/MMRN1/SERPINF2/SERPINA3/ISLR/ORM1/CRISPLD2/RARRES2/FGR/LCN2/S100A8/CXCL1/PTX3/S100A12/CHIT1/FGA	20
CC	GO:0060205	cytoplasmic vesicle lumen	20/351	325/19518	1.97E-06	9.51E-05	8.35E-05	RNASE2/HGF/IDH1/TGFB2/LYZ/MMRN1/SERPINF2/SERPINA3/ISLR/ORM1/CRISPLD2/RARRES2/FGR/LCN2/S100A8/CXCL1/PTX3/S100A12/CHIT1/FGA	20
CC	GO:0031983	vesicle lumen	20/351	327/19518	2.16E-06	9.51E-05	8.35E-05	RNASE2/HGF/IDH1/TGFB2/LYZ/MMRN1/SERPINF2/SERPINA3/ISLR/ORM1/CRISPLD2/RARRES2/FGR/LCN2/S100A8/CXCL1/PTX3/S100A12/CHIT1/FGA	20
CC	GO:0042613	MHC class II protein complex	5/351	17/19518	9.47E-06	0.0003645	0.0003202	HLA-DPA1/HLA-DOA/HLA-DOB/HLA-DQA1/HLA-DQB1	5
CC	GO:0031093	platelet alpha granule lumen	8/351	67/19518	2.61E-05	0.0008949	0.000786	HGF/TGFB2/MMRN1/SERPINF2/SERPINA3/ISLR/ORM1/FGA	8
CC	GO:0044853	plasma membrane raft	10/351	114/19518	4.04E-05	0.0012042	0.0010577	FXYD1/PTGS2/CD177/SKAP1/MS4A1/HMOX1/MS4A4A/PLVAP/HAS2/SELE	10

CC	GO:0070820	tertiary granule	12/351	164/19518	4.30E-05	0.00120042	0.00105577	HBB/IDH1/LYZ/CD177/ORM1/FPR1/SLC11A1/FPR2/CXCL1/PTX3/CHIT1/MGAM	12
CC	GO:1904724	tertiary granule lumen	7/351	55/19518	5.54E-05	0.00142413	0.00124584	HBB/IDH1/LYZ/ORM1/CXCL1/PTX3/CHIT1	7
CC	GO:0042611	MHC protein complex	5/351	25/19518	7.22E-05	0.00171711	0.00150029	HLA-DPA1/HLA-DOA/HLA-DOB/HLA-DQA1/HLA-DQB1	5
CC	GO:0030139	endocytic vesicle	17/351	347/19518	0.00019171	0.00433365	0.00380389	SGIP1/CD163/HYOU1/HLA-DPA1/HBB/DPP4/STAB1/CD3G/CFTR/SLC11A1/MARCO/SAA1/CLEC4E/CD3D/HLA-DQA1/CSF3/HLA-DQB1	17
CC	GO:0030666	endocytic vesicle membrane	12/351	196/19518	0.00023345	0.00451552	0.00396658	SGIP1/CD163/HLA-DPA1/STAB1/CD3G/CFTR/SLC11A1/MARCO/CLEC4E/CD3D/HLA-DQA1/HLA-DQB1	12
CC	GO:0031091	platelet alpha granule	8/351	91/19518	0.00023346	0.00451552	0.00396658	HGF/TGFB2/MMRN1/SERPINF2/SERPINA3/ISLR/ORM1/FGA	8

CC	GO:0030669	clathrin-coated endocytic vesicle membrane	7/351	73/19518	0.00033373	0.0060821	0.0053421	SGIP1/HLA-DPA1/CD3G/CFTR/CD3D/HLA-DQA1/HLA-DQB1	7
CC	GO:0005775	vacuolar lumen	11/351	176/19518	0.00035355	0.0060821	0.0053421	RNASE2/CTSF/LYZ/VCAN/CSPG5/SERPINA3/CD1E/DCN/LUM/OGN/CSF3	11
CC	GO:0043202	lysosomal lumen	8/351	98/19518	0.00038396	0.0063162	0.0055477	CTSF/VCAN/CSPG5/CD1E/DCN/LUM/OGN/CSF3	8
CC	GO:0071682	endocytic vesicle lumen	4/351	23/19518	0.00069946	0.0106971	0.0093956	HYOU1/HBB/SAA1/CSF3	4
CC	GO:0005581	collagen trimer	7/351	85/19518	0.0008505	0.0114454	0.0100529	C1QTNF7/GLDN/COL6A6/COL8A1/LUM/COL14A1/MARCO	7
CC	GO:00	specific granule lumen	6/351	62/19518	0.000	0.011	0.010	LYZ/ORM1/LCN2/CXCL1/PTX3/CHIT1	6

	355<br>80				85<br>35	44<br>54	05<br>29		
C<br>C	GO<br>:00<br>056<br>14	interstiti<br>al<br>matrix	3/<br>35<br>1	11/<br>195<br>18	0.0<br>00<br>85<br>47	0.0<br>11<br>44<br>54	0.0<br>10<br>05<br>29	ECM2/TNC/COL14A1	3
C<br>C	GO<br>:00<br>421<br>05	alpha-<br>beta T<br>cell<br>recepto<br>r<br>comple<br>x	3/<br>35<br>1	12/<br>195<br>18	0.0<br>01<br>12<br>45	0.0<br>14<br>43<br>04	0.0<br>12<br>67<br>47	CD3G/CD3E/CD3D	3
C<br>C	GO<br>:00<br>453<br>34	clathrin<br>-coated<br>endocy<br>tic<br>vesicle	7/<br>35<br>1	92/<br>195<br>18	0.0<br>01<br>35<br>67	0.0<br>16<br>71<br>43	0.0<br>14<br>68<br>07	SGIP1/HLA-DPA1/CD3G/CFTR/CD3D/HLA-DQA1/HLA-DQB1	7
C<br>C	GO<br>:00<br>306<br>65	clathrin<br>-coated<br>vesicle<br>membr<br>ane	8/<br>35<br>1	136<br>/19<br>518	0.0<br>03<br>24<br>17	0.0<br>37<br>09<br>02	0.0<br>32<br>57<br>75	SLC18A2/SGIP1/HLA-DPA1/CD3G/CFTR/CD3D/HLA-DQA1/HLA-DQB1	8
C<br>C	GO<br>:00	protein<br>comple<br>x	3/<br>35<br>1	17/<br>195<br>18	0.0<br>03	0.0<br>37	0.0<br>32	MMRN1/TNR/TNC	3

	98637	involved in cell-matrix adhesion			2514	0902	5775		
CC	GO:0098636	protein complex involved in cell adhesion	5/351	57/19518	0.003555596	0.03915559	0.03443919	ITGA10/MMRN1/ITGA4/TNR/TNC	5
CC	GO:0042581	specific granule	8/351	160/19518	0.008505046	0.0903325	0.07933353	LYZ/CD177/ORM1/LCN2/FPR2/CXCL1/PTX3/CHIT1	8
CC	GO:0005796	Golgi lumen	6/351	106/19518	0.01229297	0.1262767	0.1109129	VCAN/CSPG5/DCN/LUM/OGN/MUC15	6
CC	GO:0043292	contractile fiber	10/351	243/19518	0.01287556	0.1279255	0.1123611	RYR2/ENO1/ANK2/TIMP4/CASQ2/CAPN3/KRT19/MYH11/PDLIM3/IDO1	10

CC	GO:0098553	lumenal side of endoplasmic reticulum membrane	3/351	29/19518	0.01490690	0.1434479	0.1260223	HLA-DPA1/HLA-DQA1/HLA-DQB1	3
CC	GO:0072562	blood microparticle	7/351	144/19518	0.01550504	0.1446704	0.1270687	HBB/SERPINF2/SERPINA3/ORM1/C1R/CP/FGA	7
CC	GO:0005640	nuclear outer membrane	3/351	30/19518	0.01634 69	0.1480836	0.1300666	ENO1/PTGS2/DHCR7	3
CC	GO:0005901	caveola	5/351	83/19518	0.01694 42	0.1491086	0.1309669	FXYD1/PTGS2/HMOX1/PLVAP/SELE	5
CC	GO:0001527	microfibril	2/351	13/19518	0.02206 75	0.1887995	0.1658287	LTBP4/MFAP4	2

CC	GO:0101003	ficolin-1-rich granule membrane	4/351	61/19518	0.02404436	0.2001468	0.1757953	FPR1/SLC11A1/FPR2/MGAM	4
CC	GO:0005788	endoplasmic reticulum lumen	11/351	312/19518	0.02626618	0.20986898	0.18433354	HYOU1/PTGS2/VCAN/CASQ2/COL8A1/TNC/COL14A1/CP/IL6/ERP27/FGA	11
CC	GO:0098576	lumenal side of membrane	3/351	36/19518	0.02655744	0.20986898	0.18433354	HLA-DPA1/HLA-DQA1/HLA-DQB1	3
CC	GO:0030018	Z disc	6/351	129/19518	0.02924484	0.2252128	0.19781816	RYR2/ANK2/CASQ2/CAPN3/KRT19/PDLIM3	6
CC	GO:0030662	coated vesicle membrane	8/351	202/19518	0.03012214	0.2262278	0.19874473	SLC18A2/SGIP1/HLA-DPA1/CD3G/CFTR/CD3D/HLA-DQA1/HLA-DQB1	8
CC	GO:0061702	inflammasome	2/351	16/19518	0.03277714	0.24032233	0.21108837	NAIP/NLRC4	2

		complex							
CC	GO:0045178	basal part of cell	10/351	285/19518	0.0340995	0.2442152	0.2145022	P2RY1/ANK2/NAIP/ABCA8/GKN2/ACE/SLC39A14/SLC14A1/AQP9/SLC1A3	10
CC	GO:0042383	sarcolemma	6/351	136/19518	0.03654819	0.2553735	0.2243028	RYR2/FXYD1/ANK2/CAPN3/VCAM1/KRT19	6
CC	GO:0030017	sarcomere	8/351	213/19518	0.0392995	0.2618691	0.230008	RYR2/ENO1/ANK2/TIMP4/CASQ2/CAPN3/KRT19/PDLIM3	8
CC	GO:0043034	costamere	2/351	18/19518	0.04081145	0.2618691	0.230008	ANK2/KRT19	2
CC	GO:0030136	clathrin-coated vesicle	8/351	215/19518	0.04114431	0.2618691	0.230008	SLC18A2/SGIP1/HLA-DPA1/CD3G/CFTR/CD3D/HLA-DQA1/HLA-DQB1	8
CC	GO:0001772	immunological synapse	3/351	43/19518	0.04193913	0.2618691	0.230008	SKAP1/RHOH/CD3E	3

CC	GO:0031674	I band	6/351	141/19518	0.0422941	0.2618691	0.2300008	RYR2/ANK2/CASQ2/CAPN3/KRT19/PDLIM3	6
CC	GO:0070821	tertiary granule membrane	4/351	73/19518	0.04251512	0.2618691	0.2300008	CD177/SLC11A1/FPR2/MGAM	4
CC	GO:0031430	M band	2/351	20/19518	0.04955129	0.2978143	0.2615799	ENO1/ANK2	2
CC	GO:0101002	ficolin-1-rich granule	7/351	185/19518	0.05028803	0.2978143	0.2615799	HBB/IDH1/CRISPLD2/FPR1/SLC11A1/FPR2/MGAM	7
CC	GO:0009925	basal plasma membrane	9/351	266/19518	0.0519984	0.2983813	0.2620779	P2RY1/ANK2/NAIP/ABCA8/ACE/SLC39A14/SLC14A1/AQP9/SLC1A3	9
CC	GO:0043195	terminal bouton	3/351	47/19518	0.05233136	0.2983813	0.2620779	SLC18A2/PRSS12/NTRK2	3

CC	GO:0098644	complex of collagen trimers	2/351	21/19518	0.05409	0.3029041	0.2660504	COL8A1/LUM	2
CC	GO:0030016	myofibril	8/351	233/19518	0.0603944	0.3287209	0.2887261	RYR2/ENO1/ANK2/TIMP4/CASQ2/CAPN3/KRT19/PDLIM3	8
CC	GO:0045177	apical part of cell	13/351	446/19518	0.0608347	0.3287209	0.2887261	P2RY1/SCNN1G/FXYD1/ANK2/DUOX1/DDR2/SLC39A14/DPP4/SLC7A11/VCAM1/ANO1/CFTR/MGAM	13
CC	GO:0098802	plasma membrane signaling receptor	10/351	319/19518	0.0636289	0.3303884	0.2901908	ITGA10/ITGA4/SKAP1/CD79A/TRAT1/IL2RB/CD3G/CD3E/IL6/CD3D	10
CC	GO:0031968	complex organelle outer membrane	8/351	236/19518	0.0640728	0.3303884	0.2901908	ENO1/PTGS2/DHCR7/CNR1/TRAF3IP3/HMOX1/MAOA/PLA2G2A	8

CC	GO:0005604	basement membrane	4/351	84/19518	0.0648538	0.3303884	0.2901908	FREM1/HMCN1/COL8A1/TNC	4
CC	GO:0019867	outer membrane	8/351	238/19518	0.0666009	0.3303884	0.2901908	ENO1/PTGS2/DHCR7/CNR1/TRAF3IP3/HMOX1/MAOA/PLA2G2A	8
CC	GO:0030315	T-tubule	3/351	52/19518	0.0668511	0.3303884	0.2901908	FXYD1/ANK2/CAPN3	3
CC	GO:0016460	myosin II complex	2/351	24/19518	0.0686521	0.3303884	0.2901908	MYH10/MYH11	2
CC	GO:0032982	myosin filament	2/351	24/19518	0.0686521	0.3303884	0.2901908	MYH10/MYH11	2
CC	GO:0005765	lysosomal membrane	12/351	415/19518	0.0730875	0.3410751	0.2995773	HLA-DPA1/HLA-DOA/HLA-DOB/TRAF3IP3/SLC39A14/DPP4/FPR1/ANPEP/CFTR/CP/HLA-DQA1/HLA-DQB1	12

CC	GO:0098852	lytic vacuole membrane	12/351	415/19518	0.0730875	0.3410751	0.2995773	HLA-DPA1/HLA-DOA/HLA-DOB/TRAF3IP3/SLC39A14/DPP4/FPR1/ANPEP/CFTR/CP/HLA-DQA1/HLA-DQB1	12
CC	GO:0031941	filamentous actin	2/351	27/19518	0.0843281	0.382159	0.3356626	DPYSL3/PDLIM3	2
CC	GO:0016324	apical plasma membrane	11/351	381/19518	0.0843728	0.382159	0.3356626	P2RY1/SCNN1G/FXYD1/ANK2/DUOX1/DDR2/SLC39A14/DPP4/ANO1/CFTR/MGAM	11
CC	GO:0090575	RNA polymerase II transcription regulator complex	8/351	254/19518	0.089024	0.3973827	0.349034	NFATC2/STAT4/JUN/JUNB/JUND/HLF/ATF3/FOS	8
CC	GO:0005942	phosphatidylinositol 3-kinase	2/351	29/19518	0.0953188	0.4194029	0.3683751	PIK3CG/SOCS3	2

		complex							
CC	GO:0002102	podosome	2/351	30/19518	0.1009597	0.4370946	0.3839143	SH3PXD2B/VCAM1	2
CC	GO:0012507	ER to Golgi transport vesicle membrane	3/351	63/19518	0.1043733	0.4370946	0.3839143	HLA-DPA1/HLA-DQA1/HLA-DQB1	3
CC	GO:0032421	stereocilium bundle	3/351	63/19518	0.1043733	0.4370946	0.3839143	PCDH15/PKHD1L1/IDO1	3
CC	GO:0015629	actin cytoskeleton	13/351	489/19518	0.10501062	0.4370946	0.3839143	CORIN/CNR1/DPYSL3/DDR2/PLEKHH2/SH3PXD2B/FMN1/FGR/VCAM1/KRT19/MYH10/MYH11/PDLIM3	13
CC	GO:0008305	integrin complex	2/351	31/19518	0.1066906	0.4381425	0.3848347	ITGA10/ITGA4	2

CC	GO:0005790	smooth endoplasmic reticulum	2/351	32/19518	0.112505064	0.455947	0.40004729	RYR2/HYOU1	2
CC	GO:0031672	A band	2/351	33/19518	0.118404023	0.47360692	0.41598862	ENO1/ANK2	2
CC	GO:0042101	T cell receptor complex	5/351	147/19518	0.126262659	0.49858885	0.43792264	SKAP1/TRAT1/CD3G/CD3E/CD3D	5
CC	GO:0005774	vacuolar membrane	12/351	460/19518	0.128000065	0.49906633	0.43834334	HLA-DPA1/HLA-DOA/HLA-DOB/TRAF3IP3/SLC39A14/DPP4/FPR1/ANPEP/CFTR/CP/HLA-DQA1/HLA-DQB1	12
CC	GO:0016323	basolateral plasma membrane	7/351	234/19518	0.130333396	0.50018074	0.44075036	P2RY1/ANK2/NAIP/ABCA8/SLC39A14/SLC14A1/AQP9	7
CC	GO:00	cation channel	6/351	192/19518	0.133	0.507	0.445	TRPC6/RYR2/SCNN1G/KCNAB1/TRPC1/CASQ2	6

	34703	complex			3844	1899	4812		
CC	GO:0030027	lamellipodium	6/351	202/19518	0.1576563	0.5921724	0.5201241	CD177/DPYSL3/PLEKHH2/FGD2/DPP4/MYH10	6
CC	GO:0017053	transcription repressor complex	3/351	78/19518	0.1655308	0.6086392	0.5345874	SDR16C5/JUN/JUND	3
CC	GO:0044224	juxtaparanode region of axon	1/351	10/19518	0.1659925	0.6086392	0.5345874	KCNAB1	1
CC	GO:0098862	cluster of actin-based cell projections	5/351	162/19518	0.1680145	0.6088055	0.5347334	PCDH15/PKHD1L1/ACE/SLC7A11/IDO1	5
CC	GO:00	ion channel	8/351	299/19518	0.172	0.612	0.538	TRPC6/RYR2/SCNN1G/KCNAB1/TRPC1/CASQ2/ANO1/CFTR	8

	34702	complex			313	6103	0753		
CC	GO:0033017	sarcoplasmic reticulum membrane	2/351	42/19518	0.1743331	0.6126103	0.5380753	RYR2/CASQ2	2
CC	GO:0002178	palmitoyltransferase complex	1/351	11/19518	0.1809985	0.6126103	0.5380753	ZDHHC9	1
CC	GO:0031838	haptoglobin-hemoglobin complex	1/351	11/19518	0.1809985	0.6126103	0.5380753	HBB	1
CC	GO:0034663	endoplasmic reticulum chaperone complex	1/351	11/19518	0.1809985	0.6126103	0.5380753	HYOU1	1

		complex							
CC	GO:0036157	outer dynein arm	1/351	11/19518	0.1809985	0.6126103	0.5380753	DNAH5	1
CC	GO:0005791	rough endoplasmic reticulum	3/351	82/19518	0.1833274	0.6137483	0.5390749	SRPRB/PTGDS/SLC7A11	3
CC	GO:0005758	mitochondrial intermembrane space	3/351	84/19518	0.1924098	0.6151679	0.5403217	SHMT2/ALPL/FGR	3
CC	GO:0005583	fibrillar collagen trimer	1/351	12/19518	0.1957352	0.6151679	0.5403217	LUM	1
CC	GO:0005833	hemoglobin complex	1/351	12/19518	0.1957352	0.6151679	0.5403217	HBB	1

CC	GO:0005890	sodium:potassium-exchanging ATPase complex	1/351	12/19518	0.19573 52	0.61516 79	0.54032 17	FXYD1	1
CC	GO:0060198	clathrin-sculpted vesicle	1/351	12/19518	0.19573 52	0.61516 79	0.54032 17	SLC18A2	1
CC	GO:0098643	banded collagen fibril	1/351	12/19518	0.19573 52	0.61516 79	0.54032 17	LUM	1
CC	GO:0030135	coated vesicle	8/351	312/19518	0.20133 32	0.62636 98	0.55016 08	SLC18A2/SGIP1/HLA-DPA1/CD3G/CFTR/CD3D/HLA-DQA1/HLA-DQB1	8
CC	GO:0031464	Cul4A-RING E3 ubiquiti	1/351	13/19518	0.21020 75	0.63536 27	0.55805 95	DTL	1

		n ligase complex							
CC	GO:0030667	secretory granule membrane	8/351	319/19518	0.2177295	0.6353627	0.5580595	SLC18A2/CD177/FPR1/CPE/ANPEP/SLC11A1/FPR2/MGAM	8
CC	GO:0030673	axolemma	1/351	14/19518	0.2244202	0.6353627	0.5580595	ROBO2	1
CC	GO:0031089	platelet dense granule lumen	1/351	14/19518	0.2244202	0.6353627	0.5580595	RARRES2	1
CC	GO:0035578	azurophil granule lumen	3/351	91/19518	0.2250059	0.6353627	0.5580595	RNASE2/LYZ/SERPINA3	3
CC	GO:0014704	intercalated disc	2/351	50/19518	0.22681	0.6353627	0.5580595	FXYD1/ANK2	2

CC	GO:0097225	sperm midpiece	2/351	50/19518	0.22681	0.6353627	0.5580595	SPEF2/ACE	2
CC	GO:0005769	early endosome	10/351	423/19518	0.2326495	0.6353627	0.5580595	ANK2/SLC39A14/FGD2/CD1E/SIGLEC1/VCAM1/RABGAP1L/LIPG/CFTR/NTRK2	10
CC	GO:0150034	distal axon	7/351	277/19518	0.2326546	0.6353627	0.5580595	OLFM1/SLC18A2/PRSS12/ITGA4/CNR1/DPYSL3/NTRK2	7
CC	GO:0005782	peroxisomal matrix	2/351	51/19518	0.2334631	0.6353627	0.5580595	IDH1/EPHX2	2
CC	GO:0031907	microbody lumen	2/351	51/19518	0.2334631	0.6353627	0.5580595	IDH1/EPHX2	2
CC	GO:0030134	COPII-coated ER to Golgi transpo	3/351	93/19518	0.2345124	0.6353627	0.5580595	HLA-DPA1/HLA-DQA1/HLA-DQB1	3

		rt vesicle							
CC	GO:0030133	transport vesicle	10/351	425/19518	0.2369191	0.6353627	0.5580595	SLC18A2/HLA-DPA1/SYTL2/DPYSL3/CPA3/CRISPLD2/PCSK2/CPE/HLA-DQA1/HLA-DQB1	10
CC	GO:0005859	muscle myosin complex	1/351	15/19518	0.2383777	0.6353627	0.5580595	MYH11	1
CC	GO:0072687	meiotic spindle	1/351	15/19518	0.2383777	0.6353627	0.5580595	RPS6KA2	1
CC	GO:0098691	dopaminergic synapse	1/351	15/19518	0.2383777	0.6353627	0.5580595	SLC18A2	1
CC	GO:0031970	organelle envelope lumen	3/351	94/19518	0.2392925	0.6353627	0.5580595	SHMT2/ALPL/FGR	3
CC	GO:00	clathrin coat of	1/351	16/19518	0.252	0.656	0.576	SGIP1	1

	30132	coated pit			0848	0675	2452		
CC	GO:0016459	myosin complex	2/351	54/19518	0.2534806	0.6560675	0.5762452	MYH10/MYH11	2
CC	GO:0032154	cleavage furrow	2/351	54/19518	0.2534806	0.6560675	0.5762452	HMCN1/MYH10	2
CC	GO:0034707	chloride channel complex	2/351	55/19518	0.2601643	0.6629513	0.5822915	ANO1/CFTR	2
CC	GO:0008180	COP9 signalosome	1/351	17/19518	0.2655459	0.6629513	0.5822915	THEMIS	1
CC	GO:0032420	stereocilium	2/351	56/19518	0.2668498	0.6629513	0.5822915	PCDH15/PKHD1L1	2

CC	GO:0031526	brush border membrane	2/351	57/19518	0.27353 47	0.66295 13	0.58229 15	ACE/SLC7A11	2
CC	GO:1902495	transmembrane transporter complex	9/351	392/19518	0.27493 36	0.66295 13	0.58229 15	TRPC6/RYR2/SCNN1G/FXYD1/KCNAB1/TRPC1/CASQ2/ANO1/CFTR	9
CC	GO:0005881	cytoplasmic microtubule	3/351	102/19518	0.27803 71	0.66295 13	0.58229 15	SRPRB/SYBU/SAA1	3
CC	GO:0032588	trans-Golgi network membrane	3/351	102/19518	0.27803 71	0.66295 13	0.58229 15	HLA-DPA1/HLA-DQA1/HLA-DQB1	3
CC	GO:0043020	NADPH oxidase complex	1/351	18/19518	0.27876 54	0.66295 13	0.58229 15	DUOX1	1

CC	GO:0090533	cation-transporting ATPase complex	1/351	18/19518	0.2787654	0.6629513	0.5822915	FXYD1	1
CC	GO:0030660	Golgi-associated vesicle membrane	2/351	58/19518	0.2802169	0.6629513	0.5822915	CSPG5/CFTR	2
CC	GO:0030863	cortical cytoskeleton	3/351	104/19518	0.2878267	0.6629513	0.5822915	PLEKHH2/KRT19/SELE	3
CC	GO:0000164	protein phosphatase type 1 complex	1/351	19/19518	0.2917476	0.6629513	0.5822915	PPP1R10	1
CC	GO:00	manchette	1/351	19/19518	0.291	0.662	0.582	SPEF2	1

	02177				7476	9513	2915		
CC	GO:0030687	preribosome, large subunit precursor	1/351	19/19518	0.2917476	0.6629513	0.5822915	WDR12	1
CC	GO:0034706	sodium channel complex	1/351	19/19518	0.2917476	0.6629513	0.5822915	SCNN1G	1
CC	GO:0097449	astrocyte projection	1/351	19/19518	0.2917476	0.6629513	0.5822915	SLC7A11	1
CC	GO:0005884	actin filament	3/351	105/19518	0.2927317	0.6629513	0.5822915	DPYSL3/FMN1/PDLIM3	3
CC	GO:0005766	primary lysosome	4/351	155/19518	0.3046217	0.6798802	0.5971607	RNASE2/LYZ/SERPINA3/FPR1	4

CC	GO:0042582	azurophil granule	4/351	155/19518	0.3046217	0.6798802	0.5971607	RNASE2/LYZ/SERPINA3/FPR1	4
CC	GO:0032426	stereocilium tip	1/351	21/19518	0.3170172	0.6804413	0.5976535	PKHD1L1	1
CC	GO:0042827	platelet dense granule	1/351	21/19518	0.3170172	0.6804413	0.5976535	RARRES2	1
CC	GO:0043083	synaptic cleft	1/351	21/19518	0.3170172	0.6804413	0.5976535	PRSS12	1
CC	GO:0005741	mitochondrial outer membrane	5/351	208/19518	0.3198646	0.6804413	0.5976535	CNR1/TRAF3IP3/HMOX1/MAOA/PLA2G2A	5
CC	GO:0044304	main axon	2/351	64/19518	0.3201445	0.6804413	0.5976535	KCNAB1/ROBO2	2

CC	GO:0009898	cytoplasmic side of plasma membrane	4/351	159/19518	0.320208541	0.680441 3	0.59765 35	KCNAB1/GEM/FGR/CD2	4
CC	GO:0042470	melanosome	3/351	112/19518	0.327171748	0.68044 13	0.59765 35	SYTL2/FASN/MYH11	3
CC	GO:0048770	pigment granule	3/351	112/19518	0.327171748	0.68044 13	0.59765 35	SYTL2/FASN/MYH11	3
CC	GO:0005858	axonemal dynein complex	1/351	22/19518	0.329313128	0.68044 13	0.59765 35	DNAH5	1
CC	GO:0031258	lamellipodium membrane	1/351	22/19518	0.329313128	0.68044 13	0.59765 35	DPP4	1
CC	GO:00	axon terminus	3/351	114/19518	0.337	0.680	0.597	SLC18A2/PRSS12/NTRK2	3

	43679				0247	4413	6535		
CC	GO:1990351	transporter complex	9/351	418/19518	0.3390069	0.6804413	0.5976535	TRPC6/RYR2/SCNN1G/FXYD1/KCNAB1/TRPC1/CASQ2/ANO1/CFTR	9
CC	GO:0042734	presynaptic membrane	4/351	164/19518	0.3412291	0.6804413	0.5976535	P2RY1/CNTN1/CNR1/SLC6A4	4
CC	GO:0016327	apicolateral plasma membrane	1/351	23/19518	0.3413876	0.6804413	0.5976535	KRT19	1
CC	GO:0031252	cell leading edge	9/351	422/19518	0.3491053	0.6804413	0.5976535	ROBO2/DUOX1/CD177/DPYSL3/PLEKHH2/FGD2/DPP4/FGR/MYH10	9
CC	GO:0001725	stress fiber	2/351	69/19518	0.3530082	0.6804413	0.5976535	MYH10/PDLIM3	2
CC	GO:00	contractile actin	2/351	69/19518	0.353	0.680	0.597	MYH10/PDLIM3	2

	97517	filament bundle			0082	4413	6535		
CC	GO:0030867	rough endoplasmic reticulum membrane	1/351	24/19518	0.353242457	0.680444413	0.597656535	SRPRB	1
CC	GO:0035371	microtubule plus-end	1/351	24/19518	0.353242457	0.680444413	0.597656535	NCKAP5	1
CC	GO:0044295	axonal growth cone	1/351	24/19518	0.353242457	0.680444413	0.597656535	OLFM1	1
CC	GO:0098533	ATPase dependent transmembrane transpo	1/351	24/19518	0.353242457	0.680444413	0.597656535	FXYD1	1

		rt complex							
CC	GO:0030426	growth cone	4/351	167/19518	0.3535476	0.6804413	0.5976535	OLFM1/ITGA4/CNR1/DPYSL3	4
CC	GO:0030131	clathrin adaptor complex	1/351	25/19518	0.3648908	0.6979354	0.6130192	SGIP1	1
CC	GO:0044291	cell-cell contact zone	2/351	72/19518	0.3724634	0.6979354	0.6130192	FXYD1/ANK2	2
CC	GO:0030427	site of polarized growth	4/351	172/19518	0.3738844	0.6979354	0.6130192	OLFM1/ITGA4/CNR1/DPYSL3	4
CC	GO:0005905	clathrin-coated pit	2/351	73/19518	0.3788969	0.6979354	0.6130192	SGIP1/SELE	2
CC	GO:00	sarcoplasmic	2/351	73/19518	0.378	0.697	0.613	RYR2/CASQ2	2

	16529	reticulum			8969	9354	0192		
CC	GO:0030864	cortical actin cytoskeleton	2/351	73/19518	0.3788969	0.6979354	0.6130192	PLEKHH2/KRT19	2
CC	GO:0032153	cell division site	2/351	73/19518	0.3788969	0.6979354	0.6130192	HMCN1/MYH10	2
CC	GO:0045211	postsynaptic membrane	6/351	277/19518	0.3806921	0.6979354	0.6130192	P2RY1/ANK2/CNTN1/CSPG5/LRRTM4/SLC6A4	6
CC	GO:1904813	ficolin-1-rich granule lumen	3/351	124/19518	0.386039	0.7021632	0.6167326	HBB/IDH1/CRISPLD2	3
CC	GO:0034364	high-density lipoprotein particle	1/351	27/19518	0.3875576	0.7021632	0.6167326	SAA1	1
CC	GO:00	actomyosin	2/351	76/19518	0.398	0.709	0.623	MYH10/PDLIM3	2

	42641				0268	386	0766		
CC	GO:0001891	phagocytic cup	1/351	28/19518	0.3985867	0.7093886	0.6230766	CLEC4E	1
CC	GO:0031256	leading edge membrane	4/351	180/19518	0.4064102	0.7093886	0.6230766	ROBO2/FGD2/DPP4/FGR	4
CC	GO:0030686	90S preribosome	1/351	29/19518	0.4094177	0.7093886	0.6230766	WDR12	1
CC	GO:0032809	neuronal cell body membrane	1/351	29/19518	0.4094177	0.7093886	0.6230766	CX3CR1	1
CC	GO:0044306	neuron projection terminus	3/351	129/19518	0.4102519	0.7093886	0.6230766	SLC18A2/PRSS12/NTRK2	3

CC	GO:0030670	phagocytic vesicle membrane	2/351	78/19518	0.41062676	0.7093866	0.62307766	SLC11A1/CLEC4E	2
CC	GO:0032432	actin filament bundle	2/351	78/19518	0.41062676	0.7093866	0.62307766	MYH10/PDLIM3	2
CC	GO:0030658	transport vesicle membrane	5/351	234/19518	0.41233375	0.7093866	0.62307766	SLC18A2/HLA-DPA1/CPE/HLA-DQA1/HLA-DQB1	5
CC	GO:0019898	extrinsic component of membrane	6/351	288/19518	0.41594383	0.7093866	0.62307766	KCNAB1/SYTL2/PIK3CG/FGR/ALOX15B/SOCS3	6
CC	GO:0034704	calcium channel complex	2/351	79/19518	0.41687895	0.7093866	0.62307766	RYR2/CASQ2	2

CC	GO:0031234	extrinsic component of cytoplasmic side of plasma membrane	2/351	80/19518	0.4230978	0.7160116	0.6288961	KCNAB1/FGR	2
CC	GO:0005912	adherens junction	4/351	185/19518	0.4265808	0.7179612	0.6306084	HMCN1/FMN1/ALOX15B/PDLIM3	4
CC	GO:0031901	early endosome membrane	4/351	187/19518	0.434602	0.727486	0.6389743	SLC39A14/FGD2/CFTR/NTRK2	4
CC	GO:0044298	cell body membrane	1/351	32/19518	0.4407575	0.7338016	0.6445216	CX3CR1	1
CC	GO:00	GABA-ergic	2/351	84/19518	0.447	0.741	0.651	CNR1/CSPG5	2

	98982	synapse			6201	2203	0377		
CC	GO:0030125	clathrin vesicle coat	1/351	33/19518	0.4508311	0.7425453	0.6522014	SGIP1	1
CC	GO:0098562	cytoplasmic side of membrane	4/351	193/19518	0.4584686	0.7429471	0.6525544	KCNAB1/GEM/FGR/CD2	4
CC	GO:0005938	cell cortex	6/351	302/19518	0.4604489	0.7429471	0.6525544	ENO1/HMCN1/PLEKHH2/KRT19/MYH10/SELE	6
CC	GO:0048787	presynaptic active zone membrane	1/351	34/19518	0.4607237	0.7429471	0.6525544	P2RY1	1
CC	GO:1990752	microtubule end	1/351	34/19518	0.4607237	0.7429471	0.6525544	NCKAP5	1

CC	GO:0031965	nuclear membrane	6/351	308/19518	0.4792825	0.7461347	0.6553541	ENO1/PTGS2/PTGDS/DHCR7/DTL/NR4A1	6
CC	GO:0016235	aggresome	1/351	36/19518	0.4797791	0.7461347	0.6553541	FGR	1
CC	GO:0034358	plasma lipoprotein particle	1/351	36/19518	0.4797791	0.7461347	0.6553541	SAA1	1
CC	GO:0051233	spindle midzone	1/351	36/19518	0.4797791	0.7461347	0.6553541	GEM	1
CC	GO:0080008	Cul4-RING E3 ubiquitin ligase complex	1/351	36/19518	0.4797791	0.7461347	0.6553541	DTL	1
CC	GO:19	lipoprotein particle	1/351	36/19518	0.479	0.746	0.655	SAA1	1

	90777				9791	1347	3541		
CC	GO:0016528	sarcoplasm	2/351	90/19518	0.4832761	0.7461347	0.6553541	RYR2/CASQ2	2
CC	GO:0005802	trans-Golgi network	5/351	255/19518	0.4858004	0.7461347	0.6553541	HLA-DPA1/PCSK2/HLA-DQA1/HLA-DQB1/POSTN	5
CC	GO:0005798	Golgi-associated vesicle	2/351	91/19518	0.4890799	0.7461347	0.6553541	CSPG5/CFTR	2
CC	GO:0035579	specific granule membrane	2/351	91/19518	0.4890799	0.7461347	0.6553541	CD177/FPR2	2
CC	GO:0030119	AP-type membrane coat adaptor complex	1/351	37/19518	0.4893481	0.7461347	0.6553541	SGIP1	1

CC	GO:0019897	extrinsic component of plasma membrane	3/351	147/19518	0.494942	0.750267	0.6589837	KCNAB1/SYTL2/FGR	3
CC	GO:0097386	glial cell projection	1/351	38/19518	0.4985488	0.7527109	0.6611302	SLC7A11	1
CC	GO:0032994	protein-lipid complex	1/351	39/19518	0.5075842	0.7626143	0.6698287	SAA1	1
CC	GO:0001750	photoreceptor outer segment	2/351	95/19518	0.5118819	0.765338	0.672221	PCDH15/VCAN	2
CC	GO:0030055	cell-substrate junction	8/351	432/19518	0.5165482	0.7685838	0.6750719	HYOU1/ITGA4/DDR2/HMCN1/DPP4/TNC/ALOX15B/SLC6A4	8

CC	GO:0098791	Golgi apparatus subcompartment	7/351	378/19518	0.52202 19	0.772994	0.6789455	HLA-DPA1/FUT3/SULF1/PCSK2/HLA-DQA1/HLA-DQB1/POSTN	7
CC	GO:0000932	P-body	2/351	98/19518	0.52853 96	0.77890 04	0.68413 33	PNRC1/ZFP36	2
CC	GO:0032587	ruffle membrane	2/351	101/19518	0.54480 82	0.79718 82	0.70019 61	FGD2/FGR	2
CC	GO:0043025	neuronal cell body	9/351	500/19518	0.54612 57	0.79718 82	0.70019 61	PLXDC1/OLFM1/TGFB2/ITGA4/NQO1/PCSK2/SLC1A3/CX3CR1/CX3CL1	9
CC	GO:0097060	synaptic membrane	7/351	390/19518	0.55518 12	0.80006 54	0.70272 32	P2RY1/ANK2/CNTN1/CNR1/CSPG5/LRRTM4/SLC6A4	7
CC	GO:0005811	lipid droplet	2/351	103/19518	0.55543 51	0.80006 54	0.70272 32	PLIN2/SDR16C5	2

CC	GO:0009295	nucleoid	1/351	45/19518	0.558487 2	0.800065 4	0.702723 2	SHMT2	1
CC	GO:0042645	mitochondrial nucleoid	1/351	45/19518	0.558487 2	0.800065 4	0.702723 2	SHMT2	1
CC	GO:0005903	brush border	2/351	105/19518	0.565885 2	0.806910 4	0.708735 4	ACE/SLC7A11	2
CC	GO:0030118	clathrin coat	1/351	47/19518	0.574260 7	0.815079 6	0.715910 7	SGIP1	1
CC	GO:0097729	9+2 motile cilium	3/351	167/19518	0.580527 2	0.820194 4	0.720403 1	SPEF2/ACE/DNAH5	3
CC	GO:0031253	cell projection membrane	6/351	349/19518	0.601124 5	0.845417	0.742557	ROBO2/ACE/FGD2/DPP4/SLC7A11/FGR	6

CC	GO:0008287	protein serine/threonine phosphatase complex	1/351	52/19518	0.611279 1	0.8532762	0.7494 6	PPP1R10	1
CC	GO:0043197	dendritic spine	3/351	175/19518	0.6122534	0.8532762	0.7494 6	FCGR2B/CD3E/NTRK2	3
CC	GO:0005770	late endosome	5/351	296/19518	0.6178627	0.8557903	0.7516682	SLC39A14/CD79A/CD1E/SIGLEC1/SLC11A1	5
CC	GO:0044309	neuron spine	3/351	178/19518	0.623729	0.8557903	0.7516682	FCGR2B/CD3E/NTRK2	3
CC	GO:0030286	dynein complex	1/351	54/19518	0.6251715	0.8557903	0.7516682	DNAH5	1

CC	GO:1903293	phosphatase complex	1/351	54/19518	0.6251715	0.8557903	0.7516682	PPP1R10	1
CC	GO:0005925	focal adhesion	7/351	422/19518	0.6381168	0.8664424	0.7610243	HYOU1/ITGA4/DDR2/DPP4/TNC/ALOX15B/SLC6A4	7
CC	GO:0097733	photoreceptor cell cilium	2/351	120/19518	0.6385793	0.8664424	0.7610243	PCDH15/VCAN	2
CC	GO:0035577	azurophil granule membrane	1/351	58/19518	0.6514884	0.7887859	0.771945	FPR1	1
CC	GO:0001533	cornified envelope	1/351	59/19518	0.6577745	0.7887859	0.771945	PI3	1
CC	GO:0000791	euchromatin	1/351	60/19518	0.6639476	0.7887859	0.771945	JUN	1

CC	GO:0030120	vesicle coat	1/351	60/19518	0.66394476	0.8788759	0.7719945	SGIP1	1
CC	GO:0032589	neuron projection membrane	1/351	60/19518	0.66394476	0.8788759	0.7719945	ROBO2	1
CC	GO:0140534	endoplasmic reticulum protein-containing complex	2/351	126/19518	0.66486639	0.8788759	0.7719945	HYOU1/SRPRB	2
CC	GO:1904115	axon cytoplasm	1/351	63/19518	0.6818084	0.8974423	0.78823355	SYBU	1
CC	GO:0005637	nuclear inner membrane	1/351	64/19518	0.68754591	0.89997732	0.79047555	PTGS2	1

CC	GO:0097731	9+0 non-motile cilium	2/351	132/19518	0.6895899	0.8999732	0.7904755	PCDH15/VCAN	2
CC	GO:0005771	multivesicular body	1/351	67/19518	0.7041589	0.9112644	0.8003929	CD79A	1
CC	GO:0005844	polysome	1/351	67/19518	0.7041589	0.9112644	0.8003929	MYH10	1
CC	GO:0045335	phagocytic vesicle	2/351	141/19518	0.7238482	0.9328253	0.8193305	SLC11A1/CLEC4E	2
CC	GO:0005777	peroxisome	2/351	143/19518	0.7310142	0.9342422	0.820575	IDH1/EPHX2	2
CC	GO:0042579	microbody	2/351	143/19518	0.7310142	0.9342422	0.820575	IDH1/EPHX2	2

CC	GO:0016234	inclusion body	1/351	74/19518	0.7395706	0.9412717	0.8267492	FGR	1
CC	GO:0030684	preribosome	1/351	76/19518	0.7488885	0.9492084	0.8337203	WDR12	1
CC	GO:0008076	voltage-gated potassium channel complex	1/351	78/19518	0.7578738	0.950866	0.8351762	KCNAB1	1
CC	GO:0005795	Golgi stack	2/351	151/19518	0.758118	0.950866	0.8351762	FUT3/SULF1	2
CC	GO:0036126	sperm flagellum	2/351	153/19518	0.764515	0.950866	0.8351762	SPEF2/ACE	2

CC	GO:0031902	late endosome membrane	2/351	155/19518	0.77076649	0.9508866	0.8351762	SLC39A14/SLC11A1	2
CC	GO:0070382	exocytic vesicle	3/351	225/19518	0.77293398	0.9508866	0.8351762	SLC18A2/SYTL2/DPYSL3	3
CC	GO:0098858	actin-based cell projection	3/351	225/19518	0.77293398	0.9508866	0.8351762	PCDH15/PKHD1L1/VCAM1	3
CC	GO:0043204	perikaryon	2/351	156/19518	0.77383544	0.9508866	0.8351762	OLFM1/PCSK2	2
CC	GO:0048786	presynaptic active zone	1/351	82/19518	0.77489841	0.9508866	0.8351762	P2RY1	1
CC	GO:0010494	cytoplasmic stress granule	1/351	85/19518	0.78687715	0.96173318	0.844472	ZFP36	1

CC	GO:0034705	potassium channel complex	1/351	89/19518	0.80185 86	0.96503 36	0.84762 01	KCNAB1	1
CC	GO:0045171	intercellular bridge	1/351	89/19518	0.80185 86	0.96503 36	0.84762 01	GSTM5	1
CC	GO:0120111	neuron projection cytoplasm	1/351	89/19518	0.80185 86	0.96503 36	0.84762 01	SYBU	1
CC	GO:0097730	non-motile cilium	2/351	166/19518	0.80261 53	0.96503 36	0.84762 01	PCDH15/VCAN	2
CC	GO:0030117	membrane coat	1/351	92/19518	0.81240 5	0.96503 36	0.84762 01	SGIP1	1
CC	GO:00	coated membrane	1/351	92/19518	0.812	0.965	0.847	SGIP1	1

	48475				0.405	0336	6201		
CC	GO:0032580	Golgi cisterna membrane	1/351	93/19518	0.8157946	0.9650336	0.8476201	FUT3	1
CC	GO:0005902	microvillus	1/351	94/19518	0.8191231	0.9650336	0.8476201	VCAM1	1
CC	GO:0098685	Schaffer collateral - CA1 synapse	1/351	94/19518	0.8191231	0.9650336	0.8476201	TNR	1
CC	GO:0031514	motile cilium	3/351	248/19518	0.826213	0.9650336	0.8476201	SPEF2/ACE/DNAH5	3
CC	GO:0045095	keratin filament	1/351	97/19518	0.828753	0.9650336	0.8476201	KRT7	1

CC	GO:0036464	cytoplasmic ribonucleoprotein granule	3/351	253/19518	0.836273 4	0.965033 6	0.847620 1	PNRC1/NFKBIZ/ZFP36	3
CC	GO:0045111	intermediate filament cytoskeleton	3/351	253/19518	0.836273 4	0.965033 6	0.847620 1	KRT7/KRT19/S100A8	3
CC	GO:0072686	mitotic spindle	2/351	180/19518	0.837442 4	0.965033 6	0.847620 1	GEM/TRAT1	2
CC	GO:0005874	microtubule	6/351	461/19518	0.839570 3	0.965033 6	0.847620 1	NCKAP5/SRPRB/SYBU/TPPP3/SAA1/DNAH5	6
CC	GO:0001726	ruffle	2/351	181/19518	0.839704 6	0.965033 6	0.847620 1	FGD2/FGR	2

CC	GO:0055038	recycling endosome membrane	1/351	102/19518	0.8436801	0.965998	0.84846672	CFTR	1
CC	GO:0019814	immunoglobulin complex	1/351	108/19518	0.85988896	0.9772915	0.85838366	CD79A	1
CC	GO:0030175	filopodium	1/351	108/19518	0.85988896	0.9772915	0.85838366	VCAM1	1
CC	GO:0035770	ribonucleoprotein granule	3/351	271/19518	0.8684443	0.97858824	0.85952204	PNRC1/NFKBIZ/ZFP36	3
CC	GO:0034451	centriolar satellite	1/351	115/19518	0.87669041	0.97858824	0.85952204	TRAT1	1
CC	GO:00	recycling	2/351	200/19518	0.877	0.978	0.859	ANK2/CFTR	2

	55037	endosome			598	5824	5204		
CC	GO:0005635	nuclear envelope	6/351	490/19518	0.87782556	0.97858524	0.8595204	ENO1/PTGS2/PTGDS/DHCR7/DTL/NR4A1	6
CC	GO:0005776	autophagosome	1/351	117/19518	0.88111151	0.97858524	0.8595204	SRPX	1
CC	GO:0030496	midbody	2/351	203/19518	0.88276671	0.97858524	0.8595204	GEM/MYH10	2
CC	GO:0031985	Golgi cisterna	1/351	118/19518	0.88326659	0.97858524	0.8595204	FUT3	1
CC	GO:0030672	synaptic vesicle membrane	1/351	125/19518	0.8972736	0.98249797	0.86296611	SLC18A2	1
CC	GO:00	exocytic vesicle	1/351	125/19518	0.897	0.982	0.862	SLC18A2	1

	99501	membrane			2736	4997	9611		
CC	GO:0005923	bicellular tight junction	1/351	126/19518	0.89991329	0.98249497	0.86296611	PLXDC1	1
CC	GO:0005882	intermediate filament	2/351	214/19518	0.90000426	0.98249497	0.86296611	KRT7/KRT19	2
CC	GO:1990204	oxidoreductase complex	1/351	128/19518	0.90275514	0.98249497	0.86296611	DUOX1	1
CC	GO:0070160	tight junction	1/351	131/19518	0.90793778	0.98301113	0.86341105	PLXDC1	1
CC	GO:0005793	endoplasmic reticulum-Golgi intermediate	1/351	132/19518	0.90960046	0.98301113	0.86341105	ANPEP	1

		compartment							
CC	GO:0032993	protein-DNA complex	2/351	225/19518	0.9149925	0.9853039	0.8654241	JUND/FOS	2
CC	GO:0043296	apical junction complex	1/351	150/19518	0.93495	0.9933119	0.8724578	PLXDC1	1
CC	GO:0032838	plasma membrane bounded cell projection cytoplasm	2/351	244/19518	0.9358498	0.9933119	0.8724578	SYBU/DNAH5	2
CC	GO:0098978	glutamatergic synapse	4/351	410/19518	0.9392842	0.9933119	0.8724578	P2RY1/CNR1/CSPG5/TNR	4
CC	GO:00	microtubule associa	1/351	156/19518	0.941	0.993	0.872	DNAH5	1

	05875	ted complex			7113	3119	4578		
CC	GO:0005930	axoneme	1/351	156/19518	0.9417113	0.9933119	0.8724578	DNAH5	1
CC	GO:0097014	ciliary plasm	1/351	156/19518	0.9417113	0.9933119	0.8724578	DNAH5	1
CC	GO:0000781	chromosome, telomeric region	1/351	169/19518	0.9540523	0.9992147	0.8776425	PPP1R10	1
CC	GO:0031461	cullin-RING ubiquitin ligase complex	1/351	177/19518	0.9603131	0.9992147	0.8776425	DTL	1
CC	GO:0099568	cytoplasmic region	2/351	283/19518	0.964537	0.9992147	0.8776425	SYBU/DNAH5	2

CC	GO:0061695	transferase complex, transferring phosphorus-containing groups	2/351	306/19518	0.975171773	0.999192147	0.877766425	PIK3CG/SOCS3	2
CC	GO:0008021	synaptic vesicle	1/351	210/19518	0.978323256	0.999192147	0.877766425	SLC18A2	1
CC	GO:0016607	nuclear speck	3/351	415/19518	0.980848419	0.999192147	0.877766425	NFKBIZ/PYHIN1/NR4A2	3
CC	GO:0005819	spindle	3/351	421/19518	0.982824132	0.999192147	0.877766425	RPS6KA2/GEM/TRAT1	3
CC	GO:00	nuclear chromosome	1/351	223/19518	0.982	0.999	0.877	JUN	1

	00228				926	2147	6425		
CC	GO:0014069	postsynaptic density	2/351	339/19518	0.9852381	0.9992147	0.8776425	P2RY1/NTRK2	2
CC	GO:0032279	asymmetric synapse	2/351	353/19518	0.9881888	0.9992147	0.8776425	P2RY1/NTRK2	2
CC	GO:0099572	postsynaptic specialization	2/351	364/19518	0.9900967	0.9992147	0.8776425	P2RY1/NTRK2	2
CC	GO:0098984	neuron to neuron synapse	2/351	382/19518	0.9925899	0.9992147	0.8776425	P2RY1/NTRK2	2
CC	GO:0005759	mitochondrial matrix	3/351	484/19518	0.9929766	0.9992147	0.8776425	SHMT2/PYCR1/MTHFD2	3
CC	GO:00	ubiquitin ligase	1/351	307/19518	0.996	0.999	0.877	DTL	1

	00151	complex			3597	2147	6425		
CC	GO:0005743	mitochondrial inner membrane	2/351	497/19518	0.9988867	0.9992147	0.8776425	SHMT2/FGR	2
CC	GO:0098687	chromosomal region	1/351	390/19518	0.9992147	0.9992147	0.8776425	PPP1R10	1
MF	GO:0005201	extracellular matrix structural constituent	19/347	167/18369	4.15E-10	2.40E-07	2.02E-07	LTBP4/LTBP2/VCAN/MMRN1/MFAP4/HMCN1/COL6A6/SRPX/ABI3BP/DCN/COL8A1/TNC/LUM/DPT/ASPN/COL14A1/OGN/FGA/POSTN	19
MF	GO:0008009	chemokine activity	11/347	49/18369	1.43E-09	4.15E-07	3.48E-07	CXCL12/CCL5/CCL23/CXCL1/CXCL2/CX3CL1/CCL2/CXCL10/CCL8/CXCL9/CCL18	11
MF	GO:0042379	chemokine recepto	12/347	74/18369	1.31E-08	2.53E-06	2.13E-06	CXCL12/CCL5/CCL23/CXCL1/CXCL2/CX3CR1/CX3CL1/CCL2/CXCL10/CCL8/CXCL9/CCL18	12

		r binding							
MF	GO:0005125	cytokine activity	20/347	235/18369	2.30E-08	3.33E-06	2.79E-06	CXCL12/BMP5/TGFB2/CCL5/BMP1/TNFRSF11B/IL1B/CCL23/INHBA/TNFSF10/IL6/CXCL1/CXCL2/CX3CL1/CCL2/CSF3/CXCL10/CCL8/CXCL9/CCL18	20
MF	GO:0048018	receptor ligand activity	28/347	497/18369	2.90E-07	3.36E-05	2.82E-05	CXCL12/BMP5/HGF/TGFB2/SEMA3D/CSPG5/CCL5/SEMA6D/BMP1/TNFRSF11B/DPP4/IL1B/CCL23/INHBA/SEMA6A/TNFSF10/OGN/IL6/CXCL1/CXCL2/CX3CL1/SEMA3G/CCL2/CSF3/CXCL10/CCL8/CXCL9/CCL18	28
MF	GO:0005539	glycosaminoglycan binding	18/347	232/18369	4.66E-07	4.49E-05	3.78E-05	LTBP4/LTBP2/ADAMTS15/ECM2/VCAN/DPYSL3/SLIT3/CRISPLD2/CCL23/STAB1/SULF1/LYVE1/DCN/LIPG/SAA1/CXCL10/CCL8/POSTN	18
MF	GO:0032395	MHC class II receptor activity	5/347	10/18369	5.45E-07	4.51E-05	3.79E-05	HLA-DPA1/HLA-DOA/HLA-DOB/HLA-DQA1/HLA-DQB1	5
MF	GO:0045236	CXCR chemokine receptor binding	6/347	18/18369	6.67E-07	4.83E-05	4.05E-05	CXCL12/CXCL1/CXCL2/CX3CL1/CXCL10/CXCL9	6

MF	GO:0140375	immune receptor activity	13/347	141/18369	2.80E-06	0.00018 03	0.00015 15	HLA-DPA1/IL17RB/HLA-DOA/HLA-DOB/IL2RG/IL2RB/IL1R2/FPR1/FCGR2B/FPR2/HLA-DQA1/CX3CR1/HLA-DQB1	13
MF	GO:0005126	cytokine receptor binding	18/347	270/18369	4.11E-06	0.00023 81	0.0002	CXCL12/TGFB2/CCL5/STAP1/IL1B/CCL23/TNFSF10/IL6/CXCL1/CXCL2/CX3CR1/CX3CL1/CCL2/CSF3/CXCL10/CCL8/CXCL9/CCL18	18
MF	GO:0023026	MHC class II protein complex binding	6/347	27/18369	9.21E-06	0.00048 49	0.00040 73	HLA-DPA1/HLA-DOA/HLA-DOB/MS4A1/HLA-DQA1/HLA-DQB1	6
MF	GO:0001664	G protein-coupled receptor binding	17/347	289/18369	3.82E-05	0.00184 4	0.00154 88	P2RY1/CXCL12/CCL5/ACE/CCL23/FPR1/MARCO/SAA1/CXCL1/CXCL2/CX3CR1/CX3CL1/CCL2/CXCL10/CCL8/CXCL9/CCL18	17
MF	GO:00	extracellular matrix	5/347	22/18369	4.72E	0.002	0.001	VCAN/DCN/LUM/ASPN/OGN	5

	30021	structural constituent conferring compression resistance			-05	104	7672		
MF	GO:0023023	MHC protein complex binding	6/347	36/18369	5.25E-05	0.00021722	0.00018245	HLA-DPA1/HLA-DOA/HLA-DOB/MS4A1/HLA-DQA1/HLA-DQB1	6
MF	GO:0045499	chemorepellent activity	5/347	28/18369	0.00001606	0.00062004	0.00052079	SEMA3D/SEMA6D/DPP4/SEMA6A/SEMA3G	5
MF	GO:0008083	growth factor activity	11/347	160/18369	0.00002366	0.00085621	0.00071915	CXCL12/BMP5/HGF/TGFB2/CSPG5/BMP1/INHBA/OGN/IL6/CXCL1/CSF3	11
MF	GO:00	CCR chemokine	6/347	50/18369	0.000	0.011	0.009	CCL5/CCL23/CX3CL1/CCL2/CCL8/CCL18	6

	48020	receptor binding			3432	5881	7331		
MF	GO:0008201	heparin binding	11/347	168/18369	0.0003603	0.0115881	0.0097331	LTBP2/ADAMTS15/ECM2/SLIT3/CRISPLD2/CCL23/LIPG/SAA1/CXCL10/CCL8/POSTN	11
MF	GO:0005178	integrin binding	10/347	151/18369	0.0006053	0.018258	0.0153353	CXCL12/LTBP4/ITGA10/ECM2/ITGA4/CD177/IL1B/VCAM1/ICAM4/CX3CL1	10
MF	GO:1901681	sulfur compound binding	14/347	269/18369	0.0006307	0.018258	0.0153353	RYR2/LTBP2/CBS/ADAMTS15/ECM2/DPYSL3/SLIT3/CRISPLD2/CCL23/LIPG/SAA1/CXCL10/CCL8/POSTN	14
MF	GO:0050786	RAGE receptor binding	3/347	10/18369	0.0007267	0.0200335	0.0168279	FPR1/S100A8/S100A12	3
MF	GO:0030215	semaphorin receptor binding	4/347	23/18369	0.0008341	0.0219524	0.0184383	SEMA3D/SEMA6D/SEMA6A/SEMA3G	4

MF	GO:0035259	nuclear glucocorticoid receptor binding	3/347	13/18369	0.0016604	0.0417994	0.0351083	NR4A3/NR4A1/NR4A2	3
MF	GO:0016628	oxidoreductase activity, acting on the CH-CH group of donors, NAD or NADP as acceptor	4/347	28/18369	0.0017906	0.0431976	0.0362827	AKR1C2/DHCR7/FASN/AKR1C3	4
MF	GO:0005044	scavenger receptor activity	5/347	48/18369	0.0020559	0.0476146	0.0399927	CD163/PRSS12/STAB1/CD5/MARCO	5

MF	GO:0001228	DNA-binding transcription activator activity, RNA polymerase II-specific	18/347	468/18369	0.0036106	0.0804044	0.0675335	NFATC2/EGR1/JUN/EGR2/EGR3/FOSB/JUNB/JUND/HLF/NR4A3/TFEC/ETV5/ATF3/FOS/TFCP2L1/IRF1/NR4A1/NR4A2	18
MF	GO:0001216	DNA-binding transcription activator activity	18/347	472/18369	0.0039438	0.0814661	0.0684253	NFATC2/EGR1/JUN/EGR2/EGR3/FOSB/JUNB/JUND/HLF/NR4A3/TFEC/ETV5/ATF3/FOS/TFCP2L1/IRF1/NR4A1/NR4A2	18
MF	GO:0030170	pyridoxal phosphate binding	5/347	56/18369	0.0040573	0.0814661	0.0684253	PSAT1/SHMT2/CBS/HDC/CTH	5
MF	GO:00	amide binding	16/347	400/18369	0.004	0.081	0.068	RYR2/CD1C/HLA-DPA1/TGFB2/NQO2/FASN/CD1E/FCGR2B/SLC1A3/INHBA/ANPEP/MARCO/FPR2/FKBP5/HLA-DQA1/HLA-DQB1	16

	33218				0803	4661	4253		
MF	GO:0008237	metallopeptidase activity	10/347	197/18369	0.0043565	0.0818071	0.0687118	AGBL1/STAMBPL1/ADAMTS15/BMP1/CPA3/ACE/ADAM28/ADAMTS9/CPE/ANPEP	10
MF	GO:0070279	vitamin B6 binding	5/347	57/18369	0.00438	0.0818071	0.0687118	PSAT1/SHMT2/CBS/HDC/CTH	5
MF	GO:0038024	cargo receptor activity	6/347	83/18369	0.0048504	0.0877626	0.0737139	CD163/PRSS12/STAB1/LYVE1/CD5/MARCO	6
MF	GO:0030246	carbohydrate binding	12/347	273/18369	0.0059381	0.1041096	0.0874441	FREM1/SIGLEC10/CNTN1/VCAN/CLEC2D/SIGLEC1/CD69/KLRG1/CLEC4E/PTX3/MGAM/SELE	12
MF	GO:0042056	chemoattractant activity	4/347	39/18369	0.0061135	0.1041096	0.0874441	HGF/CCL5/CX3CL1/CXCL10	4
MF	GO:0043177	organic acid binding	10/347	212/18369	0.0072167	0.1175502	0.0987332	RYR2/SHMT2/AKR1C2/HBB/PTGDS/SIGLEC10/AKR1C3/S100A8/SLC1A3/SELE	10

MF	GO:0042169	SH2 domain binding	4/347	41/18369	0.0073088	0.1175502	0.0987332	SIGLEC10/SKAP1/LAX1/SH3PXD2B	4
MF	GO:0019956	chemokine binding	3/347	22/18369	0.007885	0.1233892	0.1036375	ITGA4/ZFP36/CX3CR1	3
MF	GO:0005518	collagen binding	5/347	66/18369	0.00815	0.1241804	0.1043302	ITGA10/ECM2/DDR2/LUM/COL14A1	5
MF	GO:0016616	oxidoreductase activity, acting on the CH-OH group of donors, NAD or NADP as	7/347	123/18369	0.0088719	0.1292399	0.1085516	AKR1C2/KCNAB1/IDH1/PHGDH/SDR16C5/FASN/AKR1C3	7

MF	GO:0015296	acceptor or monoatomic anion: monoatomic cation symporter activity	3/347	23/18369	0.00894 26	0.12923 99	0.10855 16	SLC18A2/SLC39A14/SLC6A4	3
MF	GO:0008235	metalloexopeptidase activity	5/347	68/18369	0.00922 16	0.12923 99	0.10855 16	AGBL1/CPA3/ACE/CPE/ANPEP	5
MF	GO:0019210	kinase inhibitor activity	4/347	44/18369	0.00937 49	0.12923 99	0.10855 16	TRIB3/RHOH/TRIB1/SOCS3	4
MF	GO:0005540	hyaluronic acid binding	3/347	24/18369	0.01007 92	0.12968 62	0.10892 65	VCAN/STAB1/LYVE1	3
MF	GO:00	oxidoreductase	3/347	24/18369	0.010	0.129	0.108	PTGS2/ALOX15B/IDO1	3

	16702	activity, acting on single donors with incorporation of molecular oxygen, incorporation of two atoms of oxygen			0792	6862	9265		
MF	GO:0019865	immunoglobulin binding	3/347	24/18369	0.0100792	0.1296862	0.1089265	MS4A1/MS4A2/FCGR2B	3
MF	GO:00	nuclear recepto	4/347	46/18369	0.010	0.132	0.110	NR4A3/NR1D2/NR4A1/NR4A2	4

	04879	r activity			9437	0087	8772		
MF	GO:0042887	amide transmembrane transporter activity	4/347	46/18369	0.0109437	0.1320087	0.1108772	SLC7A11/SLC14A1/AQP9/SLC1A3	4
MF	GO:0098531	ligand-activated transcription factor activity	4/347	46/18369	0.0109437	0.1320087	0.1108772	NR4A3/NR1D2/NR4A1/NR4A2	4
MF	GO:0016701	oxidoreductase activity, acting on single donors with incorpo	3/347	25/18369	0.0112296	0.1334767	0.1121102	PTGS2/ALOX15B/IDO1	3

		ration of molecular oxygen							
MF	GO:0004180	carboxypeptidase activity	4/347	47/18369	0.0117878	0.1341082	0.1126406	AGBL1/CPA3/ACE/CPE	4
MF	GO:0019955	cytokine binding	7/347	130/18369	0.0118126	0.1341082	0.1126406	LTBP4/ITGA4/IL2RG/ZFP36/IL2RB/IL1R2/CX3CR1	7
MF	GO:0004364	glutathione transferase activity	3/347	26/18369	0.01259337	0.1402256	0.1177788	GSTM5/HPGDS/GSTT1	3
MF	GO:0008238	exopeptidase activity	6/347	102/18369	0.01287821	0.1406219	0.1181117	AGBL1/CPA3/ACE/DPP4/CPE/ANPEP	6
MF	GO:0016614	oxidoreductase activity,	7/347	133/18369	0.01326671	0.1422525	0.1194812	AKR1C2/KCNAB1/IDH1/PHGDH/SDR16C5/FASN/AKR1C3	7

		acting on CH-OH group of donors							
MF	GO:0140359	ABC-type transporter activity	4/347	49/18369	0.0135985	0.1431553	0.1202395	ABCA9/ABCA6/ABCA8/CFTR	4
MF	GO:0015279	store-operated calcium channel activity	2/347	10/18369	0.0144853	0.1446034	0.1214558	TRPC6/TRPC1	2
MF	GO:0032052	bile acid binding	2/347	10/18369	0.0144853	0.1446034	0.1214558	AKR1C2/AKR1C3	2
MF	GO:0071723	lipopeptide binding	2/347	10/18369	0.0144853	0.1446034	0.1214558	CD1C/CD1E	2

MF	GO:0004175	endopeptidase activity	15/347	428/18369	0.01655515	0.1579112	0.13263334	CORIN/CTSF/PRSS12/ADAMTS15/BMP1/ACE/PGC/CTSE/DPP4/CAPN3/ADAM28/GZMK/PCSK2/ADAMTS9/C1R	15
MF	GO:0030159	signaling receptor complex adaptor activity	4/347	52/18369	0.01666304	0.1579112	0.13263334	STAP1/TRAT1/CD3G/CD3E	4
MF	GO:0008504	monoamine transmembrane transporter activity	2/347	11/18369	0.01774853	0.1579112	0.13263334	SLC18A2/SLC6A4	2
MF	GO:0016661	oxidoreductase activity, acting on	2/347	11/18369	0.01774853	0.1579112	0.13263334	CBS/NQO2	2

		other nitrogenous compounds as donors							
MF	GO:0042608	T cell receptor binding	2/347	11/18369	0.0174853	0.1579112	0.1326334	CD3G/CD3E	2
MF	GO:0046790	virion binding	2/347	11/18369	0.0174853	0.1579112	0.1326334	SIGLEC1/PTX3	2
MF	GO:0050661	NADP binding	4/347	53/18369	0.0177275	0.1579112	0.1326334	KCNAB1/IDH1/DUOX1/DHCR7	4
MF	GO:0004033	aldo-keto reductase (NADP) activity	3/347	30/18369	0.0186081	0.1621462	0.1361904	AKR1C2/KCNAB1/AKR1C3	3

MF	GO:0016209	antioxidant activity	5/347	82/18369	0.019527 8	0.162146 2	0.136190 4	HBB/PTGS2/DUOX1/NQO1/GSTT1	5
MF	GO:0004601	peroxidase activity	4/347	55/18369	0.020054 2	0.162146 2	0.136190 4	HBB/PTGS2/DUOX1/GSTT1	4
MF	GO:0004181	metallocarboxypeptidase activity	3/347	31/18369	0.020319 8	0.162146 2	0.136190 4	AGBL1/CPA3/CPE	3
MF	GO:0004032	alditol:NADP+ 1-oxidoreductase activity	2/347	12/18369	0.020723 4	0.162146 2	0.136190 4	AKR1C2/AKR1C3	2
MF	GO:0004875	complement receptor activity	2/347	12/18369	0.020723 4	0.162146 2	0.136190 4	FPR1/FPR2	2

MF	GO:0005527	macrolide binding	2/347	12/18369	0.0207234	0.1621462	0.1361904	LCN2/FKBP5	2
MF	GO:0035497	cAMP response element binding	2/347	12/18369	0.0207234	0.1621462	0.1361904	JUN/NR4A3	2
MF	GO:0070679	inositol 1,4,5 trisphosphate binding	2/347	12/18369	0.0207234	0.1621462	0.1361904	TRPC6/TRPC1	2
MF	GO:0016655	oxidoreductase activity, acting on NAD(P)H, quinone or similar	4/347	57/18369	0.0225606	0.1718762	0.1443629	AKR1C2/NQO2/NQO1/AKR1C3	4

		compound as acceptor							
MF	GO:0016684	oxidoreductase activity, acting on peroxide as acceptor	4/347	57/18369	0.02256606	0.17187662	0.14436629	HBB/PTGS2/DUOX1/GSTT1	4
MF	GO:0045028	G protein-coupled purinergic nucleotide receptor activity	2/347	13/18369	0.02418893	0.18027297	0.15142212	P2RY1/P2RY14	2

MF	GO:0016651	oxidoreductase activity, acting on NAD(P)H	5/347	87/18369	0.0245265	0.1802797	0.1514212	AKR1C2/DUOX1/NQO2/NQO1/AKR1C3	5
MF	GO:0003823	antigen binding	6/347	118/18369	0.0245977	0.1802797	0.1514212	CD1C/HLA-DPA1/ITGA4/CD1E/HLA-DQA1/HLA-DQB1	6
MF	GO:0030296	protein tyrosine kinase activator activity	3/347	34/18369	0.02595574	0.1817523	0.152658	CCL5/STAP1/CD79A	3
MF	GO:0071889	14-3-3 protein binding	3/347	34/18369	0.02595574	0.1817523	0.152658	NFATC2/ZFP36/DDIT4	3
MF	GO:00	magnesium	9/347	223/18369	0.026	0.181	0.152	RPS6KA2/ENO1/IDH1/GEM/EPHX2/WEE1/HPGDS/NLRC4/MTHFD2	9

	00287	ion binding			2688	7523	658		
MF	GO:0016627	oxidoreductase activity, acting on the CH-CH group of donors	4/347	60/18369	0.0266633	0.1817523	0.152658	AKR1C2/DHCR7/FASN/AKR1C3	4
MF	GO:0005313	L-glutamate transmembrane transporter activity	2/347	14/18369	0.0278736	0.1817523	0.152658	SLC7A11/SLC1A3	2
MF	GO:0031404	chloride ion binding	2/347	14/18369	0.0278736	0.1817523	0.152658	NQO2/ACE	2

MF	GO:0042043	neurexin family protein binding	2/347	14/18369	0.0278736	0.1817523	0.152658	SYTL2/CPE	2
MF	GO:0044594	17-beta-hydroxysteroid dehydrogenase (NAD+) activity	2/347	14/18369	0.0278736	0.1817523	0.152658	AKR1C2/AKR1C3	2
MF	GO:0070696	transmembrane receptor protein serine/threonine kinase binding	2/347	14/18369	0.0278736	0.1817523	0.152658	BMP5/INHBA	2

MF	GO:0016765	transferase activity, transferring alkyl or aryl (other than methyl) groups	4/347	61/18369	0.0281236	0.1817523	0.152658	GSTM5/CBS/HPGDS/GSTT1	4
MF	GO:0008236	serine-type peptidase activity	8/347	190/18369	0.0282516	0.1817523	0.152658	CORIN/PRSS12/BMP1/ACE/DPP4/GZMK/PCSK2/C1R	8
MF	GO:0008509	monoatomic anion transmembrane transporter activity	6/347	123/18369	0.0293725	0.1868866	0.1569704	SLC18A2/FXYD1/SLC39A14/ANO1/CFTR/SLC6A4	6

MF	GO:0004896	cytokine receptor activity	5/347	92/18369	0.03028 16	0.19057 65	0.16006 97	IL17RB/IL2RG/IL2RB/IL1R2/CX3CR1	5
MF	GO:0017171	serine hydrolase activity	8/347	194/18369	0.03142 26	0.19360 94	0.16261 71	CORIN/PRSS12/BMP1/ACE/DPP4/GZMK/PCSK2/C1R	8
MF	GO:0015378	sodium:chloride symporter activity	2/347	15/18369	0.03176 67	0.19360 94	0.16261 71	SLC18A2/SLC6A4	2
MF	GO:0140313	molecular sequestering activity	2/347	15/18369	0.03176 67	0.19360 94	0.16261 71	CASQ2/C1R	2
MF	GO:0016829	lyase activity	8/347	198/18369	0.03482 91	0.21006 31	0.17643 69	SHMT2/RNASE2/ENO1/CBS/HMGCLL1/HDC/FASN/CTH	8

MF	GO:0015373	monoatomic anion:sodium symporter activity	2/347	16/18369	0.0358594	0.2118632	0.1779489	SLC18A2/SLC6A4	2
MF	GO:0016641	oxidoreductase activity, acting on the CH-NH2 group of donors, oxygen as acceptor	2/347	16/18369	0.0358594	0.2118632	0.1779489	VCAM1/MAOA	2
MF	GO:0031406	carboxylic acid binding	8/347	200/18369	0.0366228	0.2141877	0.1799013	SHMT2/AKR1C2/PTGDS/SIGLEC10/AKR1C3/S100A8/SLC1A3/SELE	8

MF	GO:0042605	peptide antigen binding	3/347	39/18369	0.037019	0.21434	0.1800293	HLA-DPA1/HLA-DQA1/HLA-DQB1	3
MF	GO:0042277	peptide binding	11/347	317/18369	0.0391836	0.2213596	0.1859252	CD1C/HLA-DPA1/TGFB2/CD1E/FCGR2B/INHBA/ANPEP/MARCO/FPR2/HLA-DQA1/HLA-DQB1	11
MF	GO:0019838	growth factor binding	6/347	132/18369	0.0394215	0.2213596	0.1859252	LTBP4/LTBP2/IL2RG/IL2RB/IL1R2/NTRK2	6
MF	GO:0004860	protein kinase inhibitor activity	3/347	40/18369	0.0394778	0.2213596	0.1859252	TRIB3/TRIB1/SOCS3	3
MF	GO:0005068	transmembrane receptor protein tyrosine kinase	2/347	17/18369	0.0401429	0.2213596	0.1859252	STAP1/TRAT1	2

		adaptor activity							
MF	GO:0005372	water transmembrane transporter activity	2/347	17/18369	0.040141429	0.221353596	0.185929252	SLC14A1/AQP9	2
MF	GO:0030020	extracellular matrix structural constituent conferring tensile strength	3/347	41/18369	0.042010175	0.229510606	0.192927714	COL6A6/COL8A1/COL14A1	3
MF	GO:0004252	serine-type endopeptidase activity	7/347	170/18369	0.042909057	0.232171717	0.195000065	CORIN/PRSS12/BMP1/DPP4/GZMK/PCSK2/C1R	7

MF	GO:0004303	estradiol 17-beta-dehydrogenase activity	2/347	18/18369	0.04460 86	0.23283 77	0.19556 59	AKR1C2/AKR1C3	2
MF	GO:0015172	acidic amino acid transmembrane transporter activity	2/347	18/18369	0.04460 86	0.23283 77	0.19556 59	SLC7A11/SLC1A3	2
MF	GO:0016646	oxidoreductase activity, acting on the CH-NH group of donors,	2/347	18/18369	0.04460 86	0.23283 77	0.19556 59	PYCR1/MTHFD2	2

MF	GO:0008307	NAD or NADP as acceptor or structural constituent of muscle	3/347	42/18369	0.04463 73	0.23283 77	0.19556 59	CAPN3/KRT19/MYH11	3
MF	GO:0020037	heme binding	6/347	137/18369	0.04584 32	0.23699 32	0.19905 62	CBS/HBB/PTGS2/DUOX1/HMOX1/IDO1	6
MF	GO:0002020	protease binding	6/347	138/18369	0.04720 11	0.23832 86	0.20017 78	TIMP4/CD177/SERPINF2/NLRC4/DPP4/NTRK2	6
MF	GO:0004620	phospholipase activity	5/347	104/18369	0.04733 18	0.23832 86	0.20017 78	PLCB4/CCL5/HMOX1/LIPG/PLA2G2A	5
MF	GO:0004715	non-membrane spanni	3/347	43/18369	0.04733 64	0.23832 86	0.20017 78	PKDCC/WEE1/FGR	3

		ng protein tyrosine kinase activity							
MF	GO:0004745	NAD-retinol dehydrogenase activity	2/347	19/18369	0.04924481	0.2437715	0.2047022	SDR16C5/AKR1C3	2
MF	GO:0033612	receptor serine/threonine kinase binding	2/347	19/18369	0.04924481	0.2437715	0.2047022	BMP5/INHBA	2
MF	GO:0015108	chloride transmembrane transpo	5/347	107/18369	0.0523274	0.2567592	0.2156581	SLC18A2/FXYD1/ANO1/CFTR/SLC6A4	5

		rter activity							
MF	GO:0001614	purinergic nucleotide receptor activity	2/347	20/18369	0.0540533	0.2586517	0.2172476	P2RY1/P2RY14	2
MF	GO:0016502	nucleotide receptor activity	2/347	20/18369	0.0540533	0.2586517	0.2172476	P2RY1/P2RY14	2
MF	GO:0016638	oxidoreductase activity, acting on the CH-NH2 group of donors	2/347	20/18369	0.0540533	0.2586517	0.2172476	VCAM1/MAOA	2

MF	GO:0001784	phosphotyrosine residue binding	3/347	47/18369	0.0589092	0.2736633	0.2296043	STAP1/FGR/SOCS3	3
MF	GO:0001846	opsonin binding	2/347	21/18369	0.0590162	0.2736633	0.2296043	VSIG4/PTX3	2
MF	GO:0015377	chloride:monoatomic cation symporter activity	2/347	21/18369	0.0590162	0.2736633	0.2296043	SLC18A2/SLC6A4	2
MF	GO:0043027	cysteine-type endopeptidase inhibitor activity involved in apoptot	2/347	21/18369	0.0590162	0.2736633	0.2296043	NAIP/CD27	2

		ic process							
MF	GO:0046906	tetrapyrrole binding	6/347	147/18369	0.06053999	0.27819533	0.23366628	CBS/HBB/PTGS2/DUOX1/HMOX1/IDO1	6
MF	GO:0016004	phospholipase activator activity	2/347	22/18369	0.06412294	0.2892188	0.2429921	CCL5/CCL8	2
MF	GO:0033691	sialic acid binding	2/347	22/18369	0.06412294	0.2892188	0.2429921	SIGLEC10/SELE	2
MF	GO:0033293	monocarboxylic acid binding	4/347	80/18369	0.06484413	0.2892188	0.2429921	AKR1C2/PTGDS/AKR1C3/S100A8	4
MF	GO:0061135	endopeptidase regulator activity	7/347	187/18369	0.06498	0.2892188	0.2429921	NAIP/TIMP4/SERPINF2/SERPINA3/NLRC4/CD27/PI3	7

MF	GO:0019842	vitamin binding	6/347	150/18369	0.0654362	0.2892218	0.2429221	PSAT1/SHMT2/CBS/HDC/FASN/CTH	6
MF	GO:0008106	alcohol dehydrogenase (NADP+) activity	2/347	23/18369	0.0693853	0.304349	0.2556299	AKR1C2/AKR1C3	2
MF	GO:0016830	carbon-carbon lyase activity	3/347	51/18369	0.0716762	0.3103704	0.2606875	SHMT2/HMGCLL1/HDC	3
MF	GO:0035591	signaling adaptor activity	4/347	83/18369	0.0721817	0.3103704	0.2606875	STAP1/TRAT1/CD3G/CD3E	4
MF	GO:0004435	phosphatidylinositol phospholipase C activity	2/347	24/18369	0.0747767	0.3103704	0.2606875	PLCB4/CCL5	2

MF	GO:0015106	bicarbonate transmembrane transporter activity	2/347	24/18369	0.07477767	0.31003704	0.26068875	SLC39A14/CFTR	2
MF	GO:0055106	ubiquitin-protein transferase regulator activity	2/347	24/18369	0.07477767	0.31003704	0.26068875	TRIB3/TRIB1	2
MF	GO:0070412	R-SMAD binding	2/347	24/18369	0.07477767	0.31003704	0.26068875	JUN/FOS	2
MF	GO:0051287	NAD binding	3/347	52/18369	0.07504464	0.31003704	0.26068875	IDH1/PHGDH/AOX1	3

MF	GO:1901618	organic hydroxy compound transmembrane transporter activity	3/347	52/18369	0.07504664	0.31037704	0.26068875	SLC18A2/AQP9/SLC6A4	3
MF	GO:0001540	amyloid-beta binding	4/347	85/18369	0.07730306	0.3174246	0.26661136	TGFB2/FCGR2B/MARCO/FPR2	4
MF	GO:0004857	enzyme inhibitor activity	11/347	359/18369	0.07974749	0.32063633	0.26930075	TRIB3/NAIP/TIMP4/PPP1R10/SERPINF2/SERPINA3/CD27/RHOH/TRIB1/PI3/SOCS3	11
MF	GO:0008081	phosphoric diester hydrolase activity	4/347	86/18369	0.07992566	0.32063633	0.26930075	PLCB4/PDE7B/CCL5/HMOX1	4

MF	GO:0008483	transaminase activity	2/347	25/18369	0.08029 68	0.32063 33	0.26930 75	PSAT1/BCAT1	2
MF	GO:0060229	lipase activator activity	2/347	25/18369	0.08029 68	0.32063 33	0.26930 75	CCL5/CCL8	2
MF	GO:0004222	metalloendopeptidase activity	5/347	122/18369	0.08172 79	0.32411 28	0.27223	ADAMTS15/BMP1/ACE/ADAM28/ADAMTS9	5
MF	GO:0017124	SH3 domain binding	5/347	123/18369	0.08394 64	0.32952 66	0.27677 71	SGIP1/SKAP1/DPYSL3/FMN1/CD3E	5
MF	GO:0001848	complement binding	2/347	26/18369	0.08593 87	0.32952 66	0.27677 71	VSIG4/PTX3	2
MF	GO:0004190	aspartic-type endopeptidase activity	2/347	26/18369	0.08593 87	0.32952 66	0.27677 71	PGC/CTSE	2

MF	GO:0016769	transferase activity, transferring nitrogenous groups	2/347	26/18369	0.08593 87	0.32952 66	0.27677 71	PSAT1/BCAT1	2
MF	GO:0048027	mRNA 5'-UTR binding	2/347	26/18369	0.08593 87	0.32952 66	0.27677 71	SHMT2/MYH10	2
MF	GO:0016298	lipase activity	5/347	126/18369	0.09079 06	0.34252 87	0.28769 8	PLCB4/CCL5/HMOX1/LIPG/PLA2G2A	5
MF	GO:0004629	phospholipase C activity	2/347	27/18369	0.09169 59	0.34252 87	0.28769 8	PLCB4/CCL5	2
MF	GO:0038187	pattern recognition receptor activity	2/347	27/18369	0.09169 59	0.34252 87	0.28769 8	MARCO/CLEC4E	2

MF	GO:0070001	aspartic-type peptidase activity	2/347	27/18369	0.0916959	0.3425287	0.287698	PGC/CTSE	2
MF	GO:0051219	phosphoprotein binding	4/347	92/18369	0.0965895	0.3565154	0.2994457	STAP1/FGR/SFN/SOCS3	4
MF	GO:0045309	phosphorylated amino acid binding	3/347	58/18369	0.0966717	0.3565154	0.2994457	STAP1/FGR/SOCS3	3
MF	GO:0016645	oxidoreductase activity, acting on the CH-NH group of donors	2/347	28/18369	0.0975622	0.3575221	0.3002913	PYCR1/MTHFD2	2

MF	GO:0005217	intracellular ligand-gated monoatomic ion channel activity	2/347	29/18369	0.10353 12	0.37465 36	0.31468 04	RYR2/CFTR	2
MF	GO:0051861	glycolipid binding	2/347	29/18369	0.10353 12	0.37465 36	0.31468 04	CD1C/CLEC4E	2
MF	GO:0019207	kinase regulator activity	7/347	211/18369	0.10600 62	0.38122 74	0.32020 19	TRIB3/CCL5/STAP1/CD79A/RHOH/TRIB1/SOCS3	7
MF	GO:0016417	S-acyltransferase activity	2/347	30/18369	0.10959 72	0.39170 84	0.32900 51	FASN/ZDHHC9	2
MF	GO:00	endopeptidase inhibito	6/347	173/18369	0.110	0.392	0.329	NAIP/TIMP4/SERPINF2/SERPINA3/CD27/PI3	6

	04866	r activity			3742	0654	305		
MF	GO:0016836	hydro-lyase activity	3/347	62/18369	0.11233	0.3941762	0.3310779	ENO1/CBS/FASN	3
MF	GO:0098631	cell adhesion mediator activity	3/347	62/18369	0.11233	0.3941762	0.3310779	GLDN/ITGA10/CNTN1	3
MF	GO:0022884	macromolecule transmembrane transporter activity	2/347	31/18369	0.1157542	0.4013275	0.3370845	SIDT1/AZGP1	2
MF	GO:0033764	steroid dehydrogenase activity,	2/347	31/18369	0.1157542	0.4013275	0.3370845	AKR1C2/AKR1C3	2

		acting on the CH-OH group of donors, NAD or NADP as acceptor							
MF	GO:0042626	ATPase-coupled transmembrane transporter activity	4/347	99/18369	0.11789871	0.40506653	0.34022239	ABCA9/ABCA6/ABCA8/CFTR	4
MF	GO:0004713	protein tyrosine kinase activity	5/347	137/18369	0.11823315	0.40506653	0.34022239	PKDCC/WEE1/DDR2/FGR/NTRK2	5

MF	GO:0016922	nuclear receptor binding	5/347	138/18369	0.12090 18	0.41177 74	0.34586 15	JUND/TACC2/NR4A3/NR4A1/NR4A2	5
MF	GO:0061629	RNA polymerase II-specific DNA-binding transcription factor binding	10/347	348/18369	0.12493 73	0.42303 33	0.35531 56	SDR16C5/JUN/EGR2/JUND/TACC2/NR4A3/TRIB1/FOS/NR4A1/NR4A2	10
MF	GO:0030414	peptidase inhibitor activity	6/347	180/18369	0.12655 05	0.42600 43	0.35781 1	NAIP/TIMP4/SERPINF2/SERPINA3/CD27/PI3	6
MF	GO:0015103	inorganic anion transmembrane transpo	5/347	141/18369	0.12908 03	0.43200 87	0.36285 43	SLC18A2/FXYD1/ANO1/CFTR/SLC6A4	5

		rter activity							
MF	GO:0004497	monooxygenase activity	4/347	103/18369	0.130365413	0.435618686	0.365588864	AKR1C2/HMOX1/AKR1C3/CH25H	4
MF	GO:0008528	G protein-coupled peptide receptor activity	5/347	142/18369	0.131868613	0.436272727	0.366664357	APLNR/CYSLTR1/FPR1/FPR2/CX3CR1	5
MF	GO:0019887	protein kinase regulator activity	6/347	184/18369	0.136282837	0.448343425	0.376765735	TRIB3/CCL5/STAP1/CD79A/TRIB1/SOCS3	6
MF	GO:0016229	steroid dehydrogenase activity	2/347	35/18369	0.141411847	0.459242468	0.385857322	AKR1C2/AKR1C3	2

MF	GO:0016814	hydrolase activity, acting on carbon-nitrogen (but not peptide) bonds, in cyclic amidines	2/347	35/18369	0.14118 47	0.45924 68	0.38573 22	AMPD1/MTHFD2	2
MF	GO:0061134	peptidase regulator activity	7/347	230/18369	0.14639 14	0.46967 3	0.39448 95	NAIP/TIMP4/SERPINF2/SERPINA3/NLRC4/CD27/PI3	7
MF	GO:0000146	microfilament motor activity	2/347	36/18369	0.14771 71	0.46967 3	0.39448 95	MYH10/MYH11	2

MF	GO:0030280	structural constituent of skin epidermis	2/347	36/18369	0.1477171	0.4696673	0.3944895	KRT7/PI3	2
MF	GO:0043531	ADP binding	2/347	36/18369	0.1477171	0.4696673	0.3944895	P2RY1/MYH10	2
MF	GO:0001653	peptide receptor activity	5/347	148/18369	0.1491014	0.4696673	0.3944895	APLNR/CYSLTR1/FPR1/FPR2/CX3CR1	5
MF	GO:0043621	protein self-association	3/347	72/18369	0.1551753	0.4696673	0.3944895	RYR2/CCL5/CD2	3
MF	GO:0015294	solute:monoatomic cation symporter activity	4/347	112/18369	0.1621955	0.4696673	0.3944895	SLC18A2/SLC39A14/SLC1A3/SLC6A4	4

MF	GO:0005254	chloride channel activity	3/347	74/18369	0.1642879	0.469673	0.3944895	FXYD1/ANO1/CFTR	3
MF	GO:0019825	oxygen binding	2/347	39/18369	0.1676573	0.469673	0.3944895	CBS/HBB	2
MF	GO:0071949	FAD binding	2/347	39/18369	0.1676573	0.469673	0.3944895	NQO2/AOX1	2
MF	GO:0030971	receptor tyrosine kinase binding	3/347	76/18369	0.1735552	0.469673	0.3944895	STAP1/TRAT1/CD2	3
MF	GO:0003796	lysozyme activity	1/347	10/18369	0.1736687	0.469673	0.3944895	LYZ	1
MF	GO:00	FK506 binding	1/347	10/18369	0.173	0.469	0.394	FKBP5	1

	05528				0.17366687	0.469673	0.3944895		
MF	GO:0008190	eukaryotic initiation factor 4E binding	1/347	10/18369	0.17366687	0.469673	0.3944895	EIF4EBP1	1
MF	GO:0008239	dipeptidyl-peptidase activity	1/347	10/18369	0.17366687	0.469673	0.3944895	DPP4	1
MF	GO:0015643	toxic substance binding	1/347	10/18369	0.17366687	0.469673	0.3944895	EPHX2	1
MF	GO:0016175	superoxide-generating NAD(P)H oxidase activity	1/347	10/18369	0.17366687	0.469673	0.3944895	DUOX1	1

MF	GO:0016176	superoxide-generating NADPH oxidase activator activity	1/347	10/18369	0.17366687	0.4696673	0.39448895	SH3PXD2B	1
MF	GO:0016303	1-phosphatidylinositol-3-kinase activity	1/347	10/18369	0.17366687	0.4696673	0.39448895	PIK3CG	1
MF	GO:0016595	glutamate binding	1/347	10/18369	0.17366687	0.4696673	0.39448895	SLC1A3	1
MF	GO:0016653	oxidoreductase activity, acting	1/347	10/18369	0.17366687	0.4696673	0.39448895	NQO1	1

		on NAD(P)H, heme protein as acceptor							
MF	GO:0016742	hydroxymethyl-, formyl- and related transferase activity	1/347	10/18369	0.17366687	0.469673	0.3944895	SHMT2	1
MF	GO:0016832	aldehyde-lyase activity	1/347	10/18369	0.17366687	0.469673	0.3944895	SHMT2	1
MF	GO:0030023	extracellular matrix constituent	1/347	10/18369	0.17366687	0.469673	0.3944895	MMRN1	1

		conferring elasticity							
MF	GO:0031720	haptoglobin binding	1/347	10/18369	0.17366687	0.469673	0.39448895	HBB	1
MF	GO:0034987	immunoglobulin receptor binding	1/347	10/18369	0.17366687	0.469673	0.39448895	FGR	1
MF	GO:0035374	chondroitin sulfate binding	1/347	10/18369	0.17366687	0.469673	0.39448895	DPYSL3	1
MF	GO:0043121	neurotrophin binding	1/347	10/18369	0.17366687	0.469673	0.39448895	NTRK2	1
MF	GO:0043225	ATPase-coupled	1/347	10/18369	0.17366687	0.469673	0.39448895	CFTR	1

		inorganic anion transmembrane transporter activity							
MF	GO:0051378	serotonin binding	1/347	10/18369	0.17366687	0.4696673	0.3944895	SLC6A4	1
MF	GO:0052740	1-acyl-2-lysophosphatidylserine acylhydrolase activity	1/347	10/18369	0.17366687	0.4696673	0.3944895	LIPG	1
MF	GO:0052813	phosphatidylinositol bisphosphate	1/347	10/18369	0.17366687	0.4696673	0.3944895	PIK3CG	1

		kinase activity							
MF	GO:0070008	serine-type exopeptidase activity	1/347	10/18369	0.17366687	0.4696673	0.39448895	ACE	1
MF	GO:0070700	BMP receptor binding	1/347	10/18369	0.17366687	0.4696673	0.39448895	BMP5	1
MF	GO:0004177	aminopeptidase activity	2/347	40/18369	0.17440436	0.4696673	0.39448895	DPP4/ANPEP	2
MF	GO:0016709	oxidoreductase activity, acting on paired donors, with incorporation	2/347	40/18369	0.17440436	0.4696673	0.39448895	AKR1C2/AKR1C3	2

		or reduction of molecular oxygen, NAD(P)H as one donor, and incorporation of one atom of oxygen							
MF	GO:0046915	transition metal ion transmembrane transpo	2/347	40/18369	0.17440436	0.469673	0.39448495	SLC39A14/SLC11A1	2

		rter activity							
MF	GO:0016835	carbon-oxygen lyase activity	3/347	77/18369	0.178243 3	0.474082 6	0.398193 2	ENO1/CBS/FASN	3
MF	GO:0019199	transmembrane receptor protein kinase activity	3/347	77/18369	0.178243 3	0.474082 6	0.398193 2	LTBP4/DDR2/NTRK2	3
MF	GO:0005516	calmodulin binding	6/347	200/18369	0.178497 4	0.474082 6	0.398193 2	RYR2/GEM/ACE/MYH10/CTH/MYH11	6
MF	GO:0015370	solute:sodium symporter activity	3/347	79/18369	0.187721 8	0.474446 6	0.398499	SLC18A2/SLC1A3/SLC6A4	3
MF	GO:00	active transmembran	11/347	428/18369	0.188	0.474	0.398	SLC18A2/ABCA9/ABCA6/ABCA8/SLCO4A1/SLC39A14/SLC7A11/SLC1A3/CFTR/SLC11A1/SLC6A4	11

	22804	e transporter activity			4645	4466	499			
MF	GO:0001851	complement component C3b binding	1/347	11/18369	0.189287	0.474466	0.398499	VSIG4		1
MF	GO:0005024	transforming growth factor beta receptor activity	1/347	11/18369	0.189287	0.474466	0.398499	LTBP4		1
MF	GO:0005247	voltage-gated chloride channel activity	1/347	11/18369	0.189287	0.474466	0.398499	ANO1		1

MF	GO:0008046	axon guidance receptor activity	1/347	11/18369	0.189287	0.4744466	0.398499	ROBO2	1
MF	GO:0016679	oxidoreductase activity, acting on diphenols and related substances as donors	1/347	11/18369	0.189287	0.4744466	0.398499	NQO2	1
MF	GO:0019864	IgG binding	1/347	11/18369	0.189287	0.4744466	0.398499	FCGR2B	1
MF	GO:0030283	testosterone dehydrogenas	1/347	11/18369	0.189287	0.4744466	0.398499	AKR1C3	1

		e [NAD(P)] activity							
MF	GO:0035252	UDP-xylosyltransferase activity	1/347	11/18369	0.189287	0.474466	0.398499	GXYLT2	1
MF	GO:0042285	xylosyltransferase activity	1/347	11/18369	0.189287	0.474466	0.398499	GXYLT2	1
MF	GO:0043176	amine binding	1/347	11/18369	0.189287	0.474466	0.398499	SLC6A4	1
MF	GO:0052739	phosphatidylserine 1-acylhydrolase activity	1/347	11/18369	0.189287	0.474466	0.398499	LIPG	1
MF	GO:00	cysteine-type endope	2/347	43/18369	0.194	0.486	0.408	NAIP/CD27	2

	43028	ptidase regulator activity involved in apoptotic process			8831	3676	5117		
MF	GO:0000099	sulfur amino acid transmembrane transporter activity	1/347	12/18369	0.2046611	0.4935514	0.4145141	SLC7A11	1
MF	GO:0005095	GTPase inhibitor activity	1/347	12/18369	0.2046611	0.4935514	0.4145141	RHOH	1

MF	GO:0015132	prostaglandin transmembrane transporter activity	1/347	12/18369	0.2046611	0.4935514	0.4145141	SLCO4A1	1
MF	GO:0015280	ligand-gated sodium channel activity	1/347	12/18369	0.2046611	0.4935514	0.4145141	SCNN1G	1
MF	GO:0035004	phosphatidylinositol 3-kinase activity	1/347	12/18369	0.2046611	0.4935514	0.4145141	PIK3CG	1
MF	GO:0042301	phosphate ion binding	1/347	12/18369	0.2046611	0.4935514	0.4145141	MTHFD2	1

MF	GO:0086080	protein binding involved in heterotypic cell-cell adhesion	1/347	12/18369	0.2046611	0.4935514	0.4145141	GLDN	1
MF	GO:0097493	structural molecule activity conferring elasticity	1/347	12/18369	0.2046611	0.4935514	0.4145141	MMRN1	1
MF	GO:0003955	NAD(P)H dehydrogenase (quinone) activity	2/347	45/18369	0.2086981	0.4935514	0.4145141	NQO2/NQO1	2

MF	GO:0015291	secondary active transmembrane transporter activity	7/347	255/18369	0.208803 4	0.493514	0.414514 1	SLC18A2/SLCO4A1/SLC39A14/SLC7A11/SLC1A3/SLC11A1/SLC6A4	7
MF	GO:0015175	neutral L-amino acid transmembrane transporter activity	2/347	46/18369	0.215643 4	0.493514	0.414514 1	SLC7A11/SLC1A3	2
MF	GO:0001227	DNA-binding transcription repressor activity,	8/347	303/18369	0.215933 7	0.493514	0.414514 1	KLF12/ENO1/GTF2IRD1/NFATC2/JUN/TFEC/NR1D2/ATF3	8

MF	GO:0072341	RNA polymerase II-specific modified amino acid binding	3/347	85/18369	0.2168764	0.4935514	0.4145141	CBS/SYTL2/FASN	3
MF	GO:0004312	fatty acid synthase activity	1/347	13/18369	0.2196461	0.4935514	0.4145141	FASN	1
MF	GO:0008417	fucosyltransferase activity	1/347	13/18369	0.2196461	0.4935514	0.4145141	FUT3	1
MF	GO:0008603	cAMP-dependent protein kinase regulator activity	1/347	13/18369	0.2196461	0.4935514	0.4145141	CXCL10	1

MF	GO:0015166	polyol transmembrane transporter activity	1/347	13/18369	0.2196461	0.4935514	0.41451 41	AQP9	1
MF	GO:0031432	titin binding	1/347	13/18369	0.2196461	0.4935514	0.4145141	CAPN3	1
MF	GO:0034236	protein kinase A catalytic subunit binding	1/347	13/18369	0.2196461	0.4935514	0.4145141	RYR2	1
MF	GO:0035325	Toll-like receptor binding	1/347	13/18369	0.2196461	0.4935514	0.4145141	S100A8	1
MF	GO:0050998	nitric-oxide synthas	1/347	13/18369	0.2196461	0.4935514	0.4145141	SLC6A4	1

		e binding							
MF	GO:0052650	NADP-retinol dehydrogenase activity	1/347	13/18369	0.2196461	0.4935514	0.4145141	AKR1C3	1
MF	GO:0061133	endopeptidase activator activity	1/347	13/18369	0.2196461	0.4935514	0.4145141	NLRC4	1
MF	GO:0061665	SUMO ligase activity	1/347	13/18369	0.2196461	0.4935514	0.4145141	EGR2	1
MF	GO:0071714	icosanoid transmembrane transporter activity	1/347	13/18369	0.2196461	0.4935514	0.4145141	SLCO4A1	1

MF	GO:0005253	monoatomic anion channel activity	3/347	87/18369	0.22679788	0.4935145	0.41451541	FXYD1/ANO1/CFTR	3
MF	GO:0001217	DNA-binding transcription repressor activity	8/347	308/18369	0.22852554	0.4935145	0.41451541	KLF12/ENO1/GTF2IRD1/NFATC2/JUN/TFEC/NR1D2/ATF3	8
MF	GO:0016860	intramolecular oxidoreductase activity	2/347	48/18369	0.22959527	0.4935145	0.41451541	PTGDS/HPGDS	2
MF	GO:0016903	oxidoreductase activity, acting on the aldehy	2/347	48/18369	0.22959527	0.4935145	0.41451541	AKR1C3/AOX1	2

		de or oxo group of donors							
MF	GO:0043178	alcohol binding	3/347	88/18369	0.2317926	0.493514	0.4145141	TRPC6/SIDT1/TRPC1	3
MF	GO:0004065	arylsulfatase activity	1/347	14/18369	0.2343978	0.493514	0.4145141	SULF1	1
MF	GO:0004953	icosanoid receptor activity	1/347	14/18369	0.2343978	0.493514	0.4145141	CYSLTR1	1
MF	GO:0005344	oxygen carrier activity	1/347	14/18369	0.2343978	0.493514	0.4145141	HBB	1
MF	GO:0008559	ABC-type xenobiotic	1/347	14/18369	0.2343978	0.493514	0.4145141	ABCA8	1

		transporter activity							
MF	GO:0015250	water channel activity	1/347	14/18369	0.2343978	0.4935514	0.4145141	AQP9	1
MF	GO:0015926	glucosidase activity	1/347	14/18369	0.2343978	0.4935514	0.4145141	MGAM	1
MF	GO:0016307	phosphatidylinositol phosphate kinase activity	1/347	14/18369	0.2343978	0.4935514	0.4145141	PIK3CG	1
MF	GO:0019992	diacylglycerol binding	1/347	14/18369	0.2343978	0.4935514	0.4145141	RASGRP1	1
MF	GO:0042577	lipid phosphatase activity	1/347	14/18369	0.2343978	0.4935514	0.4145141	EPHX2	1

MF	GO:0061578	K63-linked deubiquitinase activity	1/347	14/18369	0.23439578	0.4935514	0.41451541	STAMBPL1	1
MF	GO:0061783	peptidoglycan muralytic activity	1/347	14/18369	0.23439578	0.4935514	0.41451541	LYZ	1
MF	GO:0071837	HMG box domain binding	1/347	14/18369	0.23439578	0.4935514	0.41451541	EGR2	1
MF	GO:0089720	caspase binding	1/347	14/18369	0.23439578	0.4935514	0.41451541	NLRC4	1
MF	GO:0005504	fatty acid binding	2/347	49/18369	0.23659504	0.49632555	0.41687756	PTGDS/S100A8	2
MF	GO:0016705	oxidoreductase activity,	5/347	177/18369	0.24362651	0.5056021	0.42466671	AKR1C2/PTGS2/HMOX1/AKR1C3/CH25H	5

		acting on paired donors, with incorporation or reduction of molecular oxygen							
MF	GO:0051213	dioxygenase activity	3/347	91/18369	0.2468887	0.5056021	0.4246671	PTGS2/ALOX15B/IDO1	3
MF	GO:0005041	low-density lipoprotein particle receptor activity	1/347	15/18369	0.2488715	0.5056021	0.4246671	STAB1	1

MF	GO:0016812	hydrolase activity, acting on carbon-nitrogen (but not peptide) bonds, in cyclic amides	1/347	15/18369	0.2488715	0.5056021	0.4246671	DPYSL3	1
MF	GO:0016846	carbon-sulfur lyase activity	1/347	15/18369	0.2488715	0.5056021	0.4246671	CTH	1
MF	GO:0031005	filamin binding	1/347	15/18369	0.2488715	0.5056021	0.4246671	DPYSL3	1
MF	GO:00	nuclear retinoid X	1/347	15/18369	0.248	0.505	0.424	NR4A2	1

	46965	receptor binding			8715	6021	6671		
MF	GO:0051371	muscle alpha-actinin binding	1/347	15/18369	0.2488715	0.5056021	0.4246671	PDLIM3	1
MF	GO:0070492	oligosaccharide binding	1/347	15/18369	0.2488715	0.5056021	0.4246671	SELE	1
MF	GO:0098632	cell-cell adhesion mediator activity	2/347	51/18369	0.2506166	0.5073672	0.4261497	GLDN/CNTN1	2
MF	GO:0070851	growth factor receptor binding	4/347	137/18369	0.2601708	0.5128581	0.4307617	MS4A1/IL1B/IL6/CSF3	4
MF	GO:0004553	hydrolase activity, hydroly	3/347	94/18369	0.2621316	0.5128581	0.4307617	LYZ/CHIT1/MGAM	3

		zing O-glycosyl compounds							
MF	GO:0000900	mRNA regulatory element binding translation repressor activity	1/347	16/18369	0.26307 23	0.51285 81	0.43076 17	SHMT2	1
MF	GO:0004198	calcium-dependent cysteine-type endopeptidase activity	1/347	16/18369	0.26307 23	0.51285 81	0.43076 17	CAPN3	1

MF	GO:0008191	metalloendopeptidase inhibitor activity	1/347	16/18369	0.263072	0.5128581	0.43076 17	TIMP4	1
MF	GO:0015347	sodium-independent organic anion transmembrane transporter activity	1/347	16/18369	0.263072	0.5128581	0.4307617	SLCO4A1	1
MF	GO:0016722	oxidoreductase activity, acting on metal ions	1/347	16/18369	0.263072	0.5128581	0.4307617	CP	1

MF	GO:0017081	chloride channel regulator activity	1/347	16/18369	0.26307723	0.51285581	0.43076617	CFTR	1
MF	GO:0017110	nucleoside diphosphate phosphatase activity	1/347	16/18369	0.26307723	0.51285581	0.43076617	ALPL	1
MF	GO:0050664	oxidoreductase activity, acting on NAD(P)H, oxygen as acceptor	1/347	16/18369	0.26307723	0.51285581	0.43076617	DUOX1	1

MF	GO:0070402	NADPH binding	1/347	16/18369	0.263072 3	0.512858 1	0.430761 7	KCNAB1	1
MF	GO:0004869	cysteine-type endopeptidase inhibitor activity	2/347	54/18369	0.271690 9	0.515577 3	0.433045 6	NAIP/CD27	2
MF	GO:0000400	four-way junction DNA binding	1/347	17/18369	0.277005 4	0.515577 3	0.433045 6	HMGB3	1
MF	GO:0004115	3',5'-cyclic-AMP phosphodiesterase activity	1/347	17/18369	0.277005 4	0.515577 3	0.433045 6	PDE7B	1
MF	GO:00	transmembrane	1/347	17/18369	0.277	0.515	0.433	LTBP4	1

	04675	receptor protein serine/threonine kinase activity			0054	5773	0456			
MF	GO:0005149	interleukin-1 receptor binding	1/347	17/18369	0.2770054	0.5155773	0.4330456	IL1B		1
MF	GO:0008308	voltage-gated monoatomic anion channel activity	1/347	17/18369	0.2770054	0.5155773	0.4330456	ANO1		1
MF	GO:0008484	sulfuric ester hydrolase activity	1/347	17/18369	0.2770054	0.5155773	0.4330456	SULF1		1

MF	GO:0008569	minus-end-directed microtubule motor activity	1/347	17/18369	0.2770054	0.5155773	0.4330456	DNAH5	1
MF	GO:0015278	calcium-release channel activity	1/347	17/18369	0.2770054	0.5155773	0.4330456	RYR2	1
MF	GO:0016805	dipeptidase activity	1/347	17/18369	0.2770054	0.5155773	0.4330456	ACE	1
MF	GO:0030169	low-density lipoprotein particle binding	1/347	17/18369	0.2770054	0.5155773	0.4330456	STAB1	1

MF	GO:0031420	alkali metal ion binding	1/347	17/18369	0.2770054	0.51555773	0.43304456	CAPN3	1
MF	GO:0047498	calcium-dependent phospholipase A2 activity	1/347	17/18369	0.2770054	0.51555773	0.43304456	PLA2G2A	1
MF	GO:0052742	phosphatidylinositol kinase activity	1/347	17/18369	0.27700054	0.51555773	0.43304456	PIK3CG	1
MF	GO:0016597	amino acid binding	2/347	55/18369	0.27871145	0.51555773	0.43304456	SHMT2/SLC1A3	2
MF	GO:0050840	extracellular matrix binding	2/347	55/18369	0.27871145	0.51555773	0.43304456	ADAMTS15/DCN	2

MF	GO:0015293	symporter activity	4/347	144/18369	0.2895107	0.5259415	0.4417507	SLC18A2/SLC39A14/SLC1A3/SLC6A4	4
MF	GO:0005229	intracellular calcium activated chloride channel activity	1/347	18/18369	0.29067658	0.5259415	0.4417507	ANO1	1
MF	GO:0008301	DNA binding, bending	1/347	18/18369	0.29067658	0.5259415	0.4417507	HMGB3	1
MF	GO:0008970	phospholipase A1 activity	1/347	18/18369	0.29067658	0.5259415	0.4417507	LIPG	1
MF	GO:00	endonuclease activity,	1/347	18/18369	0.290	0.525	0.441	RNASE2	1

	16894	active with either ribo- or deoxyribonucleic acids and producing 3'-phosphomonoesters			6758	9415	7507			
MF	GO:0030228	lipoprotein particle receptor activity	1/347	18/18369	0.2906758	0.5259415	0.4417507	STAB1		1
MF	GO:1904047	S-adenosyl-L-methionine binding	1/347	18/18369	0.2906758	0.5259415	0.4417507	CBS		1

MF	GO:0099106	ion channel regulator activity	4/347	147/18369	0.30222276	0.5384318	0.4522416	FXYD1/KCNAB1/GEM/CFTR	4
MF	GO:0000979	RNA polymerase II core promoter sequence-specific DNA binding	1/347	19/18369	0.3040884	0.5384318	0.4522416	FOS	1
MF	GO:0005326	neurotransmitter transmembrane transporter activity	1/347	19/18369	0.3040884	0.5384318	0.4522416	SLC6A4	1

MF	GO:0036041	long-chain fatty acid binding	1/347	19/18369	0.30408 84	0.53843 18	0.45224 16	S100A8	1
MF	GO:0046935	1-phosphatidylinositol-3-kinase regulator activity	1/347	19/18369	0.30408 84	0.53843 18	0.45224 16	SOCS3	1
MF	GO:0061778	intracellular chloride channel activity	1/347	19/18369	0.30408 84	0.53843 18	0.45224 16	ANO1	1
MF	GO:0099095	ligand-gated monoatomic anion	1/347	19/18369	0.30408 84	0.53843 18	0.45224 16	CFTR	1

		channel activity							
MF	GO:0046873	metal ion transmembrane transporter activity	10/347	435/18369	0.3084863	0.5445536	0.4573835	TRPC6/RYR2/SLC18A2/SCNN1G/KCNAB1/TRPC1/SLC39A14/SLC1A3/SLC11A1/SLC6A4	10
MF	GO:0015297	antiporter activity	3/347	104/18369	0.3136038	0.5504306	0.4623197	SLC18A2/SLC7A11/SLC11A1	3
MF	GO:0004714	transmembrane receptor protein tyrosine kinase activity	2/347	60/18369	0.3137169	0.5504306	0.4623197	DDR2/NTRK2	2

MF	GO:0010314	phosphatidylinositol-5-phosphate binding	1/347	20/18369	0.3172482	0.5532732	0.4647072	SH3PXD2B	1
MF	GO:0017075	syntaxin-1 binding	1/347	20/18369	0.3172482	0.5532732	0.4647072	SLC6A4	1
MF	GO:0005506	iron ion binding	4/347	152/18369	0.3235521	0.5608882	0.4711032	LCN2/CH25H/ALOX15B/AOX1	4
MF	GO:0016247	channel regulator activity	4/347	152/18369	0.3235521	0.5608882	0.4711032	FXYD1/KCNAB1/GEM/CFTR	4
MF	GO:0005507	copper ion binding	2/347	62/18369	0.3276242	0.5645667	0.4741929	CP/S100A12	2

MF	GO:0048306	calcium-dependent protein binding	2/347	62/18369	0.3276242	0.5645667	0.4741929	CD177/CASQ2	2
MF	GO:0008373	sialyltransferase activity	1/347	21/18369	0.3301598	0.5655696	0.4750352	ST6GALNAC3	1
MF	GO:0035613	RNA stem-loop binding	1/347	21/18369	0.3301598	0.5655696	0.4750352	MYH10	1
MF	GO:0016853	isomerase activity	4/347	154/18369	0.3321136	0.5672383	0.4764369	PTGDS/HPGDS/CFTR/FKBP5	4
MF	GO:0015081	sodium ion transmembrane transporter activity	4/347	155/18369	0.3363989	0.5728676	0.481165	SLC18A2/SCNN1G/SLC1A3/SLC6A4	4

MF	GO:0004602	glutathione peroxidase activity	1/347	22/18369	0.3428279	0.5753546	0.4483254	GSTT1	1
MF	GO:0004993	G protein-coupled serotonin receptor activity	1/347	22/18369	0.3428279	0.5753546	0.4483254	HRH2	1
MF	GO:0005283	amino acid:sodium symporter activity	1/347	22/18369	0.3428279	0.5753546	0.4483254	SLC1A3	1
MF	GO:0016881	acid-amino acid ligase activity	1/347	22/18369	0.3428279	0.5753546	0.4483254	GCLM	1

MF	GO:0035014	phosphatidylinositol 3-kinase regulator activity	1/347	22/18369	0.3428279	0.57535546	0.48325 4	SOCS3	1
MF	GO:0015179	L-amino acid transmembrane transporter activity	2/347	65/18369	0.34833 72	0.57941 93	0.48666 8	SLC7A11/SLC1A3	2
MF	GO:0001221	transcription coregulator binding	3/347	111/18369	0.34982 4	0.57941 93	0.48666 8	ENO1/JUND/FOS	3
MF	GO:0097110	scaffold protein binding	2/347	66/18369	0.35519 52	0.57941 93	0.48666 8	P2RY1/CD163	2

MF	GO:0004806	triglyceride lipase activity	1/347	23/18369	0.3552571	0.5794193	0.4866668	LIPG	1
MF	GO:0005160	transforming growth factor beta receptor binding	1/347	23/18369	0.3552571	0.5794193	0.4866668	TGFB2	1
MF	GO:0005385	zinc ion transmembrane transporter activity	1/347	23/18369	0.3552571	0.5794193	0.4866668	SLC39A14	1
MF	GO:0035035	histone acetyltransferase binding	1/347	23/18369	0.3552571	0.5794193	0.4866668	EGR1	1

MF	GO:0043274	phospholipase binding	1/347	23/18369	0.3552571	0.5794193	0.4866668	SELE	1
MF	GO:0050431	transforming growth factor beta binding	1/347	23/18369	0.3552571	0.5794193	0.4866668	LTBP4	1
MF	GO:0050699	WW domain binding	1/347	23/18369	0.3552571	0.5794193	0.4866668	SCNN1G	1
MF	GO:0000030	mannosyltransferase activity	1/347	24/18369	0.3674519	0.586101	0.4922801	TMTC1	1
MF	GO:0003707	nuclear steroid receptor activity	1/347	24/18369	0.3674519	0.586101	0.4922801	NR4A3	1
MF	GO:00	3',5'-cyclic-nucleoti	1/347	24/18369	0.367	0.586	0.492	PDE7B	1

	04114	de phosphodiesterase activity			4519	101	2801		
MF	GO:0005227	calcium activated cation channel activity	1/347	24/18369	0.3674519	0.586101	0.4922801	ANO1	1
MF	GO:0008093	cytoskeletal anchor activity	1/347	24/18369	0.3674519	0.586101	0.4922801	ANK2	1
MF	GO:0008395	steroid hydroxylase activity	1/347	24/18369	0.3674519	0.586101	0.4922801	CH25H	1
MF	GO:0042974	nuclear retinoic acid receptor binding	1/347	24/18369	0.3674519	0.586101	0.4922801	NR4A2	1

MF	GO:0051139	metal cation:proton antiporter activity	1/347	24/18369	0.3674519	0.586101	0.4922801	SLC11A1	1
MF	GO:0019905	syntaxin binding	2/347	68/18369	0.3688326	0.5866871	0.4927723	TXLNB/SLC6A4	2
MF	GO:0003774	cytoskeletal motor activity	3/347	115/18369	0.3704446	0.5876390	0.4935719	MYH10/MYH11/DNAH5	3
MF	GO:0001637	G protein-coupled chemoattractant receptor activity	1/347	25/18369	0.37941 67	0.59213 55	0.49734 86	CX3CR1	1

MF	GO:0004950	chemokine receptor activity	1/347	25/18369	0.3794167	0.5921355	0.4973486	CX3CR1	1
MF	GO:0008320	protein transmembrane transporter activity	1/347	25/18369	0.3794167	0.5921355	0.4973486	AZGP1	1
MF	GO:0030247	polysaccharide binding	1/347	25/18369	0.3794167	0.5921355	0.4973486	PTX3	1
MF	GO:0034237	protein kinase A regulatory subunit binding	1/347	25/18369	0.3794167	0.5921355	0.4973486	RYR2	1
MF	GO:00	2 iron, 2 sulfur	1/347	25/18369	0.379	0.592	0.497	AOX1	1

	51537	cluster binding			4167	1355	3486		
MF	GO:0016757	glycosyltransferase activity	6/347	266/18369	0.3884446	0.5959979	0.5005928	FUT3/GLT8D2/GXYLT2/ST6GALNAC3/TMTC1/HAS2	6
MF	GO:0004112	cyclic-nucleotide phosphodiesterase activity	1/347	26/18369	0.3911558	0.5959979	0.5005928	PDE7B	1
MF	GO:0019706	protein-cysteine S-palmitoyltransferase activity	1/347	26/18369	0.3911558	0.5959979	0.5005928	ZDHHC9	1
MF	GO:0019707	protein-cysteine S-acyltransferas	1/347	26/18369	0.3911558	0.5959979	0.5005928	ZDHHC9	1

		e activity							
MF	GO:0019789	SUMO transferase activity	1/347	26/18369	0.39115 58	0.59599 79	0.50059 28	EGR2	1
MF	GO:0030507	spectrin binding	1/347	26/18369	0.39115 58	0.59599 79	0.50059 28	ANK2	1
MF	GO:0035925	mRNA 3'-UTR AU-rich region binding	1/347	26/18369	0.39115 58	0.59599 79	0.50059 28	ZFP36	1
MF	GO:0070006	metalloaminopeptidase activity	1/347	26/18369	0.39115 58	0.59599 79	0.50059 28	ANPEP	1
MF	GO:0099604	ligand-gated calcium channel activity	1/347	26/18369	0.39115 58	0.59599 79	0.50059 28	RYR2	1

MF	GO:0005319	lipid transporter activity	4/347	169/18369	0.396393937	0.602393936	0.505959646	ABCA9/ABCA6/ABCA8/SLCO4A1	4
MF	GO:0015399	primary active transmembrane transporter activity	4/347	170/18369	0.400656589	0.605055791	0.508086402	ABCA9/ABCA6/ABCA8/CFTR	4
MF	GO:1990782	protein tyrosine kinase binding	3/347	121/18369	0.40113136	0.605055791	0.508086402	STAP1/TRAT1/CD2	3
MF	GO:0051959	dynein light intermediate chain binding	1/347	27/18369	0.402676735	0.605055791	0.508086402	DNAH5	1
MF	GO:00	serotonin recepto	1/347	27/18369	0.40202	0.60505	0.50808	HRH2	1

	99589	r activity			6735	5791	6402		
MF	GO:0009055	electron transfer activity	3/347	122/18369	0.4062131	0.6093196	0.5117819	PHGDH/NQO2/IDO1	3
MF	GO:0005416	amino acid:monoatomic cation symporter activity	1/347	28/18369	0.4139739	0.614592	0.5162104	SLC1A3	1
MF	GO:0051019	mitogen-activated protein kinase binding	1/347	28/18369	0.4139739	0.614592	0.5162104	ACE	1
MF	GO:0051393	alpha-actinin binding	1/347	28/18369	0.4139739	0.614592	0.5162104	PDLIM3	1

MF	GO:0080025	phosphatidylinositol-3,5-bisphosphate binding	1/347	28/18369	0.4139739	0.6145592	0.5162104	SH3PXD2B	1
MF	GO:0015267	channel activity	10/347	478/18369	0.4165459	0.6152553	0.5167675	TRPC6/RYR2/SCNN1G/FXYD1/KCNAB1/TRPC1/SLC14A1/AQP9/ANO1/CFTR	10
MF	GO:0140297	DNA-binding transcription factor binding	10/347	478/18369	0.4165459	0.6152553	0.5167675	SDR16C5/JUN/EGR2/JUND/TACC2/NR4A3/TRIB1/FOS/NR4A1/NR4A2	10
MF	GO:0022803	passive transmembrane transporter activity	10/347	479/18369	0.4190919	0.6174408	0.5186031	TRPC6/RYR2/SCNN1G/FXYD1/KCNAB1/TRPC1/SLC14A1/AQP9/ANO1/CFTR	10

MF	GO:0003924	GTPase activity	7/347	327/18369	0.4226296	0.6210724	0.5216534	GEM/RHOBTB2/RGS5/RHOH/RGS13/GBP5/GBP4	7
MF	GO:0046875	ephrin receptor binding	1/347	29/18369	0.4250612	0.6230643	0.5233265	PIK3CG	1
MF	GO:0005262	calcium channel activity	3/347	126/18369	0.4263879	0.6234308	0.5236343	TRPC6/RYR2/TRPC1	3
MF	GO:0001618	virus receptor activity	2/347	77/18369	0.4286408	0.6235754	0.5237558	DPP4/ANPEP	2
MF	GO:0046332	SMAD binding	2/347	77/18369	0.4286408	0.6235754	0.5237558	JUN/FOS	2
MF	GO:0140272	exogenous protein binding	2/347	78/18369	0.4351059	0.6247743	0.5247627	DPP4/ANPEP	2

MF	GO:0005164	tumor necrosis factor receptor binding	1/347	30/18369	0.4359392	0.6247743	0.5247627	TNFSF10	1
MF	GO:0031210	phosphatidylcholine binding	1/347	30/18369	0.4359392	0.6247743	0.5247627	RASGRP1	1
MF	GO:0042910	xenobiotic transmembrane transporter activity	1/347	30/18369	0.4359392	0.6247743	0.5247627	ABCA8	1
MF	GO:0071813	lipoprotein particle binding	1/347	30/18369	0.4359392	0.6247743	0.5247627	STAB1	1
MF	GO:0071814	protein-lipid comple	1/347	30/18369	0.4359392	0.6247743	0.5247627	STAB1	1

		x binding							
MF	GO:0008514	organic anion transmembrane transporter activity	5/347	230/18369	0.4392401	0.6279507	0.5274306	SLCO4A1/SLC39A14/SLC7A11/SLC1A3/CFTR	5
MF	GO:0044325	transmembrane transporter binding	3/347	129/18369	0.4413614	0.6294293	0.5286725	FXYD1/KCNAB1/ANK2	3
MF	GO:0001968	fibronectin binding	1/347	31/18369	0.4466121	0.6322454	0.5310379	ITGA4	1
MF	GO:0005154	epidermal growth factor recepto	1/347	31/18369	0.4466121	0.6322454	0.5310379	MS4A1	1

		r binding								
MF	GO:0048019	receptor antagonist activity	1/347	31/18369	0.44661 21	0.63224 54	0.53103 79	CCL5		1
MF	GO:0022853	active monoatomic ion transmembrane transporter activity	5/347	233/18369	0.45030 61	0.63571 66	0.53395 34	SLC18A2/SLC39A14/SLC1A3/SLC11A1/SLC6A4		5
MF	GO:0016810	hydrolase activity, acting on carbon-nitrogen (but not	3/347	131/18369	0.45126	0.63571 66	0.53395 34	AMPD1/DPYSL3/MTHFD2		3

		peptide) bonds							
MF	GO:0016798	hydrolase activity, acting on glycosyl bonds	3/347	132/18369	0.45618 25	0.63771 41	0.53563 12	LYZ/CHIT1/MGAM	3
MF	GO:0017147	Wnt-protein binding	1/347	32/18369	0.45708 35	0.63771 41	0.53563 12	FRZB	1
MF	GO:0019239	deaminase activity	1/347	32/18369	0.45708 35	0.63771 41	0.53563 12	AMPD1	1
MF	GO:0031369	translation initiation factor binding	1/347	32/18369	0.45708 35	0.63771 41	0.53563 12	EIF4EBP1	1
MF	GO:0015171	amino acid transmembran	2/347	83/18369	0.46682 25	0.64973 61	0.54572 87	SLC7A11/SLC1A3	2

		e transporter activity							
MF	GO:0030295	protein kinase activator activity	3/347	135/18369	0.4708366	0.6537516	0.5491014	CCL5/STAP1/CD79A	3
MF	GO:0019902	phosphatase binding	4/347	187/18369	0.4720291	0.6538394	0.5491752	NFATC2/SYTL2/SIGLEC10/SKAP1	4
MF	GO:0015035	protein-disulfide reductase activity	1/347	34/18369	0.4774374	0.6581816	0.5528223	STAB1	1
MF	GO:0016831	carboxy-lyase activity	1/347	34/18369	0.4774374	0.6581816	0.5528223	HDC	1
MF	GO:00	flavin adenine	2/347	85/18369	0.479	0.659	0.553	NQO2/AOX1	2

	50660	dinucleotide binding			2115	058	5584		
MF	GO:0003714	transcription corepressor activity	4/347	190/18369	0.4843197	0.6639116	0.5576351	BTG2/TRIB3/ENO1/SDR16C5	4
MF	GO:0051117	ATPase binding	2/347	86/18369	0.4853397	0.6639116	0.5576351	ANK2/TRPC1	2
MF	GO:0000217	DNA secondary structure binding	1/347	35/18369	0.4873272	0.6639116	0.5576351	HMGB3	1
MF	GO:0072349	modified amino acid transmembrane transpo	1/347	35/18369	0.4873272	0.6639116	0.5576351	SLC7A11	1

		rter activity							
MF	GO:0015276	ligand-gated monoatomic ion channel activity	3/347	140/18369	0.4948543	0.6650627	0.5586019	RYR2/SCNN1G/CFTR	3
MF	GO:0022834	ligand-gated channel activity	3/347	140/18369	0.4948543	0.6650627	0.5586019	RYR2/SCNN1G/CFTR	3
MF	GO:0004623	phospholipase A2 activity	1/347	36/18369	0.4970303	0.6650627	0.5586019	PLA2G2A	1
MF	GO:0005343	organic acid:sodium symporter activity	1/347	36/18369	0.4970303	0.6650627	0.5586019	SLC1A3	1

MF	GO:0030291	protein serine/threonine kinase inhibitor activity	1/347	36/18369	0.4970303	0.66506627	0.5586019	SFN	1
MF	GO:0042805	actinin binding	1/347	36/18369	0.4970303	0.66506627	0.5586019	PDLIM3	1
MF	GO:0043394	proteoglycan binding	1/347	36/18369	0.4970303	0.66506627	0.5586019	TNC	1
MF	GO:0019209	kinase activator activity	3/347	141/18369	0.4995931	0.66506627	0.5586019	CCL5/STAP1/CD79A	3
MF	GO:0015085	calcium ion transmembrane transpo	3/347	142/18369	0.5043093	0.66506627	0.5586019	TRPC6/RYR2/TRPC1	3

		rter activity							
MF	GO:0031625	ubiquitin protein ligase binding	6/347	301/18369	0.50470774	0.66506627	0.55860019	TRIB3/CBS/JUN/EGR2/PYHIN1/TRIB1	6
MF	GO:0001530	lipopolysaccharide binding	1/347	37/18369	0.50655503	0.66506627	0.55860019	TRIL	1
MF	GO:0016409	palmitoyltransferase activity	1/347	37/18369	0.50655503	0.66506627	0.55860019	ZDHHC9	1
MF	GO:0017080	sodium channel regulator activity	1/347	37/18369	0.50655503	0.66506627	0.55860019	FXYD1	1
MF	GO:0035198	miRNA binding	1/347	37/18369	0.50655503	0.66506627	0.55860019	SOCS3	1

MF	GO:0045505	dynein intermediate chain binding	1/347	37/18369	0.506550 3	0.665060 27	0.558601 9	DNAH5	1
MF	GO:0140318	protein transporter activity	1/347	37/18369	0.506550 3	0.665060 27	0.558601 9	AZGP1	1
MF	GO:0008194	UDP-glycosyltransferase activity	3/347	143/18369	0.509002 6	0.666770 4	0.560036 2	GLT8D2/GXYLT2/HAS2	3
MF	GO:0003755	peptidyl-prolyl cis-trans isomerase activity	1/347	38/18369	0.515890 7	0.669732 5	0.562524 2	FKBP5	1
MF	GO:0005501	retinoid binding	1/347	38/18369	0.515890 7	0.669732 5	0.562524 2	PTGDS	1

MF	GO:0019840	isoprenoid binding	1/347	38/18369	0.5158907	0.6697325	0.5625242	PTGDS	1
MF	GO:0140828	metal cation:monoatomic cation antiporter activity	1/347	38/18369	0.5158907	0.6697325	0.5625242	SLC11A1	1
MF	GO:0004867	serine-type endopeptidase inhibitor activity	2/347	92/18369	0.5211499	0.673123	0.5653719	SERPINF2/SERPINA3	2
MF	GO:0008227	G protein-coupled amine recepto	1/347	39/18369	0.5250547	0.673123	0.5653719	HRH2	1

		r activity							
MF	GO:0015036	disulfide oxidoreductase activity	1/347	39/18369	0.52505547	0.673123	0.56537 19	STAB1	1
MF	GO:0016620	oxidoreductase activity, acting on the aldehyde or oxo group of donors, NAD or NADP as acceptor	1/347	39/18369	0.52505547	0.673123	0.56537 19	AKR1C3	1

MF	GO:0016712	oxidoreductase activity, acting on paired donors, with incorporation or reduction of molecular oxygen, reduced flavin or flavoprotein as one donor, and	1/347	39/18369	0.5250547	0.6731123	0.5653719	HMOX1	1

		incorporation of one atom of oxygen							
MF	GO:0030674	protein-macromolecule adaptor activity	7/347	361/18369	0.525477	0.6731123	0.5653719	ANK2/STAP1/TRAT1/DTL/CD3G/CD3E/COL14A1	7
MF	GO:0008200	ion channel inhibitor activity	1/347	40/18369	0.5340458	0.6810848	0.5720592	CFTR	1
MF	GO:0016790	thiolester hydrolase activity	1/347	40/18369	0.5340458	0.6810848	0.5720592	FASN	1
MF	GO:0005272	sodium channel activity	1/347	41/18369	0.5428671	0.6862883	0.5764298	SCNN1G	1

MF	GO:0016248	channel inhibitor activity	1/347	41/18369	0.5428671	0.6862883	0.5764298	CFTR	1
MF	GO:0016859	cis-trans isomerase activity	1/347	41/18369	0.5428671	0.6862883	0.5764298	FKBP5	1
MF	GO:0140662	ATP-dependent protein folding chaperone	1/347	41/18369	0.5428671	0.6862883	0.5764298	HYOU1	1
MF	GO:0030547	signaling receptor inhibitor activity	1/347	42/18369	0.5515219	0.6941982	0.5830735	CCL5	1

MF	GO:0050136	NADH dehydrogenase (quinone) activity	1/347	42/18369	0.5515219	0.6941982	0.5830735	NQO1	1
MF	GO:0001046	core promoter sequence-specific DNA binding	1/347	43/18369	0.5600133	0.7018348	0.5894877	FOS	1
MF	GO:0015144	carbohydrate transmembrane transporter activity	1/347	43/18369	0.5600133	0.7018348	0.5894877	AQP9	1
MF	GO:00	ubiquitin-like protein	6/347	320/18369	0.564	0.706	0.592	TRIB3/CBS/JUN/EGR2/PYHIN1/TRIB1	6

	44389	ligase binding			5583	0028	9884		
MF	GO:0003954	NADH dehydrogenase activity	1/347	44/18369	0.5683444	0.7076804	0.5943975	NQO1	1
MF	GO:0004864	protein phosphatase inhibitor activity	1/347	44/18369	0.5683444	0.7076804	0.5943975	PPP1R10	1
MF	GO:0005216	monoatomic ion channel activity	8/347	432/18369	0.5726287	0.711485	0.5975931	TRPC6/RYR2/SCNN1G/FXYD1/KCNAB1/TRPC1/ANO1/CFTR	8
MF	GO:0001222	transcription corepressor binding	1/347	45/18369	0.5765182	0.7117356	0.5978036	ENO1	1

MF	GO:0004712	protein serine/threonine/tyrosine kinase activity	1/347	45/18369	0.5765182	0.7117356	0.5978036	RPS6KA2	1
MF	GO:0032266	phosphatidylinositol-3-phosphate binding	1/347	45/18369	0.5765182	0.7117356	0.5978036	SH3PXD2B	1
MF	GO:0051087	chaperone binding	2/347	104/18369	0.5876122	0.7238882	0.6080108	CFTR/CP	2
MF	GO:0019212	phosphatase inhibitor activity	1/347	47/18369	0.5924056	0.7282438	0.6116692	PPP1R10	1
MF	GO:00	coreceptor activity	1/347	48/18369	0.600	0.731	0.614	ITGA4	1

	15026				125	5208	4216		
MF	GO:0016879	ligase activity, forming carbon-nitrogen bonds	1/347	48/18369	0.600125	0.7315208	0.6144216	GCLM	1
MF	GO:0017046	peptide hormone binding	1/347	48/18369	0.600125	0.7315208	0.6144216	INHBA	1
MF	GO:0032813	tumor necrosis factor receptor superfamily binding	1/347	48/18369	0.600125	0.7315208	0.6144216	TNFSF10	1
MF	GO:0000149	SNARE binding	2/347	107/18369	0.6031243	0.7320942	0.6149033	TXLNB/SLC6A4	2

MF	GO:1902936	phosphatidylinositol bisphosphate binding	2/347	107/18369	0.6031243	0.7320942	0.6149033	SYTL2/SH3PXD2B	2
MF	GO:0016763	pentosyltransferase activity	1/347	49/18369	0.6076987	0.7351683	0.6174853	GXYLT2	1
MF	GO:0099094	ligand-gated monoatomic cation channel activity	2/347	108/18369	0.6081962	0.7351683	0.6174853	RYR2/SCNN1G	2
MF	GO:0015485	cholesterol binding	1/347	50/18369	0.6151293	0.7389208	0.6206371	SIDT1	1
MF	GO:0015932	nucleobase-containing	1/347	50/18369	0.6151293	0.7389208	0.6206371	SIDT1	1

		compound transmembrane transporter activity							
MF	GO:0016504	peptidase activator activity	1/347	50/18369	0.6151293	0.7389208	0.6206371	NLRC4	1
MF	GO:0051018	protein kinase A binding	1/347	51/18369	0.6224195	0.7438855	0.624807	RYR2	1
MF	GO:0061980	regulatory RNA binding	1/347	51/18369	0.6224195	0.7438855	0.624807	SOCS3	1
MF	GO:0005200	structural constituent of	2/347	111/18369	0.6231165	0.7438855	0.624807	ANK2/KRT19	2

		cytoskeleton							
MF	GO:0008146	sulfotransferase activity	1/347	52/18369	0.6295721	0.7500457	0.6299811	SULT1B1	1
MF	GO:0015459	potassium channel regulator activity	1/347	53/18369	0.6365895	0.7537532	0.6330951	KCNAB1	1
MF	GO:0050997	quaternary ammonium group binding	1/347	53/18369	0.6365895	0.7537532	0.6330951	RASGRP1	1
MF	GO:0140296	general transcription initiation factor binding	1/347	53/18369	0.6365895	0.7537532	0.6330951	JUN	1

MF	GO:0101005	deubiquitinase activity	2/347	115/18369	0.642323	0.7557235	0.63475	STAMBPL1/OTUD1	2
MF	GO:0005246	calcium channel regulator activity	1/347	54/18369	0.6434744	0.7557235	0.63475	GEM	1
MF	GO:0008374	O-acyltransferase activity	1/347	54/18369	0.6434744	0.7557235	0.63475	PIGW	1
MF	GO:0016667	oxidoreductase activity, acting on a sulfur group of donors	1/347	54/18369	0.6434744	0.7557235	0.63475	STAB1	1

MF	GO:0031593	polyubiquitin modification-dependent protein binding	1/347	55/18369	0.650229 2	0.762110 7	0.640114 8	TNIP3	1
MF	GO:0004197	cysteine-type endopeptidase activity	2/347	118/18369	0.656216 4	0.766647 1	0.643925	CTSF/CAPN3	2
MF	GO:0042578	phosphoric ester hydrolase activity	6/347	352/18369	0.656747 8	0.766647 1	0.643925	PLCB4/EPHX2/PDE7B/CCL5/ALPL/HMOX1	6
MF	GO:1901981	phosphatidylinositol phosphate binding	3/347	179/18369	0.660526 9	0.769507 2	0.646327 3	SYTL2/FGD2/SH3PXD2B	3

MF	GO:0070063	RNA polymerase binding	1/347	57/18369	0.6633584	0.76970084	0.64649663	ZFP36	1
MF	GO:1901682	sulfur compound transmembrane transporter activity	1/347	57/18369	0.6633584	0.76970084	0.64649663	SLC7A11	1
MF	GO:0032934	sterol binding	1/347	59/18369	0.67599961	0.78280835	0.65749952	SIDT1	1
MF	GO:0008234	cysteine-type peptidase activity	3/347	185/18369	0.68221668	0.7868834	0.66088805	CTSF/OTUD1/CAPN3	3
MF	GO:0004540	ribonuclease activity	2/347	124/18369	0.68270773	0.7868834	0.66088805	RNASE2/AZGP1	2

MF	GO:0051020	GTPase binding	5/347	304/18369	0.683553636	0.786834	0.660880 5	ENO1/SYTL2/FGD2/RABGAP1L/RHOH	5
MF	GO:0001786	phosphatidylserine binding	1/347	62/18369	0.694070711	0.794719 2	0.667503 4	SYTL2	1
MF	GO:0022836	gated channel activity	5/347	308/18369	0.694521 4	0.794719 2	0.667503 4	RYR2/SCNN1G/KCNAB1/ANO1/CFTR	5
MF	GO:0022839	monoatomic ion gated channel activity	5/347	308/18369	0.694521 4	0.794719 2	0.667503 4	RYR2/SCNN1G/KCNAB1/ANO1/CFTR	5
MF	GO:0031072	heat shock protein binding	2/347	128/18369	0.699425 7	0.797686 3	0.669995 6	ZFP36/FKBP5	2
MF	GO:19	promoter-specific	1/347	63/18369	0.699	0.797	0.669	EGR1	1

	90841	chromatin binding			8699	6863	9956		
MF	GO:0019783	ubiquitin-like protein peptidase activity	2/347	130/18369	0.707508	0.8048077	0.675977	STAMBPL1/OTUD1	2
MF	GO:0003779	actin binding	7/347	434/18369	0.7157403	0.8113359	0.6814602	PLEKHH2/FMN1/UACA/MYH10/MYH11/PDLIM3/SLC6A4	7
MF	GO:0016758	hexosyltransferase activity	3/347	195/18369	0.7160495	0.8113359	0.6814602	FUT3/TMTC1/HAS2	3
MF	GO:0052689	carboxylic ester hydrolase activity	2/347	133/18369	0.7192912	0.8134172	0.6832084	LIPG/PLA2G2A	2
MF	GO:00	protein folding	1/347	67/18369	0.721	0.814	0.684	HYOU1	1

	44183	chaperone			9892	8767	4342		
MF	GO:0005525	GTP binding	6/347	382/18369	0.7313592	0.8218564	0.6902966	GEM/SRPRB/RHOBTB2/RHOH/GBP5/GBP4	6
MF	GO:0051536	iron-sulfur cluster binding	1/347	69/18369	0.7324316	0.8218564	0.6902966	AOX1	1
MF	GO:0051540	metal cluster binding	1/347	69/18369	0.7324316	0.8218564	0.6902966	AOX1	1
MF	GO:0005261	monoatomic cation channel activity	5/347	324/18369	0.7357659	0.8233551	0.6915554	TRPC6/RYR2/SCNN1G/KCNAB1/TRPC1	5
MF	GO:0016782	transferase activity, transferring sulphur	1/347	70/18369	0.7375051	0.8233551	0.6915554	SULT1B1	1

		-containing groups							
MF	GO:0051015	actin filament binding	3/347	202/18369	0.7380333	0.8233551	0.6915554	MYH10/MYH11/SLC6A4	3
MF	GO:0015078	proton transmembrane transporter activity	2/347	140/18369	0.7452387	0.8297946	0.6969641	SLC18A2/SLC11A1	2
MF	GO:0003777	microtubule motor activity	1/347	73/18369	0.7521574	0.8342896	0.7007395	DNAH5	1
MF	GO:0019003	GDP binding	1/347	73/18369	0.7521574	0.8342896	0.7007395	GEM	1
MF	GO:00	RNA endonu	1/347	74/18369	0.756	0.837	0.703	RNASE2	1

	04521	clease activity			858	8982	7705		
MF	GO:0031267	small GTPase binding	4/347	273/18369	0.7611601	0.8410529	0.7064202	SYTL2/FGD2/RABGAP1L/RHOH	4
MF	GO:0005096	GTPase activator activity	4/347	280/18369	0.7781565	0.8551303	0.7182442	JUN/TAGAP/RGS5/RABGAP1L	4
MF	GO:0019001	guanyl nucleotide binding	6/347	404/18369	0.7783311	0.8551303	0.7182442	GEM/SRPRB/RHOBTB2/RHOH/GBP5/GBP4	6
MF	GO:0032561	guanyl ribonucleotide binding	6/347	404/18369	0.7783311	0.8551303	0.7182442	GEM/SRPRB/RHOBTB2/RHOH/GBP5/GBP4	6
MF	GO:0005546	phosphatidylinositol-4,5-bisphosphate binding	1/347	82/18369	0.7913399	0.8678841	0.7289202	SYTL2	1

MF	GO:0030165	PDZ domain binding	1/347	83/18369	0.79535573	0.87053328	0.7311811	CFTR	1
MF	GO:0005543	phospholipid binding	7/347	475/18369	0.7969134	0.8705903	0.73122294	SGIP1/SYTL2/STAP1/FGD2/SH3PXD2B/RASGRP1/PLA2G2A	7
MF	GO:0042562	hormone binding	1/347	84/18369	0.7992406	0.87148884	0.73198837	INHBA	1
MF	GO:0016747	acyltransferase activity, transferring groups other than amino-acyl groups	3/347	226/18369	0.80328283	0.87425555	0.73430078	PIGW/FASN/ZDHHC9	3

MF	GO:0005085	guanyl-nucleotide exchange factor activity	3/347	227/18369	0.80556845	0.87521183	0.73511165	TAGAP/FGD2/RASGRP1	3
MF	GO:0046943	carboxylic acid transmembrane transporter activity	2/347	162/18369	0.81378809	0.87772219	0.73721194	SLC7A11/SLC1A3	2
MF	GO:0005249	voltage-gated potassium channel activity	1/347	88/18369	0.8140553	0.87772219	0.73721194	KCNAB1	1
MF	GO:00	beta-catenin binding	1/347	88/18369	0.814	0.877	0.737	NR4A2	1

	08013				053	7219	2194		
MF	GO:0140104	molecular carrier activity	1/347	88/18369	0.814053	0.8777219	0.7372194	HBB	1
MF	GO:0005342	organic acid transmembrane transporter activity	2/347	163/18369	0.8164664	0.8786879	0.7380307	SLC7A11/SLC1A3	2
MF	GO:0019888	protein phosphatase regulator activity	1/347	92/18369	0.8277753	0.8878402	0.7457179	PPP1R10	1
MF	GO:0030695	GTPase regulator activity	7/347	495/18369	0.8295709	0.8878402	0.7457179	JUN/TAGAP/RGS5/FGD2/RABGAP1L/RHOH/RASGRP1	7

MF	GO:0060589	nucleoside-triphosphatase regulator activity	7/347	495/18369	0.829570 9	0.887840 2	0.745717 9	JUN/TAGAP/RGS5/FGD2/RABGAP1L/RHOH/RASGRP1	7
MF	GO:0016407	acetyltransferase activity	1/347	94/18369	0.834253	0.891203 9	0.748543 2	FASN	1
MF	GO:0015631	tubulin binding	5/347	374/18369	0.838218 9	0.893791 4	0.750716 6	SGIP1/AGBL1/FMN1/S100A8/TPPP3	5
MF	GO:0140030	modification-dependent protein binding	2/347	175/18369	0.846090 4	0.899297	0.755340 8	ANK2/TNIP3	2
MF	GO:0030594	neurotransmitter recepto	1/347	98/18369	0.846488 6	0.899297	0.755340 8	HRH2	1

		r activity								
MF	GO:0005496	steroid binding	1/347	99/18369	0.849404	0.900 7416	0.7565542	SIDT1		1
MF	GO:0016746	acyltransferase activity	3/347	253/18369	0.8598488	0.9101507	0.7644571	PIGW/FASN/ZDHHC9		3
MF	GO:0005244	voltage-gated monoatomic ion channel activity	2/347	189/18369	0.8751283	0.9201872	0.772887	KCNAB1/ANO1		2
MF	GO:0022832	voltage-gated channel activity	2/347	189/18369	0.8751283	0.9201872	0.772887	KCNAB1/ANO1		2
MF	GO:00	cysteine-type deubiq	1/347	109/18369	0.875	0.920	0.772	OTUD1		1

	04843	uitinase activity			6877	1872	887		
MF	GO:0090079	translation regulator activity, nucleic acid binding	1/347	109/18369	0.8756877	0.9201872	0.7728887	SHMT2	1
MF	GO:0045296	cadherin binding	4/347	333/18369	0.8775891	0.9205147	0.773162	ENO1/IDH1/FASN/SFN	4
MF	GO:0019208	phosphatase regulator activity	1/347	111/18369	0.8803676	0.9217592	0.7742073	PPP1R10	1
MF	GO:0008017	microtubule binding	3/347	268/18369	0.8846849	0.9246075	0.7765997	SGIP1/FMN1/S100A8	3
MF	GO:00	protein heterodimeriza	4/347	343/18369	0.891	0.929	0.780	P2RY1/ATF3/NR4A1/NR4A2	4

	46982	tion activity			274	8155	974		
MF	GO:0035091	phosphatidylinositol binding	3/347	275/18369	0.8948752	0.9318934	0.7827193	SYTL2/FGD2/SH3PXD2B	3
MF	GO:0005267	potassium channel activity	1/347	121/18369	0.9012597	0.9337795	0.7843035	KCNAB1	1
MF	GO:0004519	endonuclease activity	1/347	122/18369	0.9031373	0.9337795	0.7843035	RNASE2	1
MF	GO:0005179	hormone activity	1/347	122/18369	0.9031373	0.9337795	0.7843035	INHBA	1
MF	GO:0051082	unfolded protein binding	1/347	122/18369	0.9031373	0.9337795	0.7843035	HYOU1	1
MF	GO:00	nuclease activity	2/347	207/18369	0.905	0.934	0.784	RNASE2/AZGP1	2

	04518				0593	0986	5715		
MF	GO:0019903	protein phosphatase binding	1/347	140/18369	0.9314521	0.9579233	0.8045824	SKAP1	1
MF	GO:0022843	voltage-gated monoatomic cation channel activity	1/347	140/18369	0.9314521	0.9579233	0.8045824	KCNAB1	1
MF	GO:0015079	potassium ion transmembrane transporter activity	1/347	157/18369	0.9505648	0.9758458	0.8196336	KCNAB1	1
MF	GO:0043021	ribonucleoprotein comple	1/347	161/18369	0.9542264	0.977871	0.8213369	WDR12	1

		x binding							
MF	GO:0016874	ligase activity	1/347	165/18369	0.95761 75	0.97957 04	0.82276 44	GCLM	1
MF	GO:0004674	protein serine/threonine kinase activity	4/347	421/18369	0.95926 85	0.97957 04	0.82276 44	RPS6KA2/LTBP4/PIM2/PIK3CG	4
MF	GO:0016791	phosphatase activity	2/347	265/18369	0.96198 86	0.98061 86	0.82364 48	EPHX2/ALPL	2
MF	GO:0106310	protein serine kinase activity	3/347	363/18369	0.96929 59	0.98633 1	0.82844 27	RPS6KA2/PIM2/PIK3CG	3
MF	GO:0003713	transcription coactivator activity	2/347	282/18369	0.97115 55	0.98648 95	0.82857 59	ARID5B/JUND	2

MF	GO:0016887	ATP hydrolysis activity	3/347	376/18369	0.974629	0.988284	0.830083	ABCA8/ALPL/CFTR	3
MF	GO:0030371	translation repressor activity	2/347	302/18369	0.979229	0.991212	0.832543	SHMT2/EIF4EBP1	2
MF	GO:0016741	transferase activity, transferring one-carbon groups	1/347	221/18369	0.985598	0.995919	0.836495	SHMT2	1
MF	GO:0003730	mRNA 3'-UTR binding	1/347	252/18369	0.992083	0.997872	0.839749	ZFP36	1
MF	GO:0140098	catalytic activity, acting	2/347	396/18369	0.995713	0.997872	0.839749	RNASE2/AZGP1	2

		on RNA							
MF	GO:0045182	translation regulator activity	2/347	418/18369	0.9970944	0.9997872	0.8397449	SHMT2/EIF4EBP1	2
MF	GO:0061659	ubiquitin-like protein ligase activity	1/347	341/18369	0.9985909	0.9997872	0.8397449	EGR2	1
MF	GO:0019787	ubiquitin-like protein transferase activity	2/347	467/18369	0.9987653	0.9997872	0.8397449	EGR2/DTL	2
MF	GO:0004842	ubiquitin-protein transferase activity	1/347	438/18369	0.9997872	0.9997872	0.8397449	DTL	1

BP – biological process, CC – cellular component, DEG – differentially expressed gene, GO – Gene Ontology, MF – molecular function.

Table S6. KEGG pathway enrichment analysis for the identified DEGs.

ID	Description	Gene ratio	Background ratio	P-value	Adjusted P-value	q-value	Gene ID	Count
hsa04640	Hematopoietic cell lineage	19/210	99/8644	1.81E-12	4.86E-10	3.74E-10	CD1C/HLA-DPA1/HLA-DOA/HLA-DOB/ITGA4/MS4A1/CD1E/IL1B/IL1R2/CD5/CD3G/ANPEP/CD2/CD3E/IL6/CD3D/HLA-DQA1/CSF3/HLA-DQB1	19
hsa04061	Viral protein interaction with cytokine and cytokine receptor	16/210	100/8644	1.85E-09	2.48E-07	1.91E-07	CXCL12/CCL5/IL2RG/CCL23/IL2RB/TNFSF10/IL6/CXCL1/CXCL2/CX3CR1/CX3CL1/CCL2/CXCL10/CCL8/CXCL9/CCL18	16
hsa05323	Rheumatoid arthritis	15/210	93/8644	5.40E-09	4.21E-07	3.24E-07	CXCL12/HLA-DPA1/TGFB2/HLA-DOA/HLA-DOB/JUN/CCL5/IL1B/FOS/IL6/CXCL1/CXCL2/HLA-DQA1/CCL2/HLA-DQB1	15
hsa04657	IL-17 signaling pathway	15/210	94/8644	6.29E-09	4.21E-07	3.24E-07	PTGS2/IL17RB/JUN/FOSB/JUND/IL1B/LCN2/S100A8/FOS/IL6/CXCL1/CXCL2/CCL2/CSF3/CXCL10	15
hsa04668	TNF signaling pathway	16/210	114/8644	1.31E-08	7.04E-07	5.42E-07	PTGS2/JUN/CCL5/JUNB/IL1B/VCAM1/FOS/IRF1/IL6/CXCL1/CXCL2/CX3CL1/SOCS3/CCL2/CXCL10/SELE	16

hsa04658	Th1 and Th2 cell differentiation	14/210	92/8644	3.83E-08	1.66E-06	1.28E-06	HLA-DPA1/NFATC2/STAT4/HLA-DOA/HLA-DOB/JUN/IL2RG/IL2RB/FOS/CD3G/CD3E/CD3D/HLA-DQA1/HLA-DQB1	14
hsa04659	Th17 cell differentiation	15/210	108/8644	4.39E-08	1.66E-06	1.28E-06	HLA-DPA1/NFATC2/HLA-DOA/HLA-DOB/JUN/IL2RG/IL1B/IL2RB/FOS/CD3G/CD3E/IL6/CD3D/HLA-DQA1/HLA-DQB1	15
hsa04060	Cytokine-cytokine receptor interaction	25/210	297/8644	4.95E-08	1.66E-06	1.28E-06	CXCL12/BMP5/TGFB2/IL17RB/CCL5/IL2RG/TNFRSF11B/IL1B/CCL23/IL2RB/IL1R2/CD27/INHBA/TNFSF10/IL6/CXCL1/CXCL2/CX3CR1/CX3CL1/CCL2/CSF3/CXCL10/CCL8/CXCL9/CCL18	25
hsa05321	Inflammatory bowel disease	11/210	65/8644	3.80E-07	1.13E-05	8.70E-06	HLA-DPA1/TGFB2/STAT4/HLA-DOA/HLA-DOB/JUN/IL2RG/IL1B/IL6/HLA-DQA1/HLA-DQB1	11
hsa05166	Human T-cell leukemia virus 1 infection	19/210	222/8644	1.72E-06	4.62E-05	3.56E-05	HLA-DPA1/NFATC2/TGFB2/EGR1/HLA-DOA/HLA-DOB/JUN/EGR2/IL2RG/ZFP36/IL2RB/IL1R2/FOS/CD3G/CD3E/IL6/CD3D/HLA-DQA1/HLA-DQB1	19
hsa05140	Leishmaniasis	11/210	77/8644	2.20E-06	4.90E-05	3.78E-05	HLA-DPA1/PTGS2/TGFB2/HLA-DOA/HLA-DOB/JUN/ITGA4/IL1B/FOS/HLA-DQA1/HLA-DQB1	11

hsa04672	Intestinal immune network for IgA production	9/210	49/8644	2.20E-06	4.90E-05	3.78E-05	CXCL12/HLA-DPA1/HLA-DOA/HLA-DOB/ITGA4/ICOS/IL6/HLA-DQA1/HLA-DQB1	9
hsa05144	Malaria	9/210	50/8644	2.62E-06	5.41E-05	4.16E-05	HGF/HBB/TGFB2/IL1B/VCAM1/IL6/CCL2/CSF3/SELE	9
hsa05142	Chagas disease	12/210	102/8644	6.11E-06	0.0001127	8.68E-05	PLCB4/TGFB2/JUN/CCL5/ACE/IL1B/FOS/CD3G/CD3E/IL6/CD3D/CCL2	12
hsa04514	Cell adhesion molecules	15/210	158/8644	6.31E-06	0.0001127	8.68E-05	HLA-DPA1/TIGIT/HLA-DOA/HLA-DOB/CNTN1/VCAN/ITGA4/ICOS/SIGLEC1/VCAM1/NRXN3/CD2/HLA-DQA1/HLA-DQB1/SELE	15
hsa05143	African trypanosomiasis	7/210	37/8644	2.50E-05	0.0004194	0.0003228	PLCB4/HBB/IL1B/VCAM1/IL6/SELE/IDO1	7
hsa05332	Graft-versus-host disease	7/210	42/8644	5.92E-05	0.000933	0.0007182	HLA-DPA1/HLA-DOA/HLA-DOB/IL1B/IL6/HLA-DQA1/HLA-DQB1	7

hsa04062	Chemokine signaling pathway	15/210	192/8644	6.42E-05	0.0009557	0.0007357	PLCB4/CXCL12/PIK3CG/CCL5/FGR/CCL23/CXCL1/CXCL2/CX3CR1/CX3CL1/CCL2/CXCL10/CCL8/CXCL9/CCL18	15
hsa04940	Type I diabetes mellitus	7/210	43/8644	6.92E-05	0.0009768	0.000752	HLA-DPA1/HLA-DOA/HLA-DOB/IL1B/CPE/HLA-DQA1/HLA-DQB1	7
hsa01230	Biosynthesis of amino acids	9/210	75/8644	7.77E-05	0.0010415	0.0008018	PSAT1/SHMT2/ENO1/PYCR1/CBS/IDH1/PHGDH/BCAT1/CTH	9
hsa05310	Asthma	6/210	31/8644	8.48E-05	0.0010823	0.0008332	HLA-DPA1/HLA-DOA/HLA-DOB/MS4A2/HLA-DQA1/HLA-DQB1	6
hsa04380	Osteoclast differentiation	12/210	135/8644	0.0001035	0.0012296	0.0009466	NFATC2/TGFB2/JUN/FOSB/JUNB/JUND/TNFRSF11B/IL1B/FCGR2B/FOS/LILRA6/SOCS3	12
hsa05150	Staphylococcus aureus infection	10/210	96/8644	0.0001055	0.0012296	0.0009466	HLA-DPA1/HLA-DOA/HLA-DOB/FPR1/KRT19/FCGR2B/C1R/FPR2/HLA-DQA1/HLA-DQB1	10

hsa00260	Glycine, serine and threonine metabolism	6/210	40/8644	0.0003685	0.0041148	0.0031677	PSAT1/SHMT2/CBS/PHGDH/CTH/MAOA	6
hsa05418	Fluid shear stress and atherosclerosis	11/210	139/8644	0.0005511	0.005908	0.0045482	GSTM5/JUN/HMOX1/NQO1/IL1B/VCAM1/IL1R2/FOS/GSTT1/CCL2/SELE	11
hsa00590	Arachidonic acid metabolism	7/210	61/8644	0.0006478	0.0066777	0.0051407	PTGS2/PTGDS/EPHX2/HPGDS/AKR1C3/ALOX15B/PLA2G2A	7
hsa04933	AGE-RAGE signaling pathway in diabetic complications	9/210	100/8644	0.0006976	0.0069242	0.0053305	PLCB4/TGFB2/EGR1/JUN/IL1B/VCAM1/IL6/CCL2/SELE	9
hsa05146	Amoebiasis	9/210	102/8644	0.0008056	0.0077107	0.005936	PLCB4/CD1C/TGFB2/CD1E/IL1B/IL1R2/IL6/CXCL1/CXCL2	9
hsa05164	Influenza A	12/210	171/8644	0.0009163	0.0082799	0.0063742	HLA-DPA1/HLA-DOA/HLA-DOB/CCL5/IL1B/TNFSF10/IL6/HLA-DQA1/SOCS3/CCL2/HLA-DQB1/CXCL10	12

hsa04625	C-type lectin receptor signaling pathway	9/210	104/8644	0.0009269	0.0082799	0.0063742	NFATC2/PTGS2/JUN/EGR2/EGR3/IL1B/IRF1/IL6/CLEC4E	9
hsa00270	Cysteine and methionine metabolism	6/210	52/8644	0.0015346	0.0132672	0.0102135	PSAT1/GCLM/CBS/PHGDH/BCAT1/CTH	6
hsa04621	NOD-like receptor signaling pathway	12/210	186/8644	0.0018897	0.0155294	0.0119551	PLCB4/NAIP/JUN/CCL5/NLRC4/IL1B/IL6/CXCL1/CXCL2/GBP5/GBP4/CCL2	12
hsa05162	Measles	10/210	138/8644	0.0019122	0.0155294	0.0119551	JUN/IL2RG/IL1B/IL2RB/FCGR2B/FOS/CD3G/CD3E/IL6/CD3D	10
hsa05330	Allograft rejection	5/210	38/8644	0.0021083	0.0161439	0.0124281	HLA-DPA1/HLA-DOA/HLA-DOB/HLA-DQA1/HLA-DQB1	5
hsa05340	Primary immunodeficiency	5/210	38/8644	0.0021083	0.0161439	0.0124281	CD79A/IL2RG/ICOS/CD3E/CD3D	5

hsa05417	Lipid and atherosclerosis	13/210	215/8644	0.0022074	0.0163594	0.012594	PLCB4/NFATC2/JUN/CCL5/IL1B/VCAM1/FOS/TNFSF10/IL6/CXCL1/CXCL2/CCL2/SELE	13
hsa05134	Legionellosis	6/210	56/8644	0.0022586	0.0163594	0.012594	NAIP/NLRC4/IL1B/IL6/CXCL1/CXCL2	6
hsa00480	Glutathione metabolism	6/210	57/8644	0.0024742	0.0174493	0.0134331	GSTM5/GCLM/IDH1/HPGDS/ANPEP/GSTT1	6
hsa04216	Ferroptosis	5/210	41/8644	0.0029679	0.0203946	0.0157004	GCLM/SLC39A14/HMOX1/SLC7A11/CP	5
hsa05235	PD-L1 expression and PD-1 checkpoint pathway in cancer	7/210	89/8644	0.0057823	0.0387413	0.0298244	NFATC2/JUN/RASGRP1/FOS/CD3G/CD3E/CD3D	7
hsa05135	Yersinia infection	9/210	137/8644	0.006086	0.0397819	0.0306255	RPS6KA2/NFATC2/JUN/ITGA4/NLRC4/IL1B/FOS/IL6/CCL2	9

hsa00750	Vitamin B6 metabolism	2/210	6/8644	0.0082615	0.0527164	0.040583	PSAT1/AOX1	2
hsa05320	Autoimmune thyroid disease	5/210	53/8644	0.0089909	0.0557757	0.0429381	HLA-DPA1/HLA-DOA/HLA-DOB/HLA-DQA1/HLA-DQB1	5
hsa04660	T cell receptor signaling pathway	8/210	121/8644	0.0091572	0.0557757	0.0429381	NFATC2/JUN/ICOS/RASGRP1/FOS/CD3G/CD3E/CD3D	8
hsa05169	Epstein-Barr virus infection	11/210	202/8644	0.0101714	0.0594234	0.0457462	HLA-DPA1/HLA-DOA/HLA-DOB/JUN/CD3G/CD3E/IL6/CD3D/HLA-DQA1/HLA-DQB1/CXCL10	11
hsa05133	Pertussis	6/210	76/8644	0.0101995	0.0594234	0.0457462	JUN/IL1B/FOS/C1R/IRF1/IL6	6
hsa01240	Biosynthesis of cofactors	9/210	153/8644	0.0121593	0.0685742	0.0527908	PSAT1/SHMT2/GCLM/SDR16C5/ALPL/MTHFD2/NQO1/BCAT1/IDO1	9

hsa 05 15 2	Tuberculosis	10/ 210	180/8 644	0.0 122 82	0.068 5742	0.0 527 908	HLA-DPA1/TGFB2/HLA-DOA/HLA-DOB/IL1B/FCGR2B/IL6/CLEC4E/HLA-DQA1/HLA-DQB1	1 0
hsa 04 36 0	Axon guidance	10/ 210	182/8 644	0.0 131 963	0.072 1756	0.0 555 633	TRPC6/CXCL12/NFATC2/ROBO2/SEMA3D/TRPC1/SEMA6D/SLIT3/ SEMA6A/SEMA3G	1 0
hsa 04 62 0	Toll-like receptor signaling pathway	7/2 10	108/8 644	0.0 159 395	0.085 0305	0.0 654 594	JUN/CCL5/IL1B/FOS/IL6/CXCL10/CXCL9	7
hsa 04 66 2	B cell receptor signaling pathway	6/2 10	84/86 44	0.0 161 812	0.085 0305	0.0 654 594	NFATC2/JUN/CD79A/FCGR2B/FOS/LILRA6	6
hsa 04 61 4	Renin-angiotensin system	3/2 10	23/86 44	0.0 174 957	0.090 1699	0.0 694 16	CPA3/ACE/ANPEP	3
hsa 05 14 5	Toxoplasmosis	7/2 10	111/8 644	0.0 182 783	0.092 4261	0.0 711 529	HLA-DPA1/TGFB2/PIK3CG/HLA-DOA/HLA-DOB/HLA-DQA1/HLA-DQB1	7

hsa04512	ECM-receptor interaction	6/210	89/8644	0.0209552	0.1039999	0.0800628	FREM1/ITGA10/ITGA4/TNR/COL6A6/TNC	6
hsa04726	Serotonergic synapse	7/210	115/8644	0.0217589	0.1060254	0.0816221	PLCB4/SLC18A2/PTGS2/TRPC1/MAOA/ALOX15B/SLC6A4	7
hsa05416	Viral myocarditis	5/210	67/8644	0.0230724	0.1090557	0.0839549	HLA-DPA1/HLA-DOA/HLA-DOB/HLA-DQA1/HLA-DQB1	5
hsa02010	ABC transporters	4/210	45/8644	0.0231947	0.1090557	0.0839549	ABCA9/ABCA6/ABCA8/CFTR	4
hsa05031	Amphetamine addiction	5/210	69/8644	0.0258344	0.1193727	0.0918973	SLC18A2/JUN/FOSB/FOS/MAOA	5
hsa05204	Chemical carcinogenesis	5/210	70/8644	0.0272914	0.1239678	0.0954347	AKR1C2/GSTM5/PTGS2/HPGDS/GSTT1	5

hsa04151	PI3K-Akt signaling pathway	15/210	359/8644	0.0284346	0.1270078	0.097775	EIF4EBP1/HGF/ITGA10/PIK3CG/ITGA4/TNR/IL2RG/COL6A6/IL2RB/DDIT4/TNC/IL6/NR4A1/NTRK2/CSF3	15
hsa00982	Drug metabolism	5/210	72/8644	0.0303 6	0.1302501	0.1002711	GSTM5/HPGDS/MAOA/AOX1/GSTT1	5
hsa05410	Hypertrophic cardiomyopathy	6/210	97/8644	0.0304378	0.1302501	0.1002711	RYR2/TGFB2/ITGA10/ITGA4/ACE/IL6	6
hsa05030	Cocaine addiction	4/210	49/8644	0.0306185	0.1302501	0.1002711	SLC18A2/JUN/FOSB/MAOA	4
hsa04145	Phagosome	8/210	152/8644	0.0317268	0.1328558	0.1022771	HLA-DPA1/HLA-DOA/HLA-DOB/FCGR2B/C1R/MARCO/HLA-DQA1/HLA-DQB1	8
hsa01523	Antifolate resistance	3/210	30/8644	0.035452	0.1461715	0.1125279	SHMT2/IL1B/IL6	3

hsa04972	Pancreatic secretion	6/210	102/8644	0.0375985	0.1526729	0.1175329	PLCB4/RYR2/TRPC1/CPA3/CFTR/PLA2G2A	6
hsa04064	NF-kappa B signaling pathway	6/210	104/8644	0.0407404	0.1609253	0.1238859	CXCL12/PTGS2/IL1B/VCAM1/CXCL1/CXCL2	6
hsa04612	Antigen processing and presentation	5/210	78/8644	0.0408318	0.1609253	0.1238859	HLA-DPA1/HLA-DOA/HLA-DOB/HLA-DQA1/HLA-DQB1	5
hsa05161	Hepatitis B	8/210	162/8644	0.0436654	0.1695989	0.1305632	NFATC2/TGFB2/STAT4/JUN/EGR2/EGR3/FOS/IL6	8
hsa05208	Chemical carcinogenesis	10/210	223/8644	0.0452181	0.1731209	0.1332745	AKR1C2/GSTM5/HGF/EPHX2/JUN/HMOX1/NQO1/AKR1C3/FOS/GSTT1	10
hsa05167	Kaposi sarcoma-associated herpesvirus infection	9/210	194/8644	0.0468047	0.1766714	0.1360078	NFATC2/PTGS2/PIK3CG/JUN/ZFP36/FOS/IL6/CXCL1/CXCL2	9

hsa05163	Human cytomegalovirus infection	10/210	225/8644	0.0475391	0.1769511	0.1362231	PLCB4/CXCL12/EIF4EBP1/NFATC2/PTGS2/CCL5/IL1B/IL6/CX3CL1/CCL2	10
hsa00350	Tyrosine metabolism	3/210	36/8644	0.0561392	0.203867	0.156944	FAH/MAOA/AOX1	3
hsa05171	Coronavirus disease	10/210	232/8644	0.0562916	0.203867	0.156944	JUN/ACE/IL1B/FOS/C1R/IL6/CCL2/CSF3/FGA/CXCL10	10
hsa04921	Oxytocin signaling pathway	7/210	154/8644	0.0811492	0.2868512	0.2208281	PLCB4/RYR2/NFATC2/PTGS2/PIK3CG/JUN/FOS	7
hsa00380	Tryptophan metabolism	3/210	42/8644	0.0813459	0.2868512	0.2208281	MAOA/AOX1/IDO1	3
hsa00670	One carbon pool by folate	2/210	20/8644	0.0838389	0.2918029	0.2246401	SHMT2/MTHFD2	2

hsa04148	Efferocytosis	7/210	156/8644	0.0855444	0.2939219	0.2262714	NFATC2/PTGS2/SIGLEC10/STAB1/CH25H/CX3CR1/CX3CL1	7
hsa05120	Epithelial cell signaling in Helicobacter pylori infection	4/210	70/8644	0.0900719	0.3055604	0.2352311	JUN/CCL5/CXCL1/CXCL2	4
hsa00290	Valine, leucine and isoleucine biosynthesis	1/210	4/8644	0.0937086	0.3139239	0.2416696	BCAT1	1
hsa00340	Histidine metabolism	2/210	22/8644	0.0988056	0.3269123	0.2516686	HDC/MAOA	2
hsa04974	Protein digestion and absorption	5/210	103/8644	0.1054362	0.3444949	0.2652043	CPA3/COL6A6/DPP4/COL8A1/COL14A1	5
hsa04623	Cytosolic DNA-sensing pathway	4/210	75/8644	0.1090478	0.3444949	0.2652043	CCL5/IL1B/IL6/CXCL10	4

hsa05130	Pathogenic Escherichia coli infection	8/210	198/8644	0.1092548	0.3444949	0.2652043	NAIP/JUN/IL1B/MYH10/FOS/TNFSF10/MYH11/IL6	8
hsa04630	JAK-STAT signaling pathway	7/210	166/8644	0.1095138	0.3444949	0.2652043	STAT4/IL2RG/IL2RB/IL6/AOX1/SOCS3/CSF3	7
hsa00280	Valine, leucine and isoleucine degradation	3/210	48/8644	0.1105469	0.3444949	0.2652043	HMGCLL1/BCAT1/AOX1	3
hsa04928	Parathyroid hormone synthesis, secretion and action	5/210	106/8644	0.1153855	0.3544316	0.2728538	PLCB4/EGR1/JUND/FOS/NR4A2	5
hsa05322	Systemic lupus erythematosus	6/210	137/8644	0.1163805	0.3544316	0.2728538	HLA-DPA1/HLA-DOA/HLA-DOB/C1R/HLA-DQA1/HLA-DQB1	6
hsa00790	Folate biosynthesis	2/210	27/8644	0.1389728	0.4184798	0.3221604	ALPL/AKR1C3	2

hsa01200	Carbon metabolism	5/210	115/8644	0.1477569	0.4399872	0.3387176	PSAT1/SHMT2/ENO1/IDH1/PHGDH	5
hsa05132	Salmonella infection	9/210	249/8644	0.1523121	0.4485674	0.3453229	NAIP/PIK3CG/JUN/NLRC4/IL1B/RHOH/FOS/TNFSF10/IL6	9
hsa04610	Complement and coagulation cascades	4/210	86/8644	0.1563263	0.4553854	0.3505716	SERPINF2/VSIG4/C1R/FGA	4
hsa04978	Mineral absorption	3/210	60/8644	0.1784336	0.5141957	0.3958459	HMOX1/MT1X/MT1M	3
hsa04611	Platelet activation	5/210	124/8644	0.1835248	0.5232411	0.4028093	P2RY1/PLCB4/PIK3CG/RASGRP1/FGA	5
hsa01210	2-Oxocarboxylic acid metabolism	2/210	33/8644	0.1907633	0.5381532	0.4142892	IDH1/BCAT1	2

hsa04010	MAPK signaling pathway	10/210	301/8644	0.1968818	0.5496284	0.4231232	RPS6KA2/HGF/TGFB2/JUN/JUND/IL1B/RASGRP1/FOS/NR4A1/NTRK2	10
hsa04510	Focal adhesion	7/210	203/8644	0.2240922	0.6132403	0.4720938	HGF/ITGA10/JUN/ITGA4/TNR/COL6A6/TNC	7
hsa05225	Hepatocellular carcinoma	6/210	168/8644	0.2244087	0.6132403	0.4720938	GSTM5/HGF/TGFB2/HMOX1/NQO1/GSTT1	6
hsa04960	Aldosterone-regulated sodium reabsorption	2/210	37/8644	0.2265328	0.6132403	0.4720938	SCNN1G/SFN	2
hsa04530	Tight junction	6/210	170/8644	0.2323135	0.6166267	0.4747009	CD1C/JUN/CD1E/MYH10/MYH11/CFTR	6
hsa05211	Renal cell carcinoma	3/210	69/8644	0.2350953	0.6166267	0.4747009	HGF/TGFB2/JUN	3

hsa 00 13 0	Ubiquinone and other terpenoid-quinone biosynthesis	1/2 10	11/86 44	0.2 371 514	0.616 6267	0.4 747 009	NQO1	1
hsa 04 91 5	Estrogen signaling pathway	5/2 10	137/8 644	0.2 400 166	0.616 6267	0.4 747 009	PLCB4/JUN/KRT19/FOS/FKBP5	5
hsa 05 41 4	Dilated cardiomyopathy	4/2 10	103/8 644	0.2 409 093	0.616 6267	0.4 747 009	RYR2/TGFB2/ITGA10/ITGA4	4
hsa 04 91 7	Prolactin signaling pathway	3/2 10	70/86 44	0.2 415 888	0.616 6267	0.4 747 009	FOS/IRF1/SOCS3	3
hsa 04 37 1	Apelin signaling pathway	5/2 10	139/8 644	0.2 491 114	0.629 8288	0.4 848 643	PLCB4/RYR2/EGR1/PIK3CG/APLNR	5
hsa 05 17 0	Human immunodeficiency virus 1 infection	7/2 10	212/8 644	0.2 568 713	0.637 4215	0.4 907 094	NFATC2/JUN/WEE1/FOS/CD3G/CD3E/CD3D	7

hsa05207	Chemical carcinogenesis	7/210	212/8644	0.2568713	0.6374215	0.4907094	RPS6KA2/EIF4EBP1/GSTM5/EPHX2/JUN/FOS/GSTT1	7
hsa04936	Alcoholic liver disease	5/210	142/8644	0.2629158	0.646082	0.4973766	FASN/IL1B/IL6/CXCL1/CXCL2	5
hsa04350	TGF-beta signaling pathway	4/210	108/8644	0.2675937	0.646082	0.4973766	BMP5/TGFB2/DCN/INHBA	4
hsa04931	Insulin resistance	4/210	108/8644	0.2675937	0.646082	0.4973766	RPS6KA2/TRIB3/IL6/SOCS3	4
hsa04066	HIF-1 signaling pathway	4/210	109/8644	0.2729966	0.6475824	0.4985317	ENO1/EIF4EBP1/HMOX1/IL6	4
hsa04020	Calcium signaling pathway	8/210	253/8644	0.2730478	0.6475824	0.4985317	PLCB4/RYR2/NFATC2/HGF/HRH2/CYSLTR1/CASQ2/NTRK2	8

hsa00860	Porphyrin metabolism	2/210	43/8644	0.2808671	0.6550448	0.5042764	HMOX1/CP	2
hsa04971	Gastric acid secretion	3/210	76/8644	0.2810826	0.6550448	0.5042764	PLCB4/HRH2/CFTR	3
hsa00980	Metabolism of xenobiotics by cytochrome P450	3/210	78/8644	0.2943906	0.6743307	0.5191234	GSTM5/HPGDS/GSTT1	3
hsa04721	Synaptic vesicle cycle	3/210	78/8644	0.2943906	0.6743307	0.5191234	SLC18A2/SLC1A3/SLC6A4	3
hsa01521	EGFR tyrosine kinase inhibitor resistance	3/210	79/8644	0.3010609	0.6837654	0.5263866	EIF4EBP1/HGF/IL6	3
hsa04670	Leukocyte transendothelial migration	4/210	115/8644	0.3057626	0.6837868	0.526403	CXCL12/ITGA4/VCAM1/RHOH	4

hsa00604	Glycosphingolipid biosynthesis	1/210	15/8644	0.3087246	0.6837868	0.526403	ST6GALNAC3	1
hsa00730	Thiamine metabolism	1/210	15/8644	0.3087246	0.6837868	0.526403	ALPL	1
hsa04973	Carbohydrate digestion and absorption	2/210	47/8644	0.3169965	0.696353	0.5360769	PLCB4/MGAM	2
hsa04932	Non-alcoholic fatty liver disease	5/210	155/8644	0.3244408	0.6982053	0.5375029	JUN/IL1B/FOS/IL6/SOCS3	5
hsa00360	Phenylalanine metabolism	1/210	16/8644	0.3255479	0.6982053	0.5375029	MAOA	1
hsa04146	Peroxisome	3/210	83/8644	0.327799	0.6982053	0.5375029	IDH1/HMGCLL1/EPHX2	3

hsa05202	Transcriptional misregulation in cancer	6/210	193/8644	0.3282607	0.6982053	0.5375029	NFKBIZ/NR4A3/IL2RB/IL1R2/ETV5/IL6	6
hsa04935	Growth hormone synthesis, secretion and action	4/210	120/8644	0.3333805	0.7003327	0.5391406	PLCB4/JUNB/FOS/SOCS3	4
hsa05412	Arrhythmogenic right ventricular cardiomyopathy	3/210	84/8644	0.3344872	0.7003327	0.5391406	RYR2/ITGA10/ITGA4	3
hsa00120	Primary bile acid biosynthesis	1/210	17/8644	0.3419636	0.7009392	0.5396075	CH25H	1
hsa00450	Selenocompound metabolism	1/210	17/8644	0.3419636	0.7009392	0.5396075	CTH	1
hsa00330	Arginine and proline metabolism	2/210	50/8644	0.3438284	0.7009392	0.5396075	PYCR1/MAOA	2

hsa04742	Taste transduction	3/210	86/8644	0.3478541	0.7009392	0.5396075	P2RY1/PLCB4/SCNN1G	3
hsa05210	Colorectal cancer	3/210	86/8644	0.3478541	0.7009392	0.5396075	TGFB2/JUN/FOS	3
hsa04913	Ovarian steroidogenesis	2/210	51/8644	0.3527008	0.7054016	0.5430429	PTGS2/AKR1C3	2
hsa00061	Fatty acid biosynthesis	1/210	18/8644	0.3579817	0.7106599	0.5470909	FASN	1
hsa04022	cGMP-PKG signaling pathway	5/210	167/8644	0.3825128	0.7537753	0.5802826	PLCB4/TRPC6/GTF2IRD1/NFATC2/PIK3CG	5
hsa00100	Steroid biosynthesis	1/210	20/8644	0.3888628	0.7606951	0.5856098	DHCR7	1

hsa04912	GnRH signaling pathway	3/210	93/8644	0.3943445	0.7658285	0.5895616	PLCB4/EGR1/JUN	3
hsa04728	Dopaminergic synapse	4/210	132/8644	0.3998773	0.7674007	0.5907719	PLCB4/SLC18A2/FOS/MAOA	4
hsa04015	Rap1 signaling pathway	6/210	210/8644	0.4022706	0.7674007	0.5907719	P2RY1/PLCB4/HGF/CNR1/SKAP1/FPR1	6
hsa00770	Pantothenate and CoA biosynthesis	1/210	21/8644	0.4037444	0.7674007	0.5907719	BCAT1	1
hsa04270	Vascular smooth muscle contraction	4/210	134/8644	0.4108918	0.7754859	0.5969963	PLCB4/MYH10/MYH11/PLA2G2A	4
hsa04713	Circadian entrainment	3/210	97/8644	0.4205462	0.7785494	0.5993546	PLCB4/RYR2/FOS	3

hsa04210	Apoptosis	4/210	136/8644	0.4218654	0.7785494	0.5993546	CTSF/JUN/FOS/TNFSF10	4
hsa04370	VEGF signaling pathway	2/210	59/8644	0.4219341	0.7785494	0.5993546	NFATC2/PTGS2	2
hsa04925	Aldosterone synthesis and secretion	3/210	98/8644	0.4270401	0.7785494	0.5993546	PLCB4/NR4A1/NR4A2	3
hsa05231	Choline metabolism in cancer	3/210	98/8644	0.4270401	0.7785494	0.5993546	EIF4EBP1/JUN/FOS	3
hsa03060	Protein export	1/210	23/8644	0.4324342	0.7830565	0.6028243	SRPRB	1
hsa00140	Steroid hormone biosynthesis	2/210	62/8644	0.4469282	0.8038708	0.6188479	AKR1C2/AKR1C3	2

hsa04929	GnRH secretion	2/210	64/8644	0.4632534	0.8276794	0.6371766	PLCB4/TRPC1	2
hsa04927	Cortisol synthesis and secretion	2/210	65/8644	0.4713 1	0.8283702	0.6377084	PLCB4/NR4A1	2
hsa00563	Glycosylphosphatidylinositol (GPI)-anchor biosynthesis	1/210	26/8644	0.4729129	0.8283702	0.6377084	PIGW	1
hsa00592	alpha-Linolenic acid metabolism	1/210	26/8644	0.4729129	0.8283702	0.6377084	PLA2G2A	1
hsa00650	Butanoate metabolism	1/210	27/8644	0.4857567	0.8369922	0.644346	HMGCLL1	1
hsa04720	Long-term potentiation	2/210	67/8644	0.4872044	0.8369922	0.644346	PLCB4/RPS6KA2	2

hsa05221	Acute myeloid leukemia	2/210	67/8644	0.4872044	0.8369922	0.644346	EIF4EBP1/PIM2	2
hsa00830	Retinol metabolism	2/210	68/8644	0.4950399	0.8398834	0.6465717	SDR16C5/AOX1	2
hsa04613	Neutrophil extracellular trap formation	5/210	191/8644	0.497055	0.8398834	0.6465717	PLCB4/FPR1/AQP9/FPR2/FGA	5
hsa00601	Glycosphingolipid biosynthesis	1/210	28/8644	0.498289	0.8398834	0.6465717	FUT3	1
hsa04924	Renin secretion	2/210	69/8644	0.5027995	0.8421891	0.6483467	PLCB4/ACE	2
hsa04725	Cholinergic synapse	3/210	113/8644	0.5207785	0.8503587	0.654636	PLCB4/PIK3CG/FOS	3

hsa00020	Citrate cycle (TCA cycle)	1/210	30/8644	0.522449	0.8503587	0.654636	IDH1	1
hsa00591	Linoleic acid metabolism	1/210	30/8644	0.522449	0.8503587	0.654636	PLA2G2A	1
hsa00630	Glyoxylate and dicarboxylate metabolism	1/210	30/8644	0.522449	0.8503587	0.654636	SHMT2	1
hsa04218	Cellular senescence	4/210	156/8644	0.5278904	0.8503587	0.654636	EIF4EBP1/NFATC2/TGFB2/IL6	4
hsa04724	Glutamatergic synapse	3/210	115/8644	0.5326573	0.8503587	0.654636	PLCB4/TRPC1/SLC1A3	3
hsa00562	Inositol phosphate metabolism	2/210	73/8644	0.5330607	0.8503587	0.654636	PLCB4/PIK3CG	2

hsa01524	Platinum drug resistance	2/210	73/8644	0.5330607	0.8503587	0.654636	GSTM5/GSTT1	2
hsa04080	Neuroactive ligand-receptor interaction	9/210	367/8644	0.5373409	0.8521146	0.6559877	P2RY1/P2RY14/APLNR/CNR1/HRH2/CYSLTR1/FPR1/P2RY10/FPR2	9
hsa00052	Galactose metabolism	1/210	32/8644	0.5454509	0.857943	0.6604746	MGAM	1
hsa04918	Thyroid hormone synthesis	2/210	75/8644	0.5477152	0.857943	0.6604746	PLCB4/DUOX1	2
hsa04722	Neurotrophin signaling pathway	3/210	119/8644	0.5559129	0.857943	0.6604746	RPS6KA2/JUN/NTRK2	3
hsa05165	Human papillomavirus infection	8/210	331/8644	0.5573851	0.857943	0.6604746	EIF4EBP1/PTGS2/ITGA10/ITGA4/TNR/COL6A6/TNC/IRF1	8

hsa05131	Shigellosis	6/210	247/8644	0.5587161	0.857943	0.6604746	PLCB4/NAIP/JUN/CCL5/NLRC4/IL1B	6
hsa05205	Proteoglycans in cancer	5/210	205/8644	0.560224	0.857943	0.6604746	HGF/ANK2/TGFB2/DCN/LUM	5
hsa04152	AMPK signaling pathway	3/210	121/8644	0.5672806	0.8590382	0.6613177	EIF4EBP1/FASN/CFTR	3
hsa04710	Circadian rhythm	1/210	34/8644	0.5673499	0.8590382	0.6613177	NR1D2	1
hsa03018	RNA degradation	2/210	79/8644	0.576052	0.8673142	0.6676889	BTG2/ENO1	2
hsa00983	Drug metabolism	2/210	80/8644	0.5829318	0.8709238	0.6704677	GSTM5/GSTT1	2

hsa00500	Starch and sucrose metabolism	1/210	36/8644	0.5881986	0.8709238	0.6704677	MGAM	1
hsa00512	Mucin type O-glycan biosynthesis	1/210	36/8644	0.5881986	0.8709238	0.6704677	ST6GALNAC3	1
hsa00760	Nicotinate and nicotinamide metabolism	1/210	37/8644	0.5982448	0.8809319	0.6781723	AOX1	1
hsa04926	Relaxin signaling pathway	3/210	129/8644	0.610934	0.8869243	0.6827854	PLCB4/JUN/FOS	3
hsa04310	Wnt signaling pathway	4/210	174/8644	0.6146655	0.8869243	0.6827854	PLCB4/FRZB/NFATC2/JUN	4
hsa04012	ErbB signaling pathway	2/210	85/8644	0.616098	0.8869243	0.6827854	EIF4EBP1/JUN	2

hsa04068	FoxO signaling pathway	3/210	131/8644	0.6213796	0.8869243	0.6827854	TGFB2/TNFSF10/IL6	3
hsa04911	Insulin secretion	2/210	86/8644	0.6224848	0.8869243	0.6827854	PLCB4/RYR2	2
hsa04142	Lysosome	3/210	132/8644	0.6265308	0.8869243	0.6827854	CTSF/CTSE/SLC11A1	3
hsa04650	Natural killer cell mediated cytotoxicity	3/210	132/8644	0.6265308	0.8869243	0.6827854	NFATC2/CD48/TNFSF10	3
hsa04260	Cardiac muscle contraction	2/210	87/8644	0.6287896	0.8869243	0.6827854	RYR2/CASQ2	2
hsa04976	Bile secretion	2/210	89/8644	0.6411544	0.897603	0.6910062	AQP9/CFTR	2

hsa04024	cAMP signaling pathway	5/210	225/8644	0.6430589	0.897603	0.6910062	RYR2/FXYD1/JUN/FOS/CFTR	5
hsa04910	Insulin signaling pathway	3/210	137/8644	0.6515649	0.9029127	0.6950938	EIF4EBP1/FASN/SOCS3	3
hsa04975	Fat digestion and absorption	1/210	43/8644	0.653601	0.9029127	0.6950938	PLA2G2A	1
hsa04810	Regulation of actin cytoskeleton	5/210	229/8644	0.658421	0.9049068	0.696629	CXCL12/ITGA10/ITGA4/MYH10/MYH11	5
hsa04970	Salivary secretion	2/210	93/8644	0.6649117	0.909165	0.6999071	PLCB4/LYZ	2
hsa03022	Basal transcription factors	1/210	45/8644	0.6703106	0.9118947	0.7020085	GTF2IRD1	1

hsa05034	Alcoholism	4/210	188/8644	0.6749427	0.9130782	0.7029196	SLC18A2/FOSB/MAOA/NTRK2	4
hsa04930	Type II diabetes mellitus	1/210	46/8644	0.6783621	0.9130782	0.7029196	SOCS3	1
hsa00514	Other types of O-glycan biosynthesis	1/210	47/8644	0.6862179	0.9130782	0.7029196	GXYLT2	1
hsa05215	Prostate cancer	2/210	97/8644	0.6873924	0.9130782	0.7029196	IL1R2/ETV5	2
hsa01522	Endocrine resistance	2/210	98/8644	0.6928164	0.9130782	0.7029196	JUN/FOS	2
hsa04750	Inflammatory mediator regulation of TRP channels	2/210	98/8644	0.6928164	0.9130782	0.7029196	PLCB4/IL1B	2

hsa00520	Amino sugar and nucleotide sugar metabolism	1/210	49/8644	0.7013611	0.9130782	0.7029196	CHIT1	1
hsa04072	Phospholipase D signaling pathway	3/210	148/8644	0.7023727	0.9130782	0.7029196	PLCB4/PIK3CG/MS4A2	3
hsa04723	Retrograde endocannabinoid signaling	3/210	148/8644	0.7023727	0.9130782	0.7029196	PLCB4/PTGS2/CNR1	3
hsa00565	Ether lipid metabolism	1/210	50/8644	0.7086577	0.9130782	0.7029196	PLA2G2A	1
hsa05110	Vibrio cholerae infection	1/210	50/8644	0.7086577	0.9130782	0.7029196	CFTR	1
hsa04979	Cholesterol metabolism	1/210	51/8644	0.7157769	0.9178383	0.7065841	LIPG	1

hsa04261	Adrenergic signaling in cardiomyocytes	3/210	154/8644	0.7276263	0.9253304	0.7123518	PLCB4/RYR2/PIK3CG	3
hsa04961	Endocrine and other factor-regulated calcium reabsorption	1/210	53/8644	0.7294999	0.9253304	0.7123518	PLCB4	1
hsa05415	Diabetic cardiomyopathy	4/210	203/8644	0.7319778	0.9253304	0.7123518	PLCB4/RYR2/TGFB2/ACE	4
hsa04150	mTOR signaling pathway	3/210	156/8644	0.7356647	0.925625	0.7125786	RPS6KA2/EIF4EBP1/DDIT4	3
hsa04110	Cell cycle	3/210	157/8644	0.7396135	0.9262449	0.7130558	TGFB2/WEE1/SFN	3
hsa04217	Necroptosis	3/210	159/8644	0.7473716	0.9316074	0.717184	STAT4/IL1B/TNFSF10	3

hsa01212	Fatty acid metabolism	1/210	57/8644	0.7549987	0.9367577	0.7211489	FASN	1
hsa04923	Regulation of lipolysis in adipocytes	1/210	58/8644	0.7609904	0.9398406	0.7235223	PTGS2	1
hsa05206	MicroRNAs in cancer	6/210	310/8644	0.7703061	0.945391	0.7277951	PTGS2/TGFB2/TNR/HMOX1/DDIT4/TNC	6
hsa04730	Long-term depression	1/210	60/8644	0.7725396	0.945391	0.7277951	PLCB4	1
hsa00561	Glycerolipid metabolism	1/210	63/8644	0.7888303	0.9609387	0.7397643	LIPG	1
hsa05020	Prion disease	5/210	272/8644	0.7960291	0.9626831	0.7411072	RYR2/EGR1/CCL5/IL1B/IL6	5

hsa04071	Sphingolipid signaling pathway	2/210	121/8644	0.7974464	0.9626831	0.7411072	PLCB4/MS4A2	2
hsa00010	Glycolysis / Gluconeogenesis	1/210	67/8644	0.8087587	0.9641697	0.7422516	ENO1	1
hsa04664	Fc epsilon RI signaling pathway	1/210	68/8644	0.813441	0.9641697	0.7422516	MS4A2	1
hsa04920	Adipocytokine signaling pathway	1/210	69/8644	0.8180093	0.9641697	0.7422516	SOCS3	1
hsa00230	Purine metabolism	2/210	128/8644	0.8223853	0.9641697	0.7422516	AMPD1/PDE7B	2
hsa05230	Central carbon metabolism in cancer	1/210	70/8644	0.8224662	0.9641697	0.7422516	IDH1	1

hsa04622	RIG-I-like receptor signaling pathway	1/210	71/8644	0.8268145	0.9641697	0.7422516	CXCL10	1
hsa04014	Ras signaling pathway	4/210	236/8644	0.8305874	0.9641697	0.7422516	HGF/RASGRP1/NTRK2/PLA2G2A	4
hsa05218	Melanoma	1/210	72/8644	0.8310567	0.9641697	0.7422516	HGF	1
hsa05223	Non-small cell lung cancer	1/210	72/8644	0.8310567	0.9641697	0.7422516	HGF	1
hsa04115	p53 signaling pathway	1/210	74/8644	0.8392335	0.965959	0.7436291	SFN	1
hsa03320	PPAR signaling pathway	1/210	75/8644	0.8431729	0.965959	0.7436291	PLIN2	1

hsa05212	Pancreatic cancer	1/210	76/8644	0.8470163	0.965959	0.7436291	TGFB2	1
hsa05220	Chronic myeloid leukemia	1/210	76/8644	0.8470163	0.965959	0.7436291	TGFB2	1
hsa04814	Motor proteins	3/210	193/8644	0.8529244	0.9685752	0.7456431	MYH10/MYH11/DNAH5	3
hsa04120	Ubiquitin mediated proteolysis	2/210	142/8644	0.8641772	0.9726062	0.7487463	RHOBTB2/SOCS3	2
hsa05224	Breast cancer	2/210	147/8644	0.876786	0.9726062	0.7487463	JUN/FOS	2
hsa01232	Nucleotide metabolism	1/210	85/8644	0.8776496	0.9726062	0.7487463	AMPD1	1

hsa05203	Viral carcinogenesis	3/210	204/8644	0.8777204	0.9726062	0.7487463	JUN/EGR2/EGR3	3
hsa05226	Gastric cancer	2/210	149/8644	0.8815218	0.9726062	0.7487463	HGF/TGFB2	2
hsa04540	Gap junction	1/210	88/8644	0.8864372	0.9726062	0.7487463	PLCB4	1
hsa04211	Longevity regulating pathway	1/210	89/8644	0.8892245	0.9726062	0.7487463	EIF4EBP1	1
hsa05032	Morphine addiction	1/210	91/8644	0.8945965	0.9726062	0.7487463	PDE7B	1
hsa04934	Cushing syndrome	2/210	155/8644	0.8947418	0.9726062	0.7487463	PLCB4/NR4A1	2

hsa05222	Small cell lung cancer	1/210	92/8644	0.8971845	0.9726062	0.7487463	PTGS2	1
hsa04390	Hippo signaling pathway	2/210	157/8644	0.8988361	0.9726062	0.7487463	BMP5/TGFB2	2
hsa05160	Hepatitis C	2/210	158/8644	0.9008274	0.9726062	0.7487463	SOCS3/CXCL10	2
hsa04070	Phosphatidylinositol signaling system	1/210	97/8644	0.9092057	0.9726062	0.7487463	PLCB4	1
hsa04666	Fc gamma R-mediated phagocytosis	1/210	97/8644	0.9092057	0.9726062	0.7487463	FCGR2B	1
hsa00564	Glycerophospholipid metabolism	1/210	99/8644	0.9136128	0.9726062	0.7487463	PLA2G2A	1

hsa04916	Melanogenesis	1/210	101/8644	0.9178069	0.9726062	0.7487463	PLCB4	1
hsa04914	Progesterone-mediated oocyte maturation	1/210	102/8644	0.9198274	0.9726062	0.7487463	RPS6KA2	1
hsa04137	Mitophagy	1/210	103/8644	0.9217984	0.9726062	0.7487463	JUN	1
hsa04922	Glucagon signaling pathway	1/210	107/8644	0.9292117	0.9765832	0.751808	PLCB4	1
hsa04919	Thyroid hormone signaling pathway	1/210	121/8644	0.9500643	0.9892348	0.7615476	PLCB4	1
hsa05012	Parkinson disease	3/210	266/8644	0.9596942	0.9892348	0.7615476	SLC18A2/SLC39A14/MAOA	3

hsa05010	Alzheimer disease	5/210	384/8644	0.9602127	0.9892348	0.7615476	PLCB4/PTGS2/SLC39A14/IL1B/IL6	5
hsa04114	Oocyte meiosis	1/210	131/8644	0.961095	0.9892348	0.7615476	RPS6KA2	1
hsa04550	Signaling pathways regulating pluripotency of stem cells	1/210	143/8644	0.9711763	0.9892348	0.7615476	INHBA	1
hsa05017	Spinocerebellar ataxia	1/210	143/8644	0.9711763	0.9892348	0.7615476	PLCB4	1
hsa05022	Pathways of neurodegeneration	6/210	476/8644	0.9774683	0.9892348	0.7615476	PLCB4/RYR2/PTGS2/IL1B/IL6/DNAH5	6
hsa04714	Thermogenesis	2/210	232/8644	0.9786624	0.9892348	0.7615476	RPS6KA2/CNR1	2

hsa05016	Huntington disease	3/210	306/8644	0.9812638	0.9892348	0.7615476	PLCB4/SLC1A3/DNAH5	3
hsa04140	Autophagy	1/210	165/8644	0.9833865	0.9892348	0.7615476	DDIT4	1
hsa04141	Protein processing in endoplasmic reticulum	1/210	170/8644	0.9853448	0.9892348	0.7615476	HYOU1	1
hsa04144	Endocytosis	2/210	250/8644	0.9855436	0.9892348	0.7615476	IL2RG/IL2RB	2
hsa05014	Amyotrophic lateral sclerosis	1/210	364/8644	0.9998936	0.9998936	0.7697531	DNAH5	1

DEG – differentially expressed gene, KEGG – Kyoto Encyclopedia of Genes and Genomes.

Table S7. Characteristics and quality assessment of instrumental variables used in the MR analysis.
Panel A. Characteristics and directionality assessment of candidate instrumental variables.

Exposure	Sample size Exposure	Outcome	Sample size Outcome	SNP	Effect allele	Other allele	Effect allele frequency	Beta Exposure	SE Exposure	P-value Exposure	F statistic	Beta Outcome	SE Outcome	Steiger R^2 for Exposure	Steiger R^2 for Outcome	Steiger direction	Steiger P-value	mr_keep	Final outlier	Detection level	Outliers Radial MR IVW	Outliers Radial MR Egger	Outliers PRESSO	Outliers LOO
UACA-mediated FEV1/FVC	475645	Chronic Obstructive Pulmonary Disease	394244	rs11072162	C	G	0.494	-0.009	0.002	1.02E-05	19.475	0.016	0.01	4.15E-05	1.85E-05	TRUE	0.32	FALSE						
UACA-mediated FEV1/FVC	475645	Chronic Obstructive Pulmonary	394244	rs117715	A	G	0.985	0.04	0.008	2.50E-06	22.165	-0.056	0.068	4.74E-05	5.71E-06	TRUE	0.037	TRUE						

		Disease		3971													
UACA-mediated FEV1/FVC	475645	Chronic Obstructive Pulmonary Disease	394244	rs149926387	A	G	0.983	0.039	0.008	1.02E-06	23.883	-0.019	0.033	5.11E-05	2.70E-06	TRUE	0.011	TRUE
UACA-mediated FEV1/FVC	475645	Chronic Obstructive Pulmonary Disease	394244	rs28719624	A	G	0.884	0.013	0.003	5.84E-05	16.152	-0.043	0.017	3.47E-05	5.23E-05	FALSE	0.533	TRUE
UACA-medi	475645	Chronic Obstru	394244	rs35	A	G	0.84	-0.	0.003	7.22E-19	78.6	0.027	0.0	1.68E-04	3.01E-05	TRUE	5.24	TR

ated FEV1/FVC		ctive Pulmonary Disease		683356				025			95		14				E-04	UE
UACA-mediated FEV1/FVC	475645	Chronic Obstructive Pulmonary Disease	394244	rs4776542	T	C	0.586	-0.018	0.002	3.23E-17	71.2	0.006	0.01	1.52E-04	2.77E-06	TRUE	7.53E-07	TRUE
UACA-mediated FEV1/FVC	475645	Chronic Obstructive Pulmonary Disease	394244	rs4777345	C	G	0.613	0.015	0.002	2.51E-12	49.042	-6.85E-04	0.011	1.05E-04	3.42E-08	TRUE	3.13E-06	TRUE
UACA-mediated	475645	Chronic Obstructive	394244	rs567	A	G	0.089	0.014	0.004	9.68E-05	15.1	-0.025	0.017	3.24E-05	1.83E-05	TRUE	0.51	TRUE

FEV1/FVC		Pulmonary Disease		39943							94							
UACA-mediated FEV1/FVC	475645	Chronic Obstructive Pulmonary Disease	394244	rs62015473	A	G	0.246	-0.028	0.002	7.90E-31	133.264	0.031	0.012	2.84E-04	5.35E-05	TRUE	9.45E-06	TRUE
UACA-mediated FEV1/FVC	475645	Chronic Obstructive Pulmonary Disease	394244	rs62016788	T	G	0.966	0.028	0.006	1.38E-06	23.31	-0.08	0.032	4.98E-05	5.22E-05	FALSE	0.936	TRUE
UACA-medi	475645	Chronic Obstru	394244	rs62	C	G	0.091	-0.	0.004	1.35E-05	18.9	0.046	0.0	4.04E-05	3.32E-05	TRUE	0.781	TR

ated FEV1/FVC		ctive Pulmonary Disease		0177677				016			31		23					UE
UACA-mediated FEV1/FVC	475645	Chronic Obstructive Pulmonary Disease	394244	rs62017772	T	C	0.981	0.053	0.008	1.87E-12	49.618	-0.1	0.038	1.06E-04	5.45E-05	TRUE	0.176	TRUE
UACA-mediated FEV1/FVC	475645	Chronic Obstructive Pulmonary Disease	394244	rs62019106	A	G	0.016	-0.046	0.008	1.68E-08	31.843	0.111	0.044	6.79E-05	5.17E-05	TRUE	0.624	TRUE

UACA-mediated FEV1/FVC	475645	Chronic Obstructive Pulmonary Disease	394244	rs71813395	C	G	0.829	0.014	0.003	5.89E-07	24.95	-0.014	0.013	5.32E-05	9.37E-06	TRUE	0.049	TRUE
UACA-mediated FEV1/FVC	475645	Chronic Obstructive Pulmonary Disease	394244	rs72755465	C	G	0.93	0.017	0.004	3.98E-05	16.884	-0.047	0.02	3.61E-05	4.61E-05	FALSE	0.715	TRUE
UACA-mediated FEV1/FVC	475645	Chronic Obstructive Pulmonary Disease	394244	rs72759647	C	G	0.06	0.016	0.004	1.73E-04	14.1	-0.037	0.023	3.01E-05	2.13E-05	TRUE	0.686	TRUE

UACA-mediated FEV1/FVC	475645	Chronic Obstructive Pulmonary Disease	394244	rs79309344	A	C	0.037	-0.021	0.005	1.05E-04	15.047	0.057	0.029	3.22E-05	3.19E-05	TRUE	0.991	TRUE
UACA-mediated FEV1/FVC	475645	Chronic Obstructive Pulmonary Disease	394244	rs79745150	T	C	0.971	0.054	0.006	4.79E-18	74.961	-0.036	0.025	1.61E-04	1.67E-05	TRUE	6.69E-05	TRUE
UACA-mediated FEV1/FVC	475645	Chronic Obstructive Pulmonary Disease	394244	rs8026415	A	G	0.942	0.017	0.004	1.79E-04	14.033	-0.025	0.024	2.99E-05	8.83E-06	TRUE	0.246	TRUE

UACA-mediated FEV1/FVC	475645	Chronic Obstructive Pulmonary Disease	394244	rs8041949	T	G	0.505	0.014	0.002	4.51E-11	43.375	-0.017	0.01	9.26E-05	2.19E-05	TRUE	0.022	TRUE	
UACA-mediated FEV1/FVC	475645	Chronic Obstructive Pulmonary Disease	394244	rs71755526	T	C	0.053	-0.018	0.005	7.80E-05	15.602	0.086	0.032	3.33E-05	5.73E-05	FALSE	0.403	TRUE	Yes
IRF1-mediated FEV1/FVC	475645	Chronic Obstructive Pulmonary Disease	394244	rs10455052	A	C	0.136	-0.014	0.003	4.96E-06	20.857	0.042	0.011	4.45E-05	1.22E-04	FALSE	0.043	TRUE	

IRF1-mediated FEV1/FVC	475645	Chronic Obstructive Pulmonary Disease	394244	rs11568506	A	G	0.023	-0.03	0.007	1.63E-05	18.585	0.053	0.034	3.97E-05	2.01E-05	TRUE	0.398	TRUE
IRF1-mediated FEV1/FVC	475645	Chronic Obstructive Pulmonary Disease	394244	rs116332641	C	G	0.165	0.011	0.003	9.14E-05	15.304	-0.019	0.017	3.26E-05	1.00E-05	TRUE	0.237	TRUE
IRF1-mediated FEV1/FVC	475645	Chronic Obstructive Pulmonary	394244	rs117399	T	C	0.337	0.009	0.002	4.87E-05	16.5	-0.033	0.012	3.52E-05	5.70E-05	FALSE	0.452	TRUE

		Disease		35														
IRF1-mediated FEV1/FVC	475645	Chronic Obstructive Pulmonary Disease	394244	rs11746729	A	G	0.455	0.008	0.002	9.52E-05	15.233	-0.034	0.012	3.25E-05	6.99E-05	FALSE	0.217	TRUE
IRF1-mediated FEV1/FVC	475645	Chronic Obstructive Pulmonary Disease	394244	rs12153431	T	C	0.894	0.015	0.003	4.01E-06	21.261	-0.037	0.014	4.54E-05	5.78E-05	FALSE	0.687	TRUE
IRF1-mediated FEV1/FVC	475645	Chronic Obstructive Pulmonary	394244	rs12956	A	G	0.1	-0.015	0.003	1.87E-05	18.318	0.004	0.012	3.91E-05	8.95E-07	TRUE	0.014	TRUE

		Disease		83												
IRF1-mediated FEV1/FVC	475645	Chronic Obstructive Pulmonary Disease	394244	rs162901	A	G	0.893	0.02	0.003	2.84E-09	35.284	-0.01	0.016	7.52E-05	2.89E-06	TRUE	0.001	TRUE
IRF1-mediated FEV1/FVC	475645	Chronic Obstructive Pulmonary Disease	394244	rs2070729	A	C	0.442	-0.015	0.002	2.11E-13	53.905	0.041	0.01	1.15E-04	1.27E-04	FALSE	0.803	TRUE
IRF1-mediated FEV1/FVC	475645	Chronic Obstructive Pulmonary Disease	394244	rs2073644	A	T	0.544	0.019	0.002	9.19E-21	87.329	0.047	0.01	1.86E-04	1.65E-04	TRUE	0.713	FALSE

IRF1-mediated FEV1/FVC	475645	Chronic Obstructive Pulmonary Disease	394244	rs2539442	T	C	0.061	-0.016	0.004	1.81E-04	14.018	0.004	0.015	2.99E-05	6.30E-07	TRUE	0.03	TRUE
IRF1-mediated FEV1/FVC	475645	Chronic Obstructive Pulmonary Disease	394244	rs2558887	A	C	0.577	0.012	0.002	2.88E-08	30.78	-0.003	0.01	6.56E-05	5.31E-07	TRUE	6.20E-04	TRUE
IRF1-mediated FEV1/FVC	475645	Chronic Obstructive Pulmonary Disease	394244	rs266677735	A	G	0.148	-0.018	0.003	1.22E-09	36.93	0.044	0.012	7.87E-05	1.06E-04	FALSE	0.505	TRUE

IRF1-mediated FEV1/FVC	475645	Chronic Obstructive Pulmonary Disease	394244	rs2745558	A	G	0.621	0.008	0.002	1.76E-04	14.078	-0.038	0.01	3.00E-05	1.11E-04	FALSE	0.019	TRUE
IRF1-mediated FEV1/FVC	475645	Chronic Obstructive Pulmonary Disease	394244	rs39140018	T	C	0.987	0.04	0.009	7.81E-06	19.981	-0.024	0.04	4.26E-05	2.84E-06	TRUE	0.024	TRUE
IRF1-mediated FEV1/FVC	475645	Chronic Obstructive Pulmonary Disease	394244	rs5555696853	T	G	0.973	0.028	0.007	1.86E-04	13.973	-0.026	0.036	4.14E-05	4.61E-06	TRUE	0.046	TRUE

IRF1-mediated FEV1/FVC	475645	Chronic Obstructive Pulmonary Disease	394244	rs71583468	A	G	0.082	0.017	0.004	4.41E-06	21.077	-0.013	0.023	4.49E-05	2.82E-06	TRUE	0.02	TRUE
IRF1-mediated FEV1/FVC	475645	Chronic Obstructive Pulmonary Disease	394244	rs72795177	A	G	0.11	0.018	0.003	3.95E-08	30.173	-0.019	0.021	6.44E-05	6.64E-06	TRUE	0.011	TRUE
IRF1-mediated FEV1/FVC	475645	Chronic Obstructive Pulmonary Disease	394244	rs77031552	T	C	0.768	0.029	0.002	4.79E-32	138.839	-0.055	0.011	2.96E-04	1.91E-04	TRUE	0.117	TRUE

IRF1-mediated FEV1/FVC	475645	Chronic Obstructive Pulmonary Disease	394244	rs8091213	T	C	0.735	0.009	0.002	1.02E-04	15.1101	-0.025	0.011	3.22E-05	4.59E-05	FALSE	0.611	TRUE
PIK3CG-mediated FEV1/FVC	475645	Chronic Obstructive Pulmonary Disease	394244	rs2392929	T	G	0.797	0.015	0.003	1.38E-08	32.217	-9.99E-04	0.011	6.87E-05	6.35E-08	TRUE	1.91E-04	TRUE
PIK3CG-mediated FEV1/FVC	475645	Chronic Obstructive Pulmonary Disease	394244	rs3421882	T	C	0.558	0.01	0.002	1.40E-06	23.281	-0.005	0.01	4.96E-05	1.68E-06	TRUE	0.008	TRUE

PIK3CG-mediated FEV1/FVC	475645	Chronic Obstructive Pulmonary Disease	394244	rs560347741	A	G	0.783	-0.01	0.003	9.02E-05	15.335	0.027	0.012	3.27E-05	4.02E-05	FALSE	0.773	TRUE
TNFSF10-mediated FEV1/FVC	475645	Chronic Obstructive Pulmonary Disease	394244	rs4894532	T	G	0.368	-0.012	0.002	1.11E-08	32.638	0.009	0.011	6.96E-05	5.66E-06	TRUE	0.006	TRUE
TNFSF10-mediated FEV1/FVC	475645	Chronic Obstructive Pulmonary Disease	394244	rs62228316	A	G	0.054	0.036	0.005	5.76E-15	60.98	-0.031	0.021	1.30E-04	1.77E-05	TRUE	8.34E-04	TRUE

TNFSF10-mediated FEV1/FVC	475645	Chronic Obstructive Pulmonary Disease	394244	rs9858290	A	C	0.472	0.008	0.002	5.06E-05	16.427	-0.002	0.01	3.50E-05	1.99E-07	TRUE	0.011	TRUE

COPD – chronic obstructive pulmonary disease, FEV1/FVC – forced expiratory volume in one second/forced vital capacity, IV – instrumental variable, IVW – inverse-variance weighted, LOO – leave-one-out, MR – Mendelian randomisation, MR-Egger – Mendelian randomisation-Egger regression, MR-PRESSO – Mendelian Randomization Pleiotropy RESidual Sum and Outlier, R^2 – proportion of variance explained, SE – standard error, SNP – single nucleotide polymorphism, β – beta coefficient.

Panel B. Instrument strength and genetic association characteristics of the selected instrumental variables.

SNP	Chromosome	Position	Effect allele	Other allele	Exposure	Exposure MAF	Exposure FDR	Exposure F statistic	Exposure Beta	Positive Control	Positive Control Beta
rs62019106	15	71286476	A	G	UACA-mediated FEV1_FVC	0.016	1.37E-05	31.843	-0.046	COPD	0.111
rs62015473	15	71555837	A	G	UACA-mediated FEV1_FVC	0.246	6.38E-27	133.264	-0.028	COPD	0.031
rs35683356	15	71539772	A	G	UACA-mediated FEV1_FVC	0.16	2.28E-15	78.695	-0.025	COPD	0.027
rs79309344	15	70937762	A	C	UACA-mediated FEV1_FVC	0.037	0.028	15.047	-0.021	COPD	0.057

rs7175526	15	71415834	T	C	UACA-mediated FEV1_FVC	0.053	0.022	15.603	-0.018	COPD	0.086
rs4776542	15	71543975	T	C	UACA-mediated FEV1_FVC	0.414	8.82E-14	71.2	-0.018	COPD	0.006
rs62017767	15	71499299	C	G	UACA-mediated FEV1_FVC	0.091	0.005	18.931	-0.016	COPD	0.046
rs11072162	15	70666303	C	G	UACA-mediated FEV1_FVC	0.494	0.004	19.475	-0.009	COPD	0.016
rs28719624	15	71114858	A	G	UACA-mediated FEV1_FVC	0.116	0.017	16.152	0.013	COPD	-0.043
rs8041949	15	71529661	T	G	UACA-mediated FEV1_FVC	0.495	5.83E-08	43.375	0.014	COPD	-0.017
rs7181395	15	70669203	C	G	UACA-mediated FEV1_FVC	0.171	3.36E-04	24.95	0.014	COPD	-0.014
rs56739943	15	71049952	A	G	UACA-mediated FEV1_FVC	0.089	0.026	15.194	0.014	COPD	-0.025
rs4777345	15	71522857	C	G	UACA-mediated FEV1_FVC	0.387	3.95E-09	49.042	0.015	COPD	-6.85E-04
rs72759647	15	71540999	C	G	UACA-mediated FEV1_FVC	0.06	0.042	14.1	0.016	COPD	-0.037
rs8026415	15	71487066	A	G	UACA-mediated FEV1_FVC	0.058	0.043	14.033	0.017	COPD	-0.025
rs72755465	15	70922041	C	G	UACA-mediated FEV1_FVC	0.07	0.012	16.884	0.017	COPD	-0.047
rs62016788	15	71432376	T	G	UACA-mediated FEV1_FVC	0.034	7.14E-04	23.31	0.028	COPD	-0.08

rs149926387	15	70767116	A	G	UACA-mediated FEV1_FVC	0.017	5.48E-04	23.883	0.039	COPD	-0.019
rs117153971	15	71531246	A	G	UACA-mediated FEV1_FVC	0.015	0.001	22.165	0.04	COPD	-0.056
rs62017772	15	71529271	T	C	UACA-mediated FEV1_FVC	0.019	3.01E-09	49.618	0.053	COPD	-0.1
rs79745150	15	71543487	T	C	UACA-mediated FEV1_FVC	0.029	1.41E-14	74.961	0.054	COPD	-0.036
rs11568506	5	131671460	A	G	IRF1-mediated FEV1_FVC	0.023	0.006	18.585	-0.03	COPD	0.053
rs2667735	5	131625376	A	G	IRF1-mediated FEV1_FVC	0.148	1.24E-06	36.93	-0.018	COPD	0.044
rs253942	5	131320462	T	C	IRF1-mediated FEV1_FVC	0.061	0.043	14.018	-0.016	COPD	0.004
rs2070729	5	131819921	A	C	IRF1-mediated FEV1_FVC	0.442	3.85E-10	53.905	-0.015	COPD	0.041
rs1295683	5	131998876	A	G	IRF1-mediated FEV1_FVC	0.1	0.007	18.318	-0.015	COPD	0.004
rs10455052	5	132034588	A	C	IRF1-mediated FEV1_FVC	0.136	0.002	20.857	-0.014	COPD	0.042

rs274558	5	131721174	A	G	IRF1-mediated FEV1_FVC	0.379	0.042	14.078	0.008	COPD	-0.038
rs11746729	5	132123536	A	G	IRF1-mediated FEV1_FVC	0.455	0.026	15.233	0.008	COPD	-0.034
rs11739935	5	131380658	T	C	IRF1-mediated FEV1_FVC	0.337	0.015	16.5	0.009	COPD	-0.033
rs809123	5	132138164	T	C	IRF1-mediated FEV1_FVC	0.265	0.027	15.101	0.009	COPD	-0.025
rs116332641	5	132280062	C	G	IRF1-mediated FEV1_FVC	0.165	0.025	15.304	0.011	COPD	-0.019
rs25887	5	131416061	A	C	IRF1-mediated FEV1_FVC	0.423	2.25E-05	30.78	0.012	COPD	-0.003
rs12153431	5	131818657	T	C	IRF1-mediated FEV1_FVC	0.106	0.002	21.261	0.015	COPD	-0.037
rs71583468	5	131621431	A	G	IRF1-mediated FEV1_FVC	0.082	0.002	21.077	0.017	COPD	-0.013

rs72795177	5	131748768	A	G	IRF1-mediated FEV1_FVC	0.11	2.98E-05	30.173	0.018	COPD	-0.019
rs2073644	5	131723289	A	T	IRF1-mediated FEV1_FVC	0.456	3.37E-17	87.329	0.019	COPD	-0.047
rs162901	5	131620366	A	G	IRF1-mediated FEV1_FVC	0.107	2.69E-06	35.284	0.02	COPD	-0.01
rs555696853	5	132089661	T	G	IRF1-mediated FEV1_FVC	0.027	0.044	13.973	0.028	COPD	-0.026
rs7703152	5	131783213	T	C	IRF1-mediated FEV1_FVC	0.232	4.40E-28	138.839	0.029	COPD	-0.055
rs3914018	5	131637281	T	C	IRF1-mediated FEV1_FVC	0.013	0.003	19.981	0.04	COPD	-0.024
rs56034741	7	106415995	A	G	PIK3CG-mediated FEV1_FVC	0.217	0.025	15.335	-0.01	COPD	0.027
rs342182	7	106302691	T	C	PIK3CG-mediated FEV1_FVC	0.442	7.23E-04	23.281	0.01	COPD	-0.005

rs2392929	7	106414069	T	G	PIK3CG-mediated FEV1_FVC	0.202	1.15E-05	32.217	0.015	COPD	-9.99E-04
rs4894532	3	171974912	T	G	TNFSF10-mediated FEV1_FVC	0.368	9.44E-06	32.638	-0.012	COPD	0.009
rs9858290	3	171863992	A	C	TNFSF10-mediated FEV1_FVC	0.472	0.015	16.427	0.008	COPD	-0.002
rs62283816	3	171826840	A	G	TNFSF10-mediated FEV1_FVC	0.054	1.26E-11	60.98	0.036	COPD	-0.031
rs1187274	9	87419789	C	G	NTRK2-mediated FEV1_FVC	0.336	0.046	13.883	0.008	COPD	-0.012
rs71514273	1	26834960	A	G	SFN-mediated FEV1_FVC	0.252	3.56E-04	24.83	-0.012	COPD	0.009

COPD – chronic obstructive pulmonary disease, FDR – false discovery rate, FEV1/FVC – forced expiratory volume in one second/forced vital capacity, IV – instrumental variable, MAF – minor allele frequency, MR – Mendelian randomisation, SNP – single nucleotide polymorphism, β – beta coefficient.

Table S8. IVW estimates and Cochran's Q heterogeneity tests.

Exposure	Outcome	nSNPs	Outlier removed	IVW β (95% CI)	IVW OR (95% CI)	P-value	Cochran's Q	Q df	Q P-value
UACA-mediated FEV1/FVC	COPD	20	0	-1.24 (-1.64 - -0.85)	0.29 (0.19 - 0.43)	7.39E-10	23.603	19	0.212
IRF1-mediated FEV1/FVC	COPD	17	0	-1.66 (-2.15 - -1.18)	0.19 (0.12 - 0.31)	1.49E-11	22.551	16	0.126
PIK3CG-mediated FEV1/FVC	COPD	3	0	-0.73 (-2.15 - 0.69)	0.48 (0.12 - 2)	0.314	3.44	2	0.179
TNFSF10-mediated FEV1/FVC	COPD	3	0	-0.73 (-1.63 - 0.16)	0.48 (0.2 - 1.17)	0.106	0.231	2	0.891

CI – confidence interval, COPD – chronic obstructive pulmonary disease, df – degrees of freedom, FEV1/FVC – forced expiratory volume in one second/forced vital capacity, IVW – inverse-variance weighted, nSNP – number of single nucleotide polymorphisms, OR – odds ratio, SNP – single nucleotide polymorphism, β – beta coefficient.

Table S9. MR-Egger estimates and intercept tests for directional pleiotropy.

Exposure	Outcome	MR-Egger β (95% CI)	MR-Egger OR (95% CI)	P-value	Intercept	SE	HP P-value
UACA-mediated FEV1/FVC	COPD	-1.11 (-2.1 - -0.13)	0.33 (0.12 - 0.88)	0.04	-0.003	0.011	0.779
IRF1-mediated FEV1/FVC	COPD	-1.04 (-2.28 - 0.21)	0.35 (0.1 - 1.23)	0.123	-0.011	0.01	0.303
PIK3CG-mediated FEV1/FVC	COPD	2.99 (-4.82 - 10.8)	19.93 (8.11E-03 - 49018.54)	0.59	-0.044	0.046	0.516
TNFSF10-mediated FEV1/FVC	COPD	-1.05 (-2.73 - 0.63)	0.35 (0.07 - 1.89)	0.437	0.006	0.013	0.742

CI – confidence interval, COPD – chronic obstructive pulmonary disease, FEV1/FVC – forced expiratory volume in one second/forced vital capacity, MR – Mendelian randomisation, MR-Egger – Mendelian randomisation-Egger regression, OR – odds ratio, SE – standard error, β – beta coefficient.

Table S10. MR-PRESSO global test and outlier-corrected MR estimates.

Exposure	Outcome	RSSobs	Global P-value	Raw P-value	Raw β (95% CI)	Outlier-corrected P-value	Outlier-corrected β (95% CI)
UACA-mediated FEV1/FVC	COPD	25.929	0.295	6.44E-06	-1.242 (-1.331 - -1.154)		
IRF1-mediated FEV1/FVC	COPD	25.44	0.216	4.67E-06	-1.663 (-1.78 - -1.545)		
PIK3CG-mediated FEV1/FVC	COPD						
TNFSF10-mediated FEV1/FVC	COPD						

RSSobs and Global P-value represent the MR-PRESSO global test results. Raw P-value and β represent the MR estimates before outlier correction. Outlier-corrected P-value and β represent the MR estimates after removal of detected outliers.

CI – confidence interval, COPD – chronic obstructive pulmonary disease, FEV1/FVC – forced expiratory volume in one second/forced vital capacity, MR – Mendelian randomisation, MR-PRESSO – Mendelian Randomization Pleiotropy RESidual Sum and Outlier, RSSobs – observed residual sum of squares, β – beta coefficient.

Table S11. Causal estimates from weighted median and weighted mode analyses.

Exposure	Outcome	Weighted Median β (95% CI)	Weighted Median OR (95% CI)	Weighted Median (P-value)	Weighted Modeβ (95% CI)	Weighted Mode OR (95% CI)	Weighted Mode (P-value)
UACA-mediated FEV1/FVC	COPD	-1.08 (-1.63 - -0.54)	0.34 (0.2 - 0.58)	9.12E-05	-0.91 (-1.46 - -0.36)	0.4 (0.23 - 0.7)	0.004
IRF1-mediated FEV1/FVC	COPD	-1.83 (-2.46 - -1.21)	0.16 (0.09 - 0.3)	9.41E-09	-2.03 (-2.87 - -1.19)	0.13 (0.06 - 0.31)	2.25E-04
PIK3CG-mediated FEV1/FVC	COPD	-0.29 (-1.6 - 1.01)	0.75 (0.2 - 2.75)	0.661	-0.2 (-1.54 - 1.14)	0.82 (0.21 - 3.14)	0.798
TNFSF10-mediated FEV1/FVC	COPD	-0.79 (-1.79 - 0.2)	0.45 (0.17 - 1.22)	0.117	-0.82 (-1.88 - 0.24)	0.44 (0.15 - 1.27)	0.269

CI – confidence interval, COPD – chronic obstructive pulmonary disease, FEV1/FVC – forced expiratory volume in one second/forced vital capacity, MR – Mendelian randomisation, OR – odds ratio, β – beta coefficient.

Table S12. Statistical power and minimum detectable odds ratios for the causal outcomes.

Exposure	Outcome	nSNPs	R^2 (%)	F statistic (mean)	F statistic (min)	Cases, n	Total, n	OR	P-value	Power at observed OR	Minimum detectable OR (80% power)
IRF1-mediated FEV1/FVC	COPD	17	0.132	36.9	13.4	21,617	394,244	0.19	1.49e-11	**>0.999**	1.72
UACA-mediated FEV1/FVC	COPD	20	0.175	41.6	12.2	21,617	394,244	0.29	7.39e-10	**>0.999**	1.60
PIK3CG-mediated FEV1/FVC	COPD	3	0.013	20.4	11.1	21,617	394,244	0.48	0.314	0.220	5.64
TNFSF10-mediated FEV1/FVC	COPD	3	0.022	34.6	16.0	21,617	394,244	0.48	0.106	0.341	3.77

COPD – chronic obstructive pulmonary disease, FEV1/FVC – forced expiratory volume in one second/forced vital capacity, MR – Mendelian randomisation, nSNP – number of single nucleotide polymorphisms, OR – odds ratio, R^2 – proportion of variance explained, SNP – single nucleotide polymorphism.

Table S13. MVMR analyses adjusting for smoking-related genetic effects using lifetime and current smoking phenotypes.
Panel A. MVMR analysis adjusted for lifetime smoking

**Univariable IVW**			**MVMR-IVW**							**Pleiotropy & heterogeneity tests**	
**nSNPs**	**OR (95% CI)**	**P**	**nSNPs**	**F (FEV1/FVC)**	**F (smoking)**	**Direct effect of FEV1/FVC OR (95% CI)**	**P**	**Direct effect of lifetime smoking OR (95% CI)**	**P**	**MVMR-Egger intercept (P)**	**MVMR-Q (P)**
17	0.19 (0.12–0.31)	**1.49e-11**	17	9.63	2.21	**0.19 (0.12–0.30)**	**5.19e-06**	1.46 (0.78–2.72)	0.258	-0.0084 (0.440)	16.95 (0.259)
20	0.29 (0.19–0.43)	**7.39e-10**	20	7.33	1.31	**0.29 (0.19–0.43)**	**9.69e-06**	1.21 (0.63–2.31)	0.570	0.0015 (0.918)	21.49 (0.205)
3	0.48 (0.12–2.00)	0.314	3	19.50	1.71	0.50 (0.28–0.90)	0.259	3.62 (1.69–7.73)	0.186	-0.0207 (N/A)	0.16 (N/A)
3	0.48 (0.20–1.17)	0.106	3	0.83	0.55	0.54 (0.40–0.72)	0.148	1.65 (0.84–3.25)	0.382	0.0280 (N/A)	0.07 (N/A)

CI – confidence interval, FEV1/FVC – forced expiratory volume in one second/forced vital capacity, IVW – inverse-variance weighted, MVMR – multivariable Mendelian randomisation, MVMR-Egger – multivariable Mendelian randomisation-Egger regression, MVMR-Q – multivariable Mendelian randomisation Q statistic, N/A – not available, nSNP – number of single nucleotide polymorphisms, OR – odds ratio.

Panel B. MVMR analysis adjusted for current smoking

Univariable IVW			MVMR-IVW							Pleiotropy & heterogeneity tests	
**nSNPs**	**OR (95% CI)**	**P**	**nSNPs**	**F (FEV1/FVC)**	**F (smoking)**	**Direct effect of FEV1/FVC OR (95% CI)**	**P**	**Direct effect current of smoking OR (95% CI)**	**P**	**MVMR-Egger intercept (P)**	**MVMR-Q (P)**
17	0.19 (0.12–0.31)	**1.49e-11**	17	8.51	2.44	**0.19 (0.12–0.31)**	**6.81e-06**	1.26 (0.82–1.94)	0.310	-0.0095 (0.373)	17.51 (0.230)
20	0.29 (0.19–0.43)	**7.39e-10**	20	10.54	1.84	**0.31 (0.21–0.46)**	**1.50e-05**	1.28 (0.90–1.81)	0.185	-0.0046 (0.661)	18.96 (0.331)
3	0.48 (0.12–2.00)	0.314	3	0.21	0.18	0.53 (0.02–18.18)	0.785	1.18 (0.01–165.56)	0.957	-0.0999 (N/A)	3.27 (N/A)
3	0.48 (0.20–1.17)	0.106	3	9.37	0.57	0.45 (0.34–0.58)	0.108	1.40 (0.87–2.23)	0.396	0.0246 (N/A)	0.07 (N/A)

CI – confidence interval, FEV1/FVC – forced expiratory volume in one second/forced vital capacity, IVW – inverse-variance weighted, MVMR – multivariable Mendelian randomisation, MVMR-Egger – multivariable Mendelian randomisation-Egger regression, MVMR-Q – multivariable Mendelian randomisation Q statistic, N/A – not available, nSNP – number of single nucleotide polymorphisms, OR – odds ratio.

Supplementary Methods

1. Study design

A multi-stage pipeline was established to identify PANoptosis-related gene signatures and evaluate their roles in COPD susceptibility (**Figure 1**). Transcriptomic datasets were first analyzed to identify global differentially expressed genes, which were intersected with PANoptosis regulators and subsequently utilized to stratify patient molecular subtypes. Concurrently, comprehensive immune infiltration profiling was performed to characterize the immune microenvironment. Top feature genes with high discriminative performance were then selected using complementary machine learning algorithms. Next, the genetic causal relevance of these genes was evaluated through univariable MR using the FEV1/FVC ratio, followed by MVMR adjusting for smoking phenotypes to control for genetic confounding. Lastly, the transcriptional profiles of key feature genes were validated by qRT-PCR in human lung tissue specimens.

2. COPD data collection and preprocessing

Transcriptomic datasets were retrieved from the Gene Expression Omnibus (GEO) database (https://www.ncbi.nlm.nih.gov/geo/), encompassing five datasets: GSE151052, GSE37768, GSE103174, GSE38974, and GSE106986. These datasets are de-identified and publicly available and therefore did not require additional ethical

approval. Specifically, GSE151052, GSE37768, and GSE103174 were merged to serve as the discovery training set, whereas GSE38974 and GSE106986 were utilized as independent external cohorts to validate the predictive models (**Table 1**). During data preprocessing, microarray probes were mapped to official gene symbols, and probes without corresponding symbols were excluded. When multiple probes correspond to the same gene, the median expression value was calculated. Background correction and normalization were performed using the normalizeBetweenArrays method within each individual dataset. Batch effects were removed from the merged samples using the ComBat function from the "sva" package, with dataset source specified as the batch variable and disease status included as the biological variable to minimize technical variation while preserving disease-related signals. Principal component analysis (PCA) was performed before and after ComBat correction to assess data quality and batch-effect removal. After correction, all samples showed good clustering with no evident outliers; therefore, all samples were retained for subsequent analysis. Finally, the PANoptosis-related genes were referenced from a previous study [1].

3. Identification of DEGs

Differential gene expression analysis between COPD and control specimens in the merged dataset was conducted using the "limma" R package. Genes meeting the thresholds of an unadjusted *P*-value < 0.05 and $|\log_2 FC| > 0.5$ were defined as

DEGs. The false discovery rate (FDR) was calculated using the Benjamini-Hochberg method to control for multiple testing. To isolate disease-specific pyroptosis, apoptosis, and necroptosis signatures, these identified DEGs were intersected with the PANoptosis-related gene set. Crucially, the final PANoptosis-related DEGs all strictly adhered to the more stringent criterion of FDR < 0.05 and were retained for subsequent downstream analyses.

4. Enrichment analysis

Functional enrichment analyses of the DEGs were performed using the "clusterProfiler" R package. Both Gene Ontology (GO) terms, encompassing biological processes (BP), cellular components (CC), and molecular functions (MF), and Kyoto Encyclopedia of Genes and Genomes (KEGG) pathways were evaluated. *P*-values were adjusted for multiple testing using the Benjamini-Hochberg FDR method. Terms and pathways with an adjusted *P*-value < 0.05 were considered significantly enriched and retained for further interpretation.

5. Immune infiltration and correlation analyses

Immune cell infiltration drives COPD pathogenesis [2]. The enrichment scores of 28 immune cell subtypes were acquired using single-sample Gene Set Enrichment Analysis (ssGSEA), as previously reported [3]. The ssGSEA analysis was conducted using the "GSVA" package by transforming gene expression matrices between samples into gene set

expression matrices. Differences in immune cell abundance between two groups were assessed using the Wilcoxon rank-sum test.

To validate these immune infiltration patterns, CIBERSORT analysis was additionally performed using the LM22 immune cell signature matrix to deconvolute the expression profiles and estimate the relative proportions of 22 immune cell subsets. The CIBERSORT analysis was conducted with 1,000 permutations under standard mode, and only samples with CIBERSORT output *P*-value < 0.05 were included for subsequent analyses. After obtaining the immune cell fraction matrix, cell types with a relative abundance of zero across all samples were excluded. Differences in CIBERSORT-estimated immune cell infiltration between COPD and control groups were likewise assessed using the Wilcoxon rank-sum test.

To enhance the robustness of our conclusions, this study jointly utilized both independent methodologies. While ssGSEA reflects the functional activity enrichment levels of 28 immune signatures, CIBERSORT quantifies the relative abundance of cell subsets. Potential discrepancies between estimated cell proportions and functional activities were comprehensively interpreted by cross-referencing and aligning outputs from both algorithms. Given that the majority of variables did not strictly follow a normal distribution and the inherent relationships between gene expression and immune features might be non-linear, non-parametric Spearman correlation analysis was uniformly adopted. Specifically, Spearman correlation

analysis was used to examine the relationships between the identified feature genes and immune cell abundances, with a two-tailed *P*-value < 0.05 considered the threshold for biologically meaningful statistical significance.

6. Unsupervised clustering

Unsupervised consensus clustering was employed to identify distinct molecular subtypes of COPD patients based on the expression profiles of the identified differential PANoptosis-related genes in the merged dataset using the "ConsensusClusterPlus" R package. To ensure stability, the clustering algorithm was executed with 500 iterations (reps = 500), selecting 80% of the samples per iteration (pItem = 0.8) via agglomerative hierarchical clustering with Euclidean distance (distance = "euclidean"). The optimal number of subtypes (k) was determined by comprehensively evaluating the trend and smoothness of the Cumulative Distribution Function (CDF) curve, cluster consistency scores, and the tracking plot consensus matrix. Finally, the distribution and distinct clustering patterns of the determined patient subtypes were visualized and projected using t-distributed Stochastic Neighbor Embedding (t-SNE) and PCA implemented via the "Rtsne" R package.

7. Identification of feature genes

Feature genes were identified from the overlapping PANoptosis-related DEGs within the merged dataset using a consensus machine learning framework comprising three complementary algorithms: SVM-RFE, LASSO regression, and Random Forest (RF). Differentially expressed PANoptosis-related genes, identified from the merged and batch-corrected expression profiles, were utilized as the initial input gene set for the subsequent machine learning analyses.

First, SVM-RFE was performed using the "caret" R package leveraging a radial basis function (RBF) kernel for feature selection. This recursive algorithm sequentially eliminated unimportant variables to isolate the feature genes with the highest impact on classification. Specifically, the feature selection process was configured in rfeControl by specifying functions = caretFuncs, and method = svmRadial was implemented within the rfe function to predict the optimal feature subset. During this iterative feature elimination, a 10-fold cross-validation approach was adopted to rigorously evaluate model performance, ultimately selecting the optimal feature subset that exhibited the highest classification accuracy.

Second, to compute and select regularized linear models while retaining the most informative variables, Least Absolute Shrinkage and Selection Operator (LASSO) regression was executed using the "glmnet" R package. A binomial distribution error model was applied for the classification task. The regularized penalty parameter lambda was determined based on the 1-standard error (1-SE) criterion, which selects the lambda value at one standard error above the minimum mean

squared error as the minimum criterion for model construction. Genes retaining non-zero coefficients under this threshold were considered the optimal variables with robust predictive capability.

Third, the Random Forest algorithm was utilized to rank gene importance, where genes exhibiting a Gini coefficient (Mean Decrease Gini) greater than 1 were recognized as crucial features.

The final feature genes were defined by taking the strict intersection of the optimal gene subsets generated by all three machine learning algorithms. Finally, the diagnostic performance and discriminative capacity of these final feature genes across different datasets were evaluated and presented using receiver operating characteristic (ROC) curves via the "pROC" R package.

8. Protein-protein interaction (PPI) networks

The GeneMANIA database (http://www.genemania.org) was utilized to construct a PPI network to explore the functional connectivity among the identified feature genes and their closely related co-functional partners. Based on documented gene-gene interactions, GeneMANIA algorithmically retrieves and integrates baseline networks to identify additional genes that likely share biological functions with the input feature genes, thereby comprehensively unveiling the underlying molecular interaction landscapes.

9. Nomogram construction and evaluation

Nomogram construction and subsequent rigorous evaluation were performed using the "rms" R package. A predictive nomogram was built based on the feature genes and visualized using the regplot function from the "rms" package. To systematically assess the clinical decision-making performance and geometric accuracy of the constructed nomogram, calibration curves were generated utilizing the calibrate function within the "rms" package. Concurrently, Decision Curve Analysis (DCA) was executed using the "ggDCA" R package to determine the clinical usefulness and net benefit of the model across different threshold probabilities.

10. MR analysis

10.1 Data sources for MR analysis

An MR analysis was conducted to evaluate whether genetically predicted increases in FEV1/FVC, mediated by variants near the feature genes, are associated with COPD risk. Genome-wide association study (GWAS) summary statistics for the FEV1/FVC ratio were retrieved from the GWAS Catalog (ID: GCST90292611), encompassing 475,645 individuals and 53,205,680 SNPs, derived from a publication [4]. For clinical outcomes, GWAS data for COPD (J10_COPD, containing 21,617 cases and 372,627 controls) were sourced from the FinnGen R11 database. Within the FinnGen database, COPD

cases were identified via national registry data based on ICD-coded hospital discharge records and mortality registries. Crucially, granular clinical details regarding disease severity, such as the Global Initiative for Chronic Obstructive Lung Disease (GOLD) classification, are unavailable in the public GWAS summary statistics. Consequently, severity-stratified analyses could not be performed. Details on data sources are summarized in **Table S3**.

10.2 Selection of instrumental variables (IVs)

Single nucleotide polymorphisms (SNPs) located within ±500 kb genomic window of target genes were selected as potential IVs. To ensure the biological and statistical validity of the instruments, candidate IVs were rigorously filtered based on the following criteria: (i) significant association with the FEV1/FVC ratio (FDR < 0.05) [5]; (ii) minor allele frequency (MAF) $>$ 1%; (iii) F-statistic > 10 to exclude weak IVs; and (iv) low linkage disequilibrium (LD $r^2 < 0.3$) within a 500 kb clumping window, given the limited number of available *cis*-acting variants [6]. Variants with opposite effects on FEV1/FVC and COPD risk were retained to reflect the expected inverse relationship between lung function and disease risk.

10.3 MR analysis

Causal effect estimates between genetically proxied target perturbations and clinical outcomes were calculated using four complementary models: inverse-variance weighted (IVW), MR-Egger, Weighted Median, and Weighted Mode. The IVW

method served as the primary indicator for causal determination, while the remaining three models provided robust cross-verification. Causal associations satisfying this stringent threshold, FDR < 0.05, were defined as possessing strong evidence, whereas results meeting an unadjusted *P*-value < 0.05 were designated as yielding suggestive evidence. Furthermore, the Steiger directionality test (Steiger filtering) was implemented to mathematically confirm that the causal arrow unfailingly pointed from the exposure to the outcome, thereby precluding potential biases from reverse causality.

10.4 Sensitivity analysis and hierarchical outlier removal

To guarantee the reliability of causal inference, horizontal pleiotropy, instrument heterogeneity, and sensitivity were comprehensively evaluated. Instrument heterogeneity was examined using Cochrane's Q test based on the IVW model, supplemented and cross-verified by Cochrane's Q test derived from the MR-Egger regression model, while horizontal pleiotropy was rigorously evaluated using the MR-Egger intercept framework alongside the Mendelian Randomization Pleiotropy Residual Sum and Outlier (MR-PRESSO) global test. Sensitivity testing was further performed via a leave-one-out analysis, sequentially omitting each individual SNP to assess whether the global causal estimate was disproportionately driven by any single influential variant.

For outlier identification and rigorous data cleaning, Radial MR analyses based on both the IVW and MR-Egger regression models were implemented to quantify individual variant contributions to global heterogeneity via Cochran's Q and Rucker's Q statistics, and outlying SNPs were systematically eliminated using a strict hierarchical tier system: (i) Level 1 Outliers, defined as SNPs identified as outliers simultaneously in both Radial MR models, were prioritized for immediate exclusion, followed by a re-evaluation of heterogeneity; (ii) Level 2 Outliers, where SNPs identified as outliers in either of the two Radial MR models individually, were further removed if significant heterogeneity persisted; and (iii) Level 3 Outliers, which were ultimately eliminated based on the aberrant trajectories detected in the leave-one-out sensitivity analysis if heterogeneity remained unmitigated after Level 2 filtration, with the MR-PRESSO outlier test introduced as a supplementary validation tool to assist and cross-reference these radial adjustments.

10.5 Quantitative assessment of statistical power

To rigorously evaluate the reliability and statistical robustness of the identified MR findings, post-hoc statistical power analyses were systematically performed using the web-based mRnd platform. The cumulative variance explained (R^2) by the compliant cis-IVs post-hierarchical outlier removal, the empirical case-to-control numbers and total sample size within the COPD outcome cohort, and the observed effect sizes (odds ratios, ORs) were integrated to calculate the exact statistical

power under a nominal type I error rate of α = 0.05, thereby quantifying the reliability of our positive causal inferences. Furthermore, the minimum detectable OR (MDOR) at a standard 80% statistical power was computed to determine the lower limit of our model's effect resolution. This metric serves as the core benchmark for establishing the credibility of any negative (non-causal) findings, as an inflated MDOR value would explicitly highlight that moderate true genetic effects falling between the protective and risk thresholds might be statistically overlooked due to insufficient sample-driven power.

10.6 Statistical analysis

All MR analyses were executed in R software (version 4.3.0) utilizing the "TwoSampleMR" (version 0.4.26) and "RadialMR" (version 1.1) packages. Forest plots were constructed using the "forestplot" package (version 3.1.3). Scatter plots, leave-one-out plots, and funnel plots were produced using built-in functions within "TwoSampleMR" and "RadialMR".

11. Multivariable Mendelian randomization (MVMR) analysis

11.1 Data sources for MVMR analysis

To systematically evaluate whether the observed causal associations between target gene-mediated perturbations (FEV1/FVC ratio) and COPD risk were independent of smoking-related genetic confounding, MVMR analyses were introduced. Specifically, two questionnaire-based smoking phenotypes derived from the Million Veteran Program (MVP)

cohort were integrated into the multivariable framework as negative-control covariates: lifetime smoking ("In your lifetime, have you smoked a total of at least 100 cigarettes?") was utilized as the primary analysis covariate to reflect cumulative historical smoking exposure, whereas current smoking ("Do you currently smoke cigarettes?") served as the sensitivity analysis covariate to represent active smoking status [7]. The MVMR framework was executed across all evaluable exposure-outcome combinations among core candidate therapeutic targets and the clinical COPD outcome. Given that the three foundational data sources (GWAS Catalog, MVP, and FinnGen) originated from independent international biobanks, the degree of sample overlap was substantially minimal, effectively attenuating any potential biases towards observational associations.

11.2 Selection of IVs

Under the standard MVMR paradigm, a dual-exposure model was constructed, integrating target gene-mediated FEV1/FVC (X_1) and smoking behaviors (X_2) to jointly estimate their direct causal effects on the COPD outcome (Y). To preserve the baseline comparability and rigorous biological target-specificity, the MVMR framework strictly adhered to the identical cis-IVs retained after the hierarchical outlier removal in the prior primary univariate analyses. No extra *trans*-acting

smoking-associated variants from independent smoking GWASs were supplemented, ensuring that the instruments remained strictly anchored within the target gene regions.

Three-way genetic data harmonization was performed by mapping these compliant univariate cis-SNPs simultaneously across the exposure (FEV1/FVC, GCST90292611), the smoking covariates (MVP cohorts), and the outcome (FinnGen R11 database) summary datasets, retaining only overlapping variants. Allelic alignment and palindromic strand corrections were executed via the harmonise_data function within the "TwoSampleMR" package (action = 2).

Instrumental variable strength within the multi-exposure setting was rigorously evaluated using the Sanderson-Windmeijer (SW) conditional F-statistic, applying the conventional empirical threshold of $F > 10$ to define strong independent instrument capacity.

11.3 MVMR analysis and sensitivity analysis

The direct causal effect of each target-mediated exposure on COPD, conditioning on the fixed level of smoking covariates, was primarily estimated via the multivariable inverse-variance weighted (MVMR-IVW) method. To safeguard against potential directional horizontal pleiotropy, multivariable MR-Egger regression (MVMR-Egger) was employed as a secondary sensitivity check, with the intercept test quantifying persistent pleiotropic bias. Concurrently, multivariable

Cochrane's Q statistics (MVMR-Q tests) were systematically implemented to rigorously evaluate the underlying heterogeneity and overdispersion among the multi-exposure instrumental variables, thereby ensuring the overall statistical stability of the multivariable causal framework.

11.4 Statistical analysis

All MVMR calculations and diagnostic tests were executed in R software (version 4.3.0). Specifically, the "MVMR" package (version 0.4) was utilized to perform the primary analysis and diagnostic evaluations via its core functions, including format_mvmr, ivw_mvmr, strength_mvmr, and pleiotropy_mvmr. To execute the MVMR-Egger regressions and cross-validation, the "MendelianRandomization" package (version 0.9) was employed with the mr_mvinput and mr_mvegger functions. Additionally, all upstream data preprocessing and three-way harmonization were facilitated using the "TwoSampleMR" package (version 0.6).

12. Human subjects and clinical specimen collection

Non-tumorous lung tissue specimens were collected from patients undergoing lobectomy or pneumonectomy for lung cancer at the Affiliated Huai'an No.1 People's Hospital of Nanjing Medical University between January 10, 2025, and February 10, 2025. Based on preoperative spirometry, patients were classified as COPD (n = 6) or non-COPD controls (n

= 9). COPD was defined according to the Global Initiative for Chronic Obstructive Lung Disease (GOLD) criteria as a post-bronchodilator FEV1/FVC ratio < 0.70 [8].

Patients were strictly excluded if they presented with a history of asthma, alternative chronic respiratory disorders, active pulmonary infections, or documented autoimmune diseases. Additionally, individuals who had received systemic corticosteroids or immunosuppressive therapies within the past three months prior to surgery were deemed ineligible. Prior to enrollment, written informed consent was mandatory and obtained from all participants. This study was formally approved by the Institutional Review Board of the Affiliated Huai'an No.1 People's Hospital of Nanjing Medical University (Approval No.: KY-2025-003-01) and conducted in accordance with ethical guidelines.

13. Quantitative real-time polymerase chain reaction (qRT-PCR)

Total RNA was extracted using the FastPure Cell/Tissue kit (Vazyme, RC112) according to the manufacturer's protocol. RNA purity and concentration were assessed by ultraviolet (UV) spectrophotometry. Reverse transcription was performed using the HiScript III 1st strand cDNA synthesis kit (Vazyme, RC312) with genomic DNA removal at 42℃ for 2 min, followed by first-strand cDNA synthesis at 37℃ for 15 min and reaction termination at 85℃ for 5 sec. qRT-PCR was multiplexed on 96-well plates using the Taq Pro Universal SYBR qPCR Master Mix (Vazyme, Q712) on a calibrated qPCR detection

platform. Each sample was assayed in triplicate to ensure technical reproducibility, and target gene transcripts were normalized to the endogenous control *GAPDH*. Relative fold changes in gene expression were calculated using the comparative $2^{-\Delta\Delta Ct}$ method. The designated primer sequences for *PIK3CG*, *IL1B*, *UACA*, *SFN*, *IRF1*, *NTRK2*, *TNFSF10*, and *GAPDH* are provided in **Table S3**.

14. Statistical analysis

All statistical analyses and data visualizations were conducted using R software (version 4.3.0). Specifically, the "FactoMineR" and "factoextra" R packages were utilized for PCA and its subsequent visualization. Heatmaps were generated using the "pheatmap" package. The "ggvenn" package was employed for Venn diagram visualization. Receiver operating characteristic (ROC) curves were visualized using the "pROC" package, and feature gene correlation analysis was performed and visualized using the "ggpairs" package. Unless otherwise specified, plots were generated using "ggplot2" package or the base "plot" function in R.

Regarding hypothesis testing, the non-parametric Wilcoxon rank-sum test was employed to evaluate differences in continuous variables between two independent groups, whereas the one-way Analysis of Variance (ANOVA) was applied for comparisons among three groups. Based on the distributional features of the transcriptomic and immune data, non-

parametric Spearman correlation analysis was uniformly utilized to examine associations. For the experimental validation segment, qRT-PCR data were statistically analyzed and visualized using GraphPad Prism (version 8.0), with inter-group differences assessed via the two-tailed unpaired Student's *t*-test. A *P*-value < 0.05 was considered statistically significant. Statistical significance levels were conventionally designated as follows: * *P* < 0.05, ** *P* < 0.01, *** *P* < 0.001, and "ns" indicated no statistical significance.

Reference:

1. Song F, Wang C-G, Mao J-Z, Liang X-L, Zhang Y. PANoptosis-based molecular subtyping and HPAN-index predicts therapeutic response and survival in hepatocellular carcinoma. Frontiers in Immunology. 2023;14:1197152.
2. Son ES, Park J-W, Kim YJ, Jeong SH, Hong JH, Kim S-H, et al. Effects of antioxidants on oxidative stress and inflammatory responses of human bronchial epithelial cells exposed to particulate matter and cigarette smoke extract. Toxicology in Vitro. 2020;67:104883.
3. Charoentong P, Finotello F, Angelova M, Mayer C, Efremova M, Rieder D, et al. Pan-cancer immunogenomic analyses reveal genotype-immunophenotype relationships and predictors of response to checkpoint blockade. Cell reports. 2017;18(1):248-62.
4. Shrine N, Izquierdo AG, Chen J, Packer R, Hall RJ, Guyatt AL, et al. Multi-ancestry genome-wide association analyses improve resolution of genes and pathways influencing lung function and chronic obstructive pulmonary disease risk. Nature genetics. 2023;55(3):410-22.

5. Chen Z, Wang X, Teng Z, Huang J, Mo J, Qu C, et al. A comprehensive assessment of the association between common drugs and psychiatric disorders using Mendelian randomization and real-world pharmacovigilance database. EBioMedicine. 2024;107.
6. Yang Y, Lorincz-Comi N, Li M, Zhu X. A multivariable cis-Mendelian randomization method robust to weak instrument bias and horizontal pleiotropy bias. Briefings in Bioinformatics. 2025;26(3):bbaf250.
7. Gaziano JM, Concato J, Brophy M, Fiore L, Pyarajan S, Breeling J, et al. Million Veteran Program: A mega-biobank to study genetic influences on health and disease. Journal of clinical epidemiology. 2016;70:214-23.
8. Hoesein FAM, Zanen P, Lammers J-WJ. Lower limit of normal or FEV1/FVC< 0.70 in diagnosing COPD: an evidence-based review. Respiratory medicine. 2011;105(6):907-15.
